# Catalytic Enantioselective Elimination of Cyclobutanols

**DOI:** 10.1021/jacs.5c22741

**Published:** 2026-04-15

**Authors:** Pravin Kumar, Howard Díaz-Salazar, Anna Iagarmina, Łukasz Woźniak

**Affiliations:** † Faculty of Chemistry, 37799 Jagiellonian University, Gronostajowa 2, 30-387 Kraków, Poland; ‡ Doctoral School of Exact and Natural Sciences, Jagiellonian University, Prof. St. Łojasiewicza 11, 30-387 Kraków, Poland

## Abstract

Brønsted acid-mediated elimination of alcohols is a fundamental transformation in organic chemistry, yet its application in enantioselective catalysis remains largely unexplored. Here, we report a catalytic, enantioselective elimination of cyclobutanols and their trichloroacetimidate derivatives that afford cyclobutenes bearing all-carbon quaternary stereocenters. Mechanistic studies revealed that a chiral Brønsted acid catalyst mediates concerted, enantioselective elimination of cyclobutanols, whereas their trichloroacetimidates undergo stepwise elimination via a catalyst-substrate covalent intermediate. This research lays the foundation for further investigations of enantioselective elimination reactions.

## Graphical abstract

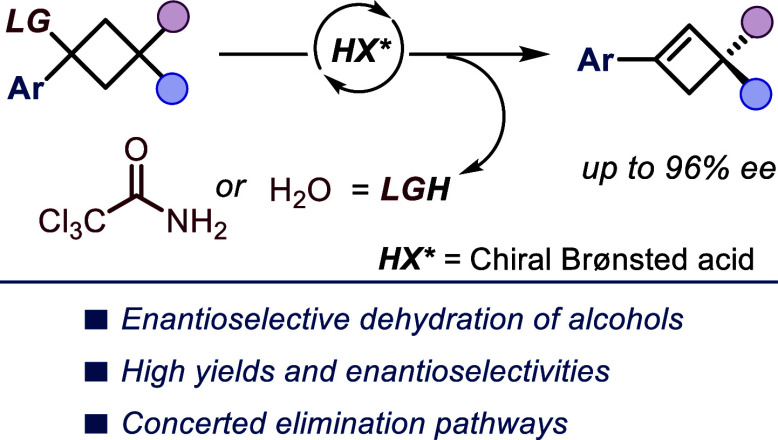

Carbocations are central to molecular synthesis.^1^ These reactive intermediates play a pivotal role in the synthesis of numerous pharmaceuticals, natural products, and materials.^2^ Exerting precise enantiocontrol over a broad range of carbocation-mediated reaction classes, however, remains challenging.^3^ Over the last decades, the development of catalytic enantioselective reactions involving carbocations has been at the forefront of organic synthesis. Consequently, numerous enantioselective additions,^4^ substitutions,^5,6^ rearrangements,^7^ olefin hydrofunctionalizations^8^ and pericyclic reactions^9^ now constitute powerful strategies for constructing optically active molecules (Figure 1A). Moreover, Brønsted acid activation of strained rings has recently emerged, enabling isomerization^10^ and fragmentation^11^ reactions that furnish olefins featuring neighboring tertiary stereocenters (Figure 1B). In contrast, one of the most general approaches to olefin formation remains the unimolecular elimination (E1). However, being competitive with unimolecular substitution (S_N_1) or rearrangements, E1 elimination has been largely an undesired side reaction in asymmetric catalysis (Figure 1C). Although E1-like mechanism operates in enantioselective amine–carbonyl condensations,^12^ or in synthesis of enol silanes,^13^ a direct enantioselective elimination of simple alcohols remains unrealized.

**Figure 1:**
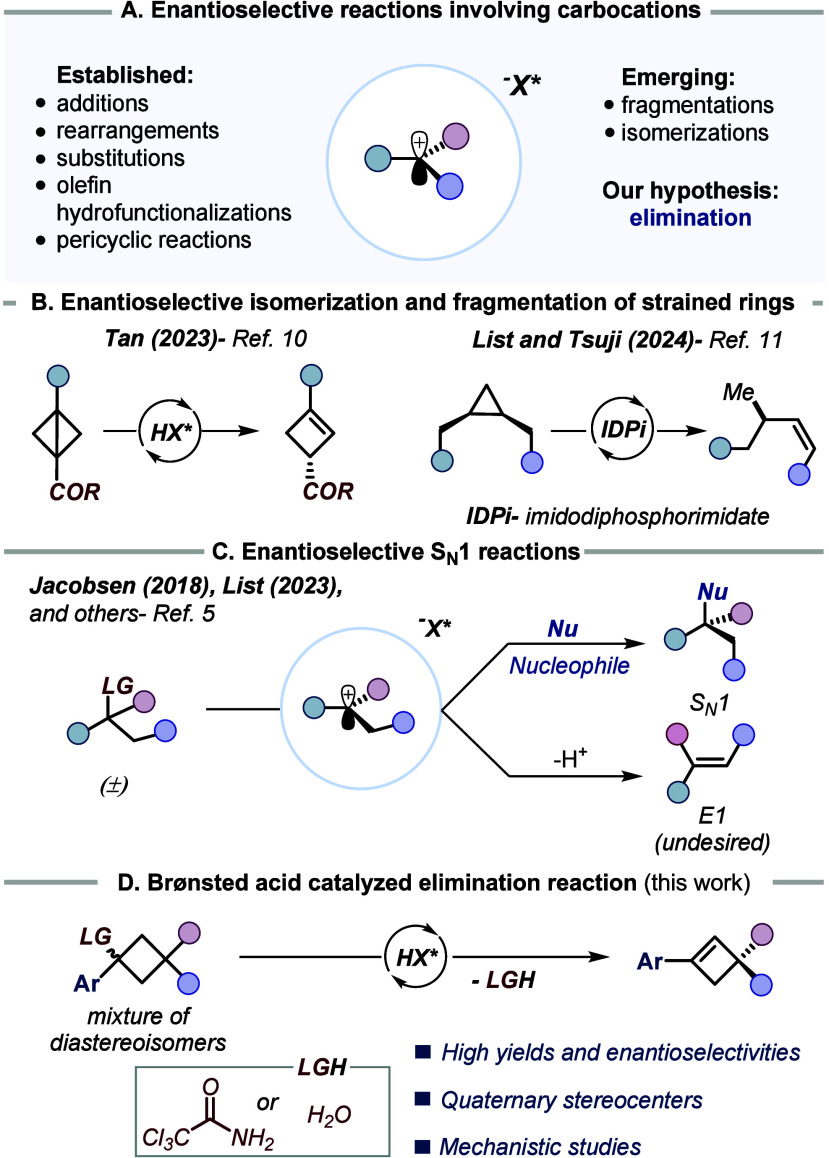
(A) Prochiral carbocations as intermediates in diverse enantioselective reactions. (B) Activation of strained rings by chiral Brønsted acids leading to olefinic products. (C) Enantioselective S_N_1 reaction and competing elimination. (D) Enantioselective elimination of cyclobutanols. LG: leaving group.

We questioned whether chiral Brønsted acid catalysts, which exert enantiocontrol over the carbocation in S_N_1 reactions, would influence the stereochemistry of the elimination reaction (Figure 1D). We initially anticipated that the elimination of benzylic tertiary alcohols would primarily proceed via E1 mechanism. Therefore, the catalytic system would need to overcome several key challenges: (a) differentiating between the two faces of high-energy carbocationic intermediates in the absence of a nucleophile, (b) minimizing undesired substitution with the leaving group and competing rearrangement pathways, and (c) selectively generating carbocations from the substrate but not from the olefinic product. Accordingly, we expected that fine-tuning both the catalyst’s chiral environment and its acidity would be critical for realizing this transformation.

Our approach toward catalytic enantioselective elimination reaction involves desymmetrization of cyclobutanols to afford cyclobutenes bearing all-carbon quaternary stereocenters. The cyclobutene ring is a prevalent motif in biologically active molecules and natural products,^14^ and serves as a valuable synthetic building block.^15^ Although several strategies for the synthesis of enantioenriched cyclobutenes have been reported,^16^ organocatalytic approaches remain underdeveloped. Voituriez and co-workers reported a phosphine-catalyzed enantioselective Michael addition/Wittig sequence that affords densely substituted cyclobutenes.^17^ Organocatalytic [2 + 2] cycloadditions between alkynes and alkenes have also been shown to produce ring-fused cyclobutenes.^18^ Recently, Tan developed an elegant Brønsted acid-catalyzed isomerization of bicyclo[1.1.0]­butanes (BCBs).^10^ The isomerization reaction, however, does not enable the formation of quaternary stereocenters. We envisioned that exerting enantiocontrol over the elimination reaction of cyclobutanols would provide direct access to enantioenriched cyclobutenes, thus revealing the untapped potential of this long-established reactivity in asymmetric catalysis. Herein, we report a realization of this idea.

We investigated the feasibility of an enantioselective elimination reaction using a diastereomeric mixture of cyclobutanol **1a** in the presence of phosphate-derived chiral Brønsted acids. Representative results from our extensive studies are presented in Table 1, and additional details can be found in the Supporting Information (SI). Performing the reaction with phosphoric acid **C1** in *m*-xylene at room temperature furnished product **2a** in high yield and with low enantiomeric excess after 48 h (Table 1, entry 1). Variation of the aromatic substituents at the catalyst’s 3,3′-positions did not improve the reaction outcome (entries 2 and 3), but changing the catalyst skeleton to the SPINOL-derived chiral phosphoric acid **C4** resulted in improved enantioselectivity (entry 4). Intriguingly, shortening the reaction time had a positive effect on the enantiomeric excess (entries 4–6), delivering the cyclobutene with an improved 68% ee after 16 h; however, the yield decreased to 52%. We initially attributed this outcome to slow product racemization occurring via protonation of the cyclobutene by the catalyst. The significantly more acidic *N*-triflyl phosphoramide^19^ **C5** delivered the product quantitatively but without enantiocontrol, presumably due to rapid racemization (entry 7). Gratifyingly, decreasing the temperature restored enantioselectivity (entries 8 and 9), affording the product with 89% ee at −30 °C. Finally, performing the reaction in mesitylene and increasing the reaction time to 24 h delivered cyclobutene **2a** in 81% yield and 90% ee (entry 11). Further prolonging the reaction time resulted in a slight decrease in yield and enantiomeric excess (entry 12).

**Table 1:**
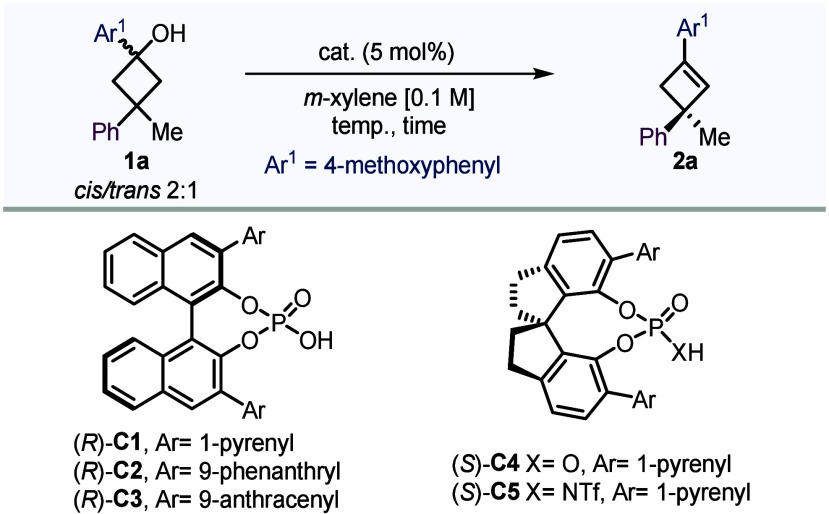
Optimization Studies^a^

entry	cat.	temp.	time (h)	yield (%)	ee (%)
1	**C1**	r.t.	48	86	27
2	**C2**	r.t.	48	91	20
3	**C3**	r.t.	48	30	31
4	**C4**	r.t.	48	76	56
5	**C4**	r.t.	24	75	60
6	**C4**	r.t.	16	52	68
7	**C5**	r.t.	16	99	0
8	**C5**	–10 °C	16	99	65
9	**C5**	–30 °C	16	80	89
10^b^	**C5**	–30 °C	16	62	91
11^b^	**C5**	–30 °C	24	84 (81)^c^	91 (90)^c^
12^b^	**C5**	–30 °C	40	80	89

a Reactions performed on a 0.06 mmol scale using 0.6 mL of *m*-xylene. Yields of **2a** determined by ^1^H NMR analysis of the crude mixture using trichloroethylene as the internal standard. Enantiomeric excess measured using HPLC analysis on a chiral stationary phase.

b Reaction in mesitylene,

c Reaction scale: 0.10 mmol, provided isolated yield.

We then evaluated the synthetic potential of the enantioselective elimination reaction (Figure 2). Initially, we observed that, for most substrates, increasing the reaction time to 48 h significantly improved the yield without compromising the enantioselectivity (see the SI). Cyclobutenes bearing quaternary stereocenters with diverse *para*-substituted phenyl rings **2b**-**2f** were obtained in high enantioselectivity. Cyclobutanol **1g**, bearing a nitro substituent, required a higher catalyst loading to furnish **2g** in good yield, albeit with reduced enantioselectivity. *Ortho*-substituents were also well tolerated, delivering products **2i** and **2j** with good yields and 90–92% ee. Cyclobutenes bearing *meta*-substituted phenyl **2m** and **2n** were obtained with high yields and enantioselectivities (91–92% ee), while naphthyl substituted products **2k** and **2l** formed with 86–91% ee. Notably, cyclobutanol containing 1,3-benzodioxole moiety delivered cyclobutene **2o** with 88% yield and 90% ee. Cyclobutene **2h** was obtained in moderate enantiomeric excess, underscoring the importance of the methyl substituent at the quaternary stereocenter for achieving high enantioselectivity.

**Figure 2:**
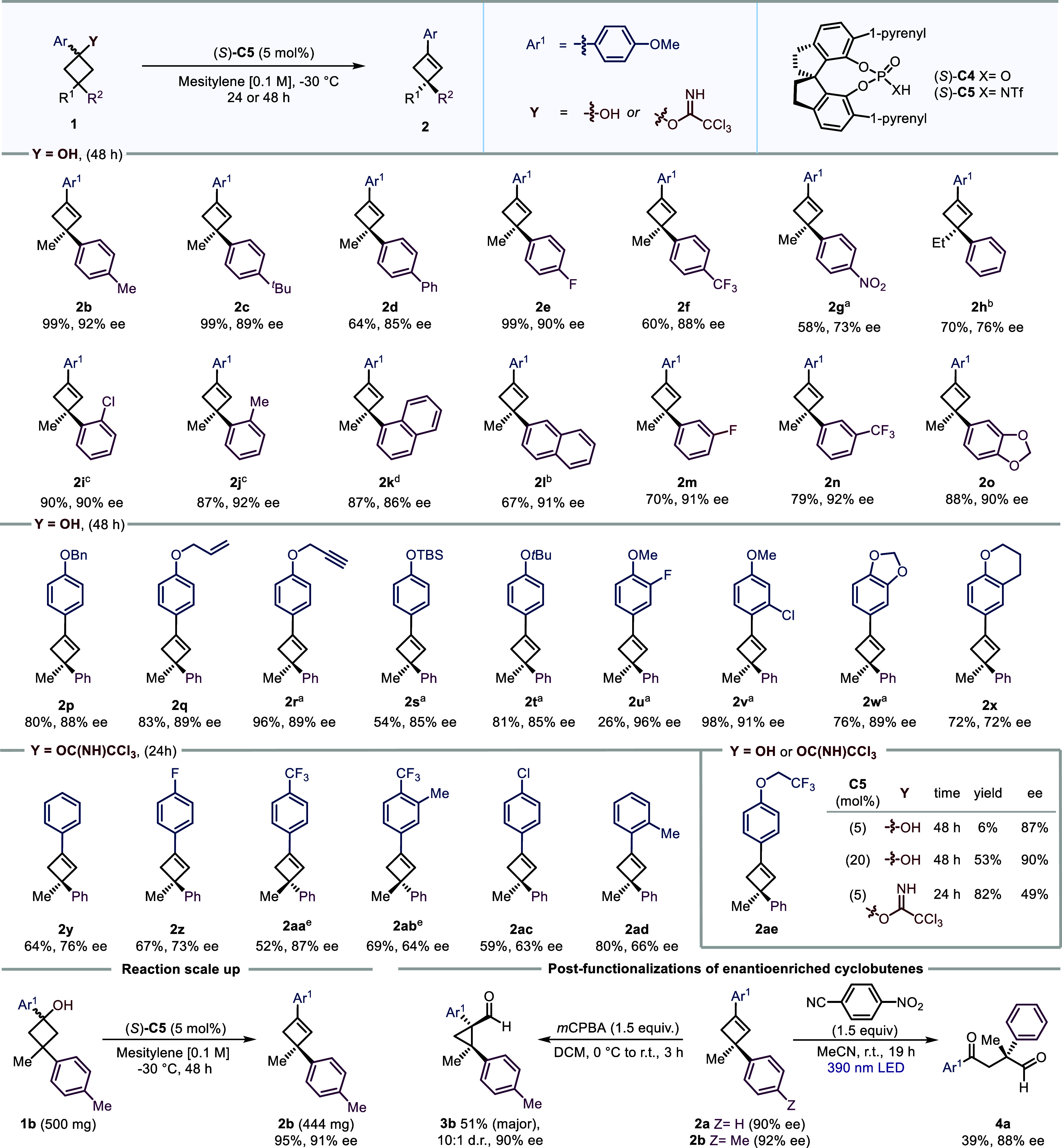
Scope and synthetic potential of enantioselective elimination reaction. All reactions were conducted on a 0.1 mmol scale using mixture of *cis/trans* isomers of **1** unless noted. Yields of isolated products are provided. ee values were determined using chromatographic methods (see the SI). Legend: ^*a*^using 20 mol % of catalyst loading; ^*b*^reaction time 24 h; ^*c*^using *trans*-diastereoisomer as a substrate; ^*d*^using *cis*-diastereoisomer as a substrate; ^*e*^using 5 mol % of **C4**.

As expected, the reaction is more sensitive to the properties of the substituents of the tertiary alcohol. Cyclobutanols possessing 4-benzyloxy and -allyloxy phenyls **1p** and **1q** underwent elimination with high yields and enantioselectivity. Products with other alkoxy and silyloxy substituents **2r**–**2t** formed with good yields and high enantioselectivities upon increasing the catalyst loading to 20 mol %. The same conditions were required for cyclobutanols **1u** and **1v**, which bear halogen substituents on the aryl ring in addition to a 4-methoxy group, as well as for **1w**, which contains a 1,3-benzodioxole moiety. The *meta*-fluoro substituent significantly diminishes the reactivity of **1u**; nonetheless, the cyclobutene was formed with excellent 96% ee. Cyclobutene **2x**, containing a chromane scaffold, was obtained in good yield, however with a lower enantiomeric excess (72% ee). Cyclobutanols lacking *para*-alkoxy aryl groups are unreactive in our standard reaction conditions. Therefore, we prepared 2,2,2-trichloroacetimidates **1y**–**1ad** as more easily ionized cation precursors. These highly reactive substrates were fully consumed within 24 h. The reaction enantioselectivity, however, was strongly responsive to substrate modifications, delivering products **2y**–**2ad** with moderate to good enantiomeric excesses (63–87% ee). Interestingly, cyclobutenes **2aa** and **2ab** bearing a *para*-trifluoromethyl substituent were obtained in higher enantiomeric excess when phosphoric acid **C4** was employed as the catalyst (see the SI). Cyclobutene **2ae** was accessed from both the corresponding cyclobutanol and its trichloroacetimidate derivative. Although the latter undergoes reaction more rapidly, it furnishes the product with markedly diminished enantiomeric excess (vide infra). To further assess the synthetic utility of the enantioselective elimination, we carried out an 18-fold scale-up of the reaction, which afforded cyclobutene **2b** with only a slight reduction in yield and enantioselectivity. Subsequent oxidative ring contraction^20^ furnished cyclopropane **3b** in 51% yield with good diastereoselectivity and well-maintained enantiomeric integrity of the substrate. Furthermore, oxidative cleavage of **2a** with a nitroarene under purple-light irradiation^21^ afforded 1,4-dicarbonyl **4a** in moderate yield.

Contrary to our initial hypothesis, the pronounced dependence of enantioselectivity on the nature of the leaving group indicates that the reaction does not proceed via an E1 mechanism or that distinct mechanisms may be operative depending on the leaving group. As the extent of reactant deprotonation in the rate-determining step is a distinguishing feature of different elimination mechanisms, we conducted a series of kinetic studies. The reaction orders with respect to the substrate and catalyst were investigated using variable time normalization analysis (VTNA).^22^ The initial analysis was performed using cyclobutanol **1a** in the presence of chiral phosphoric acid **C4** at room temperature. The normalized curves overlapped best when assuming a fractional-order dependence on the substrate (Figure 3A) and a first-order dependence on the catalyst (see the SI). Further, we performed an equimolar intermolecular competition experiment between **1a** and **1a-D** (Figure 3B). The KIE of 2.1 indicates that C–H bond cleavage is rate-limiting. The same analysis was performed using the most selective catalyst, *N*-triflyl phosphoramide **C5**, at −10 °C, revealing zero-order dependence on **1a** at low substrate concentrations, along with first-order dependence on **C5**. Furthermore, a significantly larger KIE of 7.6 was observed, consistent with values reported for E2 (bimolecular elimination) reactions.^23^ Additionally, no catalyst–substrate covalent adducts were detected by ^31^P NMR spectroscopy over the course of both reactions. This suggests that elimination of cyclobutanols with both catalysts—**C4** at room temperature and **C5** at −30 °C—proceeds via an E2 mechanism, with a significantly different extent of deprotonation in the transition state. A substrate order lower than 1 could be attributed to concurrent side reactions of cyclobutanol, such as epimerization via an S_N_1 pathway or to the formation of off-cycle species such as unreactive hydrogen-bonded aggregates of **1a**.^24^ To evaluate the former, we performed eliminations of diastereomerically pure cyclobutanols **1i-** *trans* and **1i-** *cis*. Cyclobutene **2i** was formed with comparable yields and enantioselectivity (Figure 3D). In both cases, a mixture of **1i-** *cis* and **1i-** *trans* was isolated after 48 h. Monitoring the reaction progress by ^1^H NMR spectroscopy further confirmed that background substrate epimerization competes with elimination. The reaction profiles also revealed that *cis* and *trans* diastereoisomers are consumed at different rates, which is consistent with concerted elimination pathway.^25^ Furthermore, the absence of a nonlinear effect^26^ suggests that a single catalyst molecule is involved in the transition state of the enantiodetermining step (Figure 3C). Although the dependence of enantioselectivity on the leaving group could be rationalized by the E2 mechanism, we carried out further studies with trichloroacetimidate **1ag**. Using catalyst **C4** at room temperature, we observed rapid initial consumption of the substrate to an extent approximately equal to the catalyst concentration, followed by slow formation of the product (zeroth order in **1ag**, Figure 3A). Further, first order dependence of the reaction rate on the concentration of **C4** was observed. Analysis using **C5** at −20 °C revealed a zero-order dependence on **1ag** at low substrate concentrations, as well as a negative-order dependence on **C5**. The latter could be attributed to the formation of hydrogen-bonded catalyst aggregates, which may constitute an off-cycle catalyst reservoir.^27^ Moreover, the absence of a nonlinear effect in the formation of **2z** (see the SI) suggests that a single molecule of catalyst is involved in the enantiodetermining and rate-limiting deprotonation. Following the reaction progress by ^31^P NMR spectroscopy also pointed to different behavior of the catalyst with respect to the elimination of cyclobutanols. No catalyst peak was observed after mixing **1ag** with **C4**. Instead, new peaks appeared on ^31^P NMR spectra, which vanished at the end of the process along with the emergence of the signal corresponding to the catalyst (Figure 3F). This suggests involvement of a covalently linked substrate–catalyst intermediate, consistent with studies of chiral Brønsted acid-catalyzed reactions of trichloroacetimidates,^28^ acetals^29^ or olefins.^8a,30^ We tentatively assigned the new signals to two diastereomers of **IV**. An analogous observation was made using catalyst **C5** at low temperatures (see the SI). HRMS analysis further supports the formation of the proposed intermediate as a new peak at *m*/*z* 953, corresponding to **IV**, appears during the course of the reaction. Moreover, Hammett analysis with a series of trichloroacetamide derivatives did not give a linear correlation with substituent parameters σ nor σ^+^. This, together with the KIE of 1.5–2.1, further supports a mechanism that does not involve rate-limiting formation of a carbocationic intermediate. Instead, a concerted rate- and enantiodetermining elimination from **IV** might be operative.

**Figure 3:**
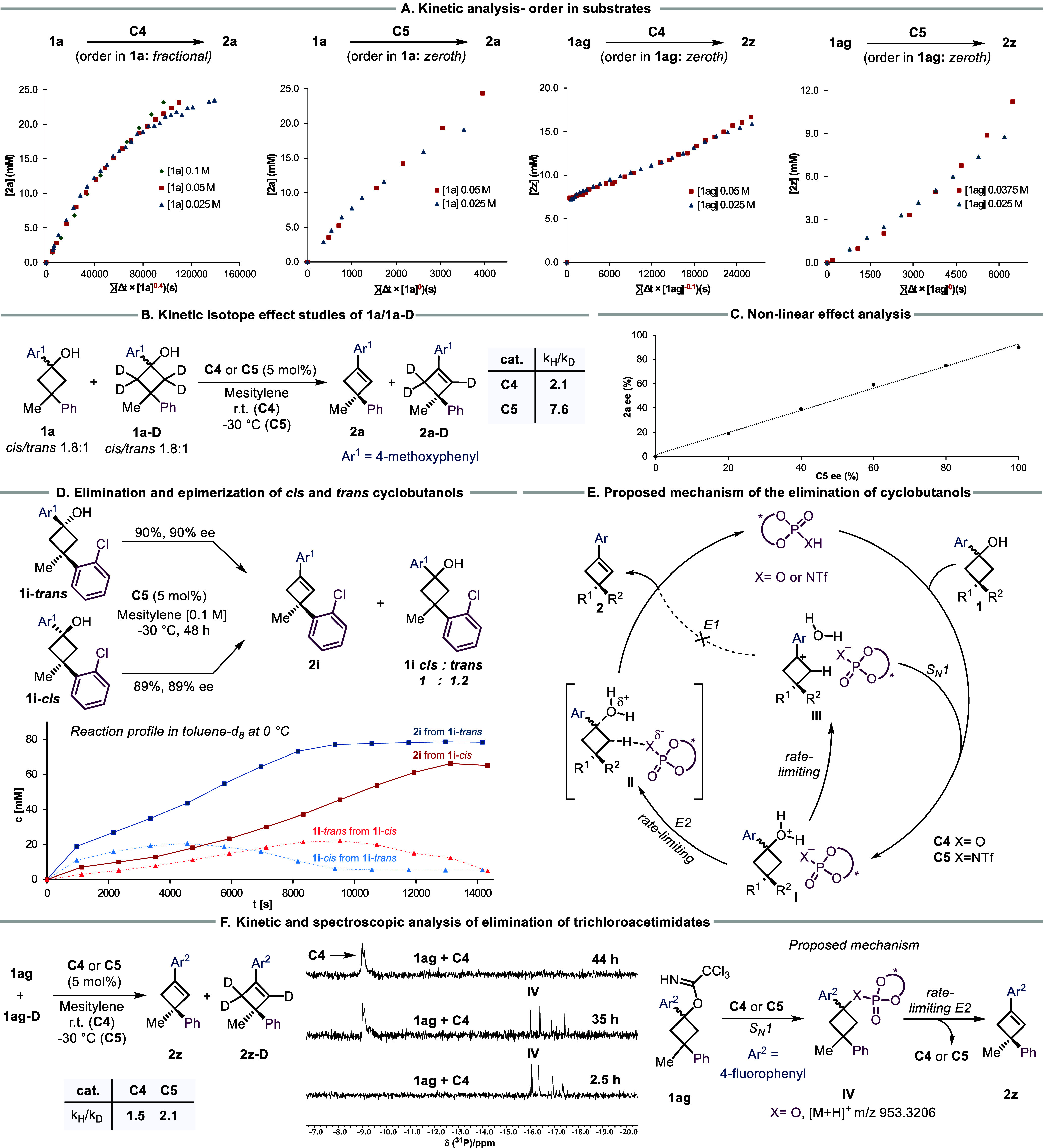
(A) Reaction orders in substrates determined using VTNA. (B) Competitive KIE study of cyclobutanol elimination. (C) Non-linear effect study. (D) Reactions employing diastereomerically pure substrates. (E) Proposed E2 elimination mechanism with concurrent substrate epimerization. (F) Mechanistic study of the elimination of trichloroacetimidates.

In summary, we have developed a catalytic enantioselective elimination of cyclobutanols that unlocks the untapped potential of simple alcohol dehydration for the construction of new stereogenic centers. We have demonstrated that chiral Brønsted acids convert diastereomeric mixtures of cyclobutanols and their corresponding trichloroacetimidates into cyclobutenes with good to high enantiomeric excess. Mechanistic studies revealed that the reaction does not proceed via the E1 mechanism, instead, concerted elimination pathways are operative. The strategy described herein may be generalizable to the construction of diverse stereogenic elements.

## Supplementary Material



## Notes

Experimental procedures, optimization studies, mechanistic studies, characterization data and NMR spectra of new compounds (PDF)
