# Supplementary material for: Catalytic Enantioselective Elimination of Cyclobutanols
Source: J Am Chem Soc. 2026 Apr 15;148(16):16568–75. doi: 10.1021/jacs.5c22741 (PMC13298886; doi:10.1021/jacs.5c22741)
Supplement: Supplementary file 1 [file ja5c22741_si_001.pdf]

Catalytic Enantioselective Eliminaion of Cyclobutanols

Pravin Kumar,[a] Howard Díaz Salazar,[a] Anna Iagarmina,[a,b] Łukasz Woźniak[a*]

a. Faculty of Chemistry, Jagiellonian University, Gronostajowa 2, 30-387 Kraków, Poland, b. Doctoral School of Exact and Natural Sciences, Jagiellonian University, Prof. St. Łojasiewicza 11, 30-387 Kraków, Poland.

Supporting Information

Table of Contents

General Information 3

Preparation of substrates 4

General Scheme for the Preparation of Cyclobutanols 4

Synthesis of cyclobutanones 4

General procedure A for the synthesis of cyclobutanols 7

General procedure B for the synthesis of cyclobutanols 7

General procedure C for the synthesis of trichloroacetimidates 23

Enantioselective elimination of cyclobutanols 30

Optimization studies 30

General procedure D for enantioselective elimination of cyclobutanols 33

General procedure E for enantioselective elimination of cyclobutyl trichloroacetimidates 34

Unsuccessful and moderately successful substrates 67

Reaction scale-up and post-functionalizations 67

Mechanistic studies 71

^{1}H NMR Reaction monitoring. 71

Experiments with the isolated*cis* and *trans* substrates 73

Reaction order determination 74

Monitoring the reaction progress by ^{31}P NMR 86

ESI-MS Studies 90

Reactions with nucleophilic additives 95

Hammett analysis 98

Racemization studies 101

Non-Linear effect analysis 101

References 103

NMR spectra 104

General Information

All commercially available compounds were used without purification. Unless otherwise noted, all reactions were performed in oven-dried glassware. All reactions were run under an argon or nitrogen atmosphere. Proton nuclear magnetic resonance (^{1}H NMR) data were acquired on JEOL JNM-ECZ400S 400 MHz, JEOL JNM-ECZ500R 500 MHz, or JEOL JNMECZ600S 600 MHz spectrometer at 298 K unless otherwise noted. Chemical shifts (δ) are reported in parts per million (ppm) relative to incompletely deuterated $CDCl_3$ (7.26 ppm), C_6D_6 (7.16 ppm), or toluene-d_8 (2.09 ppm). Splitting patterns are designated as s, singlet; d, doublet; t, triplet; q, quartet; m, multiplet; br, broad. Proton-decoupled ^{13}C nuclear magnetic resonance ($^{13}C\{^{1}H\}$ NMR) data were acquired on a JEOL JNM-ECZ400S 400 MHz or JEOL JNM-ECZ600S 600 MHz spectrometer at 298 K. Chemical shifts are reported in ppm relative to residual solvent peaks in $CDCl_3$ (77.16 ppm), C_6D_6 (128.06 ppm). ^{19}F nuclear magnetic resonance (^{19}F NMR) data were acquired at JEOL JNM-ECZ400S 400 MHz spectrometer at 298 K. Infrared (IR) spectra were recorded with a Fourier transform infrared (FTIR) spectrometer, ThermoFisher Scientific Nicolet iS5 with ATR iD7, and are reported in wavenumbers (cm^{-1}). High-resolution mass spectra (HRMS) were recorded with an electrospray ionization time-of-flight (ESI-TOF) on Bruker Daltonics micrOTOF-Q II mass spectrometer. Optical rotations were measured at room temperature with a polarimeter JascoP-2000 using a 5.0 cm cell with a Na 589 nm filter. Determination of enantiomeric purity was performed by HPLC analysis on a chiral stationary phase in on Agilent 1260 Infinity III series instrumentation, or Knauer advanced scientific instruments with Daicel Chiralpak IA, IB, IC, ID, or IF, AD-H, or OJ-H columns, and hexane, i-PrOH, and/or DCM as the eluents.

Preparation of substrates

General Scheme for the Preparation of Cyclobutanols

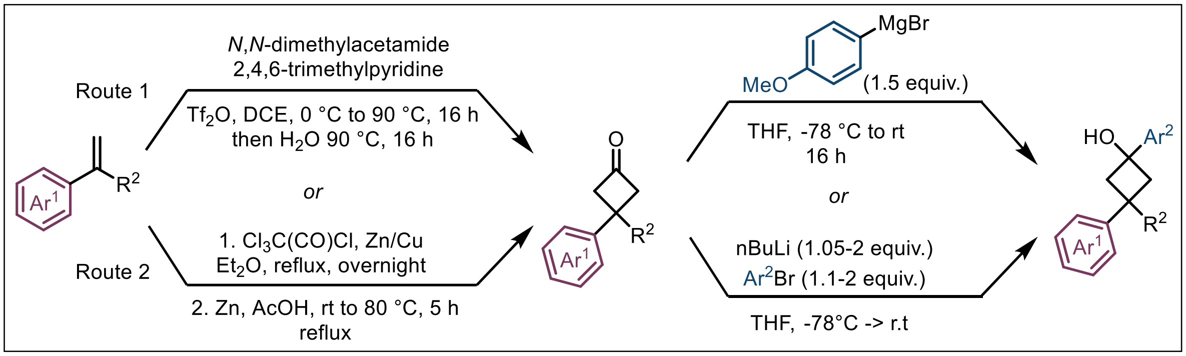

Synthesis of cyclobutanones

Cyclobutanones S1,[1] S2,[2] S3[3,4], S4[3,4], S5[4], S6[5], S7[1], S8[6], S9, S10[2], S11[3], S12[2,6], and S13[7] were previously reported and synthesized following the reported procedure.[8,9]

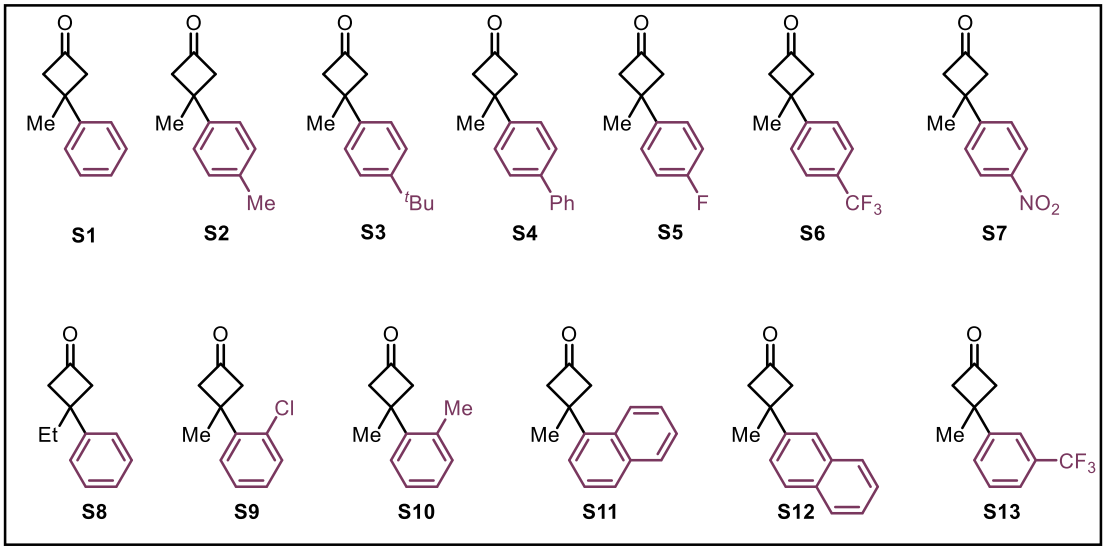

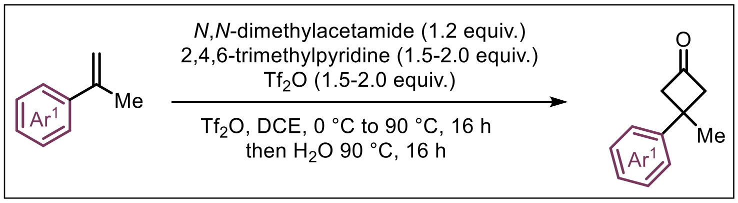

A Schlenk tube was charged with *N*,*N*-dimethylacetamide (1.2 equiv.) in 1,2-dichloroethane (DCE) (0.5M). The reaction solution was kept at room temperature in a water bath. Tf_2O (1.5-2.0 equiv.) was added dropwise *(observation of a white precipitate)* and the reaction mixture was stirred at room temperature for 30 min. A mixture of styrene (1.0 equiv.) and 2,4,6-trimethylpyridine (1.5-2.0 equiv.) in 1,2-dichloroethane (2M) was then added dropwise to the reaction mixture *(observation of a solution colored rose, red, and finally dark)*, which was then stirred at 90°C overnight. The reaction was allowed to cool to room temperature, and water was added. The reaction mixture was stirred at 90°C for an additional 24 h. The mixture was allowed to cool to room temperature, and the layers were separated. The aqueous layer was extracted with DCM (3x50mL). The combined organic layers were dried over $MgSO_4$, filtered, and the solvent was removed under reduced pressure. The residue was purified by flash column chromatography to yield the desired product.

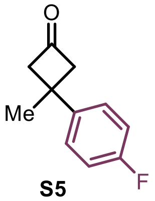

S5

3-(4-fluorophenyl)-3-methylcyclobutan-1-one (S5). *N*,*N*-dimethylacetamide (768 mg, 0.82 mL, 8.81 mmol, 1.2 equiv.), Tf_2O (3.11 g, 1.85 ml, 11.0 mmol, 1.5 equiv.), 1-fluoro-4-(prop-1-en-2-yl)benzene (1.0 g, 1.0 ml, 7.34 mmol, 1.0 equiv.), 2,4,6-trimethylpyridine (1.33 g, 1.46 ml, 11.0 mmol, 1.5 equiv.). The crude mixture was purified by column chromatography (EtOAc-hexane, 1-5%) to afford the corresponding cyclobutanone as a yellow oil in 67% yield (900 mg, 5.05 mmol).

^{1}H NMR (400 MHz, $CDCl_3$): δ 7.29 – 7.21 (m, 2H), 7.07 – 7.00 (m, 2H), 3.47 – 3.37 (m, 2H), 3.15 – 3.06 (m, 2H), 1.58 (s, 3H) ppm; **^{13}C NMR (101 MHz, $CDCl_3$)**: δ 206.3, 161.4 (d, *J* = 245.1 Hz),144.1 (d, *J* = 3.0 Hz), 127.4 (d, *J* = 7.8 Hz), 115.4 (d, *J* = 21.2 Hz), 59.5, 33.7, 31.2 ppm; **^{19}F NMR (376 MHz, $CDCl_3$)**: δ -116.71 ppm; ***R*f**: 0.45 (Hexane/EtOAc, 95:5).

Spectral data match the ones reported in the literature.[4]

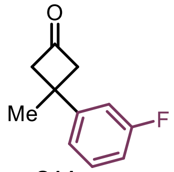

S14

3-(3-fluorophenyl)-3-methylcyclobutan-1-one (S14). *N*,*N*-dimethylacetamide (1.15 g, 1.23 mL,13.2 mmol, 1.2 equiv.), Tf_2O (6.22 g, 3.71 ml, 22.0 mmol, 2.0 equiv.), 1-fluoro-3-(prop-1-en-2-yl)benzene (1.5 g, 11.0 mmol, 1.0 equiv.), 2,4,6-trimethylpyridine (2.36 g, 2.55 ml, 22.0 mmol, 2.0 equiv.). The crude mixture was purified by column chromatography (EtOAc-hexane, 1-5%) to afford the corresponding cyclobutanone as a yellow oil in 89% yield (1.75 g, 9.82 mmol).

^{1}H NMR (400 MHz, $CDCl_3$): δ 7.37 – 7.26 (m, 1H), 7.10 – 7.04 (m, 1H), 7.02 – 6.96 (m, 1H), 6.96 – 6.89 (m, 1H), 3.47 – 3.38 (m, 2H), 3.14 – 3.06 (m, 2H), 1.59 (s, 3H) ppm; **^{13}C NMR (101 MHz, $CDCl_3$)**: δ 205.9, 163.0 (d, *J* = 246.3 Hz), 151.0 (d, *J* = 6.7 Hz), 130.3 (d, *J* = 8.2 Hz), 121.4 (d, *J* = 3.0 Hz), 113.3 (d, *J* = 21.1 Hz),113.0 (d, *J* = 21.7 Hz), 59.3, 34.0, 30.9 ppm; **^{19}F NMR (376 MHz, $CDCl_3$)**: δ -112.45 ppm; **IR (ATR)**: 2960, 2924, 1779, 1615, 1588, 1491, 1435, 1381, 1302, 1269, 1213, 1159, 1143, 1075, 954, 920, 868, 785, 735, 699, 536, 450 cm^{-1}; **HRMS (ESI)**: calcd. for $[C_{11}H_{11}FO+Na]^+$, $[M+Na]^+$: 206.0686; found: 206.0690; ***R*f**: 0.46 (Hexane/EtOAc, 95:5).

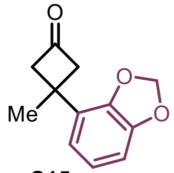

S15

3-(benzo[d][1,3]dioxol-4-yl)-3-methylcyclobutan-1-one (S15). *N*,*N*-dimethylacetamide (645 mg, 686 µl, 7.40 mmol, 1.2 equiv.), Tf_2O (2.61 g, 1.55 ml, 9.25 mmol, 1.5 equiv.), 5-(prop-1-en-2-yl)benzo[d][1,3]dioxole (1.0 g, 6.17 mmol, 1.0 equiv.), 2,4,6-trimethylpyridine (1.12 g, 1.22 ml, 9.25 mmol, 1.5 equiv.). The crude mixture was purified by column chromatography (EtOAc-hexane, 1-5%) to afford the corresponding cyclobutanone as a yellow solid in 43% yield (570 mg, 2.79 mmol).

^{1}H NMR (400 MHz, $CDCl_3$): δ 6.81 – 6.70 (m, 3H), 5.94 (s, 2H), 3.44 – 3.35 (m, 2H), 3.10 – 3.00 (m, 2H), 1.56 (s, 3H) ppm; **^{13}C NMR (101 MHz, $CDCl_3$)**: δ 206.6, 147.9, 146.0, 142.5, 118.6, 108.2, 106.7, 101.2, 59.4, 34.0, 31.3; **IR (ATR)**: 2956, 2920, 1777, 1609, 1503, 1486, 1434, 1378, 1344, 1297, 1230, 1184, 1108, 1076, 1035, 934, 859, 807, 728, 658, 636, 575, 529, 416 cm^{-1}; **HRMS (ESI)**: calcd. for $[C_{12}H1_2O_3+Na]^+$, $[M+Na]^+$: 227.0679; found: 227.0677; ***R*f**: 0.27 (Hexane/EtOAc, 95:5); **m.p.**: 62–64°C.

Procedure for *α*-deuteration of a 3-methyl-3-phenylcyclobutan-1-one[10]

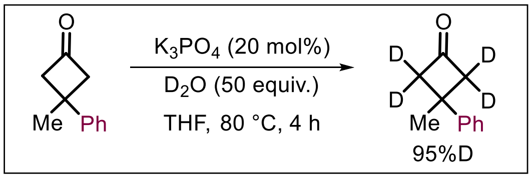

An oven-dried 35 mL two-neck pressure tube equipped with a magnetic stirring bar was charged with 3-methyl-3-phenylcyclobutan-1-one **S1** (280 mg, 1.75 mmol, 1.0 equiv). The tube was evacuated and backfilled with argon 3 times. Next, K_3PO_4 (74.2 mg, 0.35 mmol, 0.2 equiv), D_2O (1.58 mL, 87.4 mmol, 50 equiv), and dry THF (6.32 mL, V_{THF}/V_{D2O} = 4/1) were added. The resulting mixture was then heated at 80 °C for 4 hours. After this, the mixture cooled down to room

temperature, and was extracted directly with diethyl ether, evaporated under reduced pressure, and the residue was purified by column chromatography on silica gel (EtOAc-hexane 5-10%) to give 3-methyl-3-phenylcyclobutan-1-one-2,2,4,4-d_4 **S1-D** with 95% D-incorporation (94% yield, 270 mg, 1.64 mmol) as a yellow oil.

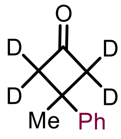

S1-D

^{1}H NMR (400 MHz, $CDCl_3$): δ 7.46 – 7.32 (m, 2H), 7.34 – 7.28 (m, 2H), 7.25 (t, J = 7.2 Hz, 1H), 3.44 (s, 0.10H), 3.09 (s, 0.10H), 1.60 (s, 3H) ppm; **^{13}C NMR (101 MHz, $CDCl_3$)**: δ 206.9, 148.4, 128.7, 126.4, 125.8, 58.9 (quint, J = 12.95 Hz), 33.6, 31.0 ppm; **IR (ATR)**: 2957, 2362, 1777, 1602, 1495, 1445, 1276, 1154, 1082, 1028, 767, 698, 532 cm^{-1}; **HRMS (ESI)**: calcd. for $[C_{11}H_8D_4O+Na]^+$, $[M+Na]^+$: 187.1031; found: 187.1036; ***R*f**: 0.47 (Hexane/EtOAc, 9:1).

General procedure A for the synthesis of cyclobutanols

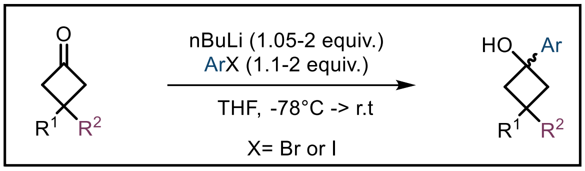

A flame-dried two-neck flask equipped with a stirring bar was charged with an aryl halide and anhydrous THF (0.075 M) under an Ar atmosphere. After cooling to -78 °C, n-BuLi (1.6 M in hexane, 1.1-2.2 equiv.) was added dropwise, and the mixture was stirred at this temperature for 1 h. Next, cyclobutanone was added at -78 °C. The reaction mixture was slowly warmed up to room temperature while stirring for 16 h. The mixture was quenched with water at 0 °C and extracted with EtOAc or Et_2O three times. The combined organic layers were washed with brine, dried over Na_2SO_4, and the solvent was evaporated. The mixture was purified by column chromatography with EtOAc-hexane.

General procedure B for the synthesis of cyclobutanols

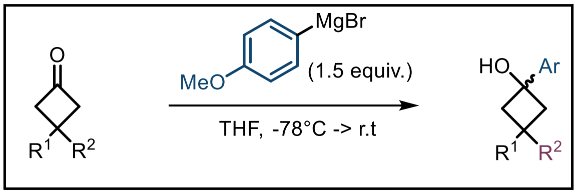

A flame-dried two-neck flask equipped with a stirring bar was charged with the cyclobutanone and anhydrous THF (0.2 M) under an Ar atmosphere. After cooling to -78 °C, 4-anisylmagnesium bromide (1.0 M in THF, 1.5 equiv.) was added dropwise. Next, the reaction mixture was slowly warmed up to room temperature while stirring for 16 h. The mixture was quenched with water at 0 °C and washed with EtOAc three times. The combined organic layers were washed with brine,

dried over Na_2SO_4, and the solvent was evaporated. The mixture was purified by column chromatography with EtOAc-hex.

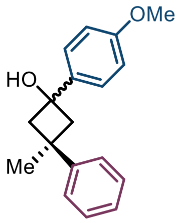

1a

***cis/trans*-1-(4-Methoxyphenyl)-3-methyl-3-phenylcyclobutan-1-ol (1a).** Following the General Procedure A. 1-bromo-4-methoxybenzene (3.07 g, 2.1 ml, 16.41 mmol, 2.2 equiv.), n-BuLi (1.6 M in hexane, 10.3 ml, 16.41 mmol, 2.2 equiv.), 3-methyl-3-phenylcyclobutan-1-one (1.2 g, 7.46 mmol, 1 equiv.). The crude mixture was purified by column chromatography (EtOAc-hexane, 5-10%) to afford a mixture of two inseparable diastereoisomers as a white solid in 82% yield, 2:1 dr (1.64 g, 6.13 mmol).

^{1}H NMR (400 MHz, $CDCl_3$): (mixture of diastereomers, signals are reported as seen) δ 7.50 – 7.44 (m, 1.7H), 7.38 – 7.12 (m, 7.6H), 6.99 – 6.88 (m, 1.7H), 6.84 – 6.76 (m, 1H), 3.84 (s, 3H), 3.77 (s, 1.5H), 2.99 – 2.82 (m, 4.3H), 2.63 – 2.56 (m, 1H), 1.91 (bs, 0.5H), 1.79 (bs, 0.8H), 1.71 (s, 1.5H), 1.25 (s, 3H) ppm: **^{13}C NMR (101 MHz, $CDCl_3$)**: (mixture of diastereomers, signals are reported as seen) δ 159.0, 158.6, 151.9, 151.8, 140.0, 138.8, 128.4, 128.3, 127.2, 126.1, 125.6, 125.4, 125.4, 125.3, 116.1, 114.9, 114.0, 113.7, 72.8, 72.2, 55.4, 55.4, 48.9, 48.6, 36.0, 34.2, 32.8, 31.3 ppm; ***R*f**: 0.16 (Hexane/EtOAc, 9:1).

Spectral data match the ones reported in the literature.[11]

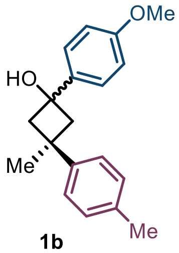

1b

***cis/trans*-1-(4-methoxyphenyl)-3-methyl-3-(*p*-tolyl)cyclobutan-1-ol (1b).** Following the General Procedure A. 1-bromo-4-methoxybenzene (2.92 g, 1.96 ml, 15.6 mmol, 1.1 equiv.), n-BuLi (1.6 M in hexane, 9.75 ml, 15.6 mmol, 1.1 equiv.), 3-methyl-3-phenylcyclobutan-1-one (2.47 g, 14.2 mmol, 1 equiv.). The crude mixture was purified by column chromatography (EtOAc-hexane, 5-10%) to afford a mixture of two inseparable diastereoisomers as a white solid in 87% yield, 1.3:1 dr (3.5 g, 12.0 mmol).

^{1}H NMR (400 MHz, $CDCl_3$): (mixture of diastereomers, signals are reported as seen) δ 7.47 (d, *J* = 8.8 Hz, 2H), 7.27 – 7.14 (m, 5.8H), 7.12 – 7.05 (m, 3.1H), 6.93 (d, *J* = 8.7 Hz, 2H), 6.81 (d, *J* = 8.7 Hz, 1.5H), 3.83 (s, 3H), 3.76 (s, 2.39H), 3.00 – 2.79 (m, 5.6H), 2.62 – 2.55 (m, 1.6H), 2.34 (s, 3H), 2.31 (s, 2.39H), 1.92 (s, 0.75H), 1.80 (s, 1H), 1.69 (s, 2.39H), 1.24 (s, 3H) ppm; **^{13}C NMR (101 MHz, $CDCl_3$)**: (mixture of diastereomers, signals are reported as seen) δ 159.0, 158.7, 148.9, 140.1, 138.9, 135.0, 134.9, 129.2, 129.0, 127.2, 126.1, 125.3, 125.2, 114.0, 113.8, 72.8, 72.3, 55.5, 55.4, 49.1, 48.7, 35.6, 34.0, 32.9, 31.4, 21.1, 21.1 ppm.; **IR (ATR):** 3287, 3052, 3027, 2962, 2916, 1618, 1600, 1573, 1506, 1492, 1413, 1376, 1299, 1265, 1243, 1176, 1156, 1111, 1096, 1067, 1028, 977, 843, 807, 764, 736, 681, 649, 533, 485, 457 cm^{-1}; **HRMS (ESI)**: calcd. for $[C_{19}H_{22}O_2+Na]^+$, $[M+Na]^+$: 305.1512; found: 305.1515; ***R*f**: 0.17 (Hexane/EtOAc, 9:1); **m.p.**: 77–78°C.

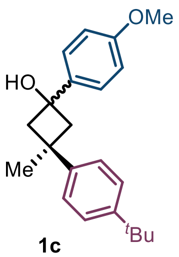

1c

***cis/trans*-3-(4-(*tert*-butyl)phenyl)-1-(4-methoxyphenyl)-3-methylcyclobutan-1-ol (1c).** Following the General Procedure A. 1-bromo-4-methoxybenzene (363 mg, 244 µl, 1.94 mmol, 1.1 equiv.), n-BuLi (1.6 M in hexane, 1.21 ml, 1.94 mmol, 1.1 equiv.), 3-methyl-3-phenylcyclobutan-1-one (350 mg, 1.62 mmol, 1 equiv.). The crude mixture was purified by column chromatography (EtOAc-hexane, 5-10%) to afford a mixture of two inseparable diastereoisomers as a white solid in 57% yield, 10:1 dr (300 mg, 0.925 mmol).

^{1}H NMR (400 MHz, CDCl$_3$): (mixture of diastereomers, signals are reported as seen) δ 7.47 (d, *J* = 8.7 Hz, 2H), 7.36 (d, *J* = 8.4 Hz, 2H), 7.30 (d, *J* = 8.4 Hz, 0.2H), 7.24 (d, *J* = 8.2 Hz, 2H), 7.10 (d, *J* = 8.2 Hz, 0.2H), 6.93 (d, *J* = 8.7 Hz, 2H), 6.81 (d, *J* = 8.7 Hz, 0.2H), 6.75 (d, *J* = 8.2 Hz, 0.2H), 3.83 (s, 3H), 3.76 (s, 0.3H) 2.98 2.80 (m, 4.3H), 2.61 2.53 (m, 0.2H), 1.92 (s, 0.07H) 1.83 (s, 0.9H), 1.70 (s, 0.3H), 1.32 (s, 9H), 1.29 (s, 1H), 1.24 (s, 3H) ppm; **^{13}C NMR (101 MHz, CDCl$_3$)**: (mixture of diastereomers, signals are reported as seen) δ 158.9, 148.7, 148.2, 138.9, 127.2, 125.3, 125.0, 113.9, 72.2, 55.4, 49.1, 34.4, 33.7, 31.5, 31.2 ppm; **IR (ATR):** 3366, 2995, 2866, 2835, 1609, 1583, 1511, 1463, 1397, 1362, 1298, 1251, 1177, 1125, 1095, 1033, 897, 831, 734, 644, 576, 429 cm^{-1}; **HRMS (ESI)**: calcd. for [$C_{22}H_{28}O_2$+Na]$^+$, [M+Na]$^+$: 347.1982; found: 347.1983; ***R*f**: 0.19 (Hexane/EtOAc, 9:1); **m.p.**: 107–109°C

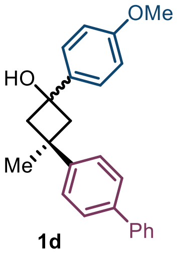

1d

***cis/trans*-3-([1,1'-biphenyl]-4-yl)-1-(4-methoxyphenyl)-3-methylcyclobutan-1-ol (1d)**. Following the General Procedure (B). 3-([1,1'-biphenyl]-4-yl)-3-methylcyclobutan-1-one (1.0 g, 4.2 mmol, 1 equiv.), 4-anisylmagnesium bromide (1.0 M in THF, 6.3 ml, 6.34 mmol, 1.5 equiv.). The crude mixture was purified by column chromatography (EtOAc-hexane, 20%) to afford a mixture of two inseparable diastereoisomers as a white solid in 61 % yield, 1.1:1 dr (895 mg, 2.60 mmol).

^{1}H NMR (400 MHz, CDCl$_3$): (mixture of diastereomers, signals are reported as seen) δ 7.62 – 7.54 (m, 6H), 7.53 – 7.46 (m, 4H), 7.45 – 7.36 (m, 6H), 7.35 – 7.29 (m, 2H), 7.28 – 7.22 (m, 4H), 6.94 (d, *J* = 8.6 Hz, 2H), 6.82 (d, *J* = 8.7 Hz, 1.8H), 3.83 (s, 3H), 3.76 (s, 2.9H), 3.04 – 2.86 (m, 6H), 2.69 – 2.59 (m, 1.8H), 1.84 (s, 0.69),1.74 (s, 2.9H), 1.55 (s, 0.94H), 1.29 (s, 3H) ppm; **^{13}C NMR (101 MHz, CDCl$_3$)**: (mixture of diastereomers, signals are reported as seen) δ 159.0, 158.7, 150.9, 141.2, 141.1, 139.9, 138.8, 138.5, 138.4, 128.8, 127.2, 127.2, 127.1, 127.1, 127.1, 126.1, 125.8, 125.7, 114.0, 113.8, 72.8, 72.2, 55.4, 55.4, 49.0, 48.6, 35.8, 34.1, 32.7, 31.3 ppm; **IR (ATR):** 3852, 3750, 3420, 3027, 2955, 2930, 2863, 2834, 1771, 1610, 1513, 1486, 1298, 1249, 1178, 1076, 1036, 834, 767, 698, 574 cm^{-1}; **HRMS (ESI)**: calcd. for [$C_{24}H_{24}O_2$+Na]$^+$, [M+Na]$^+$: 367.1669; found: 367.1669; ***R*f**: 0.15 (Hexane/EtOAc, 9:1); **m.p.**: 125–127°C.

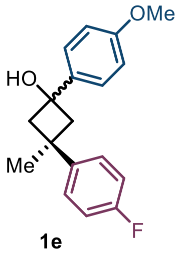

1e

***cis/trans*-3-(4-fluorophenyl)-1-(4-methoxyphenyl)-3-methylcyclobutan-1-ol (1e).** Following the General Procedure A. 1-bromo-4-methoxybenzene (504 mg, 338 µl, 2.69 mmol, 1.2 equiv.), n-BuLi (1.6 M in hexane, 1.68 ml, 2.69 mmol, 1.2 equiv.), 3-(4-fluorophenyl)-3-methylcyclobutan-1-one (400 mg, 2.24 mmol, 1 equiv.). The crude mixture was purified by column chromatography (EtOAc-hexane, 5-10%) to afford a mixture of two inseparable diastereoisomers as a white solid in 93% yield, 1.2:1 dr (600 mg, 2.10 mmol).

^{1}H NMR (400 MHz, $CDCl_3$): (mixture of diastereomers, signals are reported as seen) δ 7.46 (d, J = 8.8 Hz, 2H), 7.27 – 7.19 (m, 4H), 7.14 – 7.08 (m, 1.68H), 7.03 – 6.96 (m, 2.5H), 6.96 – 6.91 (m, 3H), 6.81 (d, J = 8.8 Hz, 1.68H), 3.82 (s, 3H), 3.76 (s, 2.5H), 2.96 – 2.89 (m, 3.7H), 2.85 – 2.77 (m, 2H), 2.62 – 2.56 (m, 1.64H), 1.96 (bs, 1.52H), 1.67 (s, 2.5H), 1.22 (s, 3H) ppm: **^{13}C NMR (101 MHz, $CDCl_3$)**: (mixture of diastereomers, signals are reported as seen) δ 160.9 (d, J = 243.3 Hz), 160.8 (d, J = 243.2 Hz), 159.0, 158.7, 147.6 (d, J = 3.2 Hz), 147.4 (d, J = 3.0 Hz), 139.7, 138.7, 127.1, 126.9 (d, J = 8.0 Hz), 126.6 (d, J = 7.8 Hz), 126.1, 115.1 (d, J = 10.1 Hz), 114.9 (d, J = 10.1 Hz), 114.0, 113.8, 72.5, 72.0, 55.4, 55.3, 49.9, 48.7, 35.5, 33.8, 32.8, 31.4 ppm; **^{19}F NMR (376 MHz, $CDCl_3$)**: (mixture of diastereomers) δ -118.13, -118.23; **IR (ATR):** 3393, 2931, 2836, 1609, 1583, 1508, 1463, 1372, 1298, 1218, 1177, 1159, 1105, 1032, 829, 804, 735, 652, 545, 441 cm^{-1}; **HRMS (ESI)**: calcd. for $[C_{18}H_{19}FO_2+Na]^+$, $[M+Na]^+$: 309.1261; found: 309.1263; ***R*f**: 0.15 (Hexane/EtOAc, 9:1); **m.p.**: 54–55°C.

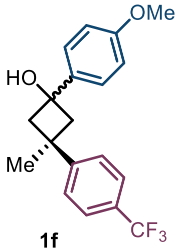

1f

***cis/trans*-1-(4-methoxyphenyl)-3-methyl-3-(4-(trifluoromethyl)phenyl)cyclobutan-1-ol (1f).** Following the General Procedure A. 1-bromo-4-methoxybenzene (154 mg, 103 µl, 822 µmol, 1.25 equiv.), n-BuLi (1.6 M in hexane, 493 µl, 789 µmol, 1.2 equiv.), 3-methyl-3-(4-(trifluoromethyl)phenyl)cyclobutan-1-one (150 mg, 657 µmol, 1 equiv.). The crude mixture was purified by column chromatography (EtOAc-hexane 5-10%) to afford a mixture of two inseparable diastereoisomers as a white solid in 57% yield, 1.1:1 dr (126 mg, 375 µmol).

^{1}H NMR (400 MHz, $CDCl_3$): (mixture of diastereomers, signals are reported as seen) δ 7.58 (d, J = 8.0 Hz, 1.82H), 7.53 (d, J = 8.0 Hz, 2H), 7.46 (d, J = 8.8 Hz, 1.88H), 7.39 (d, J = 8.1 Hz, 1.88H), 7.30 – 7.19 (m, 5H), 6.94 (d, J = 8.7 Hz, 1.82H), 6.81 (d, J = 8.8 Hz, 2H), 3.83 (s, 2.77H), 3.76 (s, 3H), 2.95 (d, J = 11.5 Hz, 4H), 2.85 (d, J = 13.0 Hz, 1.89H), 2.68 – 2.60 (m, 2H), 1.93 (s, 0.9H), 1.79 (s, 0.82H) 1.70 (s, 2.84H), 1.24 (s, 3H) ppm; **^{13}C NMR (101 MHz, $CDCl_3$)**: (mixture of diastereomers, signals are reported as seen) δ 159.1, 158.8, 155.9, 155.8, 139.5, 138.5, 127.1, 126.1, 125.7, 125.6, 125.2 (dq, J = 7.7, 3.7 Hz), 116.1, 114.9, 114.1, 113.8, 72.6, 72.1, 55.4, 55.4, 48.7, 48.4, 36.2, 34.4, 32.5, 31.01 ppm; **^{19}F NMR (376 MHz, $CDCl_3$)**: (mixture of diastereomers) δ -62.12, -62.14; **IR (ATR):** 3317, 2935, 2837, 1611, 1580, 1511, 1458, 1407, 1325, 1299, 1246, 1177, 1106, 1065, 1015, 954, 896, 829, 694, 609, 563, 528 cm^{-1}; **HRMS (ESI)**: calcd. for $[C_{19}H_{19}F_3O_2+Na]^+$, $[M+Na]^+$: 359.1229; found: 359.1225; ***R*f**: 0.17 (Hexane/EtOAc, 8:2); **m.p.**: 99–101°C.

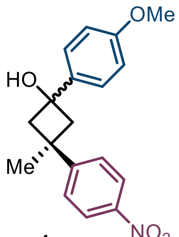

1g

***trans/cis*-3-Methyl-3-(4-nitrophenyl)-1-phenylcyclobutan-1-ol (1g).** Following the General Procedure B. 3-methyl-3-(4-nitrophenyl)cyclobutan-1-one (622 mg, 3.03 mmol, 1 equiv.), 4-anisylmagnesium bromide (1.0 M in THF, 4.5 ml, 4.55 mmol, 1.5 equiv.). The crude mixture was purified by column chromatography (EtOAc-hexane, 15%) to afford a mixture of two inseparable diastereoisomers as a beige solid in 64% yield, 1.8:1 dr (617 mg, 1.95 mmol).

^{1}H NMR (400 MHz, $CDCl_3$): (mixture of diastereomers, signals are reported as seen) δ 8.19 (d, J = 8.5 Hz, 1.1H), 8.13 (d, *J* = 8.6 Hz, 1.9H), 7.45 (dd, *J* = 11.1, 8.4 Hz, 2.5H), 7.31 (d, *J* = 8.5 Hz, 2H), 7.23 (d, *J* = 8.7 Hz, 1.9H), 6.95 (d, *J* = 8.6 Hz, 1.2H), 6.82 (d, *J* = 8.7 Hz, 2H), 3.84 (s, 1.7H), 3.76 (s, 3H), 3.02 – 2.94 (m, 3.1H), 2.90 – 2.82 (m, 1.3H), 2.72 – 2.65 (m, 1.9H), 2.04 (bs, 0.9H), 1.90 (bs, 0.6H), 1.72 (s, 3H), 1.27 (s, 1.7H) ppm; **^{13}C NMR (101 MHz, $CDCl_3$)**: (mixture of diastereomers, signals are reported as seen) δ 159.5, 159.4, 159.2, 158.9, 146.0, 145.9, 139.1, 138.3, 127.1, 126.4, 126.3, 126.1, 123.9, 123.8, 114.1, 113.9, 72.5, 72.0, 55.5, 55.4, 48.7, 48.3, 36.6, 34.8, 32.3, 30.9, 22.8, 14.3 ppm; **IR (ATR):** 3404, 2933, 2836, 1601, 1512, 1456, 1418, 1345, 1297, 1248, 1178, 1102, 1090, 1034, 953, 898, 855, 833, 757, 731, 702, 622, 564, 536 cm^{-1}; **HRMS (ESI)**: calcd. for $[C_{18}H_{19}NO_4+Na]^+$, $[M+Na]^+$: 336.1212; found: 336.1205; ***R*f**: 0.18 (Hexane/EtOAc, 8:2); **m.p.**: 96–98°C.

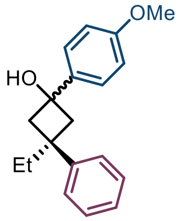

1h

***cis/trans*-3-Ethyl-1-(4-methoxyphenyl)-3-phenylcyclobutan-1-ol (1h).** Following the General Procedure B. 3-ethyl-3-phenylcyclobutan-1-one (228 mg, 1.31 mmol, 1 equiv.), 4-anisylmagnesium bromide (1.0 M in THF, 2.0 ml, 1.97 mmol, 1.5 equiv.). The crude mixture was purified by column chromatography (EtOAc-hexane, 20%) to afford a mixture of two inseparable diastereoisomers as a white solid in 76% yield, 1.3:1 dr (281 mg, 0.99 mmol).

^{1}H NMR (400 MHz, $CDCl_3$): (mixture of diastereomers, signals are reported as seen) δ 7.47 – 7.41 (m, 1.9H), 7.36 – 7.28 (m, 2.4H), 7.26 – 7.07 (m, 7.5H), 6.96 – 6.89 (m, 2H), 6.81 – 6.69 (m, 2.1H), 3.82 (s, 3H), 3.74 (s, 2.3H), 2.97 – 2.85 (m, 3.5H), 2.83 – 2.75 (m, 2H), 2.69 – 2.55 (m, 1.5H), 2.07 (q, *J* = 7.3 Hz, 1.5H), 2.01 (bs, 0.8H), 1.89 (bs, 1H), 1.55 (q, *J* = 7.3 Hz, 2H), 0.67 (t, *J* = 7.3 Hz, 2.3H), 0.53 (t, *J* = 7.3 Hz, 3H) ppm; **^{13}C NMR (101 MHz, $CDCl_3$)**: (mixture of diastereomers, signals are reported as seen) δ 158.9, 158.6, 149.2, 148.9, 140.1, 139.0, 128.1, 127.9, 127.0, 126.5, 126.4, 126.1, 125.5, 125.4, 116.1, 114.9, 113.9, 113.7, 73.0, 72.7, 55.4, 55.4, 47.4, 47.3, 39.7, 38.7, 37.0, 36.1, 9.0, 8.9 ppm; **IR (ATR):** 3312, 2962, 2932, 2873, 2833, 1610, 1581, 1510, 1494, 1444, 1417, 1376, 1301, 1242, 1176, 1110, 1029, 981, 949, 895, 826, 759, 701, 616, 571, 550 cm^{-1}; **HRMS (ESI)**: calcd. for $[C_{19}H_{22}O_2+Na]^+$, $[M+Na]^+$: 305.1517; found: 305.1510; ***R*f**: 0.22 (Hexane/EtOAc, 8:2); **m.p.**: 102–104°C.

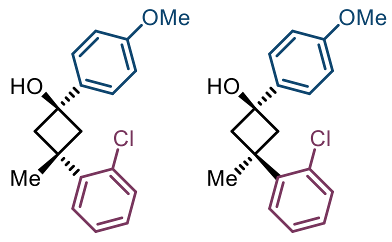

1i-*trans* 1i-*cis*

trans/cis*-3-(2-Chlorophenyl)-1-(4-methoxyphenyl)-3-methylcyclobutan-1-ol (1i-*trans* and 1i-*cis*).** Following the General Procedure A. 1-bromo-4-methoxybenzene (423 mg, 284 µl, 2.26 mmol, 1.1 equiv.), n-BuLi (1.6 M in hexane, 1.35 ml, 2.16 mmol, 1.05 equiv.), 3-(2-chlorophenyl)-3-methylcyclobutan-1-one (400 mg, 2.05 mmol, 1 equiv.). The crude mixture was purified by column chromatography (EtOAc-hexane, 1-6%) to afford two separable diastereoisomers: **1i-*trans as a yellow gel in 15% yield (91 mg, 0.30 mmol), **1i-*cis*** as a yellow solid in 23% yield (140 mg, 0.46 mmol).

1i-*trans*: **^{1}H NMR (400 MHz, $CDCl_3$)**: δ 7.31 – 7.24 (m, 3H), 7.22 – 7.06 (m, 3H), 6.88 – 6.79 (m, 2H), 3.77 (s, 3H), 3.08 – 2.95 (m, 2H), 2.76 – 2.67 (m, 2H), 1.91 (s, 1H), 1.79 (s, 3H) ppm; **^{13}C NMR (101 MHz, $CDCl_3$)**: δ 158.7, 147.8, 140.1, 132.7, 130.7, 127.6, 127.3, 126.8, 126.1, 113.8, 73.1, 55.5, 48.6, 37.7, 28.7 ppm; **IR (ATR)**: 3407, 3063, 2958, 2931, 2867, 2834, 1610, 1582, 1513, 1473, 1432, 1297, 1178, 1103, 1078, 1039, 1005, 952, 898, 829, 793, 756, 733, 720, 659, 615, 559, 515, 462, 403 cm^{-1}; **HRMS (ESI)**: calcd. for $[C_{18}H_{19}ClO_2+Na]^+$, $[M+Na]^+$: 325.0971; found: 325.0962; ***R*f**: 0.38 (Hexane/EtOAc, 8:2).

1i- *cis*: **^{1}H NMR (400 MHz, $CDCl_3$)**: δ 7.55 – 7.48 (m, 2H), 7.34 – 7.29 (m, 1H), 7.27 – 7.20 (m, 2H), 7.13 (ddd, J = 7.8, 5.6, 3.3 Hz, 1H), 6.99 – 6.90 (m, 2H), 3.83 (s, 3H), 3.16 – 3.06 (m, 2H), 2.98 – 2.86 (m, 2H), 1.86 (s, 1H), 1.22 (s, 3H) ppm; **^{13}C NMR (101 MHz, $CDCl_3$)**: δ 159.1, 148.1, 138.5, 132.5, 130.6, 127.6, 127.5, 127.3, 126.9, 114.1, 71.7, 55.5, 48.9, 34.6, 27.4 ppm; **IR (ATR)**: 3358, 3062, 2969, 2933, 2866, 2835, 1609, 1514, 1474, 1424, 1372, 1297, 1250, 1234, 1178, 1113, 1034, 943, 901, 832, 809, 754, 732, 660, 623, 559, 535, 461, 409 cm^{-1}; **HRMS (ESI)**: calcd. for $[C_{18}H_{19}ClO_2+Na]^+$, $[M+Na]^+$: 325.0971; found: 325.0970; ***R*f**: 0.29 (Hexane/EtOAc, 8:2); **m.p.**: 77–80°C.

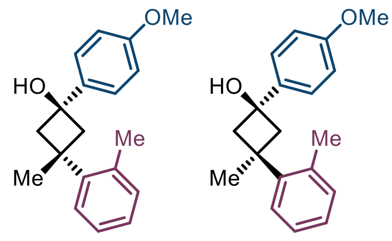

1j-*trans* 1j-*cis*

trans/cis*-1-(4-Methoxyphenyl)-3-methyl-3-(*o*-tolyl)cyclobutan-1-ol (1j-*trans and* 1j-*cis*).** Following the General Procedure B. 3-methyl-3-(*o*-tolyl)cyclobutan-1-one (419 mg, 2.41 mmol, 1 equiv.), 4-anisylmagnesium bromide (1.0 M in THF, 3.6 ml, 3.61 mmol, 1.5 equiv.). The crude mixture was purified by column chromatography (EtOAc-hexane, 15%) to afford two separable diastereoisomers: **2j-*trans as a white solid in 45% yield (304 mg, 1.08 mmol) and **2j-*cis*** as a white solid in 27% yield (180 mg, 0.64 mmol).

2j-*trans*: ^{1}H NMR (400 MHz, $CDCl_3$): δ 7.31 – 7.21 (m, 2H), 7.19 – 7.01 (m, 4H), 6.85 – 6.77 (m, 2H), 3.76 (s, 3H), 3.01 – 2.95 (m, 2H), 2.67 – 2.61 (m, 2H), 2.29 (s, 3H), 1.98 (s, 1H), 1.75 (s, 3H) ppm; **^{13}C NMR (101 MHz, $CDCl_3$)**: δ 158.7, 149.1, 140.2, 134.9, 131.5, 126.0, 125.9, 125.9, 113.8, 73.3, 55.4, 48.9, 37.5, 29.7, 20.2 ppm; **IR (ATR)**: 3410, 2955, 2926, 2867, 2834, 1609, 1582, 1512, 1488, 1455, 1416, 1295, 1177, 1087, 1111, 1073, 1036, 1004, 950, 897, 828, 795,

760, 730, 672, 637, 616, 563, 510, 461 cm^{-1}; **HRMS (ESI)**: calcd. for $[C_{19}H_{22}O_2+Na]^+$, $[M+Na]^+$: 305.1517; found: 305.1516; ***R*f**: 0.23 (Hexane/EtOAc, 8:2); **m.p.**: 72–74°C.

2j-*cis*: ^{1}H NMR (400 MHz, CDCl3): δ 7.55 – 7.46 (m, 2H), 7.21 – 7.07 (m, 4H), 6.97 – 6.90 (m, 2H), 3.83 (s, 3H), 3.10 – 3.00 (m, 2H), 2.92 – 2.85 (m, 2H), 2.29 (s, 3H), 1.90 (s, 1H), 1.15 (s, 3H) ppm; **^{13}C NMR (101 MHz, CDCl3)** δ 159.0, 149.4, 138.5, 134.7, 131.4, 127.6, 126.0, 126.0, 125.9, 114.0, 71.8, 55.4, 49.2, 34.2, 28.2, 20.3 ppm**. IR (ATR):** 3551, 2964, 2931, 2866, 2835, 1610, 1583, 1514, 1488, 1456, 1421, 1371, 1297, 1250, 1235, 1177, 1110, 1094, 1031, 939, 903, 832, 809, 759, 729, 672, 638, 626, 561, 459 cm^{-1}; **HRMS (ESI)**: calcd. for $[C_{19}H_{22}O_2+Na]^+$, $[M+Na]^+$: 305.1517; found: 305.1508; ***R*f**: 0.20 (Hexane/EtOAc, 8:2); **m.p.**: 81–83°C.

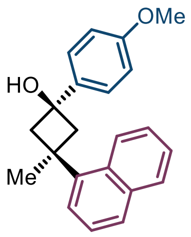

1k

***cis*-1-(4-methoxyphenyl)-3-methyl-3-(naphthalen-1-yl)cyclobutan-1-ol (1k).** Following the General Procedure A. 1-bromo-4-methoxybenzene (1.87 g, 1.25 mL, 10.0 mmol, 3.29 equiv.), n-BuLi (1.6 M in hexane, 6.26 ml, 10.0 mmol, 3.29 equiv.), 3-methyl-3-(naphthalen-1-yl)cyclobutan-1-one (640 mg, 3.04 mmol, 1 equiv.). The crude mixture was purified by column chromatography (EtOAc-hexane, 5-10%) to afford a mixture of two inseparable diastereoisomers as a white solid in 94% yield (915 mg, 2.87 mmol).

^{1}H NMR (400 MHz, CDCl3): δ 7.92 – 7.83 (m, 2H), 7.70 (d, J = 8.1 Hz, 1H), 7.57 (d, J = 8.7 Hz, 2H), 7.49 – 7.41 (m, 3H), 7.35 (d, J = 6.0 Hz, 1H), 6.97 (d, J = 8.8 Hz, 2H), 3.85 (s, 3H), 3.30 (d, J = 13.1 Hz, 2H), 3.12 (d, J = 12.9 Hz, 2H), 1.83 (s, 0.84H), 1.42 (s, 3H) ppm; **^{13}C NMR (101 MHz, CDCl3)**: δ 159.1, 147.7, 138.6, 134.9, 130.4, 129.4, 127.5, 126.8, 125.5, 125.4, 125.3, 123.3, 114.1, 72.1, 55.4, 50.2, 34.5, 29.7 ppm; **IR (ATR):** 3326, 3046, 2959, 2930, 2835, 1609, 1583, 1513, 1457, 1395, 1296, 1262, 1240, 1177, 1111, 1031, 1003, 902, 833, 801, 778, 733, 665, 553, 438 cm^{-1}; **HRMS (ESI)**: calcd. for $[C_{22}H_{22}O_2+Na]^+$, $[M+Na]^+$: 341.1512; found: 341.1510; ***R*f**: 0.17 (Hexane/EtOAc, 8:2); **m.p.**: 70–73°C.

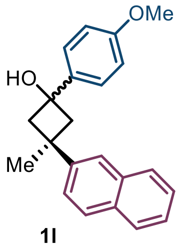

1l

***cis/trans*-1-(4-Methoxyphenyl)-3-methyl-3-(naphthalen-2-yl)cyclobutan-1-ol (1l).** Following the General Procedure A. 1-bromo-4-methoxybenzene (1.87 g, 1.3 ml, 10.08 mmol, 2.2 equiv.), n-BuLi (1.6 M in hexane, 6.3 ml, 10.08 mmol, 2.2 equiv.), 3-methyl-3-(naphthalen-2-yl)cyclobutan-1-one (964 mg, 4.58 mmol, 1 equiv.). The crude mixture was purified by column chromatography (EtOAc-hexane, 10%) to afford a mixture of two inseparable diastereoisomers as a white solid in 70% yield, 1.5:1 dr (1.02 g, 3.20 mmol).

^{1}H NMR (400 MHz, CDCl3): (mixture of diastereomers, signals are reported as seen) δ 7.88 – 7.73 (m, 4.9H), 7.70 (s, 1H), 7.57 (s, 0.7H), 7.52 – 7.37 (m, 6.2H), 7.33 (dd, J = 8.5, 1.9 Hz, 0.7H), 7.29 – 7.19 (m, 1.5H), 6.99 – 6.90 (m, 2H), 6.82 – 6.74 (m, 1.3H), 3.82 (s, 3H), 3.72 (s, 2H), 3.15 – 2.87 (m, 5.3H), 2.73 – 2.60 (m, 1.3H), 2.00 (bs, 1.6H), 1.77 (s, 2H), 1.32 (s, 3H) ppm; **^{13}C NMR (101 MHz, CDCl3)**: (mixture of diastereomers, signals are reported as seen) δ 159.0, 158.6, 149.1, 140.0, 138.9, 133.5, 133.4, 131.8, 131.7, 128.3, 128.2, 127.9, 127.7, 127.7, 127.7, 127.2, 127.2,

126.1, 126.1, 126.1, 125.4, 125.3, 124.7, 124.6, 123.1, 123.0, 114.0, 113.8, 72.8, 72.3, 55.4, 55.4, 48.9, 48.7, 36.1, 34.5, 32.7, 31.2 ppm; **IR (ATR)**: 3396, 3052, 2956, 2931, 2863, 2835, 1631, 1609, 1582, 1512, 1456, 1419, 1349, 1297, 1248, 1177, 1132, 1110, 1092, 1032, 950, 900, 856, 831, 820, 746, 703, 664, 622, 590, 477, 419, 408 cm^{-1}; **HRMS (ESI)**: calcd. for $[C_{22}H_{22}O_2+Na]^+$, $[M+Na]^+$: 341.1517; found: 341.1512; ***R*f**: 0.16 (Hexane/EtOAc, 8:2); **m.p.**: 121–123°C.

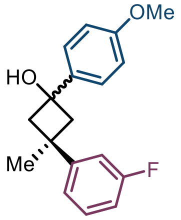

1m

***cis/trans*-3-(3-fluorophenyl)-1-(4-methoxyphenyl)-3-methylcyclobutan-1-ol (1m).** Following the General Procedure A. 1-bromo-4-methoxybenzene (378 mg, 254 µl, 2.02 mmol, 1.2 equiv.), n-BuLi (1.6 M in hexane, 1.26 ml, 2.02 mmol, 1.2 equiv.), 3-(3-fluorophenyl)-3-methylcyclobutan-1-one (300 mg, 1.68 mmol, 1 equiv.). The crude mixture was purified by column chromatography (EtOAc-hexane, 5-10%) to afford a mixture of two inseparable diastereoisomers as a white solid in 83% yield, 1.3:1 dr (400 mg, 1.40 mmol).

^{1}H NMR (400 MHz, $CDCl_3$): (mixture of diastereomers, signals are reported as seen) δ 7.46 (d, *J* = 8.7 Hz, 2H), 7.35 – 7.26 (m, 1H), 7.25 – 7.20 (m, 2H), 7.06 (d, *J* = 7.7 Hz, 1H), 7.02 – 6.79 (m, 8H), 3.82 (s, 3H), 3.76 (s, 2.3H), 2.97 – 2.88 (m, 3.6H), 2.85 – 2.78 (m, 2H), 2.62 – 2.55 (m, 1.5H), 2.07 (bs, 1.4H), 1.69 (s, 2.3H), 1.23 (s, 3H) ppm; **^{13}C NMR (101 MHz, $CDCl_3$)**: (mixture of diastereomers, signals are reported as seen) δ 163.0 (d, *J* = 245), 163.0 (d, *J* = 246), 159.0, 158.7, 154.7 (d, *J* = 6.5 Hz), 154.6 (d, *J* = 6.7 Hz), 139.7, 138.7, 129.9 (d, *J* = 8.3 Hz), 129.8 (d, *J* = 8.5 Hz), 127.2, 126.1, 121.0 (d, *J* = 2.7 Hz), 120.9 (d, *J* = 2.7 Hz), 114.0, 113.8, 112.6 (d, *J* = 10.8 Hz), 112.3 (d, *J* = 10.3 Hz), 112.2, 72.5, 71.9, 55.4, 55.4, 48.8, 48.5, 36.1, 36.0, 34.2, 34.2, 32.5, 31.1 ppm; **^{19}F NMR (376 MHz, $CDCl_3$)**: (mixture of diastereomers) δ -113.23, -113.24; **IR (ATR):** 3287, 3056, 2968, 2931, 2864, 1608, 1583, 1510, 1494, 1444, 1372, 1297, 1264, 1217, 1178, 1108, 1025, 925, 925, 895, 831, 763, 736, 643, 549 cm^{-1}; **HRMS (ESI)**: calcd. for $[C_{18}H_{19}FO_2+Na]^+$, $[M+H]^+$: 309.1261; found: 309.1264; ***R*f**: 0.15 (Hexane/EtOAc, 9:1); **m.p.**: 54–55°C.

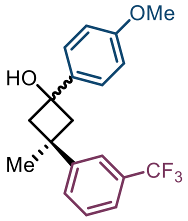

1n

***trans*/*cis*-1-(4-methoxyphenyl)-3-methyl-3-(3-(trifluoromethyl)phenyl)cyclobutan-1-ol (1n)**. Following the General Procedure B. 3-methyl-3-(3-(trifluoromethyl)phenyl)cyclobutan-1-one (250 mg, 1.1 mmol, 1 equiv.), 4-anisylmagnesium bromide (1.0 M in THF, 1.64 ml, 1.64 mmol, 1.5 equiv.). The crude mixture was purified by column chromatography (EtOAc-hexane, 5-15%) to afford a mixture of two inseparable diastereoisomers as a yellow gel in 54% yield, 1.2:1 dr (200 mg, 595 µmol).

^{1}H NMR (400 MHz, $CDCl_3$): (mixture of diastereomers, signals are reported as seen) δ 7.56 – 7.33 (m, 9H), 7.23 (d, *J* = 8.7 Hz, 2H), 6.95 (d, *J* = 8.7 Hz, 1.5H), 6.82 (d, *J* = 8.8 Hz, 2H), 3.83 (s, 2.5H), 3.76 (s, 3H), 3.02 – 2.92 (m, 3.6H), 2.90 – 2.82 (m, 1.6H), 2.69 – 2.60 (m, 2H), 2.34 (s, 0.96H), 2.20 (s, 0.68H), 1.71 (s, 3H), 1.26 (s, 2.5H) ppm; **^{13}C NMR (101 MHz, $CDCl_3$)**: (mixture of diastereomers, signals are reported as seen) δ 159.1, 158.8, 152.9, 152.8, 139.5, 138.6, 130.5 (td, *J* = 31.8, 7.7 Hz), 128.9, 128.8, 127.2, 126.1, 125.8 (d, *J* = 7.1 Hz), 123.1 (d, *J* = 6.9 Hz),122.4

(dt, J = 7.6, 3.8 Hz), 122.0 (dt, J = 11.5, 3.9 Hz), 116.2, 114.9, 114.1, 113.8, 72.6, 72.0, 55.4, 55.3, 48.6, 48.2, 36.2, 34.2, 32.6, 31.1 ppm; **^{19}F NMR (376 MHz, CDCl$_3$)**: (mixture of diastereomers) δ -62.21, -62.26 ppm; **IR (ATR):** 3392, 2933, 2837, 1610, 1513, 1333, 1296, 1274, 1247, 1161, 1118, 1070, 1034, 905, 831, 802, 737, 730, 654, 614, 557, 462 cm^{-1} **HRMS (ESI)**: calcd. for [$C_{19}H_{19}F_3O_2$+Na]$^+$, [M+Na]$^+$: 359.1229; found: 359.1232; ***R*f**: 0.17 (Hexane/EtOAc, 9:1);

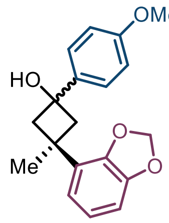

1o

***cis/trans*-3-(benzo[d][1,3]dioxol-4-yl)-1-(4-methoxyphenyl)-3-methylcyclobutan-1-ol (1o).** Following the General Procedure A. 1-bromo-4-methoxybenzene (495 mg, 332 µl, 2.64 mmol, 3.0 equiv.), n-BuLi (1.6 M in hexane, 1.65 ml, 2.64 mmol, 3.0 equiv.), 3-(benzo[d][1,3]dioxol-4-yl)-3-methylcyclobutan-1-one (180 mg, 881 µmol, 1 equiv.). The crude mixture was purified by column chromatography (EtOAc-hexane, 5-10%) to afford a mixture of two inseparable diastereoisomers as a white solid in 82% yield, 2:1 dr (225 mg, 720 µmol).

^{1}H NMR (400 MHz, CDCl$_3$): (mixture of diastereomers, signals are reported as seen) δ 7.45 (d, J = 8.7 Hz, 2H), 7.23 (d, J = 8.6 Hz, 1H), 6.92 (d, J = 8.7 Hz, 2H), 6.84 – 6.64 (m, 5H), 6.60 (d, J = 8.1 Hz, 0.5H), 5.92 (s, 2H), 5.89 (s, 1H), 3.82 (s, 3H), 3.76 (s, 1.5H), 2.94 – 2.83 (m, 3H), 2.83 – 2.75 (m, 2H), 2.59 – 2.51 (m, 1H), 2.15 (s, 0.45), 2.03 (s, 0.97), 1.66 (s, 1.5H), 1.21 (s, 3H) ppm; **^{13}C NMR (101 MHz, CDCl$_3$)**: (mixture of diastereomers, signals are reported as seen) δ 158.9, 158.6, 147.7, 147.5, 146.2, 146.1, 145.3, 145.2, 140.0, 138.8, 127.2, 126.1, 118.1, 117.9, 113.9, 113.7, 108.1, 108.0, 106.4, 106.3, 100.9, 100.9, 72.4, 71.9, 55.4, 55.4, 49.1, 48.8, 35.9, 34.2, 33.0, 31.6 ppm; **IR (ATR):** 3420, 2933, 2837, 1609, 1582, 1511, 1486, 1433, 1342, 1297, 1231, 1177, 1154, 1101, 1071, 1036, 937, 862, 833, 809, 733, 703, 642, 555, 447 cm^{-1}; **HRMS (ESI)**: calcd. for [$C_{19}H_{20}O_4$+Na]$^+$, [M+Na]$^+$: 335.1254; found: 335.1254; ***R*f**: 0.16 (Hexane/EtOAc, 8:2); **m.p.**: 71–73°C.

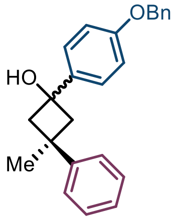

1p

***cis/trans*-1-(4-(benzyloxy)phenyl)-3-methyl-3-phenylcyclobutan-1-ol (1p).** Following the General Procedure A. 1-(benzyloxy)-4-bromobenzene (493 mg, 1.87 mmol, 1.2 equiv.), n-BuLi (1.6 M in hexane, 1.17 ml, 1.87 mmol, 1.2 equiv.), 3-methyl-3-phenylcyclobutan-1-one (250 mg, 1.56 mmol, 1 equiv.). The crude mixture was purified by column chromatography (EtOAc-hexane, 5-10%) to afford a mixture of two inseparable diastereoisomers as a white solid in 60% yield, 2:1 dr (320 mg, 929 µmol).

^{1}H NMR (400 MHz, CDCl$_3$): (mixture of diastereomers, signals are reported as seen) δ 7.48 (t, J = 8.4 Hz, 4H), 7.44 – 7.14 (m, 14.5H), 7.02 (d, J = 8.7 Hz, 2H), 6.89 (d, J = 8.7 Hz, 1H), 5.10 (s, 2H), 5.03 (s, 1H), 3.02 – 2.83 (m, 5H), 2.64 – 2.58 (m, 1H), 2.01 (s, 0.43H) 1.89 (s, 0.86H), 1.72 (s, 1.5H), 1.26 (s, 3H) ppm; **^{13}C NMR (101 MHz, CDCl$_3$)**: (mixture of diastereomers, signals are reported as seen) δ 158.2, 157.9, 151.9, 151.8, 140.2, 139.1, 137.1, 128.7, 128.7, 128.7, 128.4, 128.3, 128.1, 128.0, 127.6, 127.5, 127.2, 126.2, 125.6, 125.4, 125.3, 125.2, 114.9, 114.7, 72.6, 72.2, 70.2, 70.1, 48.9, 48.6, 36.0, 34.2, 32.8, 31.4 ppm; **IR (ATR):** 3375, 3025, 2929, 2863, 1608,

1581, 1510, 1494, 1454, 1379, 1296, 1232, 1176, 1108, 1025, 896, 830, 734, 697, 549, 457 cm^{-1}; **HRMS (ESI)**: calcd. for [$C_{24}H_{24}O_2$+Na]+, [M+H]+: 367.1669; found: 367.1668; ***R*f**: 0.17 (Hexane/EtOAc, 9:1); **m.p.**: 70–72°C.

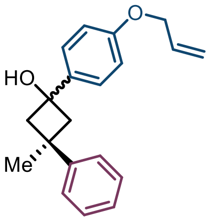

1q

***cis/trans*-1-(4-(allyloxy)phenyl)-3-methyl-3-phenylcyclobutan-1-ol (1q).** Following the General Procedure A. 1-(allyloxy)-4-bromobenzene (399 mg, 1.87 mmol, 1.2 equiv.), n-BuLi (1.6 M in hexane, 1.17 ml, 1.87 mmol, 1.2 equiv.), 3-methyl-3-phenylcyclobutan-1-one (250 mg, 1.56 mmol, 1 equiv.). The crude mixture was purified by column chromatography (EtOAc-hexane, 5-20%) to afford a mixture of two inseparable diastereoisomers as a yellow gel in 65% yield, 2:1 dr (300 mg, 1.02 mmol).

^{1}H NMR (400 MHz, $CDCl_3$): (mixture of diastereomers, signals are reported as seen) δ 7.48 – 7.39 (m, 2H), 7.35 – 7.24 (m, 4.8H), 7.21 – 7.10 (m, 3.7), 6.92 (d, J = 8.7 Hz, 2H), 6.79 (d, J = 8.8 Hz, 1H), 6.13 – 5.93 (m, 1.5H), 5.45 – 5.21 (m, 3.0H), 4.53 (d, J = 5.3 Hz, 2H), 4.46 (d, J = 5.3 Hz, 1H), 2.95 – 2.79 (m, 5H), 2.59 – 2.52 (m, 1H), 2.15 (bs, 1.5H), 1.68 (s, 1.5H) 1.22 (s, 3H) ppm; **^{13}C NMR (101 MHz, $CDCl_3$)**: (mixture of diastereomers, signals are reported as seen) δ 157.9, 157.6, 151.9, 151.8, 140.1, 139.0, 133.3, 133.3, 128.4, 128.3, 127.2, 126.1, 125.5, 125.4, 125.3, 125.2, 117.8, 117.7, 114.7, 114.5, 72.6, 72.0, 68.9, 68.9, 48.9, 48.5, 36.0, 34.1, 32.8, 31.3 ppm; **IR (ATR)**: 3392, 3023, 2930, 2864, 1717, 1648, 1608, 1581, 1494, 1445, 1363, 1296, 1233, 1177, 1108, 1025, 966, 830, 763, 736, 699, 549 cm^{-1}; **HRMS (ESI)**: calcd. for [$C_{20}H_{22}O_2$+Na]$^+$, [M+Na]$^+$: 317.1512; found: 317.1513; ***R*f**: 0.18 (Hexane/EtOAc, 9:1);

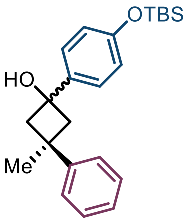

1s

***trans/cis*-1-(4-((*tert*-butyldimethylsilyl)oxy)phenyl)-3-methyl-3-phenylcyclobutan-1-ol (1s).** Following the General Procedure A. (4-bromophenoxy)(*tert*-butyl)dimethylsilane (2.15 g, 7.49 mmol, 1.2 equiv.), n-BuLi (1.6 M in hexane, 4.7 ml, 7.49 mmol, 1.2 equiv.), 3-methyl-3-phenylcyclobutan-1-one (1.0 g, 6.24 mmol, 1 equiv.). The crude mixture was purified by column chromatography (EtOAc-hexane, 5-10%) to afford a mixture of two inseparable diastereoisomers as a yellow oil in 70% yield, 1.5:1 dr (1.6 g, 4.30 mmol).

^{1}H NMR (400 MHz, $CDCl_3$): (mixture of diastereomers, signals are reported as seen) δ 7.43 – 7.25 (m, 6.9H), 7.22 – 7.09 (m, 5H), 6.90 – 6.82 (m, 1.9H), 6.78 – 6.68 (m, 1.2H), 3.04 – 2.78 (m, 5.4H), 2.65 – 2.53 (m, 1.3H), 1.98 (bs, 0.7H), 1.87 (bs, 1H), 1.69 (s, 2H), 1.23 (s, 3H), 0.99 (s, 9H), 0.95 (s, 6H), 0.21 (s, 6H), 0.15 (s, 4H) ppm; **^{13}C NMR (101 MHz, $CDCl_3$)**: (mixture of diastereomers, signals are reported as seen) δ 155.1, 154.7, 152.0, 151.9, 140.5, 139.4, 128.4, 128.3, 127.1, 126.1, 125.6, 125.4, 125.4, 125.3, 120.1, 119.9, 72.8, 72.2, 49.0, 48.5, 36.0, 34.2, 32.8, 31.3, 25.8, 25.8, 18.3, 18.3, -4.2, -4.3 ppm**; IR (ATR)**: 3361, 3024, 2955, 2929, 2858, 2361, 1606, 1540, 1510, 1495, 1472, 1445, 1362, 1253, 1171, 1107, 1028, 1009, 912, 836, 807, 779, 762, 739, 699, 683, 668, 553, 503 cm^{-1}; **HRMS (ESI)**: calcd. for [$C_{23}H_{32}O_2Si$+Na]$^+$, [M+Na]$^+$: 391.2069; found: 391.2066; ***R*f**: 0.27 (Hexane/EtOAc, 9:1); **m.p.**: 48–50°C.

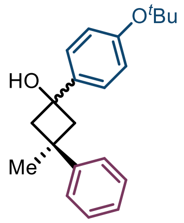

1t

***cis/trans*-1-(4-(*tert*-butoxy)phenyl)-3-methyl-3-phenylcyclobutan-1-ol (1t)**. Following the General Procedure A. 1-bromo-4-(*tert*-butoxy)benzene (515 mg, 2.25 mmol, 1.2 equiv.), n-BuLi (1.6 M in hexane, 1.4 ml, 2.25 mmol, 1.2 equiv.), 3-methyl-3-phenylcyclobutan-1-one (300 mg, 1.87 mmol, 1 equiv.). The crude mixture was purified by column chromatography (EtOAc-hexane, 10%) to afford a mixture of two inseparable diastereoisomers as a white solid in 79% yield, 5.4:1 dr (458 mg, 1.48 mmol).

^{1}H NMR (400 MHz, $CDCl_3$): (mixture of diastereomers, signals are reported as seen) δ 7.46 – 7.41 (m, 2H), 7.38 – 7.26 (m, 4.3H), 7.22 – 7.12 (m, 2H), 7.06 – 6.97 (m, 2H), 6.90 – 6.85 (m, 0.4H), 3.00 – 2.83 (m, 4.4H), 2.62 – 2.57 (m, 0.4H), 1.89 (s, 0.6H), 1.70 (s, 0.4H), 1.36 (s, 9H), 1.30 (s, 2.1H), 1.26 (s, 3H) ppm; **^{13}C NMR (101 MHz, $CDCl_3$)**: (mixture of diastereomers, signals are reported as seen) δ 154.8, 154.4, 151.9, 142.5, 141.3, 128.5, 128.3, 126.5, 125.6, 125.5, 125.4, 125.4, 125.3, 124.1, 124.1, 78.7, 78.6, 72.9, 72.3, 49.1, 48.5, 36.1, 34.4, 32.8, 31.5, 29.0, 28.9 ppm; **IR (ATR)**: 3420, 3057, 3024, 2975, 2932, 2866, 1604, 1505, 1445, 1421, 1389, 1365, 1299, 1235, 1161, 1108, 1028, 924, 897, 856, 763, 701, 568, 547, 476, 419 cm^{-1}; **HRMS (ESI)**: calcd. for $[C_{21}H_{26}O_2+Na]^+$, $[M+Na]^+$: 333.1830; found: 333.1822; ***R*f**: 0.16 (Hexane/EtOAc, 9:1); **m.p.**: 98–100°C.

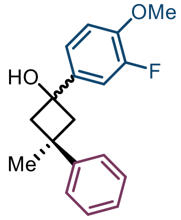

1u

***cis/trans*-1-(3-fluoro-4-methoxyphenyl)-3-methyl-3-phenylcyclobutan-1-ol (1u).** Following the General Procedure A. 4-bromo-2-fluoro-1-methoxybenzene (768 mg, 483 µl, 3.74 mmol, 1.2 equiv.), n-BuLi (1.6 M in hexane, 2.34 ml, 3.74 mmol, 1.2 equiv.), 3-methyl-3-phenylcyclobutan-1-one (500 mg, 3.12 mmol, 1 equiv.). The crude mixture was purified by column chromatography (EtOAc-hexane, 5-20%) to afford a mixture of two inseparable diastereoisomers as a yellow solid in 75 % yield, 3.6:1 dr (670 mg, 2.34 mmol).

^{1}H NMR (400 MHz, $CDCl_3$): (mixture of diastereomers, signals are reported as seen) δ 7.38 – 7.31 (m, 2.4H), 7.31 – 7.27 (m, 3H), 7.27 – 7.23 (m, 1.7H), 7.23 – 7.13 (m, 2H), 6.98 (t, *J* = 8.7 Hz, 1H), 6.84 (t, *J* = 8.7 Hz, 0.27H), 3.90 (s, 3H), 3.83 (s, 0.85H), 2.95 – 2.81 (m, 4.82H), 2.60 – 2.55 (m, 0.56H), 2.08 (s, 0.21H) 1.94 (s, 0.9H), 1.69 (s, 0.83H), 1.26 (s, 3H) ppm; **^{13}C NMR (101 MHz, $CDCl_3$)**: (mixture of diastereomers, signals are reported as seen) δ 152.4 (d, *J* = 245.6 Hz), 152.2 (d, *J* = 245.6 Hz), 151.6, 151.5, 147.0 (d, *J* = 11.0 Hz), 146.5 (d, *J* = 10.7 Hz), 140.9 (d, *J* = 5.3 Hz), 139.8 (d, *J* = 5.2 Hz), 128.5, 128.4, 125.7, 125.5, 125.3, 125.2,121.6 (d, *J* = 3.4 Hz), 120.5 (d, *J* = 3.5 Hz), 114.1 (d, *J* = 18.7 Hz), 113.3 (d, *J* = 2.3 Hz), 113.2 (d, *J* = 3.4 Hz), 113.1 (d, *J* = 13.2 Hz), 72.4, 72.0, 56.4, 56.4, 49.0, 48.7, 35.9, 34.3, 32.9, 31.5; **^{19}F NMR (376 MHz, $CDCl_3$)**: δ -134.34, -134.68 ppm; **IR (ATR):** 3382, 2934, 1622, 1583, 1515, 1495, 1443, 1306, 1269, 1217, 1177, 1137, 1026, 874, 812, 761, 734, 700, 648, 545, 458 cm^{-1}; **HRMS (ESI)**: calcd. for

$[C_{18}H_{19}FO_2+Na]^+$, $[M+Na]^+$: 309.1261; found: 309.1261; ***R*f**: 0.16 (Hexane/EtOAc, 8:2); **m.p.**: 48–49°C.

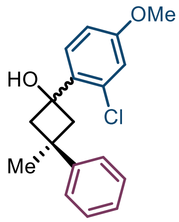

1v

***cis/trans*-1-(2-chloro-4-methoxyphenyl)-3-methyl-3-phenylcyclobutan-1-ol (1v).** Following the General Procedure A. 1-bromo-2-chloro-4-methoxybenzene (498 mg, 319 µl, 2.25 mmol, 1.2 equiv.), n-BuLi (1.6 M in hexane, 1.4 ml, 2.25 mmol, 1.2 equiv.), 3-methyl-3-phenylcyclobutan-1-one (300 mg, 1.87 mmol, 1 equiv.). The crude mixture was purified by column chromatography (EtOAc-hexane, 5-10%) to afford a mixture of two inseparable diastereoisomers as a pale yellow oil in 62% yield, 2:1 dr (350 mg, 1.16 mmol).

^{1}H NMR (400 MHz, $CDCl_3$): (mixture of diastereomers, signals are reported as seen) δ 7.46 (d, *J* = 8.7 Hz, 1H), 7.39 – 7.31 (m, 4H), 7.31 – 7.25 (m, 1H), 7.23 – 7.11 (m, 3H), 6.98 (d, *J* = 2.6 Hz, 1H), 6.88 (d, *J* = 2.6 Hz, 0.5H), 6.83 (dd, *J* = 8.7, 2.7 Hz, 1H), 6.71 (dd, *J* = 8.6, 2.6 Hz, 0.5H), 3.81 (d, *J* = 1.4 Hz, 3H), 3.75 (d, *J* = 1.5 Hz, 1.5H), 3.07 – 2.95 (m, 5H), 2.79 – 2.72 (m, 1H), 2.62 (bs, 1.16H), 1.73 (s, 1.5H), 1.28 (s, 3H) ppm; **^{13}C NMR (101 MHz, $CDCl_3$)**: (mixture of diastereomers, signals are reported as seen) δ 159.6, 159.4, 152.3, 151.4, 135.6, 134.8, 133.9, 133.3, 128.4, 128.31, 128.2, 127.9, 125.6, 125.4, 125.4, 125.1, 116.6, 116.0, 112.4, 112.3, 73.3, 72.4, 55.7, 55.6, 48.5, 47.5, 36.9, 35.0, 32.3, 32.1; **IR (ATR):** 3439, 3057, 2836, 1603, 1568, 1493, 1442, 1329, 1293, 1228, 1166, 1109, 1039, 901, 860, 842, 737, 700, 602, 545, 493 cm^{-1}; **HRMS (ESI)**: calcd. for $[C_{18}H_{19}ClO_2+Na]^+$, $[M+Na]^+$: 325.0966; found: 325.0966; ***R*f**: 0.16 (Hexane/EtOAc, 8:2).

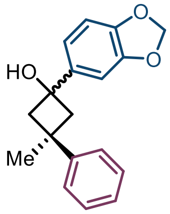

1w

***cis/trans*-1-(benzo[d][1,3]dioxol-5-yl)-3-methyl-3-phenylcyclobutan-1-ol (1w).** Following the General Procedure A. 5-bromobenzo[d][1,3]dioxole (753 mg, 453 µl, 3.74 mmol, 1.2 equiv.), n-BuLi (1.6 M in hexane, 2.34 ml, 3.74 mmol, 1.2 equiv.), 3-methyl-3-phenylcyclobutan-1-one (500 mg, 3.12 mmol, 1 equiv.). The crude mixture was purified by column chromatography (EtOAc-hexane, 5-20%) to afford a mixture of two inseparable diastereoisomers as a white solid in 85% yield, 2:1 dr (750 mg, 2.66 mmol).

^{1}H NMR (400 MHz, $CDCl_3$): (mixture of diastereomers, signals are reported as seen) δ 7.37 – 7.26 (m, 5H), 7.22 – 7.11 (m, 2.7H), 7.05 – 6.98 (m, 2H), 6.91 – 6.66 (m, 3H), 5.97 (s, 2H), 5.90 (s, 1H), 2.98 – 2.80 (m, 5H), 2.62 – 2.54 (m, 1H), 1.99 (s, 0.39H), 1.88 (s, 0.94H), 1.69 (s, 1.5H), 1.26 (s, 3H) ppm; **^{13}C NMR (101 MHz, $CDCl_3$)**: (mixture of diastereomers, signals are reported as seen) δ 151.7, 148.0, 147.8, 147.9, 146.6, 141.9, 140.7, 128.4, 128.3, 125.6, 125.4, 125.3, 125.2, 119.1, 118.0, 108.1, 108.0, 106.9, 105.9, 101.2, 101.1, 73.0, 72.5, 49.0, 48.5, 35.9, 34.2, 32.7, 31.4 ppm; **IR (ATR):** 3402, 2932, 1601, 1504, 1487, 1436, 1357, 1264, 1232, 1108, 101, 1037, 936, 867, 812, 764, 734, 700, 626, 545, 519 cm^{-1}; **HRMS (ESI)**: calcd. for $[C_{18}H_{18}O_3+Na]+$, $[M+Na]+$: 305.1148; found: 305.1148; ***R*f**: 0.15 (Hexane/EtOAc, 8:2); **m.p.**: 56–58 °C.

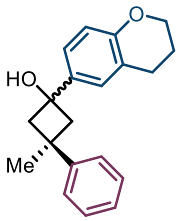

1x

***cis/trans*-1-(chroman-6-yl)-3-methyl-3-phenylcyclobutan-1-ol (1x).** Following the General Procedure A. 6-bromochromane (399 mg, 270 µl, 1.87 mmol, 1.2 equiv.), n-BuLi (1.6 M in hexane, 1.17 ml, 1.87 mmol, 1.2 equiv.), 3-methyl-3-phenylcyclobutan-1-one (250 mg, 1.56 mmol, 1 equiv.). The crude mixture was purified by column chromatography (EtOAc-hexane, 5-10%) to afford a mixture of two inseparable diastereoisomers as a white solid in 78% yield, 1:1 dr (357 mg, 1.21 mmol).

^{1}H NMR (400 MHz, CDCl$_3$): (mixture of diastereomers, signals are reported as seen) δ 7.37 – 7.25 (m, 7H), 7.22 – 7.13 (m, 5H), 7.05 – 6.97 (m, 2H), 6.81 (d, J = 8.4 Hz, 1H), 6.70 (d, J = 8.4 Hz, 1H), 4.22 – 4.17 (m, 2H), 4.16 – 4.10 (m, 2H), 2.99 – 2.89 (m, 4H), 2.86 – 2.80 (m, 4H), 2.74 – 2.68 (m, 2H), 2.63 – 2.54 (m, 2H), 2.06 – 2.00 (m, 2H), 1.98 – 1.93 (m, 2H), 1.86 (s, 0.38H), 1.82 (s, 0.74H), 1.70 (s, 3H), 1.26 (s, 3H) ppm; **^{13}C NMR (101 MHz, CDCl$_3$)**: (mixture of diastereomers, signals are reported as seen) δ 154.4, 154.0, 151.9, 139.4, 138.3, 128.4, 128.3, 127.5, 126.3, 125.5, 125.3, 125.2, 124.9, 123.9, 122.2, 121.9, 116.8, 116.7, 72.8, 72.2, 66.6, 66.6, 48.9, 48.4, 36.0, 34.2, 32.7, 31.4, 25.2, 25.1, 22.5, 22.4 ppm; **IR (ATR):** 3054, 2933, 1601, 1584, 1499, 1444, 1301, 1264, 1231, 1173, 1130, 1108, 1060, 1028, 1007, 900, 877, 825, 765, 732, 701, 546, 437 cm^{-1}; **HRMS (ESI)**: calcd. for $[C_{20}H_{22}O_2+Na]^+$, $[M+Na]^+$: 317.1512; found: 317.1511; ***R*f**: 0.18 (Hexane/EtOAc, 9:1); **m.p.**: 99–101°C.

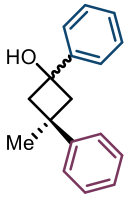

1y

***cis/trans*-3-Methyl-1,3-diphenylcyclobutan-1-ol (1y).** Following the General Procedure A. bromobenzene (972 g, 651.8 µl, 6.13 mmol, 1.5 equiv.), n-BuLi (1.6 M in hexane, 3.8 ml, 6.13 mmol, 1.5 equiv.), 3-methyl-3-phenylcyclobutan-1-one (654 mg, 4.09 mmol, 1 equiv.). The crude mixture was purified by column chromatography (EtOAc-hexane, 5-10%) to afford a mixture of two inseparable diastereoisomers as a white solid in 82% yield, 1.8:1 dr (800 mg, 3.36 mmol).

^{1}H NMR (400 MHz, CDCl$_3$): (mixture of diastereomers, signals are reported as seen) δ 7.59 – 7.52 (m, 1.9H), 7.46 – 7.36 (m, 1.9H), 7.40 – 7.23 (m, 8.4H), 7.24 – 7.12 (m, 3.6H), 3.04 – 2.83 (m, 5H), 2.66 – 2.58 (m, 1.3H), 2.01 (bs, 0.6H), 1.89 (bs, 1H), 1.72 (s, 1.7H), 1.26 (s, 3H) ppm; **^{13}C NMR (101 MHz, CDCl$_3$)**: (mixture of diastereomers, signals are reported as seen) δ 151.8, 151.7, 147.5, 146.5, 128.7, 128.5, 128.5, 128.4, 127.6, 127.2, 125.8, 125.6, 125.5, 125.4, 125.2, 124.8, 73.1, 72.6, 48.9, 48.5, 36.2, 34.5, 32.8, 31.6 ppm**;** ***R*f**: 0.19 (Hexane/EtOAc, 9:1);

Spectral data match the ones reported in the literature.[11]

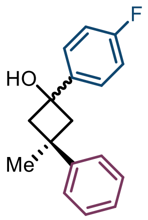

1z

***cis/trans*-1-(4-Fluorophenyl)-3-methyl-3-phenylcyclobutan-1-ol (1z).** Following the General Procedure A. 1-bromo-4-fluorobenzene (828 mg, 519 µl, 4.68 mmol, 1.5 equiv.), n-BuLi (1.6 M in hexane, 2.8 ml, 4.53 mmol, 1.45 equiv.), 3-methyl-3-phenylcyclobutan-1-one (500 mg, 3.12 mmol, 1 equiv.). The crude mixture was purified by column chromatography (EtOAc-hexane, 10%) to afford a mixture of two

inseparable diastereoisomers as a white solid in 64% yield, 1.8:1 dr (512 mg, 2.0 mmol).

^{1}H NMR (400 MHz, CDCl$_3$): (mixture of diastereomers, signals are reported as seen) δ 7.58 – 7.44 (m, 2H), 7.40 – 7.23 (m, 6.2H), 7.23 – 7.13 (m, 2.7H), 7.12 – 7.04 (m, 2H), 6.99 – 6.90 (m, 1.1H), 2.99 – 2.83 (m, 5.1H), 2.63 – 2.57 (m, 1.1H), 2.02 (s, 0.5H), 1.90 (s, 1H), 1.70 (s, 1.7H), 1.26 (s, 3H) ppm; **^{13}C NMR (101 MHz, CDCl$_3$)**: (mixture of diastereomers, signals are reported as seen) δ 163.4, 163.1, 161.0, 160.7, 151.5, 151.5, 143.4 (J = 3.4 Hz), 142.4 (d, J = 3.4 Hz), 128.5, 128.4, 127.7 (d, J = 8.2 Hz), 126.7 (d, J = 8.1 Hz), 125.7, 125.6, 125.4, 125.2, 115.5 (d, J = 21.3 Hz), 115.2 (d, J = 21.4 Hz), 72.7, 72.2, 49.2, 48.7, 36.0, 34.4, 32.9, 31.6 ppm; **^{19}F NMR (376 MHz, CDCl$_3$)**: (mixture of diastereomers) δ -115.02 (tt, J = 8.6, 5.3 Hz), -115.65 (tt, J = 8.6, 5.3 Hz) ppm; **IR (ATR):** 3353, 3058, 3024, 2971, 2933, 2865, 1602, 1510, 1445, 1422, 1373, 1225, 1159, 1114, 1028, 1014, 897, 836, 763, 748, 701, 610, 548, 511 cm^{-1}; **HRMS (ESI)**: calcd. for [$C_{17}H_{17}FO$+Na]$^+$, [M+Na]$^+$: 279.1161; found: 279.1152; ***R*f**: 0.19 (Hexane/EtOAc, 9:1); **m.p.**: 75–77°C.

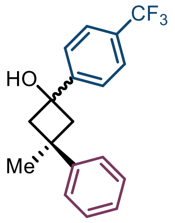

1aa

***cis/trans*-3-methyl-3-phenyl-1-(4-(trifluoromethyl)phenyl)cyclobutan-1-ol (1aa).** Following the General Procedure A. 1-iodo-4-(trifluoromethyl)benzene (1.02 g, 551 µl, 3.74 mmol, 1.2 equiv.), isopropylmagnesium chloride (2M in hexane, 1.87 ml, 3.74 mmol, 1.2 equiv.), 3-methyl-3-phenylcyclobutan-1-one (500 mg, 3.12 mmol, 1 equiv.). The crude mixture was purified by column chromatography (EtOAc-hexane, 5-10%) to afford a mixture of two inseparable diastereoisomers as a white solid in 66% yield, 1.2:1 dr (630 mg, 2.06 mmol).

^{1}H NMR (400 MHz, CDCl$_3$): (mixture of diastereomers, signals are reported as seen) δ 7.67 (s, 3.74H), 7.53 (d, *J* = 8.2 Hz, 1.55H), 7.42 (d, *J* = 8.2 Hz, 1.56H), 7.39 – 7.28 (m, 5.92H), 7.23 – 7.15 (m, 3.4H), 3.02 – 2.88 (m, 5.67H), 2.68 – 2.59 (m, 1.66H), 2.10 (s, 0.65H), 1.97 (s, 0.79H), 1.72 (s, 2.45H), 1.30 (s, 3H) ppm; **^{13}C NMR (101 MHz, CDCl$_3$)**: (mixture of diastereomers, signals are reported as seen) δ 151.2, 151.1, 150.2, 129.9, 129.6, 129.4, 129.1, 128.6, 128.5, 126.2, 125.8, 125.7 (q, *J* = 3.1 Hz)., 125.6, 125.5, 125.4, 125.3, 125.2, 125.1, 72.7, 72.4, 49.2, 48.9, 36.1, 34.8, 33.0, 31.6**; ^{19}F NMR (376 MHz, CDCl$_3$)**: δ -62.34 ppm; **IR (ATR):** 3347, 3025, 2972, 1619, 1495, 1407, 1323, 1163, 1110, 1067, 1015, 909, 843, 763, 741, 699, 606, 544, 427 cm^{-1}; **HRMS (ESI)**: calcd. for [$C_{18}H_{17}F_3O$+Na]+, [M+H]+: 329.1124; found: 329.1125; ***R*f**: 0.20 (Hexane/EtOAc, 9:1); **m.p.**: 87–90°C.

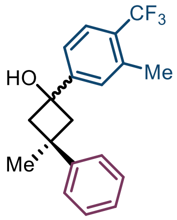

1ab

***cis/trans*-3-methyl-1-(3-methyl-4-(trifluoromethyl)phenyl)-3-phenylcyclobutan-1-ol (1ab).** Following the General Procedure A. 4-bromo-2-methyl-1-(trifluoromethyl)benzene (895 mg, 582 µl, 3.74 mmol, 1.2 equiv.), n-BuLi (1.6 M in hexane, 2.34 ml, 3.74 mmol, 1.2 equiv.), 3-methyl-3-phenylcyclobutan-1-one (500 mg, 3.12 mmol, 1 equiv.). The crude mixture was purified by column chromatography (EtOAc-hexane, 5-10%) to afford a mixture of two inseparable diastereoisomers as a white solid in 88% yield, 1.2:1 dr (880 mg, 2.75 mmol).

^{1}H NMR (400 MHz, $CDCl_3$): (mixture of diastereomers, signals are reported as seen) δ 7.64 (d, *J* = 7.9 Hz, 1H), 7.50 (d, *J* = 8.1 Hz, 0.88H), 7.44 (d, *J* = 10.0 Hz, 2H), 7.40 – 7.28 (m, 6H), 7.23 – 7.17 (m, 5H), 3.01 – 2.85 (m, 5.84H), 2.65 – 2.58 (m, 2H), 2.53 (s, 3H), 2.42 (s, 2.74H), 2.04 (s, 0.66H), 1.91 (s, 0.93H), 1.72 (s, 2.58H), 1.29 (s, 3H) ppm; **^{13}C NMR (101 MHz, $CDCl_3$)**: (mixture of diastereomers, signals are reported as seen) δ 151.3, 151.2, 150.9, 149.9, 137.12, 136.9, 129.4, 128.6, 128.4, 128.4, 128.3, 127.9, 127.3, 126.3, 126.2, 126.0 (dt, *J* = 11.5, 3.9 Hz), 125.8, 125.6, 125.4, 125.2, 123.0, 122.2, 72.6, 72.3, 49.1, 48.7, 36.1, 34.7, 33.0, 31.8, 19.6, 19.5; **^{19}F (376 MHz, $CDCl_3$)**: δ -61.42 ppm; **IR (ATR):** 3341, 2971, 1617, 1578, 1495, 1445, 1313, 1267, 1170, 1111, 1040, 890, 835, 763, 739, 700, 668, 629, 543, 449 cm^{-1}; **HRMS (ESI)**: calcd. for $[C_{19}H_{19}F_3O+Na]^+$, $[M+Na]^+$: 343.1280; found: 343.1276; ***R*f**: 0.19 (Hexane/EtOAc, 8:2); **m.p.**: 76–78 °C.

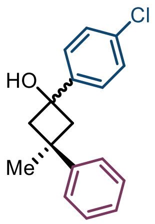

1ac

***cis/trans*-1-(4-Chlorophenyl)-3-methyl-3-phenylcyclobutan-1-ol (1ac)**. Following the General Procedure A. 1-bromo-4-chlorobenzene (905 mg, 4.68 mmol, 1.5 equiv.), n-BuLi (1.6 M in hexane, 2.8 ml, 4.53 mmol, 1.45 equiv.), 3-methyl-3-phenylcyclobutan-1-one (500 mg, 3.12 mmol, 1 equiv.). The crude mixture was purified by column chromatography (EtOAc-hexane, 15%) to afford a mixture of two inseparable diastereoisomers as a light yellow solid in 84% yield, 1.76:1 dr (714 mg, 3.12 mmol).

^{1}H NMR (400 MHz, $CDCl_3$): (mixture of diastereomers, signals are reported as seen) δ 7.51 – 7.43 (m, 1.9H), 7.40 – 7.26 (m, 6.9H), 7.25 – 7.12 (m, 5.4H), 2.99 – 2.80 (m, 4.9H), 2.64 – 2.49 (m, 1.4H) 2.07 (bs, 0.6H), 1.95 (bs, 0.9H), 1.69 (s, 1.7H), 1.26 (s, 3H) ppm; **^{13}C NMR (101 MHz, $CDCl_3$)**: (mixture of diastereomers, signals are reported as seen) δ 151.4, 151.4, 146.0, 145.0, 133.4, 132.9, 128.8, 128.5, 128.4, 128.3, 127.4, 126.4, 125.7, 125.6, 125.5, 125.4, 125.2, 72.6, 72.2, 49.1, 48.7, 36.0, 34.5, 33.0, 31.6 ppm; ***R*f**: 0.35 (Hexane/EtOAc, 85:15).

Spectral data match the ones reported in the literature.[11]

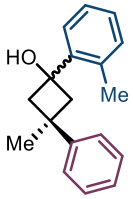

1ad

***cis/trans* 3-methyl-3-phenyl-1-(o-tolyl)cyclobutan-1-ol (1ad).** Following the General Procedure A. 1-bromo-2-methylbenzene (641 mg, 451 µl, 3.74 mmol, 1.2 equiv.), n-BuLi (1.6 M in hexane, 2.34 ml, 3.74 mmol, 1.2 equiv.), 3-methyl-3-phenylcyclobutan-1-one (500 mg, 3.12 mmol, 1 equiv.). The crude mixture was purified by column chromatography (EtOAc-hexane 5-10%) to afford a mixture of two inseparable diastereoisomers as a yellow oil 76 % yield, 1.3:1 dr (600 mg, 2.38 mmol).

^{1}H NMR (400 MHz, $CDCl_3$): (mixture of diastereomers, signals are reported as seen) δ 7.47 – 7.41 (m, 1H), 7.38 (d, *J* = 4.3 Hz, 4H), 7.32 – 7.26 (m, 1.76H), 7.24 – 7.10 (m, 9.45H), 3.12 – 2.95 (m, 5.64H), 2.76 – 2.70 (m, 1.54H), 2.47 (s, 3H), 2.45 (s, 2.36H), 1.91 (s, 0.72H), 1.83 (s, 0.93H), 1.77 (s, 2.35H) 1.31 (s, 3H) ppm; **13C NMR (101 MHz, $CDCl_3$)**: (mixture of diastereomers, signals are reported as seen) δ 152.4, 151.4, 144.0, 143.1, 137.4, 136.7, 132.1, 131.8, 128.5, 128.3,

128.0, 127.8, 125.7, 125.6, 125.6, 125.5, 125.4, 125.3, 125.1, 74.5, 73.6, 49.0, 47.8, 36.9, 35.4, 32.5, 32.9, 20.5, 20.1 ppm; ***R*f**: 0.20 (Hexane/EtOAc, 9:1).

Spectral data match the ones reported in the literature.[12]

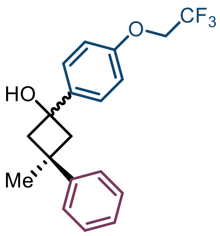

1ae

***cis/trans*-3-methyl-3-phenyl-1-(4-(2,2,2-trifluoroethoxy)phenyl)cyclobutan-1-ol (1ae).** Following the General Procedure A. 1-bromo-4-(2,2,2-trifluoroethoxy)benzene (915 mg, 3.59 mmol, 1.15 equiv.), n-BuLi (1.6 M in hexane, 2.24 ml, 3.59 mmol, 1.15 equiv.), 3-methyl-3-phenylcyclobutan-1-one (600 mg, 3.12 mmol, 1 equiv.). The crude mixture was purified by column chromatography (EtOAc-hexane, 10-20%) to afford the mixture of diastereoisomers of the product as a yellow gel in 52% yield, 2.1:1 dr (543 mg, 1.61 mmol).

^{1}H NMR (400 MHz, $CDCl_3$): (mixture of diastereomers, signals are reported as seen) δ 7.54 – 7.47 (m, 2.1H), 7.38 – 7.25 (m, 7H), 7.22 – 7.04 (m, 3.2H), 7.00 – 6.94 (m, 2.3H), 6.87 – 6.80 (m, 1H), 4.37 (q, *J* = 8.1 Hz, 2H), 4.29 (q, *J* = 8.1 Hz, 1.2H), 2.99 – 2.83 (m, 5.4H), 2.64 – 2.57 (m, 1.2H), 1.91 (s, 0.5H), 1.80 (s, 1H), 1.70 (s, 1.4H), 1.25 (s, 3H) ppm; **^{13}C NMR (101 MHz, $CDCl_3$)**: (mixture of diastereomers, signals are reported as seen) δ 156.8, 156.5, 151.7, 151.6, 141.9, 140.8, 128.5, 128.4, 127.5, 126.4, 125.7, 125.5, 125.3, 125.2, 115.0, 114.8, 72.6, 72.1, 66.0 (q, J = 35.7 Hz), 49.0, 48.6, 36.0, 34.3, 32.9, 31.5 ppm; **^{19}F NMR (376 MHz, $CDCl_3$)**: (mixture of diastereomers) δ -73.83 (t, J = 8.2 Hz, 3F), -73.87 (t, J = 8.2 Hz, 1.6F) ppm; **IR (ATR):** 3362, 3058, 3024, 2933, 2866, 1609, 1588, 1512, 1495, 1459, 1445, 1423, 1373, 1282, 1232, 1109, 1077, 1027, 973, 909, 896, 864, 831, 763, 730, 700, 666, 637, 620, 547, 409 cm^{-1}; **HRMS (ESI)**: calcd. for $[C_{19}H_{19}F_3O_2+Na]^+$, $[M+H]^+$: 359.1235; found: 359.1229; ***R*f**: 0.15 (Hexane/EtOAc, 8:2).

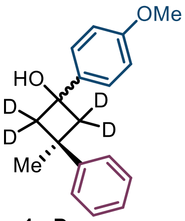

1a-D

***cis/trans*-1-(4-methoxyphenyl)-3-methyl-3-phenylcyclobutan-2,2,4,4-d$_4$-1-ol (1a-D).** Following the General Procedure A. 1-bromo-4-methoxybenzene (205 mg, 137 µl, 1.10 mmol, 1.2 equiv.), n-BuLi (1.6 M in hexane, 685 µl, 1.10 mmol, 1.2 equiv.), 3-methyl-3-phenylcyclobutan-1-one-2,2,4,4-d4 (150 mg, 0.913 mmol, 1 equiv.). The crude mixture was purified by column chromatography (EtOAc-hexane, 5-20%) to afford the mixture of diastereoisomers of the product as a white solid in 96% yield, 1.8:1 dr (240 mg, 0.881 mmol).

^{1}H NMR (400 MHz, $CDCl_3$): (mixture of diastereomers, signals are reported as seen) δ 7.49 (d, *J* = 8.7 Hz, 2H), 7.37 (t, *J* = 7.6 Hz, 2H), 7.34 – 7.29 (m, 3H), 7.28 – 7.14 (m, 4H), 6.95 (d, *J* = 8.7 Hz, 2H), 6.82 (d, *J* = 8.7 Hz, 1H), 3.84 (s, 3H), 3.77 (s, 1.65H), 2.97 – 2.3 (m, 0.24H), 2.59 (s, 0.06H), 2.19 (s, 0.56H), 2.04 (s, 0.87H), 1.72 (s, 1.67H), 1.26 (s, 3H) ppm; **^{13}C NMR (101 MHz, $CDCl_3$)**: (mixture of diastereomers, signals are reported as seen) δ 158.9, 158.6, 152.0, 151.8, 140.0, 138.9, 128.5, 128.3, 127.3, 126.2, 125.6, 125.4, 125.4, 125.3, 114.0, 72.4, 71.8, 55.4, 55.3, 48.1 (m, $-CD_2$), 35.5, 33.7, 32.7, 31.2 ppm; **IR (ATR):** 3381, 2953, 2835, 2237, 1609, 1581, 1511, 1445, 1371, 1297, 1244, 1177, 1136, 1074, 1027, 957, 831, 801, 753, 700, 612, 546 cm^{-1}; **HRMS**

(ESI): calcd. for $[C_{18}H_{16}D_4O_2+Na]^+$, $[M+Na]^+$: 295.1607; found: 295.1602; ***R*f**: 0.17 (Hexane/EtOAc, 9:1); **m.p.**: 73–75 °C.

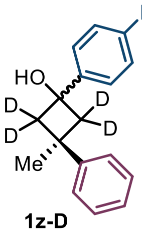

1z-D

***cis/trans*-1-(4-fluorophenyl)-3-methyl-3-phenylcyclobutan-2,2,4,4-d4-1-ol (1z-D).** Following the General Procedure A. 1-bromo-4-fluorobenzene (320 mg, 201 µl, 1.83 mmol, 1.2 equiv.), n-BuLi (1.6 M in hexane, 1.14 ml, 1.83 mmol, 1.2 equiv.), 3-methyl-3-phenylcyclobutan-1-one-2,2,4,4-d4 (250 mg, 1.52 mmol, 1 equiv.). The crude mixture was purified by column chromatography (EtOAc-hexane, 5-20%) to afford the mixture of diastereoisomers of the product as a white solid in 71% yield, 1.8:1 dr (280 mg, 1.08 mmol).

^{1}H NMR (400 MHz, $CDCl_3$): (mixture of diastereomers, signals are reported as seen) δ 7.56 – 7.47 (m, 2H), 7.42 – 7.16 (m, 9H), 7.09 (t, *J* = 8.7 Hz, 2H), 6.95 (t, *J* = 8.7 Hz, 1H), 2.94 – 2.83 (m, 0.19H), 2.64 – 2.57 (m, 0.07H), 2.12 (s, 0.47H), 1.99 (s, 0.87H), 1.70 (s, 1.67H), 1.26 (s, 3H) ppm; **^{13}C NMR (101 MHz, $CDCl_3$)**: (mixture of diastereomers, signals are reported as seen) δ 162.2 (d, *J* = 246.1 Hz), 161.9 (d, *J* = 245.4 Hz), 151.5, 151.5,143.4 (d, *J* = 3.3 Hz), 142.3 (d, *J* = 3.1 Hz), 128.5, 128.4, 127.7 (d, *J* = 8.2 Hz), 126.7 (d, *J* = 8.1 Hz), 125.7, 125.5, 125.4, 125.2, 115.5 (d, *J* = 21.2 Hz), 115.2 (d, *J* = 21.2 Hz), 72.3, 71.8, 48.3 (m, $-CD_2$), , 35.5, 33.9, 32.7, 31.4 ppm; **^{19}F NMR (376 MHz, $CDCl_3$):** (mixture of diastereomers) δ -114.98, -115.64 ppm; **IR (ATR):** 3347, 2955, 2360, 2238, 1601, 1509, 1445, 1372, 1228, 1159, 1073, 1027, 958, 834, 752, 699, 603, 540, 467 cm^{-1}; **HRMS (ESI)**: calcd. for $[C_{17}H_{13}D_4FO+Na]^+$, $[M+Na]^+$: 283.1407; found: 283.1404; ***R*f**: 0.19 (Hexane/EtOAc, 9:1); **m.p.**: 64–66°C.

General procedure C for the synthesis of trichloroacetimidates

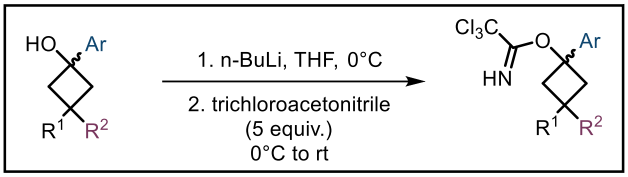

A flame-dried two-neck flask equipped with a stirring bar was charged with the cyclobutanol and anhydrous THF (0.12 M) under an Ar atmosphere. After cooling to 0 °C, n-BuLi (1.0 – 1.1 equiv) was added dropwise. The resulting mixture was stirred at 0 °C for 1 h, followed by the dropwise addition of trichloroacetonitrile (5 equiv). Stirring continued for 1 h at 0 °C, and then the reaction was allowed to reach room temperature and stirred for 2-12 h. The solvent was evaporated under reduced pressure, and the crude mixture was directly purified by column chromatography on neutral Al_2O_3 Brockman activity grade V using hexane 100%, loading the crude with hexane and a small amount of toluene.

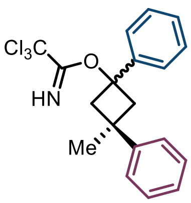

1af

***cis/trans*-3-methyl-1,3-diphenylcyclobutyl 2,2,2-trichloroacetimidate (1af).** Following the General Procedure C. 3-methyl-1,3-diphenylcyclobutan-1-ol (350 mg, 1.47 mmol, 1 equiv.), n-BuLi (1.6 M in hexane, 1.01 ml, 1.62 mmol, 1.1 equiv.), 2,2,2-trichloroacetonitrile (1.06 g, 736 µl, 7.34 mmol, 5.0 equiv.), 12 h at room temperature. The crude mixture was purified by column chromatography on Al_2O_3 Brockman activity grade V (hexane 100%) to afford a mixture of two inseparable diastereoisomers as a white solid in 41% yield, 1.6:1 dr (230 mg, 601 µmol).

^{1}H NMR (400 MHz, C_6D_6): (mixture of diastereomers, signals are reported as seen) δ 8.14 (s, 0.53H), 7.99 (s, 0.7H), 7.63 (d, J = 7.1 Hz, 2H), 7.36 (d, J = 8.2 Hz, 1.29H), 7.18 (t, J = 7.7 Hz, 2H), 7.14 – 7.02 (m, 7H), 6.99 – 6.90 (m, 4H), 3.19 (d, J = 13.9 Hz, 2H), 3.07 (d, J = 14.5 Hz, 1.3H), 2.94 (dd, J = 14.3, 3.1 Hz, 3.32H), 1.55 (s, 1.9H), 1.10 (s, 3H) ppm; **^{13}C NMR (101 MHz, C_6D_6)**: (mixture of diastereomers, signals are reported as seen) δ 158.7, 158.6, 151.3, 150.8, 142.8, 142.1, 128.4, 128.4, 127.9, 127.6, 127.4, 127.2, 125.6, 125.5, 125.1, 125.0, 92.3, 92.0, 83.0, 82.0, 46.3, 45.9, 36.6, 35.7, 32.5, 30.8 ppm; **IR (ATR):** 3333, 3025, 2954, 1659, 1601, 1533, 1494, 1464, 1316, 1240, 1073, 1022, 960, 883, 837, 791, 757, 695, 647, 545, 436 cm^{-1}; **HRMS (ESI)**: calcd. for $[C_{19}H_{18}Cl_3NO+Na]^+$, [M+Na]+: 404.0346; found: 404.0341; ***R*f**: 0.26 (Hexane); **m.p.**: 77–79°C.

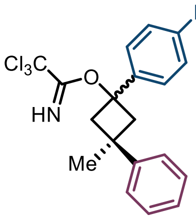

1ag

***cis/trans*-1-(4-Fluorophenyl)-3-methyl-3-phenylcyclobutyl 2,2,2-trichloroacetimidate (1ag).** Following the General Procedure C. 1-(4-fluorophenyl)-3-methyl-3-phenylcyclobutan-1-ol (300 mg, 1.17 mmol, 1 equiv.), n-BuLi (1.6 M in hexane, 805 µl, 1.29 mmol, 1.1 equiv.), 2,2,2-trichloroacetonitrile (845 mg, 587 µl, 5.85 mmol, 5.0 equiv.), 12 h at room temperature. The crude mixture was purified by column chromatography on Al_2O_3 Brockman activity grade V (hexane 100%) to afford a mixture of two inseparable diastereoisomers as a white solid in 39% yield, 1.3:1 dr (183 mg, 0.46 mmol).

^{1}H NMR (400 MHz, C_6D_6): (mixture of diastereomers, signals are reported as seen) δ 8.18 (s, 0.7H), 8.03 (s, 0.9H), 7.53 – 7.43 (m, 1.8H), 7.25 – 7.17 (m, 3.1H), 7.16 – 7.10 (m, 3.9H), 7.08 – 6.96 (m, 3.6H), 6.91 – 6.84 (m, 1.9H), 6.77 – 6.65 (m, 1.6H), 3.25 – 3.15 (m, 2H), 3.06 – 2.83 (m, 5.1H), 1.59 (s, 2.3H), 1.11 (s, 3H) ppm; **^{13}C NMR (101 MHz, C_6D_6)**: (mixture of diastereomers, signals are reported as seen) δ 162.7 (d, J = 246.6 Hz), 162.38 (d, J = 245.6 Hz), 159.0, 158.9, 151.5, 150.9, 138.6 (d, J = 3.4 Hz), 138.0 (d, J = 3.4 Hz), 129.8 (d, J = 8.2 Hz), 128.8, 128.7, 128.2, 128.1, 126.0, 125.3, 125.2, 114.9 (d, J = 21.4 Hz), 114.9 (d, J = 21.4 Hz), 92.4, 92.2, 82.7, 81.6, 46.6, 46.1, 36.9, 35.9, 32.7, 31.0 ppm; **^{19}F NMR (376 MHz, C_6D_6)**: (mixture of diastereomers) δ -114.12 (tt, J = 8.6, 5.3 Hz), -114.93 (tt, J = 8.5, 5.3 Hz) ppm; **IR (ATR):** 3334, 2956, 1660, 1602, 1511, 1495, 1445, 1419, 1374, 1317, 1289, 1230, 1160, 1071, 1026, 961, 885, 844, 828, 793, 763, 700, 649, 610, 549, 510, 434, 404 cm^{-1}; **HRMS (ESI)**: calcd. for $[C_{19}H_{17}Cl_3FNO+Na]^+$, $[M+Na]^+$: 422.0257; found: 422.0251; ***R*f**: 0.24 (Hexane); **m.p.**: 107–110°C.

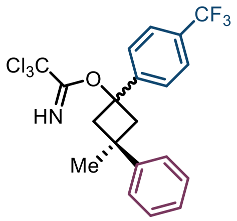

1ah

***trans/cis*-3-methyl-3-phenyl-1-(4-(trifluoromethyl)phenyl)cyclobutyl 2,2,2-trichloroacetimidate (1ah).** Following the General Procedure C. 3-methyl-3-phenyl-1-(4-(trifluoromethyl)phenyl)cyclobutan-1-ol (300 mg, 979 µmol, 1 equiv.), n-BuLi (1.6 M in hexane, 612 µl, 979 µmol, 1.0 equiv.), 2,2,2-trichloroacetonitrile (707 mg, 491 µl, 4.90 mmol, 5.0 equiv.), 2 h at room temperature. The crude mixture was purified by column chromatography on Al_2O_3 Brockman activity grade V (hexane 100%) to afford a mixture of two inseparable diastereoisomers as a white solid in 36% yield, 1.2:1 dr (158 mg, 979 µmol).

^{1}H NMR (400 MHz, C_6D_6): (mixture of diastereomers, signals are reported as seen) δ 8.10 (s, 1H), 7.94 (s, 1H), 7.47 (d, *J* = 8.2 Hz, 2H), 7.39 (d, *J* = 8.1 Hz, 2H), 7.21 – 7.03 (m, 9H), 6.98 (t, *J* = 7.0 Hz, 2H), 6.92 (d, *J* = 6.9 Hz, 2H), 3.10 (d, *J* = 14.0 Hz, 2H), 2.95 – 2.71 (m, 6H), 1.51 (s, 3H), 1.04 (s, 2.5H) ppm; **^{13}C NMR (101 MHz, C_6D_6)**: (mixture of diastereomers, signals are reported as seen) δ 158.6, 158.5, 150.8, 150.4, 146.6, 146.1, 129.8, 129.5, 129.3, 128.9, 128.5, 128.4, 127.5, 127.3, 125.6 (t, *J* = 4.5 Hz), 125.1, 125.0 (d, *J* = 4.4 Hz), 124.9, 124.8, 91.9, 91.6, 82.1, 81.2, 46.1, 45.7, 36.5, 35.7, 32.5, 30.9; **^{19}F NMR (376 MHz, C_6D_6)**: (mixture of diastereomers) δ -62.03, -62.10 ppm; **IR (ATR):** 3363, 2956, 1663, 1619, 1494, 1409, 1325, 1165, 1123, 1066, 1028, 964, 887, 848, 794, 776, 763, 700, 649, 606, 545, 433 cm^{-1}; **HRMS (ESI)**: calcd. for $[C_{20}H_{17}Cl_3F_3NO+Na]^+$, $[M+Na]^+$: 472.0220; found: 472.0221; ***R*f**: 0.27 (Hexane); **m.p.**: 93–96°C.

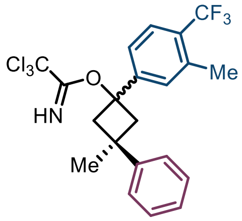

1ai

***trans/cis*-3-methyl-1-(3-methyl-4-(trifluoromethyl)phenyl)-3-phenylcyclobutyl 2,2,2-trichloroacetimidate (1ai).** Following the General Procedure C. 3-methyl-1-(3-methyl-4-(trifluoromethyl)phenyl)-3-phenylcyclobutan-1-ol (300 mg, 0.936 mmol, 1.0 equiv.), n-BuLi (1.6 M in hexane, 644 µl, 1.03 mmol, 1.0 equiv.), 2,2,2-trichloroacetonitrile (676 mg, 469 µl, 4.68 mmol, 5.0 equiv.), 2 h at room temperature. The crude mixture was purified by column chromatography on Al_2O_3 Brockman activity grade V (hexane 100%) to afford a mixture of two inseparable diastereoisomers as a yellow gel in 55% yield, 1.1:1 dr (240 mg, 516 µmol).

^{1}H NMR (400 MHz, C_6D_6): (mixture of diastereomers, signals are reported as seen) δ 8.13 (s, 0.91H), 7.98 (s, 0.81H), 7.50 – 7.43 (m, 2H), 7.30 (dd, *J* = 22.7, 8.3 Hz, 2H), 7.12 (d, *J* = 7.2 Hz, 3H), 7.08 – 7.05 (m, 3H), 7.04 – 6.90 (m, 6H), 3.14 (d, *J* = 14.0 Hz, 1.8H), 2.98 (d, *J* = 14.5 Hz, 2H), 2.89 – 2.75 (m, 4H), 2.28 (s, 3H), 2.12 (s, 2.72H), 1.52 (s, 3H), 1.08 (s, 2.70H) ppm; **^{13}C NMR (101 MHz, C_6D_6)**: (mixture of diastereomers, signals are reported as seen) δ 158., 158.6, 150.8, 150.4, 146.5, 146.1, 136.5, 130.3, 128.8, 128.5, 127.9, 127.6, 127.1, 126.1, 125.7 (q, *J* = 6.9 Hz), 125.6, 125.0, 124.5, 122.8, 91.9, 91.6, 82.1, 81.2, 46.1, 45.8, 36.5, 35.8, 32.6, 31.0, 19.2, 19.0 ppm; **^{19}F NMR (376 MHz, C_6D_6)**: (mixture of diastereomers) δ -61.05, -61.10 ppm; **IR (ATR)**: 3336, 2957, 1750, 1663, 1617, 1578, 1496, 1446, 1406, 1313, 1272, 1170, 1117, 1071, 1041, 694, 871, 843, 828, 794, 739, 700, 648, 630, 545, 449 cm^{-1}; **HRMS (ESI)**: calcd. for $[C_{21}H_{19}Cl_3F_3NO+Na]^+$, $[M+Na]^+$: 486.0377; found: 486.0374; ***R*f**: 0.22 (Hexane).

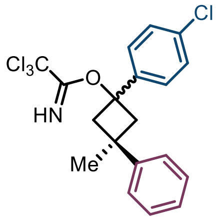
1aj

***cis/trans*-1-(4-chlorophenyl)-3-methyl-3-phenylcyclobutyl 2,2,2-trichloroacetimidate (1aj).** Following the General Procedure C. 1-(4-chlorophenyl)-3-methyl-3-phenylcyclobutan-1-ol (300 mg, 1.10 mmol, 1 equiv.), n-BuLi (1.6 M in hexane, 756 µl, 1.21 mmol, 1.1 equiv.), 2,2,2-trichloroacetonitrile (794 mg, 551 µl, 5.50 mmol, 5.0 equiv.), 12h at room temperature. The crude mixture was purified by column chromatography on Al_2O_3 Brockman activity grade V (hexane 100%) to afford a mixture of two inseparable diastereoisomers as a white solid in 19% yield, 1.2:1 dr (87 mg, 0.21 mmol).

^{1}H NMR (400 MHz, C_6D_6): (mixture of diastereomers, signals are reported as seen) δ 8.17 (s, 0.9H), 8.02 (s, 0.9H), 7.45 – 7.37 (m, 1.9H), 7.23 – 7.17 (m, 4H), 7.15 – 7.10 (m, 5H), 7.07 – 6.95 (m, 6H), 3.22 – 3.11 (m, 1.8H), 3.07 – 2.79 (m, 5.7H), 1.58 (s, 2.6H), 1.10 (s, 3H) ppm; **^{13}C NMR (101 MHz, C_6D_6)**: (mixture of diastereomers, signals are reported as seen) δ 159.0, 158.9, 151.4, 150.8, 141.4, 140.8, 134.0, 133.4, 129.3, 128.8, 128.7, 128.4, 127.6, 126.0, 126.0, 125.3, 125.2, 92.3, 92.1, 82.6, 81.5, 46.5, 46.0, 36.8, 35.9, 32.7, 31.0 ppm; **IR (ATR):** 3333, 2955, 1661, 1601, 1494, 1445, 1419, 1399, 1316, 1153, 1073, 1027, 1013, 961, 885, 844, 822, 793, 763, 699, 649, 546, 440, 419, 405 cm^{-1}; **HRMS (ESI)**: calcd. for $[C_{19}H_{17}Cl_4NO+Na]^+$, $[M+Na]^+$: 437.9962; found: 437.9955; ***R*f**: 0.26 (Hexane); **m.p.**: 113–116 °C.

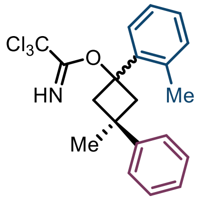
1ak

***cis/trans*-3-methyl-3-phenyl-1-(o-tolyl)cyclobutyl 2,2,2-trichloroacetimidate (1ak).** Following the General Procedure C. 3-methyl-3-phenyl-1-(o-tolyl)cyclobutan-1-ol (300 mg, 1.19 mmol, 1 equiv.), n-BuLi (1.6 M in hexane, 743 µl, 1.19 mmol, 1.1 equiv.), 2,2,2-trichloroacetonitrile (858 mg, 596 µl, 5.94 mmol, 5.0 equiv.), 2 h at room temperature. The crude mixture was purified by column chromatography on Al_2O_3 Brockman activity grade V (hexane 100%) to afford a mixture of two inseparable diastereoisomers as a white gel in 34% yield, 1.4:1 dr (161 mg, 406 µmol).

^{1}H NMR (400 MHz, C_6D_6): (mixture of diastereomers, signals are reported as seen) δ 8.07 (s, 0.67H), 7.94 (s, 0.91H), 7.79 (d, J = 7.2 Hz, 1H), 7.57 – 7.51 (m, 0.76H), 7.13 – 7.02 (m, 8H), 7.00 – 6.90 (m, 6H), 3.24 (d, J = 13.9 Hz, 2H), 3.10 (s, 4.77H), 2.41 (s, 3H), 2.36 (s, 2.17H), 1.60 (s, 2.13H), 1.09 (s, 3H) ppm; **^{13}C NMR (101 MHz, C_6D_6)**: (mixture of diastereomers, signals are reported as seen) δ 158.7, 158.6, 151.3, 151.1, 138.4, 138.3, 137.2, 136.7, 131.7, 131.2, 130.3, 129.9, 128.4, 128.3, 128.2, 125.5, 125.4, 125.1, 124.9, 124.5, 124.4, 92.5, 92.2, 84.6, 83.1, 36.9, 35.8, 31.8, 31.2, 20.3, 20.2 ppm; **IR (ATR):** 3334, 3058, 3022, 2954, 1725, 1660, 1493, 1445, 1318, 1241, 1159, 1074, 1027, 595, 836, 795, 759, 699, 654, 565, 548, 459 cm^{-1}; **HRMS (ESI)**: calcd. for $[C_{20}H_{20}Cl_3NO+Na]^+$, $[M+Na]^+$: 418.0503; found: 418.0504; ***R*f**: 0.25 (Hexane).

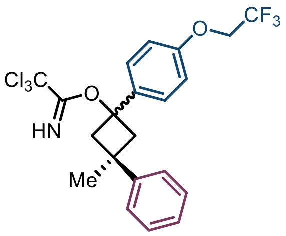

1al

***cis/trans*-3-methyl-3-phenyl-1-(4-(2,2,2-trifluoroethoxy)phenyl)cyclobutyl 2,2,2-trichloroacetimidate (1al).** Following the General Procedure C. 3-methyl-3-phenyl-1-(o-tolyl)cyclobutan-1-ol (320 mg, 0.951 mmol, 1 equiv.), n-BuLi (1.6 M in hexane, 654 µl, 1.05 mmol, 1.1 equiv.), 2,2,2-trichloroacetonitrile (858 mg, 477 µl, 4.76 mmol, 5.0 equiv.), 2 h at room temperature. The crude mixture was purified by column chromatography on Al_2O_3 Brockman activity grade V neutralized with 5% Et_3N-Hexane and eluted with Hexane (100%) to afford a mixture of two inseparable diastereoisomers as a yellow gel in 48% yield, 1.4:1 dr (222 mg, 462 µmol).

^{1}H NMR (400 MHz, C_6D_6): (mixture of diastereomers, signals are reported as seen) δ 8.16 (s, 0.67H), 8.02 (s, 0.93H), 7.49 (d, J = 8.8 Hz, 2H), 7.20 (d, J = 8.8 Hz, 1.6H), 7.16 – 7.04 (m, 4.6H), 7.03 – 6.93 (m, 4H), 6.54 (d, J = 8.8 Hz, 2H), 6.38 (d, J = 8.8 Hz, 1.5H), 3.53 (q, J = 8.2 Hz, 1.6H), 3.44 (q, J = 8.2 Hz, 2H), 3.19 (d, J = 13.9 Hz, 2H), 3.06 (d, J = 14.3 Hz, 1.5H), 2.93 (t, J = 14.1 Hz, 3.6H), 1.54 (s, 2.2H), 1.12 (s, 3H) ppm; **^{13}C NMR (101 MHz, C_6D_6)**: (mixture of diastereomers, signals are reported as seen) δ 158.9, 158.8, 156.7, 156.4, 151.3, 150.6, 136.5, 135.8, 129.1, 128.5, 128.4, 127.4, 125.6, 125.1, 124.9, 114.1, 114.1, 92.3, 92.0, 82.5, 81.5, 65.1 (dq, J = 35.4, 3.0 Hz), 46.4, 46.0, 36.5, 35.7, 32.6, 30.6 ppm; **^{19}F NMR (376 MHz, C_6D_6)**: (mixture of diastereomers) δ -73.73, -73.80 ppm; **IR (ATR)**: 3334, 2955, 1660, 1608, 1513, 1495, 1458, 1445, 1420, 1285, 1234, 1162, 1073, 1027, 962, 883, 842, 794, 762, 700, 667, 649, 549, 435 cm^{-1}; **HRMS (ESI)**: calcd. for $[C_{20}H_{19}Cl_3F_3NO+Na]^+$, $[M+Na]^+$: 502.0326; found: 502.0404; ***R*f**: 0.17 (Hexane).

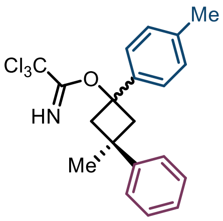

1am

***cis/trans*-3-methyl-3-phenyl-1-(*p*-tolyl)cyclobutyl 2,2,2-trichloroacetimidate (1am).** Following the General Procedure C. 3-methyl-3-phenyl-1-(*p*-tolyl)cyclobutan-1-ol (260 mg, 1.03 mmol, 1 equiv.), n-BuLi (1.6 M in hexane, 708 µl, 1.13 mmol, 1.1 equiv.), 2,2,2-trichloroacetonitrile (744 mg, 516 µl, 5.15 mmol, 5.0 equiv.), 2 h at room temperature. The crude mixture was purified by column chromatography on Al_2O_3 Brockman activity grade V neutralized with 10% Et_3N-Hexane and eluted with Hexane (100%) to afford a mixture of two inseparable diastereoisomers as a yellow gel in 49% yield, 1.6:1 dr (200 mg, 504 µmol).

^{1}H NMR (400 MHz, C_6D_6): (mixture of diastereomers, signals are reported as seen) δ 8.16 (s, 0.56H), 8.01 (s, 0.88H), 7.58 (d, J = 7.9 Hz, 2H), 7.30 (d, J = 7.7 Hz, 1H), 7.16 – 6.92 (m, 9.5H), 6.86 (d, J = 7.9 Hz, 2H), 3.21 (d, J = 14.0 Hz, 2H), 3.10 (d, J = 14.5 Hz, 1.53H), 2.98 (d, J = 13.9 Hz, 3.47H), 2.06 (s, 3H), 1.96 (s, 2H), 1.56 (s, 2H), 1.13 (s, 3H) ppm; **^{13}C NMR (101 MHz, C_6D_6)**: (mixture of diastereomers, signals are reported as seen) δ 158.8, 158.7, 151.5, 150.8, 139.8, 139.1, 137.2, 136.6, 128.6, 128.5, 128.4, 128.3, 125.8, 125.5, 125.1, 125.0, 83.0, 81.9, 46.4, 46.0, 36.6, 35.7, 32.5, 30.7, 20.8, 20.7 ppm; **IR (ATR)**: 3333, 2954, 2360, 1748, 1660, 1495, 1518, 1445,

1316, 1291, 1186, 1075, 1026, 960, 883, 794, 762, 737, 699, 649, 547, 435 cm^{-1}; **HRMS (ESI)**: calcd. for $[C_{20}H_{20}Cl_3NO+Na]^+$, $[M+Na]^+$: 418.0503; found: 418.0504; ***R*f**: 0.24 (Hexane).

***cis/trans*-1-(4-(tert-butyl)phenyl)-3-methyl-3-phenylcyclobutyl 2,2,2-trichloroacetimidate (1an).** Following the General Procedure C. 1-(4-(tert-butyl)phenyl)-3-methyl-3-phenylcyclobutan-1-ol (330 mg, 1.02 mmol, 1 equiv.), n-BuLi (1.6 M in hexane, 700 µl, 1.12 mmol, 1.1 equiv.), 2,2,2-trichloroacetonitrile (736 mg, 511 µl, 5.09 mmol, 5.0 equiv.), 2 h at room temperature. The crude mixture was purified by column chromatography on Al_2O_3 Brockman activity grade V neutralized with 10% Et_3N-Hexane and eluted with Hexane (100%) to afford a mixture of two inseparable diastereoisomers as a white solid in 13% yield, 2.25:1 dr (58 mg, 130 µmol).

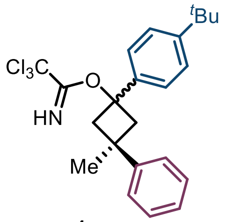

1an

^{1}H NMR (400 MHz, C_6D_6): (mixture of diastereomers, signals are reported as seen) δ 8.15 (s, 0.35H), 7.99 (s, 0.88H), 7.67 (d, *J* = 8.3 Hz, 2H), 7.39 – 7.31 (m, 3H), 7.20 – 7.04 (m, 5H), 6.97 (t, *J* = 7.0 Hz, 3H), 3.24 (d, *J* = 13.8 Hz, 2H), 3.11 (d, *J* = 14.6 Hz, 1H), 3.05 – 2.95 (m, 3H), 1.15 (s, 9H), 1.08 (s, 4H) ppm; **^{13}C NMR (101 MHz, C_6D_6)**: (mixture of diastereomers, signals are reported as seen) δ 158.73, 158.60, 151.38, 150.96, 150.29, 149.66, 140.07, 139.35, 128.38, 127.11, 125.53, 125.51, 125.29, 125.11, 125.05, 124.96, 124.88, 83.02, 81.92, 53.01, 46.44, 46.14, 36.52, 35.77, 34.23, 34.10, 32.58, 31.10, 31.05, 30.90 ppm; **IR (ATR)**: 3336, 2961, 2867, 2361, 1661, 1508, 1458, 1419, 1363, 1317, 1077, 1028, 961, 885, 845, 825, 796, 763, 700, 650, 575, 546 cm^{-1}; **HRMS (ESI)**: calcd. for $[C_{23}H_{26}Cl_3NO+Na]^+$, $[M+Na]^+$: 460.0972; found: 460.0969; ***R*f**: 0.23 (Hexane); **m.p.**: 97–99°C.

***cis/trans*-1-(4-fluorophenyl)-3-methyl-3-phenylcyclobutyl-2,2,4,4-d4 2,2,2-trichloroacetimidate (1ao).** Following the General Procedure C. 1-(4-fluorophenyl)-3-methyl-3-phenylcyclobutan-2,2,4,4-d4-1-ol (200 mg, 0.768 mmol, 1 equiv.), n-BuLi (1.6 M in hexane, 528 µl, 0.845 mmol, 1.1 equiv.), 2,2,2-trichloroacetonitrile (555 mg, 385 µl, 3.84 mmol, 5.0 equiv.), 2 h at room temperature. The crude mixture was purified by column chromatography on Al_2O_3 Brockman activity grade V neutralized with 10% Et_3N-Hexane and eluted with Hexane (100%) to afford a mixture of two inseparable diastereoisomers as a white solid in 58% yield, 1.5:1 dr (180 mg, 445 µmol).

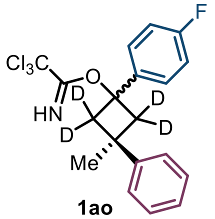

1ao

^{1}H NMR (400 MHz, C_6D_6): (mixture of diastereomers, signals are reported as seen) δ 8.11 (s, 0.57H), 7.97 (s, 0.90H), 7.44 – 7.38 (m, 2H), 7.17 – 7.03 (m, 6H), 7.03 – 6.95 (m, 2H), 6.95 – 6.89 (m, 2H), 6.81 (t, *J* = 8.7 Hz, 2H), 6.65 (t, *J* = 8.7 Hz, 1H), 1.52 (s, 2H), 1.04 (s, 3H) ppm; **^{13}C NMR (101 MHz, C_6D_6)**: (mixture of diastereomers, signals are reported as seen) δ 162.4 (d, *J* = 246.6 Hz), 162.1 (d, *J* = 245.6 Hz), 158.8, 158.6, 151.1, 150.5, 138.3 (d, *J* = 3.1 Hz), 137.7 (d, *J* = 3.2 Hz), 129.6 (d, *J* = 8.2 Hz), 128.4, 128.4, 125.6, 125.0, 124.9, 114.7, 114.7, 114.5, 114.5, 92.1, 91.9, 82.1, 81.0, 38.4, 45.6 (m, $-CD_2$), 36.1, 35.2, 32.3, 30.5 ppm; **^{19}F NMR (376 MHz, C_6D_6)**: (mixture of diastereomers) δ -114.13, -114.95 ppm; **IR (ATR)**: 3334, 2957, 2361, 1660, 1602,

1510, 1445, 1316, 1263, 1232, 1161, 1074, 1027, 1012, 963, 852, 794, 769, 752, 699, 682, 648, 540, 468, 435 cm^{-1}; **HRMS (ESI)**: calcd. for $[C_{19}H_{13}D_4Cl_3FNO+Na]^+$, $[M+Na]^+$: 426.0503; found: 426.0502; ***R*f**: 0.25 (Hexane); **m.p.**: 70–73°C.

Synthesis of 3-methyl-3-phenyl-1-(4-(prop-2-yn-1-yloxy)phenyl)cyclobutan-1-ol (1r)

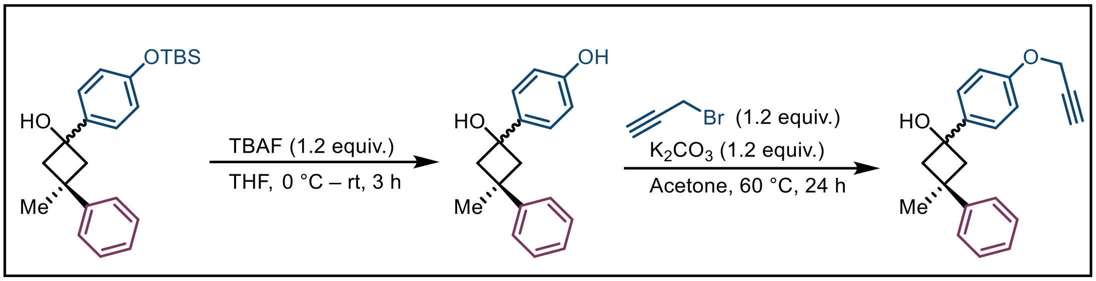

Step I: A flame-dried two-neck flask equipped with a stirring bar was charged with the TBS-alcohol (400 mg, 1.09 mmol, 1.0 equiv.) and anhydrous THF (0.2 M) under an Ar atmosphere. After cooling to 0 °C, TBAF (1M in THF, 1.30 ml, 1.30 mmol, 1.2 equiv.) was added, and the reaction mixture was allowed to warm to r.t. and stir for 3 h. The mixture was quenched with sat aq NH_4Cl and washed with EtOAc three times. The combined organic layers were washed with brine, dried over Na_2SO_4, and the solvent was evaporated. The crude mixture was subjected to the next step without further purification.

Step II: To a solution of the phenol derivative (250 mg, 0.983 mmol, 1.0 equiv.) and propargyl bromide (102 µl, 1.18 mmol, 1.2 equiv.) in acetone, K_2CO_3 (163 mg, 1.18 mmol, 1.2 equiv.) was added, and the mixture was stirred at 60 °C for 24 h. The reaction mixture was cooled to room temperature, filtered to remove the solid, and the volatiles were removed under reduced pressure. The crude mixture was purified by column chromatography (EtOAc-hexane, 5-10%) to afford a mixture of two inseparable diastereoisomers as a white solid in 75% yield, 5.7:1 dr (214 mg, 732 µmol).

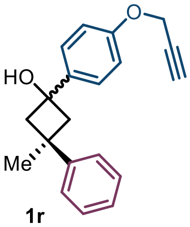

1r

^{1}H NMR (400 MHz, $CDCl_3$): (mixture of diastereomers, signals are reported as seen) δ 7.49 (d, J = 8.7 Hz, 2H), 7.38 – 7.26 (m, 4.7H), 7.23 – 7.11 (m, 1.6H), 7.01 (d, J = 8.7 Hz, 2H), 6.88 (d, J = 8.7 Hz, 0.34H) 4.71 (d, J = 2.4 Hz, 2H), 4.64 (d, J = 2.4 Hz, 0.36H), 3.0 – 2.1 (m, 4.6H), 2.62 – 2.46 (m, 1.4H), 1.94 (s, 0.14H), 1.82 (s, 0.89H), 1.70 (s, 0.56H), 1.25 (s, 3H) ppm; **^{13}C NMR (101 MHz, $CDCl_3$)**: (mixture of diastereomers, signals are reported as seen) δ 156.9, 156.6, 151.8, 140.9, 139.7, 128.4, 128.3, 127.2, 126.1, 125.6, 125.4, 125.3, 125.2, 114.9, 114.7, 78.7, 78.6, 75.7, 72.7, 72.2, 55.9, 55.8, 49.9, 48.6, 36.0, 34.3, 32.8, 31.4 ppm; **IR (ATR):** 3287, 3056, 2968, 2931, 1608, 1583, 1510, 1494, 1444, 1372, 1297, 1217, 1178, 1108, 1025, 895, 831, 763, 736, 700, 643, 549 cm^{-1}; **HRMS (ESI)**: calcd. for $[C_{20}H_{20}O_2+Na]+$, $[M+Na]+$: 315.1356; found: 315.1356; ***R*f**: 0.15 (Hexane/EtOAc, 9:1); **m.p.**: 63–65°C.

Enantioselective elimination of cyclobutanols

Optimization studies

General procedure for optimization studies

An oven-dried 8 ml vial equipped with a magnetic stir bar was charged with substrate (0.06 mmol, 1.0 equiv.). The vial was evacuated and backfilled with argon three times. Then, 0.4 ml of an indicated solvent was added, and the mixture was stirred at the indicated temperature for 20 minutes (the 20 min. stirring was performed only for reactions below room temperature). Next, the catalyst (5 µmol, 0.05 equiv.) was added in 0.2 ml of the solvent. The mixture was stirred at 700 rpm at the indicated temperature for 16-48 hours. After this, the reaction was quenched by adding Et_3N (100 µl), and the solvent was evaporated. The yields were determined by ^{1}H-NMR with trichloroethylene as an internal standard. The residue was purified by column chromatography on silica gel.

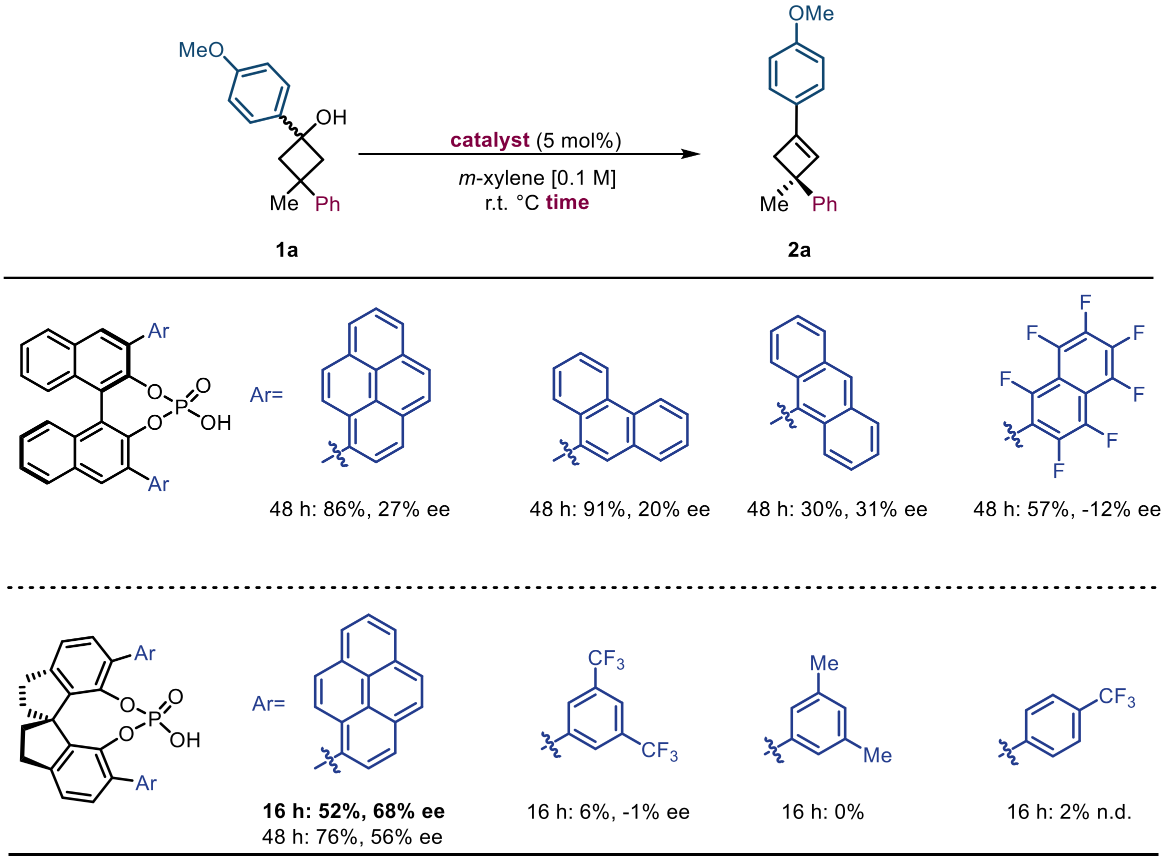

Figure S1. Evaluation of chiral phosphoric acid catalysts in the enantioselective elimination of cyclobutanols.

Table S1. Evaluation of other reaction parameters using catalyst **C4** and **C5**.

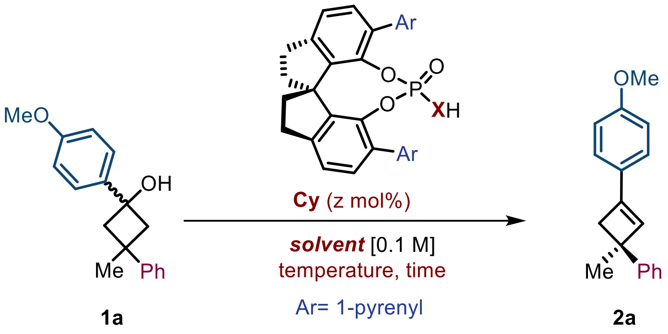

Entry	*X*	*Solvent*	*temperature*	*z*	Time [h]	Yield [%]	ee [%]
1	OH	*m*-xylene	r.t.	5%	16	52	68
2	NHTf	*m*-xylene	r.t.	5%	16	99	0
3	NHTf	*m*-xylene	-10 °C	5%	16	99	65
4	NHTf	*m*-xylene	-10 °C	3%	16	93	63
5	NHTf	*m*-xylene	-20 °C	3%	16	83	83
6	NHTf	*m*-xylene	-30 °C	3%	16	43	87
7	NHTf	*m*-xylene	-30 °C	5%	16	80	89
8	NHTf	mesitylene	-30 °C	5%	16	62	91
9	NHTf	mesitylene	-30 °C	5%	24	84	91
10	NHTf	mesitylene	-35 °C	5%	44	44	92

Table S2. Evaluation of solvents

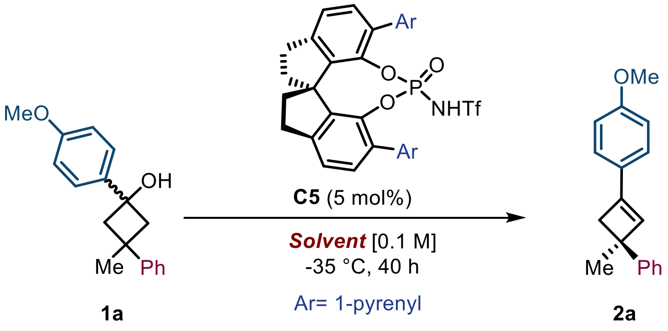

Entry	*solvent*	Yield [%]	ee [%]
1	mesitylene	44	92
2	*m*-xylene	22	91
3	toluene	31	89
4	chlorobenzene	41	69

Table S3. Evaluation of reaction concentration

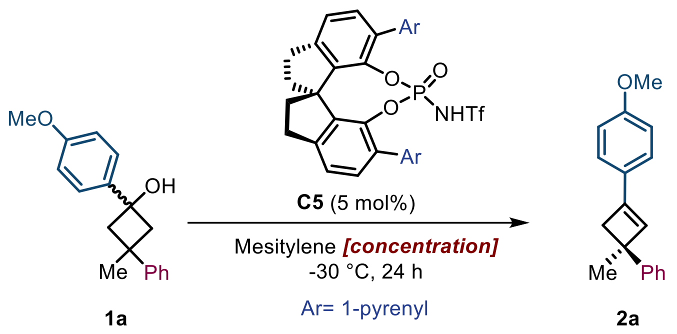

Entry	*concentration*	Yield [%]	ee [%]
1	0.15	72	91
2	0.1	84	91
3	0.075	10	91

Table S4. Further evaluation of catalysts

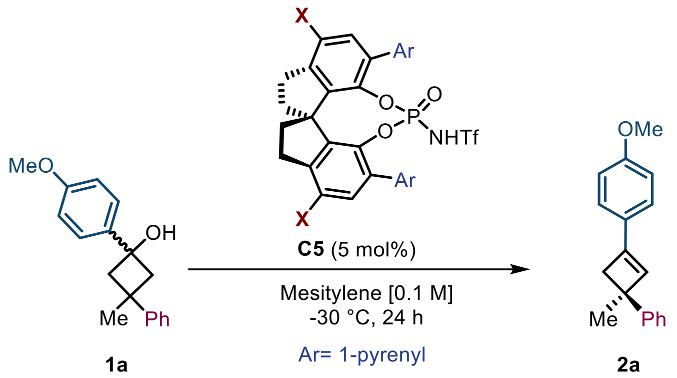

Entry	*X*	Yield [%]	ee [%]
1	H	84	91
2	Br	95	79

Table S5. Effect of desiccant

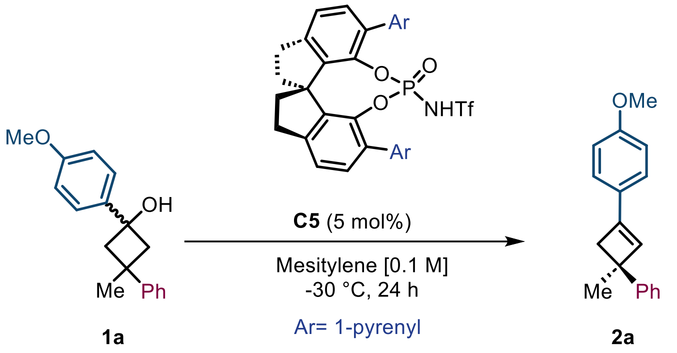

Entry	Desiccant	Yield [%]	ee [%]
1	None	84	91
2	4 Å MS	99	86

The absolute stereochemistry

The absolute configuration of product **2a** was determined to be *S* by comparison of the optical rotation values and HPLC analysis, as reported by Xia and coworkers.[12] Further comparison of the optical rotations of products **2y**, **2z**, **2ac**, and **2ad** shows good agreement with the reported compounds. The stereochemistry of other products from this series was assigned by analogy. The absolute configurations of products **2aa** and **2ab** obtained in the reaction with the catalyst (*S*)-**C4** were determined as *R* by comparison of the optical rotation values and HPLC analysis, as reported by Xia and coworkers.[13]

General procedure D for enantioselective elimination of cyclobutanols

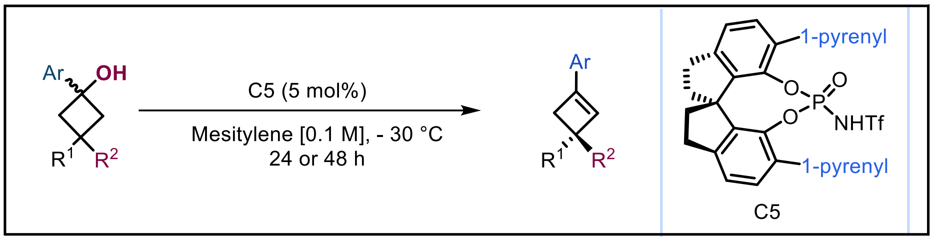

An oven-dried 8 ml vial equipped with a magnetic stir bar was charged with substrate (0.1 mmol, 1.0 equiv.). The vial was evacuated and backfilled with argon three times. Then, 0.7 ml of mesitylene was added, and the mixture was stirred at -30 °C for 20 minutes. Next, the catalyst (5 µmol, 0.05 equiv.) was added in 0.3 mL of mesitylene at -30 °C. The mixture was stirred at 700 rpm at -30 °C for 24-48 hours. After this, the reaction was quenched by adding Et_3N (100 µl), and the mixture was purified by flash column chromatography.

General procedure E for enantioselective elimination of cyclobutyl trichloroacetimidates

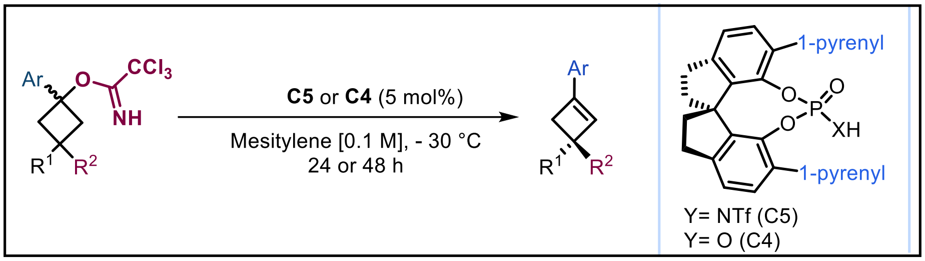

An oven-dried 8 ml vial equipped with a magnetic stir bar was charged with substrate (0.1 mmol, 1.0 equiv.). The vial was evacuated and backfilled with argon three times. Then, 0.7 ml of mesitylene was added, and the mixture was stirred at -30 °C for 20 minutes. Next, the catalyst (5 µmol, 0.05 equiv.) was added in 0.3 mL of mesitylene at -30 °C. The mixture was stirred at 700 rpm at -30 °C for 24-48 hours. After this, the reaction was quenched by adding Et_3N (100 µl), and the mixture was purified by column chromatography on Al_2O_3 Brockman activity grade V using 100% hexane.

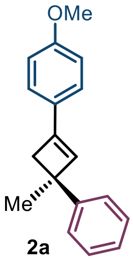

2a

(*S*)-1-methoxy-4-(3-methyl-3-phenylcyclobut-1-en-1-yl)benzene (2a). Following the General Procedure D. 1-(4-methoxyphenyl)-3-methyl-3-phenylcyclobutan-1-ol **1a** (27.0 mg, 0.100 mmol, 1 equiv., *cis/trans* 2:1), cat. **C5** (4.2 mg, 5 µmol, 0.05 equiv.), mesitylene (1 ml), reaction time 24 h. The crude mixture was purified by column chromatography (EtOAc-hexane, 1-6%) to afford the product as a white solid in 83% yield, with a 90% ee (21.0 mg, 0.084 mmol).

^{1}H NMR (400 MHz, $CDCl_3$): δ 7.39 (d, *J* = 7.1 Hz, 2H), 7.35 – 7.27 (m, 4H), 7.18 (t, *J* = 7.3 Hz, 1H), 6.86 (d, *J* = 8.6 Hz, 2H), 6.58 (s, 1H), 3.81 (s, 3H), 2.91 (q, *J* = 12.5 Hz, 2H), 1.62 (s, 3H) ppm; **^{13}C NMR (101 MHz, $CDCl_3$)**: δ 159.5, 148.1, 143.2, 131.3, 128.2, 128.0, 126.1, 126.0, 125.7, 113.8, 55.4, 45.9, 44.4, 27.8; ***R_f***: 0.55 (Hexane/EtOAc, 95:5); $[\alpha]_D^{20}$: +71.9 (c = 1.00, $CHCl_3$); **HPLC analysis**: Chiralpak AD-H, 4.6 x 250 mm; 0.8:99.2 i-PrOH/hexane, 1.0 mL/min, λ = 254 nm; τmajor = 7.0 min, τminor = 8.9 min, 90% ee.

Spectral data match the ones reported in the literature.[13]

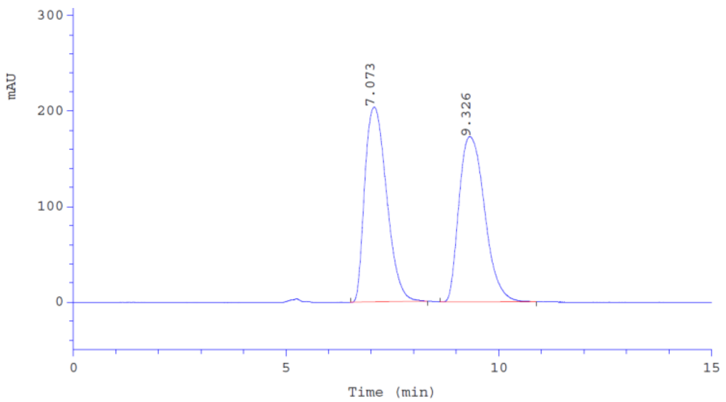

	Ret.time [min]	Start [min]	End [min]	Amount	Area [mAU*min]	Height [mAU]	% Area
1	7.073	6.51	8.32	-1	121.326	204.092	50.0704
2	9.326	8.62	10.88	-1	120.985	173.073	49.9296

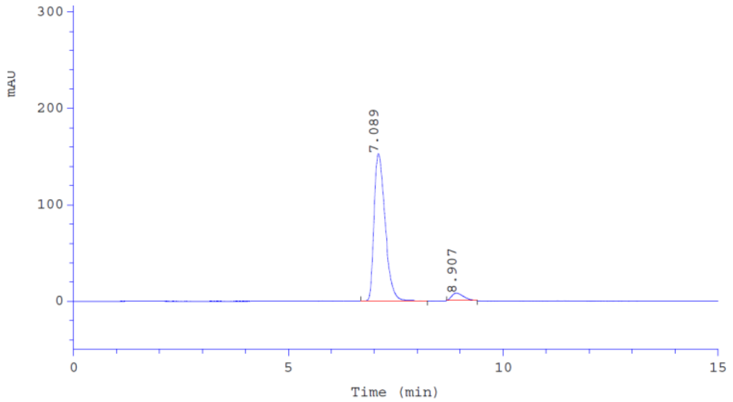

	Ret.time [min]	Start [min]	End [min]	Amount	Area [mAU*min]	Height [mAU]	% Area
1	7.089	6.69	8.24	-1	45.4054	152.633	94.8690
2	8.907	8.68	9.40	-1	2.45575	7.59683	5.1310

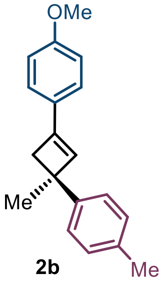

2b

(*S*)-1-methoxy-4-(3-methyl-3-(*p*-tolyl)cyclobut-1-en-1-yl)benzene (2b). Following the General Procedure D. 1-(4-methoxyphenyl)-3-methyl-3-(*p*-tolyl)cyclobutan-1-ol **1b** (28.2 mg, 0.100 mmol, 1 equiv. *cis/trans* 1.8:1), cat. **C5** (4.2 mg, 5 µmol, 0.05 equiv.), mesitylene (1 ml), reaction time 48 h. The crude mixture was purified by column chromatography (EtOAc-hexane, 1-6%) to afford the product as a white solid in 99% yield, with a 92% ee (26.2 mg, 0.099 mmol).

Reaction carried out for 24 h: 87% yield, 90% ee.

^{1}H NMR (400 MHz, $CDCl_3$): δ 7.30 (dd, J = 13.0, 8.4 Hz, 4H), 7.12 (d, J = 7.9 Hz, 2H), 6.86 (d, J = 8.7 Hz, 2H), 6.55 (s, 1H), 3.81 (s, 3H), 2.94 – 2.82 (m, 2H), 2.32 (s, 3H), 1.60 (s, 3H) ppm; **^{13}C NMR (101 MHz, $CDCl_3$)**: δ 159.4, 145.1, 143.3, 135.2, 131.5, 128.9, 128.0, 126.0, 125.9, 113.8, 55.4, 45.5, 44.5, 27.8, 21.1 ppm; **IR (ATR)**: 3032, 2999, 2961, 2914, 2862, 1619, 1602, 1508, 1466, 1318, 1251, 1175, 1107, 1031, 838, 817, 805, 783, 534, 494 cm^{-1}; **HRMS (ESI)**: calcd. for $[C_{19}H_{20}O+Na]^+$, $[M+Na]^+$: 287.1406; found: 287.1404; ***R*f**: 0.53 (Hexane/EtOAc, 9:1);

m.p.: 124–125°C; $[\alpha]_D^{20}$: +52.1 (c = 1.00, $CHCl_3$); HPLC analysis: Chiralpak IA, 4.6 x 250 mm; 0.5:99.5 i-PrOH/hexane, 1.0 mL/min, λ = 254 nm; τmajor = 7.6 min, τminor = 9.0 min, 92% ee.

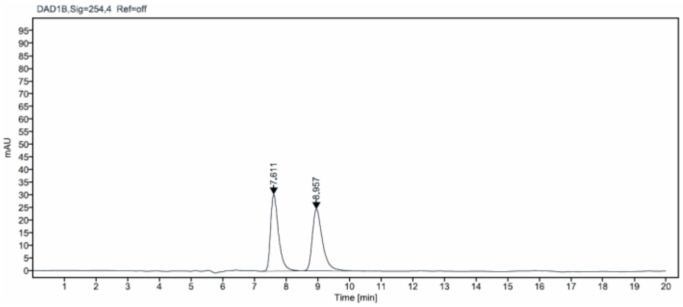

Signal: DAD1B,Sig=254,4 Ref=off

RT [min]	Width [min]	Area	Height	Area%
7.611	1.2300	530.1025	30.3486	49.2160
8.957	2.0133	546.9904	24.4554	50.7840

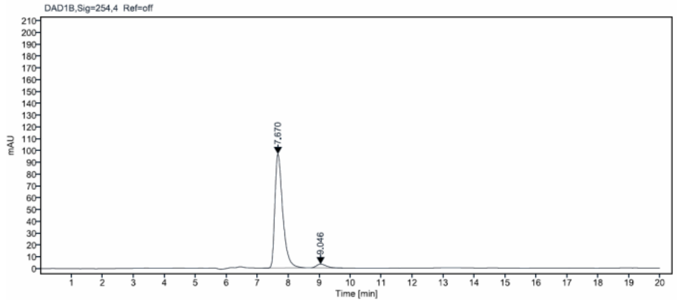

Signal: DAD1B,Sig=254,4 Ref=off

RT [min]	Width [min]	Area	Height	Area%
7.670	1.4776	1705.5835	96.6217	95.9135
9.046	1.0891	72.6687	3.2922	4.0865

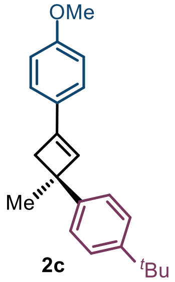

2c

(*S*)-1-(*tert*-butyl)-4-(3-(4-methoxyphenyl)-1-methylcyclobut-2-en-1-yl)benzene (**2c**). Following the General Procedure D. 3-(4-(*tert*-butyl)phenyl)-1-(4-methoxyphenyl)-3-methylcyclobutan-1-ol **1c** (32.4 mg, 0.100 mmol, 1 equiv., *cis/trans* 10:1), cat. **C5** (4.2 mg, 5 µmol, 0.05 equiv.), mesitylene (1 ml), reaction time 48 h. The crude mixture was purified by column chromatography (EtOAc-hexane, 1-6%) to afford the product as a white solid in 99% yield, with a 89% ee (30.4 mg, 0.099 mmol). **^{1}H NMR (400 MHz, C_6D_6)**: δ 7.33 – 7.26 (m, 4H), 7.22 (d, *J* = 8.6 Hz, 2H), 6.73 (d, *J* = 8.7 Hz, 2H), 6.45 (s, 1H), 3.24 (s, 3H), 2.89 (d, *J* = 12.4 Hz, 1H), 2.74 (d, *J* = 12.4 Hz, 1H), 1.56 (s, 3H), 1.23 (s, 9H) ppm; **^{13}C NMR (101 MHz, C_6D_6)**: δ 159.7, 148.1, 145.0, 143.4, 131.3, 126.2, 125.8, 125.0, 113.8, 54.5, 45.4, 44.3, 34.1, 31.3, 27.8 ppm; **IR (ATR)**: 3031, 2956, 2913, 2860, 1620, 1602, 1507, 1427, 1359, 1301, 1249, 1173, 1120, 1030, 1017, 831, 800, 791, 589, 569, 544, 459 cm^{-1} **HRMS (ESI)**: calcd. for $[C_{22}H_{26}O+Na]^+$, $[M+Na]^+$: 329.1876; found: 329.1877; ***R*f**: 0.51 (Hexane/EtOAc, 9:1); **m.p.**: 146–148°C; $[\alpha]_D^{20}$: +35.1 (c =

1.00, $CHCl_3$); HPLC analysis: Chiralpak IC, 4.6 x 250 mm; 0.5:99.5 i-PrOH/hexane, 1.0 mL/min, λ = 254 nm; τmajor = 5.4 min, τminor = 6.3 min, 89% ee.

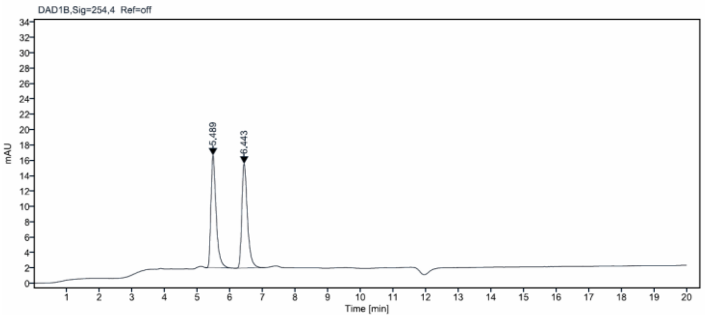

Signal:	DAD1B,Sig=254,4 Ref=off			
RT [min]	Width [min]	Area	Height	Area%
5.489	0.8312	161.4653	14.6047	49.8904
6.443	0.9302	162.1749	13.5684	50.1096

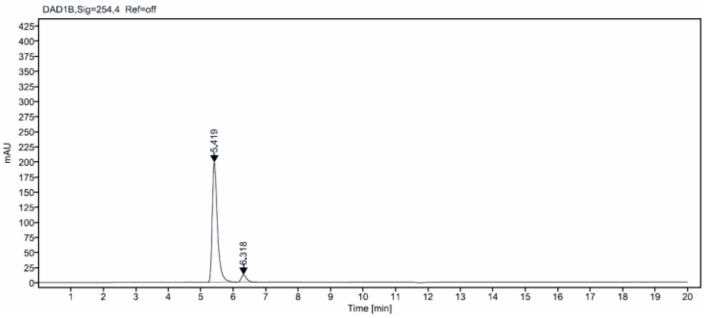

Signal:	DAD1B,Sig=254,4 Ref=off			
RT [min]	Width [min]	Area	Height	Area%
5.419	0.8950	2205.5701	198.2288	94.3252
6.318	0.5621	132.6906	11.7284	5.6748

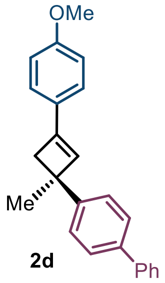

2d

(*S*)-4-(3-(4-methoxyphenyl)-1-methylcyclobut-2-en-1-yl)-1,1'-biphenyl (2d). Following the General Procedure D. 3-([1,1'-biphenyl]-4-yl)-1-(4-methoxyphenyl)-3-methylcyclobutan-1-ol **1d** (34.4 mg, 0.100 mmol, 1 equiv., *cis/trans* 1.1:1), cat. **C5** (4.2 mg, 5 μmol, 0.05 equiv.), mesitylene (1 ml), reaction time 48 h. The crude mixture was purified by column chromatography (EtOAc-hexane, 1-6%) to afford the product as a white solid in 64% yield, with a 85% ee (21.0 mg, 0.064 mmol).

^{1}H NMR (400 MHz, $CDCl_3$): δ 7.61 – 7.50 (m, 4H), 7.48 – 7.38 (m, 4H), 7.36 – 7.28 (m, 3H), 6.86 (d, *J* = 8.8 Hz, 2H), 6.59 (s, 1H), 3.81 (s, 3H), 2.93 (q, *J* = 12.5 Hz, 2H), 1.65 (s, 3H) ppm; **^{13}C NMR (101 MHz, $CDCl_3$)**: δ 159.5, 147.2, 143.5, 141.2, 138.7, 131.3, 128.8, 127.9, 127.2, 127.1, 127.0, 126.4, 126.1, 113.8, 55.4, 45.6, 44.5, 27.7 ppm; **IR (ATR)**: 3029, 2954, 2915, 2858, 2834, 1622, 1601, 1507, 1486, 1455, 1396, 1301, 1249, 1173, 1107, 1092, 1036, 1007, 836, 801, 766, 731, 697, 613, 573, 544 cm^{-1}; **HRMS (ESI)**: calcd. for $[C_{24}H_{22}O+Na]^+$, $[M+Na]^+$:

349.1563; found: 349.1560; ***R*f**: 0.45 (Hexane/EtOAc, 9:1); **m.p.**: 127–130°C; $[\alpha]_D^{20}$: +6.5 (c = 1.00, $CHCl_3$); **HPLC analysis**: Chiralpak IF, 4.6 x 250 mm; 0.5:99.5 i-PrOH/hexane, 1.0 mL/min, λ = 254 nm; τmajor= 25.3 min, τminor= 21.5 min, 85% ee.

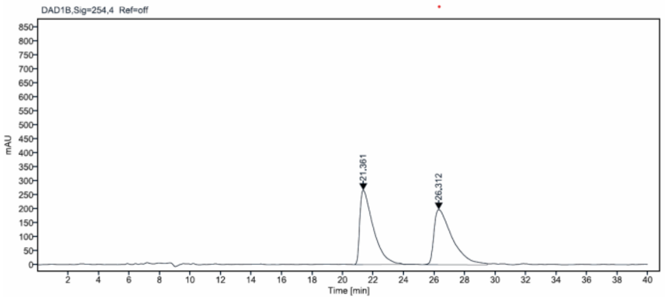

Signal: DAD1B,Sig=254,4 Ref=off

RT [min]	Width [min]	Area	Height	Area%
21.361	3.9938	16234.7254	265.9873	49.7296
26.312	4.2541	16411.2910	197.4485	50.2704

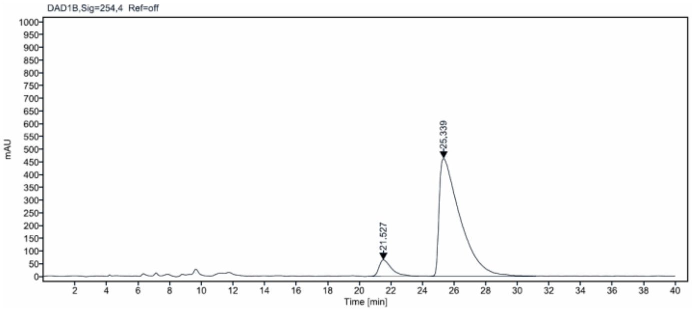

Signal: DAD1B,Sig=254,4 Ref=off

RT [min]	Width [min]	Area	Height	Area%
21.527	3.2111	3508.4945	63.8617	7.5909
25.339	6.7217	42711.0181	462.4545	92.4091

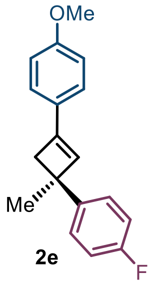

2e

(*S*)-1-fluoro-4-(3-(4-methoxyphenyl)-1-methylcyclobut-2-en-1-yl)benzene (2e). Following the General Procedure D. 3-(4-fluorophenyl)-1-(4-methoxyphenyl)-3-methylcyclobutan-1-ol **1e** (28.6 mg, 0.100 mmol, 1 equiv., *cis/trans* 1.2:1), cat. **C5** (4.2 mg, 5 µmol, 0.05 equiv.), mesitylene (1 ml), reaction time 48 h. The crude mixture was purified by column chromatography (EtOAc-hexane, 1-6%) to afford the product as a white solid in 99% yield, with a 90% ee (26.6 mg, 0.099 mmol).

Reaction carried out for 24 h: 66% yield, 86% ee.

^{1}H NMR (400 MHz, $CDCl_3$): δ 7.37 – 7.29 (m, 4H), 6.97 (t, *J* = 8.8 Hz, 2H), 6.86 (d, *J* = 8.7 Hz, 2H), 6.52 (s, 1H), 3.81 (s, 3H), 2.87 (s, 2H), 1.59 (s, 3H) ppm; **^{13}C NMR (101 MHz, $CDCl_3$):** δ161.1 (d, *J* = 243.4 Hz), 159.5, 143.8 (d, *J* = 3.3 Hz), 143.5, 131.1, 127.4 (d, *J* = 7.8 Hz), 127.4, 126.1, 114.8 (d, *J* = 21.1 Hz)., 113.8, 55.4, 45.3, 44.5, 27.8 ppm; **^{19}F NMR (376 MHz, $CDCl_3$):** δ

-118.03 ppm; **IR (ATR)**: 2966, 2964, 2944, 2864, 1618, 1600, 1573, 1508, 1465, 1318, 1251, 1221, 1175, 1160, 1098, 1087, 1030, 832, 812, 79, 651, 548 cm^{-1}; **HRMS (ESI)**: calcd. for $[C_{18}H_{17}FO+H]^+$, $[M+H]^+$: 269.1336; found: 269.1335; ***R*f**: 0.40 (Hexane/EtOAc, 95:5); **m.p.**: 112–115°C; $[\alpha]_D^{20}$: +76.7 (c = 1.00, $CHCl_3$); **HPLC analysis**: Chiralpak IA, 4.6 x 250 mm; 0.5:99.5 i-PrOH/hexane, 1.0 mL/min, λ = 254 nm; τmajor = 8.2 min, τminor = 9.2 min, 90% ee.

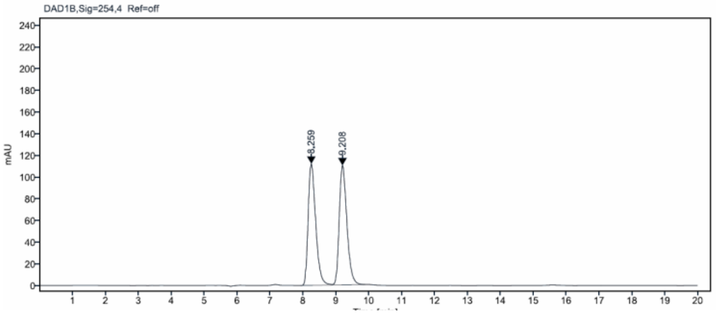

Signal: DAD1B,Sig=254,4 Ref=off

RT [min]	Width [min]	Area	Height	Area%
8.259	0.9547	1778.7438	111.9601	50.0617
9.208	0.9953	1774.3622	110.0818	49.9383

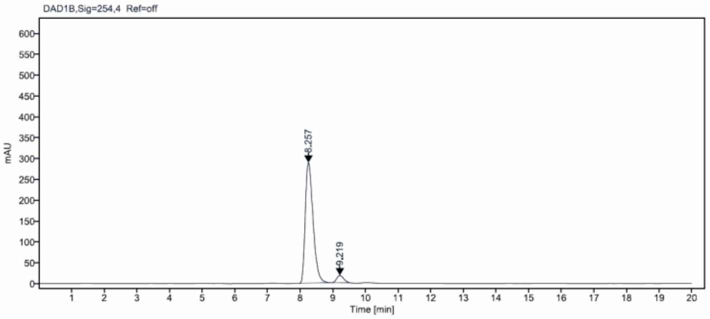

Signal: DAD1B,Sig=254,4 Ref=off

RT [min]	Width [min]	Area	Height	Area%
8.257	1.0746	4796.6453	289.0770	94.8318
9.219	0.5561	261.4091	17.5011	5.1682

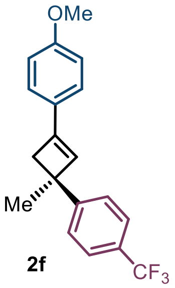

2f

(*S*)-1-methoxy-4-(3-methyl-3-(4-(trifluoromethyl)phenyl)cyclobut-1-en-1-yl)benzene (2f). Following the General Procedure D. 1-(4-methoxyphenyl)-3-methyl-3-(4-(trifluoromethyl)phenyl)cyclobutan-1-ol **1f** (33.6 mg, 0.100 mmol, 1 equiv., *cis/trans* 1.1:1), cat. **C5** (4.2 mg, 5 µmol, 0.05 equiv.), mesitylene (1 ml), reaction time 48 h. The crude mixture was purified by column chromatography (EtOAc-hexane, 1-6%) to afford the product as a white solid in 60% yield, with a 88% ee (19.0 mg, 0.060 mmol).

^{1}H NMR (400 MHz, $CDCl_3$): δ 7.55 (d, J = 8.2 Hz, 2H), 7.48 (d, J = 8.2 Hz, 2H), 7.32 (d, J = 8.7 Hz, 2H), 6.87 (d, J = 8.8 Hz, 2H), 6.54 (s, 1H), 3.81 (s, 3H), 2.91 (s, 2H), 1.62 (s, 3H) ppm; **^{13}C NMR (101 MHz, $CDCl_3$)**: δ 159.7, 152.1, 143.8, 130.3, 128.0 (d, J = 32.5 Hz), 127.5, 126.3, 126.1, 125.1 (q, J = 3.8 Hz), 123.1, 113.9, 55.4, 45.8, 44.4, 27.5 ppm; **^{19}F NMR (376 MHz,**

$CDCl_3$): δ -62.13 ppm; **IR (ATR)**: 2960, 2915, 2835, 1618, 1602, 1508, 1453, 1331, 1252, 1167, 1138, 1106, 1093, 1066, 1033, 1017, 839, 805, 696, 605 cm^{-1}; **HRMS (ESI)**: calcd. for $[C_{19}H_{17}F_3O+H]^+$, $[M+H]^+$: 319.1304; found: 319.1306; ***R*f**: 0.35 (Hexane/EtOAc, 95:5); **m.p.**: 117–120°C; $[\alpha]_D^{20}$: +40.8 (c = 1.00, $CHCl_3$); HPLC analysis: Chiralpak IF, 4.6 x 250 mm; 0.25:99.75 i-PrOH/hexane, 1.0 mL/min, λ = 254 nm; τmajor = 10.6 min, τminor = 9.3 min, 88% ee.

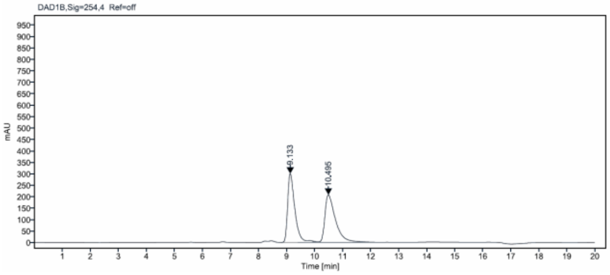

Signal: DAD1B,Sig=254,4 Ref=off

RT [min]	Width [min]	Area	Height	Area%
9.133	1.1437	5341.4342	300.2045	49.5756
10.495	2.4263	5432.8824	206.9265	50.4244

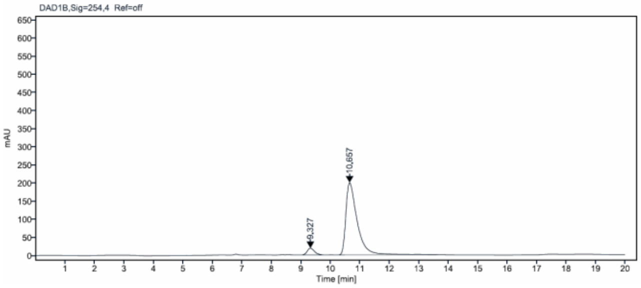

Signal: DAD1B,Sig=254,4 Ref=off

RT [min]	Width [min]	Area	Height	Area%
9.327	0.9519	327.6951	18.8429	5.7910
10.657	3.3940	5330.9729	199.4410	94.2090

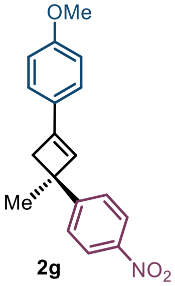

2g

(*S*)-1-Methoxy-4-(3-methyl-3-(4-nitrophenyl)cyclobut-1-en-1-yl)benzene (2g). Following the General Procedure D. 1-(4-methoxyphenyl)-3-methyl-3-(4-nitrophenyl)cyclobutan-1-ol **1g** (31.3 mg, 0.100 mmol, 1 equiv., *trans/cis* 1.8:1), cat. **C5** (16.9 mg, 20 μmol, 0.2 equiv.), mesitylene (1 ml), reaction time 48 h. The crude mixture was purified by column chromatography (EtOAc-hexane 1-5%) to afford the product as a slight yellow solid in 58% yield, 73% ee (17.0 mg, 0.058 mmol).

^{1}H NMR (400 MHz, C_6D_6): δ 7.92 (d, J = 8.7 Hz, 2H), 7.24 (d, J = 8.7 Hz, 2H), 6.93 (d, J = 8.8 Hz, 2H), 6.81 (d, J = 8.7 Hz, 2H), 6.18 (s, 1H), 3.30 (s, 3H), 2.64 (s, 2H), 1.34 (s, 3H) ppm; **^{13}C NMR (101 MHz, C_6D_6)**: δ 160.4, 155.2, 146.6, 144.4, 129.8, 127.6, 126.8, 126.5, 123.5, 114.2, 54.9, 45.8, 44.3, 27.2 ppm; **IR (ATR)**: 2920, 2850, 1602, 1508, 1463, 1346, 1275, 1260, 1172,

1088, 1035, 836, 801, 764, 750, 700, 420, 407 cm^{-1}; **HRMS (ESI)**: calcd. for $[C_{18}H_{17}NO_3+Na]^+$, $[M+Na]^+$: 318.1106; found: 318.1099; ***R*f**: 0.23 (Hexane/EtOAc, 95:5); **m.p.**: 130–132°C; $[\boldsymbol{\alpha}]_{\mathbf{D}}^{\mathbf{20}}$: +28.6 (c = 1.00, DCM); HPLC analysis: Chiralpak IA, 4.6 x 250 mm; 2.0:98.0 i-PrOH/hexane, 1.0 mL/min, λ = 254 nm; τmajor= 20.0 min, τminor= 25.0 min, 73% ee.

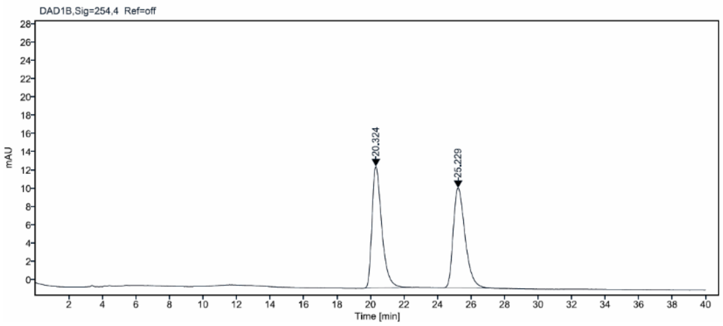

Signal: DAD1B,Sig=254,4 Ref=off

RT [min]	Width [min]	Area	Height	Area%
20.324	2.7667	539.7417	13.3031	50.0746
25.229	3.7928	538.1335	10.9462	49.9254

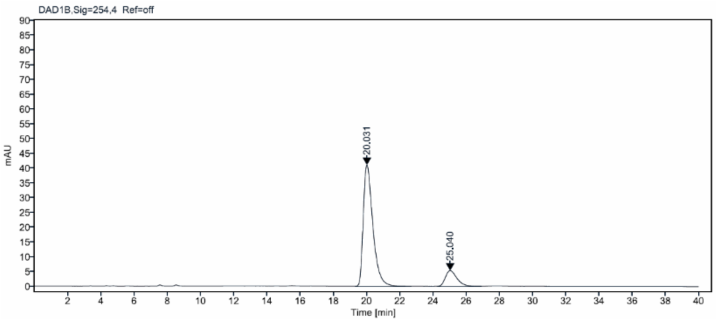

Signal: DAD1B,Sig=254,4 Ref=off

RT [min]	Width [min]	Area	Height	Area%
20.031	3.3722	1653.1439	41.0806	86.5171
25.040	2.6933	257.6263	5.3200	13.4829

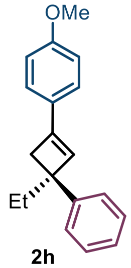

2h

(*S*)-1-(3-Ethyl-3-phenylcyclobut-1-en-1-yl)-4-methoxybenzene (2h). Following the General Procedure D. 3-ethyl-1-(4-methoxyphenyl)-3-phenylcyclobutan-1-ol **1h** (28.2 mg, 0.100 mmol, 1 equiv., *cis/trans* 1.3:1), cat. **C5** (4.2 mg, 5 µmol, 0.05 equiv.), mesitylene (1 ml), reaction time 24 h. The crude mixture was purified by column chromatography (EtOAc-hexane 1-5%) to afford the product as a white solid in 70% yield, 76% ee (18.6 mg, 0.070 mmol).

^{1}H NMR (400 MHz, C_6D_6): δ 7.33 – 7.19 (m, 6H), 7.15 – 7.09 (m, 1H), 6.83 – 6.76 (m, 2H), 6.58 (s, 1H), 3.30 (s, 3H), 2.85 (d, J = 12.5 Hz, 1H), 2.78 (d, J = 12.5 Hz, 1H), 2.03 – 1.91 (m, 1H), 1.86 – 1.71 (m, 1H), 0.84 (t, J = 7.4 Hz, 3H) ppm; **^{13}C NMR (101 MHz, C_6D_6)**: δ 160.1, 146.7, 144.5, 129.1, 128.3 (x2), 127.2, 126.5, 125.8, 114.1, 54.8, 50.7, 42.7, 34.8, 10.5 ppm; **IR (ATR):**

2915, 2849, 1622, 1602, 1506, 1444, 1419, 1376, 1300, 1269, 1246, 1171, 1106, 1030, 872, 835, 794, 756, 751, 734, 699, 615, 563, 535, 457 cm^{-1}; **HRMS (ESI)**: calcd. for $[C_{19}H_{20}O+Na]^+$, $[M+Na]^+$: 287.1412; found: 287.1408; ***R*f**: 0.32 (Hexane/EtOAc, 95:5); **m.p.**: 59–60°C; $[\alpha]_D^{20}$: +87.3 (c = 0.65, DCM); HPLC analysis: Chiralpak IA, 4.6 x 250 mm; 0.5:99.5 i-PrOH/hexane, 1.0 mL/min, λ = 254 nm; τmajor= 7.5 min, τminor= 10.1 min, 76% ee.

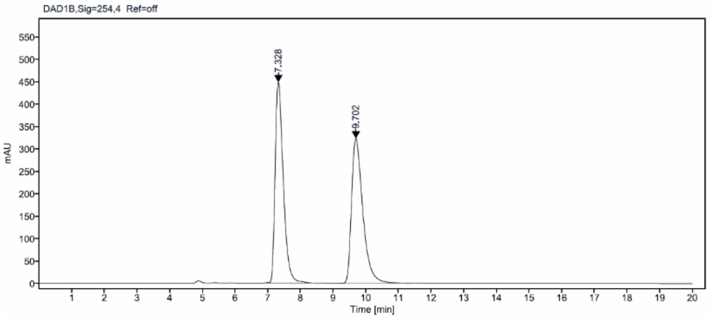

Signal: DAD1B,Sig=254,4 Ref=off

RT [min]	Width [min]	Area	Height	Area%
7.328	1.6088	7800.6508	447.8955	50.1615
9.702	2.1783	7750.4193	323.0164	49.8385

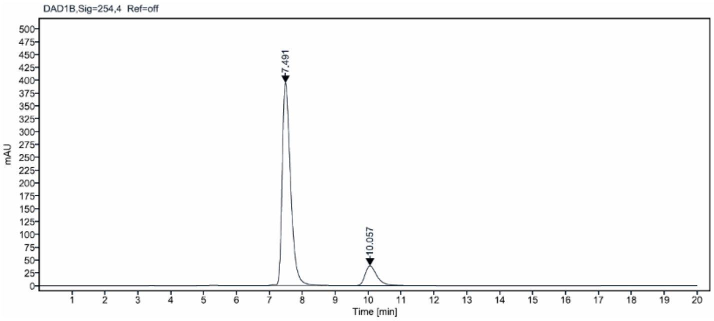

Signal: DAD1B,Sig=254,4 Ref=off

RT [min]	Width [min]	Area	Height	Area%
7.491	1.8223	6953.4022	394.4333	87.8962
10.057	1.6114	957.5192	38.4363	12.1038

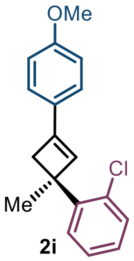

2i

(*S*)-1-chloro-2-(3-(4-methoxyphenyl)-1-methylcyclobut-2-en-1-yl)benzene (2i). Following the General Procedure D. *trans*-3-(2-chlorophenyl)-1-(4-methoxyphenyl)-3-methylcyclobutan-1-ol **1i-*trans*** (30.3 mg, 0.100 mmol, 1 equiv.), cat. **C5** (4.2 mg, 5 µmol, 0.05 equiv.), mesitylene (1 ml), reaction time 48 h. The crude mixture was purified by column chromatography (EtOAc-hexane 1-6%) to afford the product as a white solid in 90% yield, 90% ee (25.6 mg, 0.090 mmol).

^{1}H NMR (400 MHz, C_6D_6): δ 7.21 – 7.15 (m, 3H), 7.13 – 7.10 (m, 1H), 6.87 (t, J = 7.5 Hz, 1H), 6.78 – 6.68 (m, 3H), 6.52 (s, 1H), 3.22 (d, J = 0.8 Hz, 3H), 3.12 (d, J = 13.0 Hz, 1H), 2.90 (d, J = 13.0 Hz, 1H), 1.62 (s, 3H) ppm; **^{13}C NMR (101 MHz, C_6D_6)**: δ 159.9, 144.9, 143.6, 133.3, 130.4, 129.4, 128.7, 127.3, 126.4, 126.4, 113.8, 54.5, 46.6, 43.3, 26.1 ppm; **IR (ATR):** 3062, 3032, 2954,

2916, 2862, 2834, 1624, 1604, 1575, 1507, 1471, 1439, 1426, 1366, 1318, 1301, 1246, 1172, 1145, 1098, 1036, 1018, 945, 904, 859, 837, 803, 754, 731, 723, 662, 615, 564, 540, 512, 456 cm^{-1}; **HRMS (ESI)**: calcd. for $[C_{18}H_{17}ClO+H]^+$, $[M+H]^+$: 285.1041; found: 285.1042; ***R*f**: 0.39 (Hexane/EtOAc, 95:5); **m.p.**: 87–88°C; $[\alpha]_D^{20}$: +204.9 (c = 1.00, CH_2Cl_2) HPLC analysis: Chiralpak IA, 4.6 x 250 mm; 0.5:99.5 i-PrOH/hexane, 1.0 mL/min, λ = 254 nm; τmajor= 7.4 min, τminor= 8.9 min, 90% ee.

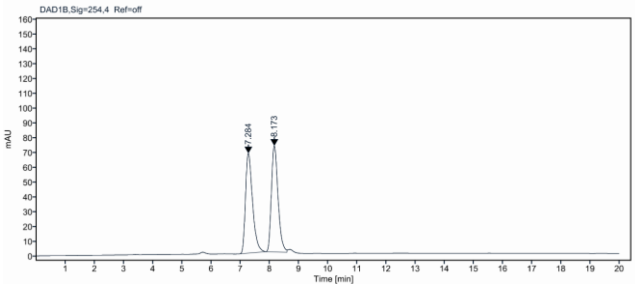

Signal: DAD1B,Sig=254,4 Ref=off

RT [min]	Width [min]	Area	Height	Area%
7.284	0.9229	1128.5201	67.3442	49.8518
8.173	0.7255	1135.2289	71.6883	50.1482

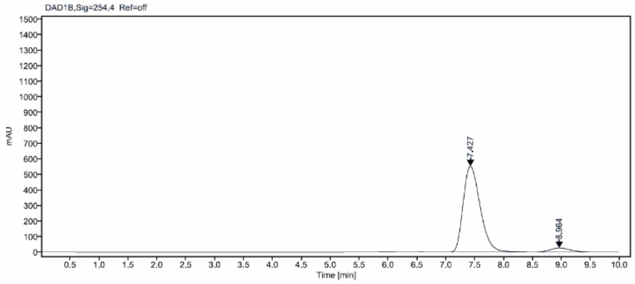

Signal: DAD1B,Sig=254,4 Ref=off

RT [min]	Width [min]	Area	Height	Area%
7.427	1.5098	11258.2736	552.7877	94.8194
8.964	1.1204	615.1146	25.7316	5.1806

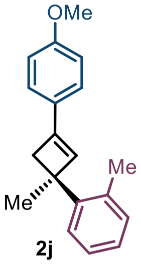

2j

(*S*)-1-(3-(4-Methoxyphenyl)-1-methylcyclobut-2-en-1-yl)-2-methylbenzene (2j). Following the General Procedure D. *trans*-1-(4-methoxyphenyl)-3-methyl-3-(o-tolyl)cyclobutan-1-ol **1j-*trans*** (28.2 mg, 0.100 mmol, 1 equiv.), cat. **C5** (4.2 mg, 5 µmol, 0.05 equiv.), mesitylene (1 ml), reaction time 48 h. The crude mixture was purified by column chromatography (EtOAc-hexane 1-3%) to afford the product as a white solid in 87% yield, 92% ee (23.1 mg, 0.087 mmol).

^{1}H NMR (600 MHz, C_6D_6): δ 7.30 – 7.25 (m, 3H), 7.14 – 7.07 (m, 3H), 6.80 (d, J = 8.7 Hz, 2H), 6.64 (s, 1H), 3.30 (s, 3H), 2.95 (d, J = 12.2 Hz, 1H), 2.77 (d, J = 12.2 Hz, 1H), 2.27 (s, 3H), 1.54 (s, 3H) ppm; **^{13}C NMR (151 MHz, C_6D_6)**: δ 160.0, 145.8, 142.7, 135.5, 131.6, 131.1, 128.4, 127.5,

126.5, 126.4, 126.0, 114.1, 54.8, 46.9, 43.5, 27.5, 20.4 ppm; **IR (ATR)**: 2954, 2834, 1625, 1603, 1575, 1508, 1488, 1420, 1318, 1247, 1172, 1133, 1105, 1078, 1036, 943, 862, 838, 810, 795, 782, 759, 728, 675, 617, 538, 453, 420 cm^{-1}; **HRMS (ESI)**: calcd. for $[C_{19}H_{20}O+Na]^+$, $[M+Na]^+$: 287.1412; found: 287.1409; ***R*f**: 0.38 (Hexane/EtOAc, 98:2); **m.p.**: 92–93°C; $[\alpha]_D^{20}$: +182.1 (c = 1.00, $CHCl_3$); HPLC analysis: Chiralpak IC, 4.6 x 250 mm; 0.5:99.5 i-PrOH/hexane, 1.0 mL/min, λ = 254 nm; τmajor= 5.5 min, τminor= 6.2 min, 92% ee.

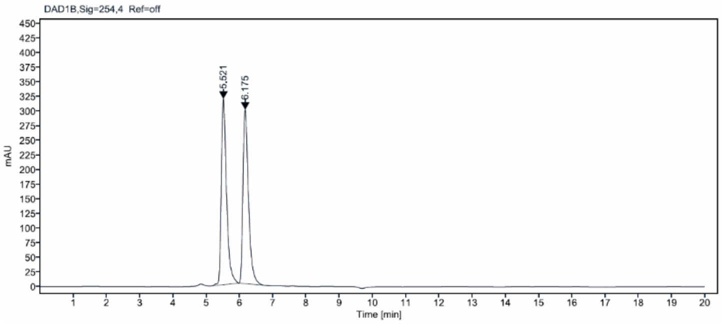

Signal: DAD1B,Sig=254,4 Ref=off

RT [min]	Width [min]	Area	Height	Area%
5.521	0.7869	3492.3689	317.4315	50.4259
6.175	1.0156	3433.3744	297.1227	49.5741

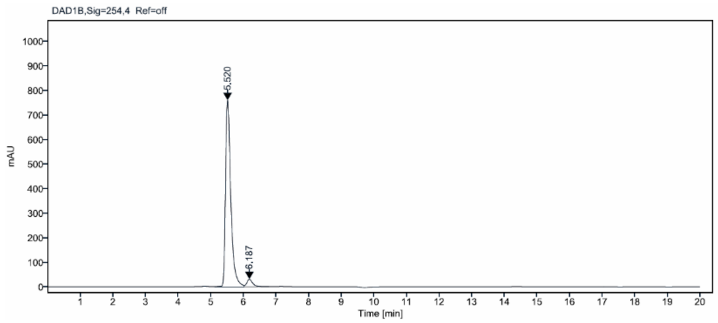

Signal: DAD1B,Sig=254,4 Ref=off

RT [min]	Width [min]	Area	Height	Area%
5.520	0.9094	8500.2630	759.3761	95.7635
6.187	0.7529	376.0436	30.5148	4.2365

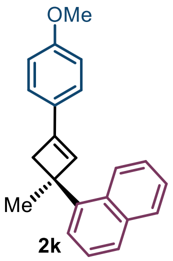

2k

(*S*)-1-(3-(4-methoxyphenyl)-1-methylcyclobut-2-en-1-yl)naphthalene (2k). Following the General Procedure D. *cis*-1-(4-methoxyphenyl)-3-methyl-3-(naphthalen-1-yl)cyclobutan-1-ol **1k-*cis*** (31.8 mg, 0.100 mmol, 1 equiv.), cat. **C5** (4.2 mg, 5 μmol, 0.05 equiv.), mesitylene (1 ml), reaction time 48 h. The crude mixture was purified by column chromatography (EtOAc-hexane, 1-6%) to afford the product as a white solid in 87% yield, with an 86% ee (26.0 mg, 0.087 mmol).

Reaction carried out for 24 h: 60% yield, 84% ee.

^{1}H NMR (400 MHz, CDCl$_3$): δ 8.13 (d, *J* = 9.0 Hz, 1H), 7.87 (d, *J* = 8.0 Hz, 1H), 7.70 (dd, *J* = 6.9, 2.6 Hz, 1H), 7.56 – 7.44 (m, 2H), 7.44 – 7.32 (m, 4H), 6.91 – 6.82 (m, 3H), 3.80 (s, 3H), 3.25 (dd, *J* = 13.3, 12.4 Hz, 2H), 1.82 (s, 3H) ppm; **^{13}C NMR (101 MHz, CDCl$_3$)**: δ 159.5, 144.0, 143.0, 134.4, 131.1, 129.2, 127.9, 126.9, 126.1, 125.6, 125.3, 125.2, 125.1, 124.4, 113.8, 55.4, 46.5, 44.2, 28.3 ppm; **IR (ATR)**: 3043, 2955, 2930, 2860, 2834, 1623, 1603, 1507, 1464, 1301, 1247, 1172, 1128, 1036, 818, 801, 778, 667, 614, 435 cm^{-1}; **HRMS (ESI)**: calcd. for $[C_{22}H_{20}O+Na]^+$, $[M+Na]^+$: 323.1406; found: 323.1403; ***R*f**: 0.34 (Hexane/EtOAc, 95:5); **m.p.**: 87–89°C; $[\alpha]_D^{20}$: +393.6 (c = 0.5, CHCl$_3$); HPLC analysis: Chiralpak ADH, 4.6 x 250 mm; 0.5:99.5 i-PrOH/hexane, 1.0 mL/min, λ = 254 nm; τmajor = 9.4 min, τminor = 12.7 min, 86% ee.

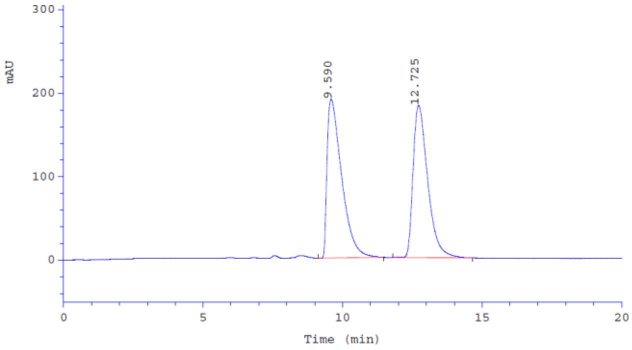

	Ret.time [min]	Start [min]	End [min]	Amount	Area [mAU*min]	Height [mAU]	% Area
1	9.590	9.12	11.47	-1	115.252	190.549	50.9297
2	12.725	11.79	14.67	-1	111.045	182.512	49.0703

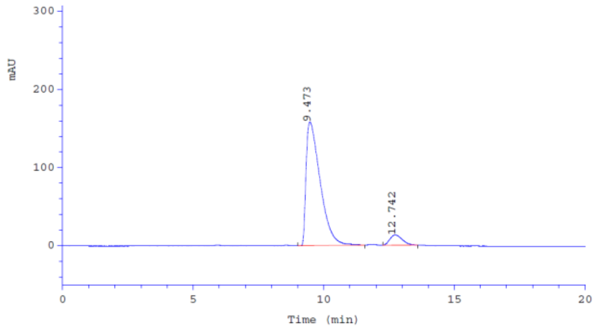

	Ret.time [min]	Start [min]	End [min]	Amount	Area [mAU*min]	Height [mAU]	% Area
1	9.473	9.01	11.56	-1	98.8492	158.505	93.2331
2	12.742	12.26	13.60	-1	7.17446	13.1564	6.7668

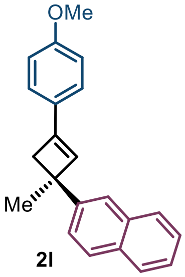

2l

(*S*)-2-(3-(4-methoxyphenyl)-1-methylcyclobut-2-en-1-yl)naphthalene (2l). Following the General Procedure D. 1-(4-methoxyphenyl)-3-methyl-3-(naphthalen-2-yl)cyclobutan-1-ol **1l** (31.8 mg, 0.100 mmol, 1 equiv., *cis/trans* 1.5:1), cat. **C5** (4.2 mg, 5 μmol, 0.05 equiv.), mesitylene (1 ml), reaction time 24 h. The crude mixture was purified by column chromatography (EtOAc-hexane 1-6%) to afford the product as a white solid in 67% yield, 91% ee (20.0 mg, 0.067 mmol).

Reaction carried out for 48 h: 97% yield, 86% ee.

^{1}H NMR (400 MHz, CDCl$_3$): δ 7.84 – 7.70 (m, 4H), 7.56 (dd, J = 8.5, 1.9 Hz, 1H), 7.48 – 7.38 (m, 2H), 7.38 – 7.33 (m, 2H), 6.91 – 6.85 (m, 2H), 6.67 (s, 1H), 3.82 (s, 3H), 3.08 – 2.92 (m, 2H), 1.71 (s, 3H) ppm; **^{13}C NMR (101 MHz, CDCl$_3$)**: δ 159.5, 145.5, 143.5, 131.9, 131.4, 127.9, 127.8, 127.6, 126.0, 125.3, 125.1, 123.9, 113.9, 46.0, 44.3, 27.5 ppm; **IR (ATR):** 3053, 2954 2924, 2857, 2835, 1622, 1602, 1574, 1507, 1464, 1420, 1316, 1301, 1249, 1172, 1130, 1105, 1036, 1016, 953, 854, 818, 795, 784, 764, 748, 621, 477 cm^{-1}; **HRMS (ESI)**: calcd. for [$C_{22}H_{20}O$+Na]$^+$, [M+Na]$^+$: 323.1412; found: 323.1407; ***R*f**: 0.35 (Hexane/EtOAc, 95:5); **m.p.**: 101–103°C; $[\alpha]_D^{20}$: -6.9 (c = 1.00, CHCl$_3$); **HPLC analysis**: Chiralpak IA, 4.6 x 250 mm; 0.5:99.5 i-PrOH/hexane, 1.0 mL/min, λ = 254 nm; τmajor= 10.6 min, τminor= 14.5 min, 91% ee.

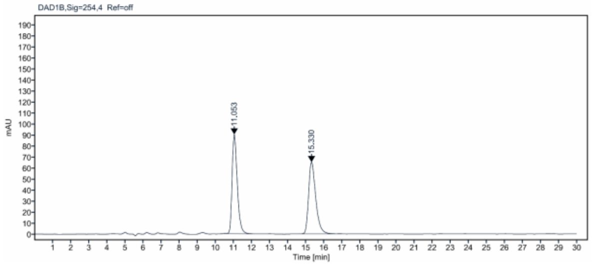

Signal: DAD1B,Sig=254,4 Ref=off

RT [min]	Width [min]	Area	Height	Area%
11.053	1.6964	1789.4663	89.9632	50.0055
15.330	2.4897	1789.0711	65.1531	49.9945

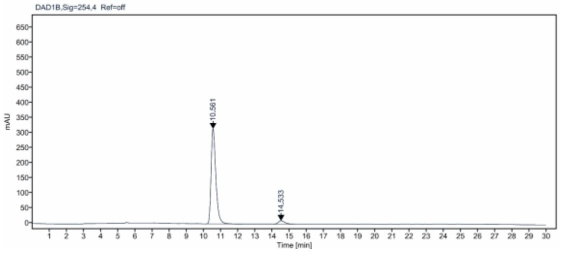

Signal: DAD1B,Sig=254,4 Ref=off

RT [min]	Width [min]	Area	Height	Area%
10.561	2.1733	6060.0259	315.6791	95.4927
14.533	1.2978	286.0363	11.0914	4.5073

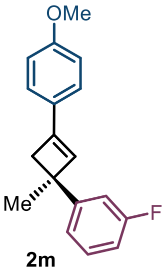

2m

(*S*)-1-fluoro-3-(3-(4-methoxyphenyl)-1-methylcyclobut-2-en-1-yl)benzene (**2m**). Following the General Procedure D. 3-(3-fluorophenyl)-1-(4-methoxyphenyl)-3-methylcyclobutan-1-ol **1m** (29.0 mg, 0.100 mmol, 1 equiv., *cis/trans* 1.3:1), cat. **C5** (4.2 mg, 5 µmol, 0.05 equiv.), mesitylene (1 ml), reaction time 48 h. The crude mixture was purified by column chromatography (EtOAc-hexane, 1-6%) to afford the product as a white solid in 70% yield, with a 91% ee (19.0 mg, 0.071 mmol).

^{1}H NMR (400 MHz, CDCl$_3$): δ 7.31 (d, J = 8.7 Hz, 2H), 7.28 – 7.21 (m, 1H), 7.14 (d, J = 8.1 Hz, 1H), 7.07 (d, J = 10.5 Hz, 1H), 6.86 (d, J = 8.7 Hz, 3H), 6.51 (s, 1H), 3.81 (s, 3H), 2.92 – 2.3 (m, 2H), 1.60 (s, 3H) ppm; **^{13}C NMR (101 MHz, CDCl$_3$)**: δ 162.9 (d, J = 245.0 Hz), 159.6, 150.8 (d, J = 6.6 Hz), 143.7, 130.7, 129.6 (d, J = 8.2 Hz), 127.7, 126.1, 121.6 (d, J = 2.5 Hz), 113.7, 113.1 (d, J = 21.2 Hz), 112.5 (d, J = 21.1 Hz), 55.4, 45.7, 44.4, 27.4 ppm; **^{19}F NMR (376 MHz, CDCl$_3$)**: δ -113.60 ppm; **IR (ATR)**: 2955, 2915, 2862, 2834, 1618, 1601, 1508, 1494, 1438, 1318, 1299, 1252, 1186, 1107, 1031, 1017, 839, 823, 788, 698, 160, 417 cm^{-1} **HRMS (ESI)**: calcd. for $[C_{18}H_{17}FO+H]^+$, $[M+H]^+$: 269.1336; found: 269.1333; ***R*$_f$**: 0.51 (Hexane/EtOAc, 95:5); **m.p.**: 99–101°C; $[\alpha]_D^{20}$: +60.6 (c = 1.00, CHCl$_3$); HPLC analysis: Chiralpak ADH, 4.6 x 250 mm; 0.8:99.2 i-PrOH/hexane, 1.0 mL/min, λ = 254 nm; τmajor= 7.2 min, τminor= 8.7 min, 91% ee.

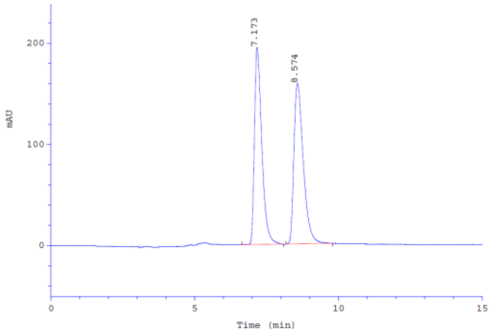

	Ret.time [min]	Start [min]	End [min]	Amount	Area [mAU*min]	Height [mAU]	% Area
1	7.173	6.64	8.08	-1	57.742	194.83	49.1890
2	8.574	8.17	9.80	-1	59.6461	158.531	50.8110

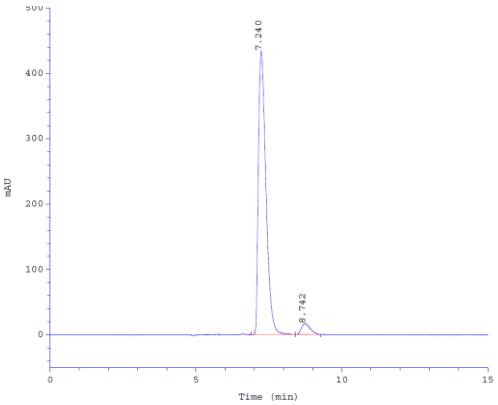

	Ret.time [min]	Start [min]	End [min]	Amount	Area [mAU*min]	Height [mAU]	% Area
1	7.240	6.90	8.41	-1	130.126	434.112	95.7278
2	8.742	8.41	9.29	-1	5.80739	16.6134	4.2722

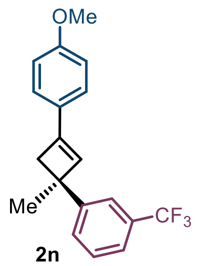

2n

(*S*)-1-(3-(4-methoxyphenyl)-1-methylcyclobut-2-en-1-yl)-3-(trifluoromethyl)benzene (**2n**). Following the General Procedure D. 1-(4-methoxyphenyl)-3-methyl-3-(3-(trifluoromethyl)phenyl)cyclobutan-1-ol **1n** (33.6 mg, 0.100 mmol, 1 equiv., *trans/cis* 1.2:1), cat. **C5** (4.2 mg, 5 µmol, 0.05 equiv.), mesitylene (1 ml), reaction time 48 h. The crude mixture was purified by column chromatography (EtOAc-hexane, 1-6%) to afford the product as a white solid in 79% yield, with a 92% ee (25.0 mg, 0.079 mmol).

^{1}H NMR (400 MHz, CDCl$_3$): δ 7.61 (s, 1H), 7.56 (d, *J* = 6.9 Hz, 1H), 7.46 – 7.38 (m, 2H), 7.32 (d, *J* = 8.9 Hz, 2H), 6.87 (d, *J* = 8.8 Hz, 2H), 6.55 (s, 1H), 3.81 (s, 3H), 2.91 (s, 2H), 1.62 (s, 3H); **^{13}C NMR (101 MHz, CDCl$_3$)**: δ 159.7, 149.0, 143.8, 130.6, 130.3, 130.3, 129.5, 128.6, 127.5, 126.2, 125.8, 122.6 (sext, *J* = 4.1 Hz), 113.9, 55.4, 45.6, 44.4, 27.6; **^{19}F (376 MHz, CDCl$_3$)**: δ -62.28; **IR (ATR)**: 2957, 1603, 1508, 1441, 1301, 1249, 1165, 1224, 1073, 1037, 839, 802, 702 cm^{-1}; **HRMS (ESI)**: calcd. for $[C_{19}H_{17}F_3O+H]^+$, $[M+H]^+$: 319.1304; found: 319.1301; ***R*f**: 0.53 (Hexane/EtOAc, 95:5); **m.p.**: 58–59°C; $[\alpha]_D^{20}$: +53.8 (c = 1.00, CHCl$_3$); HPLC analysis: Chiralpak OJH, 4.6 x 250 mm; 0.5:99.5 i-PrOH/hexane, 1.0 mL/min, λ = 254 nm; τmajor = 24.4 min, τminor = 32.1 min, 92% ee.

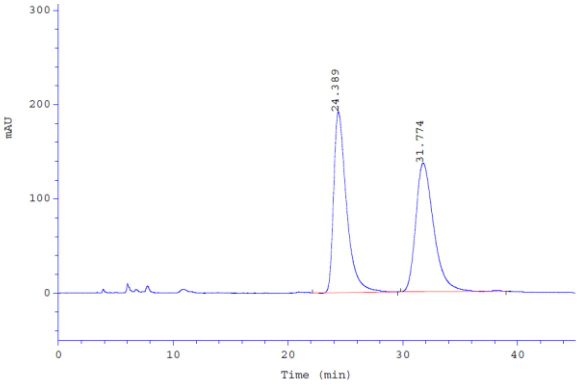

	Ret.time [min]	Start [min]	End [min]	Amount	Area [mAU*min]	Height [mAU]	% Area
1	24.389	22.12	29.54	-1	249.199	191.609	50.2779
2	31.774	29.82	39.00	-1	246.444	136.325	49.7221

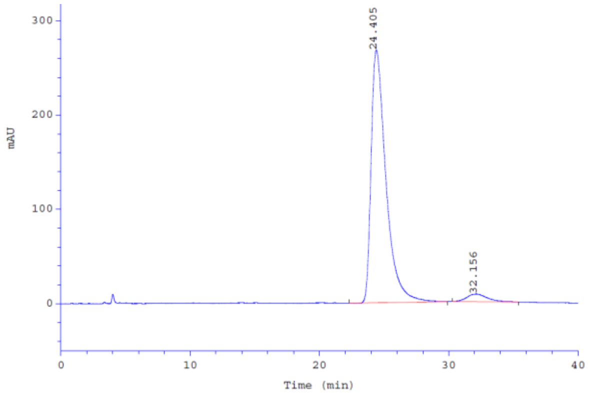

	Ret.time [min]	Start [min]	End [min]	Amount	Area [mAU*min]	Height [mAU]	% Area
1	24.405	22.30	29.91	-1	355.644	268.111	96.0156
2	32.156	30.28	35.39	-1	14.7583	8.17703	3.9844

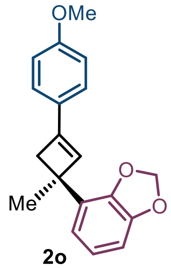

2o

(*S*)-4-(3-(4-methoxyphenyl)-1-methylcyclobut-2-en-1-yl)benzo[d][1,3]dioxole (**2o**). Following the General Procedure D. 3-(benzo[d][1,3]dioxol-4-yl)-1-(4-methoxyphenyl)-3-methylcyclobutan-1-ol **1o** (31.2 mg, 0.100 mmol, 1 equiv., *cis/trans* 2:1), cat. **C5** (4.2 mg, 5 µmol, 0.05 equiv.), mesitylene (1 ml), reaction time 48 h. The crude mixture was purified by column chromatography (EtOAc-hexane, 1-6%) to afford the product as a white solid in 88% yield, with a 90% ee (26.0 mg, 0.088 mmol).

Reaction carried out for 24 h: 30% yield, 89% ee.

^{1}H NMR (400 MHz, CDCl$_3$): δ 7.30 (d, *J* = 8.7 Hz, 2H), 6.90 – 6.79 (m, 4H), 6.74 (d, *J* = 8.0 Hz, 1H), 6.50 (s, 1H), 5.91 (s, 2H), 3.80 (s, 3H), 2.84 (dd, *J* = 12.7, 2.2 Hz, 2H), 1.57 (s, 3H) ppm; **^{13}C NMR (101 MHz, CDCl$_3$)**: δ 159.5, 147.5, 145.4, 143.3, 142.3, 131.2, 127.9, 126.1, 118.7, 113.8, 107.9, 106.9, 100.9, 55.4, 45.7, 44.5, 27.9 ppm; **IR (ATR)**: 2953, 2912, 2835, 1622, 1603, 1507, 1485, 1432, 1301, 1284, 1220, 1172, 1105, 1038, 938, 831, 810, 798, 636, 618 cm^{-1}; **HRMS (ESI)**: calcd. for [$C_{19}H_{18}O_3$+Na]$^+$, [M+Na]$^+$: 317.1148; found: 317.1144; ***R*f**: 0.32 (Hexane/EtOAc, 9:1); **m.p.**: 80–82°C; $[\alpha]_D^{20}$: +63.8 (c = 1.00, CHCl$_3$); HPLC analysis: Chiralpak ADH, 4.6 x 250 mm; 1:99 i-PrOH/hexane, 1.0 mL/min, λ = 254 nm; τmajor= 13.9 min, τminor= 21.1 min, 90% ee.

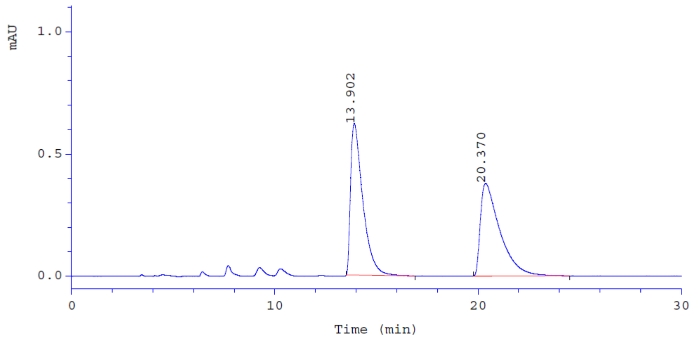

	Ret.time [min]	Start [min]	End [min]	Amount	Area [mAU*min]	Height [mAU]	% Area
1	13.902	13.52	16.90	-1	431.649	619.981	50.6480
2	20.370	19.78	24.51	-1	420.604	378.33	49.3520

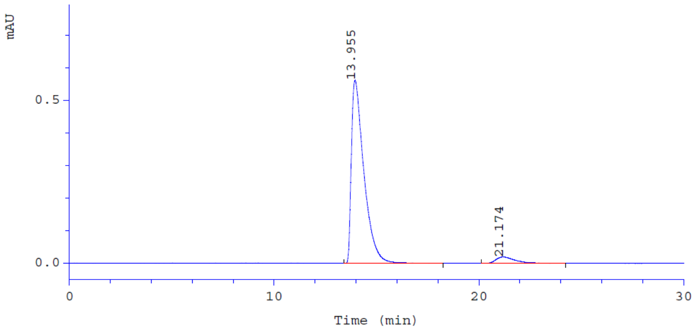

	Ret.time [min]	Start [min]	End [min]	Amount	Area [mAU*min]	Height [mAU]	% Area
1	13.955	13.42	18.24	0	399.467	562.608	94.8656
2	21.174	20.12	24.24	0	21.6204	19.4687	5.1344

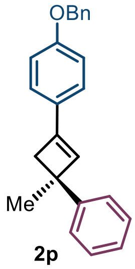

2p

(*S*)-1-(benzyloxy)-4-(3-methyl-3-phenylcyclobut-1-en-1-yl)benzene (2p). Following the General Procedure D. 1-(4-(benzyloxy)phenyl)-3-methyl-3-phenylcyclobutan-1-ol **1p** (34.4 mg, 0.100 mmol, 1 equiv., *cis/trans* 2:1), cat. **C5** (4.2 mg, 5 μmol, 0.05 equiv.), mesitylene (1 ml), reaction time 48 h. The crude mixture was purified by column chromatography (EtOAc-hexane, 1-6%) to afford the product as a white solid in 80% yield, with an 88% ee (26.0 mg, 0.080 mmol).

^{1}H NMR (400 MHz, $CDCl_3$): δ 7.46 – 7.28 (m, 11H), 7.19 (t, *J* = 7.4 Hz, 1H), 6.94 (d, *J* = 8.4 Hz, 2H), 6.58 (s, 1H), 5.08 (s, 2H), 2.91 (q, *J* = 12.5 Hz, 2H), 1.62 (s, 3H), 1.26 (s, 3H); **^{13}C NMR (101 MHz, $CDCl_3$):** δ 158.6, 148.0, 143.3, 137.0, 131.5, 128.7, 128.2, 128.1, 127.6, 126.1, 126.0, 125.7, 114.8, 70.1, 45.9, 44.4, 29.8, 27.8 ppm; **IR (ATR):** 3053, 2923, 1601, 1506, 1455, 1381, 1298, 1264, 1242, 1172, 1095, 1026, 895, 837, 802, 732, 699, 651, 536, 458 cm^{-1}; **HRMS (ESI)**: calcd. for $[C_{24}H_{22}O+Na]^+$, $[M+Na]^+$: 349.1563; found: 349.1563; ***R*f**: 0.48 (Hexane/EtOAc, 95:5); **m.p.**: 98–100°C; $[\alpha]_D^{20}$: +51.4 (c = 1.00, $CHCl_3$); HPLC analysis: Chiralpak IA, 4.6 x 250 mm; 0.5:99.5 i-PrOH/hexane, 1.0 mL/min, λ = 254 nm; τmajor= 11.0 min, τminor= 15.4 min, 88% ee.

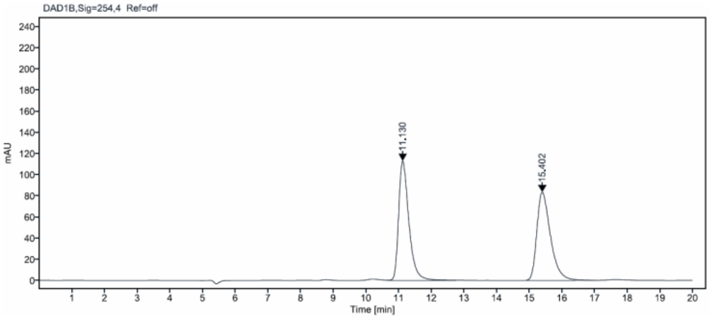

Signal: DAD1B,Sig=254,4 Ref=off

RT [min]	Width [min]	Area	Height	Area%
11.130	2.0733	2435.0079	112.6693	50.1433
15.402	2.2433	2421.0873	83.3659	49.8567

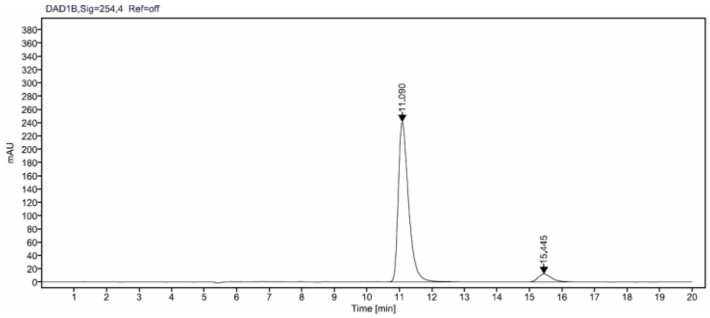

Signal: DAD1B,Sig=254,4 Ref=off

RT [min]	Width [min]	Area	Height	Area%
11.090	2.5800	5285.5005	240.7335	93.7611
15.445	1.7333	351.7021	11.9948	6.2389

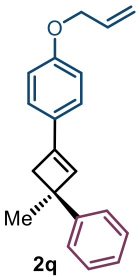

2q

(*S*)-1-(allyloxy)-4-(3-methyl-3-phenylcyclobut-1-en-1-yl)benzene (2q). Following the General Procedure D. 1-(4-(allyloxy)phenyl)-3-methyl-3-phenylcyclobutan-1-ol **1q** (29.4 mg, 0.100 mmol, 1 equiv., *cis/trans* 2:1), cat. **C5** (4.2 mg, 5 µmol, 0.05 equiv.), mesitylene (1 ml), reaction time 48 h. The crude mixture was purified by column chromatography (EtOAc-hexane, 1-6%) to afford the product as a white solid in 83% yield, with a 89 % ee (23.0 mg, 0.083 mmol).

^{1}H NMR (400 MHz, $CDCl_3$): δ 7.39 (d, *J* = 8.0 Hz, 2H), 7.35 – 7.27 (m, 4H), 7.18 (t, *J* = 7.3 Hz, 1H), 6.88 (d, *J* = 8.6 Hz, 2H), 6.58 (s, 1H), 6.10 – 5.98 (m, 1H), 5.41 (d, *J* = 17.2 Hz, 1H), 5.28 (d, *J* = 10.2 Hz, 1H), 4.54 (d, *J* = 5.3 Hz, 2H), 2.90 (dd, *J* = 12.5, 11.8 Hz, 2H), 1.61 (s, 3H) ppm; **^{13}C NMR (101 MHz, $CDCl_3$):** δ 158.4, 148.1, 143.3, 133.2, 131.4, 128.2, 128.1, 126.0, 126.0, 125.7, 117.9, 114.6, 68.9, 45.9, 44.4, 27.8 ppm; **IR (ATR)**: 3068 3027, 2964, 2916, 2861, 1619, 1600, 1507, 1494, 1455, 1318, 1297, 1249, 1184, 1174, 1024, 995, 936, 842, 808, 760, 720, 698, 537, 458 cm^{-1}; **HRMS (ESI)**: calcd. for $[C_{20}H_{20}O+Na]^+$, $[M+Na]^+$: 299.1406; found: 299.1402; ***R*f**: 0.49 (Hexane/EtOAc, 95:5); **m.p.**: 93–95°C; $[\alpha]_D^{20}$: +61.2 (c = 1.00, $CHCl_3$); HPLC analysis: Chiralpak IA, 4.6 x 250 mm; 0.5:99.5 i-PrOH/hexane, 1.0 mL/min, λ = 254 nm; τmajor = 6.9 min, τminor = 8.4 min, 89% ee.

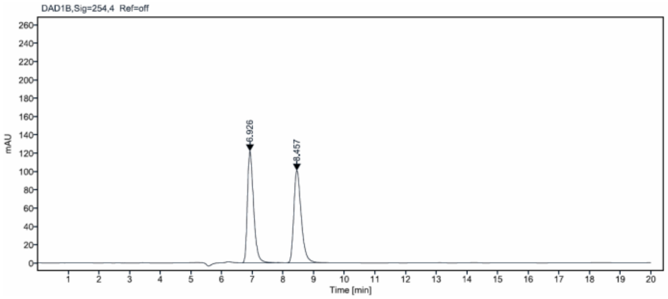

Signal: DAD1B,Sig=254,4 Ref=off

RT [min]	Width [min]	Area	Height	Area%
6.926	1.4063	1695.3485	121.7898	49.9947
8.457	1.3400	1695.7047	100.7363	50.0053

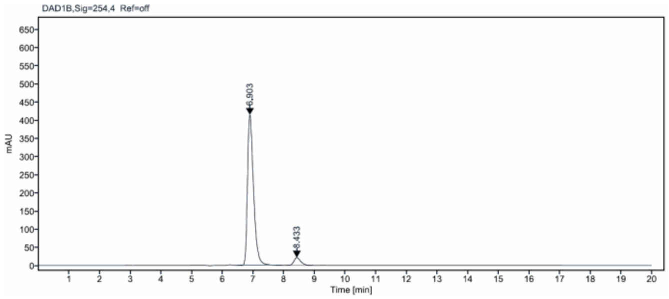

Signal: DAD1B,Sig=254,4 Ref=off

RT [min]	Width [min]	Area	Height	Area%
6.903	1.4328	5665.2347	413.7205	94.7153
8.433	0.8756	316.0949	20.7266	5.2847

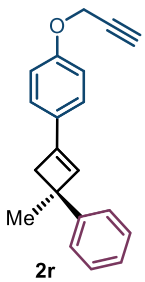

2r

(*S*)-1-(3-methyl-3-phenylcyclobut-1-en-1-yl)-4-(prop-2-yn-1-yloxy)benzene (2r). Following the General Procedure D. 3-methyl-3-phenyl-1-(4-(prop-2-yn-1-yloxy)phenyl)cyclobutan-1-ol **1r** (30.0 mg, 0.100 mmol, 1 equiv., *cis/trans* 5.7:1), cat. **C5** (17.0 mg, 20 µmol, 0.20 equiv.), mesitylene (1 ml), reaction time 48 h. The crude mixture was purified by column chromatography (EtOAc-hexane, 1-6%) to afford the product as a white solid in 96% yield, with a 89% ee (27.0 mg, 0.098 mmol).

^{1}H NMR (400 MHz, C_6D_6): δ 7.27 (d, *J* = 7.0 Hz, 2H), 7.20 – 7.12 (m, 4H), 7.05 (t, *J* = 7.3 Hz, 1H), 6.76 (d, *J* = 8.7 Hz, 2H), 6.36 (s, 1H), 4.11 (d, *J* = 2.4 Hz, 2H), 2.79 (d, *J* = 12.4 Hz, 1H), 2.66 (d, *J* = 12.4 Hz, 1H), 1.92 (t, *J* = 2.4 Hz, 1H), 1.50 (s, 3H) ppm; **^{13}C NMR (101 MHz, C_6D_6):** δ 157.6, 147.9, 143.4, 131.5, 128.7, 128.1, 127.6, 126.1, 126.0, 125.7, 114.8, 78.6, 75.4, 55.3, 45.7, 44.2, 27.6 ppm; **IR (ATR)**: 3287, 3052, 3027, 2962, 2949, 2916, 2861, 1618, 1600, 1506, 1492, 1454, 1376, 1299, 1243, 1185, 1176, 1140, 1067, 1028, 977, 843, 807, 764, 681, 533, 457 cm^{-}1; **HRMS (ESI)**: calcd. for $[C_{20}H_{18}O+H]^{+}$, $[M+H]^{+}$: 275.1430; found: 275.1428; ***R*f**: 0.42 (Hexane/EtOAc, 95:5); **m.p.**: 107–109°C; $[\boldsymbol{\alpha}]_{\mathbf{D}}^{\mathbf{20}}$: +61.8 (c = 1.00, $CHCl_3$); HPLC analysis: Chiralpak IA, 4.6 x 250 mm; 0.5:99.5 i-PrOH/hexane, 1.0 mL/min, λ = 254 nm; τmajor= 11.0 min, τminor= 14.2 min, 89% ee.

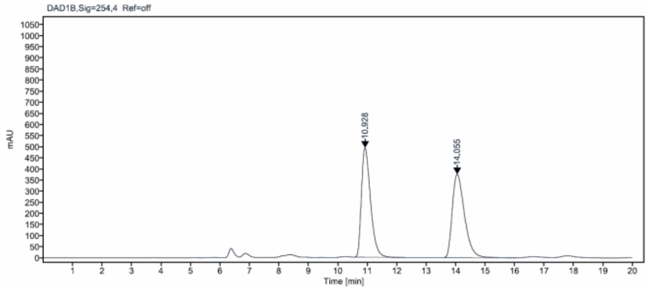

Signal: DAD1B,Sig=254,4 Ref=off

RT [min]	Width [min]	Area	Height	Area%
10.928	1.9656	10611.3037	492.4454	49.4533
14.055	2.5122	10845.9018	375.5740	50.5467

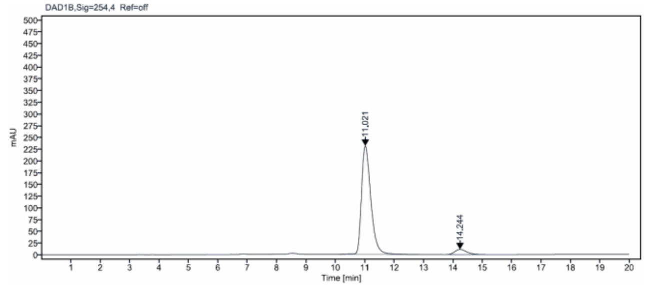

Signal: DAD1B,Sig=254,4 Ref=off

RT [min]	Width [min]	Area	Height	Area%
11.021	2.6979	5075.1796	230.7799	94.3490
14.244	1.6667	303.9763	10.8467	5.6510

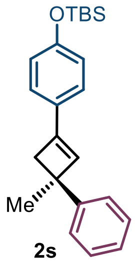

2s

(*S*)-*tert*-butyldimethyl(4-(3-methyl-3-phenylcyclobut-1-en-1-yl)phenoxy)silane (2s). Following the General Procedure D. 1-(4-((*tert*-butyldimethylsilyl)oxy)phenyl)-3-methyl-3-phenylcyclobutan-1-ol **1s** (36.9 mg, 0.100 mmol, 1 equiv., *cis/trans* 1.5:1), cat. **C5** (16.9 mg, 20 µmol, 0.2 equiv.), mesitylene (1 ml), reaction time 48 h. The crude mixture was purified by column chromatography (EtOAc-hexane 1-5%) to afford the product as a colorless oil in 54% yield, 85% ee (18.8 mg, 0.054 mmol).

^{1}H NMR (400 MHz, C_6D_6): δ 7.40 – 7.30 (m, 2H), 7.27 – 7.19 (m, 4H), 7.14 – 7.09 (m, 1H), 6.89 – 6.82 (m, 2H), 6.44 (s, 1H), 2.88 (d, J = 12.4 Hz, 1H), 2.74 (d, J = 12.5 Hz, 1H), 1.57 (s, 3H), 1.01 (s, 9H), 0.14 (s, 6H) ppm; **^{13}C NMR (101 MHz, C_6D_6)**: δ 156.0, 148.2, 143.8, 131.7, 129.1, 128.5, 126.6, 126.3, 126.0, 120.4, 46.0, 44.5, 28.0, 25.9, 18.5, -4.3 ppm; **IR (ATR):** 2929, 2857, 2360, 1598, 1505, 1472, 1288, 1252, 1166, 1096, 908, 839, 804, 782, 734, 699, 669, 536, 457 cm^{-1}; **HRMS (ESI)**: calcd. for $[C_{23}H_{30}OSi+Na]^+$, $[M+Na]^+$: 373.1964; found: 373.1961; ***R*f**: 0.35 (Hexane/EtOAc, 98:2); $[\alpha]_D^{20}$: +39.7 (C=0.94, DCM); HPLC analysis: Chiralpak IA, 4.6 x 250 mm; 2.0:98.0 DCM/hexane, 0.8 mL/min, λ = 254 nm; τmajor= 8.1 min, τminor= 9.3 min, 85% ee.

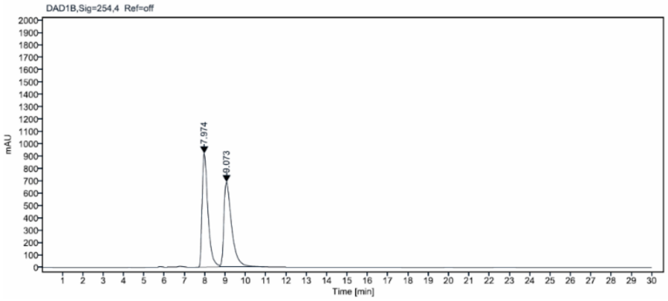

Signal: DAD1B,Sig=254,4 Ref=off

RT [min]	Width [min]	Area	Height	Area%
7.974	1.2067	18326.7115	919.5640	50.1864
9.073	2.1813	18190.5871	685.4875	49.8136

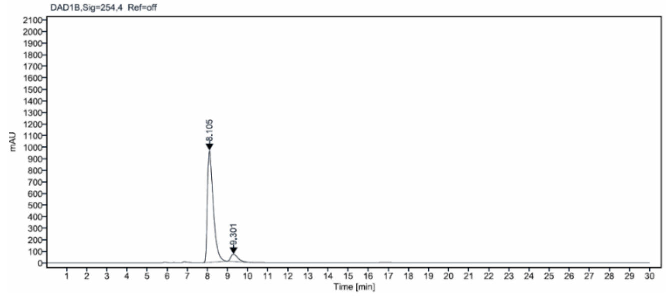

Signal: DAD1B,Sig=254,4 Ref=off

RT [min]	Width [min]	Area	Height	Area%
8.105	1.2144	19650.4881	967.0914	92.6011
9.301	1.2016	1570.0981	64.1453	7.3989

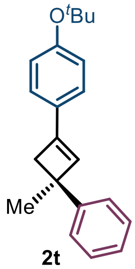

2t

(*S*)-1-(*tert*-butoxy)-4-(3-methyl-3-phenylcyclobut-1-en-1-yl)benzene (2t). Following the General Procedure D. 1-(4-(*tert*-butoxy)phenyl)-3-methyl-3-phenylcyclobutan-1-ol **1t** (31.0 mg, 0.100 mmol, 1 equiv., *cis/trans* 5.4:1), cat. **C5** (16.9 mg, 20 µmol, 0.2 equiv.), mesitylene (1 ml), reaction time 48 h. The crude mixture was purified by column chromatography (EtOAc-hexane 1-3%) to afford the product as a white solid in 81% yield, 85% ee (23.8 mg, 0.081 mmol).

^{1}H NMR (400 MHz, C_6D_6): δ 7.41 – 7.30 (m, 2H), 7.28 – 7.18 (m, 4H), 7.15 – 7.08 (m, 1H), 7.01 – 6.92 (m, 2H), 6.46 (s, 1H), 2.88 (d, J = 12.5 Hz, 1H), 2.74 (d, J = 12.5 Hz, 1H), 1.57 (s, 3H), 1.24 (s, 9H) ppm; **^{13}C NMR (101 MHz, C_6D_6)**: δ 156.1, 148.2, 143.9, 132.4, 130.4, 128.5, 126.3, 126.0, 125.9, 124.2, 78.3, 46.0, 44.6, 29.0, 27.9 ppm; **IR (ATR):** 2975, 1600, 1499, 1389, 1365, 1292, 1238, 1159, 1095, 1028, 896, 853, 803, 699, 545 cm^{-1}; **HRMS (ESI)**: calcd. for $[C_{21}H_{24}O+Na]^+$, $[M+Na]^+$: 315.1725; found: 315.1715; ***R*f**: 0.47 (Hexane/EtOAc, 95:5); **m.p.**: 49–50°C; $[\alpha]_D^{20}$: +50.218 (C=1.00, DCM); HPLC analysis: Chiralpak IF, 4.6 x 250 mm; 2.0:98.0 DCM/hexane, 1.0 mL/min, λ = 254 nm; τmajor= 28.9 min, τminor= 22.3 min, 85% ee.

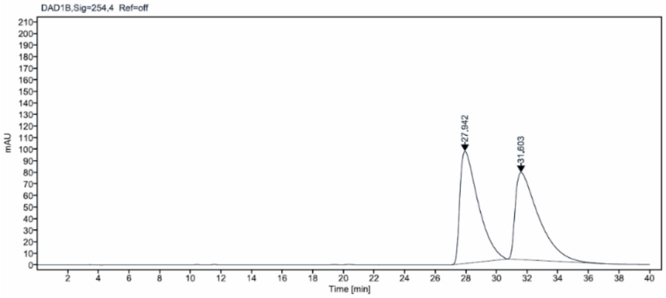

Signal: DAD1B,Sig=254,4 Ref=off

RT [min]	Width [min]	Area	Height	Area%
27.942	3.6620	8116.1928	96.8529	50.3452
31.603	6.4375	8004.8823	75.2339	49.6548

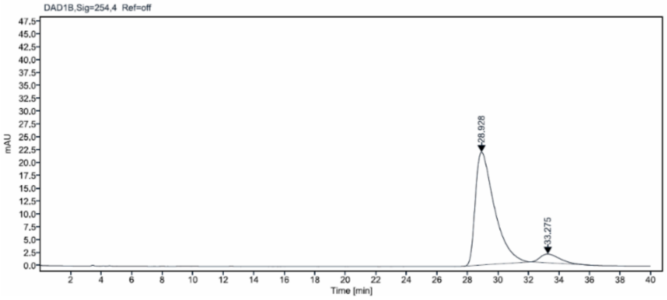

Signal: DAD1B,Sig=254,4 Ref=off

RT [min]	Width [min]	Area	Height	Area%
28.928	4.4167	1909.0581	21.9451	92.5589
33.275	3.8333	153.4752	1.7135	7.4411

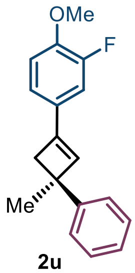
2u

(*S*)-2-fluoro-1-methoxy-4-(3-methyl-3-phenylcyclobut-1-en-1-yl)benzene (2u). Following the General Procedure D. 1-(3-fluoro-4-methoxyphenyl)-3-methyl-3-phenylcyclobutan-1-ol **1u** (28.6 mg, 0.100 mmol, 1 equiv., *cis/trans* 3.6:1), cat. **C5** (16.9 mg, 20 μmol, 0.05 equiv.), mesitylene (1 ml), reaction time 48 h. The crude mixture was purified by column chromatography (EtOAc-hexane, 1-6%) to afford the product as a white gel in 26% yield, with a 96% ee (7.0 mg, 0.026 mmol).

^{1}H NMR (400 MHz, CDCl$_3$): δ 7.39 – 7.34 (m, 2H), 7.31 (t, *J* = 7.7 Hz, 2H), 7.18 (t, *J* = 7.2 Hz, 1H), 7.11 (dd, *J* = 12.1, 2.0 Hz, 1H), 7.05 (d, *J* = 8.4 Hz, 1H), 6.90 (t, *J* = 8.5 Hz, 1H), 6.60 (s, 1H), 3.88 (s, 3H), 2.88 (q, *J* = 12.5 Hz, 2H), 1.60 (s, 3H) ppm; **^{13}C NMR (101 MHz, CDCl$_3$)**: δ 152.4 (d, *J* = 245.7 Hz), 147.7, 147.5 (d, *J* = 10.8 Hz), 142.5 (d, *J* = 2.2 Hz), 132.8, 128.6 (d, *J* = 6.4 Hz), 128.2, 125.9, 125.8, 120.8 (d, *J* = 3.4 Hz), 113.1 (d, *J* = 2.2 Hz), 112.4 (d, *J* = 18.3 Hz), 56.4, 46.0, 44.3, 27.8 ppm; **^{19}F NMR (376 MHz, CDCl$_3$):** δ -135.71 ppm; **IR (ATR)**: 2954, 2838, 1600, 1577, 1510, 1494, 1440, 1323, 1273, 1231, 1198, 1171, 1127, 1094, 1070, 1028, 950, 872, 798, 758, 698, 628, 547, 492, 445 cm^{-1}; **HRMS (ESI)**: calcd. for $[C_{18}H_{17}FO+Na]^+$, $[M+Na]^+$: 291.1156; found: 291.1152; ***R*f**: 0.35 (Hexane/EtOAc, 95:5); $[\alpha]_D^{20}$: +56.9 (c = 0.5, CHCl$_3$); HPLC analysis: Chiralpak IB, 4.6 x 250 mm; 1.0:99.0 DCM/hexane, 1.0 mL/min, λ = 254 nm; τmajor= 18.9 min, τminor= 23.1, min, 96% ee.

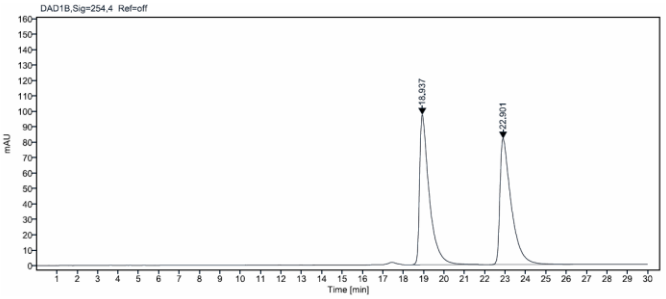

Signal: DAD1B,Sig=254,4 Ref=off

RT [min]	Width [min]	Area	Height	Area%
18.937	3.6322	3186.9580	97.1988	50.3853
22.901	4.0967	3138.2200	81.6496	49.6147

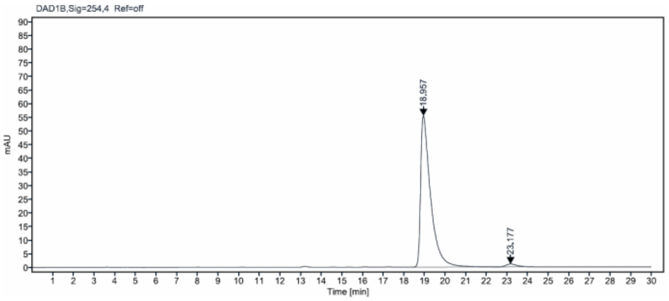

Signal: DAD1B,Sig=254,4 Ref=off

RT [min]	Width [min]	Area	Height	Area%
18.957	4.0433	1850.9531	55.4245	97.9524
23.177	1.7922	38.6926	1.0593	2.0476

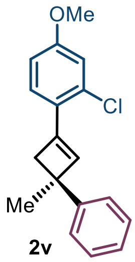

(*S*)-2-chloro-4-methoxy-1-(3-methyl-3-phenylcyclobut-1-en-1-yl)benzene (2v). Following the General Procedure D. 1-(2-chloro-4-methoxyphenyl)-3-methyl-3-phenylcyclobutan-1-ol **1v** (30.3 mg, 0.100 mmol, 1 equiv., *cis/trans* 2:1), cat. **C5** (16.9 mg, 20 µmol, 0.05 equiv.), mesitylene (1 ml), reaction time 48 h. The crude mixture was purified by column chromatography (EtOAc-hexane, 1-6%) to afford the product as a white solid in 98% yield, with a 91% ee (28.0 mg, 0.098 mmol).

^{1}H NMR (400 MHz, CDCl$_3$): δ 7.40 (d, *J* = 8.1 Hz, 2H), 7.31 (t, *J* = 7.7 Hz, 2H), 7.21 – 7.13 (m, 2H), 6.91 (d, *J* = 2.6 Hz, 1H), 6.88 (s, 1H), 6.77 (dd, *J* = 8.6, 2.6 Hz, 1H), 3.79 (s, 3H), 2.99 (q, *J* = 12.4 Hz, 2H), 1.62 (s, 3H) ppm; **^{13}C NMR (101 MHz, CDCl$_3$)**: δ 159.3, 147.8, 140.4, 137.9, 133.9, 128.7, 128.2, 126.0, 125.8, 125.4, 115.6, 112.8, 55.6, 46.3, 46.1, 27.7 ppm; **IR (ATR)**: 2955, 1597, 1490, 1438, 1278, 1220, 1181, 1035, 912, 858, 799, 760, 697, 617, 550, 441, 419 cm^{-1}; **HRMS (ESI)**: calcd. for $[C_{18}H_{17}ClO+Na]^+$, $[M+Na]^+$: 307.0860; found: 307.0857; ***R*f**: 0.41 (Hexane/EtOAc, 95:5); **m.p.**: 52–55°C; $[\alpha]_D^{20}$: +99.8 (c = 1.00, CHCl$_3$); HPLC analysis: Chiralpak IA, 4.6 x 250 mm; 0.5:99.5 i-PrOH/hexane, 1.0 mL/min, λ = 254 nm; τmajor= 7.6 min, τminor= 8.7 min, 91% ee.

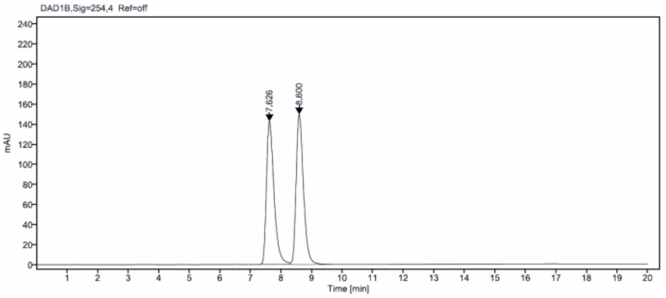

Signal: DAD1B,Sig=254,4 Ref=off

RT [min]	Width [min]	Area	Height	Area%
7.626	1.0670	2469.9650	142.8056	49.6263
8.600	1.9396	2507.1631	149.5610	50.3737

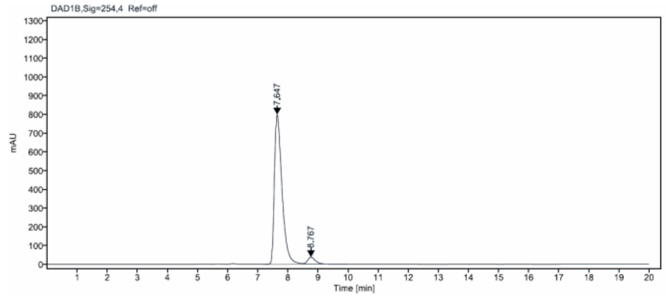

Signal: DAD1B,Sig=254,4 Ref=off

RT [min]	Width [min]	Area	Height	Area%
7.647	1.2727	14008.3254	798.7691	95.2524
8.767	1.4673	698.2104	37.4504	4.7476

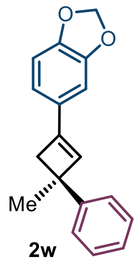

2w

(*S*)-5-(3-methyl-3-phenylcyclobut-1-en-1-yl)benzo[d][1,3]dioxole (2w). Following the General Procedure D. 1-(benzo[d][1,3]dioxol-5-yl)-3-methyl-3-phenylcyclobutan-1-ol **1w** (28.0 mg, 0.100 mmol, 1 equiv., *cis/trans* 2:1), cat. **C5** (16.9 mg, 20 µmol, 0.20 equiv.), mesitylene (1 ml), reaction time 48 h. The crude mixture was purified by column chromatography (EtOAc-hexane 1-6%) to afford the product as a white solid in 76% yield, 89% ee (20.0 mg, 0.076 mmol).

^{1}H NMR (400 MHz, C_6D_6): δ 7.26 – 7.21 (m, 2H), 7.15 (t, *J* = 7.6 Hz, 2H), 7.04 (t, *J* = 7.3 Hz, 1H), 6.87 (s, 1H), 6.60 (dd, *J* = 7.9, 12.7 Hz, 2H), 6.28 (s, 1H), 5.22 (s, 2H), 2.74 (d, *J* = 12.4 Hz, 1H), 2.60 (d, *J* = 12.4 Hz, 1H), 1.45 (s, 3H) ppm; **^{13}C NMR (101 MHz, C_6D_6)**: δ 148.1, 147.8, 147.7, 143.4, 131.7, 129.6, 128.1, 126.0, 125.7, 118.9, 108.1, 105.1, 100.8, 45.6, 44.2, 27.6 ppm; ***R*f**: 0.32 (Hexane/EtOAc, 95:5); $[\alpha]_D^{20}$: +97.6 (c = 1.00, $CHCl_3$); HPLC analysis: Chiralpak IF, 4.6 x 250 mm; 0.5:99.5 i-PrOH/hexane, 1.0 mL/min, λ = 254 nm; τmajor= 25.06 min, τminor= 20.73 min, 89% ee.

Spectral data match the ones reported in the literature.[13]

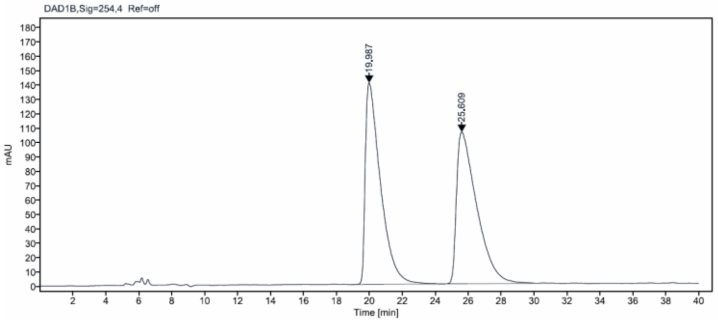

Signal: DAD1B,Sig=254,4 Ref=off

RT [min]	Width [min]	Area	Height	Area%
19.987	5.4467	9009.5160	140.2124	50.0925
25.609	5.7867	8976.2537	105.5702	49.9075

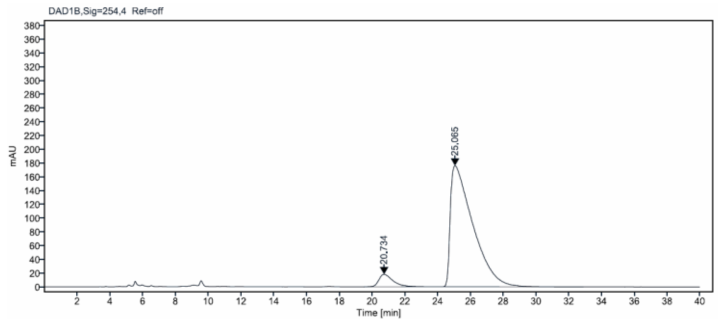

Signal: DAD1B,Sig=254,4 Ref=off

RT [min]	Width [min]	Area	Height	Area%
20.734	3.8933	1083.3600	18.1025	6.0641
25.065	8.6879	16781.6721	175.8737	93.9359

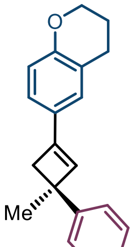

(*S*)-6-(3-methyl-3-phenylcyclobut-1-en-1-yl)chromane (**2x**). Following the General Procedure D. 1-(chroman-6-yl)-3-methyl-3-phenylcyclobutan-1-ol **1x** (29.4 mg, 0.100 mmol, 1 equiv., *cis/trans* 1:1), cat. **C5** (4.2 mg, 5 μmol, 0.05 equiv.), mesitylene (1 ml), reaction time 48 h. The crude mixture was purified by column chromatography (EtOAc-hexane, 1-6%) to afford the product as a white solid in 72% yield, with a 72% ee (20.0 mg, 0.072 mmol).

^{1}H NMR (400 MHz, CDCl$_3$): δ 7.38 (d, *J* = 6.8 Hz, 2H), 7.30 (t, *J* = 7.5 Hz, 2H), 7.16 (dd, *J* = 17.5, 7.7 Hz, 2H), 7.05 (s, 1H), 6.74 (d, *J* = 8.4 Hz, 1H), 6.53 (s, 1H), 4.22 – 4.13 (m, 2H), 2.88 (q, *J* = 12.5 Hz, 2H), 2.77 (t, *J* = 6.5 Hz, 2H), 2.04 – 1.95 (m, 2H), 1.60 (s, 3H) ppm; **^{13}C NMR (101 MHz, CDCl$_3$)**: δ 154.9, 148.1, 143.5, 131.0, 128.1, 127.4, 126.2, 125.9, 125.7, 123.9, 122.0, 116.7, 66.7, 45.7, 44.5, 27.7, 25.0, 22.4 ppm; **IR (ATR)**: 3023, 2951, 2864, 1623, 1599, 1494, 1310, 1261, 1229, 1123, 1095, 1062, 826, 802, 761, 700, 547 cm^{-1}; **HRMS (ESI)**: calcd. for [$C_{20}H_{20}O$+Na]$^+$, [M+Na]$^+$: 299.1406; found: 299.1411; ***R*f**: 0.48 (Hexane/EtOAc, 95:5); **m.p.**: 56–58°C. $[\alpha]_D^{20}$: +53.5 (c = 1.00, CHCl$_3$); HPLC analysis: Chiralpak IF, 4.6 x 250 mm; 0.5:99.5 i-PrOH/hexane, 1.0 mL/min, λ = 254 nm; τmajor = 9.9 min, τminor = 8.8 min, 72% ee.

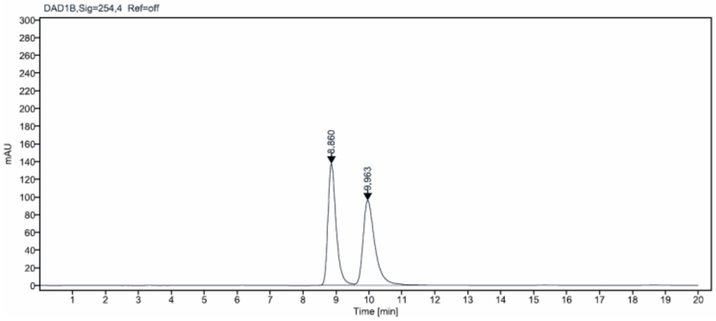

Signal: DAD1B,Sig=254,4 Ref=off

RT [min]	Width [min]	Area	Height	Area%
8.860	1.0795	2373.5281	137.5321	49.5109
9.963	3.0539	2420.4221	95.9272	50.4891

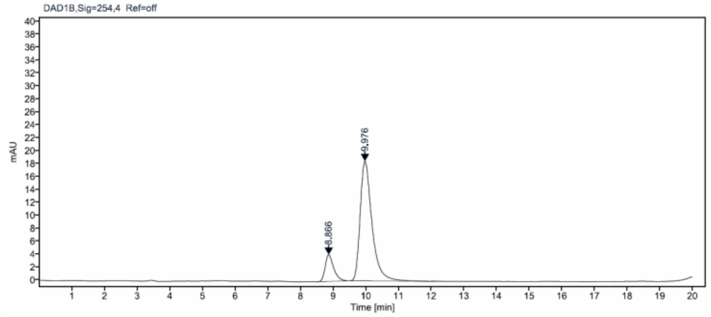

Signal: DAD1B,Sig=254,4 Ref=off

RT [min]	Width [min]	Area	Height	Area%
8.866	1.0033	74.7909	4.1212	13.8548
9.976	2.6324	465.0296	18.5158	86.1452

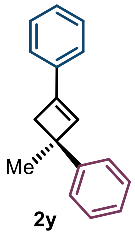

2y

(*S*)-(3-methylcyclobut-1-ene-1,3-diyl)dibenzene (2y). Following the General Procedure E. 3-methyl-1,3-diphenylcyclobutyl 2,2,2-trichloroacetimidate **1af** (38.3 mg, 0.100 mmol, 1 equiv., *cis/trans* 1.6:1), cat. **C5** (4.2 mg, 5 μmol, 0.05 equiv.), mesitylene (1 ml), reaction time 24 h. The crude mixture was purified by column chromatography (EtOAc-hexane, 1-6%) to afford the product as a white solid in 64% yield, with a 76% ee (14.0 mg, 0.064 mmol).

Employing (*S*)-**C4** (0.05 equiv): 30%, -30% ee.

^{1}H NMR (400 MHz, C_6D_6): δ 7.23 (t, *J* = 7.2 Hz, 4H), 7.16 (t, *J* = 7.8 Hz, 2H), 7.10 – 6.99 (m, 3H), 6.45 (s, 1H), 2.80 (d, *J* = 12.5 Hz, 1H), 2.66 (d, *J* = 12.5 Hz, 1H), 1.47 (s, 3H); **^{13}C NMR (101 MHz, C_6D_6)**: δ 147.63, 143.92, 134.85, 133.63, 128.26, 128.16, 125.97, 125.71, 124.76, 45.85, 44.08, 27.48; ***R*f**: 0.51 (Hexane/EtOAc, 98:2); $[\alpha]_D^{20}$: +36.2 (c = 1, $CHCl_3$); HPLC analysis: Chiralpak IF, 4.6 x 250 mm; 1.0:99.0 DCM/hexane, 1.0 mL/min, λ = 254 nm; τmajor= 11.4 min, τminor= 10.4, min, 76% ee.

Spectral data match the ones reported in the literature.[13]

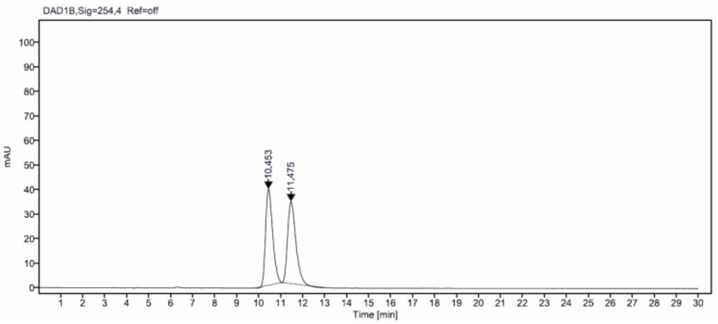

Signal: DAD1B,Sig=254,4 Ref=off

RT [min]	Width [min]	Area	Height	Area%
10.453	1.2450	868.0159	39.2196	49.9607
11.475	2.0278	869.3802	33.5216	50.0393

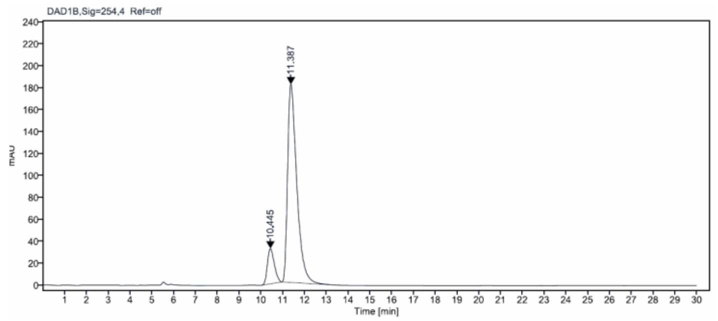

Signal: DAD1B,Sig=254,4 Ref=off

RT [min]	Width [min]	Area	Height	Area%
10.445	0.9161	706.0230	32.0786	11.8025
11.387	2.3490	5275.9583	180.5626	88.1975

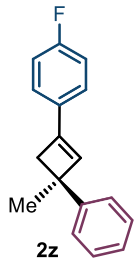

(*S*)-1-Fluoro-4-(3-methyl-3-phenylcyclobut-1-en-1-yl)benzene (2z). Following the General Procedure E. 1-(4-fluorophenyl)-3-methyl-3-phenylcyclobutyl 2,2,2-trichloroacetimidate **1ag** (40.1 mg, 0.100 mmol, 1 equiv., *cis/trans* 1.3:1), cat. **C5** (4.2 mg, 5 µmol, 0.05 equiv.), mesitylene (1 ml), reaction time 24 h. The crude mixture was purified by column chromatography on Al_2O_3 Brockman activity grade V (hexane 100%) to afford the product as a colorless oil in 67% yield, 73% ee (16.0 mg, 0.067 mmol).

Emplying (*S*)-**C4** (0.05 equiv): 25%, -42% ee.

^{1}H NMR (400 MHz, C_6D_6): δ 7.31 – 7.27 (m, 2H), 7.28 – 7.18 (m, 2H), 7.14 – 7.08 (m, 1H), 7.04 – 6.97 (m, 2H), 6.83 – 6.74 (m, 2H), 6.38 (s, 1H), 2.77 (d, *J* = 12.5 Hz, 1H), 2.63 (d, *J* = 12.5 Hz, 1H), 1.52 (s, 3H) ppm; **^{13}C NMR (101 MHz, C_6D_6)**: δ 162.9 (d, J = 247.1 Hz), 147.8, 143.1, 133.4 (d, *J* = 2.4 Hz), 131.4 (d, *J* = 2.9 Hz), 128.5, 126.8 (d, *J* = 8.2 Hz), 126.2, 126.1, 115.5 (d, *J* = 21.7 Hz), 46.1, 44.4, 27.8 ppm; **^{19}F NMR (376 MHz, C_6D_6)**: δ -113.26 (tt, *J* = 8.7, 5.4 Hz) ppm; $\boldsymbol{R}_f$: 0.69 (Hexane); $[\boldsymbol{\alpha}]_{\mathbf{D}}^{\mathbf{20}}$: +63.6 (C=0.73, DCM); HPLC analysis: Chiralpak IB, 4.6 x 250 mm; 2.0:98.0 DCM/hexane, 1.0 mL/min, λ = 254 nm; τmajor= 6.6 min, τminor= 7.2 min, 73% ee.

Compound **2z** was previously reported.[13]

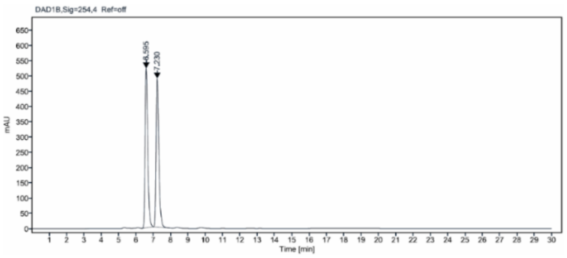

Signal: DAD1B,Sig=254,4 Ref=off

RT [min]	Width [min]	Area	Height	Area%
6.595	0.6794	5481.4598	522.7467	50.0283
7.230	1.0118	5475.2554	487.0422	49.9717

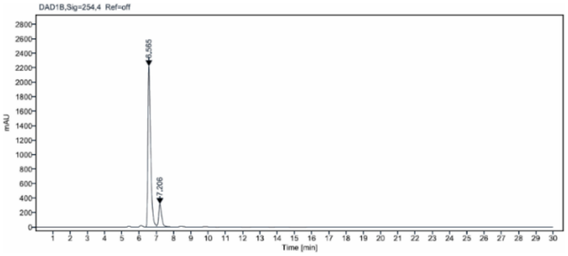

Signal: DAD1B,Sig=254,4 Ref=off

RT [min]	Width [min]	Area	Height	Area%
6.565	0.6679	24365.6100	2221.2112	86.5459
7.206	1.1445	3787.7991	316.4942	13.4541

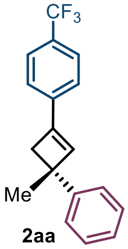

(*R*)-1-(3-methyl-3-phenylcyclobut-1-en-1-yl)-4-(trifluoromethyl)benzene (2aa). Following the General Procedure E. 3-methyl-3-phenyl-1-(4-(trifluoromethyl)phenyl)cyclobutyl 2,2,2-trichloroacetimidate **1ah** (40.0 mg, 0.100 mmol, 1 equiv. *trans/cis* 1.2:1), cat. **C4** (3.6 mg, 5 µmol, 0.05 equiv.), mesitylene (1 ml), reaction time 24 h. The crude mixture was purified by column chromatography (EtOAc-hexane, 1-6%) to afford the product as a white solid in 52% yield, with a 87% ee (15.0 mg, 0.052 mmol).

Employing (*S*)-**C5** (0.05 equiv): 20%, -19% ee.

^{1}H NMR (400 MHz, C_6D_6): δ 7.28 (d, *J* = 8.8 Hz, 2H), 7.22 – 7.13 (m, 4H), 7.08 – 7.02 (m, 1H), 6.95 (d, *J* = 8.1 Hz, 2H), 6.39 (s, 1H), 2.66 (d, *J* = 12.5 Hz, 1H), 2.51 (d, *J* = 12.5 Hz, 1H), 1.42 (s, 3H) ppm; **^{13}C NMR (101 MHz, C_6D_6)**: δ 147.0, 142.5, 137.8, 136.6, 129.5, 128.3, 127.6, 126.0, 125.9, 125.2 (q, *J* = 3.6 Hz), 124.9, 46.1, 43.8, 27.3; **^{19}F NMR (376 MHz, C_6D_6)**: δ -62.01 ppm; ***R*f**: 0.56 (Hexane); $[\alpha]_D^{20}$: -62.1 (c = 1.00, $CHCl_3$); HPLC analysis: Chiralpak AD-H, 4.6 x 250 mm; 0.2:99.8 i-PrOH/hexane, 1.0 mL/min, λ = 254 nm; τmajor = 5.4 min, τminor= 4.7 min, 87% ee.

Compound **2aa** was previously reported.[13]

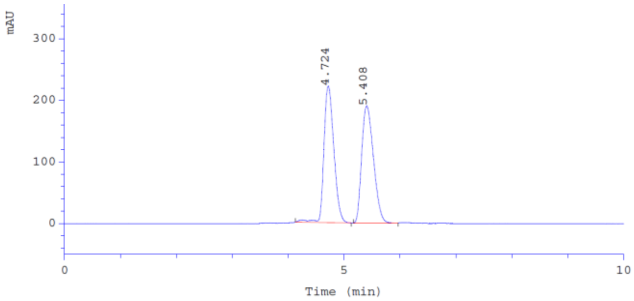

	Ret.time [min]	Start [min]	End [min]	Amount	Area [mAU*min]	Height [mAU]	% Area
1	4.724	4.13	5.13	-1	45.3548	221.518	49.4544
2	5.408	5.17	5.97	-1	46.3556	190.247	50.5456

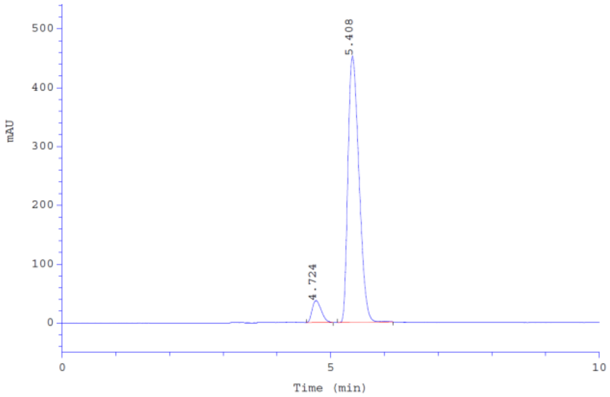

	Ret.time [min]	Start [min]	End [min]	Amount	Area [mAU*min]	Height [mAU]	% Area
1	4.724	4.55	5.05	-1	7.31422	37.4365	6.3581
2	5.408	5.13	6.16	-1	107.723	452.821	93.6419

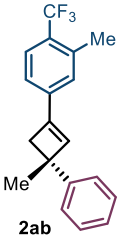

2ab

(*R*)-2-methyl-4-(3-methyl-3-phenylcyclobut-1-en-1-yl)-1-(trifluoromethyl)benzene (**2ab**). Following the General Procedure E. 3-methyl-1-(3-methyl-4-(trifluoromethyl)phenyl)-3-phenylcyclobutyl 2,2,2-trichloroacetimidate **1ai** (46.5 mg, 0.100 mmol, 1 equiv., *trans/cis* 1.1:1), cat. **C4** (3.6 mg, 5 µmol, 0.05 equiv.), mesitylene (1 ml), reaction time 24 h. The crude mixture was purified by column chromatography (EtOAc-hexane 1-6%) to afford the product as a colorless gel in 69% yield, 64% ee (21.0 mg, 0.069 mmol).

Employing (*S*)-**C5** (0.05 equiv): 66%, -17% ee.

^{1}H NMR (400 MHz, C_6D_6): δ 7.37 (d, *J* = 8.1 Hz, 1H), 7.24 – 7.13 (m, 4H), 7.05 (t, *J* = 7.1 Hz, 1H), 6.91 (s, 1H), 6.85 (d, *J* = 8.1 Hz, 1H), 6.42 (s, 1H), 2.70 (d, *J* = 12.5 Hz, 1H), 2.56 (d, *J* = 12.5 Hz, 1H), 2.21 (s, 3H), 1.45 (s, 3H) ppm; **^{13}C NMR (101 MHz, C_6D_6)**: δ 147.1, 142.6, 137.7, 136.6, 136.5, 128.3, 128.1, 127.6,, 126.5, 125.9, 125.9 (q, *J* = 7.8 Hz), 125.8, 122.0, 46.2, 43.9, 27.3, 18.9 ppm; **^{19}F NMR (376 MHz, C_6D_6)**: δ -60.86 ppm; **IR (ATR)**; 3026, 2957, 1726, 1602, 1566, 1494, 1445, 1313, 1264, 1167, 1111, 1042, 947, 887, 863, 835, 808, 760, 740, 697, 669, 621, 559, 543, 455 cm^{-1}; **HRMS (ESI)**: calcd. for $[C_{19}H_{17}F_3+H]^+$, $[M+H]^+$: 303.1355; found: 303.1358; ***R*f**: 0.54 (Hexane); $[\alpha]_D^{20}$: -40.55 (c = 1.00, $CHCl_3$); HPLC analysis: Chiralpak IB, 4.6 x 250 mm; 0.5:99.5 DCM/hexane, 1.0 mL/min, λ = 254 nm; τmajor = 7.56 min, τminor= 6.97 min, 64% ee.

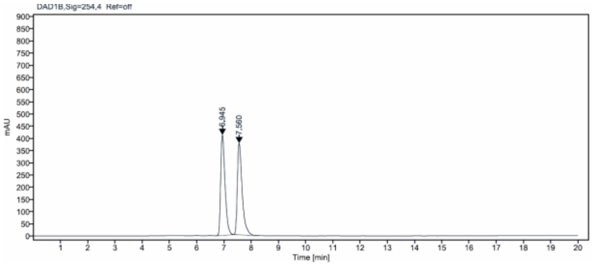

Signal: DAD1B,Sig=254,4 Ref=off

RT [min]	Width [min]	Area	Height	Area%
6.945	0.6538	4535.5394	411.8159	49.5077
7.560	0.9888	4625.7432	375.8745	50.4923

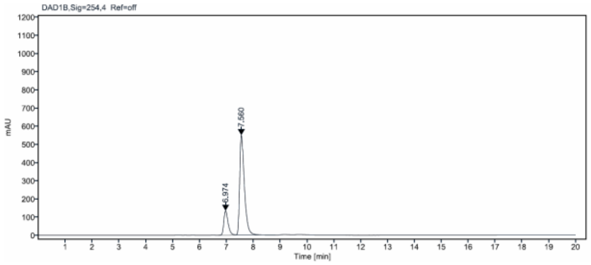

Signal: DAD1B,Sig=254,4 Ref=off

RT [min]	Width [min]	Area	Height	Area%
6.974	0.6305	1478.5406	133.9702	18.0237
7.560	1.4695	6724.7542	549.8763	81.9763

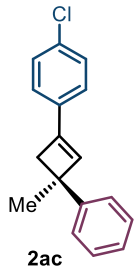

2ac

(*S*)-1-Chloro-4-(3-methyl-3-phenylcyclobut-1-en-1-yl)benzene (2ac). Following the General Procedure **E**. 1-(4-chlorophenyl)-3-methyl-3-phenylcyclobutyl 2,2,2-trichloroacetimidate **1aj** (41.7 mg, 0.100 mmol, 1 equiv., *cis/trans* 1.2:1), cat. **C5** (4.2 mg, 5 µmol, 0.05 equiv.), mesitylene (1 ml), reaction time 24 h. The crude mixture was purified by column chromatography on Al_2O_3 Brockman activity grade V (hexane, 100%) to afford the product as a colorless oil in 59% yield and 63% ee (15.1 mg, 0.059 mmol).

^{1}H NMR (400 MHz, C_6D_6): δ 7.29 – 7.19 (m, 4H), 7.14 – 7.07 (m, 3H), 6.97 – 6.92 (m, 2H), 6.40 (s, 1H), 2.73 (d, J = 12.5 Hz, 1H), 2.59 (d, J = 12.5 Hz, 1H), 1.50 (s, 3H) ppm; **^{13}C NMR (101 MHz, C_6D_6)**: δ 147.6, 143.0, 134.7, 133.8, 133.5, 128.8, 128.5, 126.4, 126.2, 126.1, 46.2, 44.2, 27.7 ppm; ***R*f**: 0.71 (Hexane); $[\alpha]_D^{20}$: +49.7 (C=1.00, DCM); HPLC analysis: Chiralpak IB, 4.6 x 250 mm; 2.0:98.0 DCM/hexane, 1.0 mL/min, λ = 254 nm; τmajor= 8.5 min, τminor= 9.3 min, 63% ee.

Compound **2ac** was previously reported.[13]

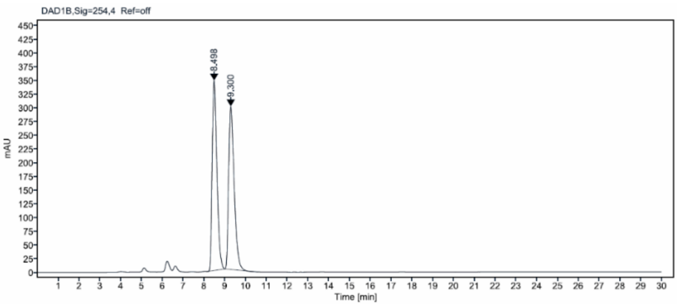

Signal: DAD1B,Sig=254,4 Ref=off

RT [min]	Width [min]	Area	Height	Area%
8.498	0.8921	5473.6343	345.9357	49.8378
9.300	1.6121	5509.2657	297.2038	50.1622

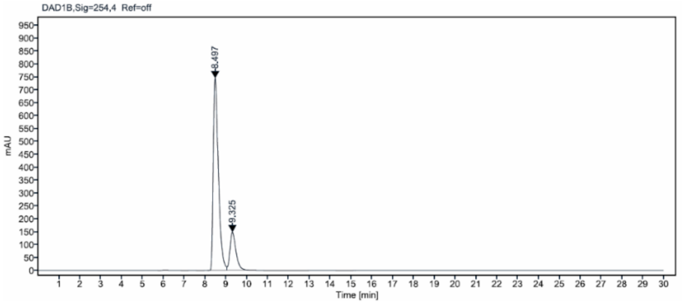

Signal: DAD1B,Sig=254,4 Ref=off

RT [min]	Width [min]	Area	Height	Area%
8.497	0.9383	12700.0403	744.3611	81.4056
9.325	1.0065	2900.8930	147.1818	18.5944

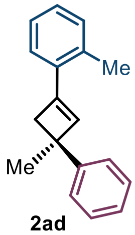

2ad

(*S*)-1-methyl-2-(3-methyl-3-phenylcyclobut-1-en-1-yl)benzene (2ad). Following the General Procedure E. 3-methyl-3-phenyl-1-(o-tolyl)cyclobutyl 2,2,2-trichloroacetimidate **1ak** (40.0 mg, 0.100 mmol, 1 equiv., *cis/trans* 1.4:1), cat. **C5** (4.2 mg, 5 µmol, 0.05 equiv.), mesitylene (1 ml), reaction time 24 h. The crude mixture was purified by column chromatography (EtOAc-hexane, 1-6%) to afford the product as a white solid in 80% yield, with an 66% ee (19.0 mg, 0.81 mmol).

Employing (*S*)-**C4** (0.05 equiv): 30%, -18% ee

^{1}H NMR (400 MHz, C_6D_6): δ 7.27 (d, *J* = 7.0 Hz, 2H), 7.17 (t, *J* = 7.7 Hz, 2H), 7.08 – 6.92 (m, 5H), 6.34 (s, 1H), 2.90 (d, *J* = 12.4 Hz, 1H), 2.75 (d, *J* = 12.4 Hz, 1H), 2.21 (s, 3H), 1.49 (s, 3H) ppm; **^{13}C NMR (101 MHz, C_6D_6)**: δ 147.7, 143.8, 137.7, 136.9, 133.4, 130.5, 128.2, 127.7, 126.8, 126.0, 125.8, 125.7, 45.9, 45.8, 27.6, 21.78; ***R_f***: 0.65 (Hexane); $[\alpha]_D^{20}$: +47.9 (c = 1.00, $CHCl_3$); HPLC analysis: Chiralpak IF, 4.6 x 250 mm; 0.1:99.9 DCM/hexane, 1.0 mL/min, λ = 254 nm; τmajor = 11.9 min, τminor = 10.3 min, 66% ee.

Compound **2ad** was previously reported.[13]

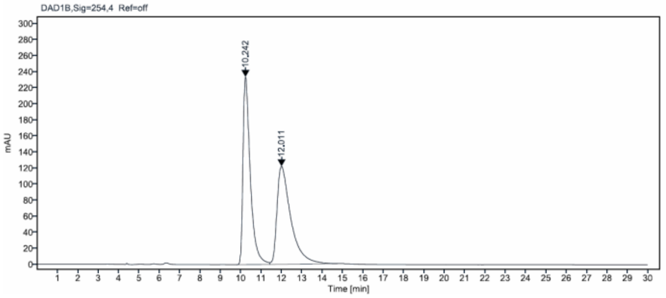

Signal: DAD1B,Sig=254,4 Ref=off

RT [min]	Width [min]	Area	Height	Area%
10.242	1.6379	5815.7861	233.8845	50.3399
12.011	3.3321	5737.2584	122.0305	49.6601

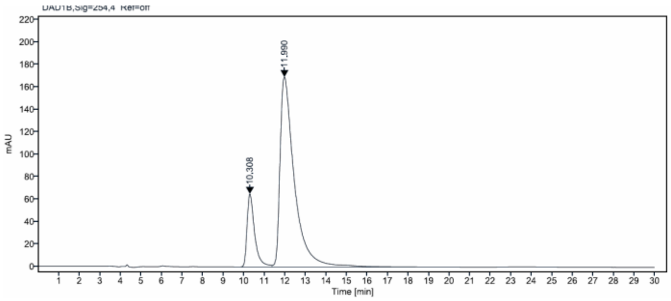

Signal: DAD1B,Sig=254,4 Ref=off

RT [min]	Width [min]	Area	Height	Area%
10.308	1.5944	1666.0085	65.1006	16.8520
11.990	6.9756	8220.1336	169.2229	83.1480

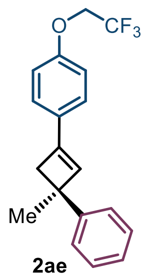

2ae

(*S*)-1-(3-methyl-3-phenylcyclobut-1-en-1-yl)-4-(2,2,2-trifluoroethoxy)benzene (2ae). Following the General Procedure D. 3-methyl-3-phenyl-1-(4-(2,2,2-trifluoroethoxy)phenyl)cyclobutan-1-ol **1ae** (33.6 mg, 0.100 mmol, 1 equiv., cis/trans 2.1:1), cat. **C5** (16.9 mg, 20 µmol, 0.2 equiv.), mesitylene (1 ml), reaction time 48 h. The crude mixture was purified by column chromatography (EtOAc-hexane, 0-8%) to afford the product as a white gel in 53% yield and 90% ee (17.0 mg, 0.053 mmol).

With **1al** (48.1 mg, 0.100 mmol, 1 equiv., *cis/trans* 1.4:1) and (*S*)-**C5** (0.05 equiv): 82%, 49% ee. With **1al** (48.1 mg, 0.100 mmol, 1 equiv., *cis/trans* 1.4:1) and (*S*)-**C4** (0.05 equiv): traces of product were formed.

^{1}H NMR (400 MHz, C_6D_6): δ 7.29 – 7.24 (m, 2H), 7.21 – 7.15 (m, 2H), 7.09 – 7.02 (m, 3H), 6.50 – 6.45 (m, 2H), 6.40 (s, 1H), 3.51 (q, *J* = 8.2 Hz, 2H), 2.81 (d, *J* = 12.5 Hz, 1H), 2.67 (d, *J* = 12.4 Hz, 1H), 1.50 (s, 3H) ppm; **^{13}C NMR (101 MHz, C_6D_6)**: δ 157.0, 147.7, 143.0, 132.3, 129.6, 128.2, 126.2, 126.0, 125.8, 123.7 (d, *J* = 277.9 Hz), 114.6, 65.1 (q, *J* = 35.2 Hz), 45.8, 44.1, 27.6 ppm; **^{19}FNMR** (376 MHz, **C_6D_6**) δ -73.67 (t, *J* = 8.2 Hz) ppm; **IR (ATR)**: 3057, 3028, 2956, 2919, 2864, 1623, 1601, 1580, 1540, 1506, 1495, 1458, 1445, 1427, 1370, 1319, 1284, 1237, 1107, 1095, 1078, 1028, 1010, 975, 945, 906, 863, 836, 801, 762, 718, 700, 674, 618, 534, 499, 453, 419 cm^{-1}; $[\alpha]_D^{20}$: +52.1 (c = 1.00, CH_2Cl_2); **HRMS (ESI)**: calcd. for $[C_{18}H_{17}F_3O+H]^+$, $[M+H]^+$: 319.1304; found: 319.1301; ***R*f**: 0.57 (Hexane); **HPLC analysis**: Chiralpak IB, 4.6 x 250 mm; 2:98 DCM/hexane, 1.0 mL/min, λ = 254 nm; τmajor= 15.1 min, τminor= 19.0 min, 90% ee.

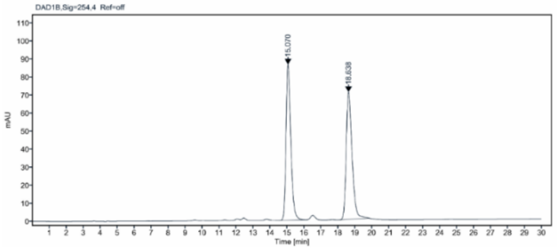

Signal: DAD1B,Sig=254,4 Ref=off

RT [min]	Width [min]	Area	Height	Area%
15.070	1.6683	1683.0320	86.2587	49.9033
18.638	1.7750	1689.5559	70.4918	50.0967

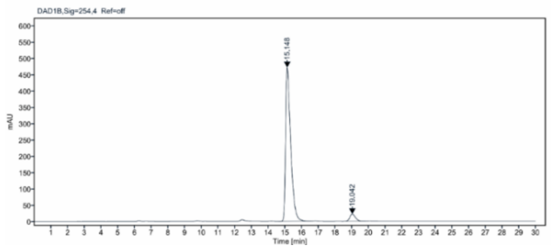

Signal: DAD1B,Sig=254,4 Ref=off

RT [min]	Width [min]	Area	Height	Area%
15.148	1.9367	9988.3160	471.6340	95.0182
19.042	1.3053	523.6831	22.2675	4.9818

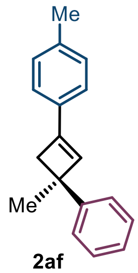

2af

(*S*)-1-methyl-2-(3-methyl-3-phenylcyclobut-1-en-1-yl)benzene (2af). Following the General Procedure E. 3-methyl-3-phenyl-1-(*p*-tolyl)cyclobutyl 2,2,2-trichloroacetimidate **1am** (24.0 mg, 60 µmol, 1 equiv., *cis/trans* 1.6:1), cat. **C5** (2.6 mg, 3 µmol, 0.05 equiv.), mesitylene (0.6 ml), reaction time 24 h. The crude mixture was purified by column chromatography (EtOAc-hexane, 1-6%) to afford the product as a pale yellow oil in 78% yield, with an 59% ee (11.0 mg, 47 µmol).

^{1}H NMR (400 MHz, C_6D_6): δ 7.29 – 7.23 (m, 2H), 7.20 (d, *J* = 8.1 Hz, 2H), 7.19 – 7.12 (m, 2H), 7.06 – 7.00 (m, 2H), 6.94 (d, *J* = 7.5 Hz, 2H), 6.45 (s, 1H), 2.83 (d, *J* = 12.5 Hz, 1H), 2.69 (d, *J* = 12.4 Hz, 1H), 2.06 (s, 3H), 1.49 (s, 3H) ppm; **^{13}C NMR (101 MHz, C_6D_6)**: δ 147.9, 144., 137.5, 132.6, 132.4, 129.1, 128.3, 126.1, 125.8, 124.9, 45.9, 44.3, 27.7, 21.2 ppm; ***R*f**: 0.64 (Hexane); $[\boldsymbol{\alpha}]_{\mathbf{D}}^{\mathbf{20}}$: +135.4 (c = 1.00, $CHCl_3$); HPLC analysis: Chiralpak IB, 4.6 x 250 mm; 0.5:99.5 DCM/hexane, 1.0 mL/min, λ = 254 nm; τmajor = 7.5 min, τminor = 8.5 min, 59% ee. Compound **2af** was previously reported.[12]

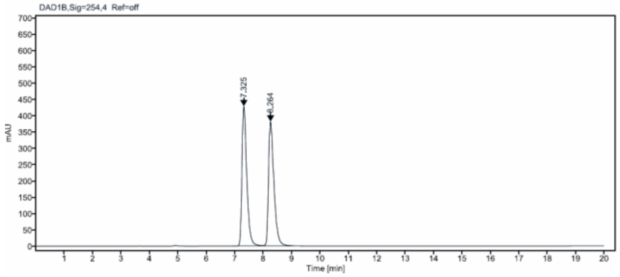

Signal: DAD1B,Sig=254,4 Ref=off

RT [min]	Width [min]	Area	Height	Area%
7.325	1.0984	5061.1265	428.5408	49.9759
8.264	1.4216	5066.0179	381.5821	50.0241

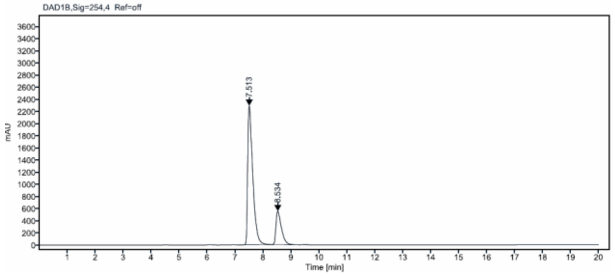

Signal: DAD1B,Sig=254,4 Ref=off

RT [min]	Width [min]	Area	Height	Area%
7.513	1.1444	28870.0110	2289.3263	79.3997
8.534	1.4329	7490.3606	548.8504	20.6003

Unsuccessful and moderately successful substrates

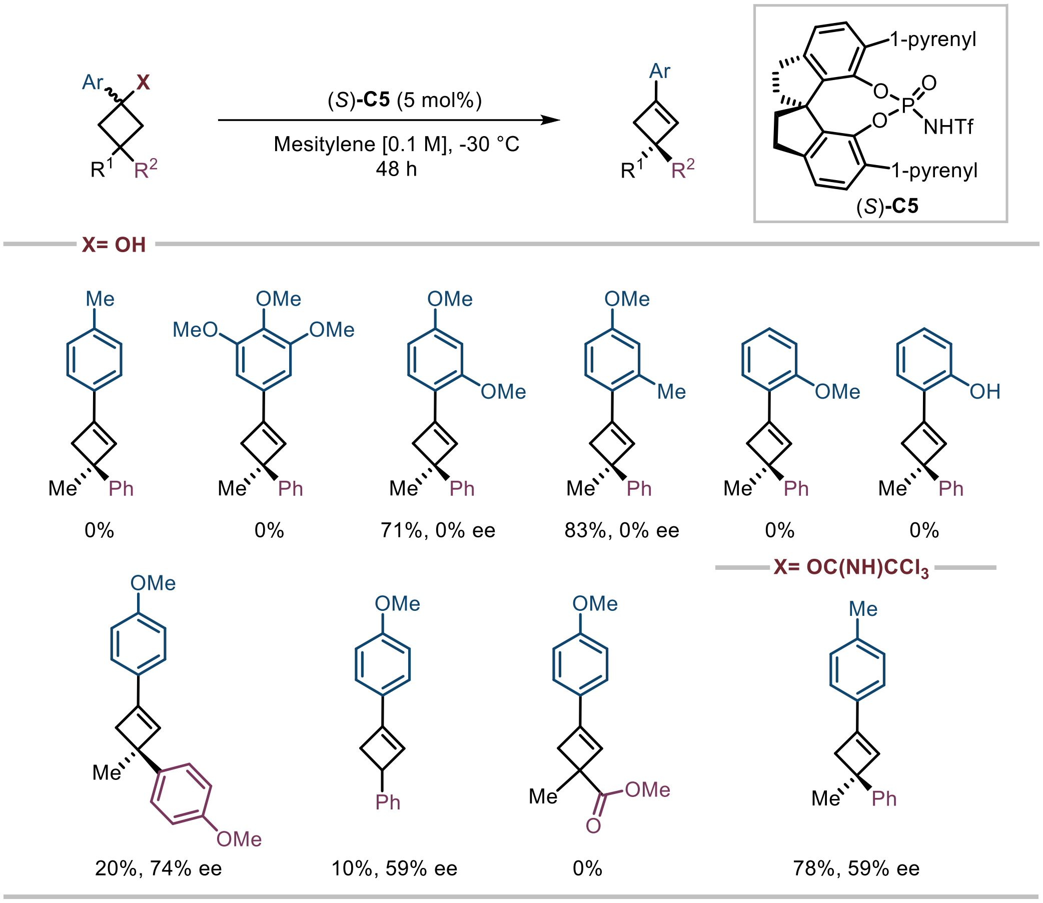

Reaction scale-up and post-functionalizations

Reaction scale-up

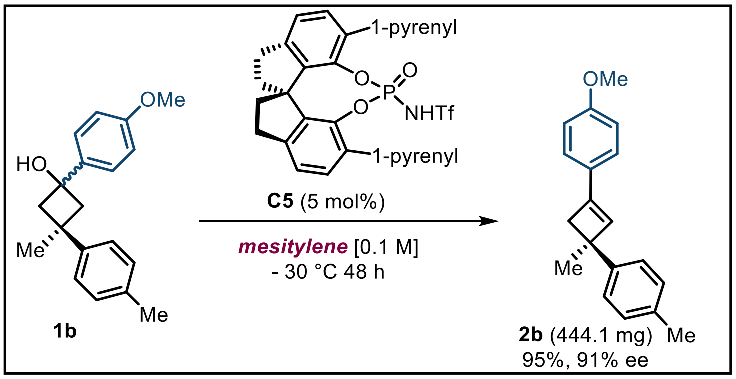

A flame-dried 100 ml Schlenk flask equipped with a magnetic stirring bar was charged with substrate (500 mg, 1.77 mmol, 1 equiv.). The flask was evacuated and backfilled with argon 3 times. Next, 12.7 ml mesitylene was added, and the mixture was stirred at -30 °C for 60 min. Next, the catalyst (74.9 mg, 0.089 mmol, 0.05 equiv.), in mesitylene (5.0 ml), was then added

slowly over 30 min (by using a syringe pump) at -30 °C. The mixture was stirred (700 rpm) at -30 °C for 48 h. After this, the reaction was quenched by the addition of Et_3N (1.8 ml), and the mixture was purified by column chromatography (EtOAc-hexane, 3%) to afford the expected product with 95% yield and 91% ee (444 mg, 1.68 mmol).

Post-Functionalizations

Procedure for the oxidative ring contraction of cyclobutene 2b[14]

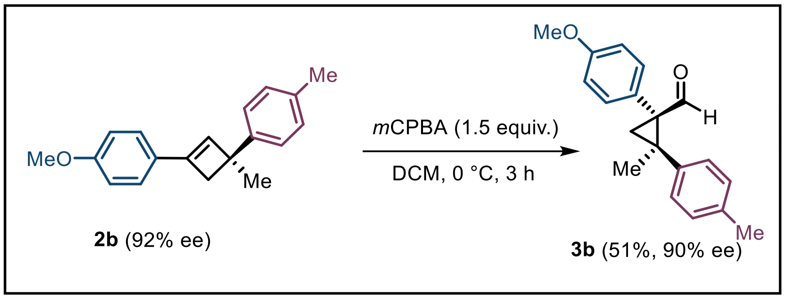

An oven-dried 8 ml vial equipped with a magnetic stirring bar was charged with (*S*)-1-methoxy-4-(3-methyl-3-(*p*-tolyl)cyclobut-1-en-1-yl)benzene **2b** (24.3 mg, 0.092 mmol, 1 equiv., 92% ee). The vial was evacuated and backfilled with argon 3 times. Next, 0.62 ml of dry DCM was added, and the mixture was stirred at 0 °C for 20 min. Subsequently, 3-chloroperbenzoic acid (freshly purified) (23.8 mg, 0.138 mmol, 1.5 equiv.) was added in 0.3 ml of dry DCM at 0 °C. The reaction mixture was stirred at 0 °C for 3 h. Then the reaction mixture was allowed to warm to room temperature and quenched by the addition of 1 M NaOH solution, followed by extraction with DCM (three times). The combined organic layers were dried over Na_2SO_4. Concentration under reduced pressure after filtration gave the crude product, which was subjected to silica gel column chromatography (ethyl acetate-hexane 10%) to give the major diastereoisomer of cyclopropyl aldehyde **3b** as a colorless oil in 51% yield, 90% ee (10:1 dr was determined from a crude sample) (13.1 mg, 46.7 μmol).

(1*S*,2*R*)-1-(4-Methoxyphenyl)-2-methyl-2-(*p*-tolyl)cyclopropane-1-carbaldehyde (3b). ^{1}H NMR (400 MHz, $CDCl_3$) δ 8.73 (s, 1H), 7.30 – 7.22 (m, 4H), 7.15 (d, J = 7.8 Hz, 2H), 6.96 (d, J = 8.3 Hz, 2H), 3.84 (s, 3H), 2.33 (s, 4H), 1.75 (d, J = 5.3 Hz, 1H), 1.13 (s, 3H) ppm; **^{13}C NMR (101 MHz, $CDCl_3$)** δ 200.8, 159.1, 138.9, 136.8, 131.9, 129.6, 128.8, 127.0, 114.1, 55.4, 47.3, 38.3, 26.3, 24.4, 21.2 ppm; **IR (ATR)**: 2956, 2921, 2836, 2727, 2360, 1705, 1611, 1577, 1515, 1457, 1376, 1288, 1247, 1177, 1131, 1110, 1072, 1030, 916, 893, 820, 750, 668, 598, 536 cm^{-1}; **HRMS (ESI)**: calcd. for $[C_{19}H_{20}O_2+Na]^+$, $[M+Na]^+$: 303.1361; found: 303.1359; ***R*f**: 0.19 (Hexane/EtOAc, 9:1); $[\alpha]_D^{20}$: +227.6 (c = 0.69, $CHCl_3$); HPLC analysis: Chiralpak IA, 4.6 x 250 mm; 5.0:95.0 i-PrOH/hexane, 1.0 mL/min, λ = 230 nm; τmajor= 12.9 min, τminor= 9.9 min, 90% ee.

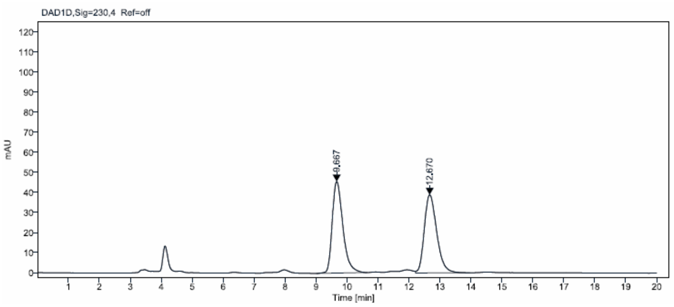

Signal:	DAD1D,Sig=230,4 Ref=off			
RT [min]	Width [min]	Area	Height	Area%
9,667	1,6361	1092,1230	45,5907	50,1571
12,670	1,7465	1085,2830	38,9288	49,8429

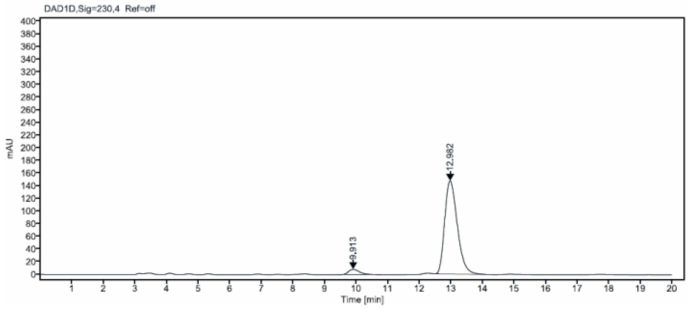

Signal:	DAD1D,Sig=230,4 Ref=off			
RT [min]	Width [min]	Area	Height	Area%
9.913	1.3643	207.2310	8.4433	4.8289
12.982	1.7648	4084.2757	147.1657	95.1711

Procedure for the oxidative ring cleavage of cyclobutene 2a[15]

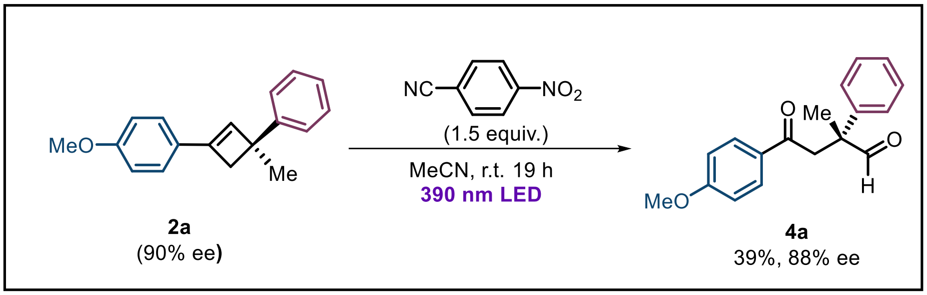

A 4 ml vial equipped with a stirring bar was charged with 4-nitrobenzonitrile (22 mg, 0.15 mmol, 1.5 equiv.) and cyclobutene **2b** (25 mg, 0.1 mmol, 1 equiv., 90% ee). The vial was evacuated and backfilled with argon 3 times. Next, 1 ml of anhydrous MeCN was added, and the mixture was degassed by bubbling argon for 15 minutes. The reaction vial was placed 3 cm in front of a 390 nm lamp, and the reaction was stirred under irradiation for 19 h. Then, the solvent was removed

in vacuo, and the crude mixture was purified by column chromatography (EtOAc-hexane 5-15%) to afford the product as a yellow solid in 39% yield (11 mg, 0.039 mmol, 88% ee).

(*R*)-4-(4-methoxyphenyl)-2-methyl-4-oxo-2-phenylbutanal (4a): **^{1}H NMR (400 MHz, $CDCl_3$)**: δ 9.74 (s, 1H), 7.91 (d, *J* = 8.7 Hz, 2H), 7.40 – 7.29 (m, 5H), 6.95 – 6.88 (m, 2H), 3.86 (s, 3H), 3.70 – 3.66 (m, 2H), 1.64 (s, 3H) ppm; **^{13}C NMR (101 MHz, $CDCl_3$)**: δ 201.77, 195.85, 163.81, 140.05, 130.56, 129.94, 128.99, 127.44, 126.98, 113.86, 55.60, 51.91, 45.87, 21.01 ppm; ***R*f**: 0.22 (Hexane/EtOAc, 85:15); $[\boldsymbol{\alpha}]_{\mathbf{D}}^{\mathbf{20}}$: -32.6 (c = 1, $CHCl_3$); **HPLC analysis**: Chiralpak IF, 4.6 x 250 mm; 20:80 i-PrOH/hexane, 1.0 mL/min, λ = 254 nm; τminor = 17.27 min, τmajor= 20.12 min, 88% ee.

Spectral data match the ones reported in the literature.[16]

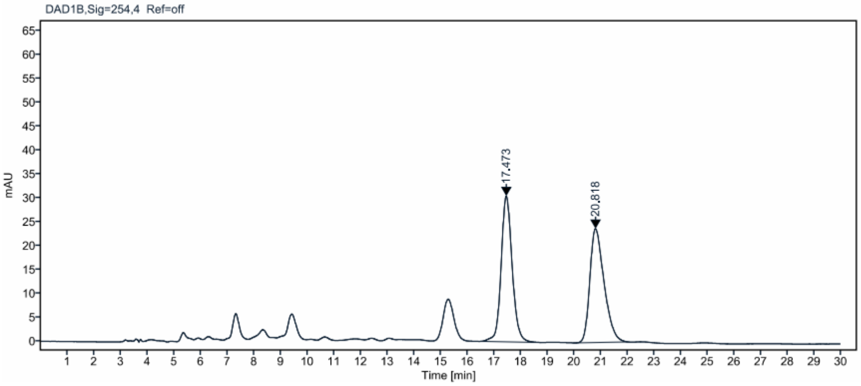

Signal: DAD1B,Sig=254,4 Ref=off

RT [min]	Width [min]	Area	Height	Area%
17.473	2.2781	879.4474	30.5403	49.9125
20.818	2.0517	882.5321	23.8280	50.0875

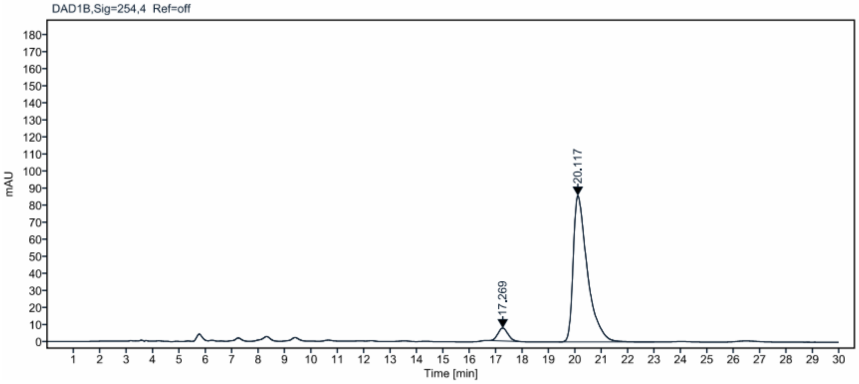

Signal: DAD1B,Sig=254,4 Ref=off

RT [min]	Width [min]	Area	Height	Area%
17.269	1.3552	202.3517	7.5951	5.9334
20.117	3.6138	3208.0055	85.7891	94.0666

Mechanistic studies

^{1}H NMR Reaction monitoring.

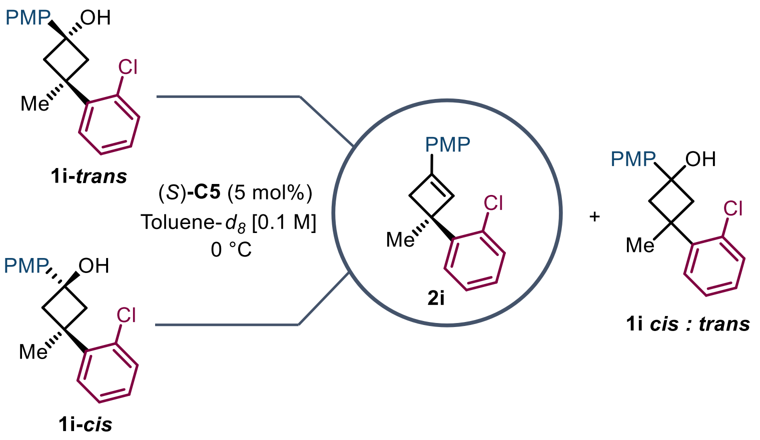

Figure S2. Elimination of diastereomerically pure substrates.

General NMR data processing: NMR data were imported into MNOVA 14.2.3 using the reaction-monitoring plugin and processed therein. After phase and baseline correction, NMR concentration profiles were generated using the olefinic signal at 6.53 ppm (1 × H) of the cyclobutene (**2i**) and the methyl groups (3 × H) of the diastereomeric cyclobutanols at 1.88 ppm (**1i-*trans***) and 1.19 ppm (**1i-*cis***). All concentration values were referenced to the first acquired ^{1}H NMR spectrum.

Sample preparation and data acquisition

An oven-dried 4 ml vial was charged with the diastereoisomerically pure substrate (**1i-*trans*** or **1i-*cis***). The vial was evacuated and backfilled with argon three times. Next, 0.7 ml of toluene-d_8 was added. Then, 0.6 ml of this solution was transferred to a screw-cap NMR tube, which was subsequently placed in the NMR spectrometer at 0 °C. After shimming, a ^{1}H NMR spectrum was acquired. Following a 20-minute temperature-equilibration period, a catalyst solution (5 mol% in 260 µl of toluene-d_8) was added to the NMR tube containing the reaction mixture. After fast shimming, ^{1}H NMR spectra were acquired every 20 minutes until the starting material was consumed to >80%.

The following Figures 1 & 2 show ^{1}H NMR spectra taken at different time points during the elimination reaction of diastereoisomerically pure cyclobutanols **1i** at 0 °C in toluene-d_8.

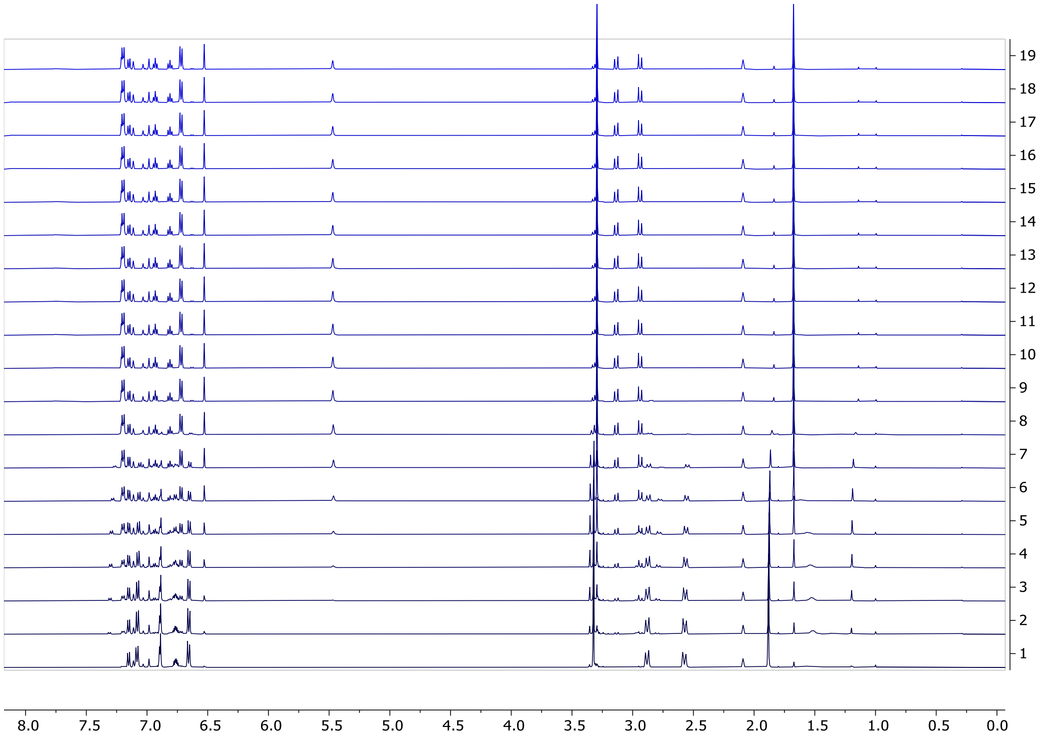

Figure S3. Stacked ^{1}H NMR spectra at different time points during the elimination reaction of **1i-*trans*** (0.1M in toluene-d_8) in the presence of catalyst (*S*)-**C5** (5 mol%).

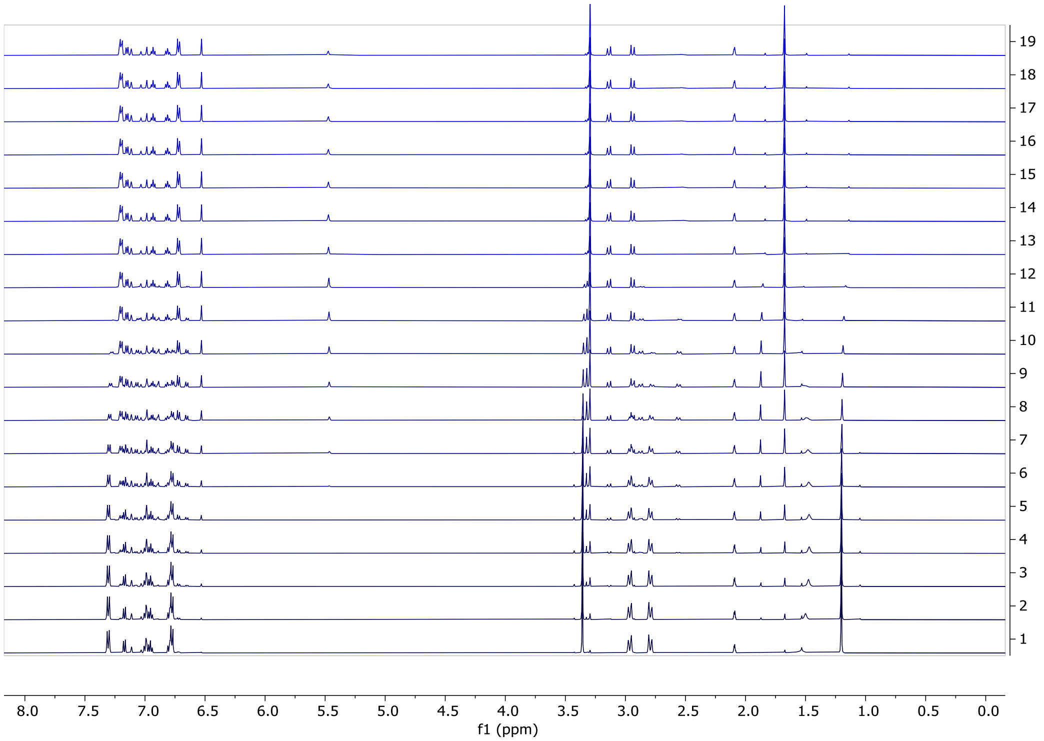

Figure S4. Stacked ^{1}H NMR spectra at different time points during the elimination reaction of **1i-*cis*** (0.1M in toluene-d_8) in the presence of catalyst (*S*)-**C5** (5 mol%).

Experiments with the isolated*cis* and *trans* substrates

Eliminations of separable, diastereomerically pure substrates were performed using cyclobutanols **1i** and **1j** bearing *ortho*-chloro- and *ortho*-methyl-substituted phenyl rings, respectively. While both diastereomers of **1i** afforded the product in similar yields, in the case of substrate **1j,** the cis isomer converted to product **2j** in lower yield than the corresponding trans isomer.

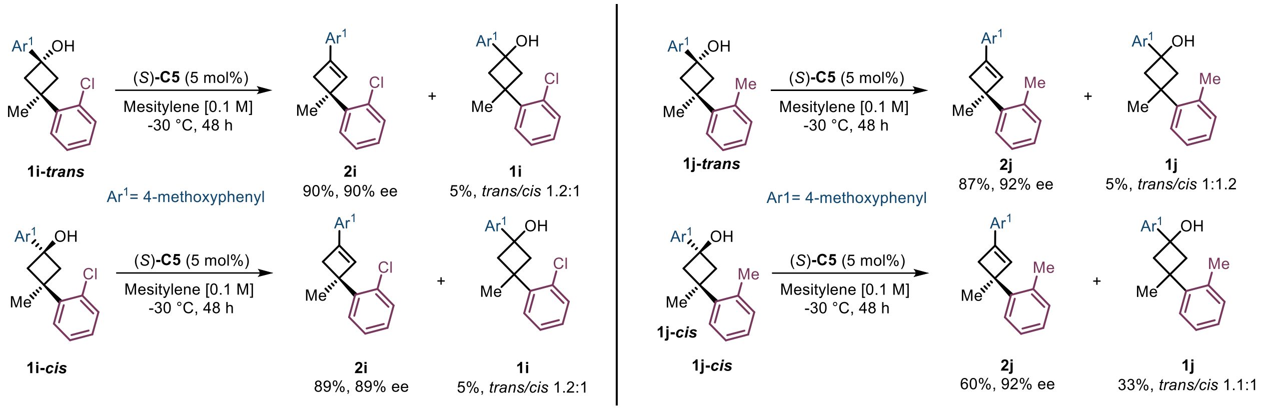

Figure S5. Elimination reactions of diastereomerically pure substrates.

The reaction profiles of **1i-*trans*** and **1i-*cis,*** examined separately, revealed slower consumption of the cis isomer, which is consistent with the results obtained for **1j**. On the other hand, the reaction profile obtained using an inseparable mixture of **1a-*cis*** and **1a-*trans*** indicated rapid consumption of **1a-*cis***, along with a temporary increase in the concentration of **1a-*trans***. Since the elimination of cyclobutanols proceeds primarily via an E2 mechanism, the reactivity of the *cis* and *trans* diastereoisomers may strongly depend on the substrate structure. *Ortho* substitution on the phenyl ring may significantly influence the steric environment around the reactive center, thereby hampering elimination from **1i-*cis*** and **1j-*cis***.

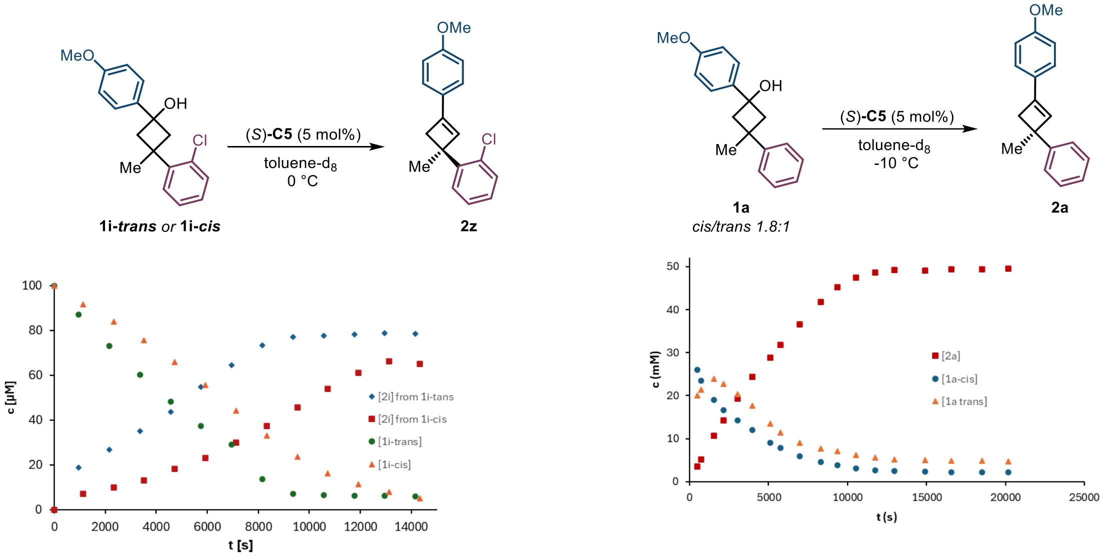

Figure S6. Reaction profile of elimination of **1i-*trans***, **1i-*cis,*** and the **1a** (*trans/cis* 1.8:1).

Reaction order determination

Elimination of cyclobutanol **1a** with catalyst **C4**

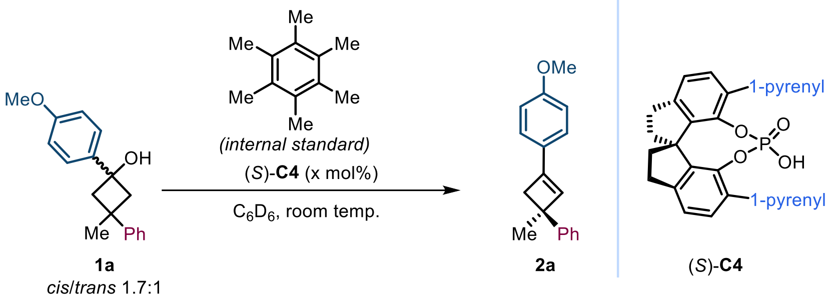

The reaction orders of substrates **1a** and catalyst (*S*)-**C4** were determined by variable time normalization analysis (VTNA).[17] Different concentrations of each component of interest were used to determine the respective order.

Order in the substrate.

An oven-dried NMR tube with a screw cap having a septum was charged with **1a** (0.06 mmol/ 0.03 mmol/ 0.015 mmol), 0.3 ml of C_6D_6, hexamethylbenzene (0.01 mmol) in 0.1 ml of C_6D_6, and (*S*)-**C4** (0.003 mmol) in 0.2 ml of C_6D_6. The NMR tube was then transferred to the NMR spectrometer, and after shimming, ^{1}H NMR spectra were acquired every 20-30 minutes until the starting material was > 60% consumed.

The following figure shows concentration profiles of **2a** using different initial concentrations of **1a** (0.1 M, 0.05M, 0.025M in C_6D_6) in the presence of catalyst (*S*)-**C4** with times scales normalized to different substrate orders (0th, 0.4th, 1st):

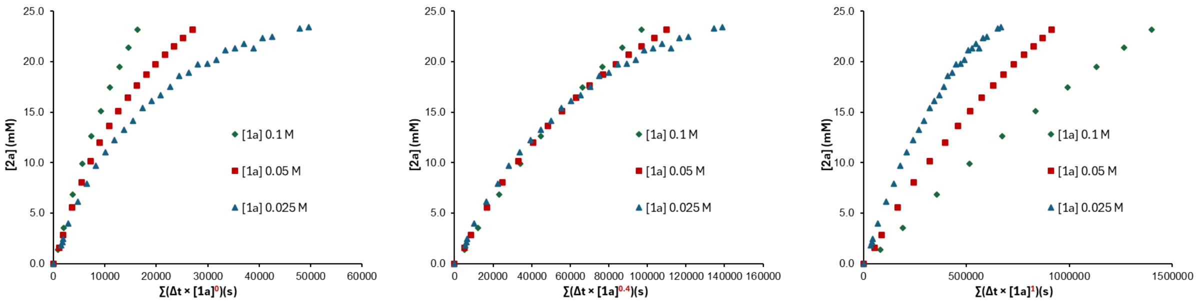

Figure S7. NMR concentration profiles with time scales normalized to different catalyst orders (left: zeroth order, middle: fractional order (0.4), right: first order).

The best overlap is observed assuming a fractional-order (0.4th) dependence on substrate concentration.

The following concentration profiles were obtained for using different initial concentrations of **1a** (0.1 M, 0.05M, 0.025 M):

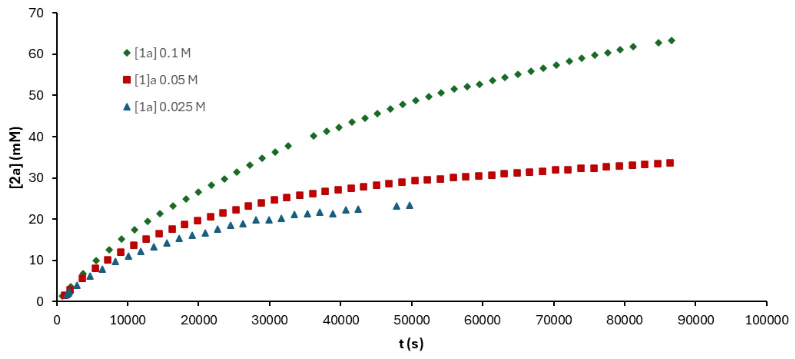

Figure S8. Concentration profiles obtained for different initial concentrations of **1a** in the presence of 5 mM catalyst (S)-**C4**.

The following concentration profiles of **2a**, **1a-*cis*,** and **1a-*trans*** were obtained for using **1a** (0.05M), in the presence of 5 mM catalyst (*S*)-**C4**.

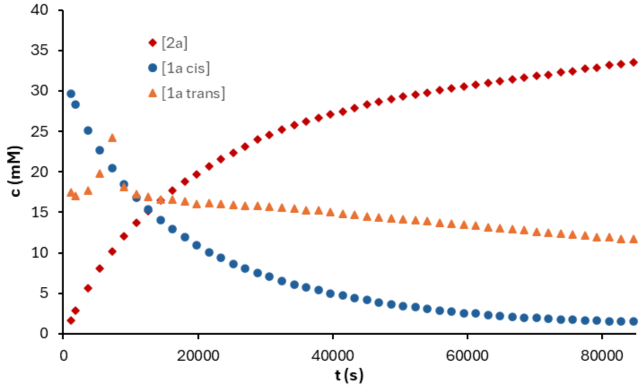

Figure S9. Concentration profiles obtained for reaction with **1a** (0.05M) in the presence of 5 mM catalyst (*S*)-**C4**. The profile indicates that **1a-*cis*** and **1a-*trans*** are consumed at different rates.

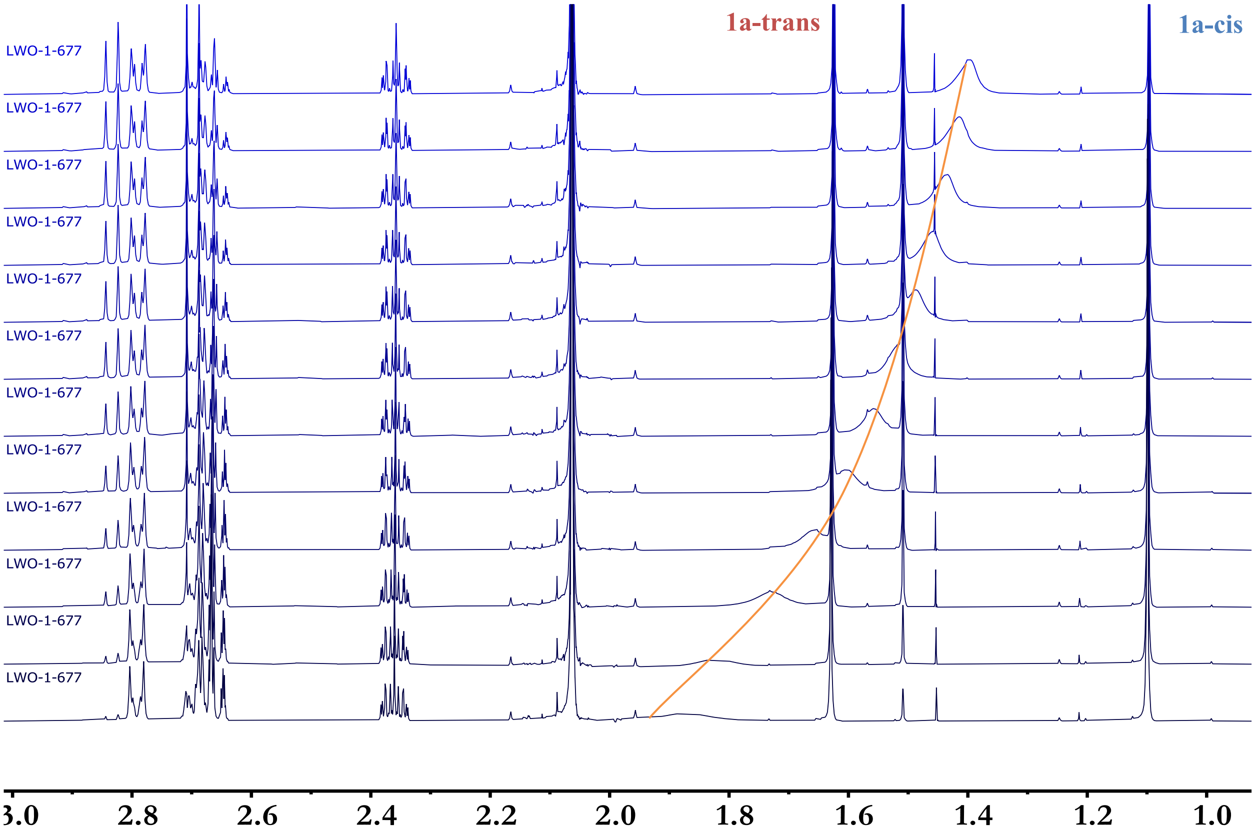

Figure S10. Stacked ^{1}H NMR spectra at different time points during the elimination reaction of **1a** of catalyst (*S*)-**C5** (5 mol%), which cocntration profile is presented on Figure S9.

Besides the decay of substrates **1a-*cis*** and **1a-*trans*** and the formation of product **2a**, a broad signal—attributed to an average OH species, possibly residual water—shifts from 1.9 to 1.2 over the course of the reaction. As a result, it appears as a "bump" in the concentration profile (Figure S9). Although the "OH bump" was not removed during the normalization process, it did not appear to significantly influence the VTNA outcome.

Order in the catalyst.

An oven dried NMR tube with a screw cup having a septum was charged with **1a** (0.03 mmol), 0.3 ml or 0.1 ml of C_6D_6, hexamethylbenzene (0.01 mmol) in 0.1 ml of C_6D_6 and (*S*)-**C4** (0.003 mmol) in 0.2 ml of C_6D_6 or (*S*)-**C4** (0.006 mmol) in 0.4 ml of C_6D_6. The NMR tube was then transferred to the NMR spectrometer, and after shimming, 1H NMR spectra were acquired every 20-30 minutes until the starting material was > 60% consumed.

The following figure shows concentration profiles of **2a** (initial concentration of **1a** 0.05M, in C_6D_6) in the presence of different amounts of catalyst (*S*)-**C4** with time scales normalized to different substrate orders (0th, 1st, 2nd):

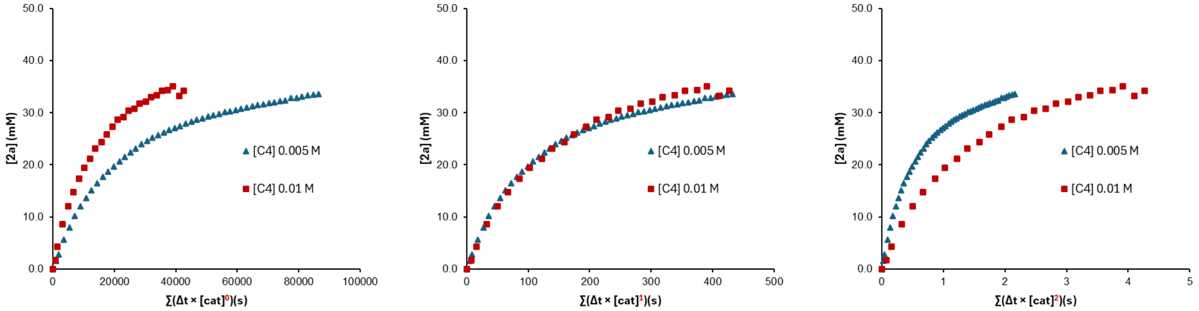

Figure S11. NMR concentration profiles with time scales normalized to different catalyst orders (left: zeroth order, middle: first order, right: second order). The best overlap is observed assuming a first-order dependence on catalyst concentration.

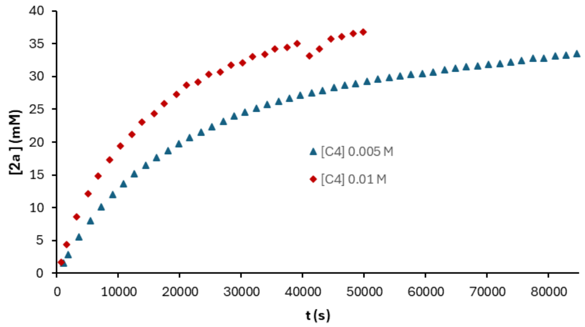

Figure S12. Concentration profiles obtained using different concentrations of the catalyst (*S*)-**C4**, with an initial concentration of **1a** of 0.05 M.

Elimination of cyclobutanol **1a** with catalyst **C5**

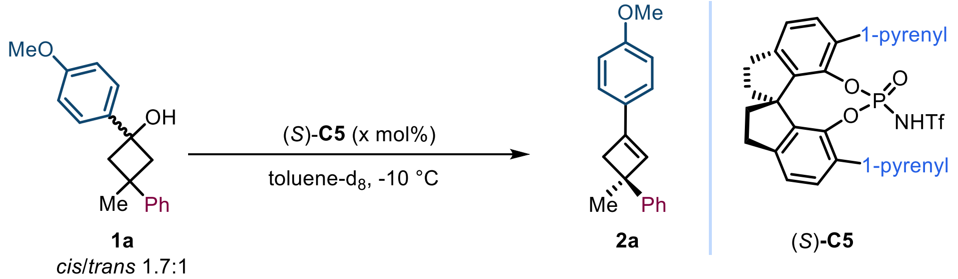

Order in the substrate.

An oven-dried NMR tube with a screw cap having a septum was charged with **1a** (0.045 mmol/ 0.03 mmol/ 0.015 mmol), 0.4 ml of toluene-d_8. The NMR tube was cooled down to -10°C, and (*S*)-**C5** (0.0025 mmol) in 0.2 ml of toluene-d_8 was added. After shimming, ^{1}H NMR spectra were acquired every 10-30 minutes until the starting material was > 80% consumed.

Due to the overlap between the signals of hexamethylbenzene and toluene, the analysis was performed without the internal standard. All concentration points were referenced to the first acquired ^1H NMR spectrum.

The following figure shows concentration profiles of **2a** using different initial concentrations of **1a** (0.05M, 0.025M in toluene-d_8) in the presence of catalyst (*S*)-**C5** with times scales normalized to different substrate orders (0th, 0.5^{th}, 1st):

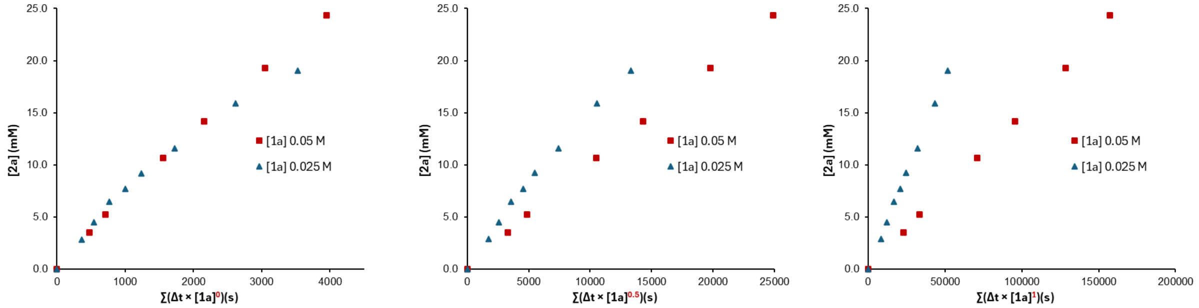

Figure S13. NMR concentration profiles with time scales normalized to different catalyst orders (left: zeroth order, middle: fractional order (0.5), right: first order).

The best overlap is observed assuming a zeroth-order dependence on substrate concentration.

The following concentration profiles were obtained for using different initial concentrations of **1a** (0.75 M, 0.05 M, 0.025 M). While zero-order dependence on **1a** was observed at low substrate initial concentrations (0.025 M and 0.05 M), the reaction rate significantly decreased at a higher initial concentration of **1a** (0.075 M), resulting in a negative order with respect to **1a** at higher substrate concentrations (0.05 M and 0.075 M). This can be attributed to the formation of hydrogen-bonded aggregates between **1a** molecules at higher concentrations in a nonpolar solvent.[18] Formation of off-cycle aggregates of **1a** could decrease the concentration of the reactive monomeric substrate, leading to orders lower than 1 or even negative with respect to substrate.

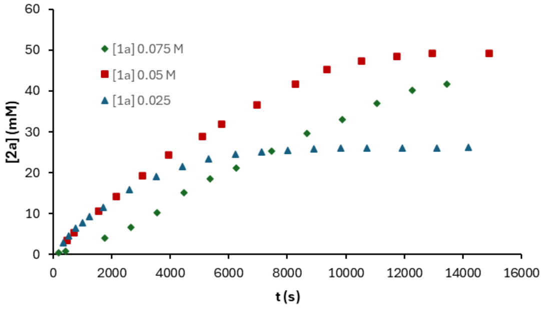

Figure S14. Concentration profiles obtained for different initial concentrations of **1a** in the presence of 2.5 mM catalyst (*S*)-**C5**.

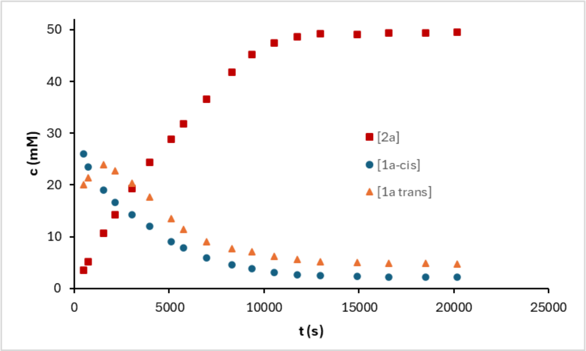

Figure S15. Concentration profiles obtained for reaction with **1a** (0.05M) in the presence of 2.5 mM catalyst (*S*)-**C5**.

Order in the catalyst.

An oven-dried NMR tube with a screw cap having a septum was charged with **1a** (0.03 mmol), 0.4 ml of toluene-d_8. The NMR tube was cooled down to -10°C, and (*S*)-**C5** (0.0021 mmol) in 0.2 ml of toluene-d_8 or (*S*)-**C5** (0.0042 mmol) in 0.2 ml of toluene-d_8 was added. After shimming, ^{1}H NMR spectra were acquired every 10-30 minutes until the starting material was > 80% consumed.

The following figure shows concentration profiles of **2a** (initial concentration of **1a** 0.05M, in toluene-d_8) in the presence of different amounts of catalyst (S)-**C5** with time scales normalized to different substrate orders (0th, 1st, 2nd):

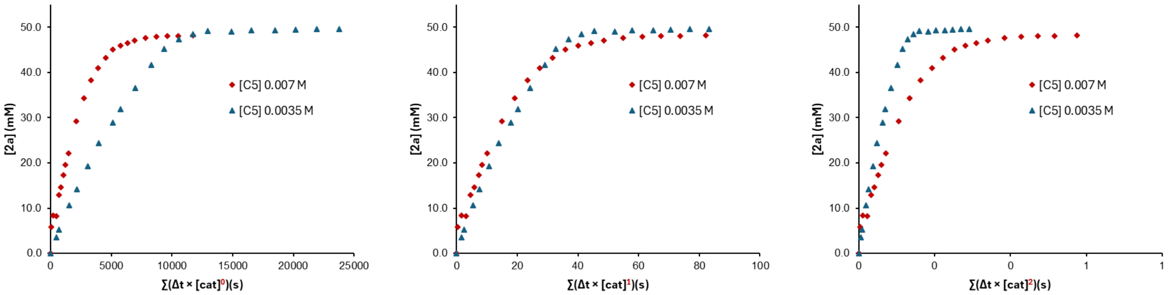

Figure S16. NMR concentration profiles with time scales normalized to different catalyst orders (left: zeroth order, middle: first order, right: second order). The best overlap is observed assuming a first-order dependence on catalyst concentration.

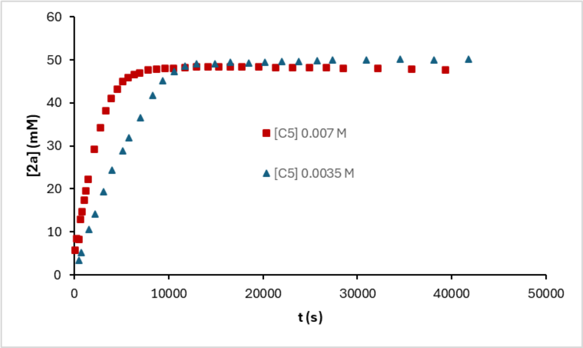

Figure S17. Concentration profiles obtained using different concentrations of the catalyst (*S*)-**C5**, with an initial concentration of **1a** of 0.05 M.

Elimination of trichloroacetimidate **1ag**

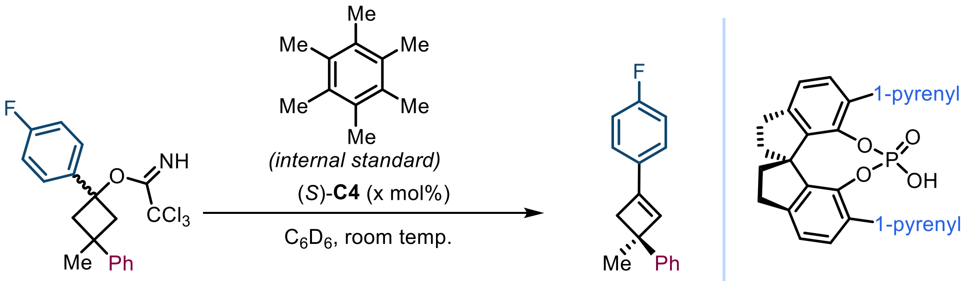

An oven-dried NMR tube with a screw cap having a septum was charged with **1ag** (0.03 mmol/ 0.015 mmol), 0.3 ml of C_6D_6, hexamethylbenzene (0.005 mmol) in 0.1 ml of C_6D_6, and *(S)*-**C4** (0.0043 mmol) in 0.2 ml of C_6D_6. The NMR tube was then transferred to the NMR spectrometer, and after shimming, 1H NMR spectra were acquired every 20-30 minutes until the starting material was > 80% consumed.

The following figure shows concentration profiles of **2z** using different initial concentrations of **1ag** 0.05M, 0.025M in C_6D_6 in the presence of catalyst (*S*)-**C4** with times scales normalized to different substrate orders (-0.1th, 0.4th, 1st):

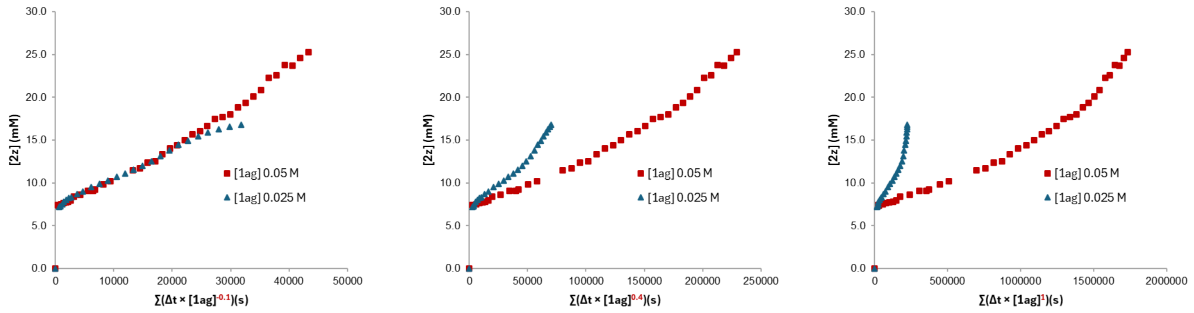

Figure S18. NMR concentration profiles with time scales normalized to different substrate orders (left: assumed zeroth order (-0.1), middle: fractional order (0.4), right: first order). The best overlap is observed assuming a zeroth-order (-0.1th) dependence on substrate concentration.

The following concentration profiles were obtained for using different initial concentrations of **1ag** (0.05M, 0.025 M):

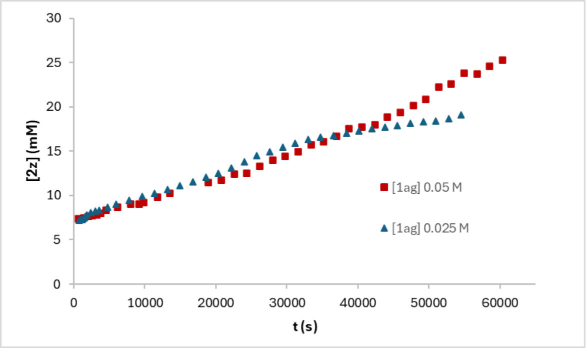

Figure S19. Concentration profiles obtained for different initial concentrations of **1ag** in the presence of 7.2 mM catalyst (*S*)-**C4**.

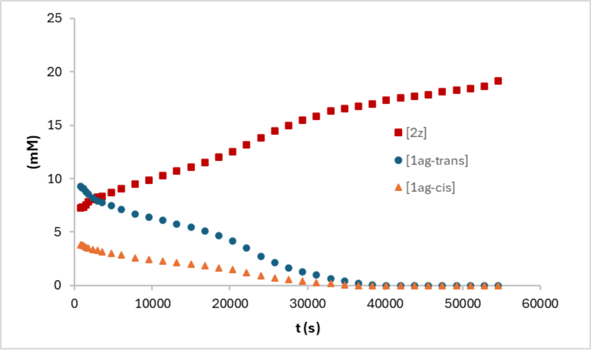

Figure S20. Concentration profiles obtained for reaction with **1ag** (0.025M) in the presence of 5 mM catalyst (*S*)-**C4**. The profile indicates that **1ag-*cis*** and **1ag-*trans*** are consumed at similar rates.

Order in the catalyst.

An oven dried NMR tube with a screw cup having a septum was charged with **1ag** (0.03 mmol), 0.3 ml or 0.1 ml of C_6D_6, hexamethylbenzene (0.005 mmol) in 0.1 ml of C_6D_6 and (*S*)-**C4** (0.0043 mmol) in 0.2 ml of C_6D_6 or (*S*)-**C4** (0.01 mmol) in 0.4 ml of C_6D_6. The NMR tube was then transferred to the NMR spectrometer, and after shimming, ^{1}H NMR spectra were acquired every 20-30 minutes until the starting material was > 60% consumed.

The following figure shows concentration profiles of **2z** (initial concentration of **1ag** 0.05M, in C_6D_6) in the presence of different amounts of catalyst (*S*)-**C4** with time scales normalized to zeroth, first, and second order. ^{1}H NMR analysis performed a few minutes after catalyst addition indicates rapid product formation at concentrations comparable to the added catalyst, followed by a slower conversion (FigureS21a). To apply VTNA, the reaction profile obtained using catalyst (*S*)-**C4** at a concentration of 0.017 M was shifted vertically until the starting points of the two reaction profiles matched (Figure S21b).

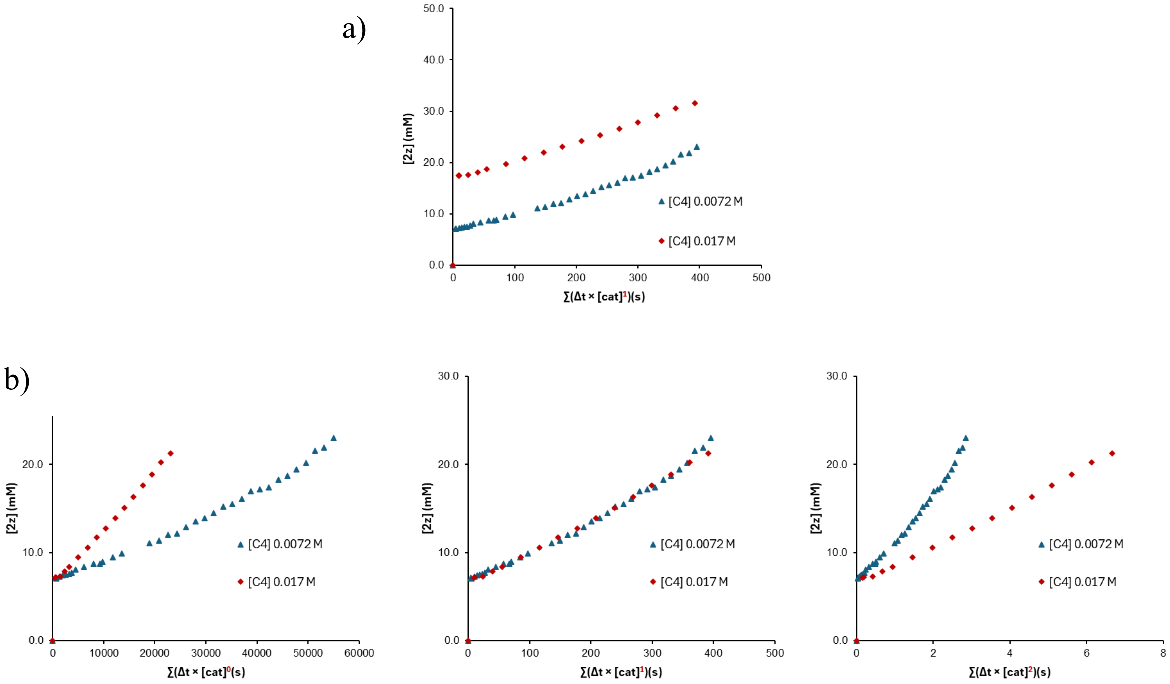

Figure S21. a) NMR concentration profiles starting at different coordinates with time scales normalized to the first catalyst order. b) Vertically shifted NMR concentration profiles of **2z** with time scales normalized to different catalyst orders (left: zeroth order, middle: first order, right: second order). The best overlap is observed assuming a first-order dependence on catalyst concentration.

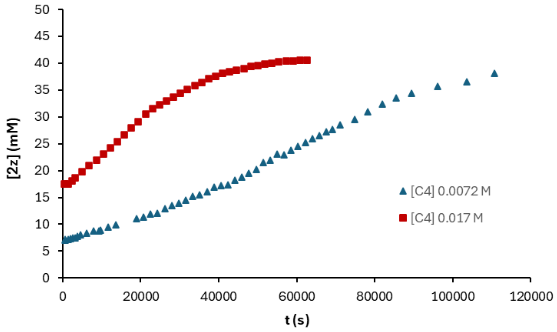

Figure S22. Concentration profiles obtained using different concentrations of the catalyst (*S*)-**C4**, with an initial concentration of **1ag** of 0.05 M.

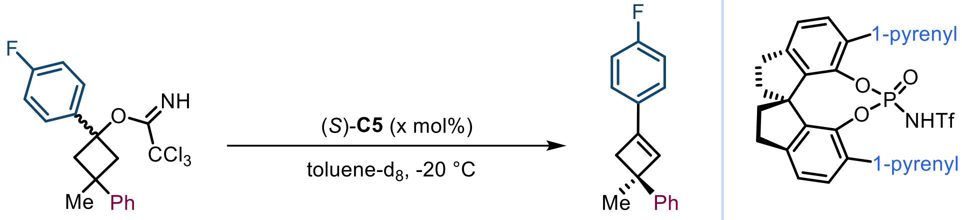

1ag
cis/*trans* 2:1

2z

(*S*)-**C5**

Order in the substrate.

An oven-dried NMR tube with a screw cap having a septum was charged with **1ag** (0.03 mmol/ 0.0225 mmol/ 0.015 mmol), 0.4 ml of toluene-d_8. The NMR tube was cooled down to -20°C, and (*S*)-**C5** (0.0015 mmol) in 0.2 ml of toluene-d_8 was added. After shimming, ^{1}H NMR spectra were acquired every 10-20 minutes until the starting material was > 80% consumed.

Due to the overlap between the signals of hexamethylbenzene and toluene, the analysis was performed without the internal standard. All concentration points were referenced to the first acquired ^{1}H NMR spectrum.

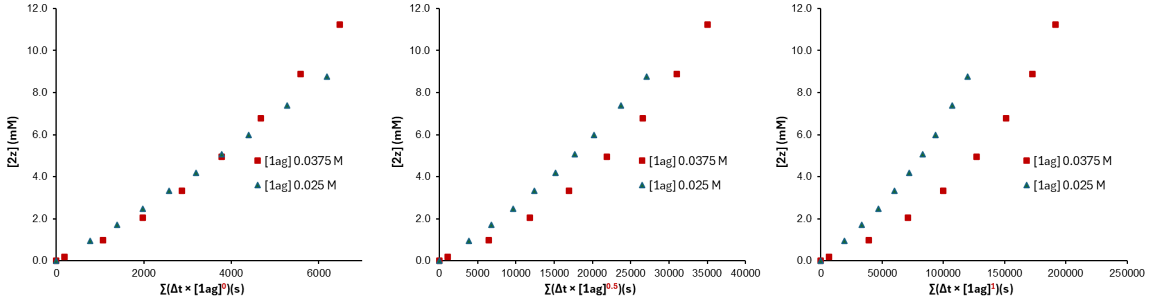

Figure S23. NMR concentration profiles with time scales normalized to different substrate orders (left: assumed zeroth order, middle: fractional order (0.5), right: first order). The best overlap is observed assuming a zeroth-order dependence on substrate concentration.

The following concentration profiles were obtained for using different initial concentrations of **1ag** (0.5 M, 0.0375 M, 0.025 M). While zero-order dependence on **1ag** was observed at low initial substrate concentrations (0.025 and 0.0375 M), the reaction rate decreases significantly at a higher initial concentration (0.05 M), resulting in a negative order with respect to **1ag** at higher substrate concentrations (0.0375 and 0.05 M). This behavior may be attributed to inhibition of the elimination from the catalyst–substrate covalent intermediate **V** (Figure S31) by excess **1ag**, possibly arising from hydrogen-bonding interactions between the substrate and the intermediate.

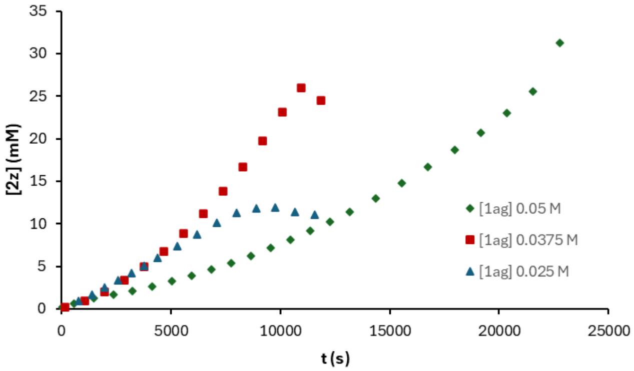

Figure S24. Concentration profiles obtained for different initial concentrations of **1ag** in the presence of 2.5 mM catalyst (*S*)-**C5**.

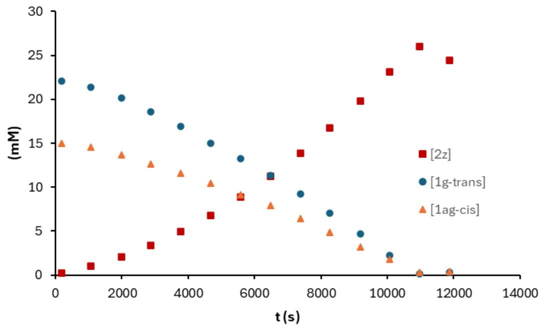

Figure S25. Concentration profiles obtained for reaction with **1ag** (0.0375M) in the presence of 2.5 mM catalyst (*S*)-**C5**. The profile indicates that **1ag-*cis*** and **1ag-*trans*** are consumed at similar rates.

Order in the catalyst.

An oven-dried NMR tube with a screw cap having a septum was charged with **1ag** (0.0225 mmol) and 0.5 ml or 0.4 ml of toluene-d_8. The NMR tube was cooled down to -20°C, and (*S*)-**C5** (0.75 µmol) in 0.1 ml of toluene-d_8 or (*S*)-**C5** (1.5 µmol) in 0.2 ml of toluene-d_8 was added. After shimming, ^{1}H NMR spectra were acquired every 10-20 minutes until the starting material was > 80% consumed.

The following figure shows concentration profiles of **2z** (initial concentration of **1ag** 0.0375M, in toluene-d_8) in the presence of different amounts of catalyst (S)-**C5** with time scales normalized to different substrate orders (-0.6th, 0th 1st):

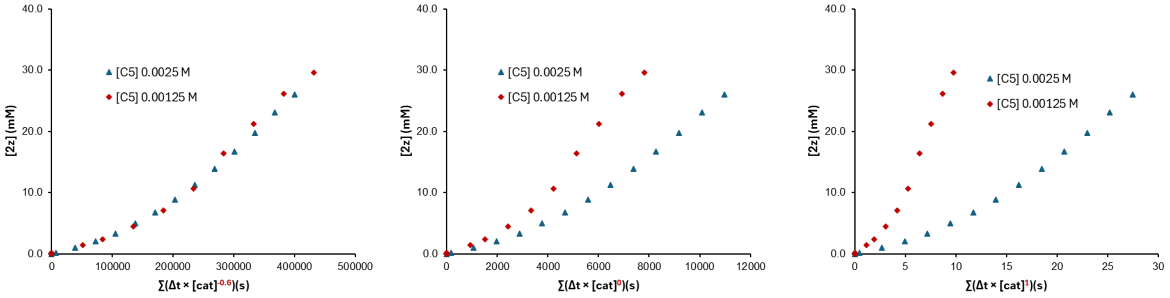

Figure S26. NMR concentration profiles with time scales normalized to different catalyst orders (left: negative, middle: zeroth, right: second order). The best overlap is observed assuming a negative (-0.6) dependence on catalyst concentration.

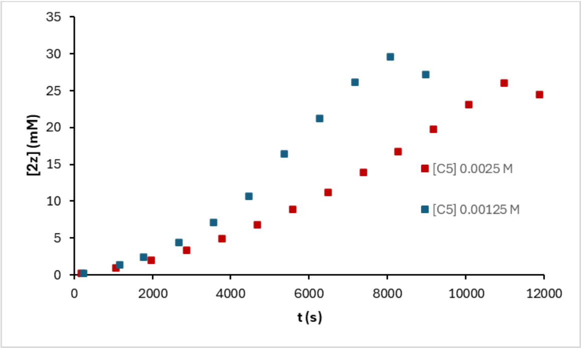

Figure S27. Concentration profiles obtained using different concentrations of the catalyst (*S*)-**C5**, with an initial concentration of **1ag** of 0.0375 M.

Monitoring the reaction progress by ^{31}P NMR

To investigate the formation of a catalyst-substrate covalent intermediates **IV** and **V,** we followed the reaction progress by ^{31}P NMR and ^{1}H spectroscopy until the starting material was > 80% consumed. Selected ^{31}P NMR spectra are shown in **Figures S28-31.**

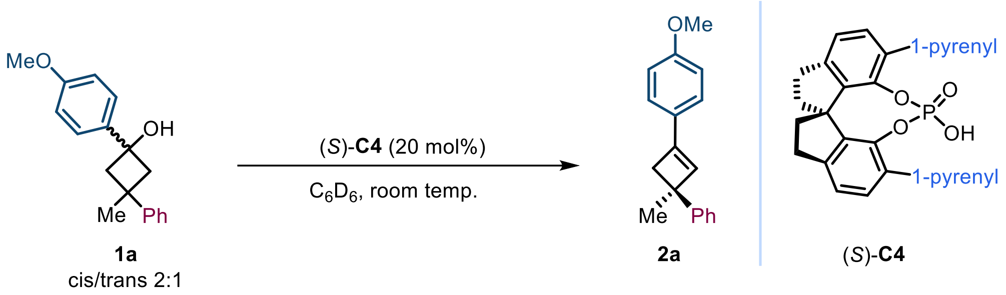

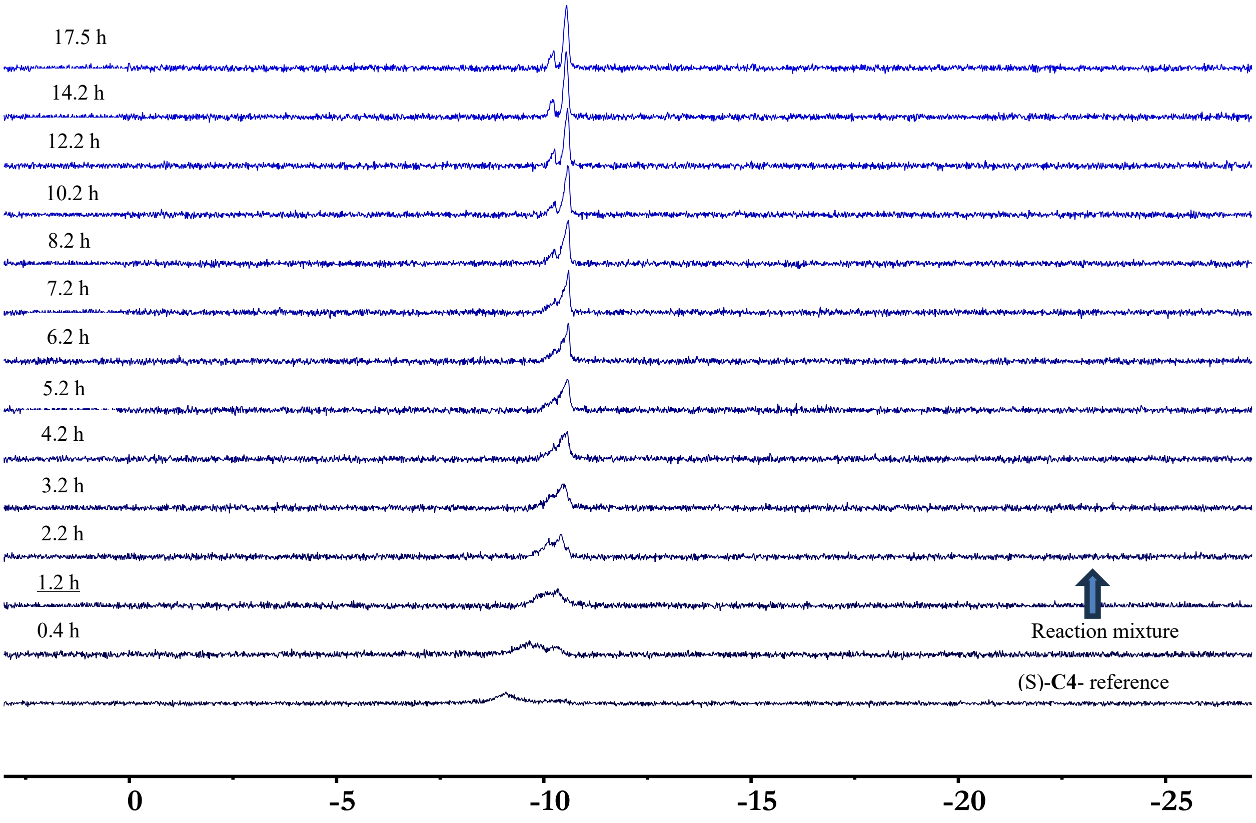

Figure S28. ^{31}P NMR spectra of the elimination of **1a** using chiral phosphoric acid (*S*)-**C4.**

A small shift in the peak of (*S*)-**C4** could be attributed to interactions between the catalyst and the hydroxyl group of **1a** or with water formed during the reaction**.** Splitting of the broad catalyst signals into two peaks could be due to the presence of different rotamers[19] rather than the formation of a new species. The ^{31}P NMR reaction profile is markedly different from the profile presented in Figure S30, where formation of a catalyst–substrate intermediate is postulated.

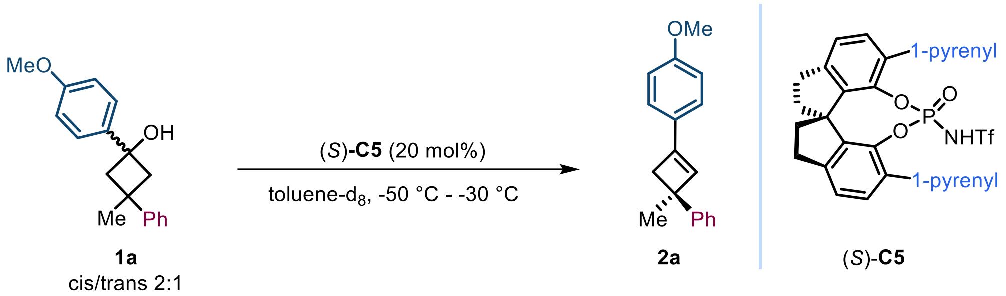

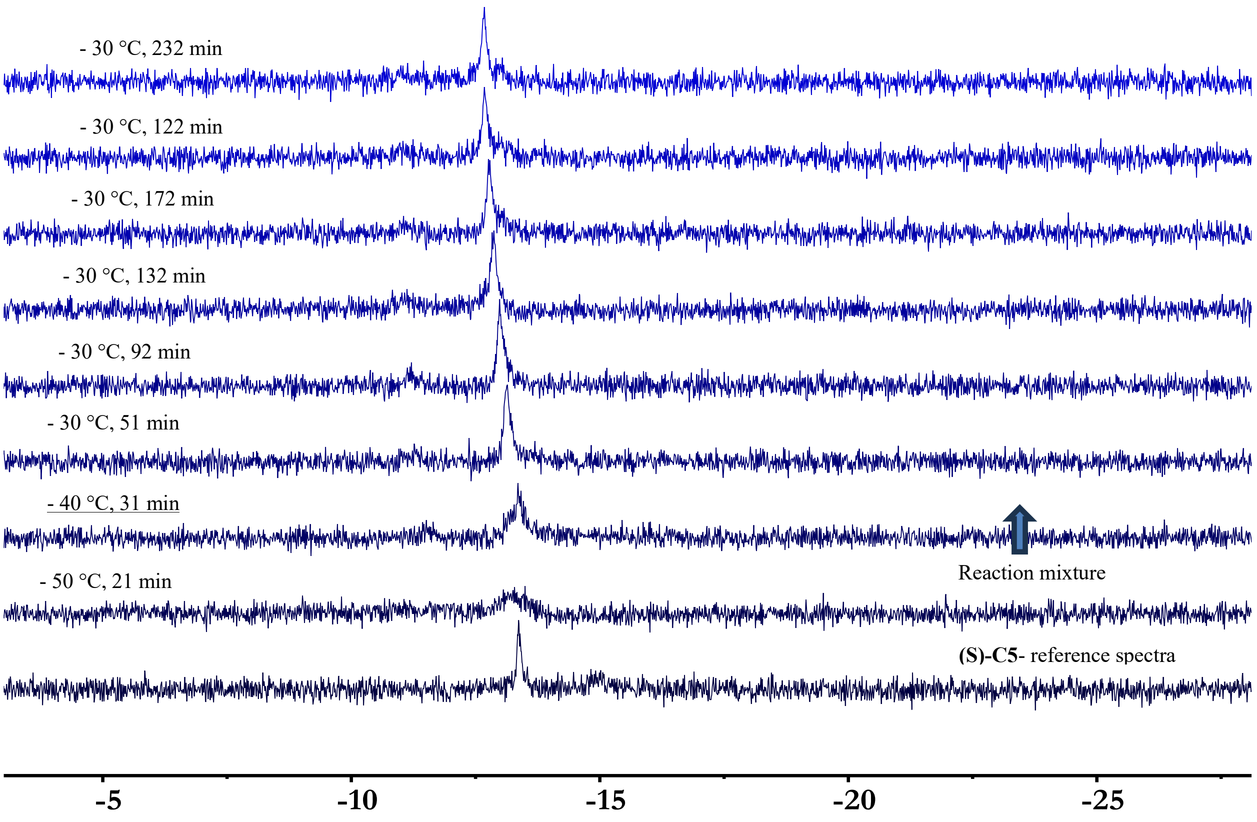

Figure S29. ^{31}P NMR spectra of the elimination of **1a** using chiral *N*-triflyl phosphoramide (*S*)-**C5.**

Due to the high reactivity of (*S*)-**C5**, the experiment was initiated at −50 °C, followed by a gradual increase in temperature until reaction progress was observed by ^{1}H NMR. A small shift in the peak of (*S*)-**C5** could be attributed to interaction between the catalyst and the hydroxyl group of **1a** or with water formed during the reaction.

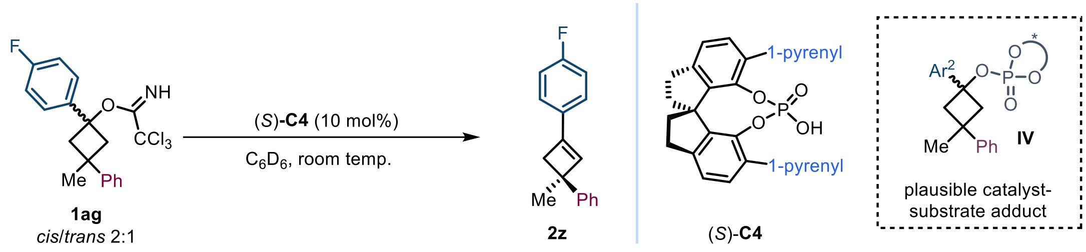

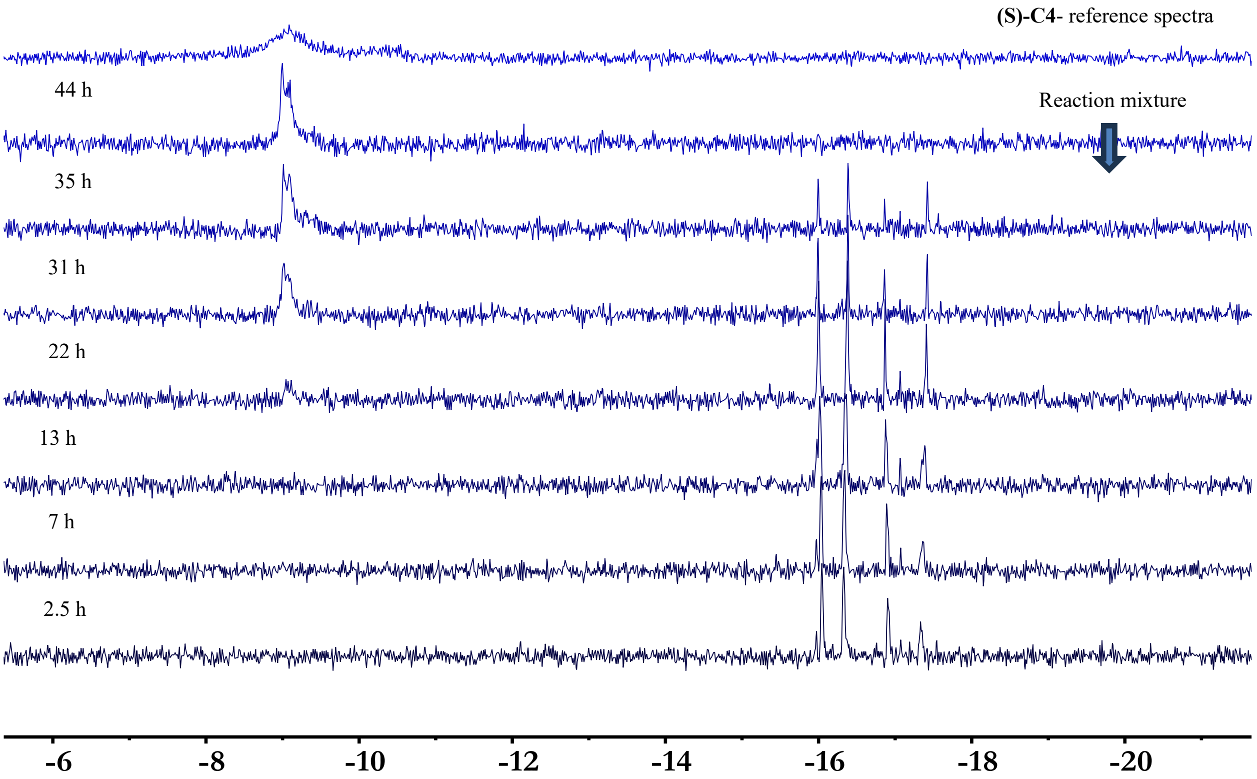

Figure S30. ^{31}P NMR spectra of the elimination of **1ag** using chiral phosphoric acid **(*S*)-C4.**

We tentatively assigned the new peaks at ^{31}P NMR spectra to the catalyst-substrate adduct **IV**. Two diastereomers of **IV** can be formed; therefore, only two peaks are expected. Additional signals may be attributed to the presence of different rotamers. After consumption of **1ag** (observed by ^{1}H NMR), a growth of the peak at −9 ppm corresponding to (*S*)-**C4** and the disappearance of signals corresponding to the catalyst–substrate adducts were observed.

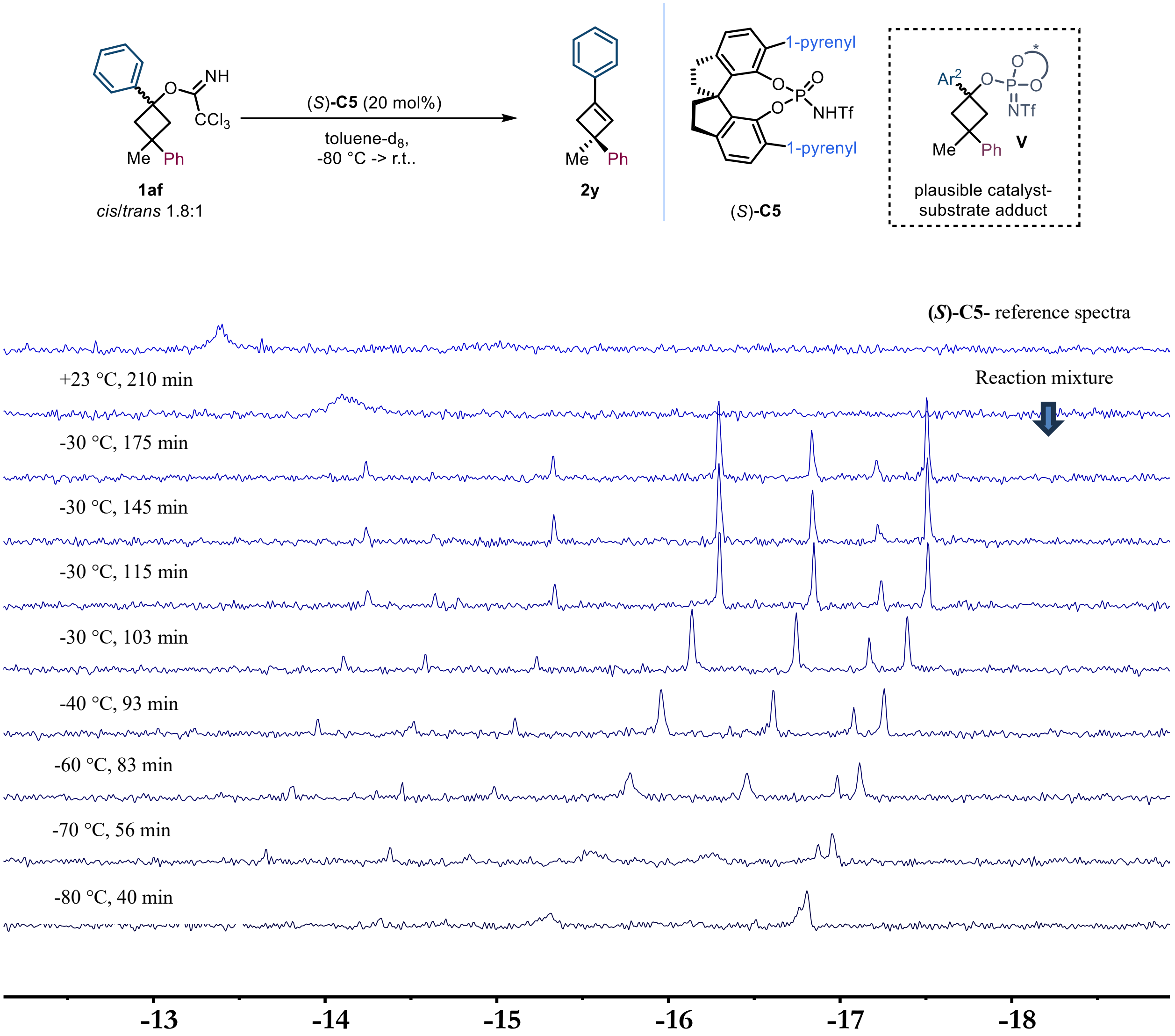

Figure S31. ^{31}P NMR spectra of the elimination of **1af** using *N*-triflyl phosphoramide **(*S*)-C5.**

Due to the high reactivity of (*S*)-**C5**, the experiment was initiated at −80 °C, followed by a gradual increase in temperature until reaction progress was observed by ^{1}H NMR. After full consumption of **1y** was observed by ^{1}H NMR, the reaction mixture was warmed up to room temperature. We tentatively assigned the new peaks at ^{31}P NMR spectra to the catalyst-substrate adduct **V**. Two diastereomers of **V** can be formed; therefore, only two peaks are expected. Additional signals may be attributed to the presence of different rotamers. After warming up the reaction to room temperature disappearance of peaks corresponding to the catalyst–substrate adducts and the appearance of the signal can be attributed to the catalyst (*S*)-**C5** were observed. A small signal shift relative to the reference spectra may be attributed to the presence of trichloroacetamide in the reaction mixture.

ESI-MS Studies

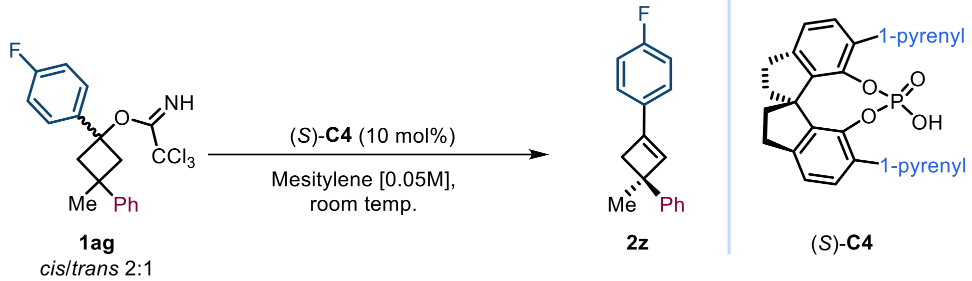

In order to further examine the formation of a catalyst-substrate adduct **IV,** we analyzed the reaction mixture by Electrospray Ionization Mass Spectroscopy (ESI-MS). The elimination reaction of **1ag** (6 mg, 15 µmol) with catalyst (*S*)-**C4** (1.1 mg, 1.5 µmol) in 300 µL of mesitylene was performed at room temperature. The samples of the reaction mixture were monitored at different times by HRMS. A few minutes **1ag**, catalyst (*S*)-**C4** was combined, and the new peak at m/z 953.3 could be detected, which matches the mass of the covalent intermediate [**IV** + H]$^+$. The intermediate **IV** and the catalyst **(S)-C4** (m/z 715.2) were also detected during the further course of the reaction.

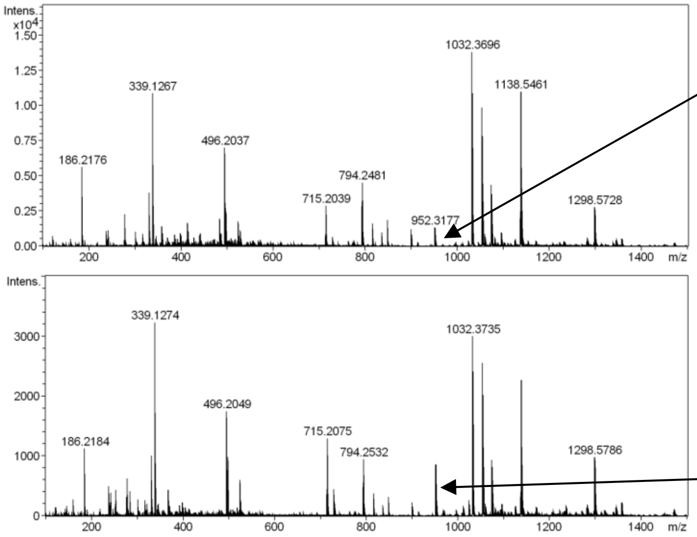

#	m/z	Res.	S/N	I	I %	FWHM
138	952.3177	5130	91.3	1335	9.7	0.1856
139	953.3206	4975	66.6	975	7.1	0.1916

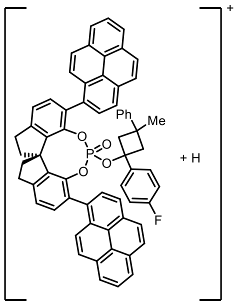

Chemical Formula: [$C_{66}H_{46}FO_4P$ + H]$^+$
HRMS calc.: 953.3191
10 min reaction time:
HRMS found.: 953.3206
deviation: 0.0015
3h reaction time:
HRMS found.: 953.3236
deviation: 0.0045

#	m/z	Res.	S/N	I	I %	FWHM
72	952.3218	5140	192.8	856	26.5	0.1853
73	953.3236	5073	139.5	619	19.2	0.1879

#	m/z	Res.	S/N	I	I %	FWHM
115	715.2039	5062	212.6	2887	20.9	0.1413

#	m/z	Res.	S/N	I	I %	FWHM
55	715.2075	5108	325.7	1297	40.2	0.1400

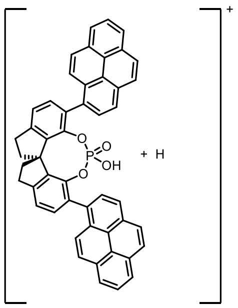

Chemical Formula: [$C_{49}H_{31}O_4P$ + H]$^+$
HRMS calc.: 715.2023
10 min reaction time:
HRMS found.: 715.2039
deviation: 0.0016
3h reaction time:
HRMS found.: 715.2075
deviation: 0.0052

Figure S33. ESI-MS analysis of the elimination reaction of **1ag** with catalyst **(*S*)-C4**.

Kinetic isotope effect studies

General procedure for Kinetic Isotope Effect Studies of Cyclobutanols

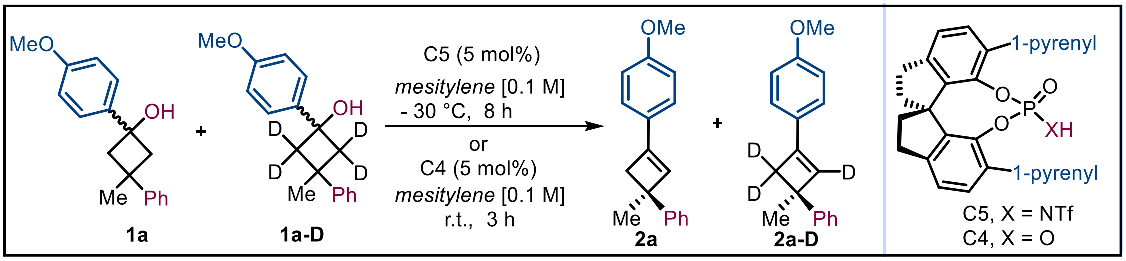

Sample preparation for *N*-triflyl phosphoramides-catalyzed reaction.

An oven-dried 8 ml vial equipped with a magnetic stirring bar was charged with 1-(4-methoxyphenyl)-3-methyl-3-phenylcyclobutan-1-ol **1a** (26.8 mg, 0.100 mmol, 1.0 equiv., *cis*/*trans* 1.8:1) and 1-(4-methoxyphenyl)-3-methyl-3-phenylcyclobutan-2,2,4,4-d4-1-ol **1a-D (95%D)** (27.2 mg, 0.100 mmol, 1 equiv., *cis*/*trans* 1.8:1). The vial was evacuated and backfilled with argon 3 times. Next, 0.7 ml of mesitylene was added, and the mixture was stirred at -30 °C for 20 min. Next, the catalyst **C5** (4.2 mg, 5 µmol, 0.05 equiv.) was added in 0.3 mL of mesitylene at -30 °C. The mixture was stirred (700 rpm) at -30 °C for 8 h. After this, the reaction was quenched by the addition of Et_3N (100 µl), and the solution was transferred to a 50 ml round-bottom flask with hexane, and the solvent was removed under reduced pressure. The mixture was purified by column chromatography (EtOAc-hexane 5%). The ratio k_H/k_D was determined by ^{1}H NMR analysis of the isolated product to be 7.61 (average of two runs).

Sample preparation for phosphoric acid-catalyzed reaction

An oven-dried 8 ml vial equipped with a magnetic stirring bar was charged with 1-(4-methoxyphenyl)-3-methyl-3-phenylcyclobutan-1-ol **1a** (26.8 mg, 0.100 mmol, 1.0 equiv., *cis*/*trans* 1.8:1), 1-(4-methoxyphenyl)-3-methyl-3-phenylcyclobutan-2,2,4,4-d4-1-ol **1a-D (95%D)** (27.2 mg, 0.100 mmol, 1 equiv., *cis*/*trans* 1.8:1) and catalyst **C4** (3.57 mg, 5 µmol, 0.05 equiv.). The vial was evacuated and backfilled with argon 3 times. Next, 1.0 ml of mesitylene was added, and the mixture was stirred at room temperature for 3 hours. After this, the reaction was quenched by the addition of Et_3N (100 µl), and the solution was transferred to a 50 ml round-bottom flask with hexane, and the solvent was removed under reduced pressure. The mixture was further purified by column chromatography (EtOAc-hexane 5%). The ratio k_H/k_D was determined by ^{1}H NMR analysis of the isolated product to be 2.09 (average of two runs).

The observed KIE_{exp} were conversion–corrected by using the formula provided below[20]:

$KIE = \frac{\ln(1-F)}{\ln[1-F(\frac{R}{R_0})}$ where, F: fractional conversion, R: proportion of an isotopic component in the product, R_0; proportion of an isotopic component in the substrate.

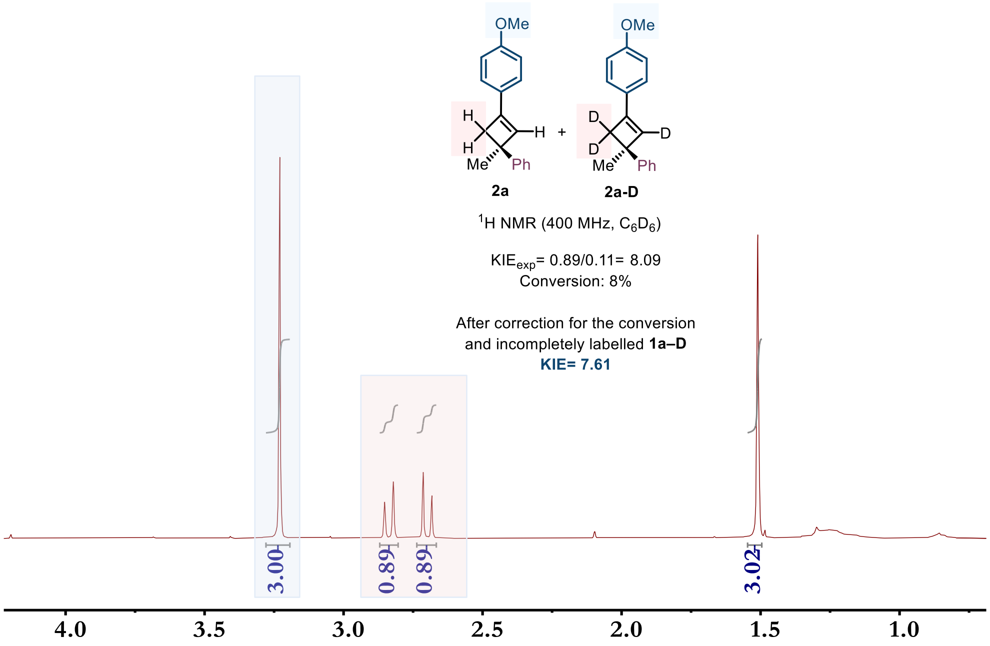

Figure 34. Expanded region of the ^{1}H NMR spectra of **2a** and **2a-D** obtained using catalyst **C5**.

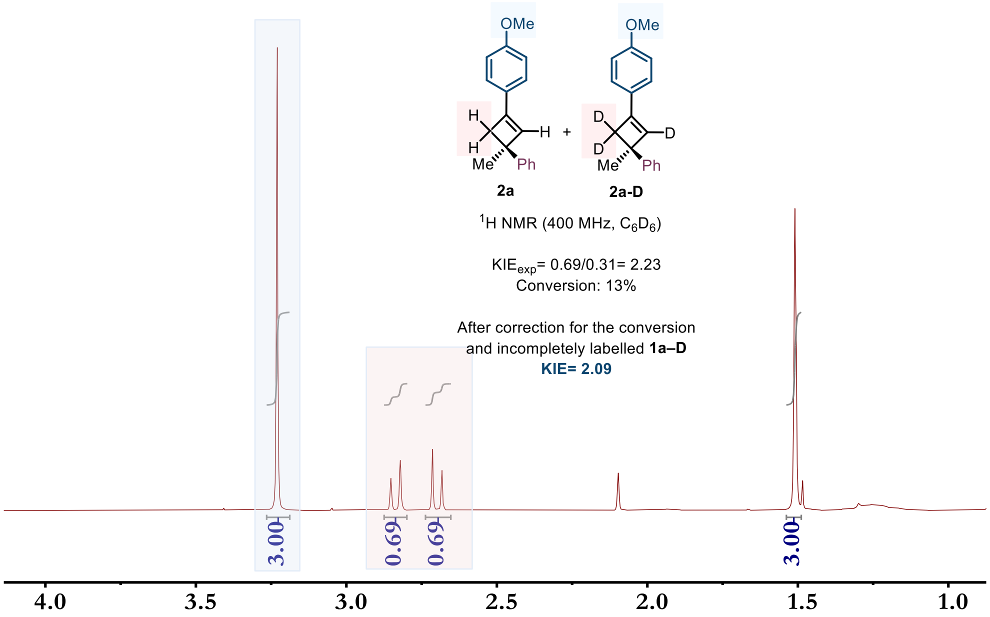

Figure 35. Expanded region of the ^{1}H NMR spectra of **2a** and **2a-D** obtained using catalyst **C4**.

General procedure for Kinetic Isotope Effect Studies of TCA

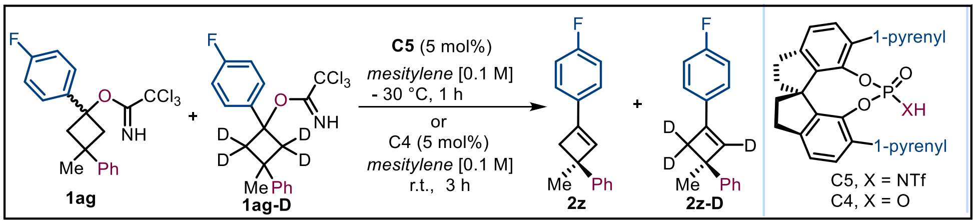

Sample preparation for *N*-triflyl phosphoramides-catalyzed reaction.

An oven-dried 8 ml vial equipped with a magnetic stirring bar was charged with 1-(4-fluorophenyl)-3-methyl-3-phenylcyclobutyl 2,2,2-trichloroacetimidate **1ag** (20.0 mg, 0.05 mmol, 1.0 equiv.) and 1-(4-fluorophenyl)-3-methyl-3-phenylcyclobutyl-2,2,4,4-d_4 2,2,2-trichloroacetimidate **1ag-D (96%D)** (20.0 mg, 0.05 mmol, 1.0 equiv.). The vial was evacuated and backfilled with argon 3 times. Next, 0.3 ml of mesitylene was added, and the mixture was stirred at -30 °C for 20 min. Next, the catalyst **C5** (2.1 mg, 2.5 µmol, 0.05 equiv.) was added in 0.2 mL of mesitylene at -30 °C. The mixture was stirred (700 rpm) at -30 °C for 1 h. After this, the reaction was quenched by the addition of Et_3N (100 µl), and the solution was transferred to a 50 ml round-bottom flask with hexane, and the solvent was removed under reduced pressure. The mixture was further purified by column chromatography on neutral alumina (EtOAc-hexane 5%). The ratio k_H/k_D was determined by ^{1}H NMR analysis of the isolated product to be 2.12 (average of two runs).

Sample preparation for acid-catalyzed reaction

An oven-dried 8 ml vial equipped with a magnetic stirring bar was charged with 1-(4-fluorophenyl)-3-methyl-3-phenylcyclobutyl 2,2,2-trichloroacetimidate **1ag** (20.0 mg, 0.05 mmol, 1.0 equiv.) and 1-(4-fluorophenyl)-3-methyl-3-phenylcyclobutyl-2,2,4,4-d4 2,2,2-trichloroacetimidate **1ag-D (96%D)** (20.0 mg, 0.05 mmol, 1 equiv.) and catalyst **C4** (1.8 mg, 2.5 µmol, 0.05 equiv.). The vial was evacuated and backfilled with argon 3 times. Next, 0.5 ml of mesitylene was added, and the mixture was stirred at room temperature for 3 hours. After this, the reaction was quenched by the addition of Et_3N (100 µl), and the solution was transferred to a 50 ml round-bottom flask with hexane, and the solvent was removed under reduced pressure. The mixture was further purified by column chromatography on neutral alumina (EtOAc-hexane 5%). The ratio k_H/k_D was determined by ^{1}H NMR analysis of the isolated product to be 1.52 (average of two runs).

The observed KIE_{exp} were conversion–corrected by using the formula provided below[20]:

$KIE = \frac{\ln(1-F)}{\ln[1-F(\frac{R}{R_0})}$ where, F: fractional conversion, R: proportion of an isotopic component in the product, R_0; proportion of an isotopic component in the substrate.

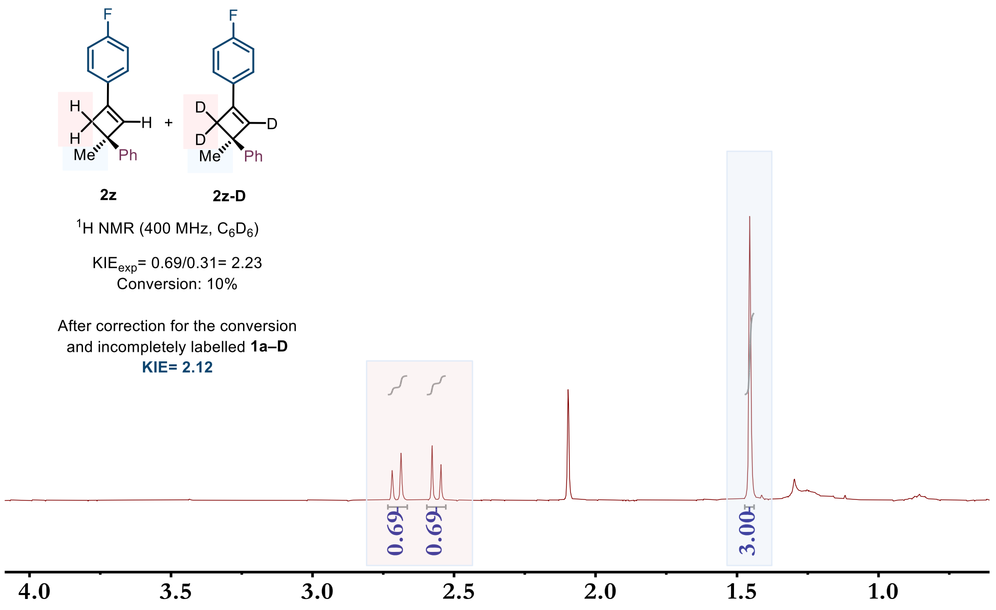

Figure 36. Expanded region of the ^{1}H NMR spectra of **2z** and **2z-D** obtained using catalyst **C5**.

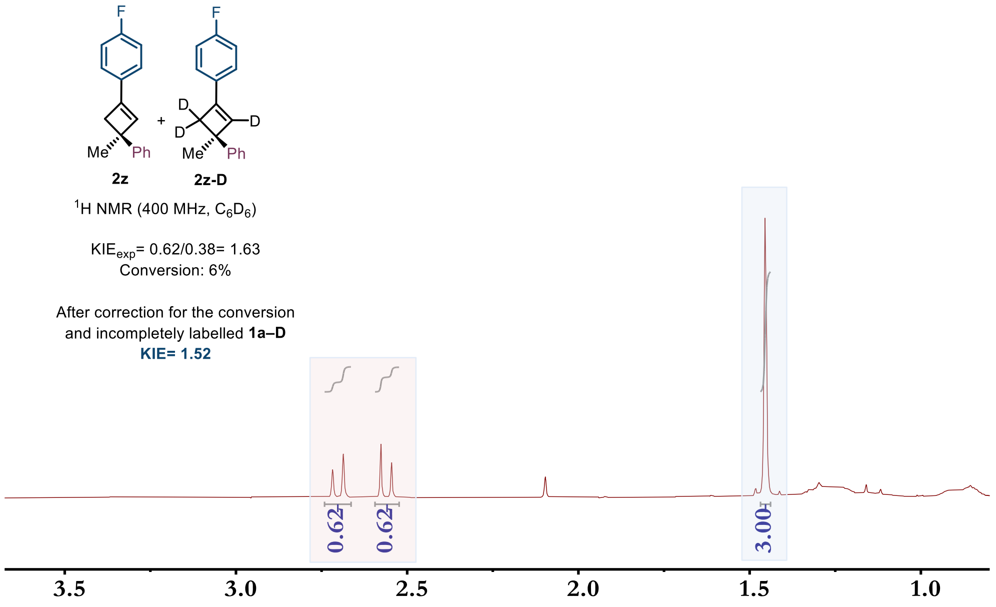

Figure 37. Expanded region of the ^{1}H NMR spectra of **2z** and **2z-D** obtained using catalyst **C4**.

Reactions with nucleophilic additives

To evaluate the formation of a carbocation intermediate, we performed experiments with EtOH, benzothiazole-2(3H)-thione, and water. Ethanol suppresses the reaction (elimination and epimerization) of **1a**, but it reacts with trichloroacetimidate **1aj** to form ether **5c**. This is consistent with the generation of a carbocationic intermediate from **1aj** but not from **1a**, which is proposed to eliminate via an E2 mechanism. Addition of water under standard reaction conditions lowered the yield of the elimination product to 28% but did not influence the enantioselectivity. The opposite ratio of cis and trans diastereoisomers in the remaining **1a** indicates that background epimerization via S_N1 is taking place. Formation of product **5b** in the presence of benzothiazole-2(3H)-thione further suggests the formation of a carbocationic intermediate during the reaction; however, it might not be involved in the elimination process.

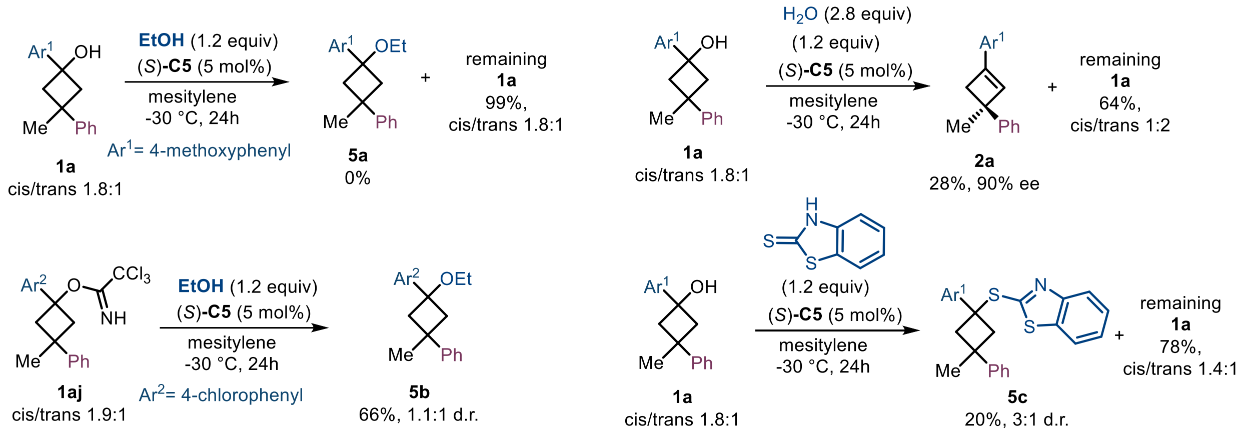

Figure S38. Reactions with diverse nucleophilic additives.

Reaction with the ethanol additive

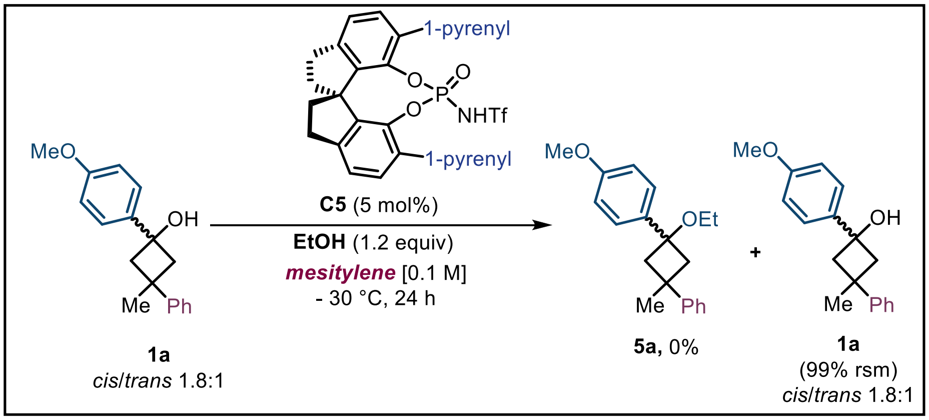

An oven-dried 8 ml vial equipped with a magnetic stirring bar was charged with 1-(4-methoxyphenyl)-3-methyl-3-phenylcyclobutan-1-ol **1a** (26.8 mg, 0.100 mmol, 1 equiv., *cis*/*trans* 1.8:1). The vial was evacuated and backfilled with argon 3 times. Next, 0.7 ml of mesitylene and ethanol (7.01 µl, 0.120 mmol, 1.2 equiv.) were added, and the mixture was stirred at -30 °C for 20

min. Next, the catalyst (4.2 mg, 5 µmol, 0.05 equiv.) was added in 0.3 mL of mesitylene at -30 °C. The mixture was stirred (700 rpm) at -30 °C for 24 h. After this, the reaction was quenched by the addition of Et_3N (100 µl), and the mixture was purified by column chromatography (EtOAc-hexane 5-10%), which afforded only unreacted starting material **1a** (*cis*/*trans* = 1.8:1; 26.5 mg, 0.100 mmol).

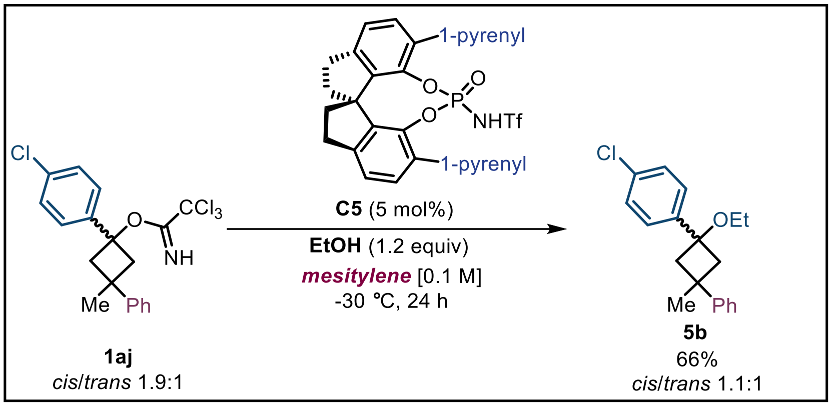

An oven-dried 8 ml vial equipped with a magnetic stirring bar was charged with 1-(4-chlorophenyl)-3-methyl-3-phenylcyclobutyl 2,2,2-trichloroacetimidate **1aj** (25.0 mg, 0.06 mmol, 1 equiv., *cis*/*trans* 1.9:1). The vial was evacuated and backfilled with argon 3 times. Next, 0.4 ml of mesitylene and ethanol (4.2 µl, 0.072 mmol, 1.2 equiv.) were added, and the mixture was stirred at -30 °C for 20 min. Next, the catalyst (2.5 mg, 3 µmol, 0.05 equiv.) was added in 0.2 mL of mesitylene at -30 °C. The mixture was stirred (700 rpm) at -30 °C for 24 h. After this, the reaction was quenched by the addition of Et_3N (100 µl), and the mixture was purified by column chromatography (EtOAc-hexane 5-10%), which afforded trapping product **5b** in 66% yield (12.0 mg, 0.04 mmol, *cis*/*trans* = 1.1:1).

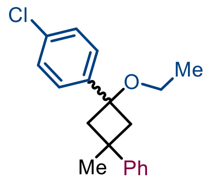

5b

^{1}H NMR (400 MHz, $CDCl_3$): (mixture of diastereomers, signals are reported as seen) δ 7.44 (d, *J* = 8.3 Hz,1.79H), 7.38 – 7.30 (m, 3.47H), 7.30 – 7.09 (m, 12H), 3.12 (q, *J* = 7.0 Hz, 2H), 2.94 (q, *J* = 7.0 Hz, 1.85H), 2.87 – 2.74 (m, 5.66H), 2.69 – 2.61 (m, 2H), 1.64 (s, 3H), 1.22 (s, .68H), 1.15 (t, *J* = 7.0 Hz, 3H), 0.96 (t, *J* = 7.0 Hz, 2.68H); **^{13}C NMR (101 MHz, $CDCl_3$)**: (mixture of diastereomers, signals are reported as seen) δ 152.1, 151.7, 143.1, 142.8, 133.2, 132.8, 128.7, 128.5, 128.4, 128.3, 128.2, 127.6, 125.5, 125.4, 125.2, 125.1, 76.8, 75.8, 58.3, 57.9, 45.6, 44.3, 36.7, 34.4, 32.6, 31.4, 15.7, 15.5; **IR (ATR):** 3058, 3024, 2973, 2931, 2867, 1748, 1601, 1492, 1445, 1419, 1293, 1241, 1117, 1091, 1068, 1028, 1014, 829, 763, 738, 700, 552, 405 cm^{-1} **HRMS (ESI)**: calcd. for $[C_{19}H_{21}ClO_2+Na]^+$, $[M+Na]^+$: 323.1173; found: 323.1174; ***R*f**: 0.36 (Hexane/EtOAc, 95:5);

Reaction with water additive

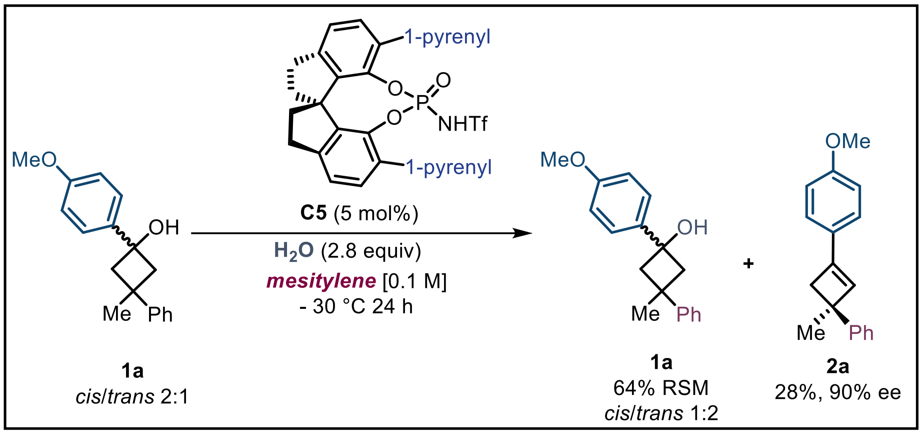

An oven-dried 8 ml vial equipped with a magnetic stirring bar was charged with substrate 1-(4-methoxyphenyl)-3-methyl-3-phenylcyclobutan-1-ol **1a** (26.8 mg, 0.100 mmol, 1 equiv., *cis*/*trans* 1.75:1). The vial was evacuated and backfilled with argon 3 times. Next, 0.7 ml of mesitylene and distilled water (5.0 µl, 0.18 mmol, 2.8 equiv.) were added, and the mixture was stirred at -30 °C for 20 min. Next, the catalyst (4.2 mg, 5 µmol, 0.05 equiv.) was added in 0.3 mL of mesitylene at -30 °C. The mixture was stirred (700 rpm) at -30 °C for 24 h. After this, the reaction was quenched by the addition of Et_3N (100 µl) and the mixture was purified by column chromatography (EtOAc-hexane 5-10%) to afford the elimination product **2a** as a white solid in 28% yield, 94.9:5.1 er (6.9 mg, 0.028 mmol), along with 64% recovered starting material **1a** (*cis*/*trans* = 1:2; 17.2 mg, 0.064 mmol).

Reaction with the benzo[d]thiazole-2(3H)-thione additive

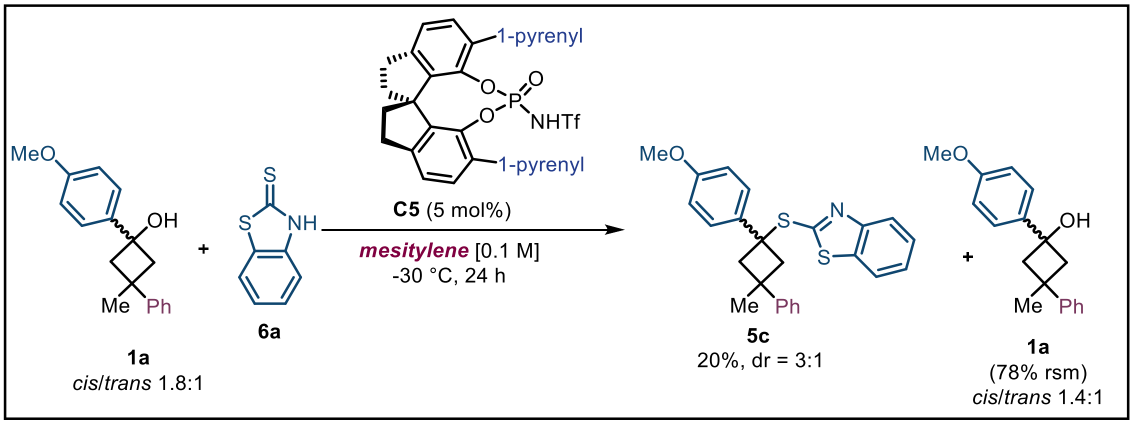

An oven-dried 8 ml vial equipped with a magnetic stirring bar was charged with 1-(4-methoxyphenyl)-3-methyl-3-phenylcyclobutan-1-ol **1a** (26.8 mg, 0.100 mmol, 1 equiv., *cis*/*trans* 1.8:1) and benzo[d]thiazole-2(3H)-thione **6a** (20.1 mg, 0.120 mmol, 1.2 equiv.). The vial was evacuated and backfilled with argon 3 times. Next, 0.7 ml of mesitylene was added, and the

mixture was stirred at -30 °C for 20 min. Next, the catalyst (4.2 mg, 5 µmol, 0.05 equiv.) was added in 0.3 mL of mesitylene at -30 °C. The mixture was stirred (700 rpm) at -30 °C for 24 h. After this, the reaction was quenched by the addition of Et_3N (100 µl), and the mixture was purified by column chromatography (EtOAc-hexane 5-10%), which afforded **5c** as a white solid in 20% yield, dr = 3:1 (7.0 mg, 0.02 mmol) along with unreacted starting material **1a,** 78% (21.0 mg, 0.078 mmol, *cis*/*trans* = 1.4:1).

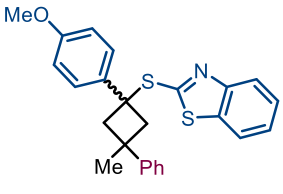

5c

^{1}H NMR (400 MHz, $CDCl_3$): (mixture of diastereomers, signals are reported as seen) δ 7.94 (d, *J* = 8.2 Hz, 0.24H), 7.74 – 7.63 (m, 2H), 7.50 (d, *J* = 7.0 Hz, 2H), 7.39 – 7.19 (m, 8H), 7.18 – 7.09 (m, 2H), 6.84 (d, *J* = 8.6 Hz, 1H), 6.69 (d, *J* = 7.1 Hz, 0.60H), 3.78 (s, 3H), 3.70 (s, 1H), 3.55 (d, *J* = 11.8 Hz, 2H), 3.33 (d, *J* = 12.3 Hz, 0.60H), 3.22 – 3.12 (m, 2.56H), 1.80 (s, 1H), 1.25 (s, 3H) ppm; **^{13}C NMR (101 MHz, $CDCl_3$)**: (mixture of diastereomers, signals are reported as seen) δ 164.0, 163.7, 158.5, 158.2, 153.4, 151.9, 151.0, 137.3, 136.3, 128.7, 128.4, 128.3, 128.0, 126.0, 125.9, 125.6, 125.0, 124.9, 124.7, 124.6, 122.4, 121.0, 120.9, 113.6, 113.3, 55.3, 55.2, 51.8, 50.8, 48.4, 48.2, 38.0, 37.5, 32.9, 32.0 ppm; **IR (ATR):** 3057, 2953, 2834, 1607, 1580, 1510, 1455, 1424, 1372, 1298, 1251, 1177, 1093, 1032, 979, 816, 802, 756, 727, 700, 546, 430 cm^{-1} **HRMS (ESI)**: calcd. for $[C_{25}H_{23}NOS_2+Na]^+$, $[M+Na]^+$: 440.1113; found: 440.1113; ***R*f**: 0.34 (Hexane/EtOAc, 95:5); **m.p.**: 119–121°C.

Hammett analysis

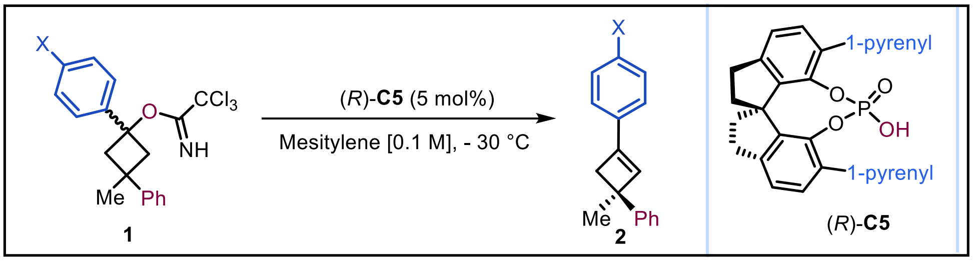

The Hammett analysis[21-23] was performed by comparing the relative rates of product formation of *p*-X substituted aryl 2,2,2-trichloroacetimidates **1** to that of the *p*-H substituted aryl **1**.

Sample preparation

An oven-dried 8 ml vial equipped with a magnetic stir bar was charged with both the *p*-X substituted aryl and *p*-H substituted aryl substrates **1** (0.05 mmol, 1.0 equiv. each). The vial was evacuated and backfilled with argon three times. Then, 350 µl of mesitylene was added, and the mixture was stirred at -30 °C for 20 minutes. Next, the catalyst (*R*)-**C5** (2.5 µmol, 0.05 equiv.) dissolved in 150 µL of mesitylene was added at -30 °C. The reaction mixture was stirred at 700 rpm at -30 °C for 1 hour. After this, the reaction was quenched by adding Et_3N (50 µl). The solution was transferred to a 50 mL round-bottom flask with toluene, and the solvent was removed under

reduced pressure. After drying, the residue was dissolved in C_6D_6 and transferred to an NMR tube for acquisition of the ^1H NMR spectrum.

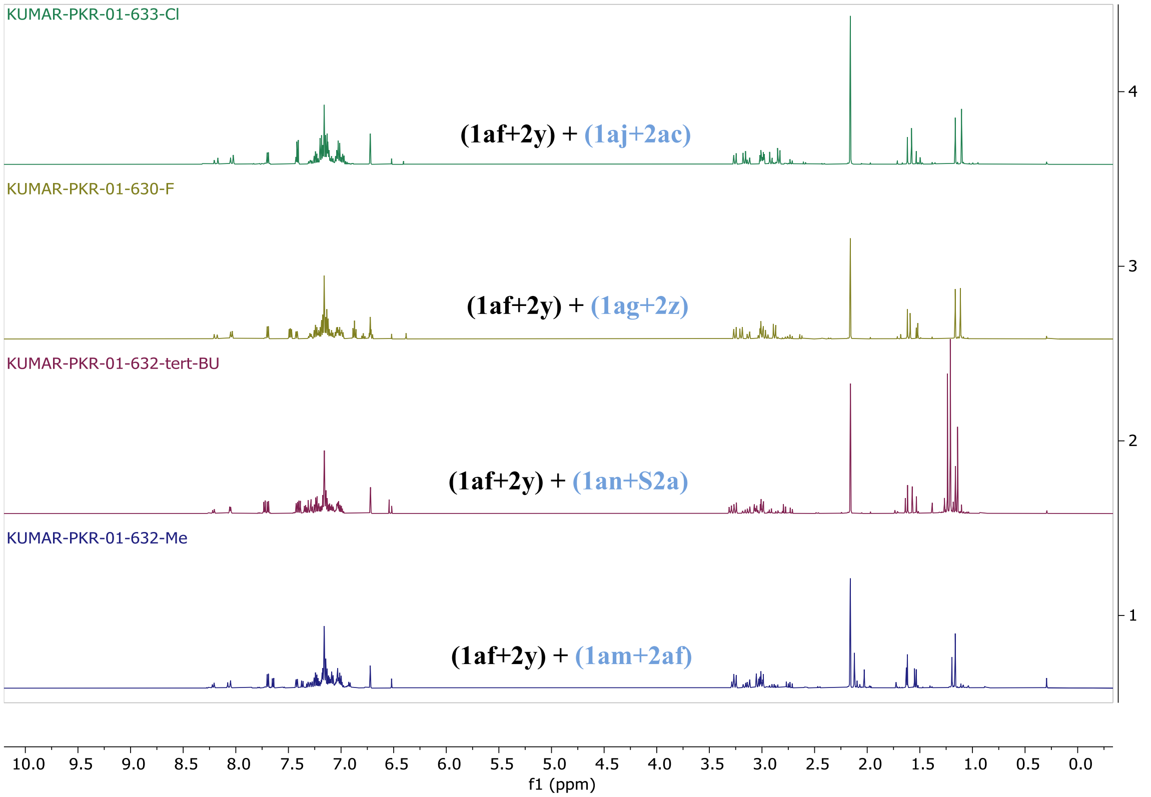

Figure 39. ^1H NMR spectra of the crude reaction mixtures obtained using the indicated mixtures of substrates.

All relevant parameters used for the analysis are given as follows:

substrate	*p*-substituent	σ_p	σ_p^+	log (k_X/k_H)
**1af**	X = H	0.000	0.000	0.000
**1am**	X = Me	-0.170	-0.311	0.045
**1an**	X = *t*Bu	-0.197	-0.260	0.283
**1ag**	X = F	0.060	-0.073	0.146
**1aj**	X = Cl	0.230	0.110	-0.367

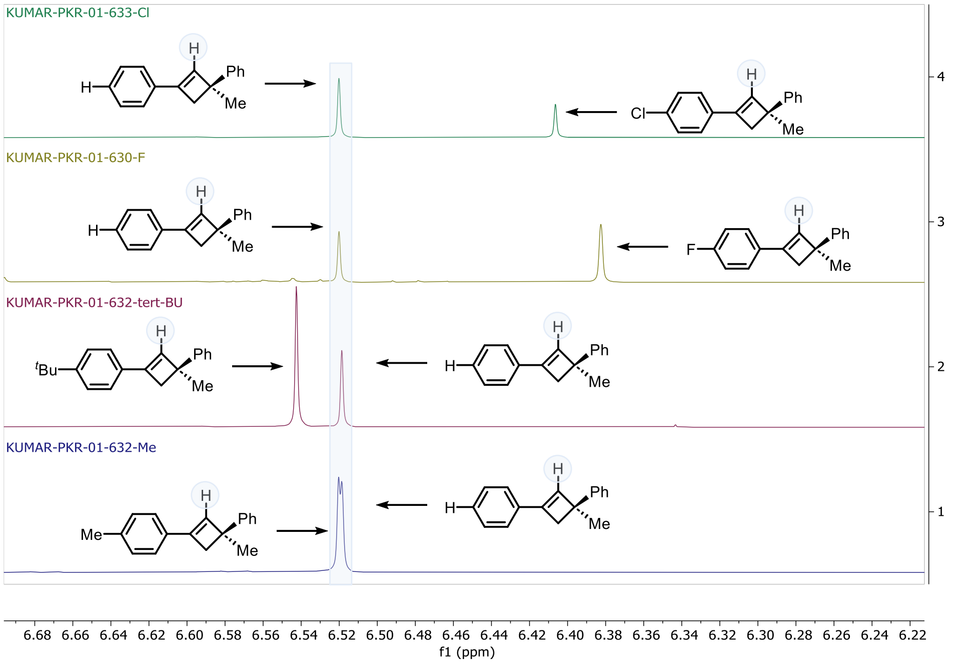

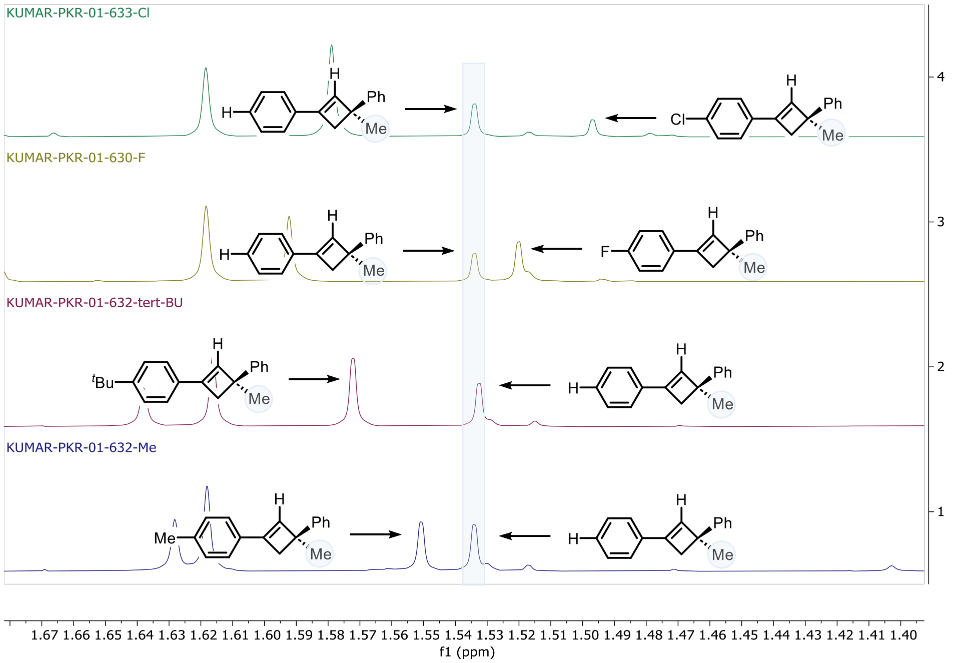

Figure 40. Expanded regions of the ^{1}H NMR spectra presented in Figure S39.

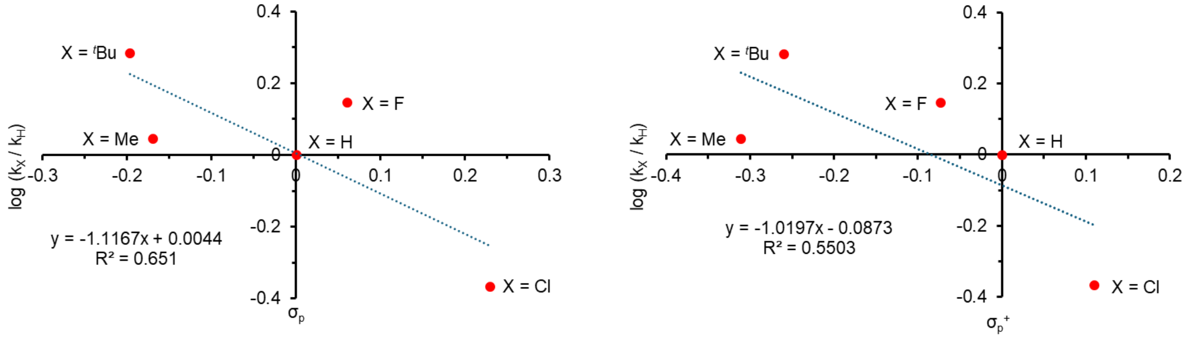

Figure 41. Hammett analysis.

No linear correlation was observed through plotting log (k_X/k_H) against either σ_p or σ_p^+.

Racemization studies

To evaluate the extent of product racemization during the reaction, we subjected enantioenriched cyclobutene **2a** to our reaction conditions, which resulted in slight erosion of enantiomeric excess at –30 °C. However, complete racemization took place at room temperature.

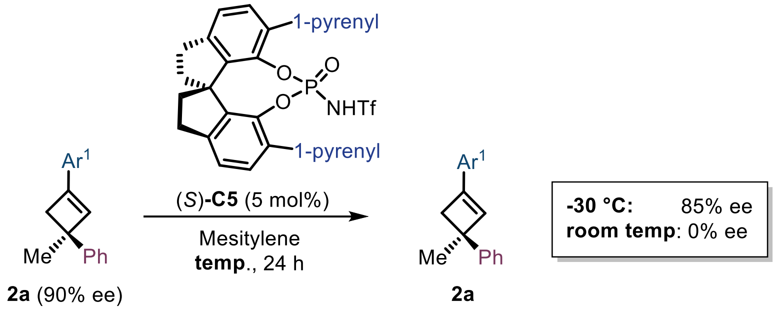

Non-Linear effect analysis

Non-Linear effect (NLE) study on the enantioselective elimination of cyclobutanol

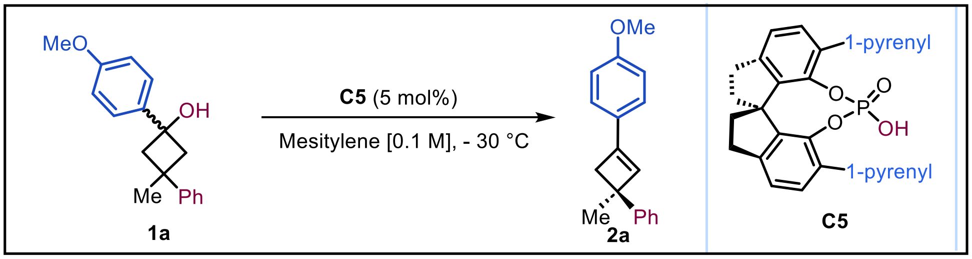

According to general procedure D for the enantioselective elimination of cyclobutanols, NLE experiments were carried out using cyclobutanol **1a** and racemic and scalemic mixtures of catalyst **C5** (5 mol%) at -30 °C for 24 h. The enantiomeric excess of the product **2a** was subsequently plotted against the enantiomeric excess of the catalyst.

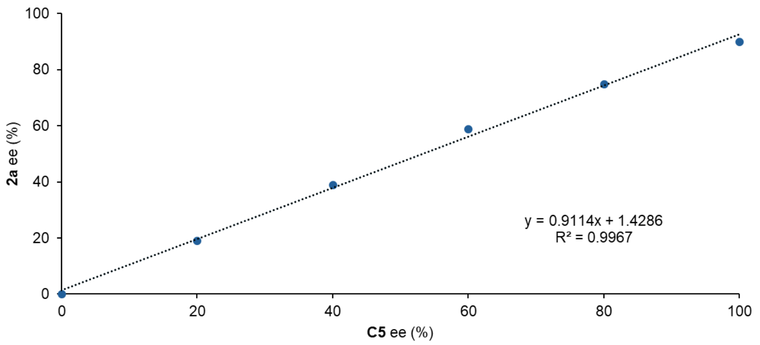

Figure S42. Non-linear effect study for the formation of product **2a** with 5 mol% of catalyst **C5**.

Non-Linear effect study on the enantioselective elimination of trichlorocetimidate

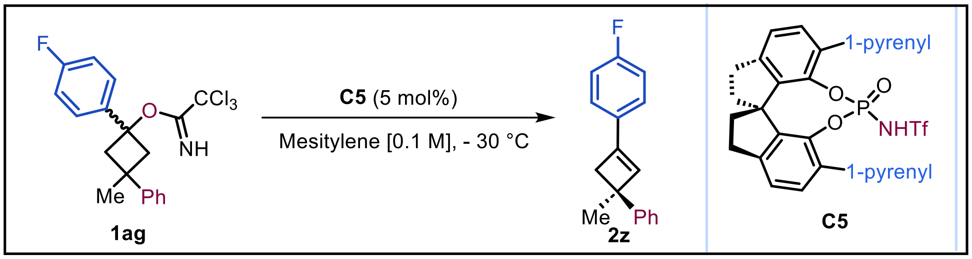

According to general procedure E for enantioselective elimination of cyclobutyl trichloroacetimidates, NLE experiments were carried out using cyclobutyl trichloroacetimidate **1ag** and racemic and scalemic mixtures of catalyst **C5** (5 mol%) at -30 °C for 24 h. The enantiomeric excess of the product **2z** was subsequently plotted against the enantiomeric excess of the catalyst.

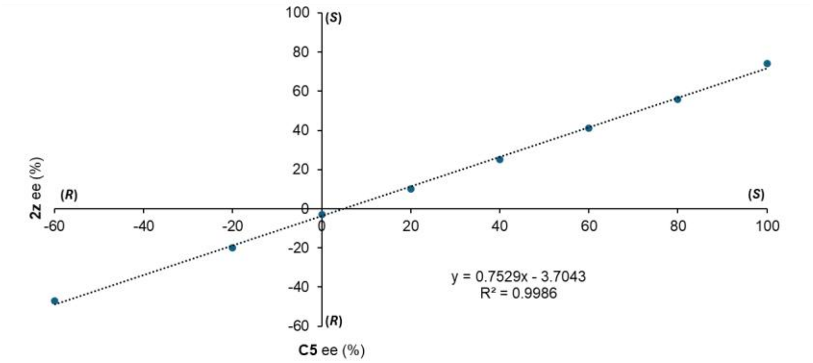

Figure S43. Non-linear effect study for the formation of product **2z** with 5 mol% of catalyst **C5**.

References

1. Ano, Y.; Takahashi, D.; Yamada, Y.; Chatani, N. *ACS Catal.* **2023**, *13*, 2234–2239.
2. Matsuda, T.; Yuihara, I. *Chem. Commun*., **2015**, *51*, 7393–7396.
3. Li, R.; Shi, X.; Zhao, D. *Chin. J. Chem*. **2023**, *41*, 1679–1683.
4. Ma, Y.; Ai, Y.; Yu, S. *Synlett* **2023**, *34*, 359–363.
5. Sietmann, J.; Tenberge, M.; Wahl, J. M. *Angew. Chem. Int. Ed*. **2023**, *62*, e202215381.
6. Capel, E.; Rodríguez-Rodríguez, M.; Uria, U.; Pedron, M.; Tejero, T.; Vicario, J. L.; Merino, P. *J. Org. Chem*. **2022**, *87*, 693−707.
7. Shuai, B.; Fang, P.; Mei, T.-S. *Synlett* **2021**, *32*, 1637–1641.
8. (a) *Organic Syntheses, Coll. Vol. 8, p.306 (1993); Vol. 69, p.199 (1990). (b)* Guo, J.; Xu, X.; Xing, Q.; Gao, Z.; Gou, J.; Yu, B. *Org. Lett*. **2018**, *20*, 7410–7414.
9. (a) Krepski, L. R.; Hassner, A. *J. Org. Chem*. **1978**, *43*, 2879–2881. (b) Hassner, A.; Dillon, J. L. *J. Org. Chem*. **1983**, *48*, 3382–3386.
10. Fan, H.-k.; Yang, S.; Li, J.-h.; Teng, Q.-q.; Chen, M. *Eur. J. Org. Chem*. **2022**, e202201218.
11. Seiser, T.; Roth, O. A.; Cramer, N. *Angew. Chem. Int. Ed*. **2009**, 48, 6320–6323.
12. Brunetti, A.; Pintus, A.; Lombardi, L.; Kovtun, A.; Mascietti, F.; Bruno, F.; Ravera, E.; Melucci, M.; Bertuzzi, G.; Bandini, M. *Chin. J. Chem*. **2023**, *41*, 1333–1340.
13. Yan, X.; Zhu, Y.; Xia, Y. *Angew. Chem. Int. Ed.* **2023**, *62*, e202304462.
14. Baumann, A. N.; Schüppel, F.; Eisold, M.; Kreppel, A.; de Vivie-Riedle, R.; Didier, D. *J. Org. Chem*. **2018**, *83*, 4905–4921.
15. Wise, D. E.; Gogarnoiu, E. S.; Duke, A. D.; Paolillo, J. M.; Vacala, T. L.; Hussain, W. A.; Parasram, M. *J. Am. Chem. Soc*. **2022**, *144*, 15437−15442.
16. Ni, M.; Zhang, J.; Liang, X.; Jiang, Y.; Loh, T.-P. *Chem. Commun*., **2017**, *53*, 12286–12289.
17. Burés, J. *Angew. Chem., Int. Ed.* **2016**, *55*, 16084−16087.
18. Fletcher, A. N.; Heller, C. A. *J. Phys. Chem. A* **1967**, *71*, 3742−3756.
19. Kötzner, L.; Webber, M. J.; Martínez, A.; Fusco, C.; De; List, B. *Angew. Chem., Int. Ed.* **2014**, *53*, 5202−5205.
20. Singleton, D. A.; Thomas, A. A. *J. Am. Chem. Soc.* **1995**, *117*, 9357–9358.
21. Hansch, C.; Leo, A.; Taft, R. W. *Chem. Rev*. **1991**, *91*, 165-195.
22. Maji, R.; Ghosh, S.; Grossmann, O.; Zhang, P.; Leutzsch, M.; Tsuji, N.; List, B. *J. Am. Chem. Soc.* **2023**, *145*, 8788-8793.
23. Lin, S.-L.; Chen, Y.-H.; Liu, H.-H.; Xiang, S. H.; Tan, B. *J. Am. Chem. Soc.* **2023**, *145*, 21152−21158.

NMR spectra

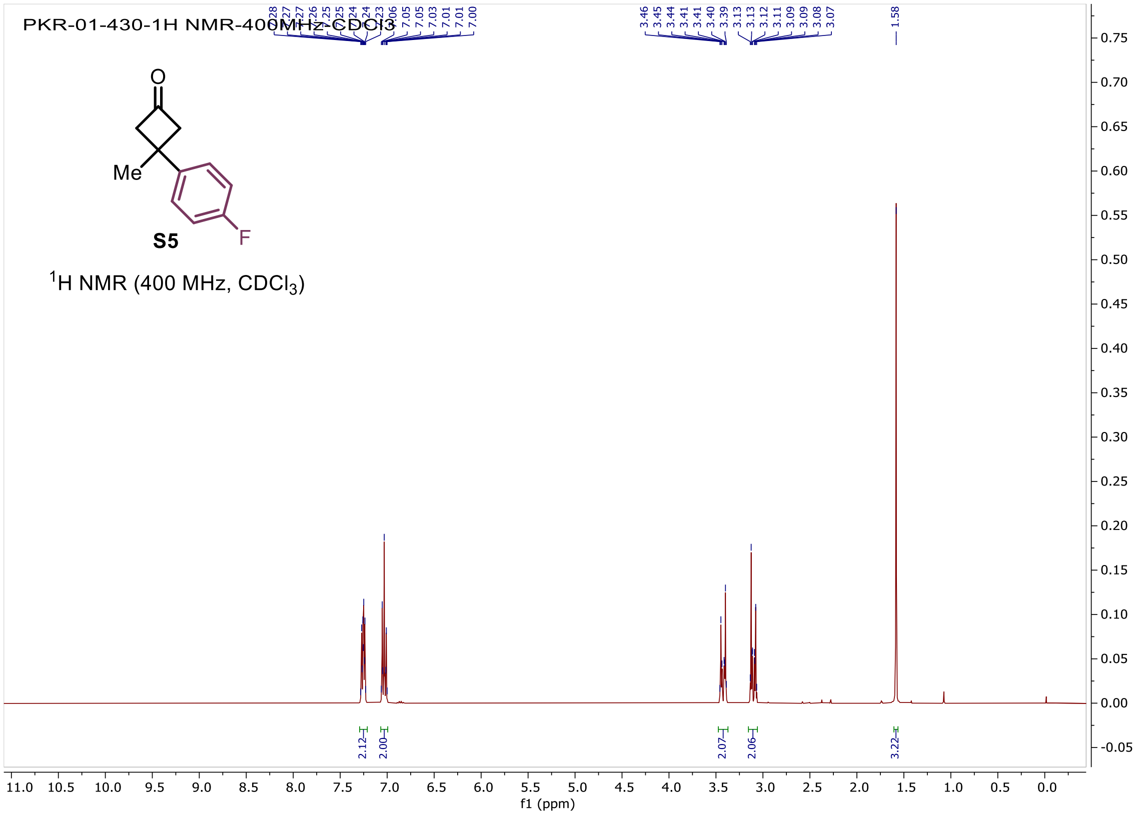
PKR-01-430-1H NMR-400MHz-CDCl3
O
Me
F
S5
1H NMR (400 MHz, CDCl3)
f1 (ppm)

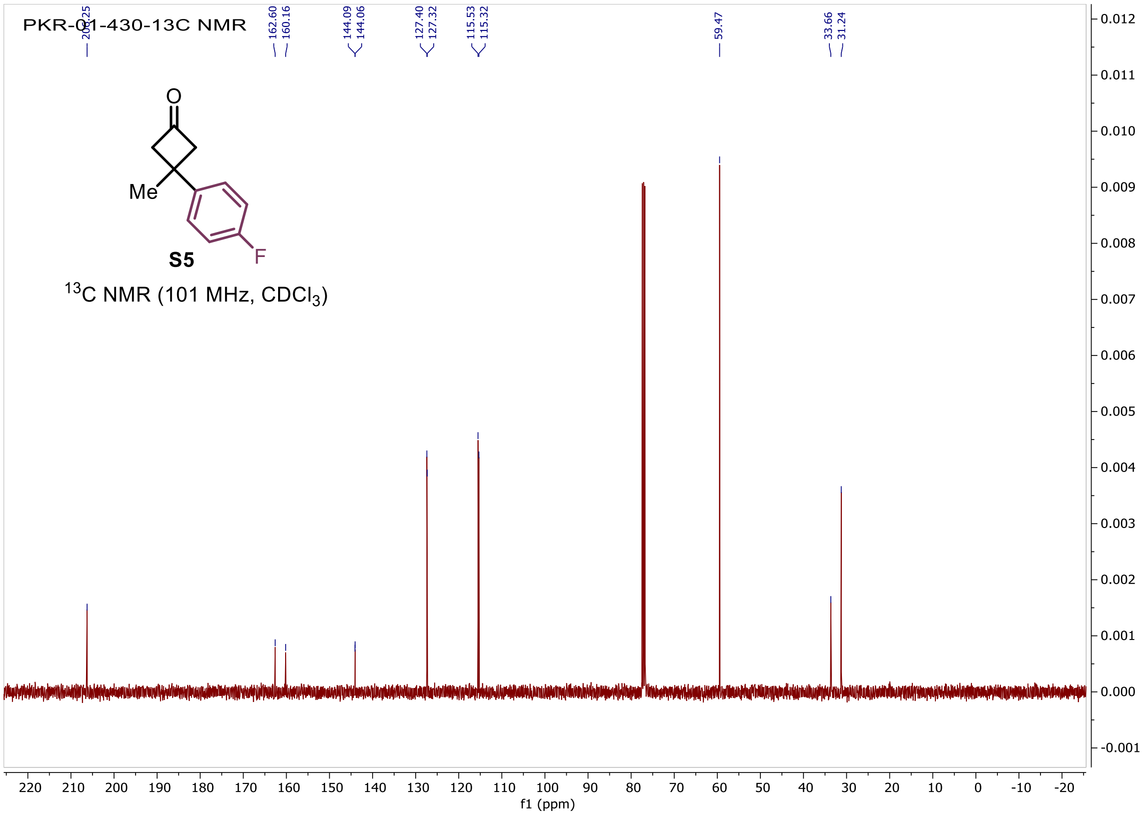
PKR-01-430-13C NMR
O
Me
F
S5
13C NMR (101 MHz, CDCl3)
f1 (ppm)

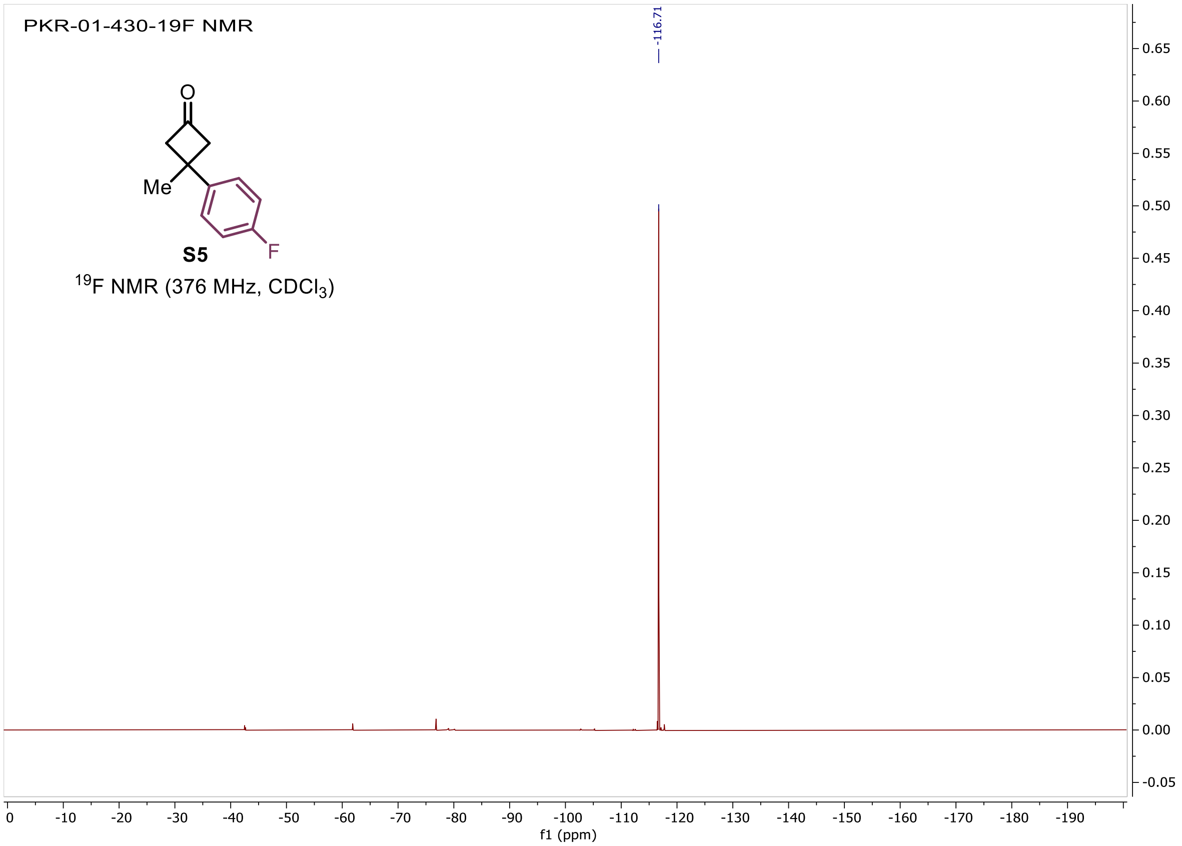
PKR-01-430-19F NMR
-116.71
O
Me
F
S5
19F NMR (376 MHz, CDCl3)
f1 (ppm)

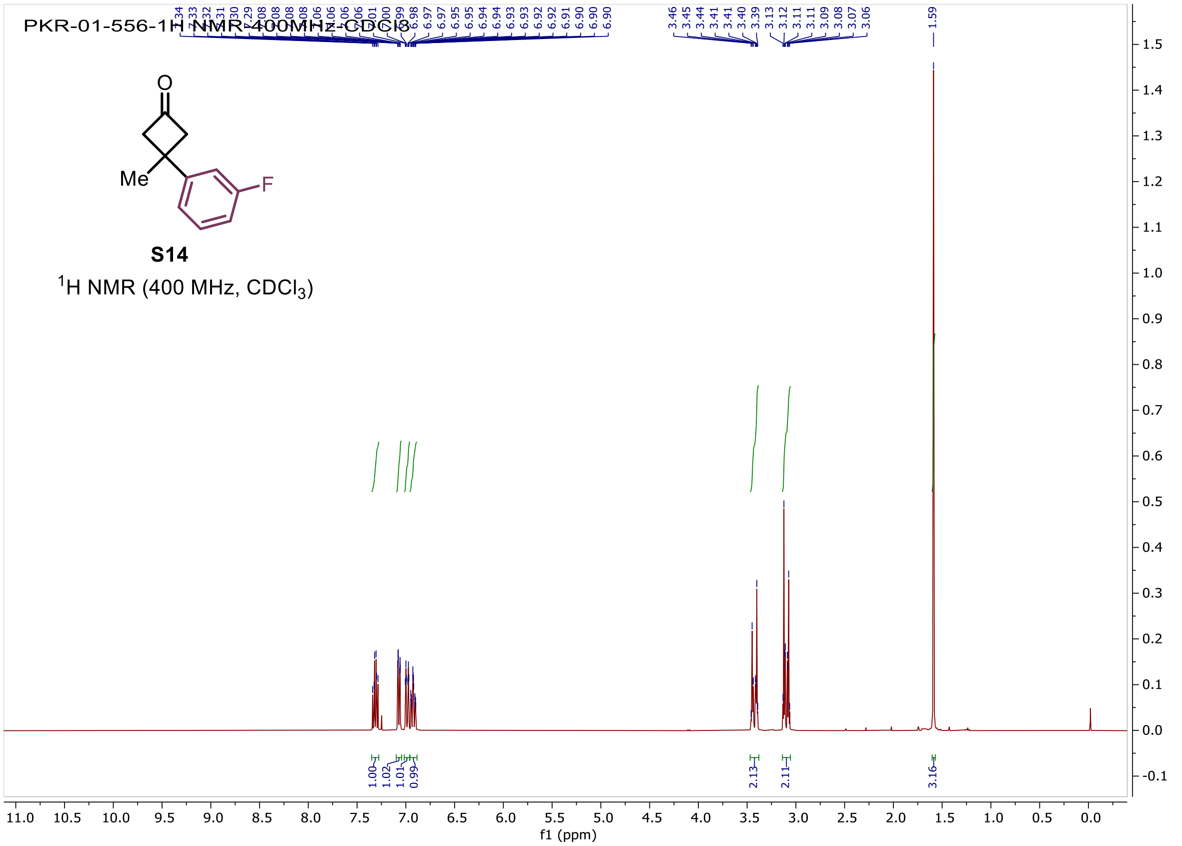
PKR-01-556-1H NMR-400MHz-CDCl3
1.59
O
Me
F
S14
1H NMR (400 MHz, CDCl3)
1.00
1.02
1.01
0.99
2.13
2.11
3.16
f1 (ppm)

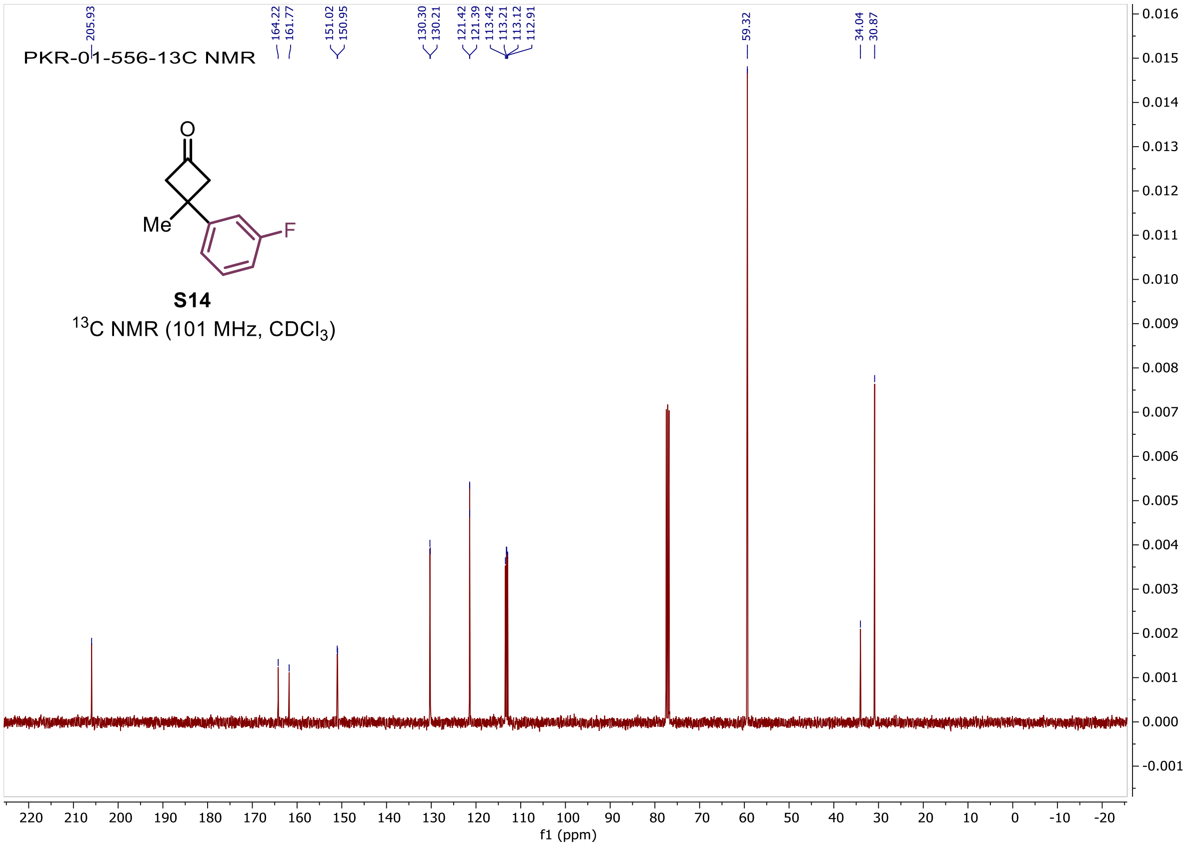
PKR-01-556-13C NMR
205.93
164.22
161.77
151.02
150.95
130.30
130.21
121.42
121.39
113.42
113.21
113.12
112.91
59.32
34.04
30.87
O
Me
F
S14
^{13}C NMR (101 MHz, CDCl$_3$)
f1 (ppm)

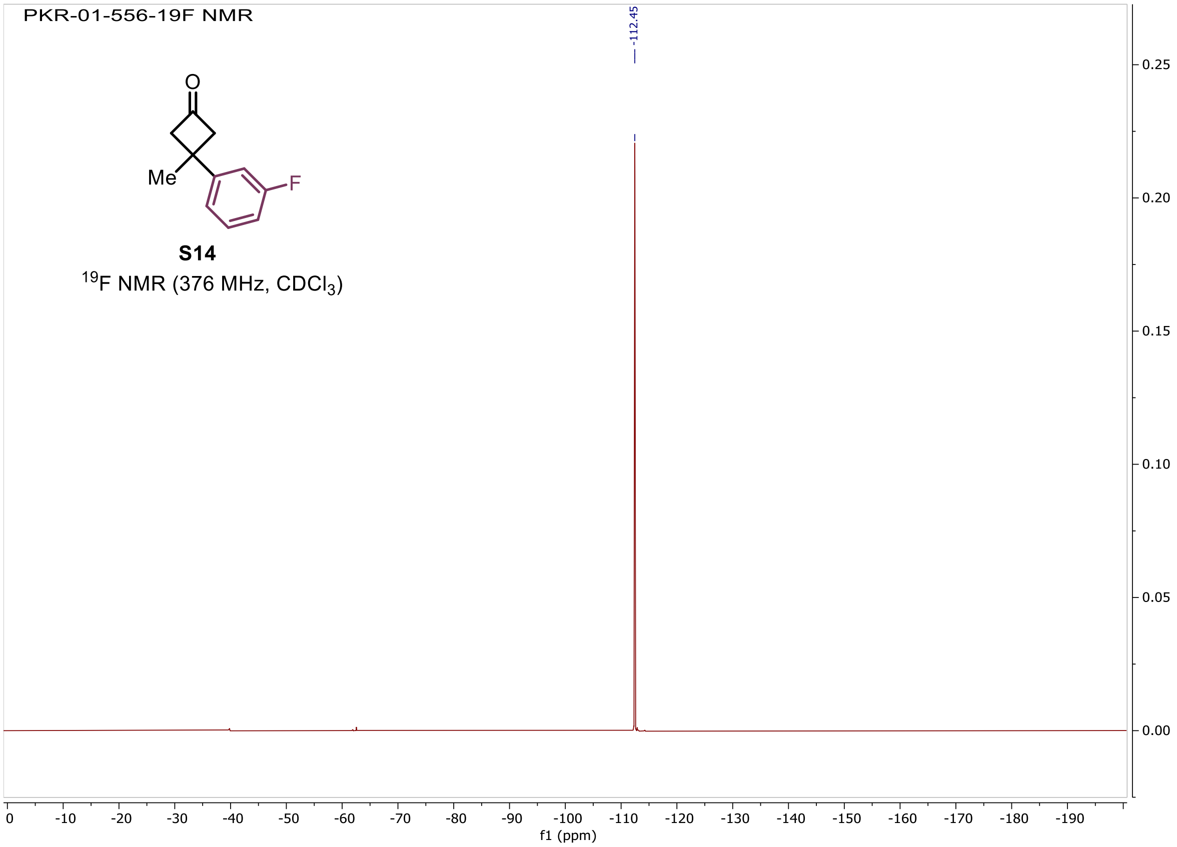
PKR-01-556-19F NMR
-112.45
O
Me
F
S14
^{19}F NMR (376 MHz, CDCl$_3$)
f1 (ppm)

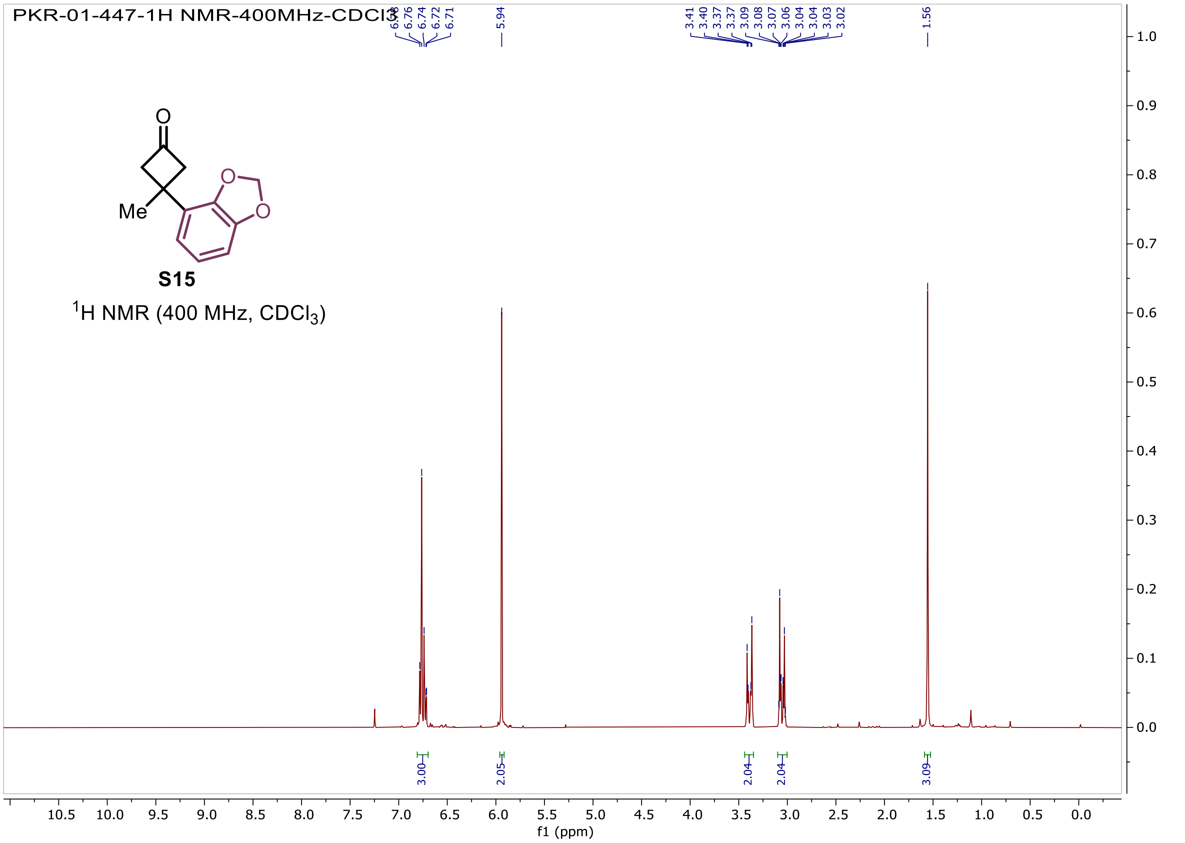
PKR-01-447-1H NMR-400MHz-CDCl3
S15
1H NMR (400 MHz, CDCl3)
Me
O
6.76
6.74
6.72
6.71
5.94
3.41
3.40
3.37
3.37
3.09
3.08
3.07
3.06
3.04
3.04
3.03
3.02
1.56
3.00
2.05
2.04
2.04
3.09
f1 (ppm)

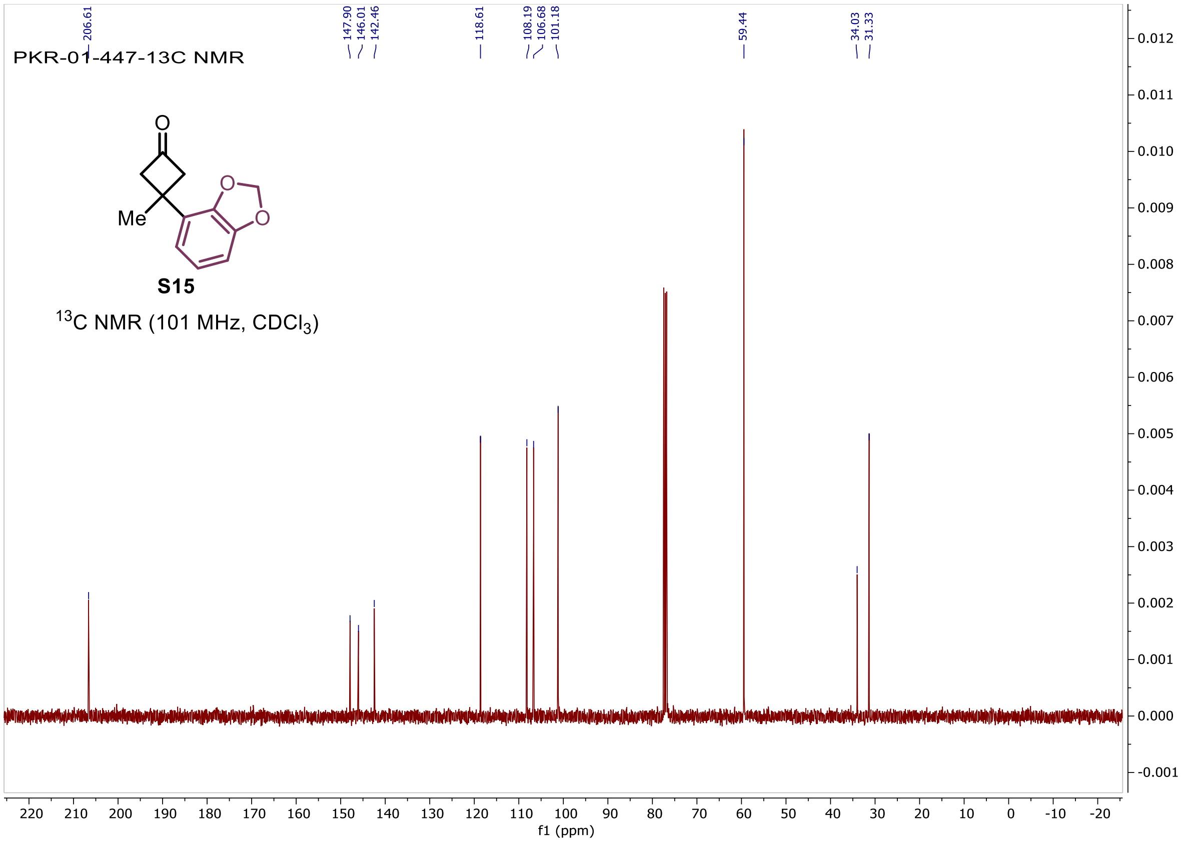
PKR-01-447-13C NMR
S15
13C NMR (101 MHz, CDCl3)
Me
O
206.61
147.90
146.01
142.46
118.61
108.19
106.68
101.18
59.44
34.03
31.33
f1 (ppm)

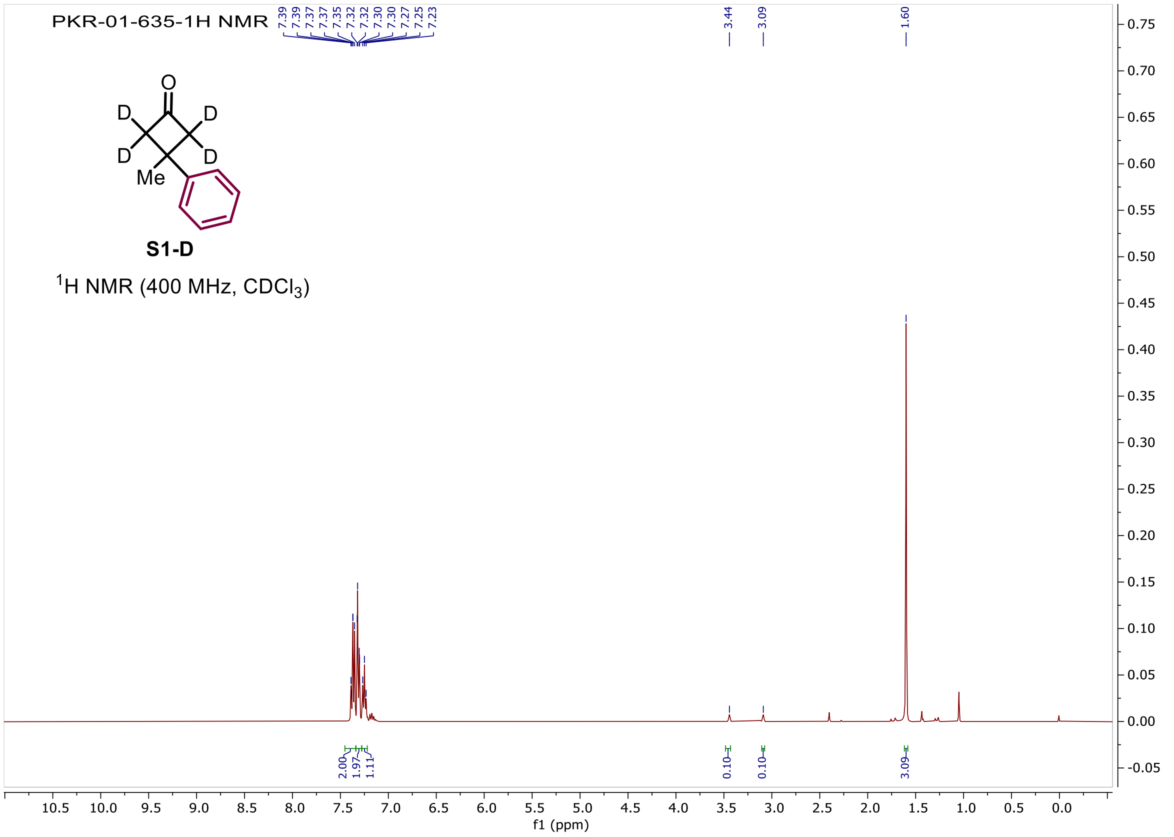
PKR-01-635-1H NMR
S1-D
^{1}H NMR (400 MHz, $CDCl_3$)
7.39 7.39 7.37 7.37 7.35 7.32 7.32 7.30 7.30 7.27 7.25 7.23
3.44
3.09
1.60
2.00 1.97 1.11
0.10
0.10
3.09
f1 (ppm)

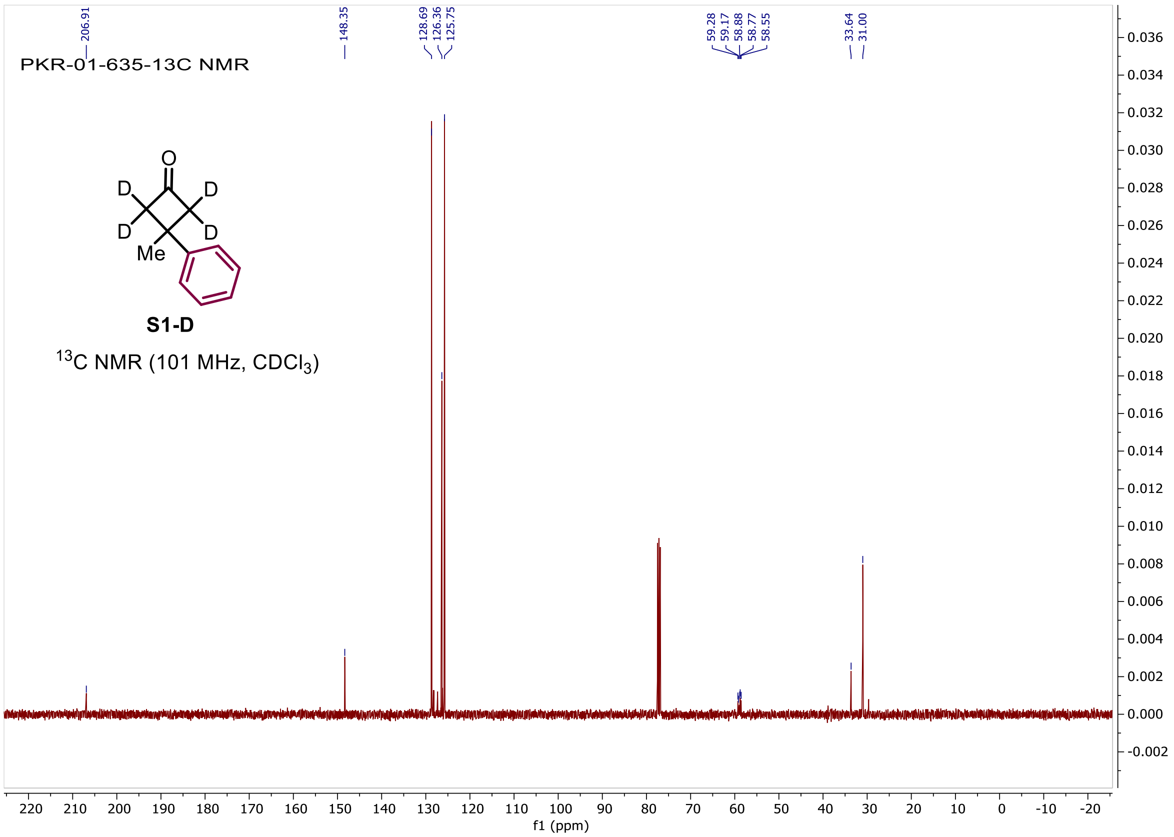
PKR-01-635-13C NMR
S1-D
^{13}C NMR (101 MHz, $CDCl_3$)
206.91
148.35
128.69 126.36 125.75
59.28 59.17 58.88 58.77 58.55
33.64 31.00
f1 (ppm)

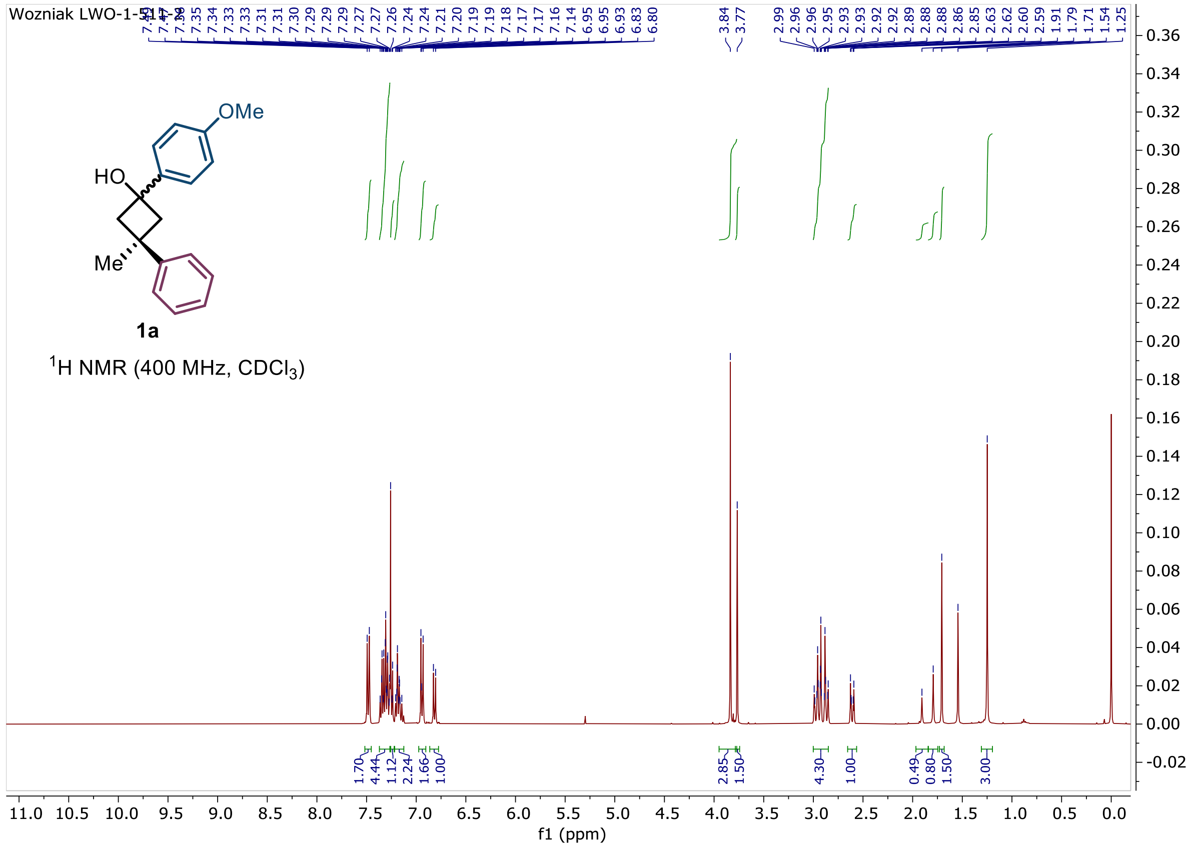

Wozniak LWO-1-517-3
OMe
HO
Me
1a
^{1}H NMR (400 MHz, CDCl$_3$)
f1 (ppm)

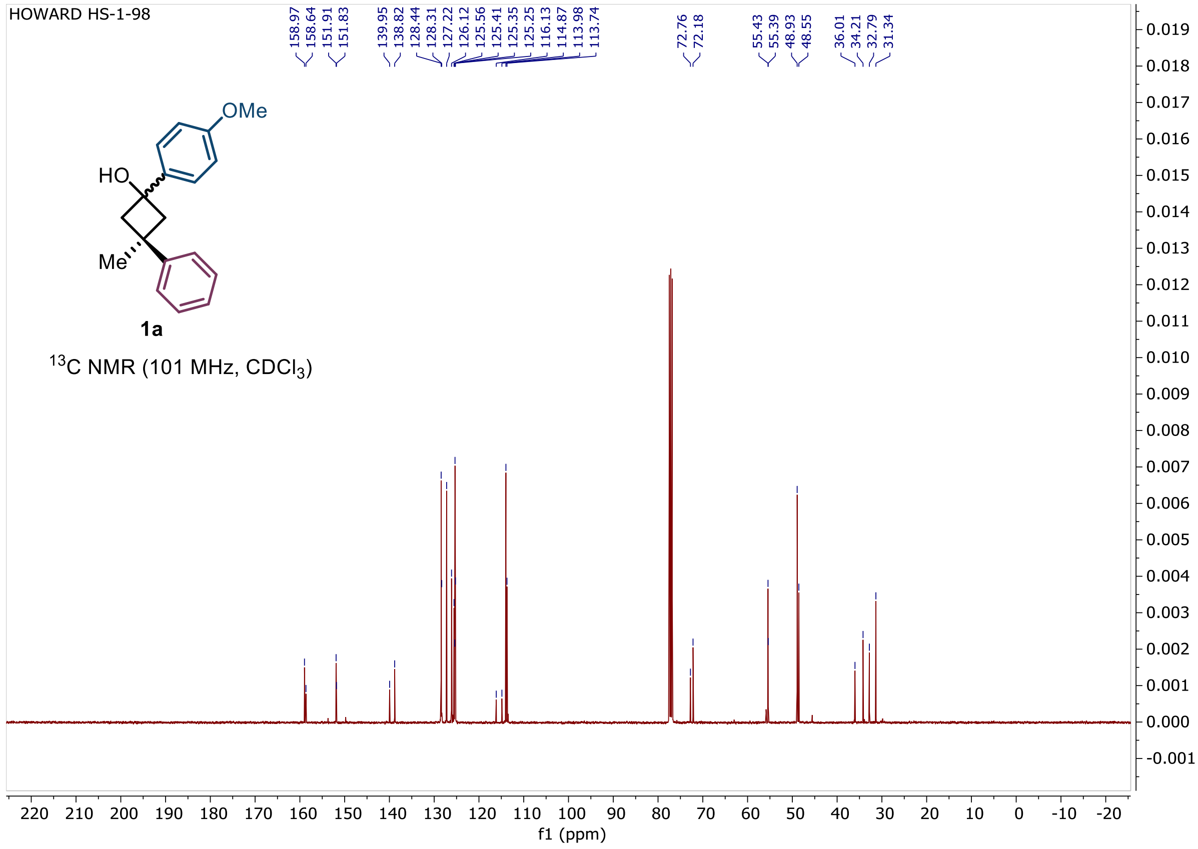

HOWARD HS-1-98
158.97
158.64
151.91
151.83
139.95
138.82
128.44
128.31
127.22
126.12
125.56
125.41
125.35
125.25
116.13
114.87
113.98
113.74
72.76
72.18
55.43
55.39
48.93
48.55
36.01
34.21
32.79
31.34
OMe
HO
Me
1a
^{13}C NMR (101 MHz, CDCl$_3$)
f1 (ppm)

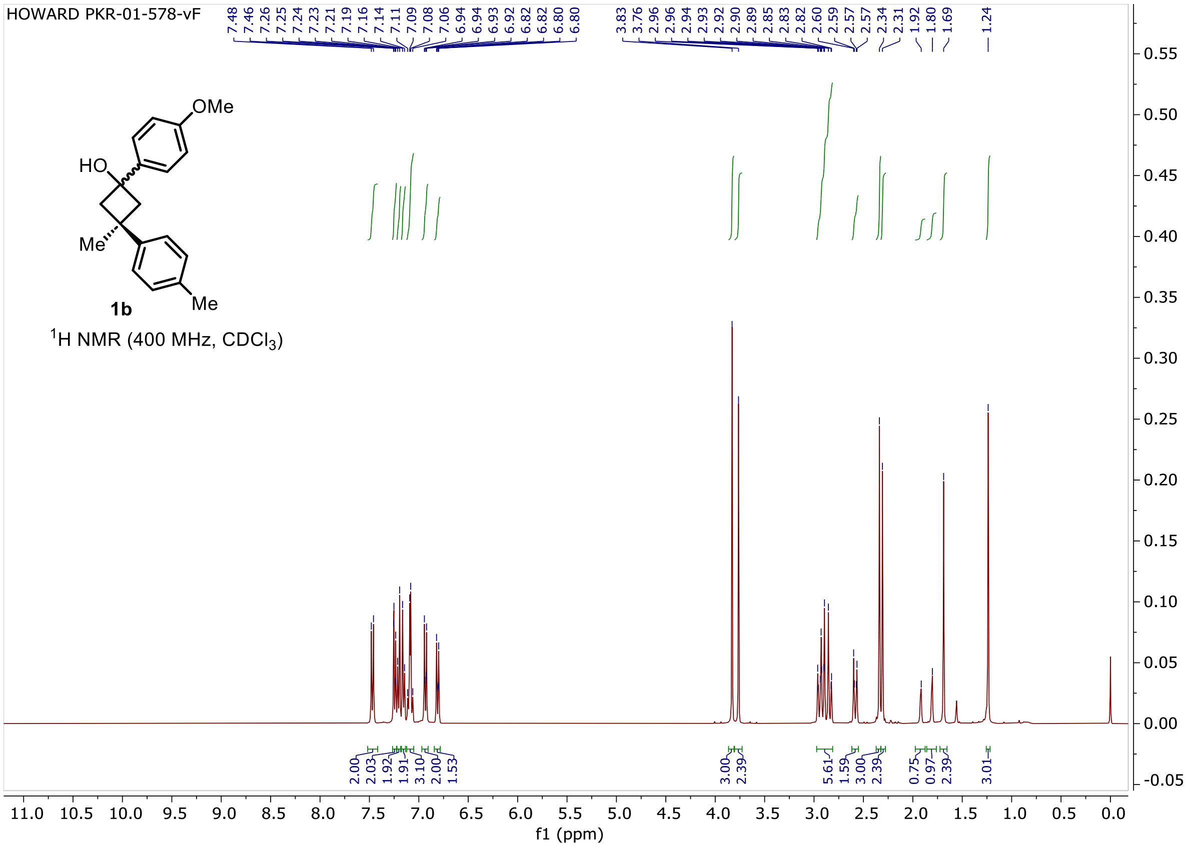
HOWARD PKR-01-578-vF
7.48 7.46 7.26 7.25 7.24 7.23 7.21 7.19 7.16 7.14 7.11 7.09 7.08 7.06 6.94 6.94 6.93 6.92 6.82 6.82 6.80 6.80
3.83 3.76 2.96 2.96 2.94 2.93 2.92 2.90 2.89 2.85 2.83 2.82 2.60 2.59 2.57 2.57 2.34 2.31 1.92 1.80 1.69 1.24
OMe
HO
Me
Me
1b
1H NMR (400 MHz, CDCl3)
2.00 2.03 1.92 1.91 3.10 2.00 1.53 3.00 2.39 5.61 1.59 3.00 2.39 0.75 0.97 2.39 3.01
f1 (ppm)

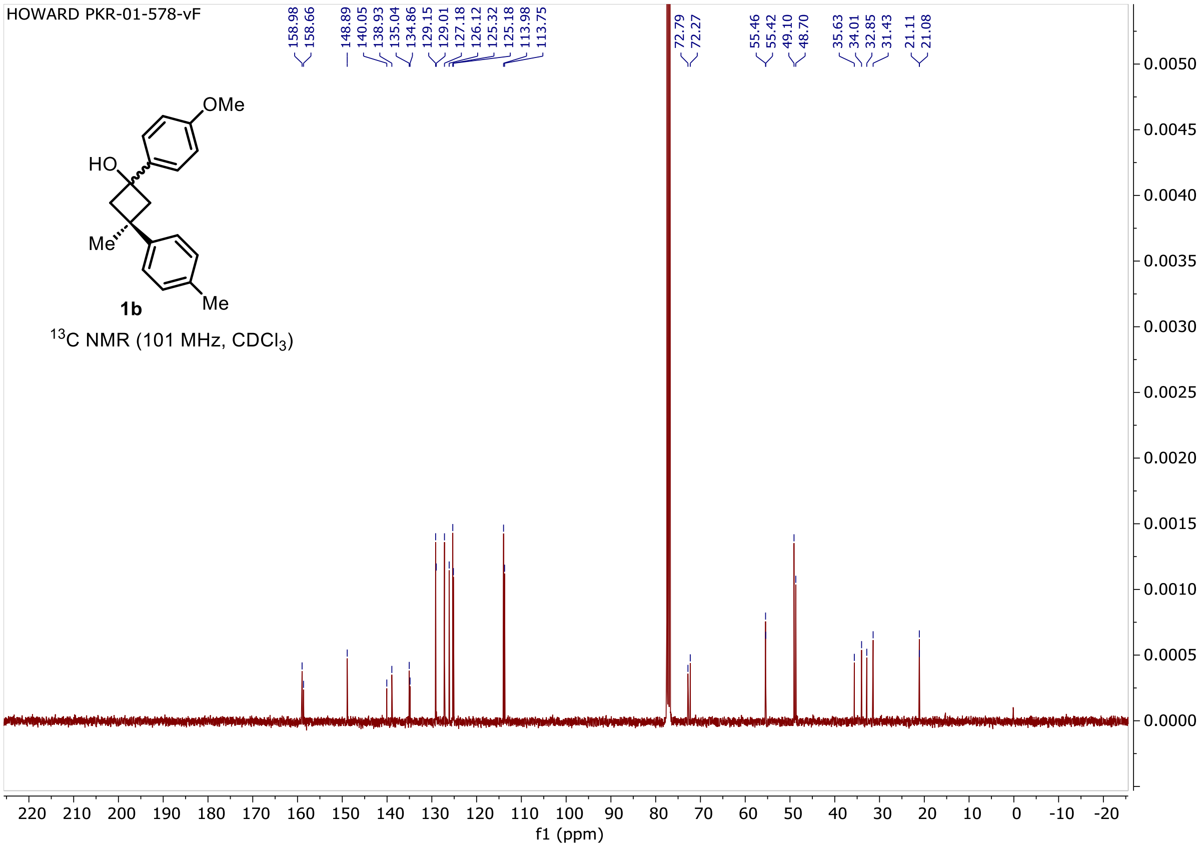
HOWARD PKR-01-578-vF
158.98 158.66 148.89 140.05 138.93 135.04 134.86 129.15 129.01 127.18 126.12 125.32 125.18 113.98 113.75
72.79 72.27 55.46 55.42 49.10 48.70 35.63 34.01 32.85 31.43 21.11 21.08
OMe
HO
Me
Me
1b
13C NMR (101 MHz, CDCl3)
f1 (ppm)

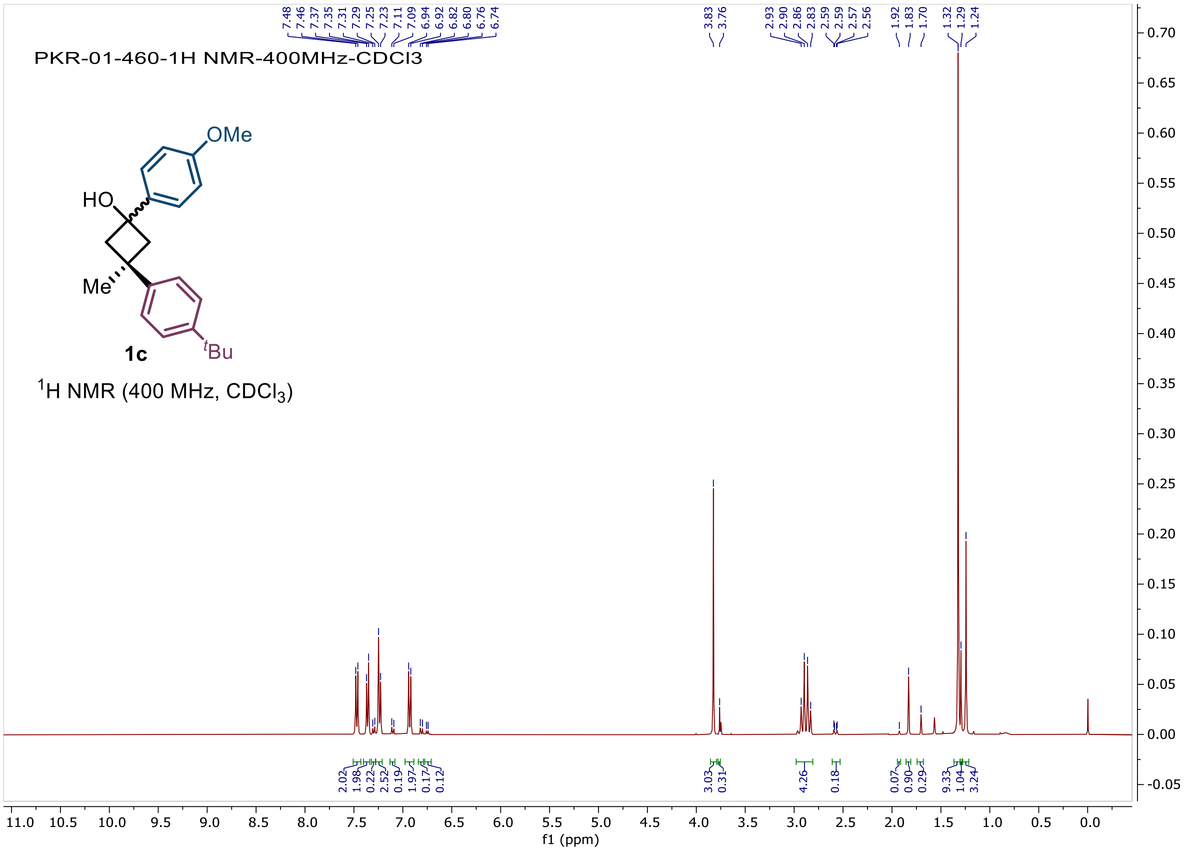
PKR-01-460-1H NMR-400MHz-CDCl3
OMe
HO
Me
tBu
1c
1H NMR (400 MHz, CDCl3)
7.48 7.46 7.37 7.35 7.31 7.29 7.25 7.23 7.11 7.09 6.94 6.92 6.82 6.80 6.76 6.74
3.83 3.76
2.93 2.90 2.86 2.83 2.59 2.59 2.57 2.56
1.92 1.83 1.70
1.32 1.29 1.24
2.02 1.98 0.22 2.52 0.19 1.97 0.17 0.12
3.03 0.31
4.26
0.18
0.07 0.90 0.29
9.33 1.04 3.24
f1 (ppm)

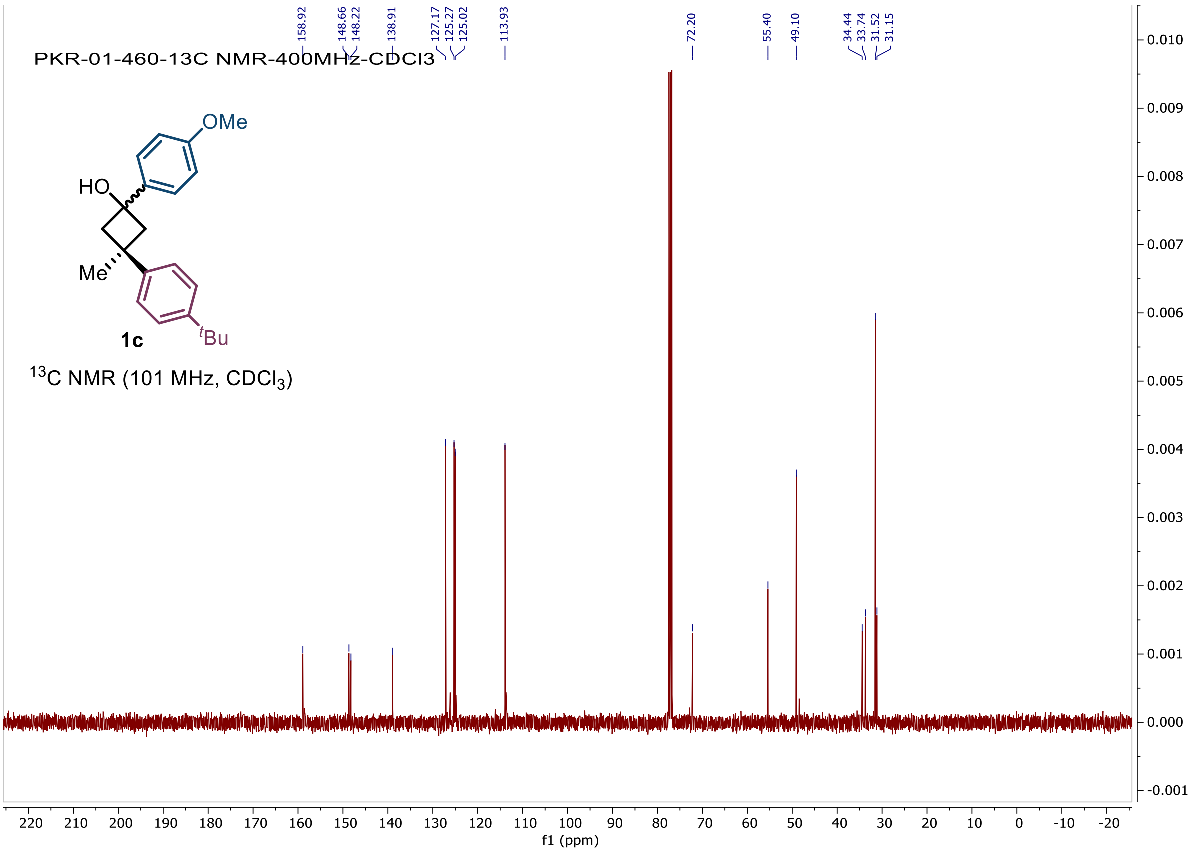
PKR-01-460-13C NMR-400MHz-CDCl3
OMe
HO
Me
tBu
1c
13C NMR (101 MHz, CDCl3)
158.92
148.66 148.22
138.91
127.17 125.27 125.02
113.93
72.20
55.40
49.10
34.44 33.74 31.52 31.15
f1 (ppm)

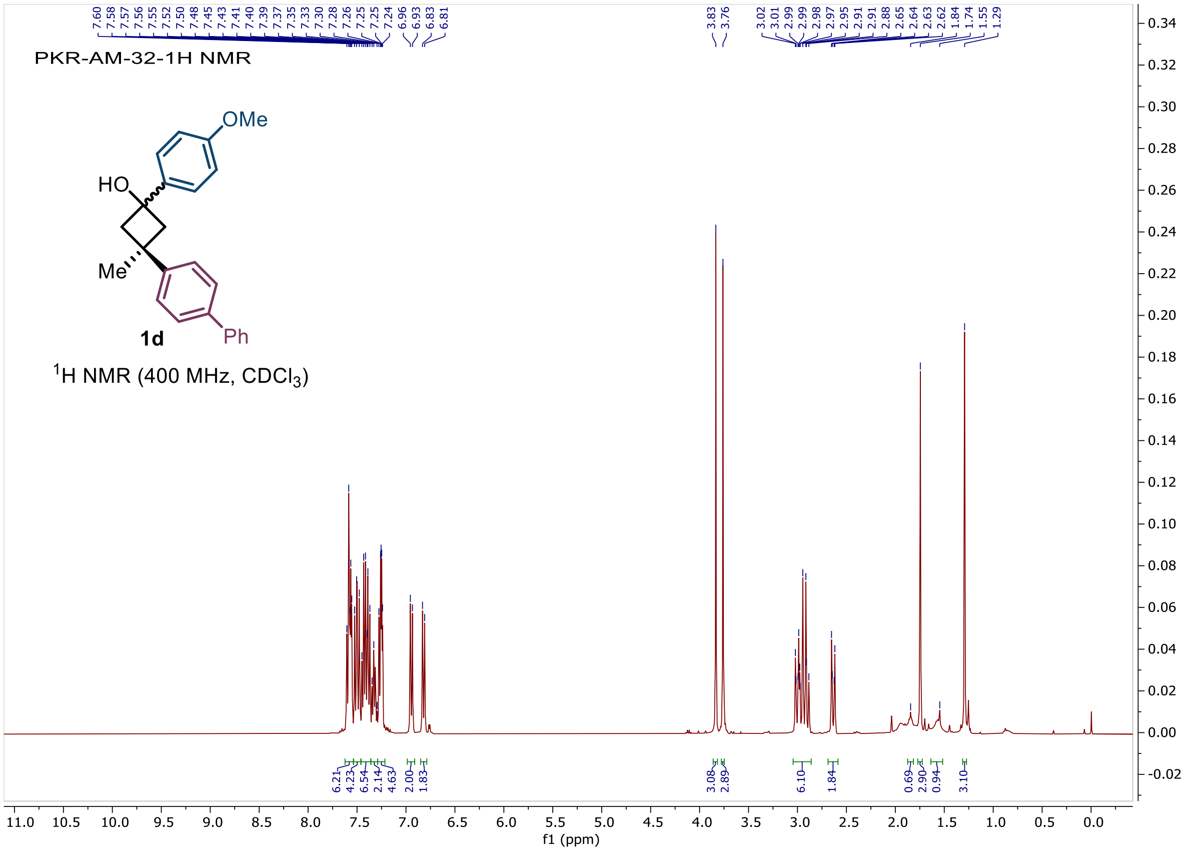
PKR-AM-32-1H NMR
OMe
HO
Me
Ph
1d
1H NMR (400 MHz, CDCl3)
f1 (ppm)

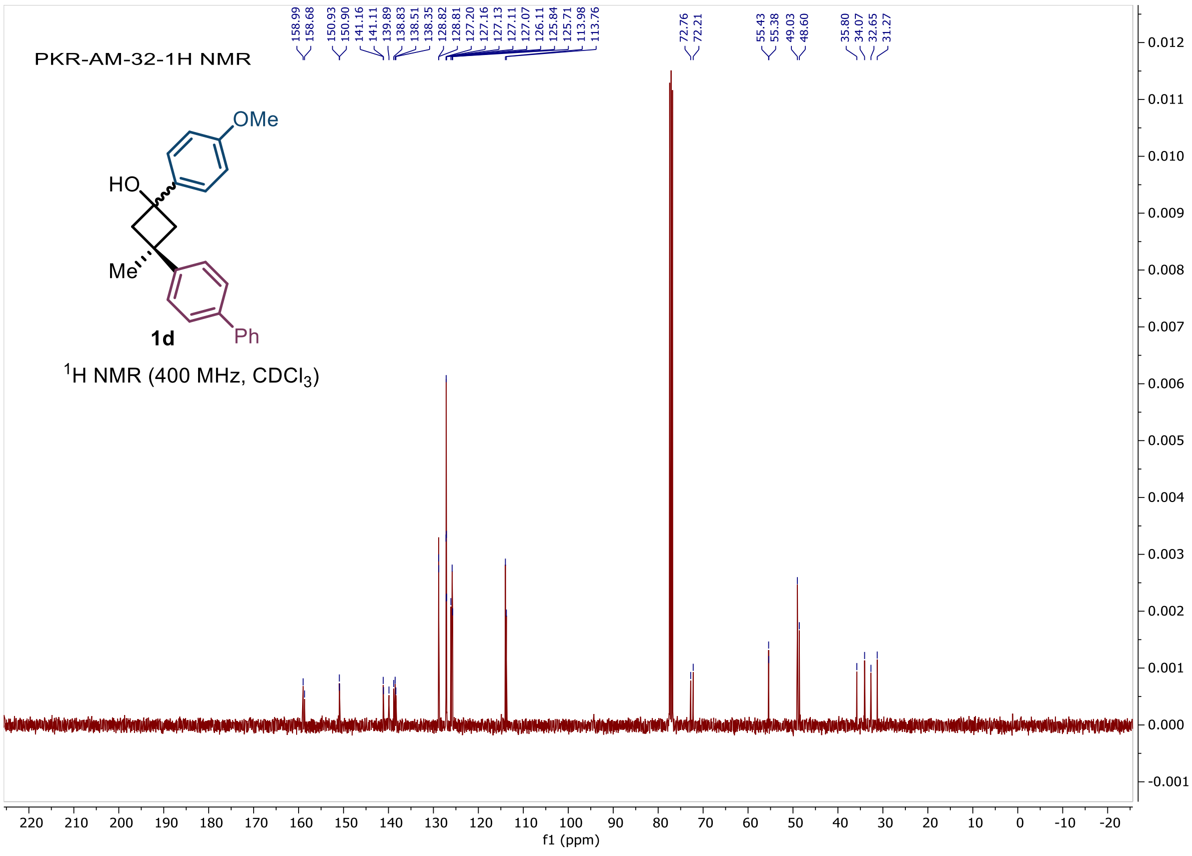
PKR-AM-32-1H NMR
OMe
HO
Me
Ph
1d
1H NMR (400 MHz, CDCl3)
f1 (ppm)

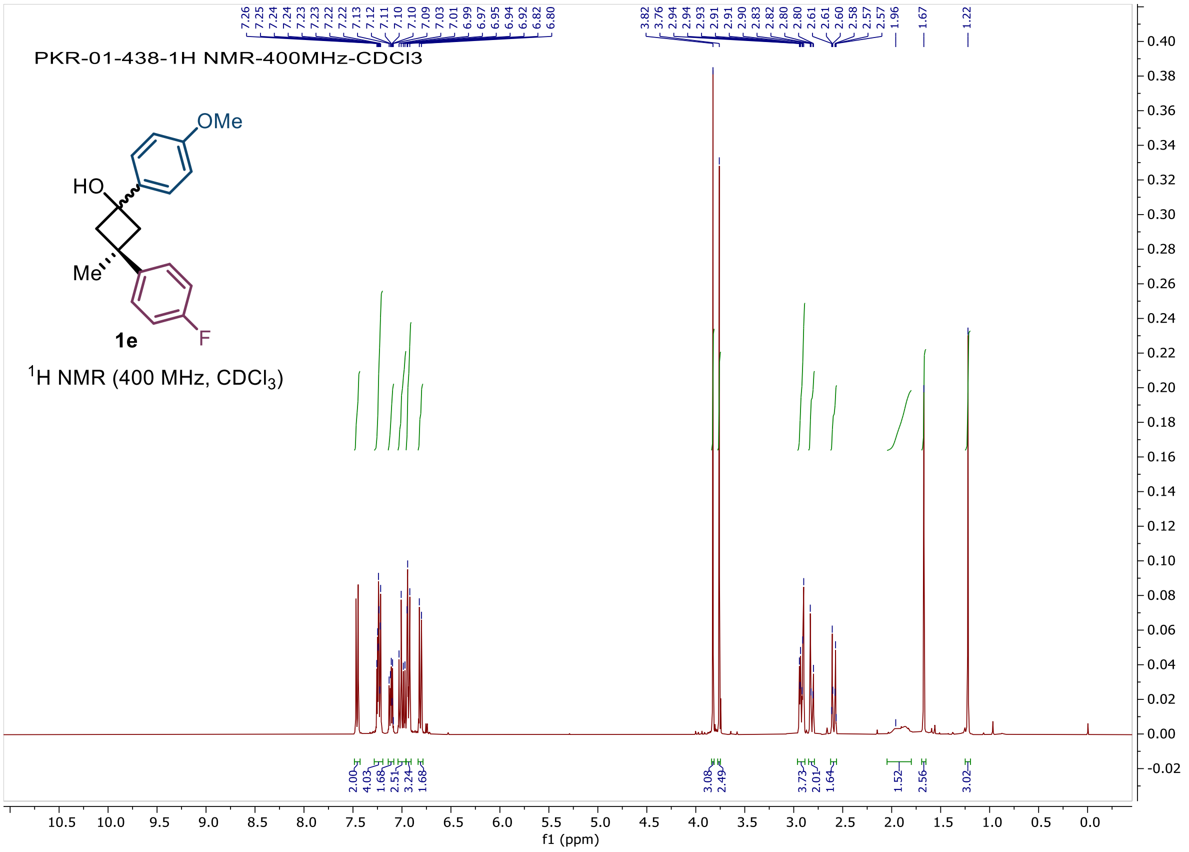
PKR-01-438-1H NMR-400MHz-CDCl3
OMe
HO
Me
F
1e
^{1}H NMR (400 MHz, CDCl$_3$)
f1 (ppm)

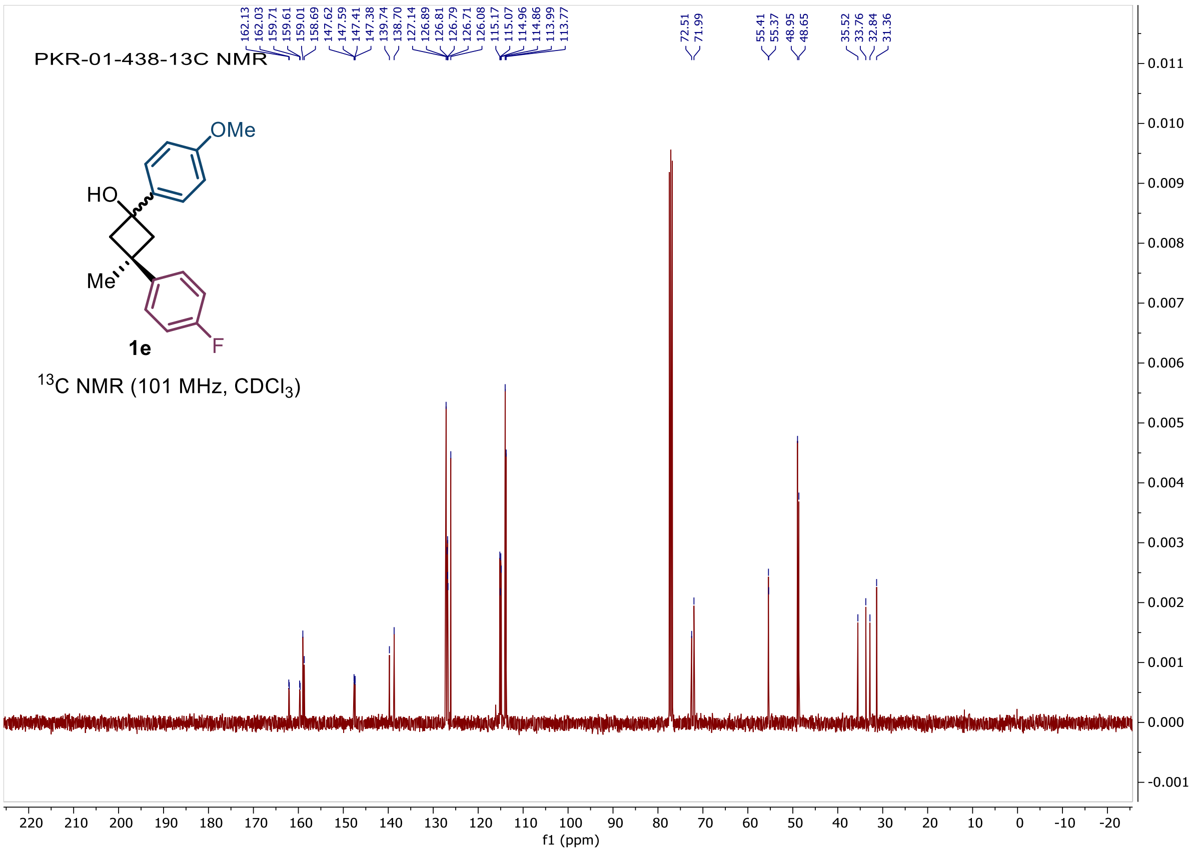
PKR-01-438-13C NMR
OMe
HO
Me
F
1e
^{13}C NMR (101 MHz, CDCl$_3$)
f1 (ppm)

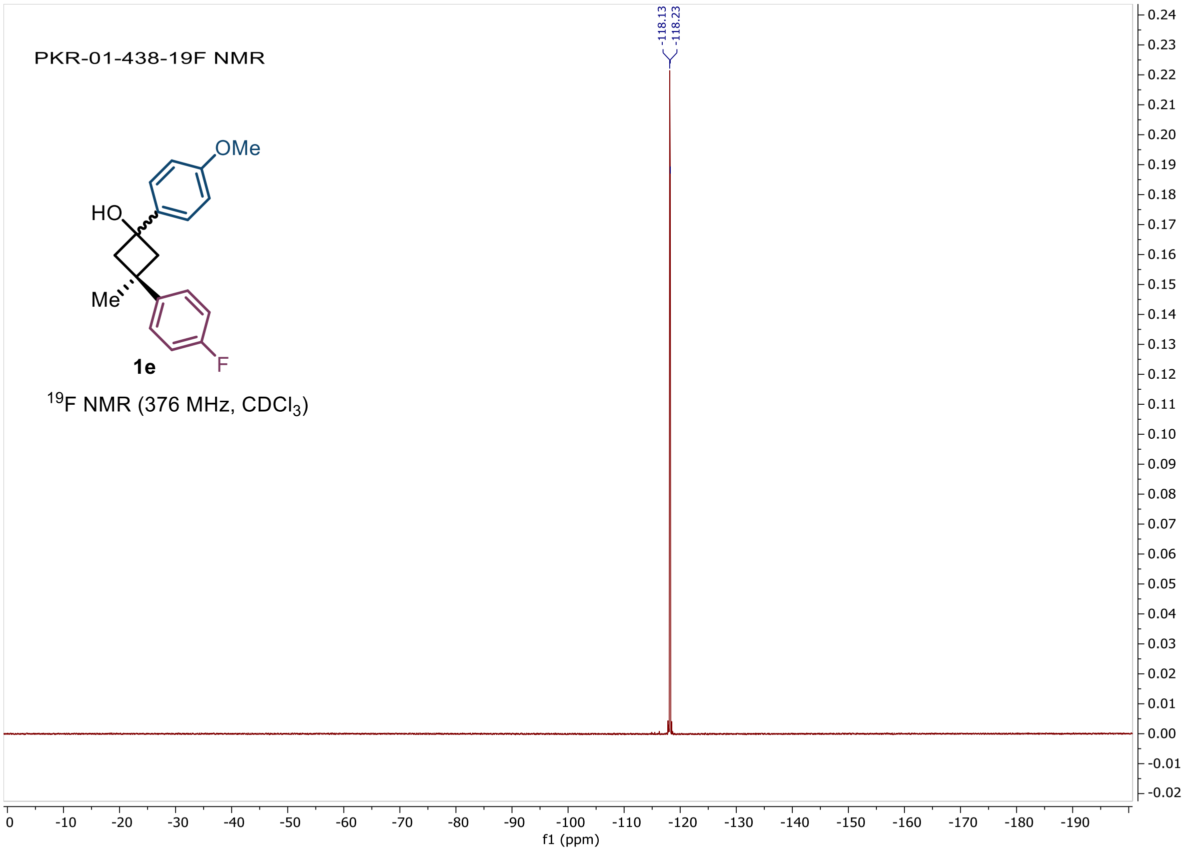
PKR-01-438-19F NMR
OMe
HO
Me
F
1e
^{19}F NMR (376 MHz, CDCl$_3$)
-118.13
-118.23
f1 (ppm)

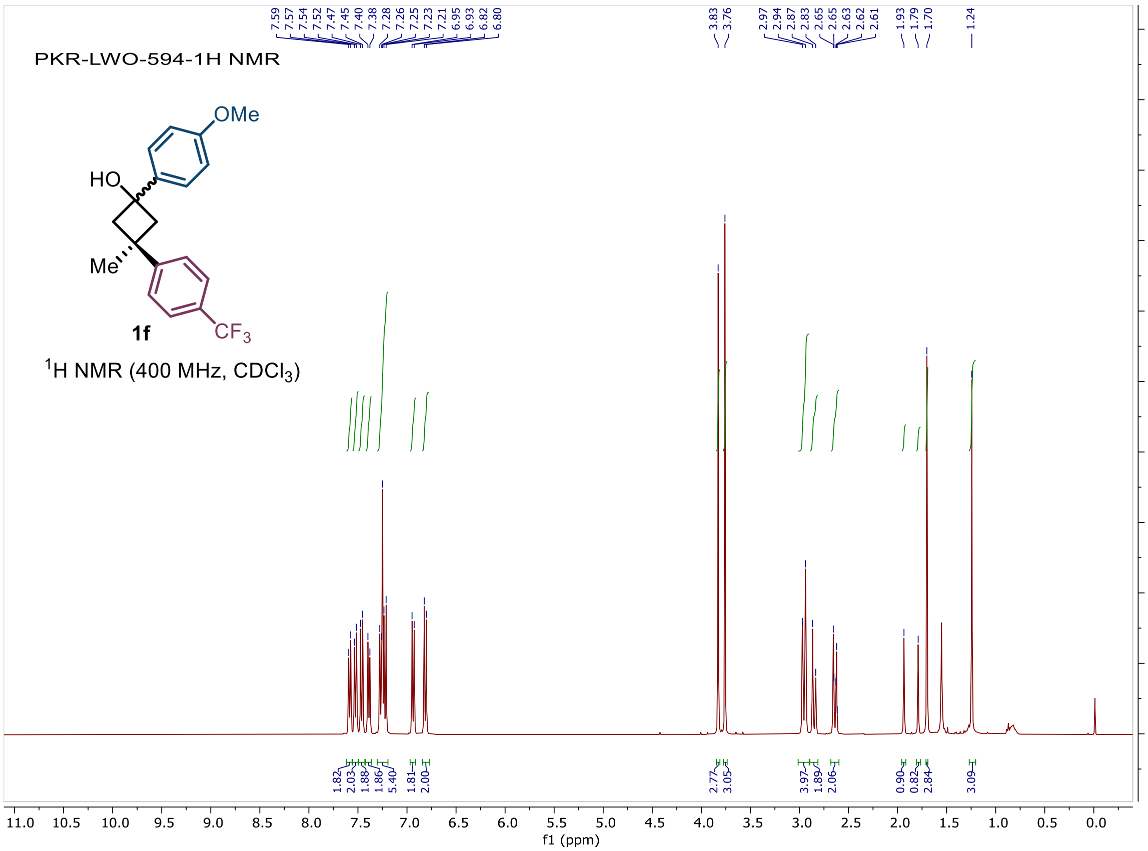
PKR-LWO-594-1H NMR
OMe
HO
Me
CF$_3$
1f
^{1}H NMR (400 MHz, CDCl$_3$)
7.59 7.57 7.54 7.52 7.47 7.45 7.40 7.38 7.28 7.26 7.25 7.23 7.21 6.95 6.93 6.82 6.80
3.83 3.76
2.97 2.94 2.87 2.83 2.65 2.63 2.62 2.61
1.93 1.79 1.70
1.24
1.82 2.03 1.88 1.86 5.40 1.81 2.00
2.77 3.05
3.97 1.89 2.06
0.90 0.82 2.84
3.09
f1 (ppm)

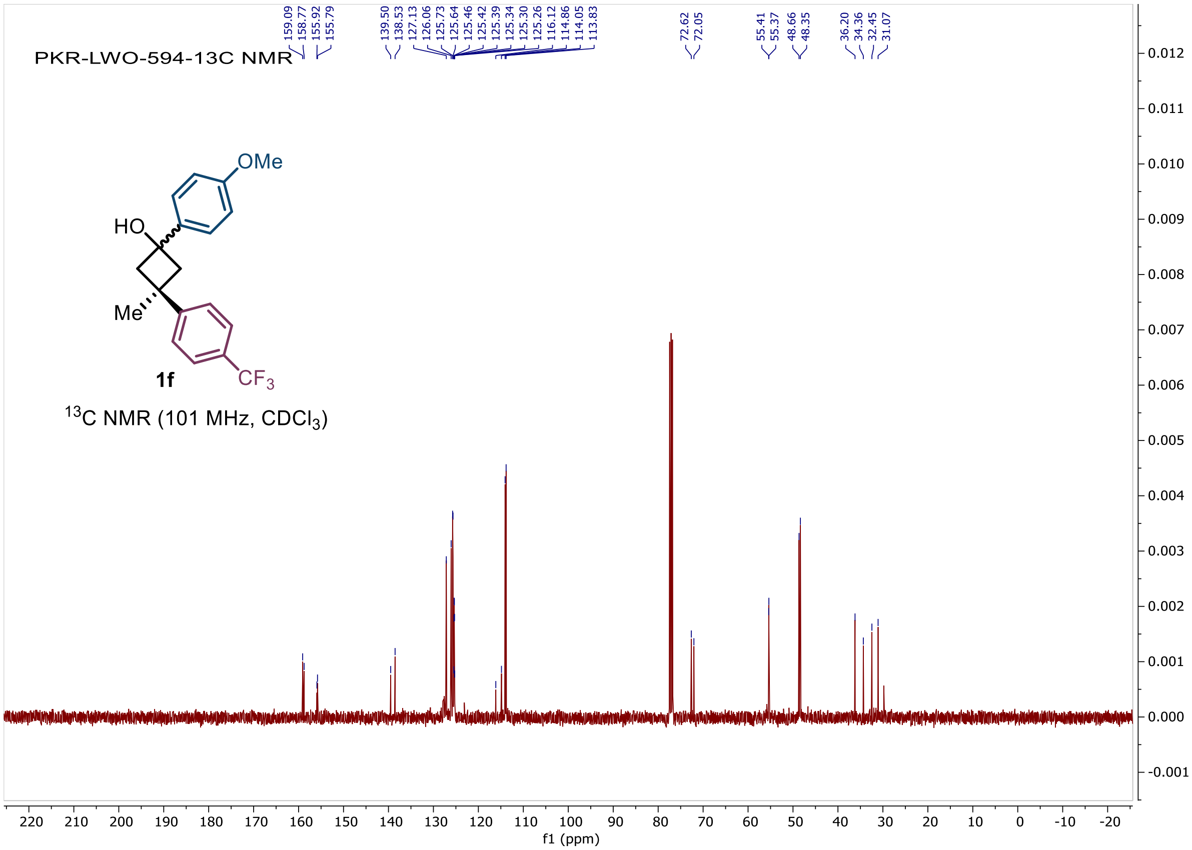
PKR-LWO-594-13C NMR
159.09
158.77
155.92
155.79
139.50
138.53
127.13
126.06
125.73
125.64
125.46
125.42
125.39
125.34
125.30
125.26
116.12
114.86
114.05
113.83
72.62
72.05
55.41
55.37
48.66
48.35
36.20
34.36
32.45
31.07
OMe
HO
Me
CF3
1f
13C NMR (101 MHz, CDCl3)
f1 (ppm)

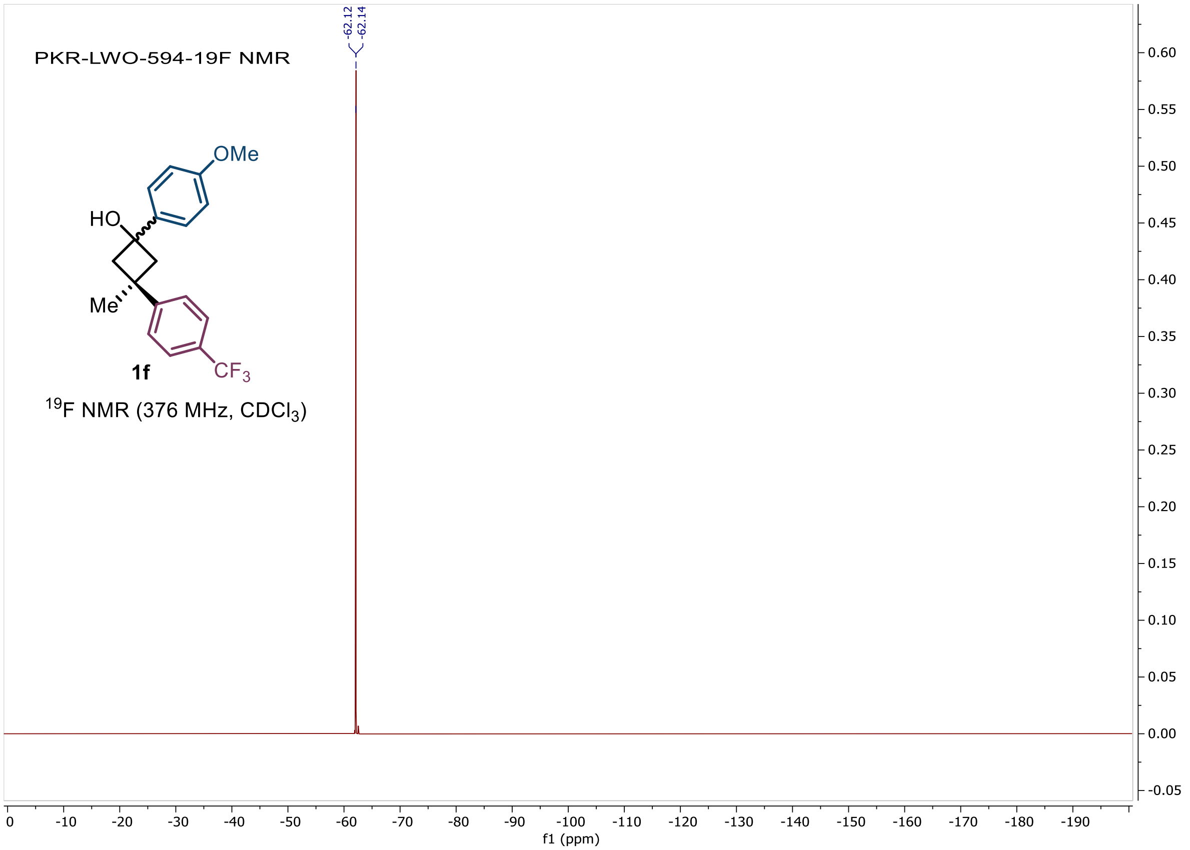
PKR-LWO-594-19F NMR
-62.12
-62.14
OMe
HO
Me
CF3
1f
19F NMR (376 MHz, CDCl3)
f1 (ppm)

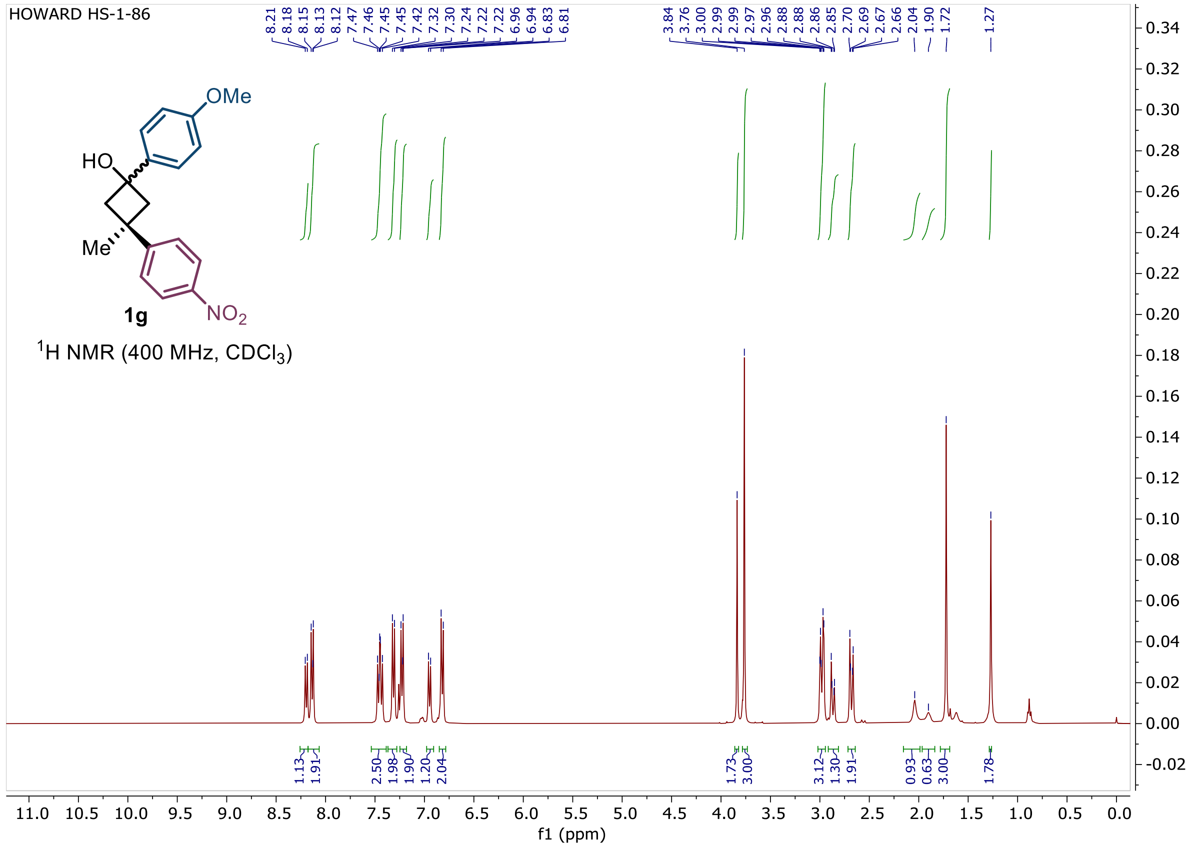
HOWARD HS-1-86
OMe
HO
Me
1g
NO2
1H NMR (400 MHz, CDCl3)
f1 (ppm)

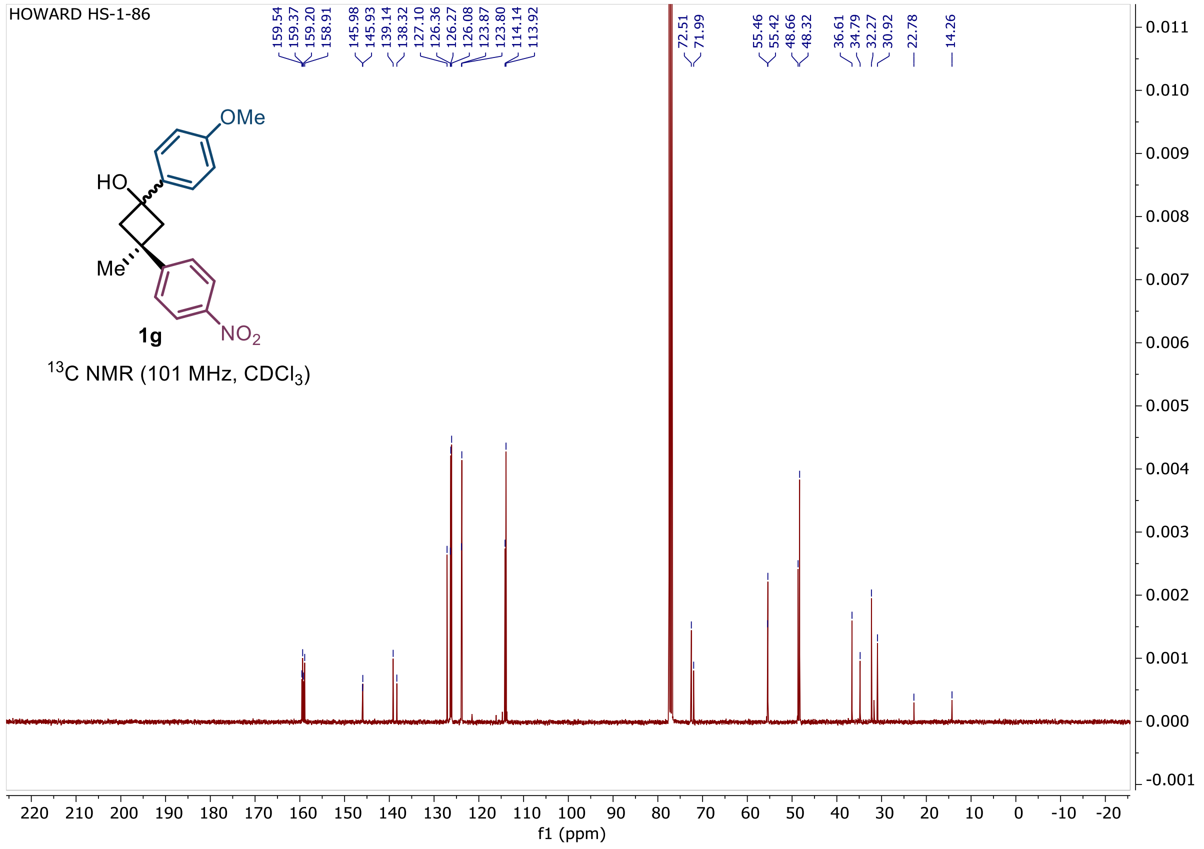
HOWARD HS-1-86
OMe
HO
Me
1g
NO2
13C NMR (101 MHz, CDCl3)
f1 (ppm)

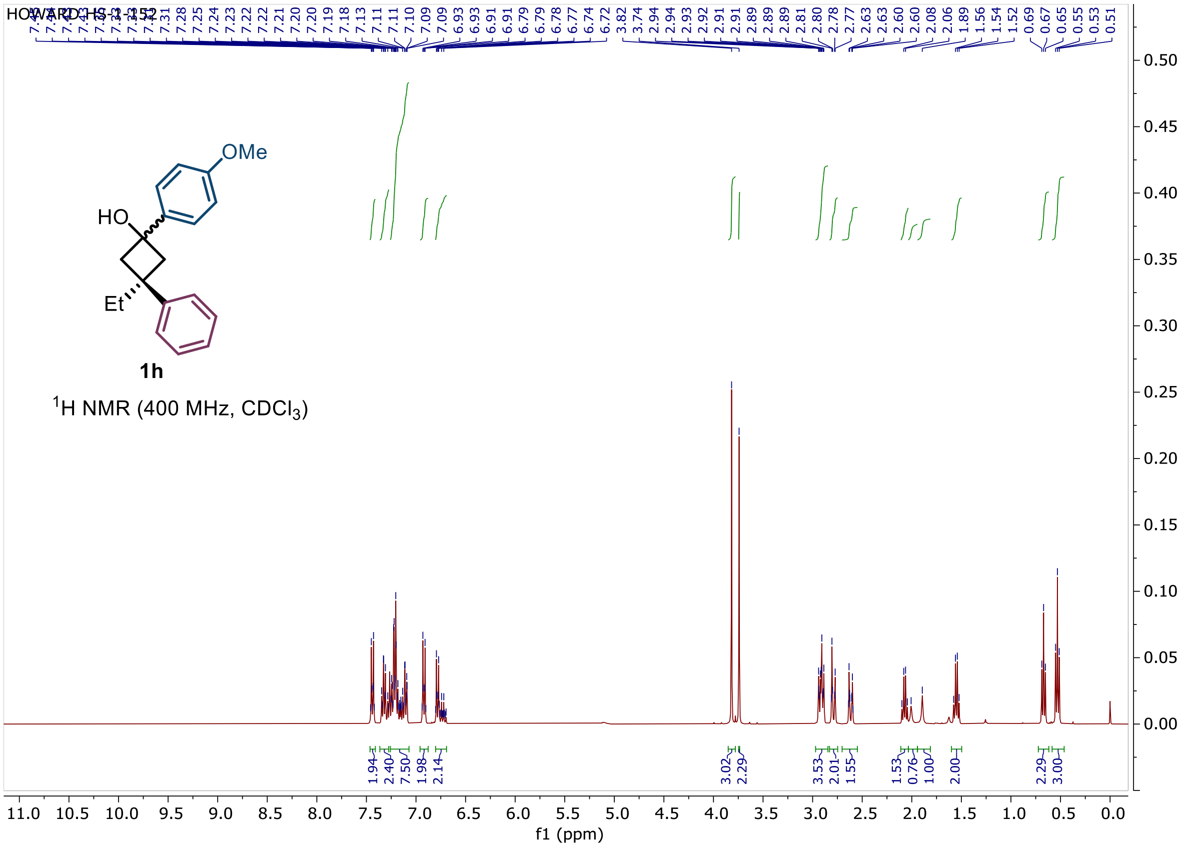
HOWARD HS-1-152
7.31
7.28
7.25
7.24
7.23
7.22
7.22
7.21
7.20
7.20
7.19
7.18
7.13
7.11
7.11
7.10
7.09
7.09
6.93
6.93
6.91
6.91
6.79
6.79
6.78
6.77
6.74
6.72
3.82
3.74
2.94
2.93
2.92
2.91
2.91
2.89
2.89
2.89
2.81
2.80
2.78
2.77
2.63
2.63
2.60
2.60
2.08
2.06
1.89
1.56
1.54
1.52
0.69
0.67
0.65
0.55
0.53
0.51
OMe
HO
Et
1h
1H NMR (400 MHz, CDCl3)
1.94
2.40
7.50
1.98
2.14
3.02
2.29
3.53
2.01
1.55
1.53
0.76
1.00
2.00
2.29
3.00
f1 (ppm)

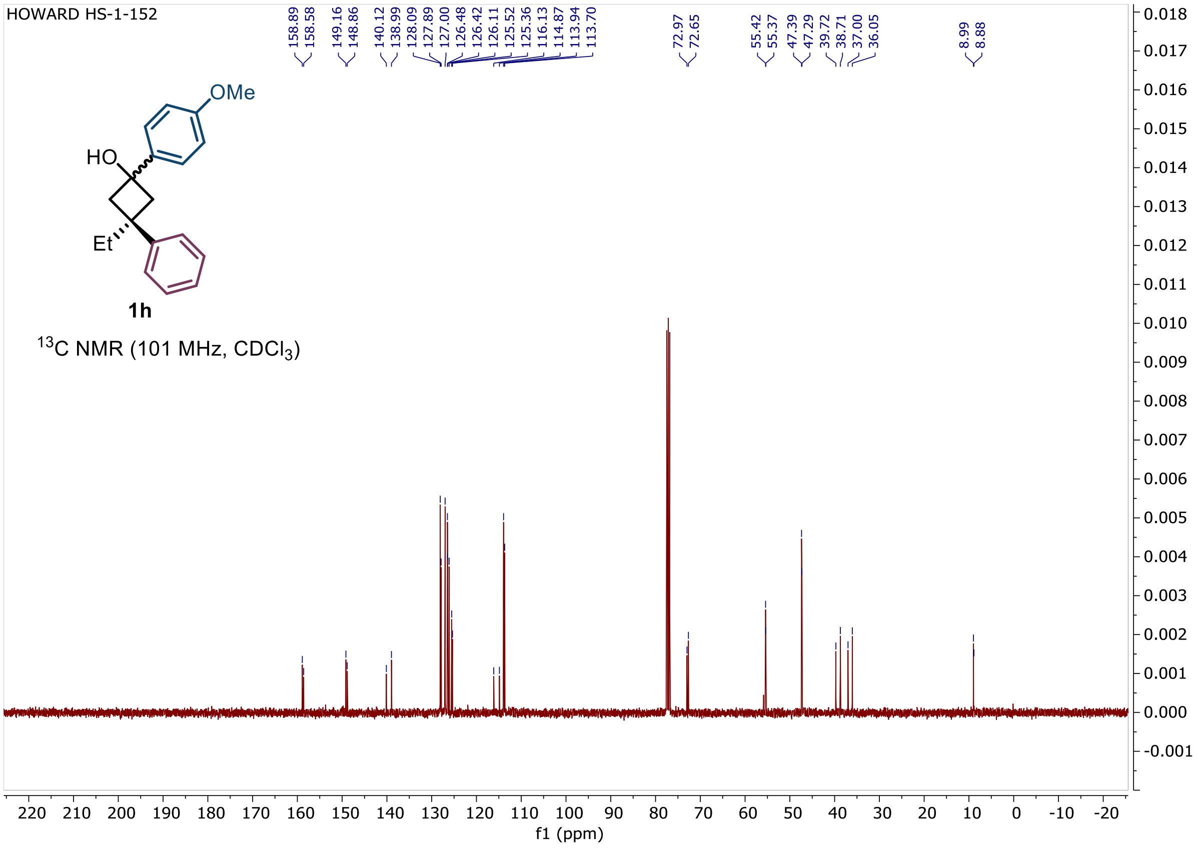
HOWARD HS-1-152
158.89
158.58
149.16
148.86
140.12
138.99
128.09
127.89
127.00
126.48
126.42
126.11
125.52
125.36
116.13
114.87
113.94
113.70
72.97
72.65
55.42
55.37
47.39
47.29
39.72
38.71
37.00
36.05
8.99
8.88
OMe
HO
Et
1h
13C NMR (101 MHz, CDCl3)
f1 (ppm)

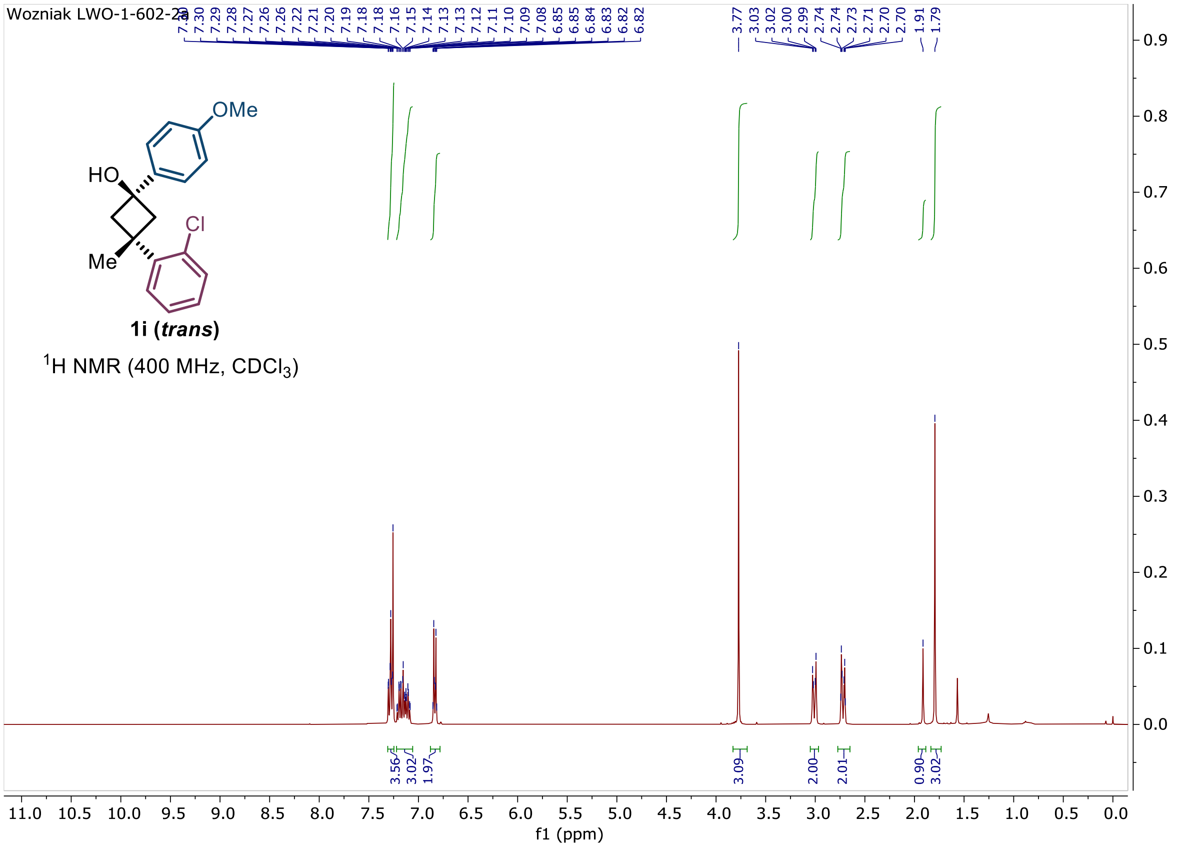

Wozniak LWO-1-602-2a
OMe
HO
Cl
Me
1i (trans)
1H NMR (400 MHz, CDCl3)
f1 (ppm)

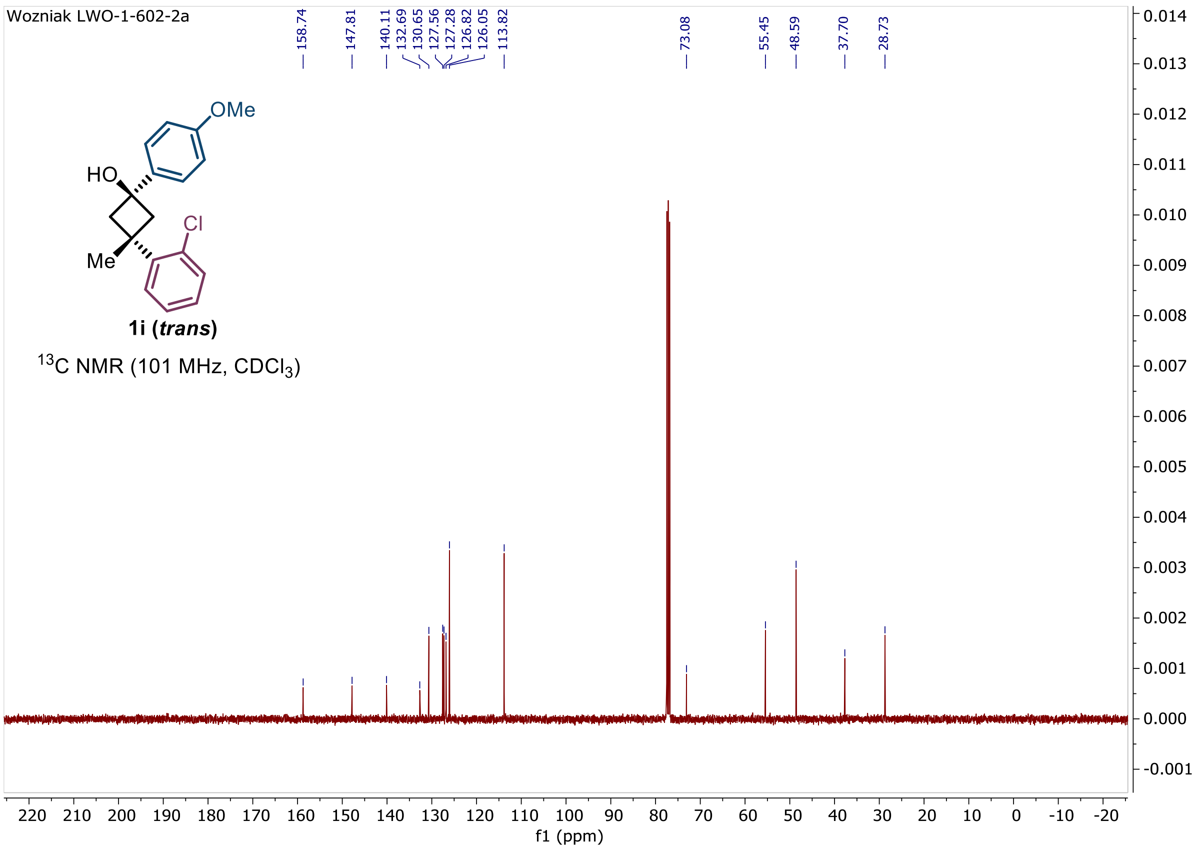

Wozniak LWO-1-602-2a
158.74
147.81
140.11
132.69
130.65
127.56
127.28
126.82
126.05
113.82
73.08
55.45
48.59
37.70
28.73
OMe
HO
Cl
Me
1i (trans)
13C NMR (101 MHz, CDCl3)
f1 (ppm)

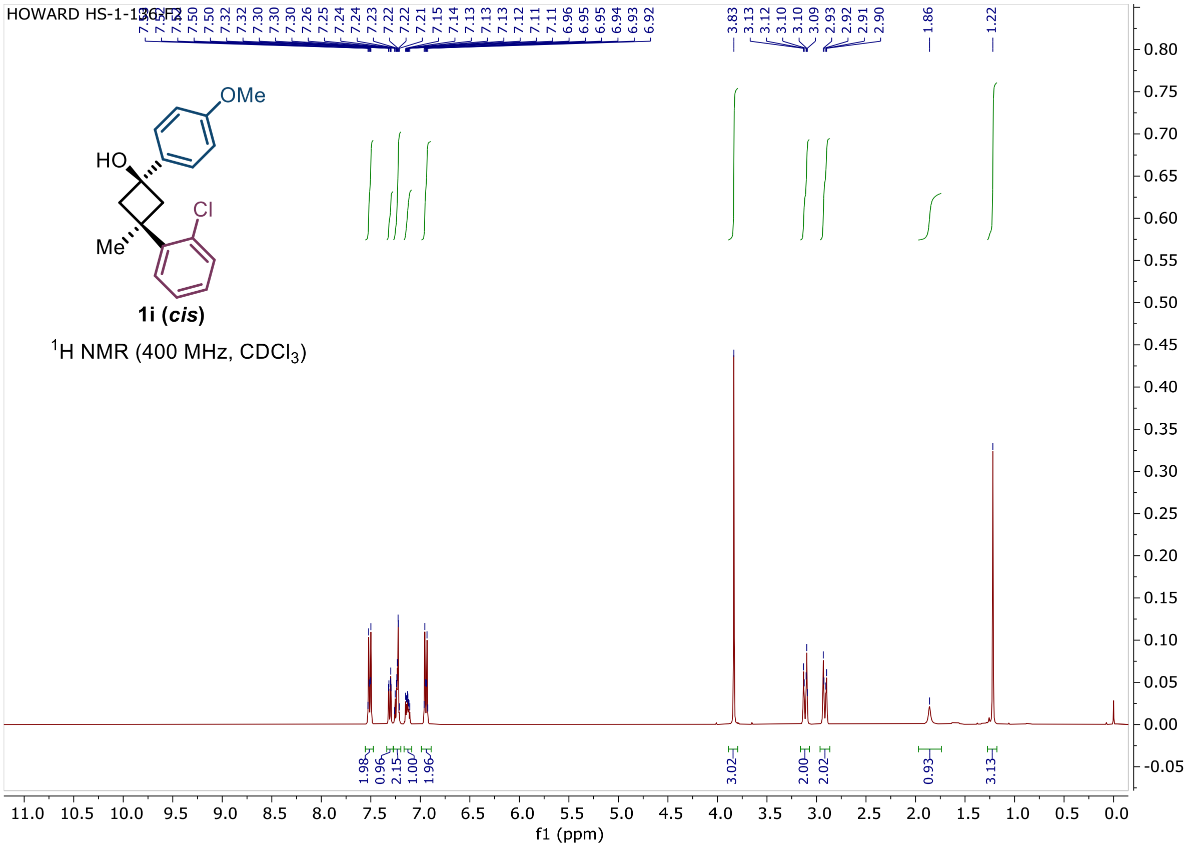

HOWARD HS-1-136-F2
7.50
7.50
7.32
7.32
7.30
7.30
7.30
7.26
7.25
7.24
7.24
7.23
7.22
7.22
7.21
7.15
7.14
7.13
7.13
7.13
7.12
7.11
7.11
6.96
6.95
6.95
6.94
6.93
6.92
3.83
3.13
3.12
3.10
3.10
3.09
2.93
2.92
2.91
2.90
1.86
1.22
OMe
HO
Cl
Me
1i (cis)
^{1}H NMR (400 MHz, $CDCl_3$)
1.98
0.96
2.15
1.00
1.96
3.02
2.00
2.02
0.93
3.13
f1 (ppm)

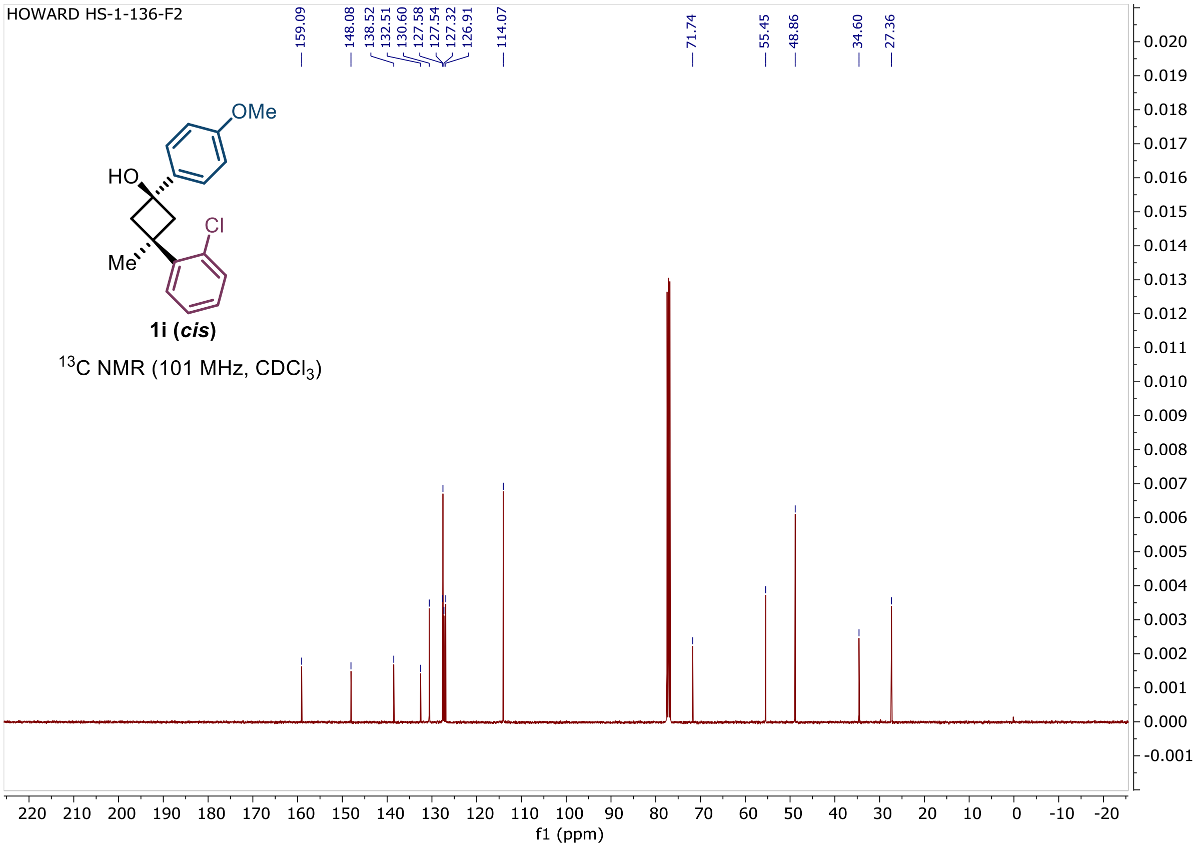

HOWARD HS-1-136-F2
159.09
148.08
138.52
132.51
130.60
127.58
127.54
127.32
126.91
114.07
71.74
55.45
48.86
34.60
27.36
OMe
HO
Cl
Me
1i (cis)
^{13}C NMR (101 MHz, $CDCl_3$)
f1 (ppm)

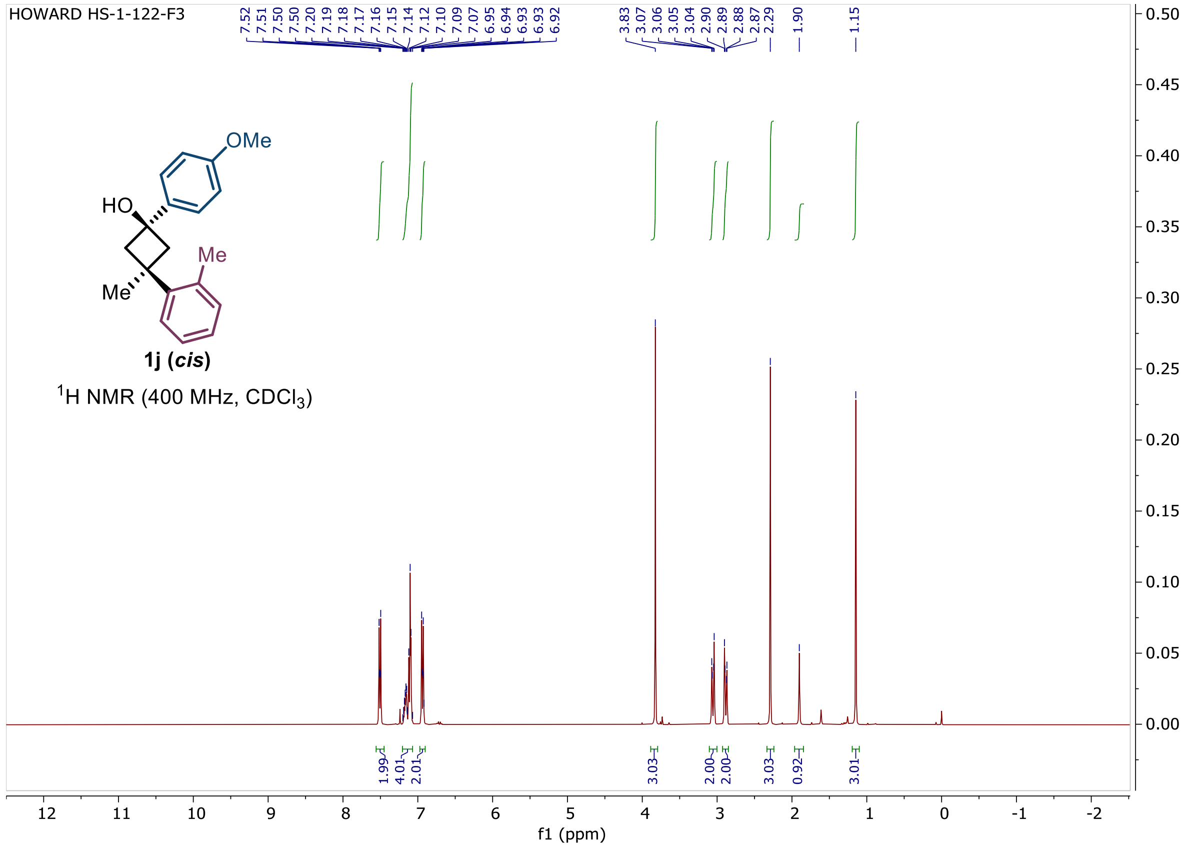
HOWARD HS-1-122-F3
OMe
HO
Me
Me
1j (cis)
^{1}H NMR (400 MHz, CDCl$_3$)
f1 (ppm)

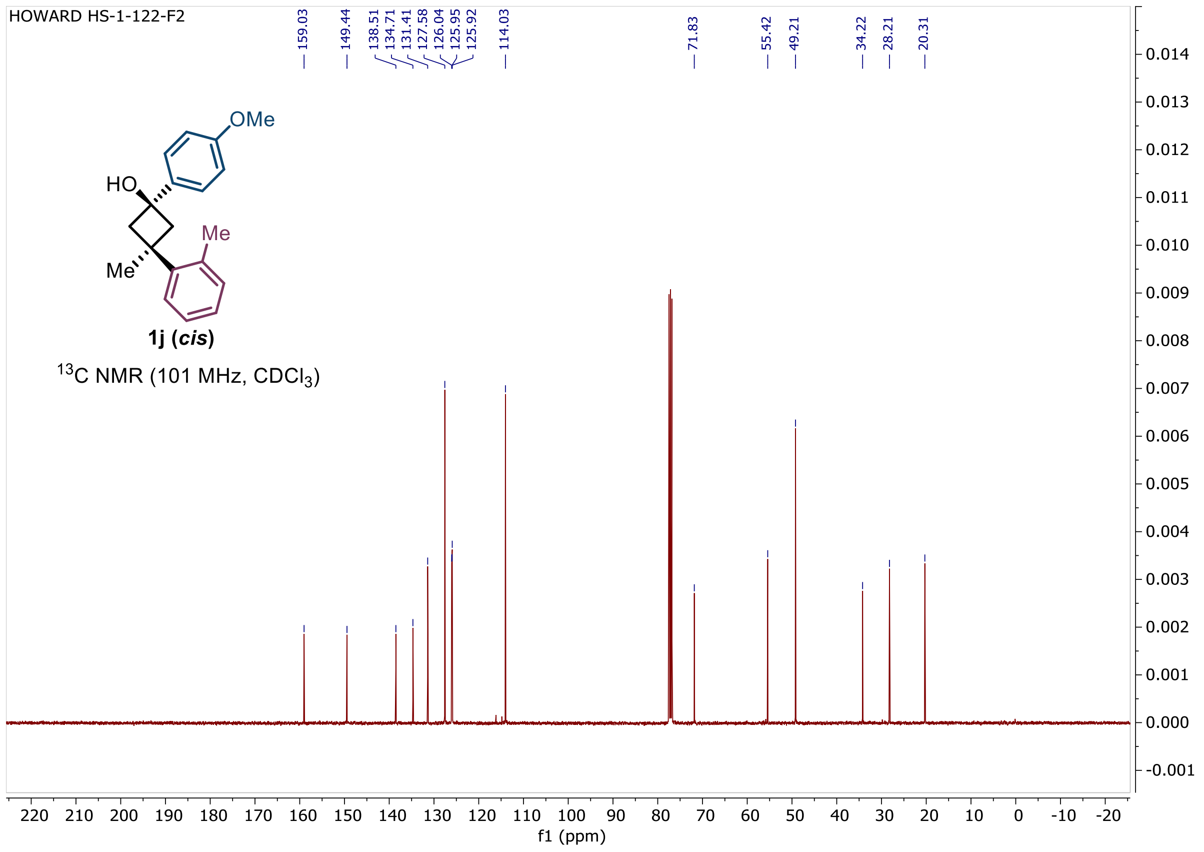
HOWARD HS-1-122-F2
OMe
HO
Me
Me
1j (cis)
^{13}C NMR (101 MHz, CDCl$_3$)
f1 (ppm)

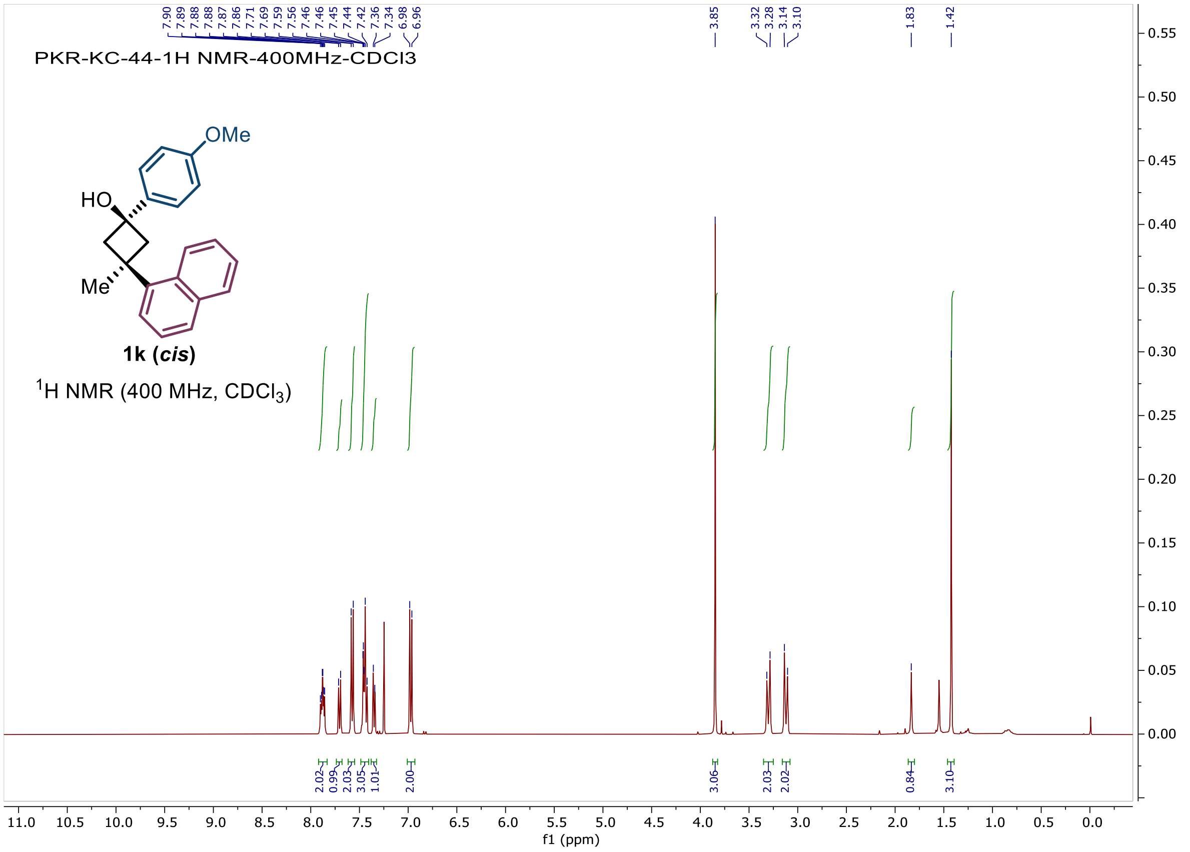
PKR-KC-44-1H NMR-400MHz-CDCl3
OMe
HO
Me
1k (cis)
^{1}H NMR (400 MHz, CDCl$_3$)
f1 (ppm)

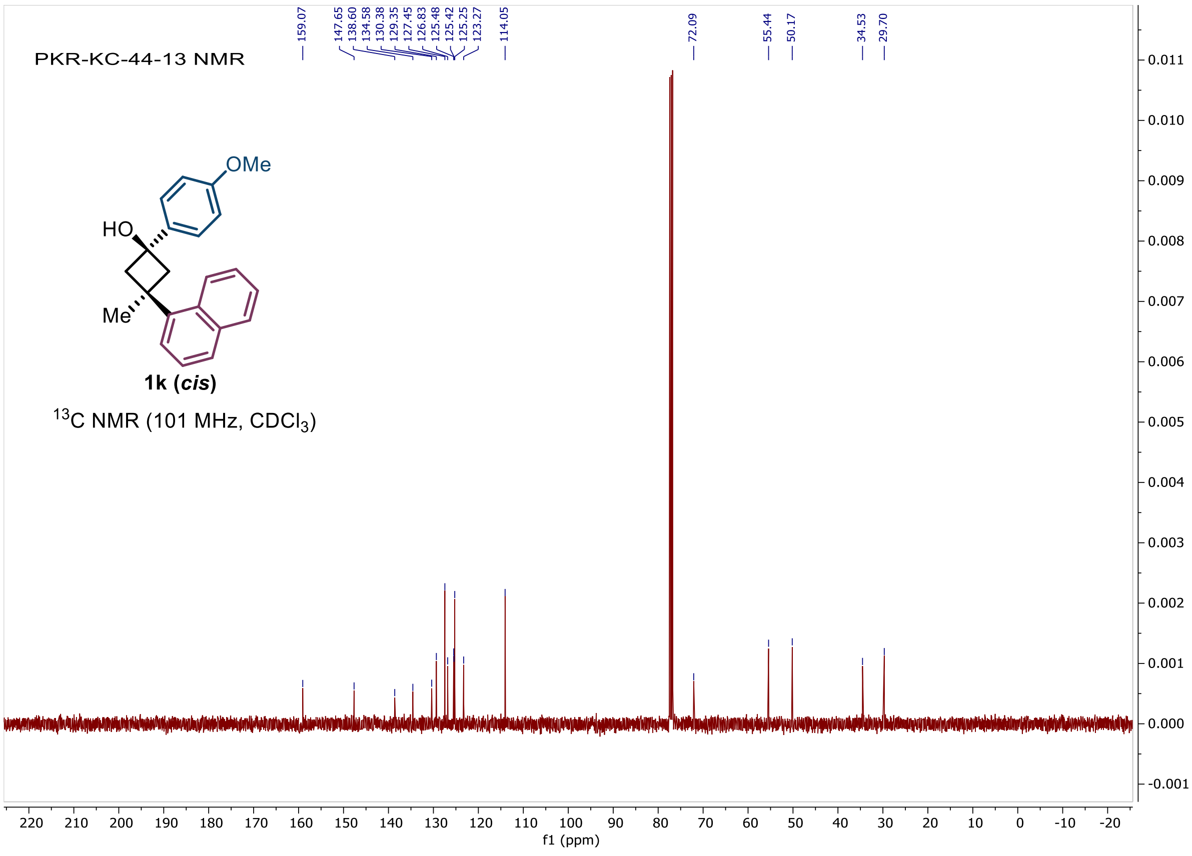
PKR-KC-44-13 NMR
OMe
HO
Me
1k (cis)
^{13}C NMR (101 MHz, CDCl$_3$)
f1 (ppm)

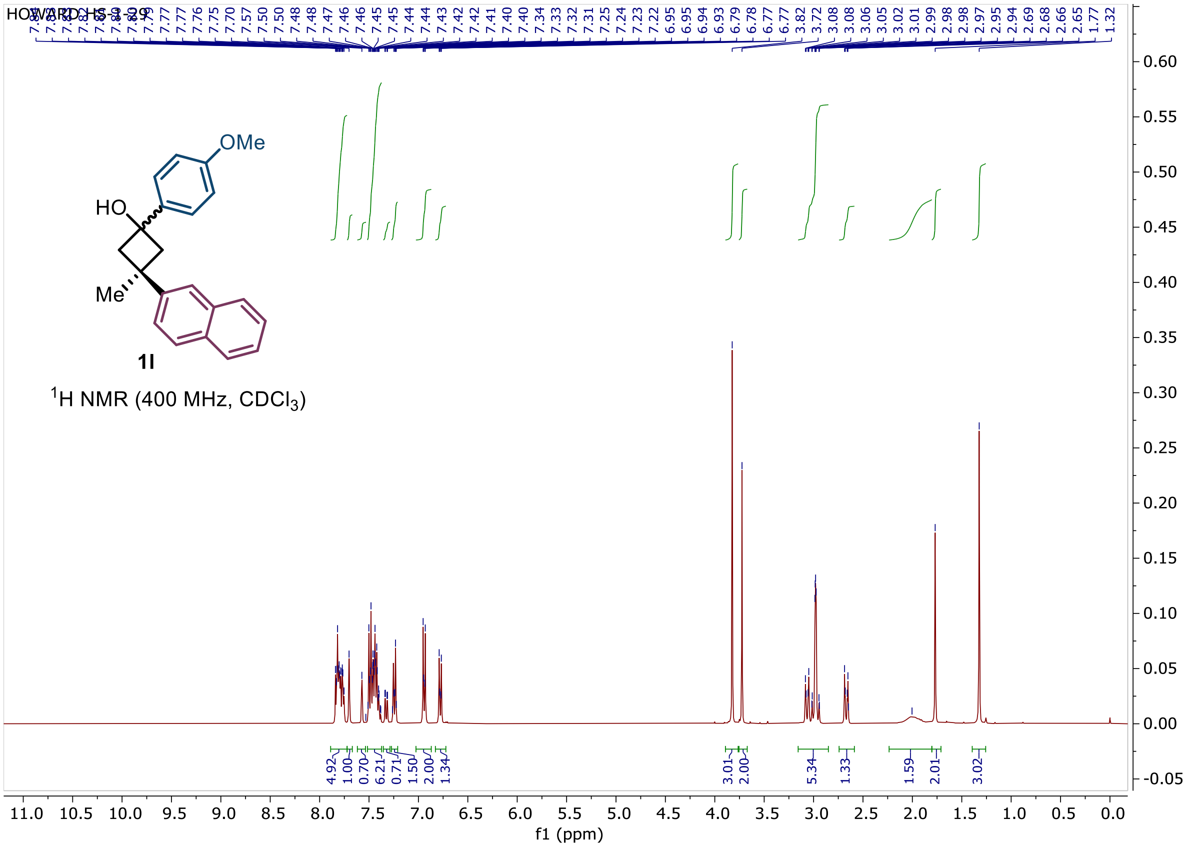
HOWARD
OMe
HO
Me
1l
1H NMR (400 MHz, CDCl3)
f1 (ppm)

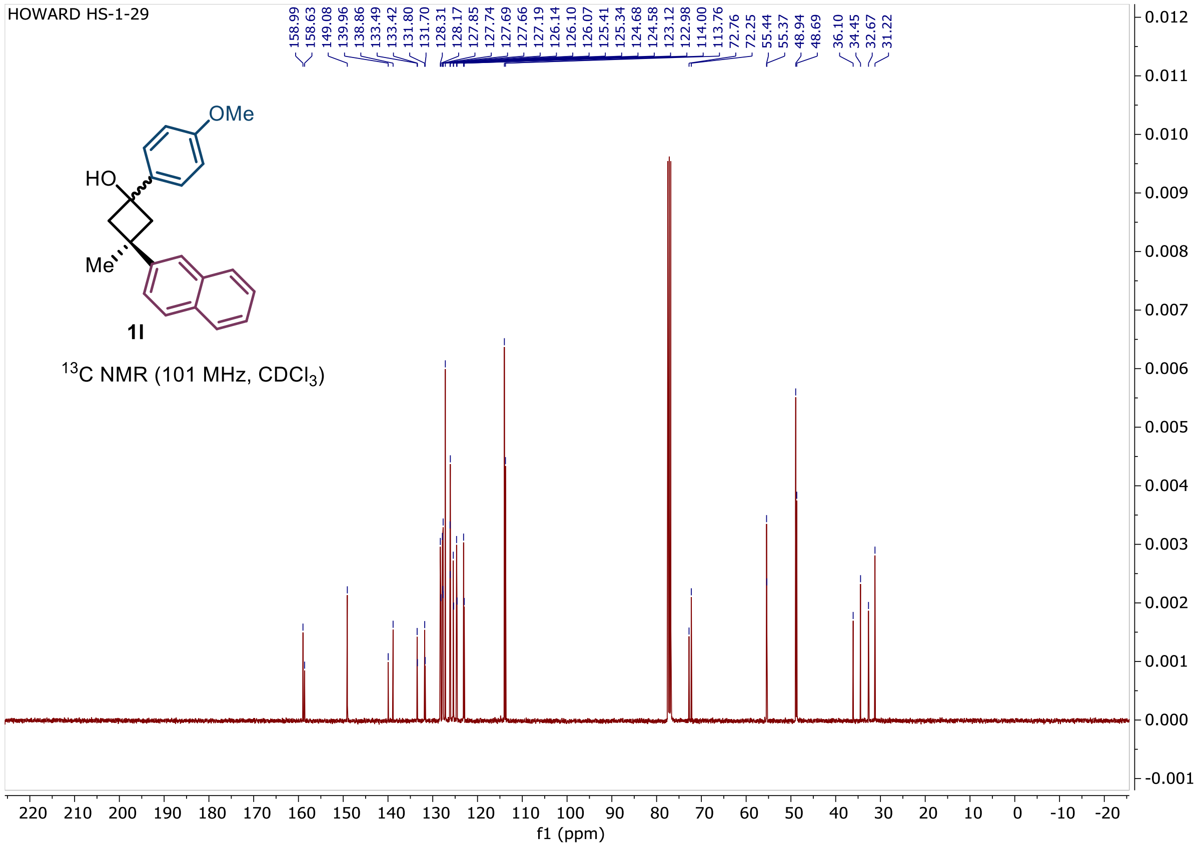
HOWARD HS-1-29
OMe
HO
Me
1l
13C NMR (101 MHz, CDCl3)
f1 (ppm)

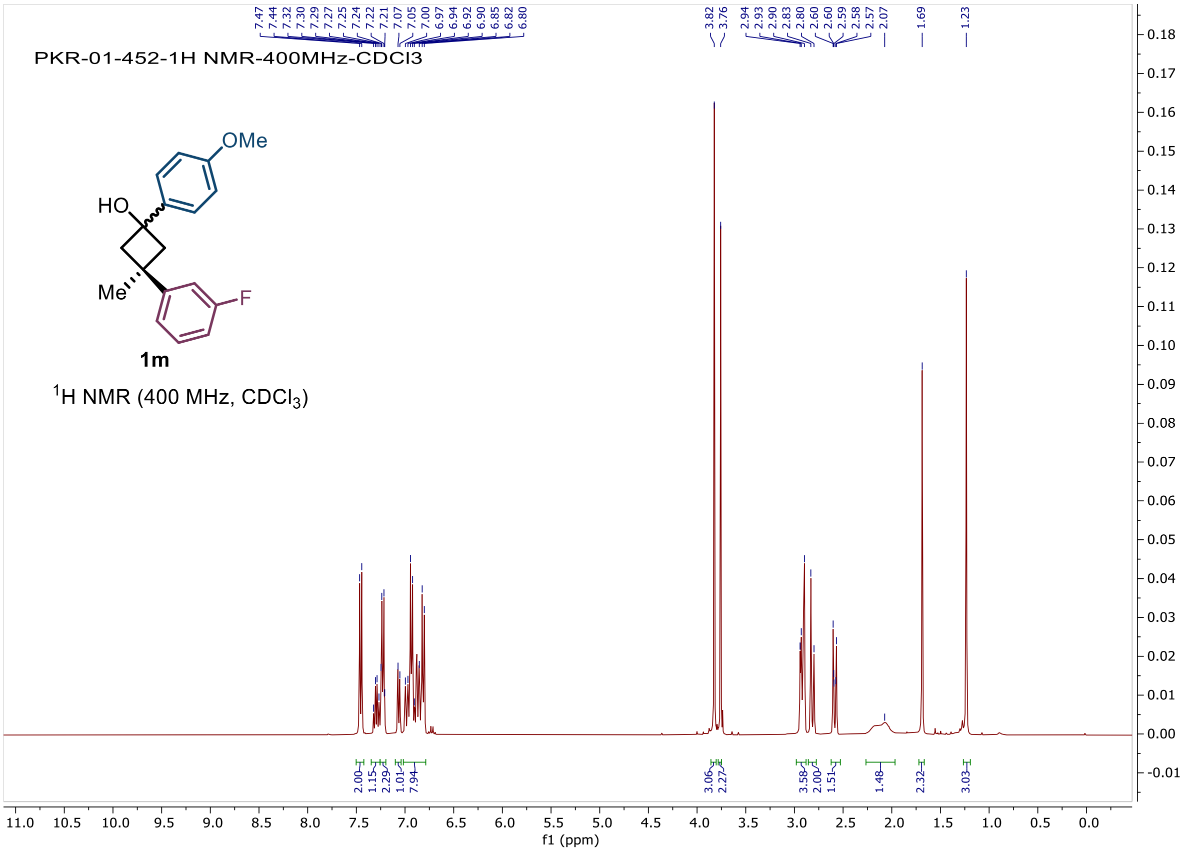
PKR-01-452-1H NMR-400MHz-CDCl3
7.47 7.44 7.32 7.30 7.29 7.27 7.25 7.24 7.22 7.21 7.07 7.05 7.00 6.97 6.94 6.92 6.90 6.85 6.82 6.80
3.82 3.76
2.94 2.93 2.90 2.83 2.80 2.60 2.59 2.58 2.57 2.07
1.69
1.23
OMe
HO
Me
F
1m
^{1}H NMR (400 MHz, CDCl$_3$)
2.00 1.15 2.29 1.01 7.94
3.06 2.27
3.58 2.00 1.51
1.48
2.32
3.03
f1 (ppm)

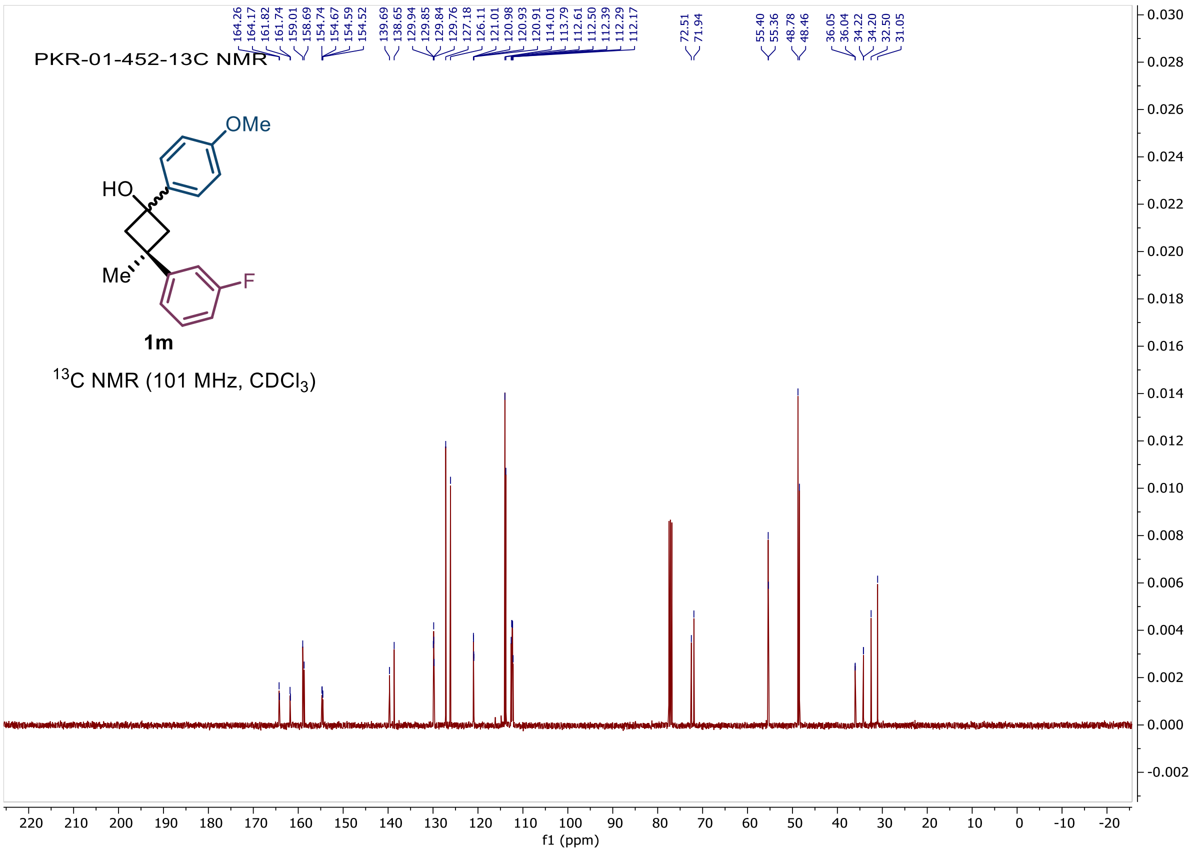
PKR-01-452-13C NMR
164.26 164.17 161.82 161.74 159.01 158.69 154.74 154.67 154.59 154.52 139.69 138.65 129.94 129.85 129.84 129.76 127.18 126.11 121.01 120.98 120.93 120.91 114.01 113.79 112.61 112.50 112.39 112.29 112.17
72.51 71.94
55.40 55.36 48.78 48.46
36.05 36.04 34.22 34.20 32.50 31.05
OMe
HO
Me
F
1m
^{13}C NMR (101 MHz, CDCl$_3$)
f1 (ppm)

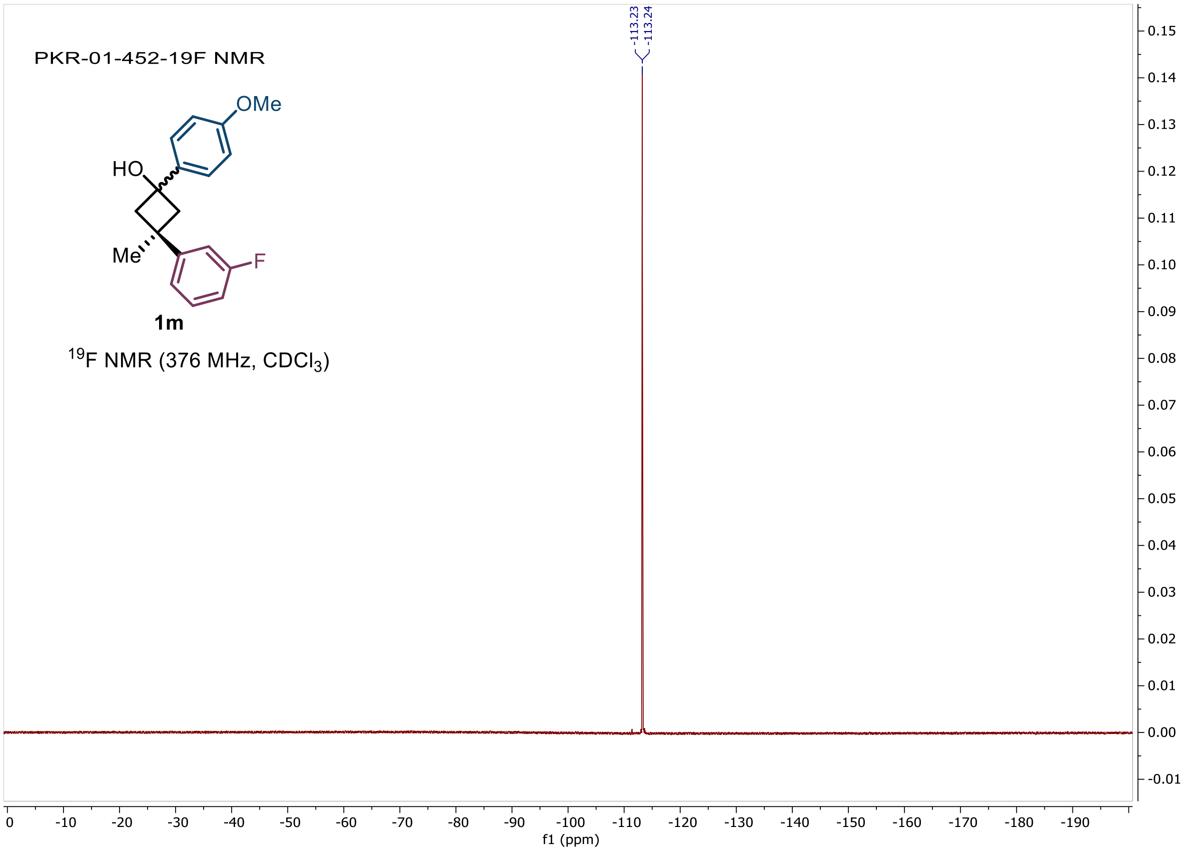
PKR-01-452-19F NMR
OMe
HO
Me
F
1m
^{19}F NMR (376 MHz, CDCl$_3$)
-113.23
-113.24
f1 (ppm)

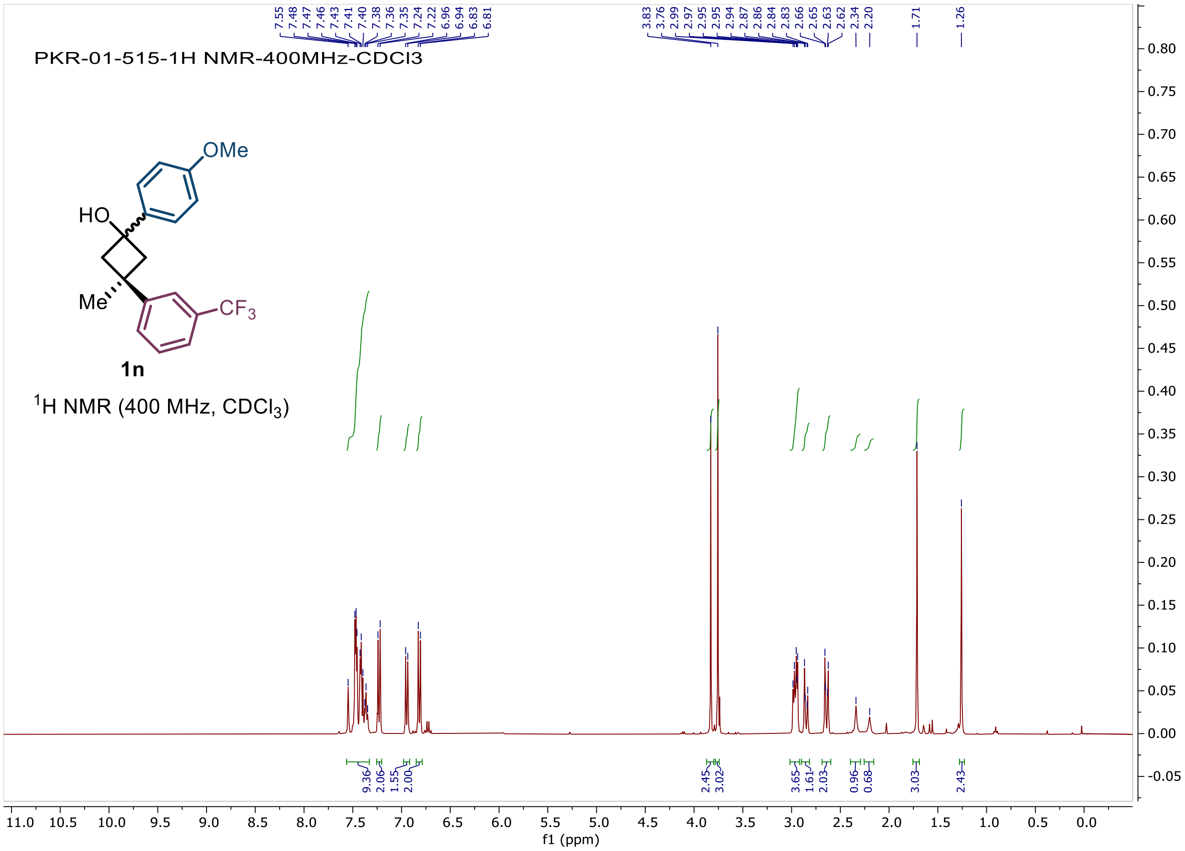
PKR-01-515-1H NMR-400MHz-CDCl3
OMe
HO
Me
CF$_3$
1n
^{1}H NMR (400 MHz, CDCl$_3$)
7.55 7.48 7.47 7.46 7.43 7.41 7.40 7.38 7.36 7.35 7.24 7.22 6.96 6.94 6.83 6.81
3.83 3.76 2.99 2.97 2.95 2.95 2.94 2.87 2.86 2.84 2.83 2.66 2.65 2.63 2.62 2.34 2.20
1.71
1.26
9.36 2.06 1.55 2.00 2.45 3.02 3.65 1.61 2.03 0.96 0.68 3.03 2.43
f1 (ppm)

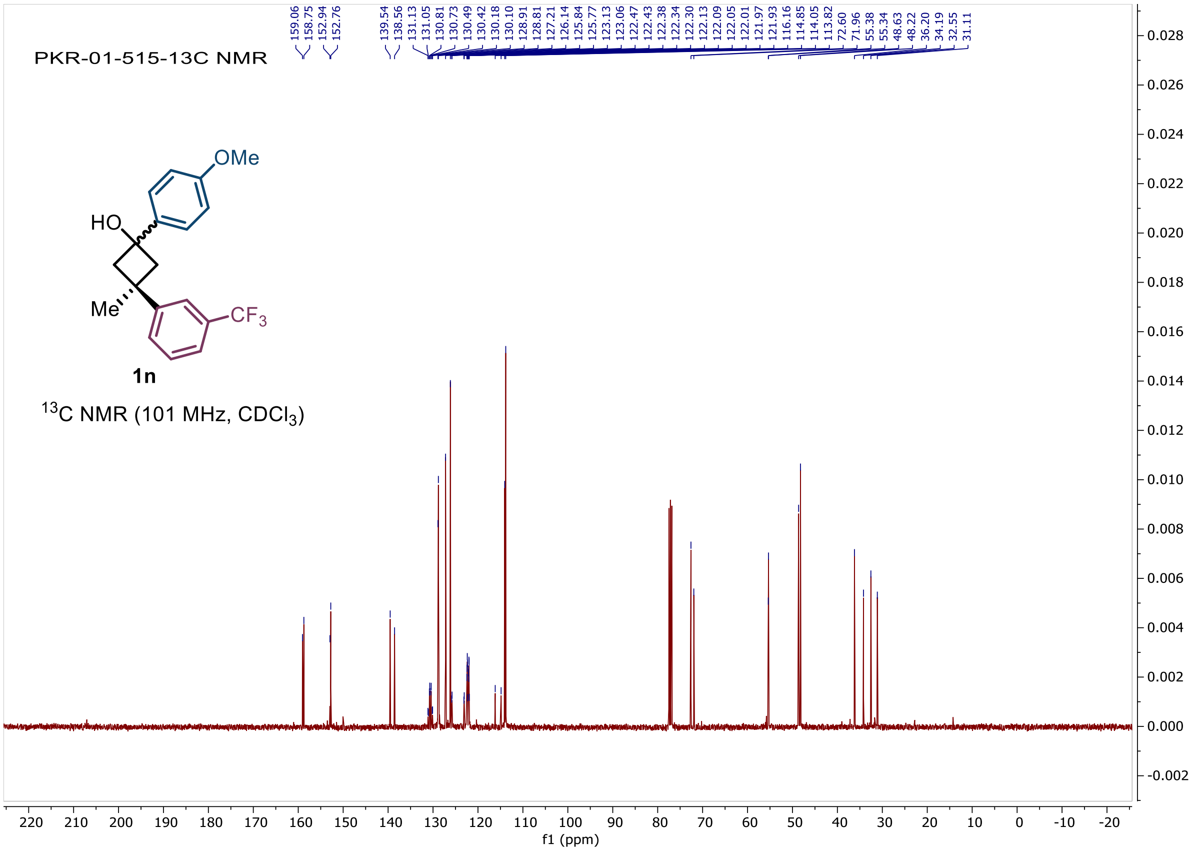
PKR-01-515-13C NMR
OMe
HO
Me
CF3
1n
13C NMR (101 MHz, CDCl3)
f1 (ppm)

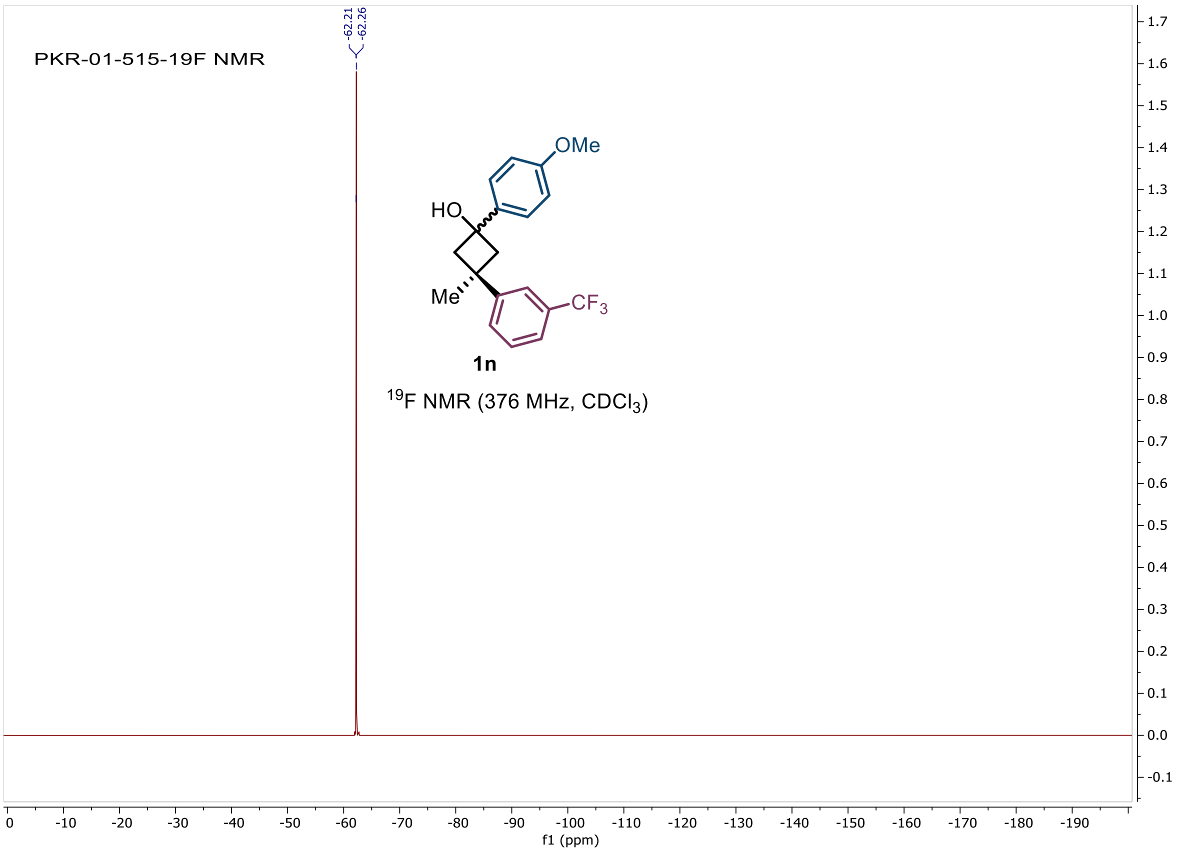
PKR-01-515-19F NMR
-62.21
-62.26
OMe
HO
Me
CF3
1n
19F NMR (376 MHz, CDCl3)
f1 (ppm)

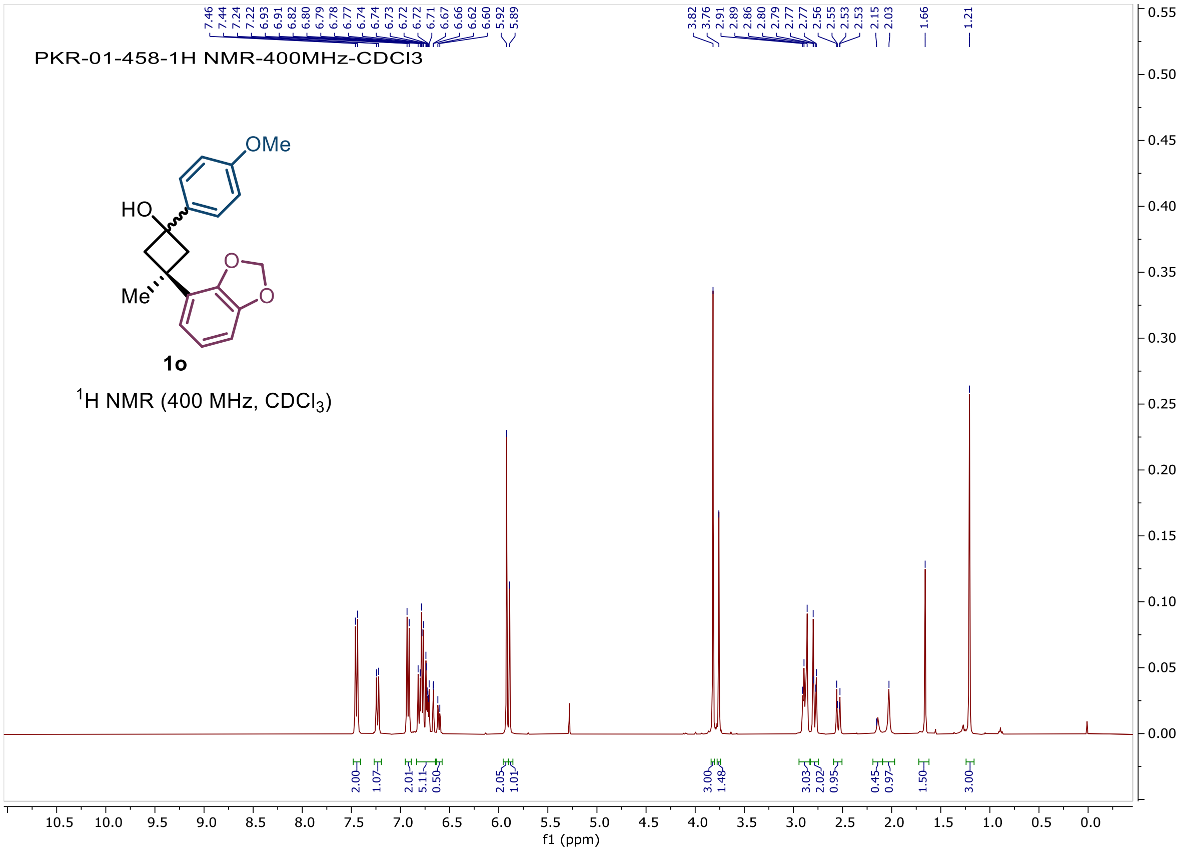
PKR-01-458-1H NMR-400MHz-CDCl3
7.46 7.44 7.24 7.22 6.93 6.91 6.82 6.80 6.79 6.78 6.77 6.74 6.74 6.73 6.72 6.72 6.71 6.67 6.66 6.62 6.60 5.92 5.89
3.82 3.76 2.91 2.89 2.86 2.80 2.79 2.77 2.56 2.55 2.53 2.53 2.15 2.03 1.66 1.21
OMe
HO
Me
O
O
1o
^{1}H NMR (400 MHz, CDCl$_3$)
2.00 1.07 2.01 5.11 0.50 2.05 1.01 3.00 1.48 3.03 2.02 0.95 0.45 0.97 1.50 3.00
f1 (ppm)

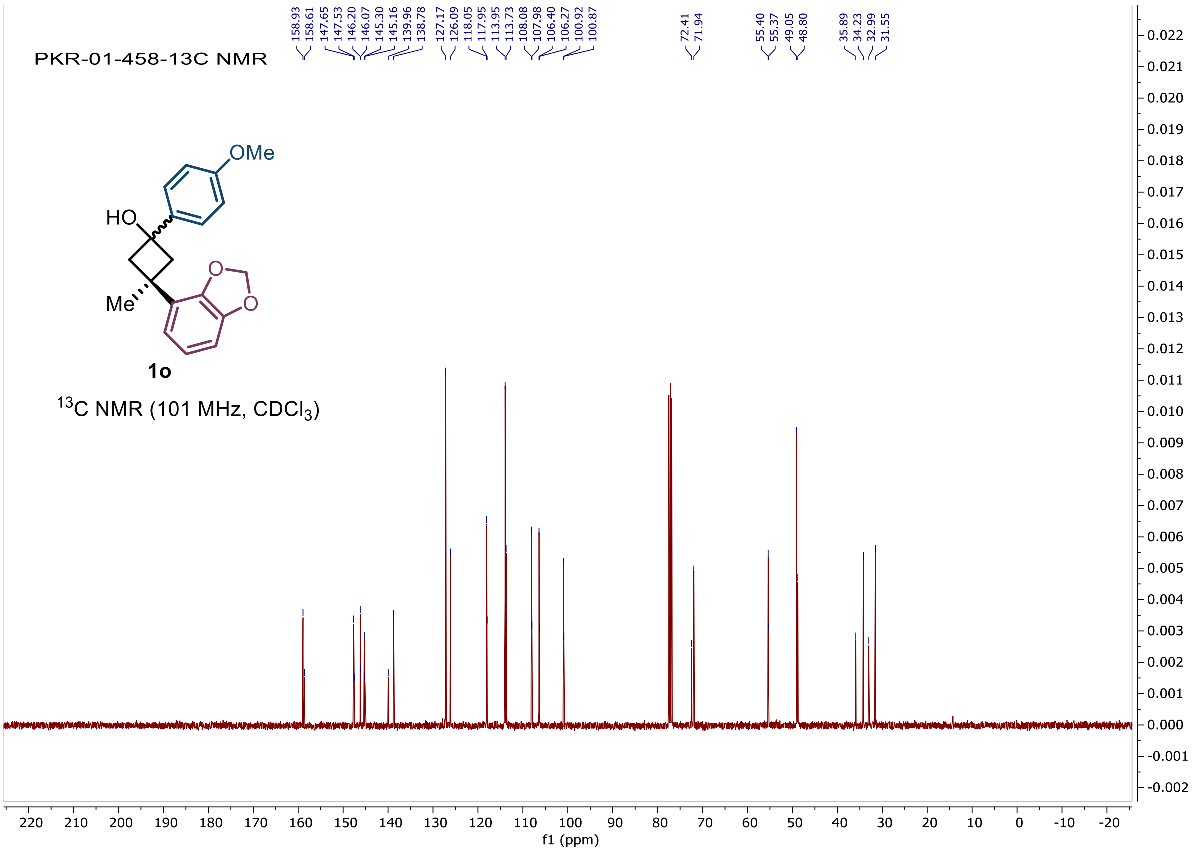
PKR-01-458-13C NMR
158.93 158.61 147.65 147.53 146.20 146.07 145.30 145.16 139.96 138.78 127.17 126.09 118.05 117.95 113.95 113.73 108.08 107.98 106.40 106.27 100.92 100.87
72.41 71.94 55.40 55.37 49.05 48.80 35.89 34.23 32.99 31.55
OMe
HO
Me
O
O
1o
^{13}C NMR (101 MHz, CDCl$_3$)
f1 (ppm)

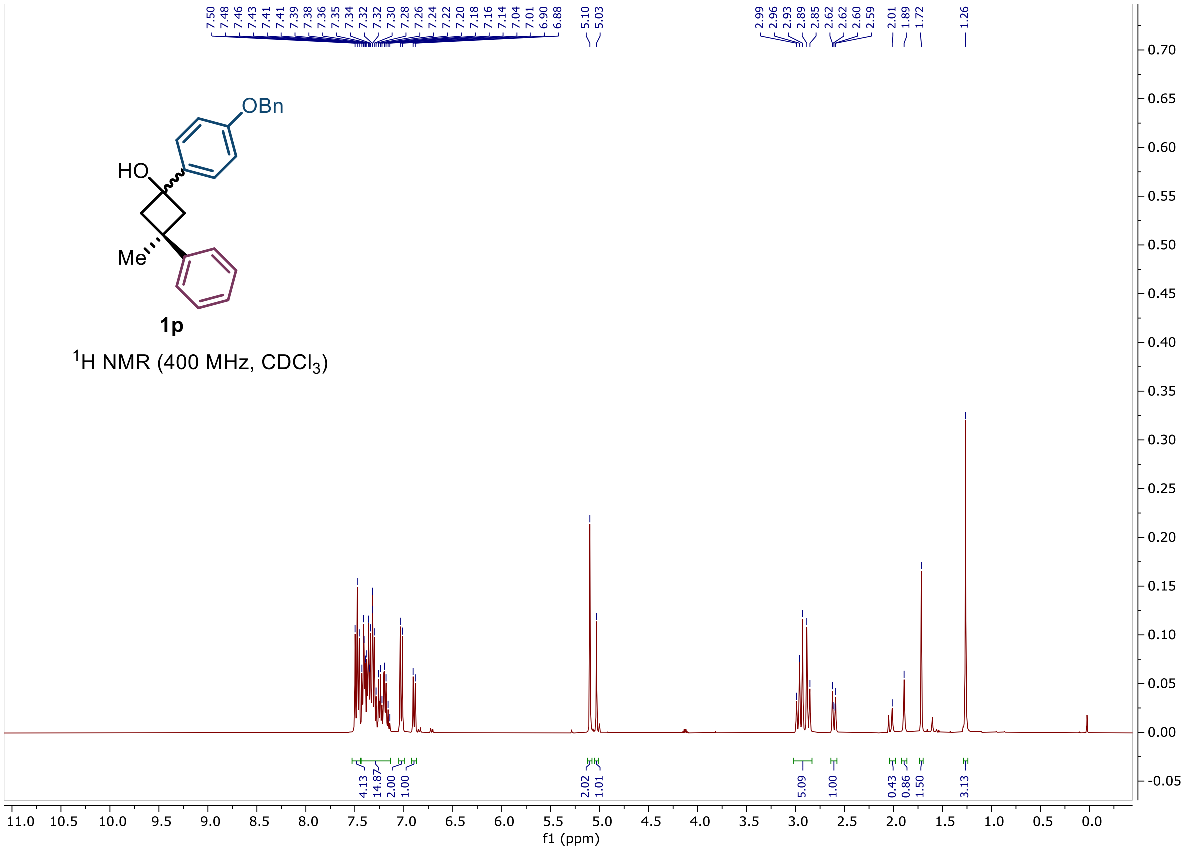

OBn
HO
Me
1p
^{1}H NMR (400 MHz, CDCl$_3$)
f1 (ppm)

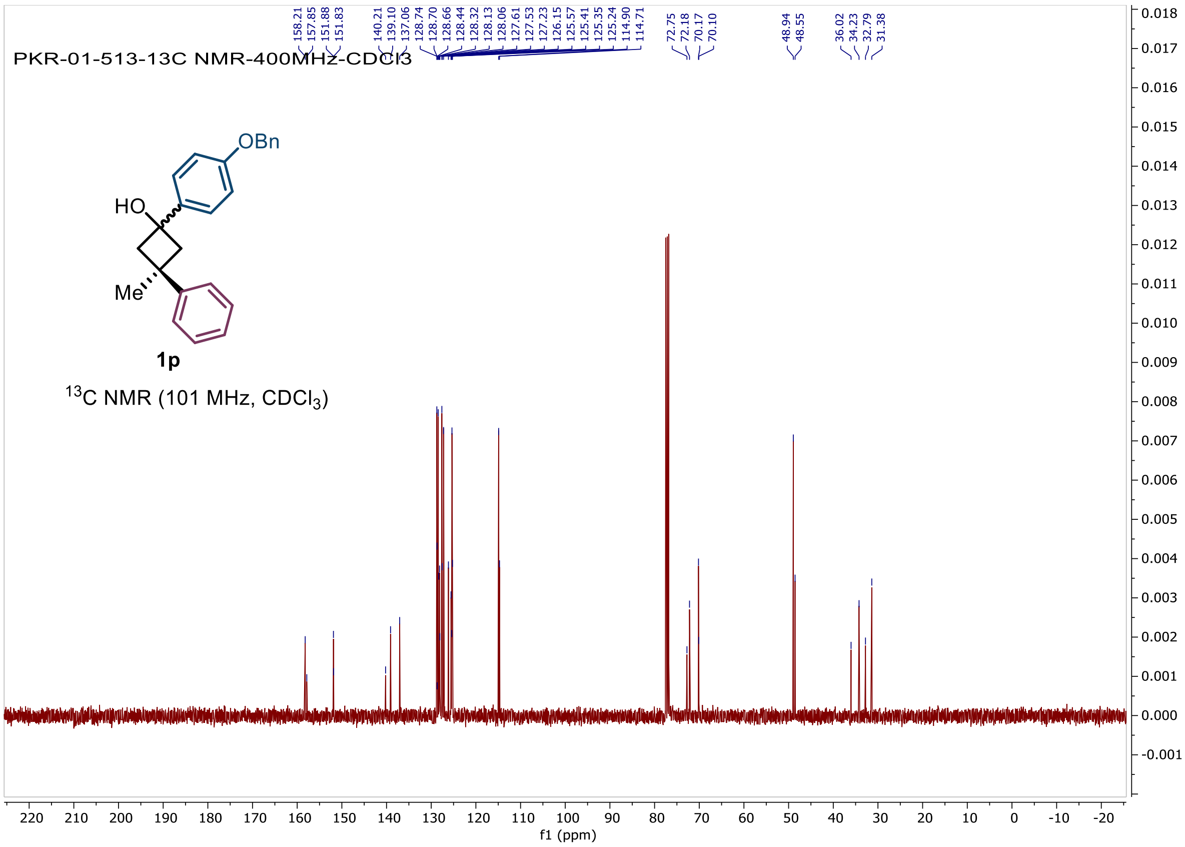

PKR-01-513-13C NMR-400MHz-CDCl3
OBn
HO
Me
1p
^{13}C NMR (101 MHz, CDCl$_3$)
f1 (ppm)

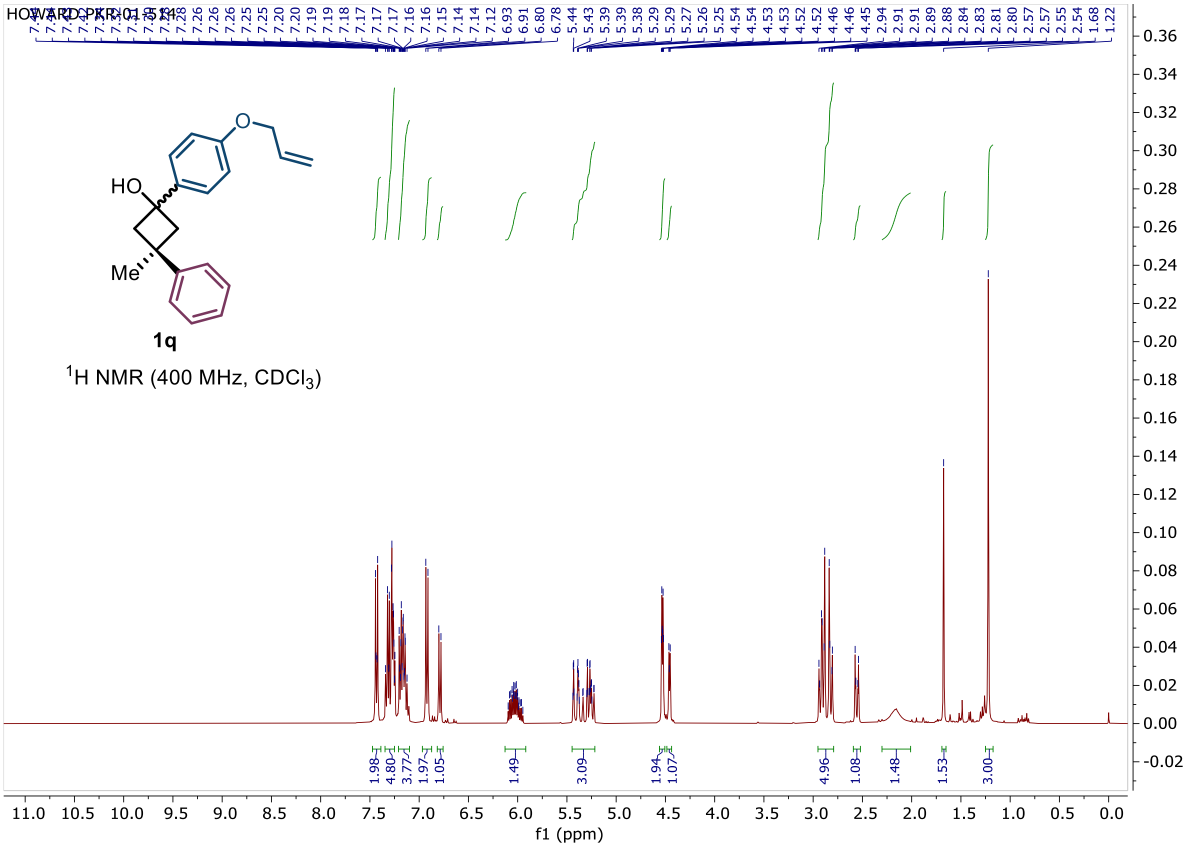
HOWARD PKR-01-514
HO
Me
1q
¹H NMR (400 MHz, CDCl₃)
f1 (ppm)

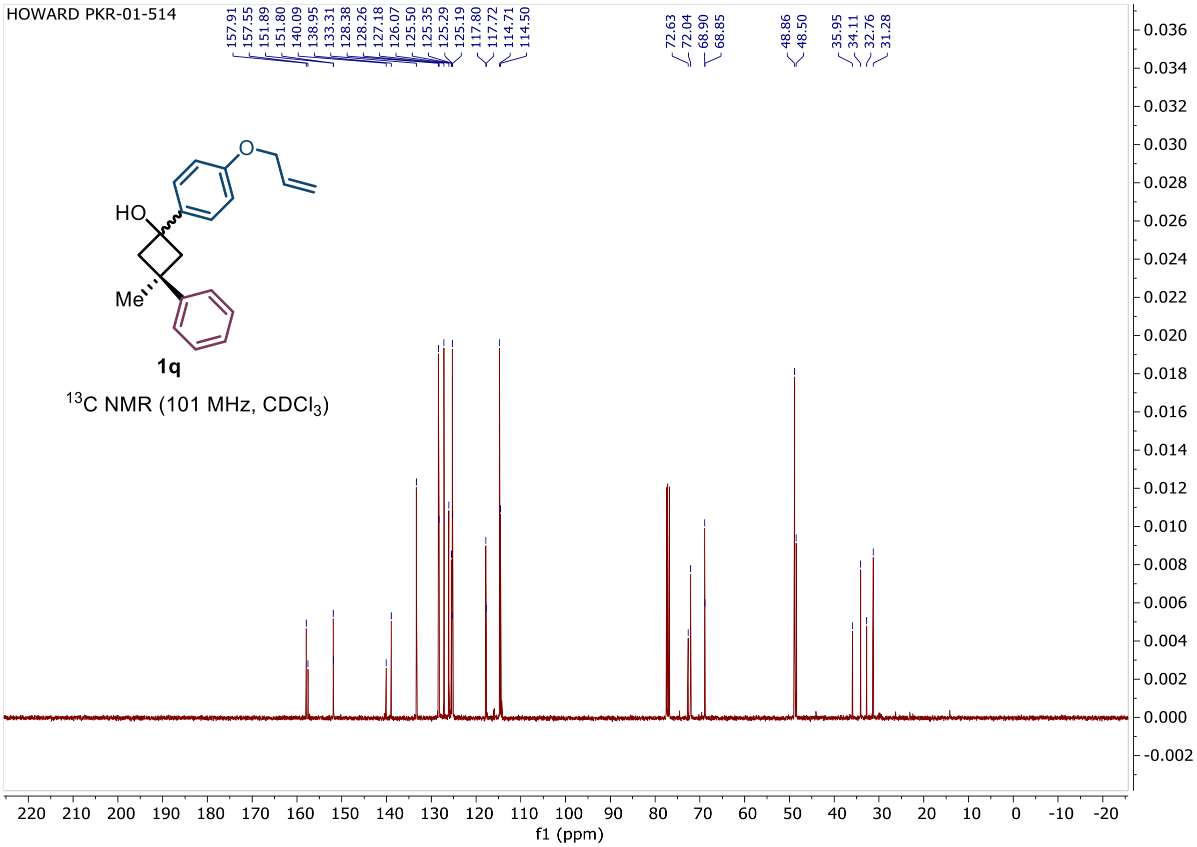
HOWARD PKR-01-514
HO
Me
1q
¹³C NMR (101 MHz, CDCl₃)
f1 (ppm)

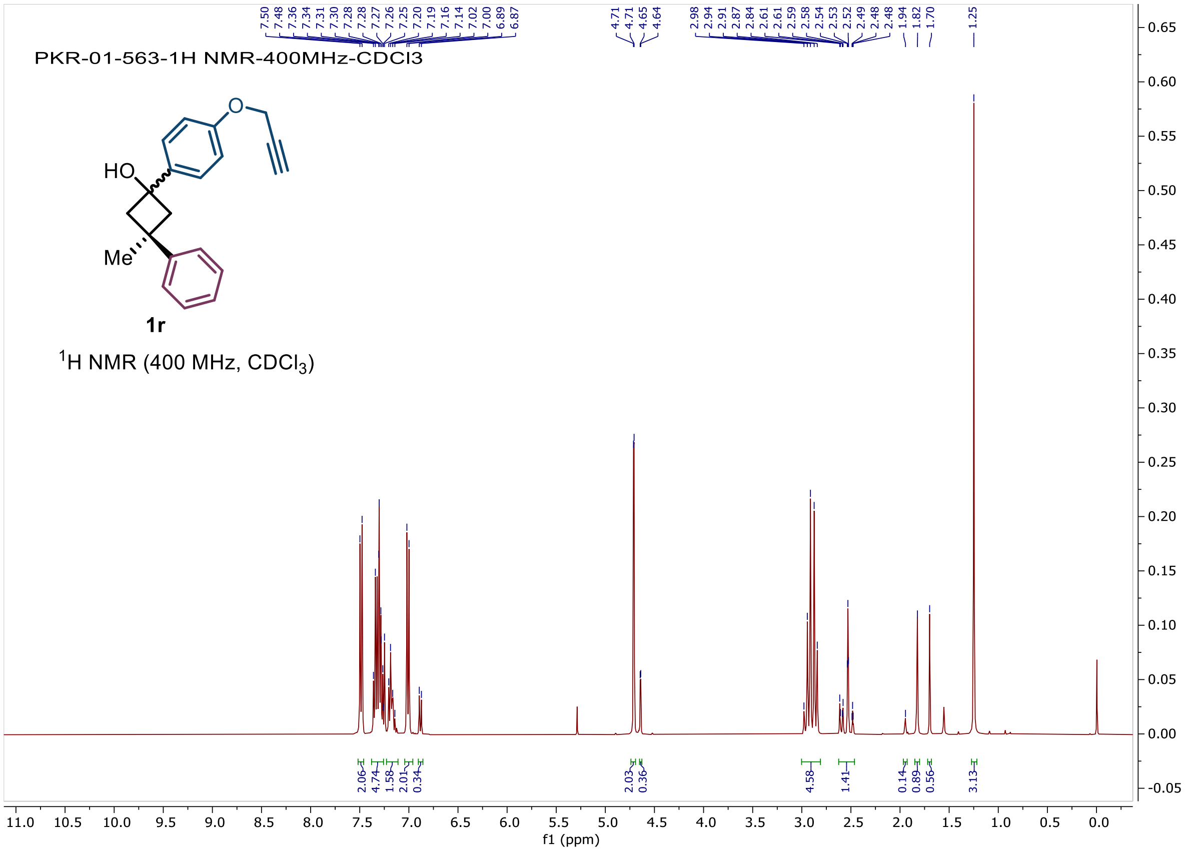
PKR-01-563-1H NMR-400MHz-CDCl3
7.50 7.48 7.36 7.34 7.31 7.30 7.28 7.28 7.27 7.26 7.25 7.25 7.20 7.19 7.16 7.14 7.02 7.00 6.89 6.87
4.71 4.71 4.65 4.64
2.98 2.94 2.91 2.87 2.84 2.61 2.61 2.59 2.58 2.54 2.53 2.52 2.49 2.48 2.48 1.94 1.82 1.70
1.25
HO
O
Me
1r
^{1}H NMR (400 MHz, $CDCl_3$)
2.06 4.74 1.58 2.01 0.34
2.03 0.36
4.58 1.41 0.14 0.89 0.56 3.13
f1 (ppm)

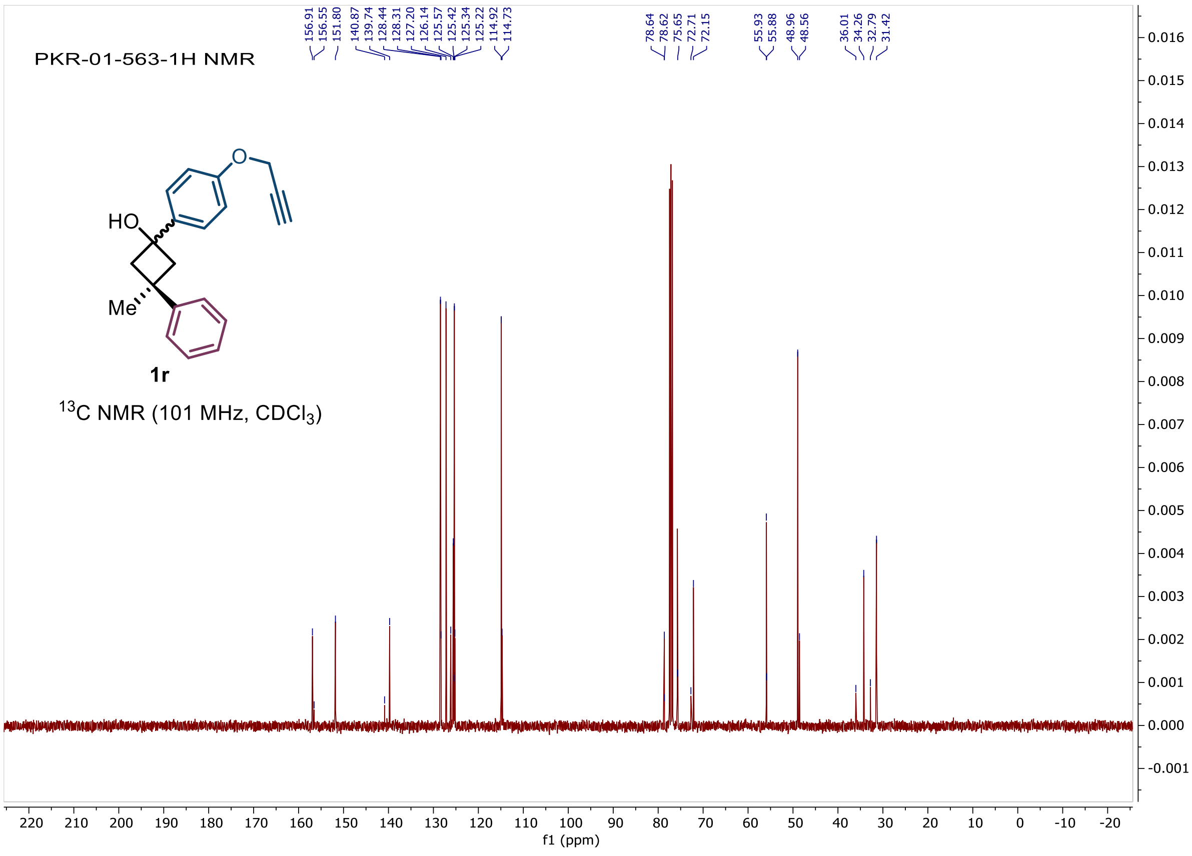
PKR-01-563-1H NMR
156.91 156.55 151.80 140.87 139.74 128.44 128.31 127.20 126.14 125.57 125.42 125.34 125.22 114.92 114.73
78.64 78.62 75.65 72.71 72.15
55.93 55.88 48.96 48.56
36.01 34.26 32.79 31.42
HO
O
Me
1r
^{13}C NMR (101 MHz, $CDCl_3$)
f1 (ppm)

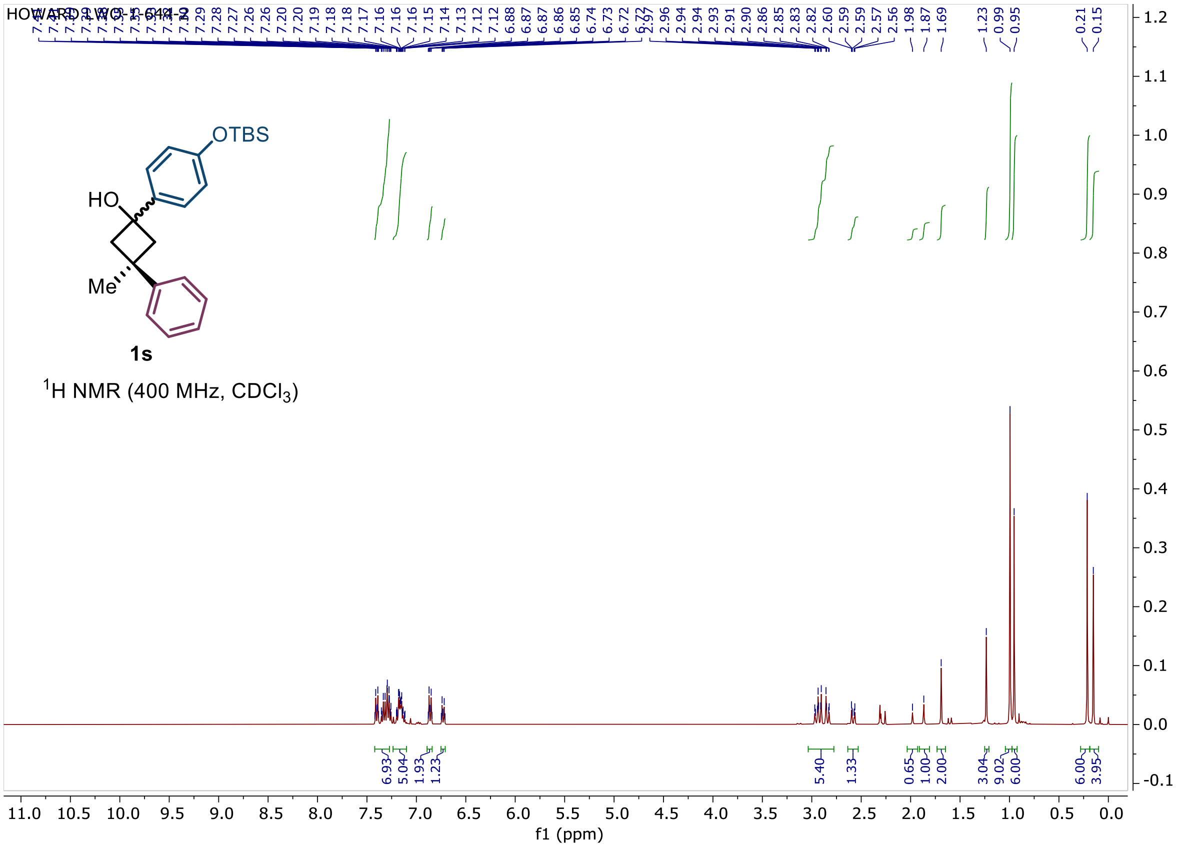
HOWARD LWO-1-644-2
OTBS
HO
Me
1s
1H NMR (400 MHz, CDCl3)
f1 (ppm)

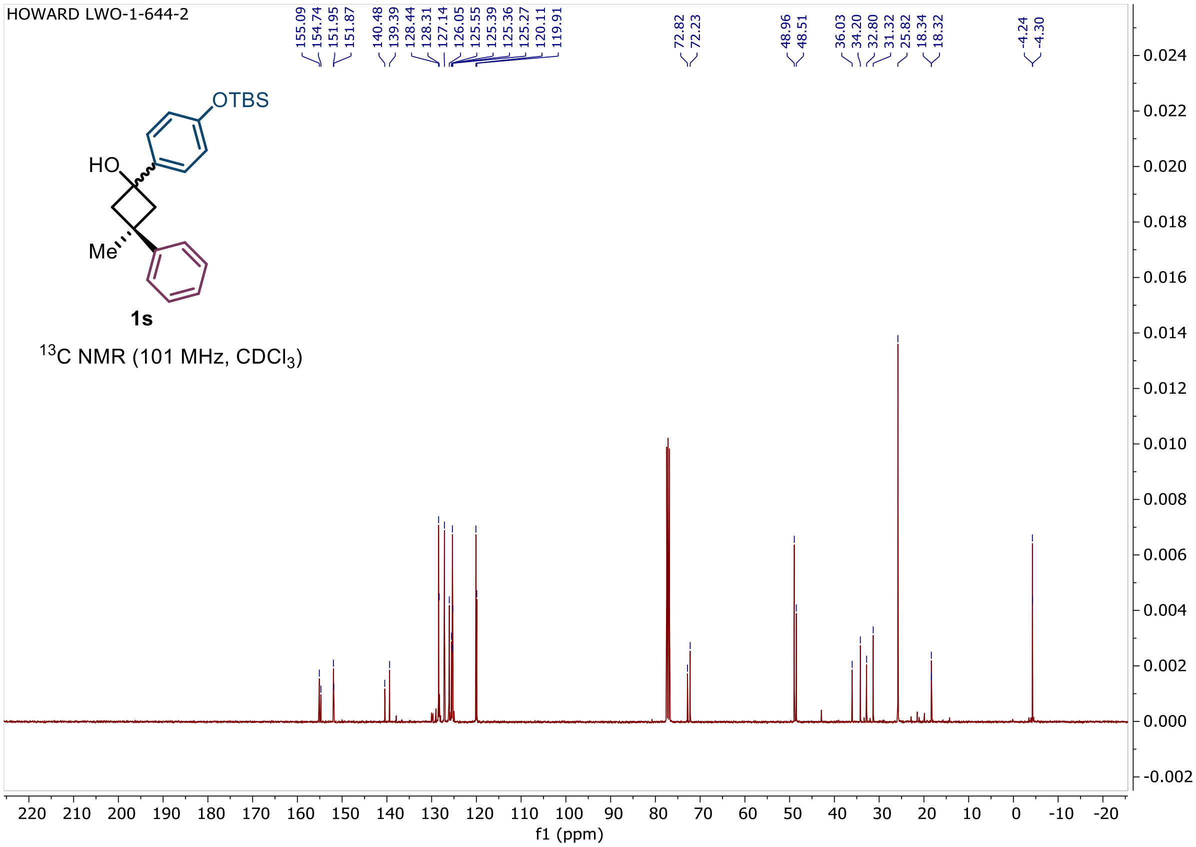
HOWARD LWO-1-644-2
155.09
154.74
151.95
151.87
140.48
139.39
128.44
128.31
127.14
126.05
125.55
125.39
125.36
125.27
120.11
119.91
72.82
72.23
48.96
48.51
36.03
34.20
32.80
31.32
25.82
18.34
18.32
-4.24
-4.30
OTBS
HO
Me
1s
13C NMR (101 MHz, CDCl3)
f1 (ppm)

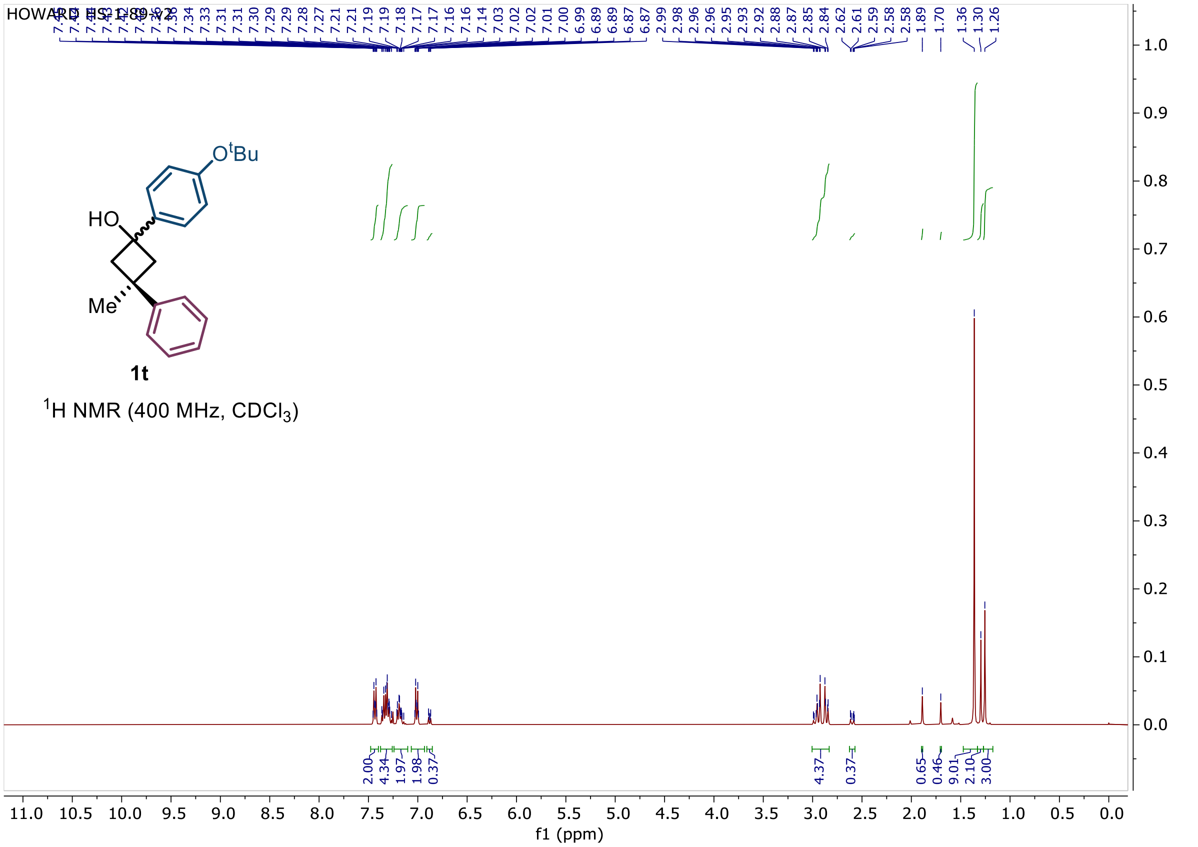
HOWARD HS-1-89-v2
7.43 7.42 7.41 7.41 7.34 7.33 7.31 7.31 7.30 7.29 7.29 7.28 7.27 7.21 7.21 7.19 7.19 7.18 7.17 7.17 7.16 7.16 7.14 7.03 7.02 7.02 7.01 7.00 6.99 6.89 6.89 6.87 2.99 2.98 2.96 2.96 2.95 2.93 2.92 2.88 2.87 2.85 2.84 2.62 2.61 2.59 2.58 1.89 1.70 1.36 1.30 1.26
O^{t}Bu
HO
Me
1t
^{1}H NMR (400 MHz, $CDCl_3$)
2.00 4.34 1.97 1.98 0.37 4.37 0.37 0.65 0.46 9.01 2.10 3.00
f1 (ppm)

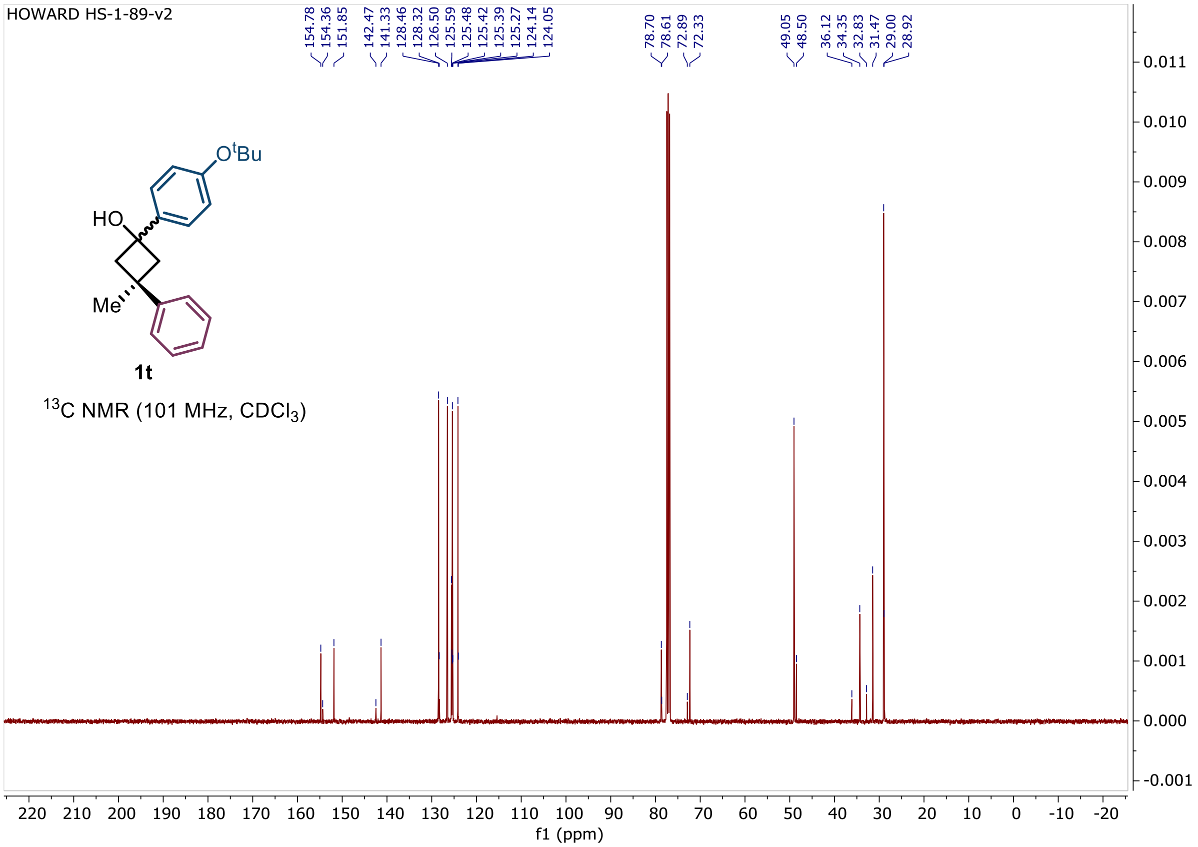
HOWARD HS-1-89-v2
154.78 154.36 151.85 142.47 141.33 128.46 128.32 126.50 125.59 125.48 125.42 125.39 125.27 124.14 124.05 78.70 78.61 72.89 72.33 49.05 48.50 36.12 34.35 32.83 31.47 29.00 28.92
O^{t}Bu
HO
Me
1t
^{13}C NMR (101 MHz, $CDCl_3$)
f1 (ppm)

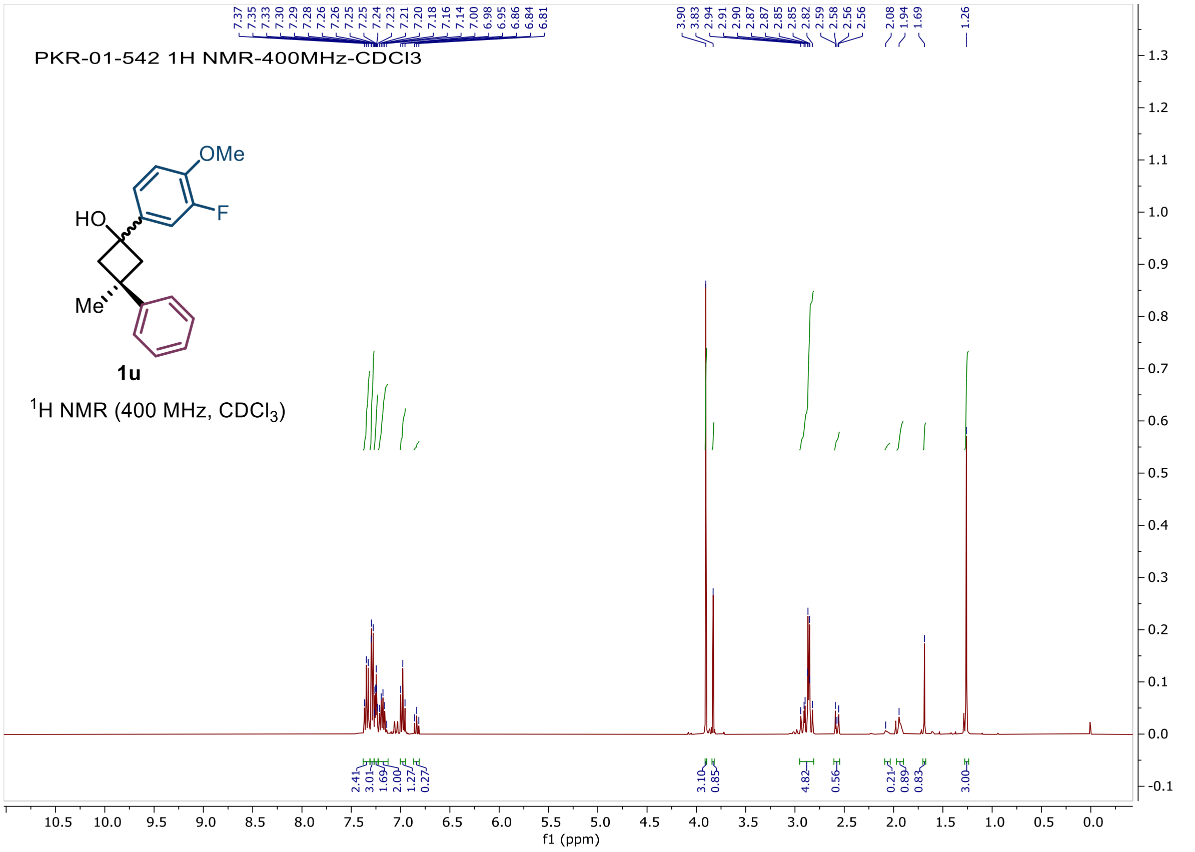
PKR-01-542 1H NMR-400MHz-CDCl3
7.37 7.35 7.33 7.30 7.29 7.28 7.26 7.26 7.25 7.25 7.24 7.23 7.21 7.20 7.18 7.16 7.14 7.00 6.98 6.95 6.86 6.84 6.81
3.90 3.83 2.94 2.91 2.90 2.87 2.87 2.85 2.85 2.82 2.59 2.58 2.56 2.56 2.08 1.94 1.69 1.26
OMe
HO
F
Me
1u
1H NMR (400 MHz, CDCl3)
2.41 3.01 1.69 2.00 1.27 0.27 3.10 0.85 4.82 0.56 0.21 0.89 0.83 3.00
f1 (ppm)

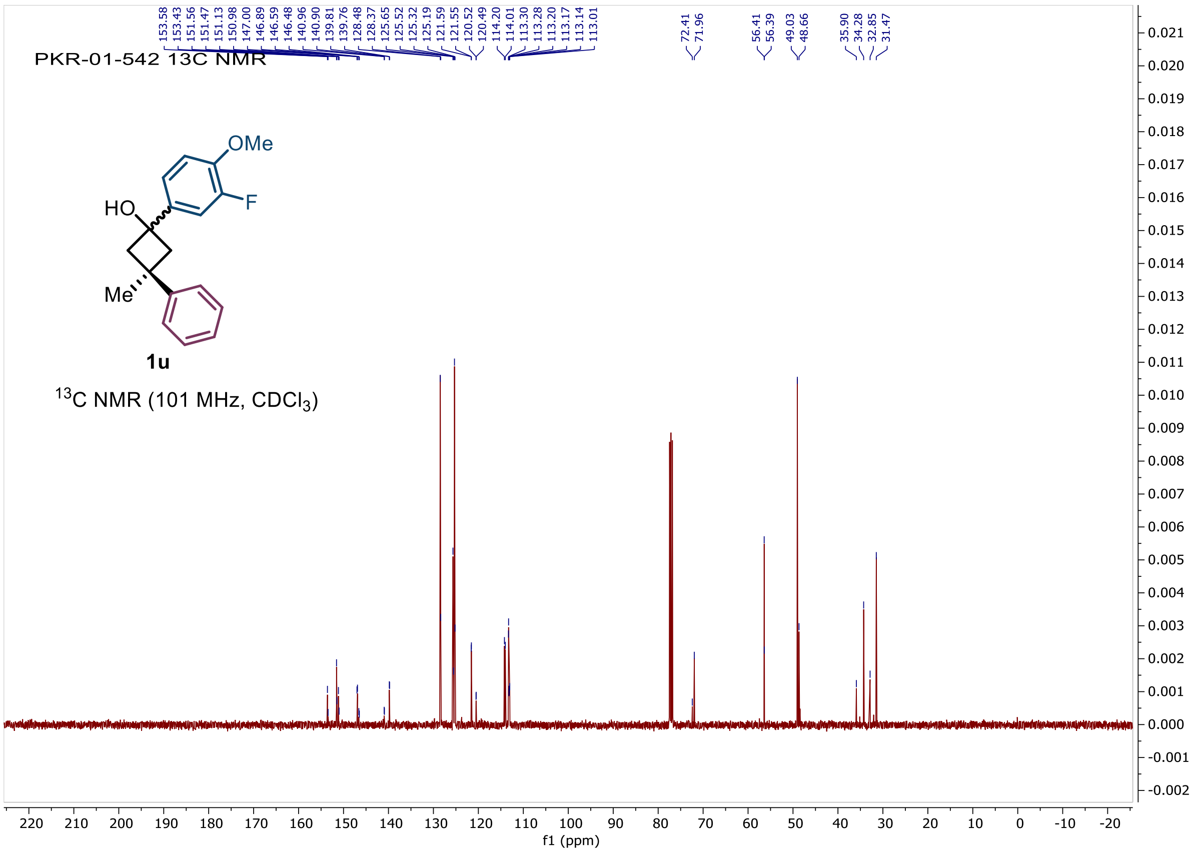
PKR-01-542 13C NMR
153.58 153.43 151.56 151.47 151.13 150.98 147.00 146.89 146.59 146.48 140.96 140.90 139.81 139.76 128.48 128.37 125.65 125.52 125.32 125.19 121.59 121.55 120.52 120.49 114.20 114.01 113.30 113.28 113.20 113.17 113.14 113.01
72.41 71.96 56.41 56.39 49.03 48.66 35.90 34.28 32.85 31.47
OMe
HO
F
Me
1u
13C NMR (101 MHz, CDCl3)
f1 (ppm)

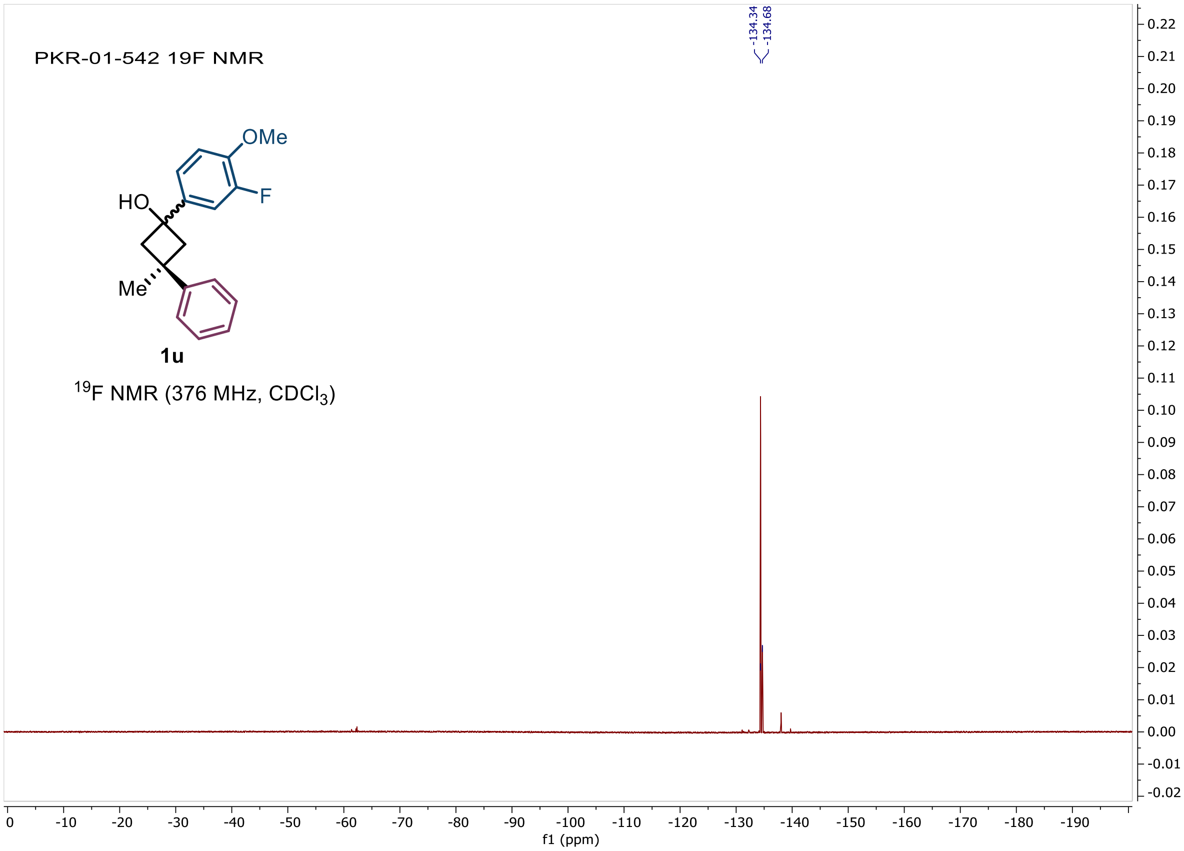
PKR-01-542 19F NMR
OMe
F
HO
Me
1u
^{19}F NMR (376 MHz, $CDCl_3$)
-134.34
-134.68
f1 (ppm)

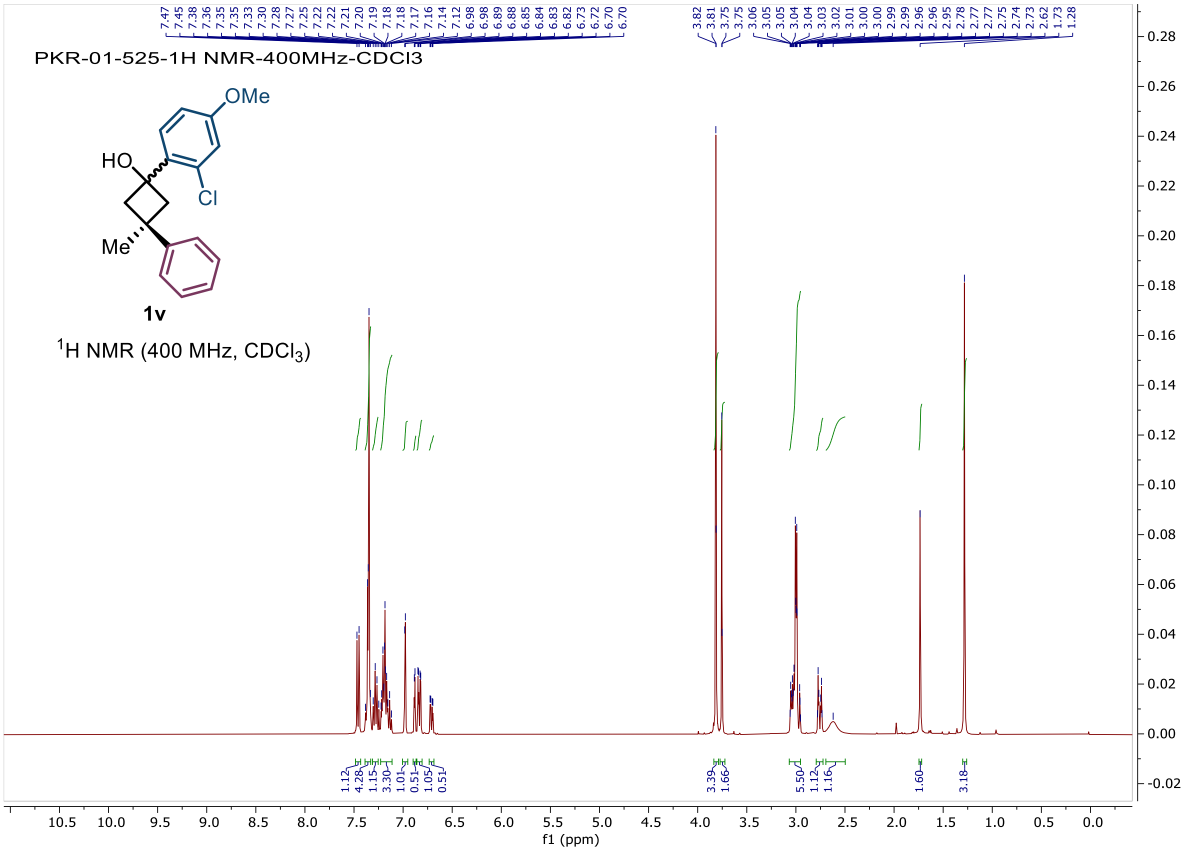
PKR-01-525-1H NMR-400MHz-CDCl3
OMe
HO
Cl
Me
1v
^{1}H NMR (400 MHz, $CDCl_3$)
7.47 7.45 7.38 7.36 7.35 7.33 7.30 7.28 7.27 7.25 7.22 7.22 7.21 7.20 7.19 7.18 7.18 7.17 7.16 7.14 7.12 6.98 6.98 6.89 6.88 6.85 6.84 6.83 6.82 6.73 6.72 6.70 6.70
3.82 3.81 3.75 3.75 3.06 3.05 3.05 3.04 3.04 3.03 3.02 3.01 3.00 2.99 2.99 2.96 2.95 2.78 2.77 2.77 2.75 2.74 2.73 2.62 1.73 1.28
1.12 4.28 1.15 3.30 1.01 0.51 1.05 0.51
3.39 1.66
5.50 1.12 1.16
1.60
3.18
f1 (ppm)

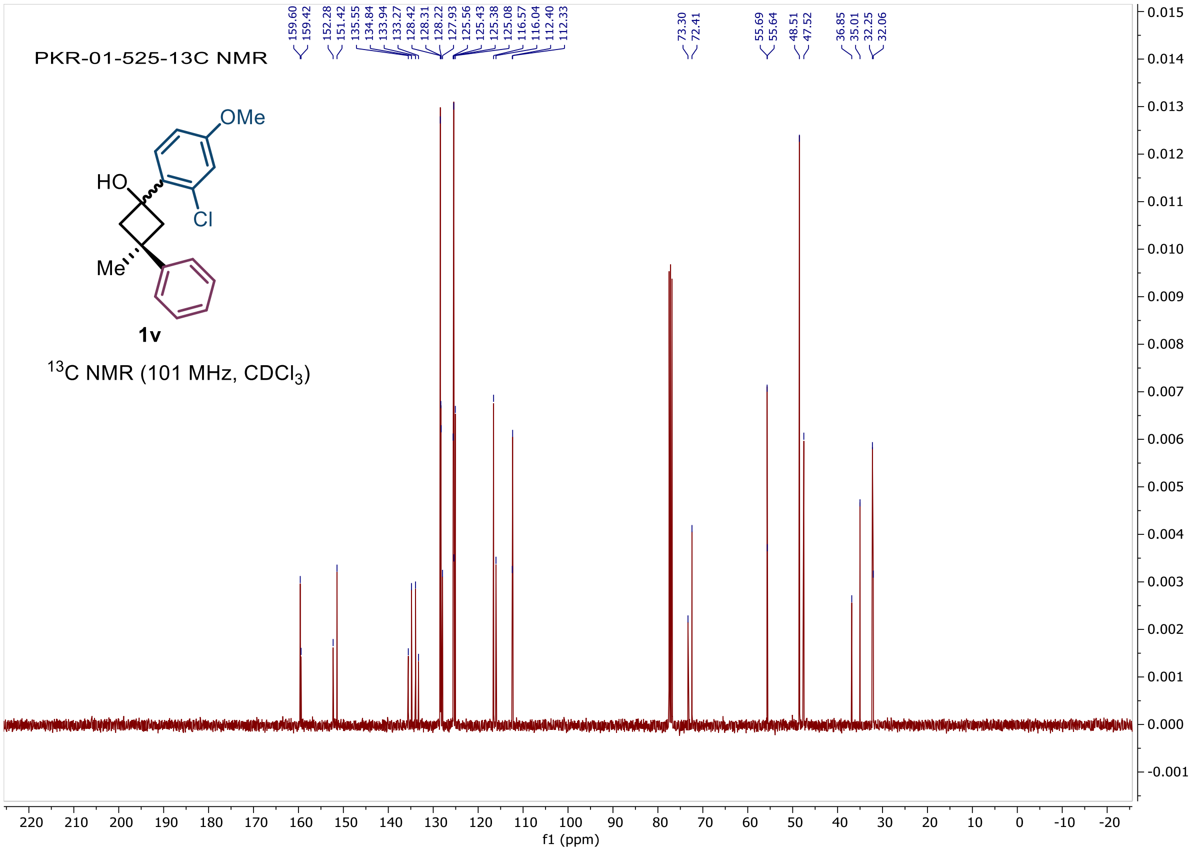
PKR-01-525-13C NMR
159.60
159.42
152.28
151.42
135.55
134.84
133.94
133.27
128.42
128.31
128.22
127.93
125.56
125.43
125.38
125.08
116.57
116.04
112.40
112.33
73.30
72.41
55.69
55.64
48.51
47.52
36.85
35.01
32.25
32.06
OMe
HO
Cl
Me
1v
^{13}C NMR (101 MHz, CDCl$_3$)
f1 (ppm)

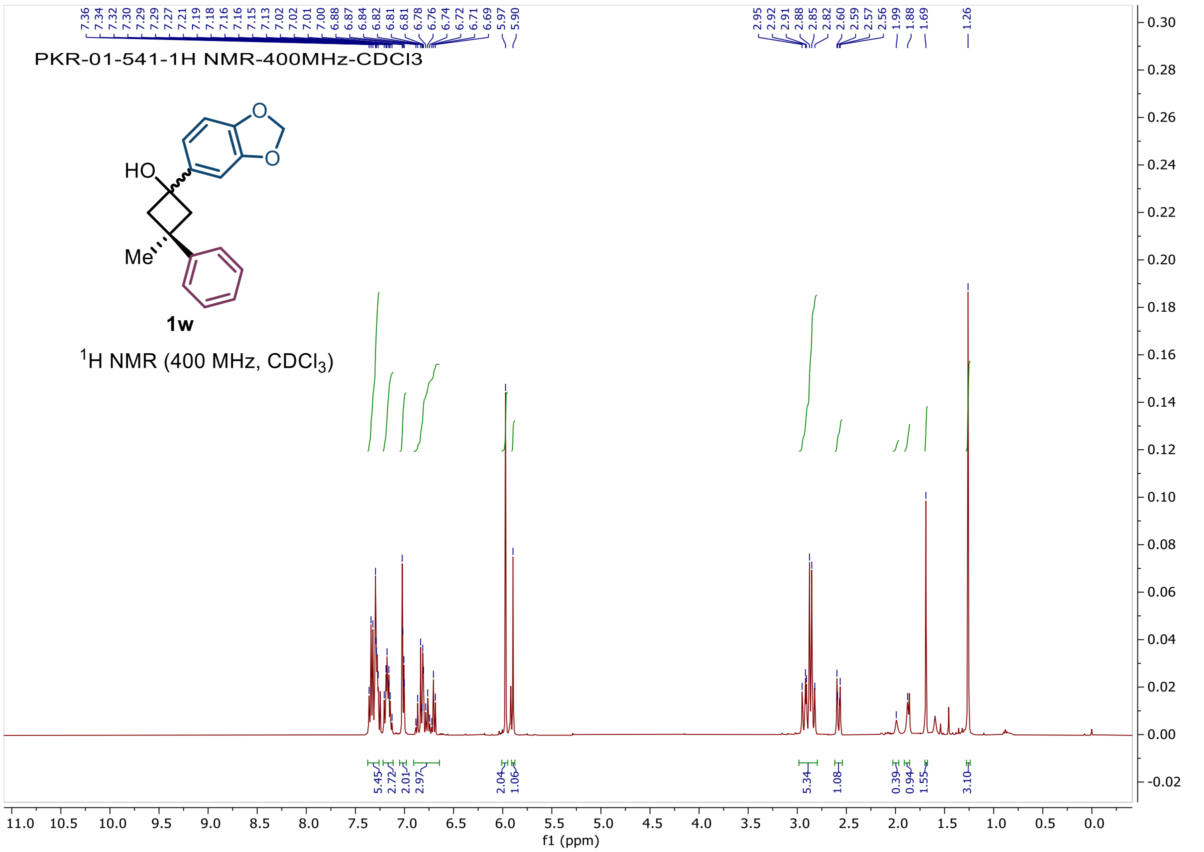
PKR-01-541-1H NMR-400MHz-CDCl3
7.36
7.34
7.32
7.30
7.29
7.29
7.27
7.21
7.19
7.18
7.16
7.16
7.15
7.13
7.13
7.02
7.02
7.01
7.00
6.88
6.87
6.84
6.82
6.81
6.81
6.78
6.76
6.74
6.72
6.71
6.69
5.97
5.90
2.95
2.92
2.91
2.88
2.85
2.82
2.60
2.59
2.57
2.56
1.99
1.88
1.69
1.26
HO
O
O
Me
1w
^{1}H NMR (400 MHz, CDCl$_3$)
5.45
2.72
2.01
2.97
2.04
1.06
5.34
1.08
0.39
0.94
1.55
3.10
f1 (ppm)

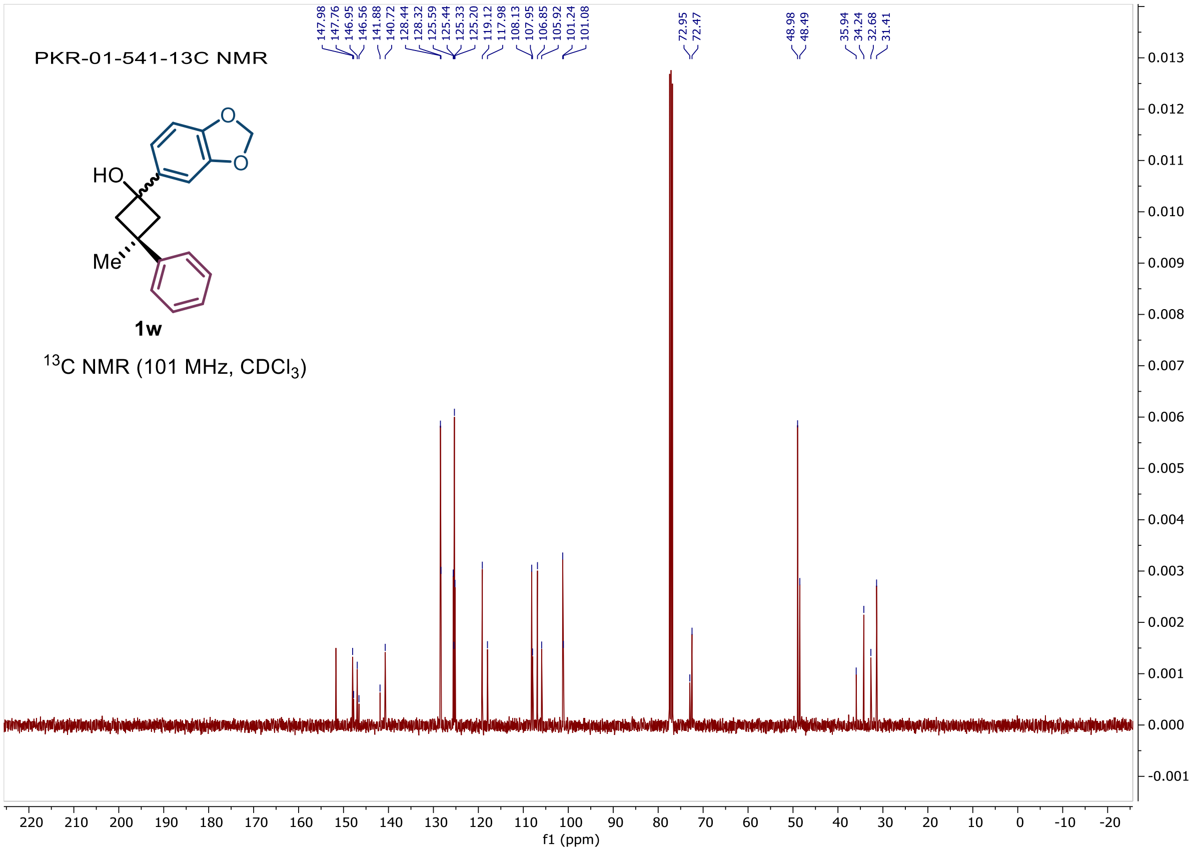
PKR-01-541-13C NMR
1w
^{13}C NMR (101 MHz, CDCl$_3$)
147.98 147.76 146.95 146.56 141.88 140.72 128.44 128.32 125.59 125.44 125.33 125.20 119.12 117.98 108.13 107.95 106.85 105.92 101.24 101.08
72.95 72.47
48.98 48.49
35.94 34.24 32.68 31.41
f1 (ppm)

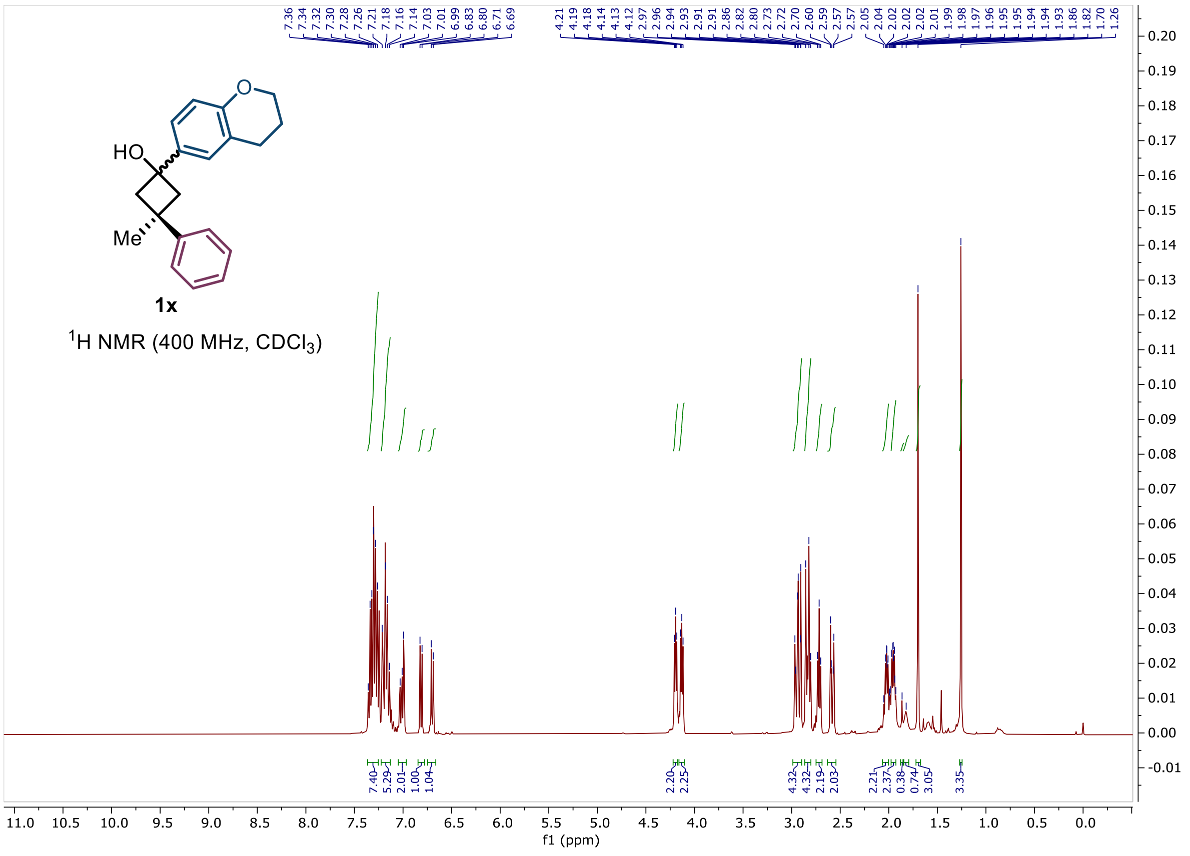
1x
^{1}H NMR (400 MHz, CDCl$_3$)
7.36 7.34 7.32 7.30 7.28 7.26 7.21 7.18 7.16 7.14 7.03 7.01 6.99 6.83 6.80 6.71 6.69
4.21 4.19 4.18 4.14 4.13 4.12 2.97 2.96 2.94 2.93 2.91 2.91 2.86 2.82 2.80 2.73 2.72 2.70 2.60 2.59 2.57 2.57 2.05 2.04 2.02 2.02 2.02 2.01 1.99 1.98 1.97 1.96 1.95 1.95 1.94 1.94 1.93 1.86 1.82 1.70 1.26
7.40 5.29 2.01 1.00 1.04 2.20 2.25 4.32 4.32 2.19 2.03 2.21 2.37 0.38 0.74 3.05 3.35
f1 (ppm)

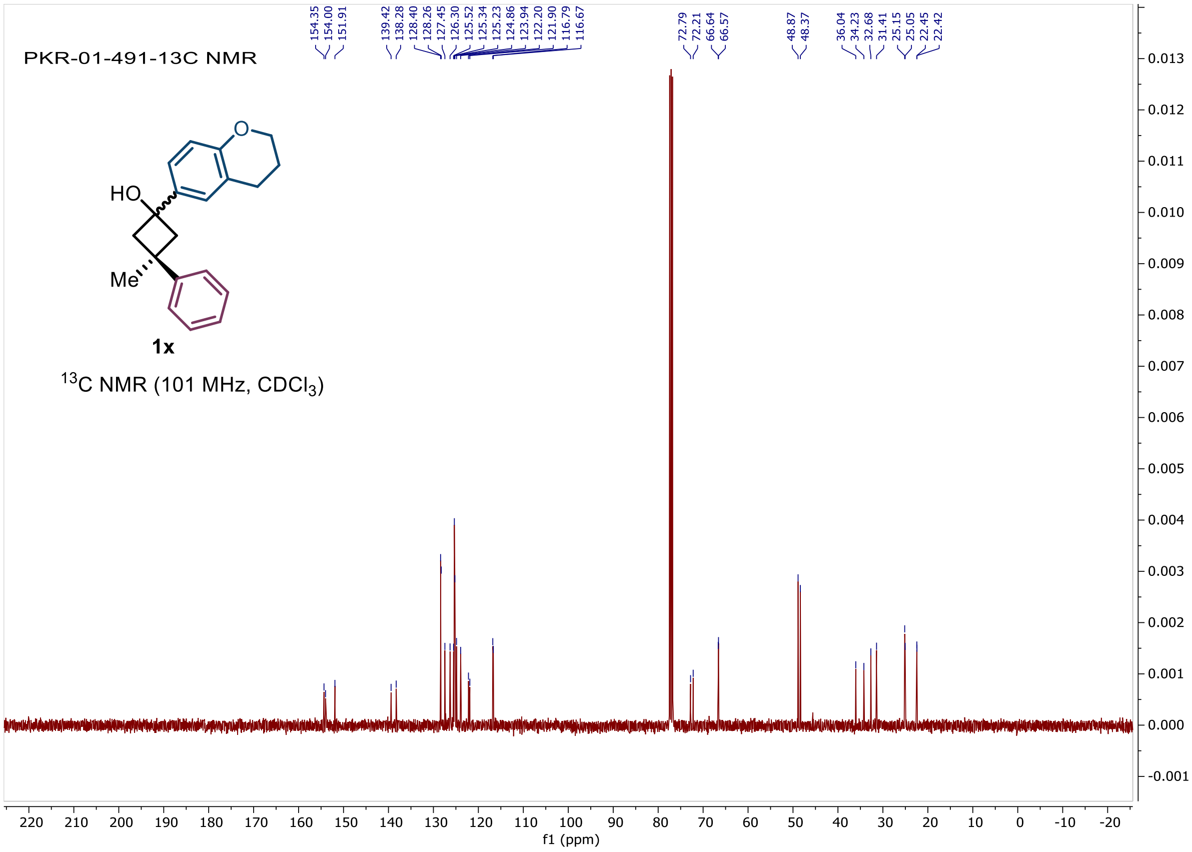
PKR-01-491-13C NMR
1x
^{13}C NMR (101 MHz, CDCl$_3$)
f1 (ppm)

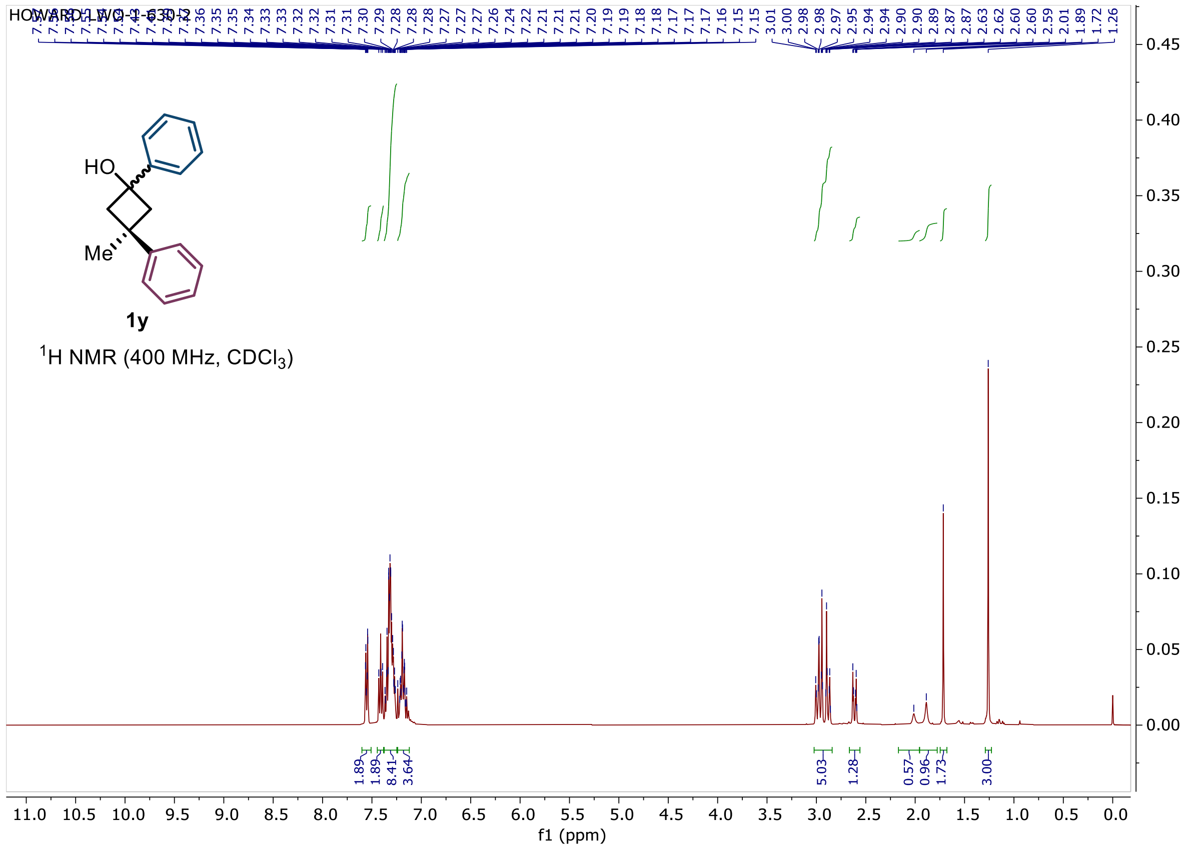
1y
^{1}H NMR (400 MHz, CDCl$_3$)
f1 (ppm)

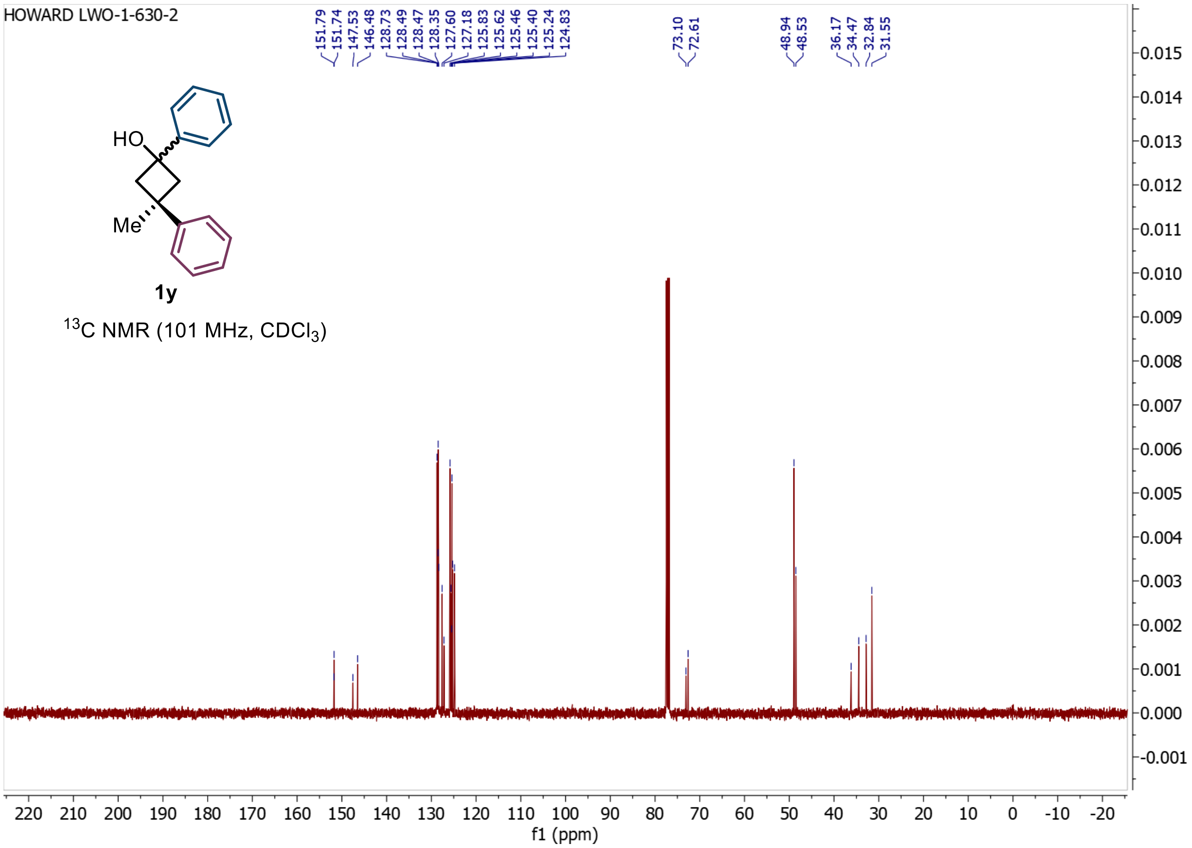
HOWARD LWO-1-630-2
151.79 151.74 147.53 146.48 128.73 128.49 128.47 128.35 127.60 127.18 125.83 125.62 125.46 125.40 125.24 124.83
73.10 72.61
48.94 48.53
36.17 34.47 32.84 31.55
HO
Me
1y
13C NMR (101 MHz, CDCl3)
f1 (ppm)

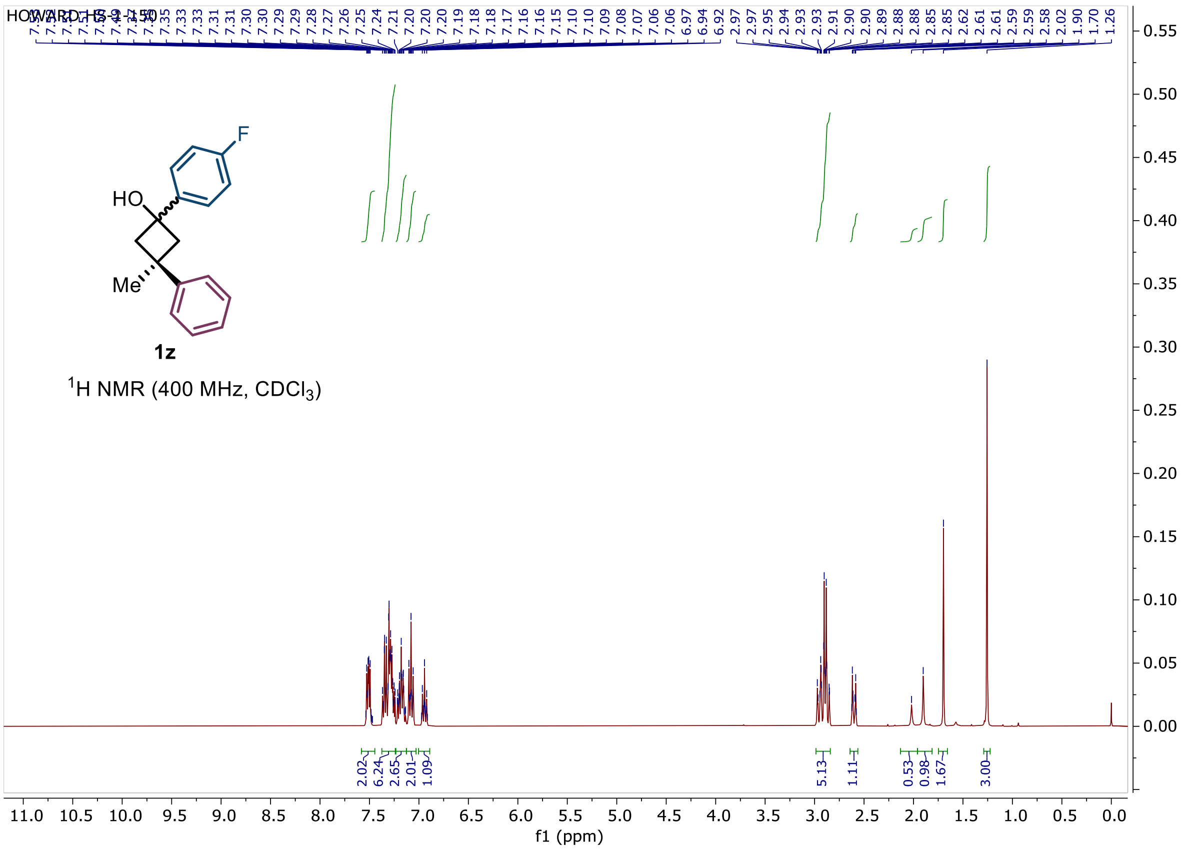
7.35 7.33 7.33 7.31 7.30 7.30 7.29 7.28 7.27 7.26 7.25 7.24 7.21 7.20 7.20 7.19 7.18 7.18 7.17 7.16 7.15 7.10 7.09 7.08 7.07 7.06 6.97 6.94 6.92 2.97 2.97 2.95 2.94 2.93 2.93 2.91 2.90 2.90 2.89 2.88 2.85 2.62 2.61 2.61 2.59 2.59 2.58 2.02 1.90 1.70 1.26
F
HO
Me
1z
1H NMR (400 MHz, CDCl3)
2.02 6.24 2.65 2.01 1.09 5.13 1.11 0.53 0.98 1.67 3.00
f1 (ppm)

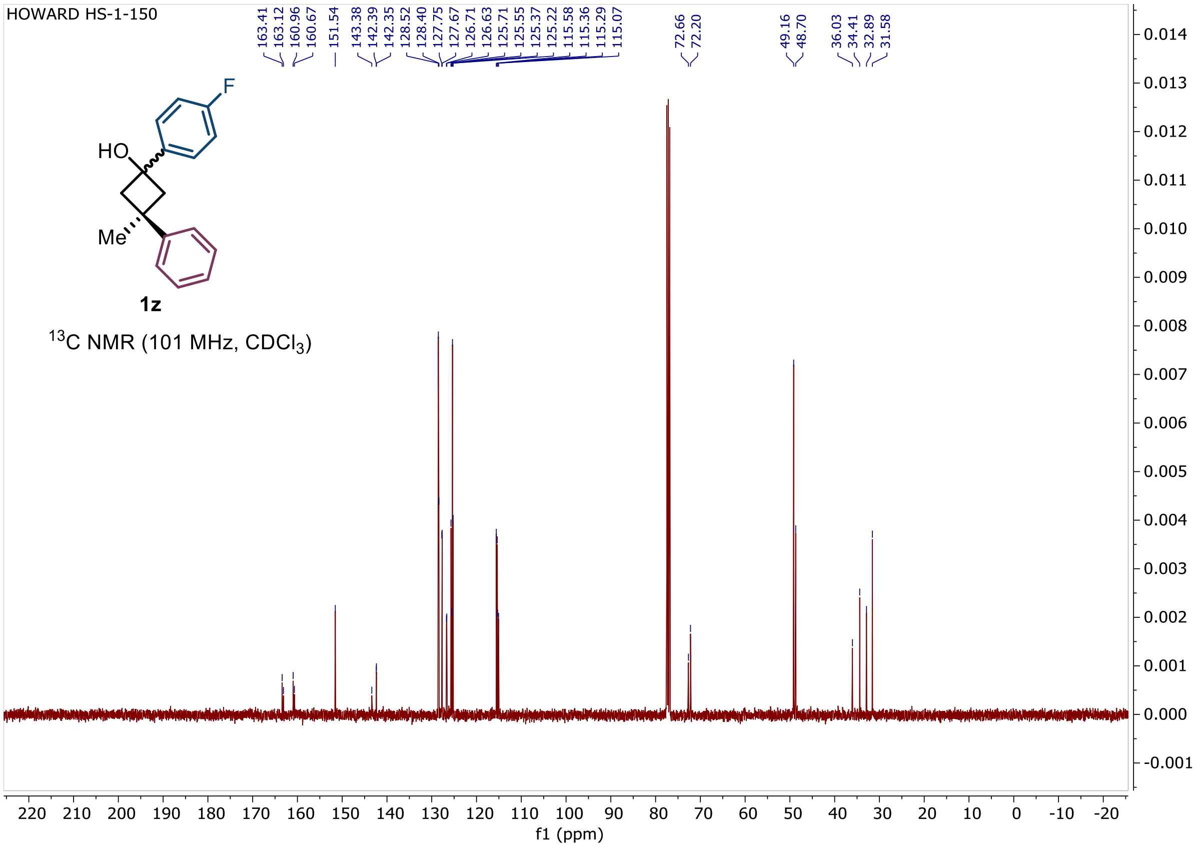
HOWARD HS-1-150
163.41
163.12
160.96
160.67
151.54
143.38
142.39
142.35
128.52
128.40
127.75
127.67
126.71
126.63
125.71
125.55
125.37
125.22
115.58
115.36
115.29
115.07
72.66
72.20
49.16
48.70
36.03
34.41
32.89
31.58
F
HO
Me
1z
13C NMR (101 MHz, CDCl3)
f1 (ppm)

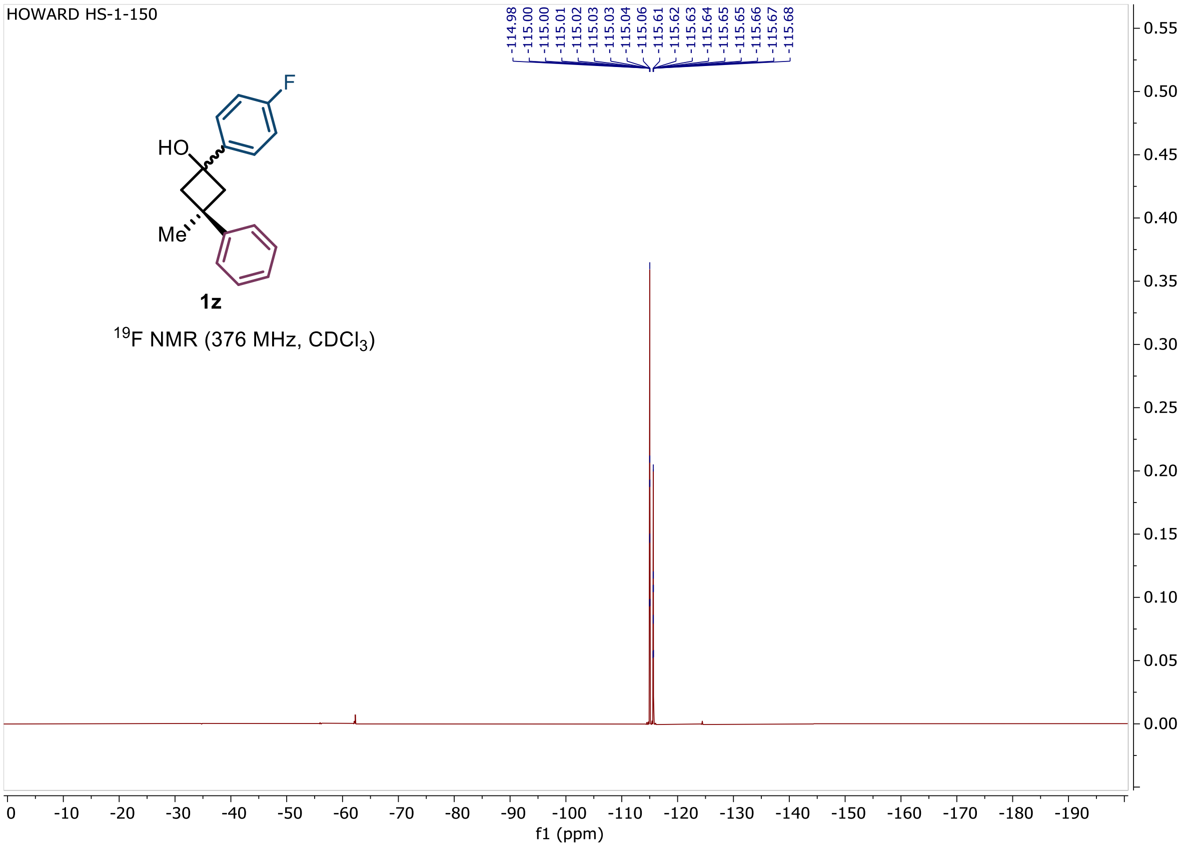
HOWARD HS-1-150
-114.98
-115.00
-115.00
-115.01
-115.02
-115.03
-115.04
-115.06
-115.61
-115.62
-115.63
-115.64
-115.65
-115.66
-115.67
-115.68
F
HO
Me
1z
19F NMR (376 MHz, CDCl3)
f1 (ppm)

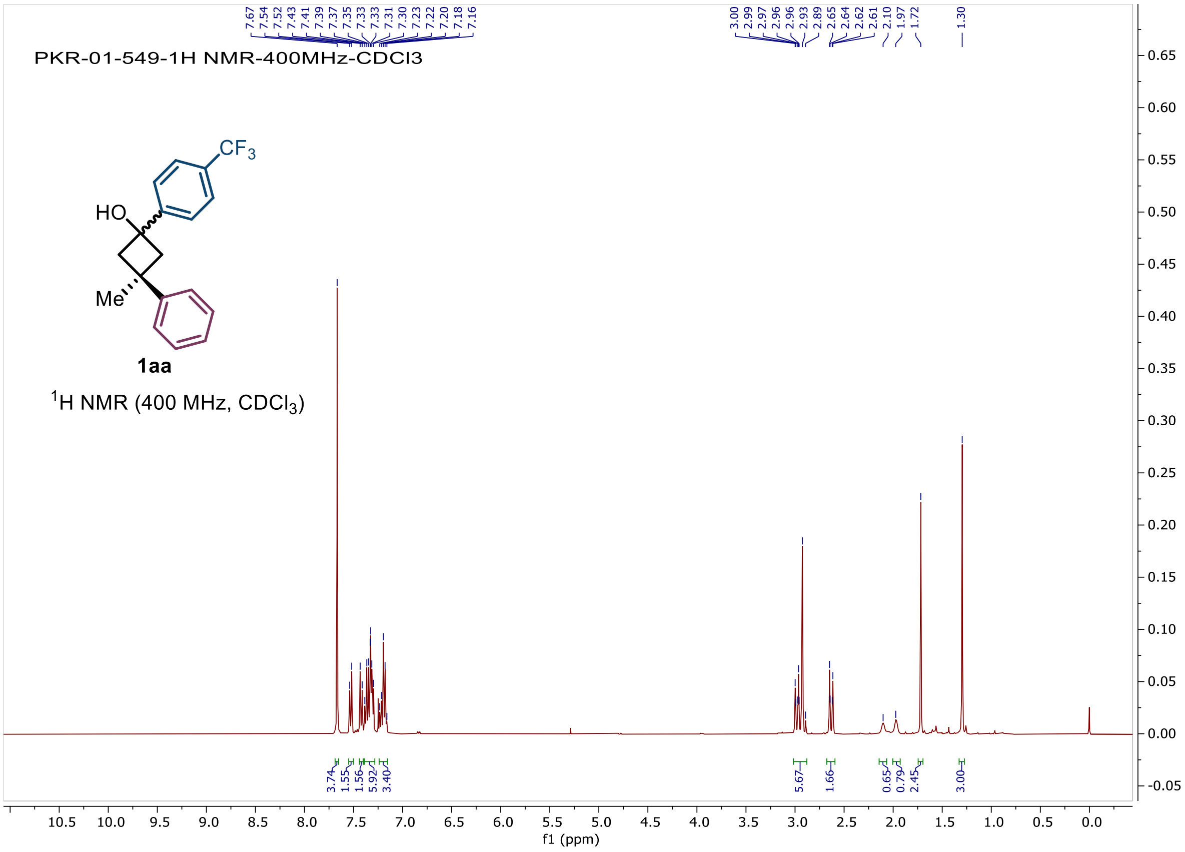
PKR-01-549-1H NMR-400MHz-CDCl3
CF3
HO
Me
1aa
1H NMR (400 MHz, CDCl3)
f1 (ppm)

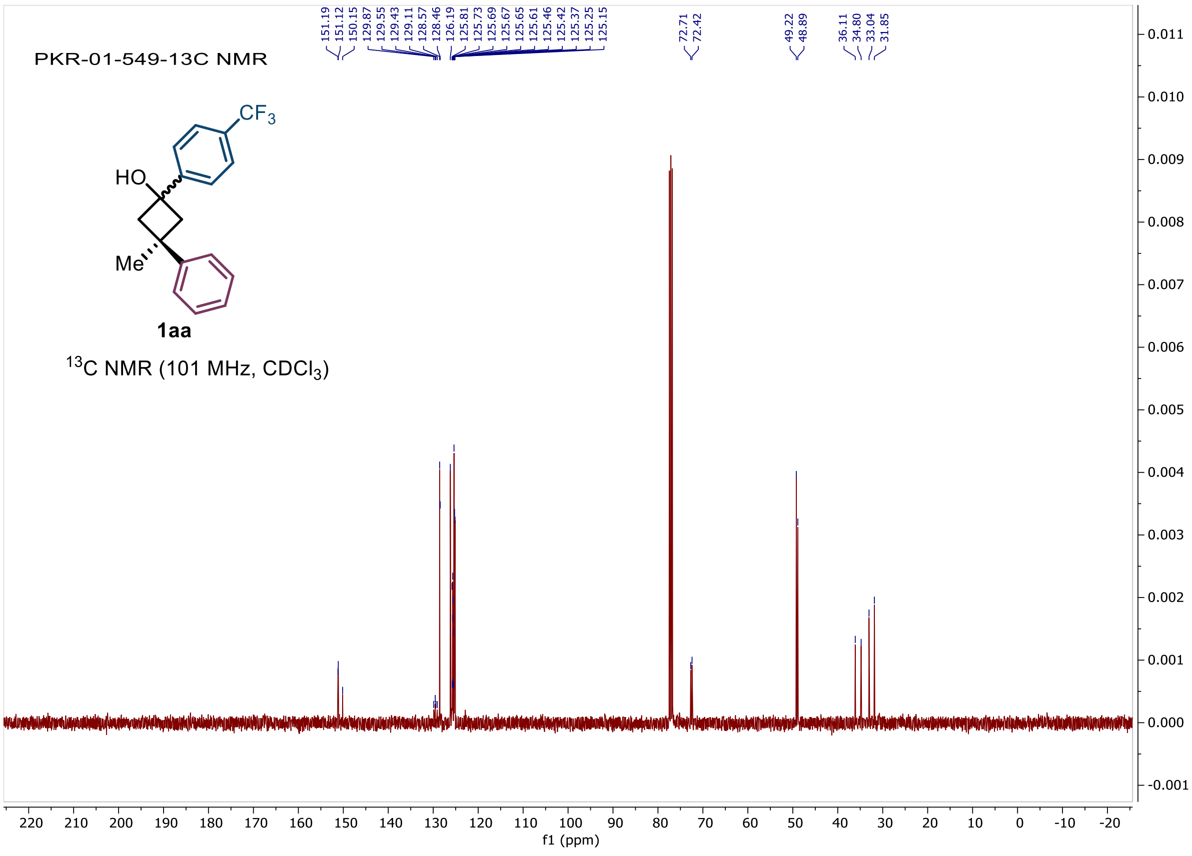
PKR-01-549-13C NMR
CF3
HO
Me
1aa
13C NMR (101 MHz, CDCl3)
f1 (ppm)

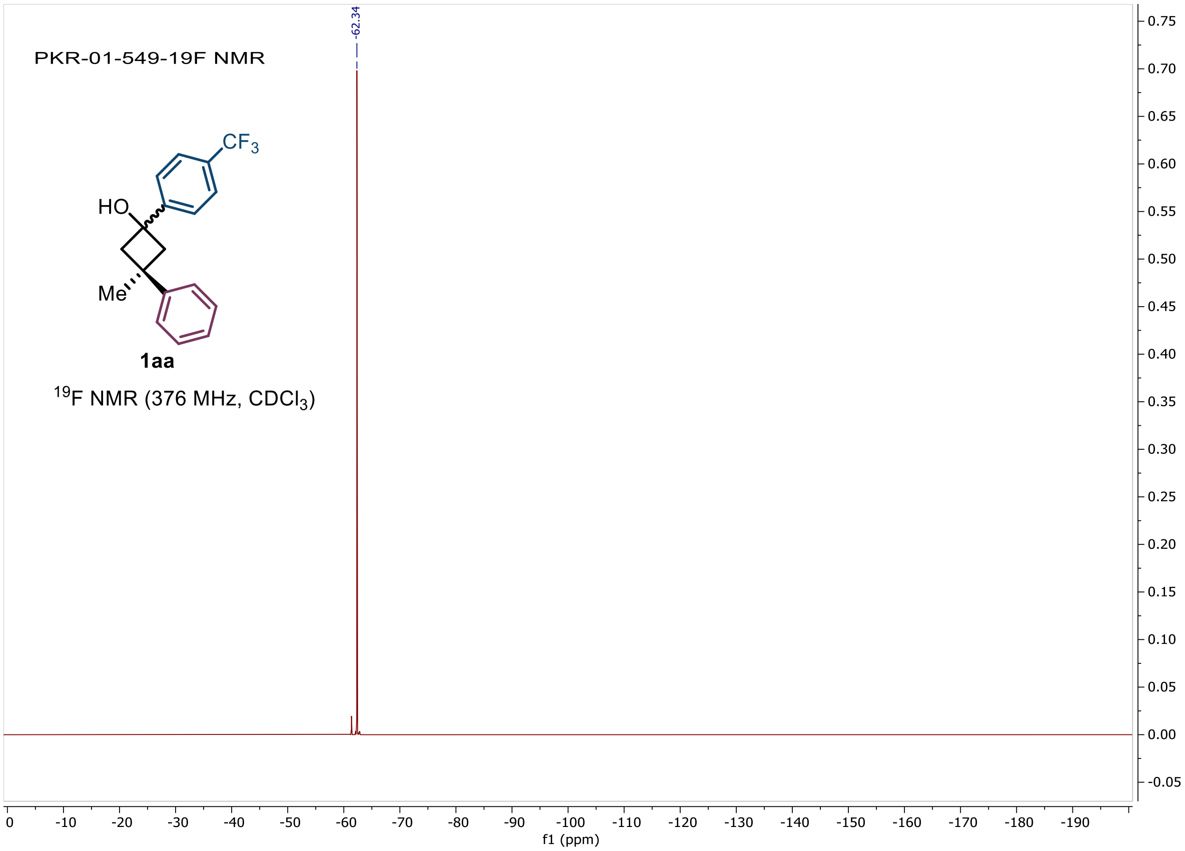
PKR-01-549-19F NMR
CF3
HO
Me
1aa
19F NMR (376 MHz, CDCl3)
-62.34
f1 (ppm)

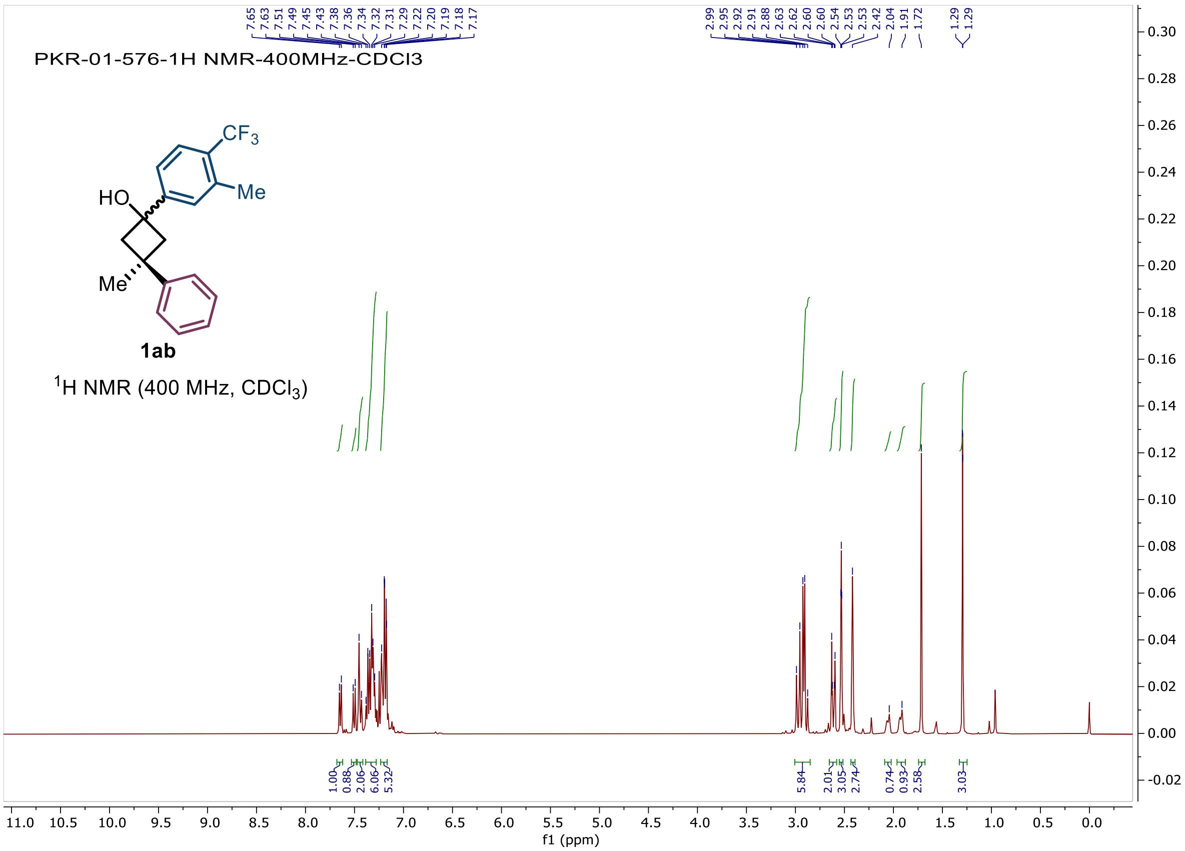
PKR-01-576-1H NMR-400MHz-CDCl3
CF3
Me
HO
Me
1ab
1H NMR (400 MHz, CDCl3)
7.65 7.63 7.51 7.49 7.45 7.43 7.38 7.36 7.34 7.32 7.31 7.29 7.22 7.20 7.19 7.18 7.17
2.99 2.95 2.92 2.91 2.88 2.63 2.62 2.60 2.54 2.53 2.53 2.42 2.04 1.91 1.72 1.29 1.29
1.00 0.88 2.06 6.06 5.32 5.84 2.01 3.05 2.74 0.74 0.93 2.58 3.03
f1 (ppm)

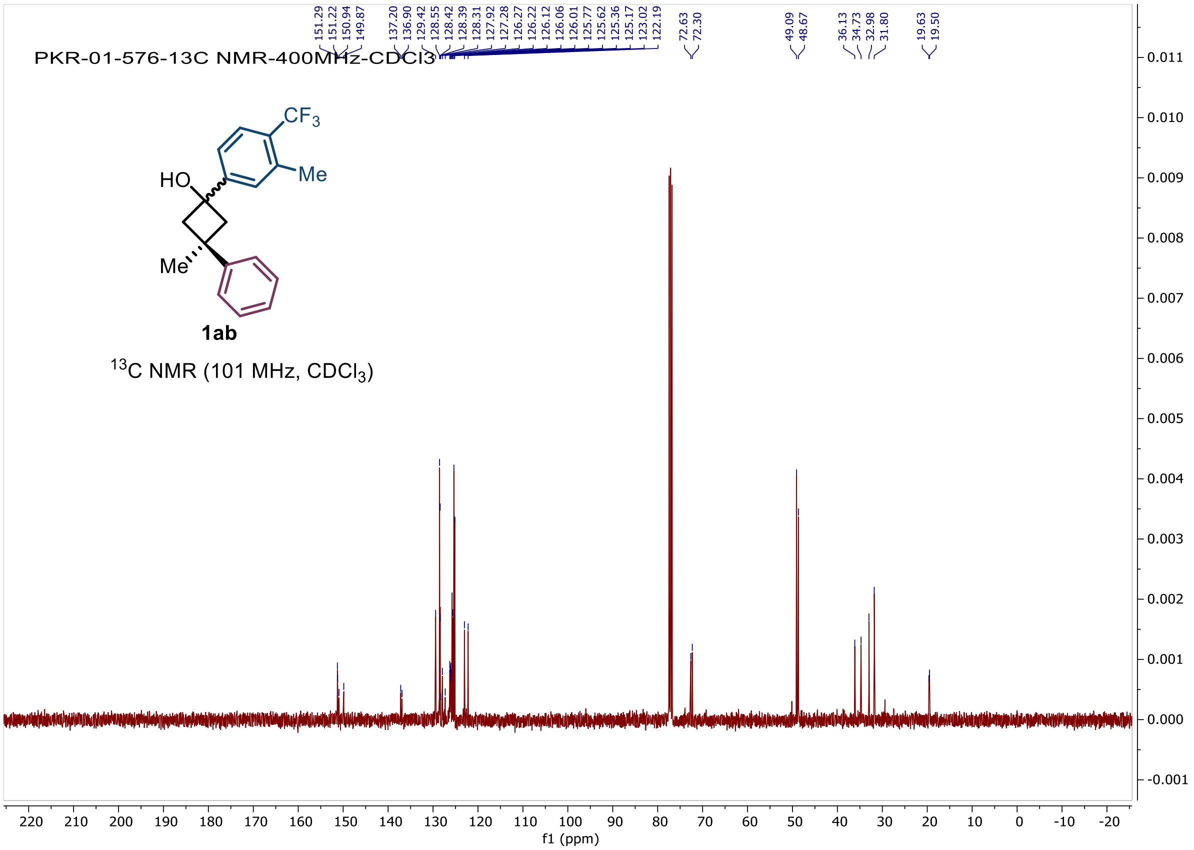
PKR-01-576-13C NMR-400MHz-CDCl3
1ab
^{13}C NMR (101 MHz, CDCl$_3$)
151.29
151.22
150.94
149.87
137.20
136.90
129.42
128.55
128.42
128.39
128.31
127.92
127.28
126.27
126.22
126.12
126.06
126.01
125.77
125.62
125.36
125.17
123.02
122.19
72.63
72.30
49.09
48.67
36.13
34.73
32.98
31.80
19.63
19.50
f1 (ppm)

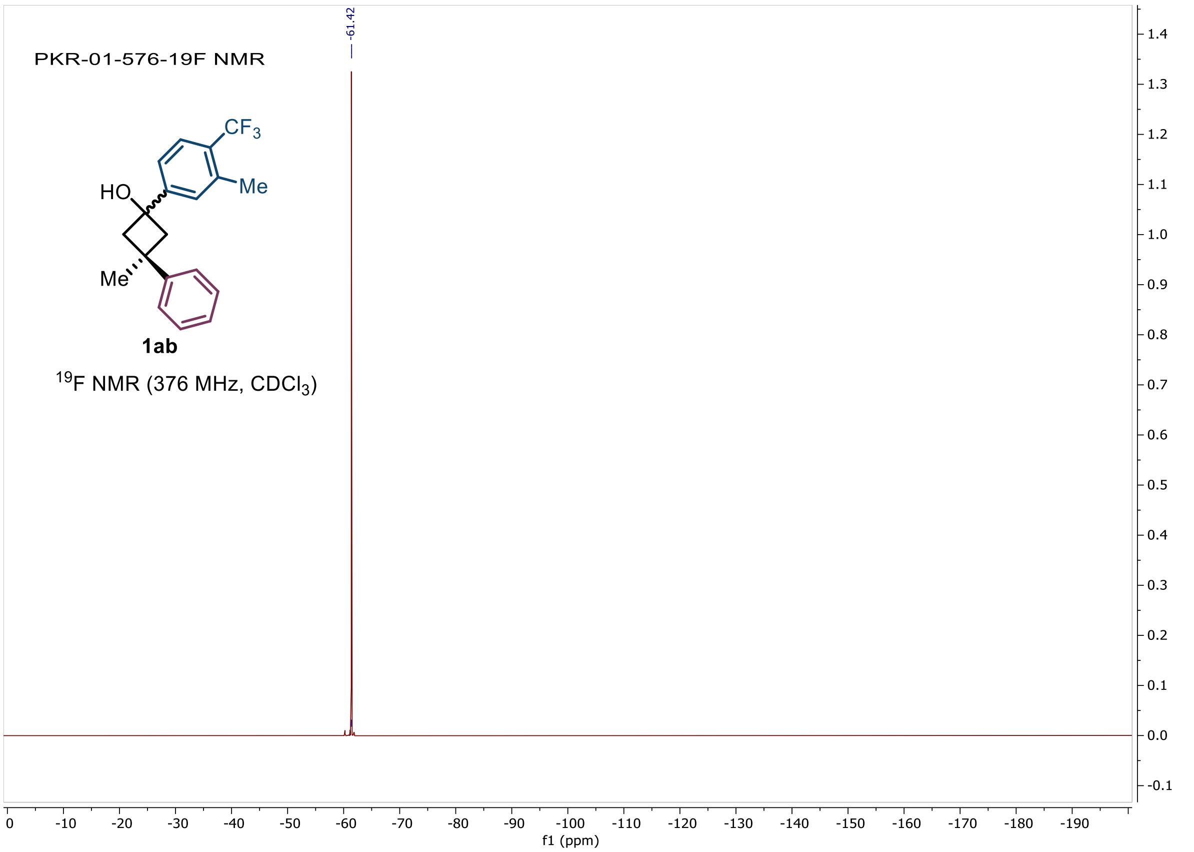
PKR-01-576-19F NMR
1ab
^{19}F NMR (376 MHz, CDCl$_3$)
-61.42
f1 (ppm)

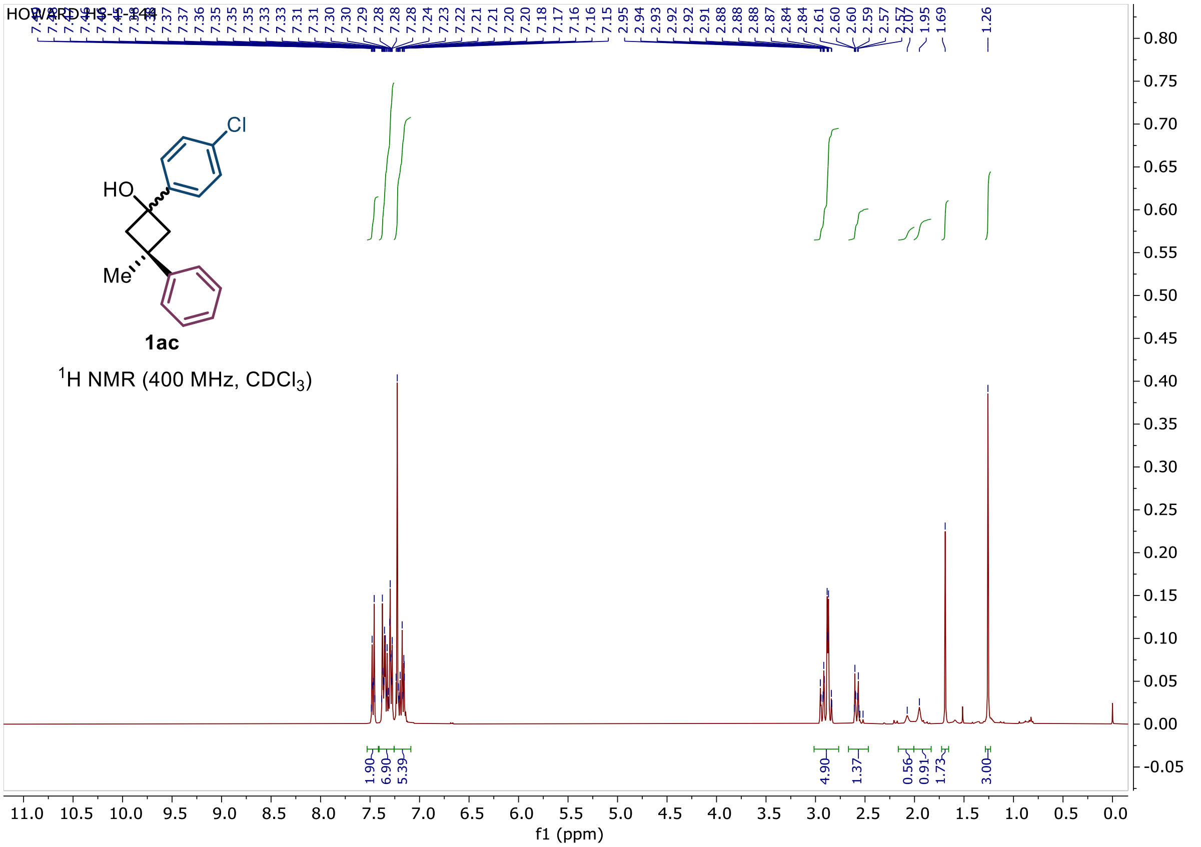
HOWARD HS-1-144
HO
Me
Cl
1ac
^{1}H NMR (400 MHz, CDCl$_3$)
f1 (ppm)

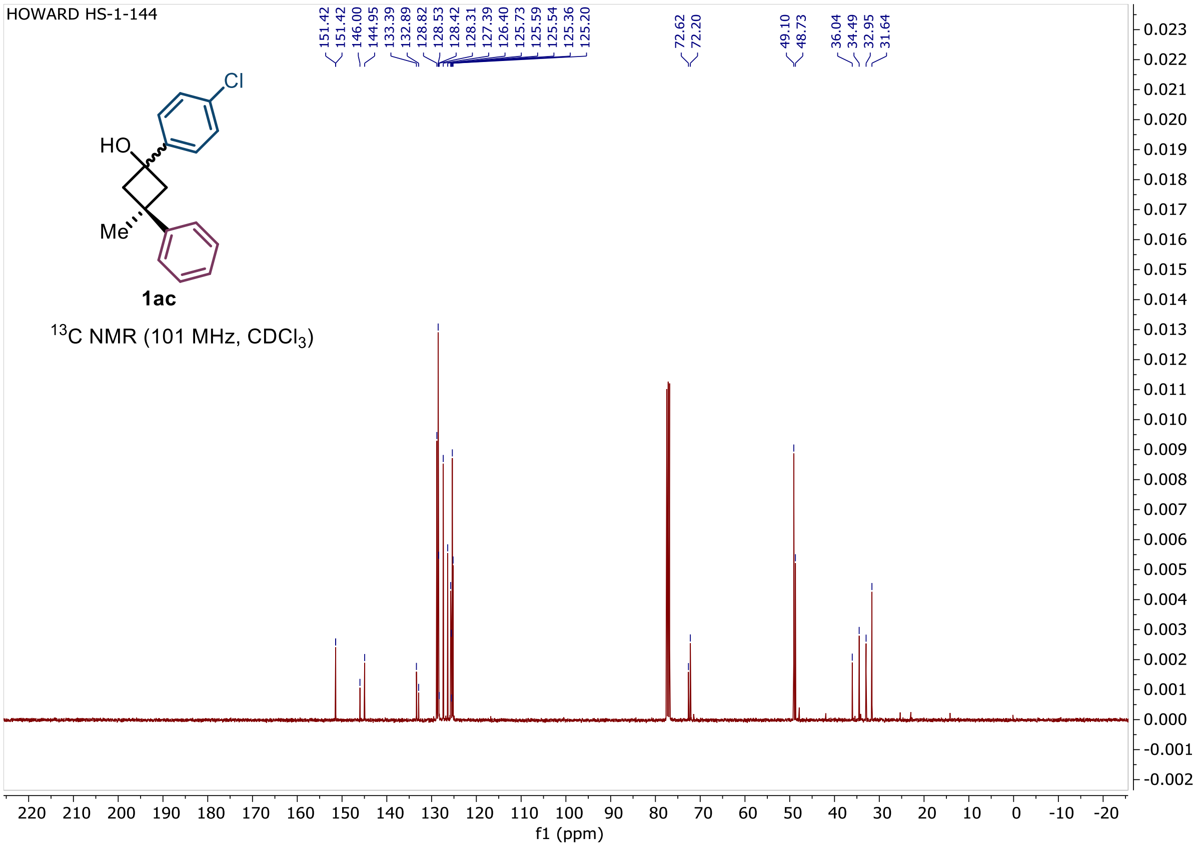
HOWARD HS-1-144
151.42
151.42
146.00
144.95
133.39
132.89
128.82
128.53
128.42
128.31
127.39
126.40
125.73
125.59
125.54
125.36
125.20
72.62
72.20
49.10
48.73
36.04
34.49
32.95
31.64
HO
Me
Cl
1ac
^{13}C NMR (101 MHz, CDCl$_3$)
f1 (ppm)

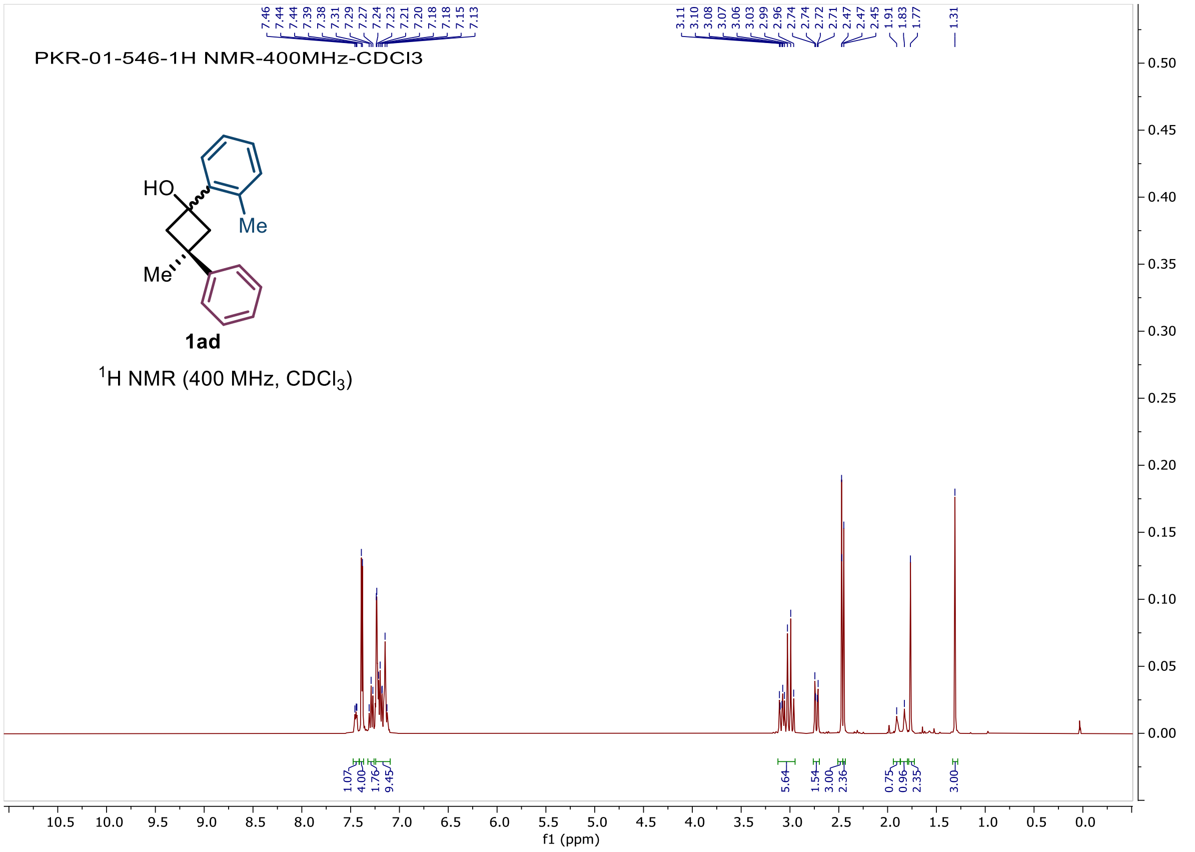
PKR-01-546-1H NMR-400MHz-CDCl3
HO
Me
Me
1ad
^{1}H NMR (400 MHz, CDCl$_3$)
f1 (ppm)

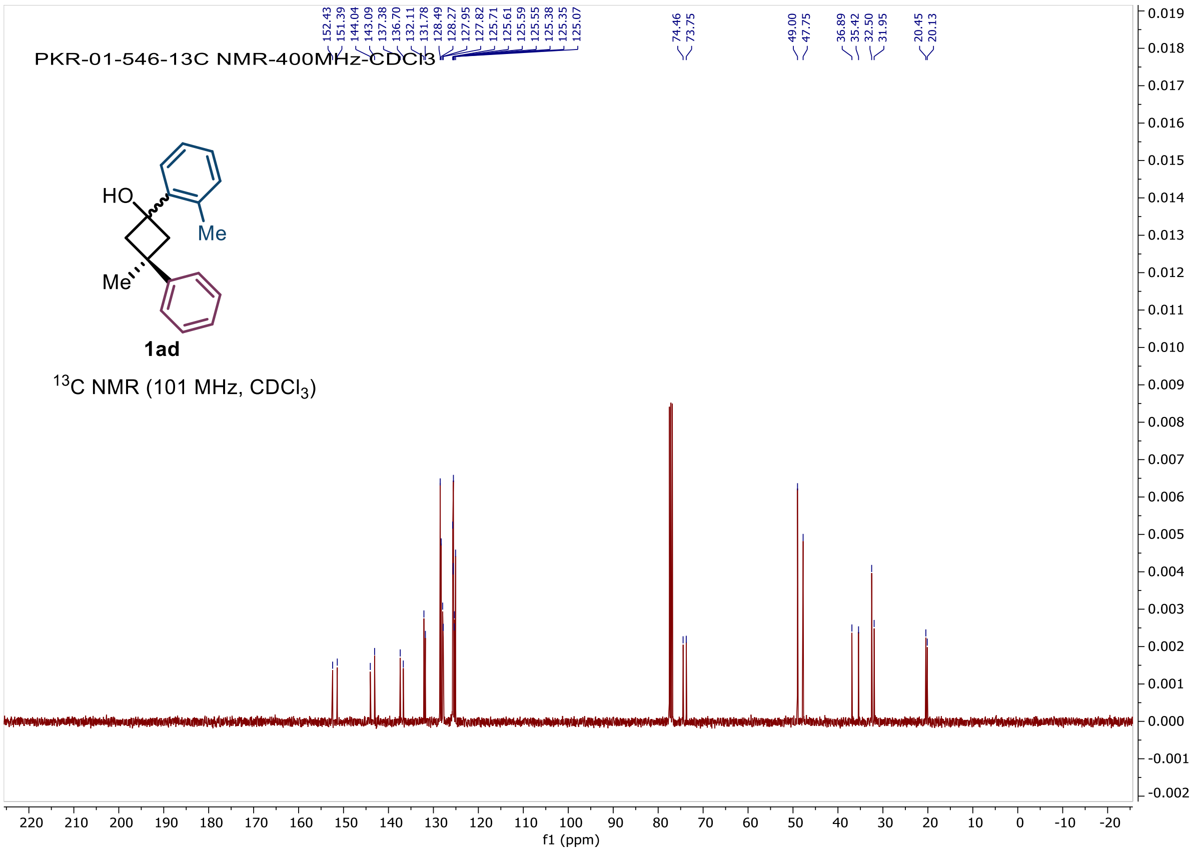
PKR-01-546-13C NMR-400MHz-CDCl3
HO
Me
Me
1ad
^{13}C NMR (101 MHz, CDCl$_3$)
f1 (ppm)

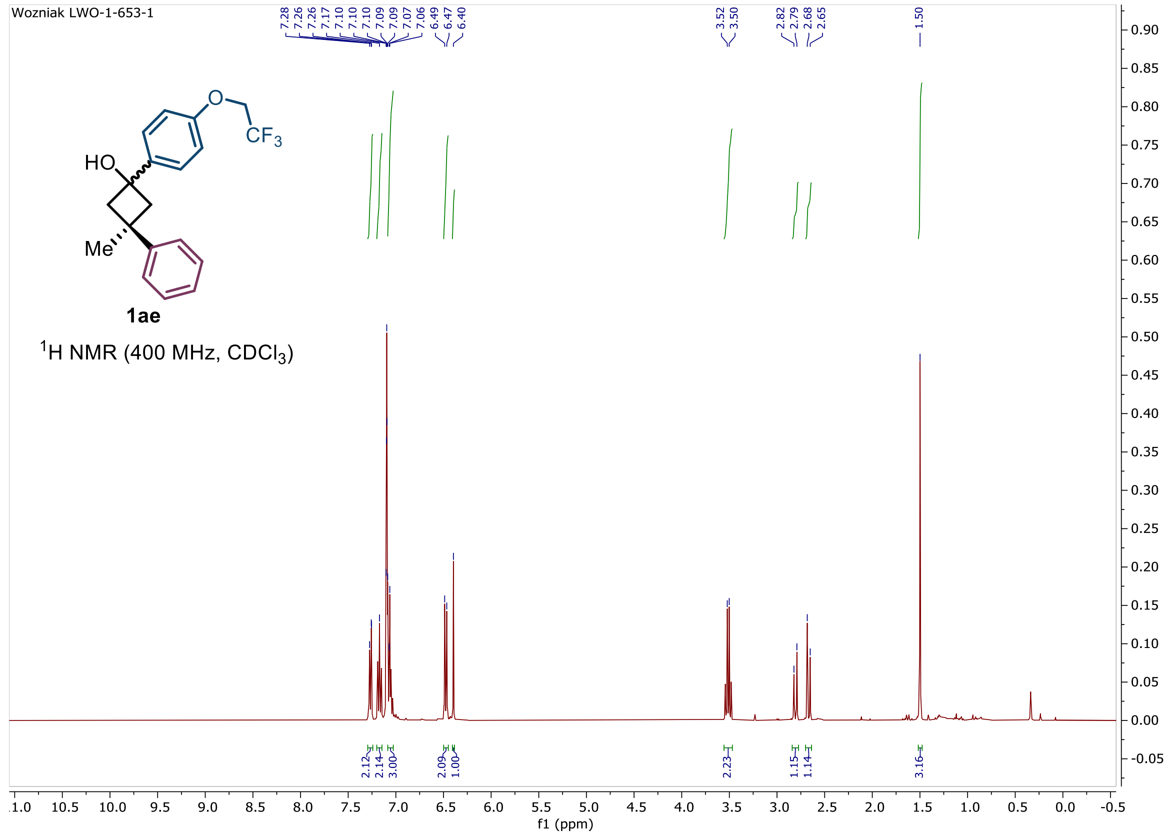
Wozniak LWO-1-653-1
7.28
7.26
7.26
7.17
7.10
7.10
7.09
7.09
7.07
7.06
6.49
6.47
6.40
3.52
3.50
2.82
2.79
2.68
2.65
1.50
HO
O
CF3
Me
1ae
1H NMR (400 MHz, CDCl3)
2.12
2.14
3.00
2.09
1.00
2.23
1.15
1.14
3.16
f1 (ppm)

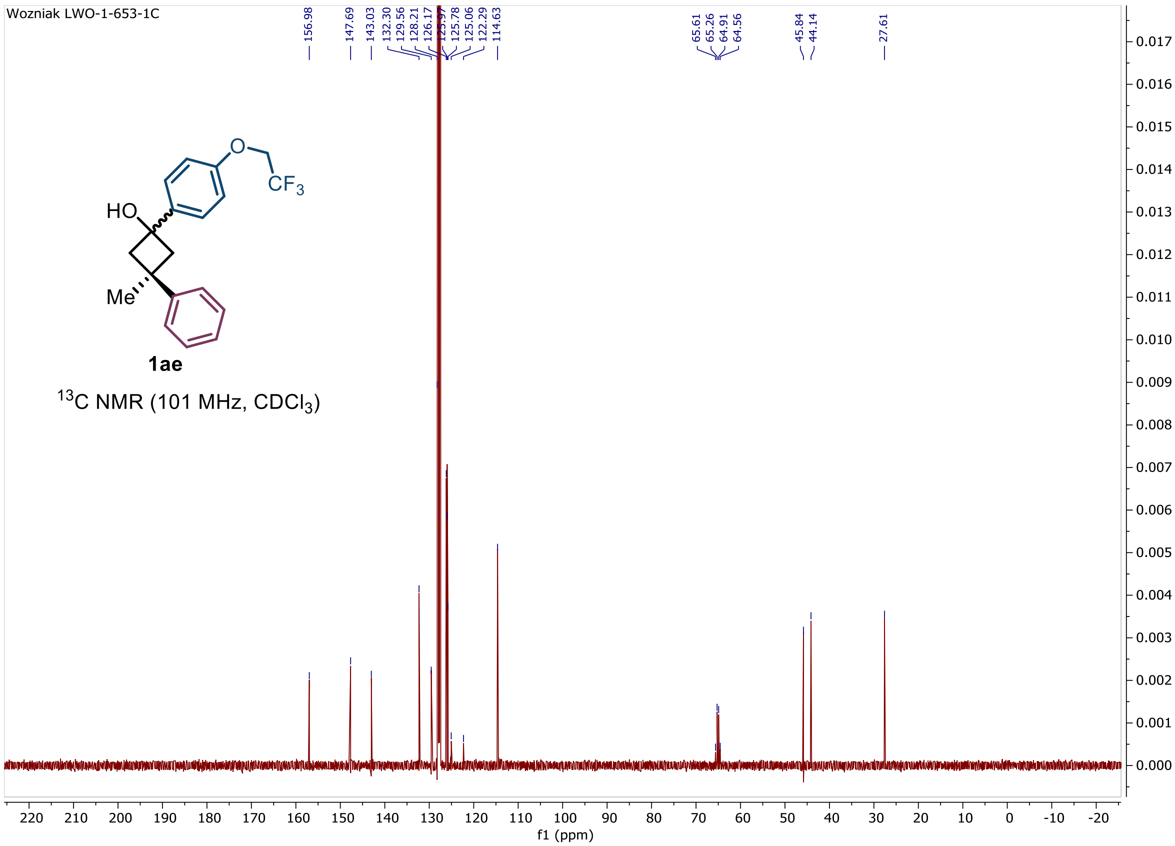
Wozniak LWO-1-653-1C
156.98
147.69
143.03
132.30
129.56
128.21
126.17
125.97
125.78
125.06
122.29
114.63
65.61
65.26
64.91
64.56
45.84
44.14
27.61
HO
O
CF3
Me
1ae
13C NMR (101 MHz, CDCl3)
f1 (ppm)

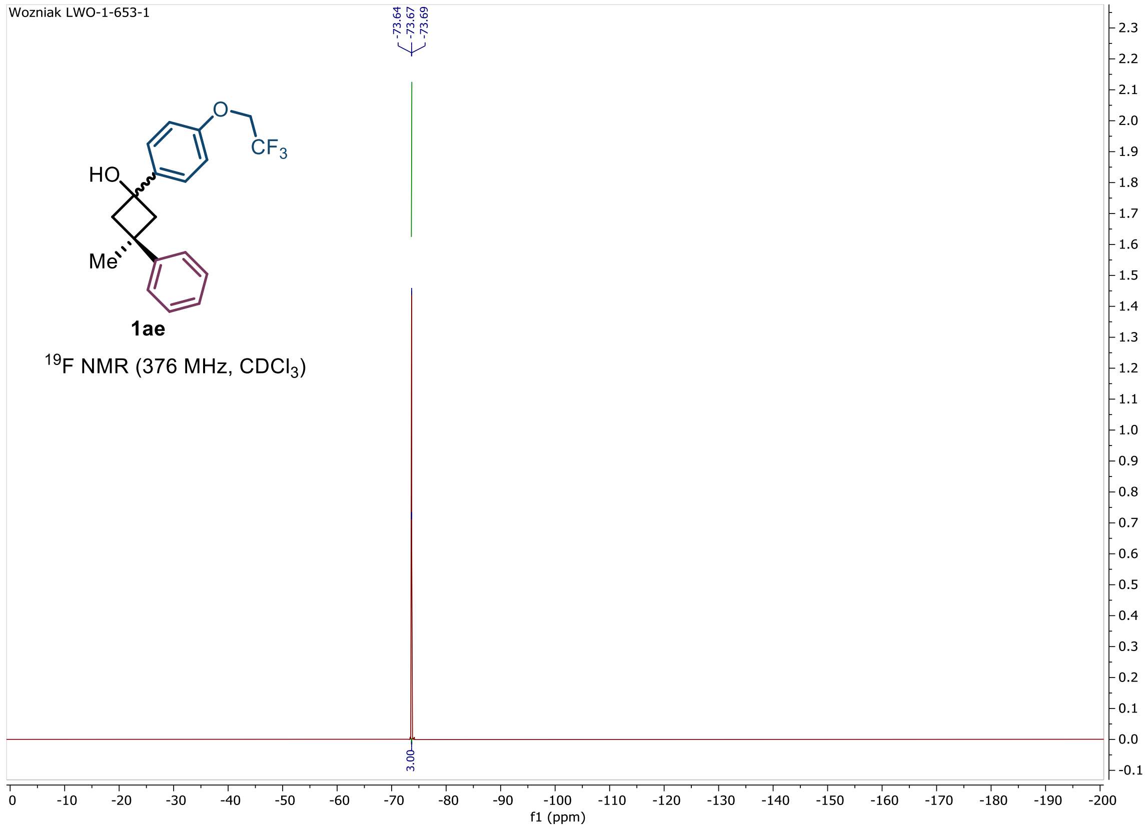

Wozniak LWO-1-653-1
-73.64
-73.67
-73.69
HO
O
CF3
Me
1ae
19F NMR (376 MHz, CDCl3)
3.00
f1 (ppm)

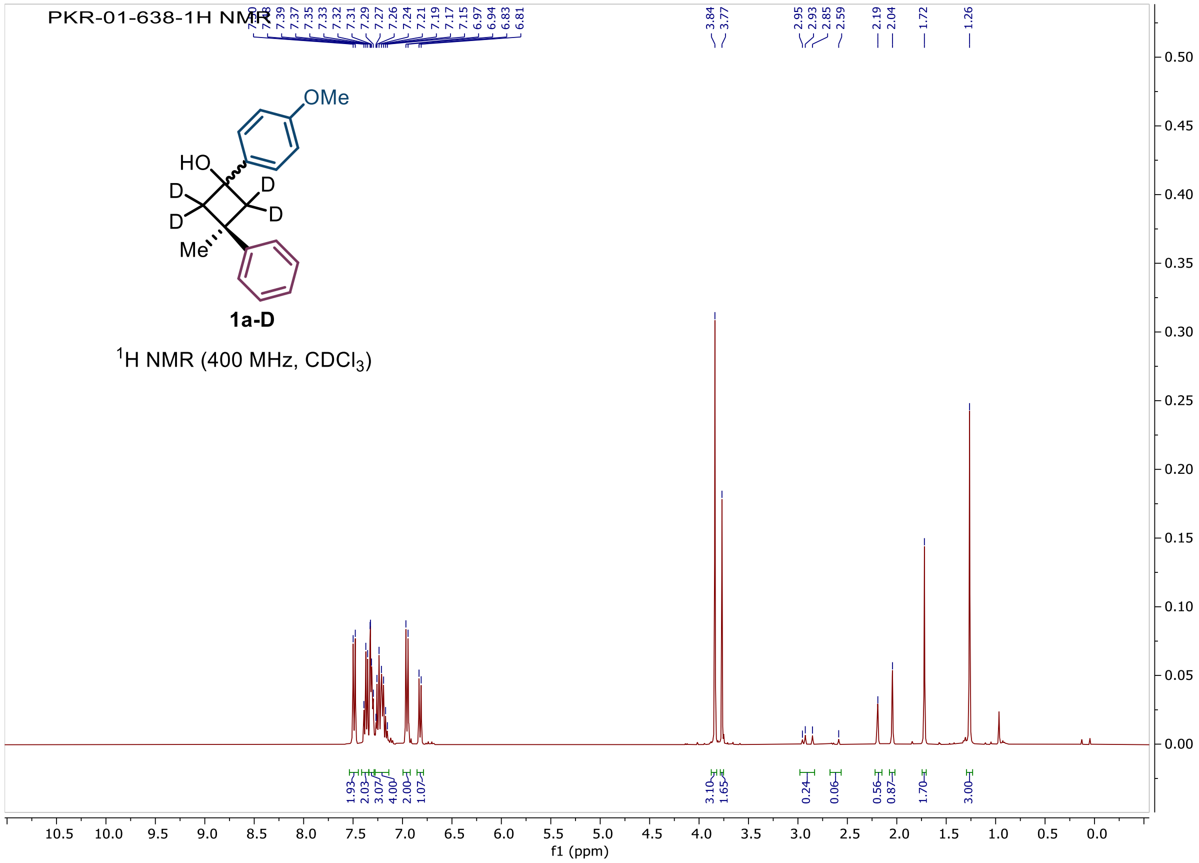

PKR-01-638-1H NMR
7.39
7.37
7.35
7.33
7.32
7.31
7.29
7.27
7.26
7.24
7.21
7.19
7.17
7.15
6.97
6.94
6.83
6.81
3.84
3.77
2.95
2.93
2.85
2.59
2.19
2.04
1.72
1.26
OMe
HO
D
D
D
D
Me
1a-D
1H NMR (400 MHz, CDCl3)
1.93
2.03
3.07
4.00
2.00
1.07
3.10
1.65
0.24
0.06
0.56
0.87
1.70
3.00
f1 (ppm)

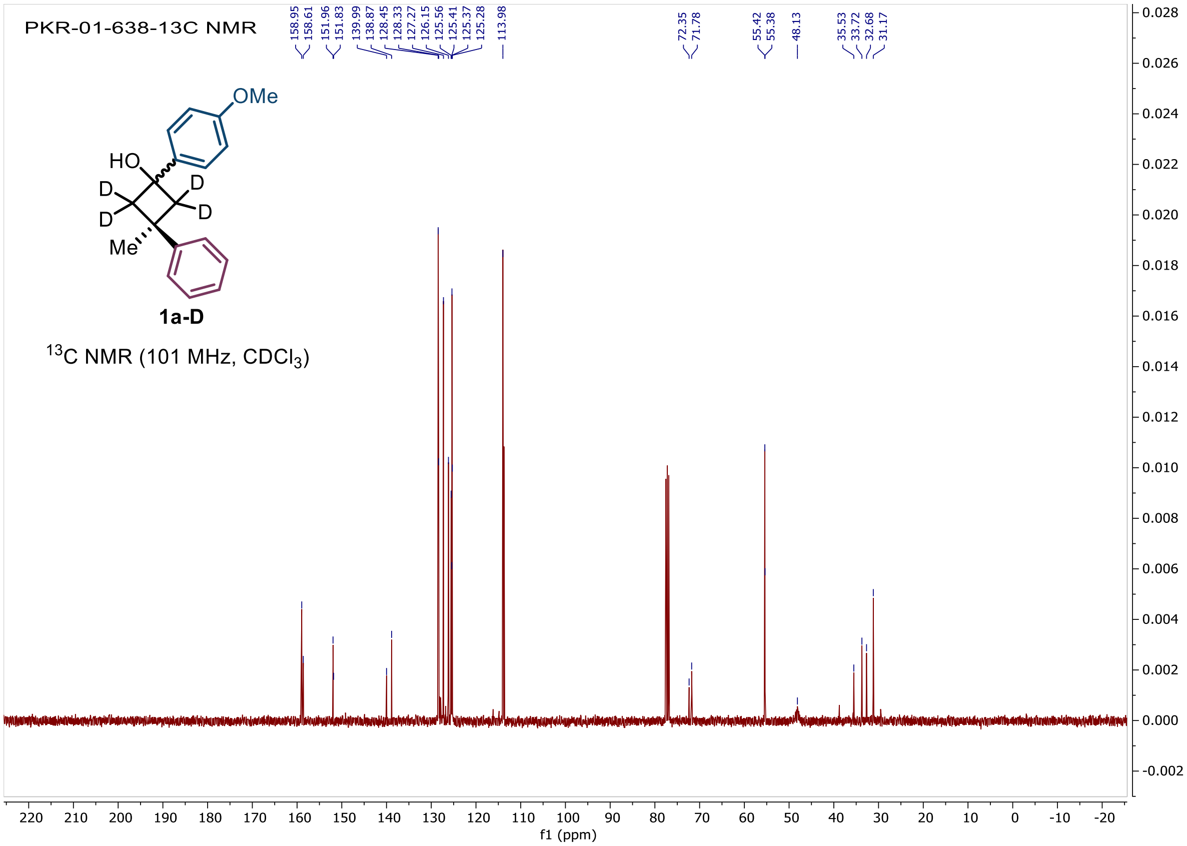
PKR-01-638-13C NMR
1a-D
^{13}C NMR (101 MHz, CDCl$_3$)
f1 (ppm)

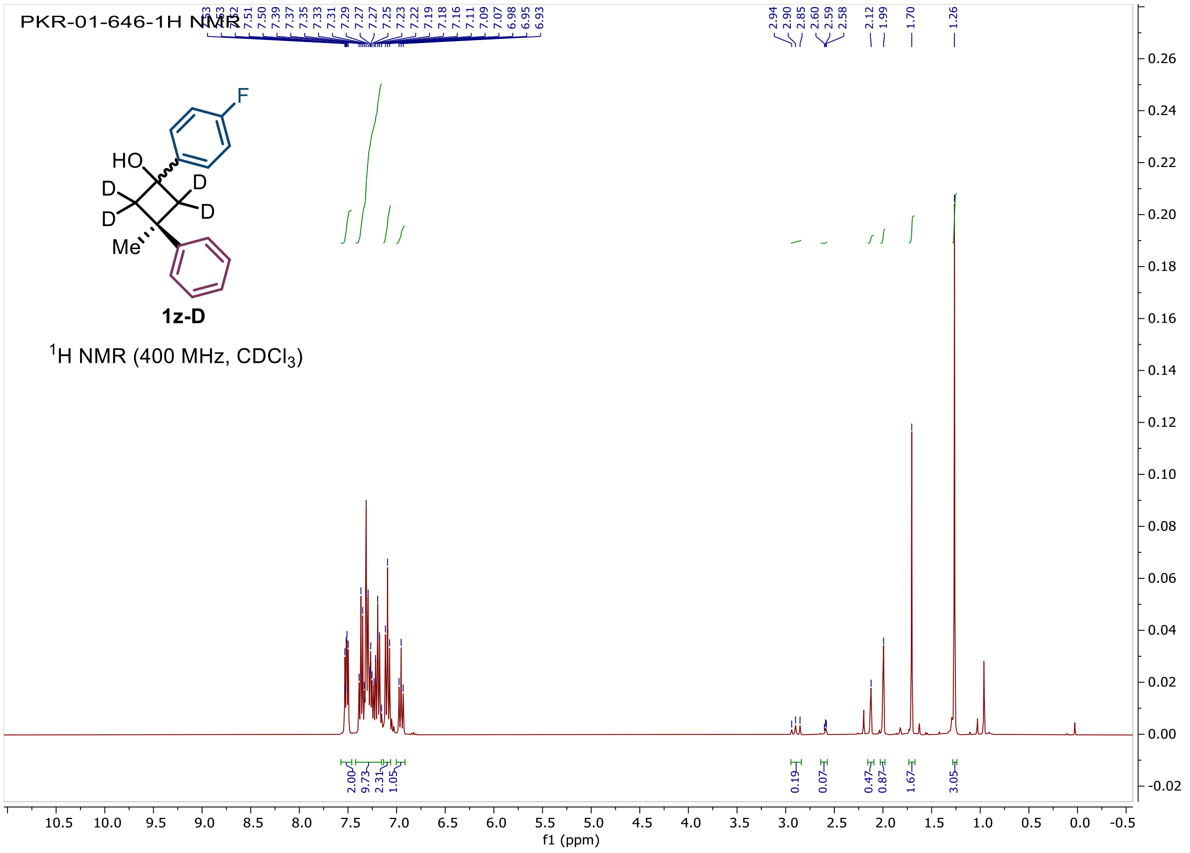
PKR-01-646-1H NMR
1z-D
^{1}H NMR (400 MHz, CDCl$_3$)
f1 (ppm)

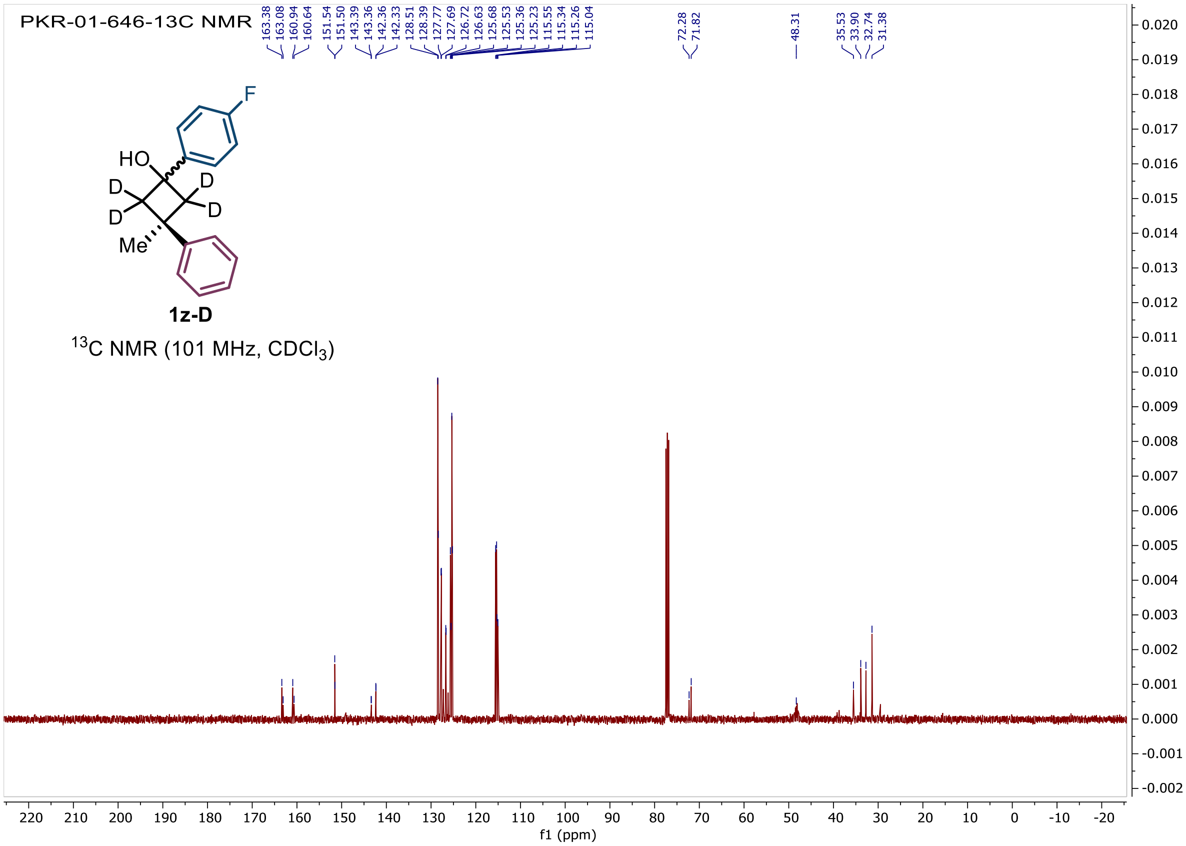
PKR-01-646-13C NMR
163.38
163.08
160.94
160.64
151.54
151.50
143.39
143.36
142.36
142.33
128.51
128.39
127.77
127.69
126.72
126.63
125.68
125.53
125.36
125.23
115.55
115.34
115.26
115.04
72.28
71.82
48.31
35.53
33.90
32.74
31.38
F
HO
D
D
D
D
Me
1z-D
^{13}C NMR (101 MHz, CDCl$_3$)
f1 (ppm)

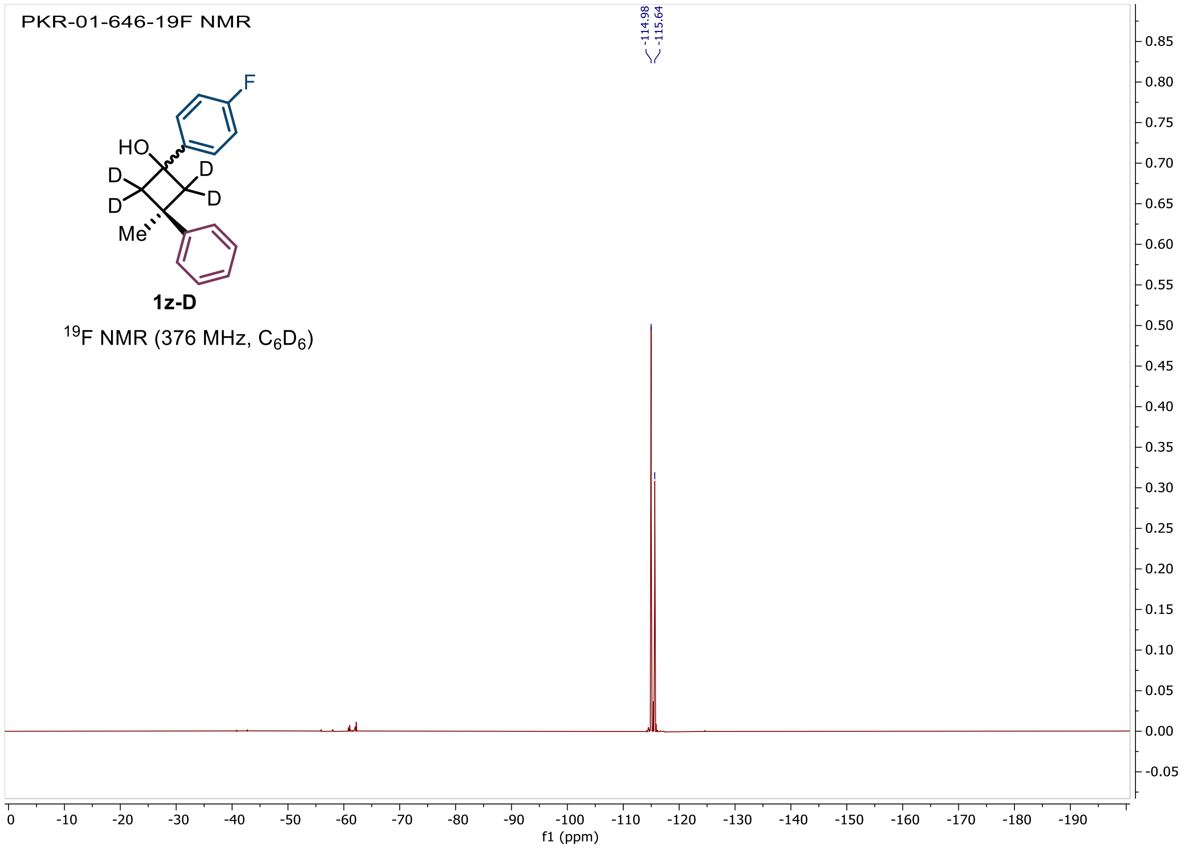
PKR-01-646-19F NMR
-114.98
-115.64
F
HO
D
D
D
D
Me
1z-D
^{19}F NMR (376 MHz, C$_6$D$_6$)
f1 (ppm)

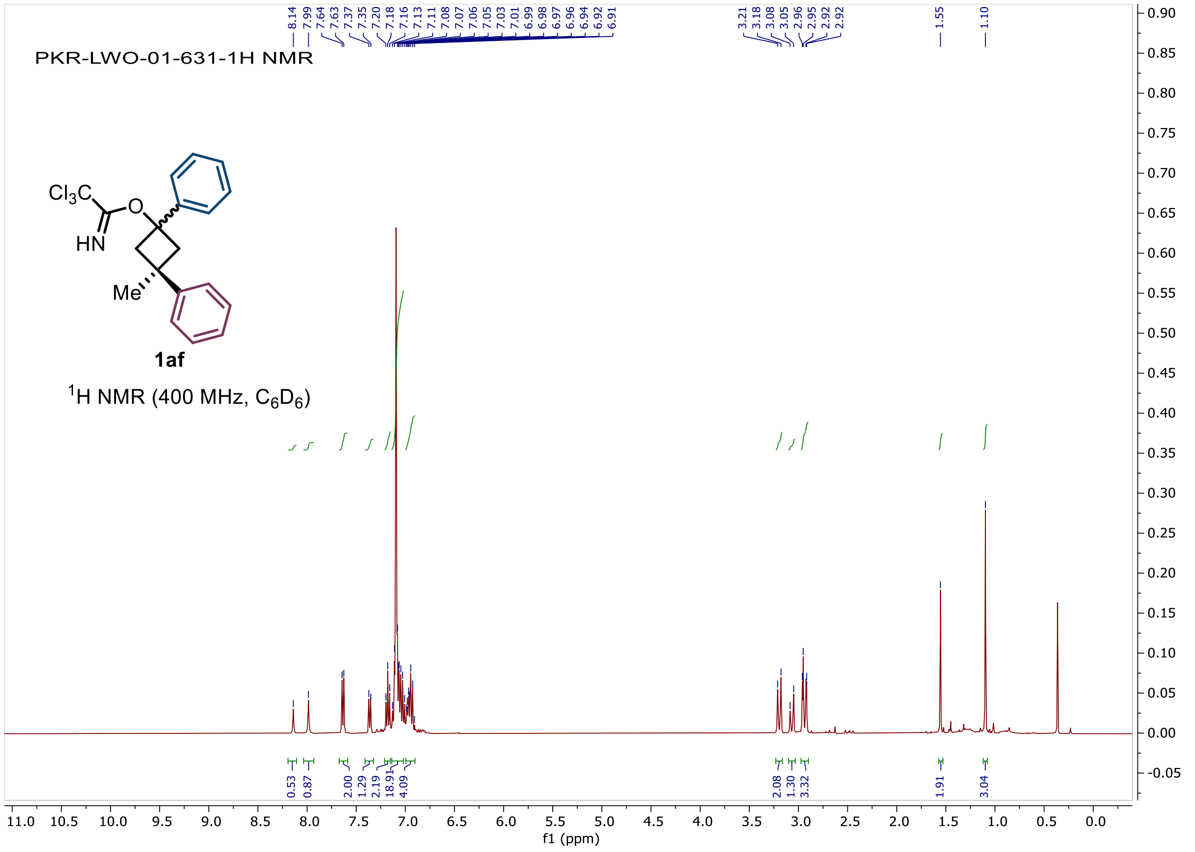
PKR-LWO-01-631-1H NMR
Cl3C
O
HN
Me
1af
1H NMR (400 MHz, C6D6)
8.14 7.99 7.64 7.63 7.37 7.35 7.20 7.18 7.16 7.13 7.11 7.08 7.07 7.06 7.05 7.03 7.01 6.99 6.98 6.97 6.96 6.94 6.92 6.91
3.21 3.18 3.08 3.05 2.96 2.95 2.92 2.92
1.55
1.10
0.53 0.87 2.00 1.29 2.19 18.91 4.09
2.08 1.30 3.32
1.91
3.04
f1 (ppm)

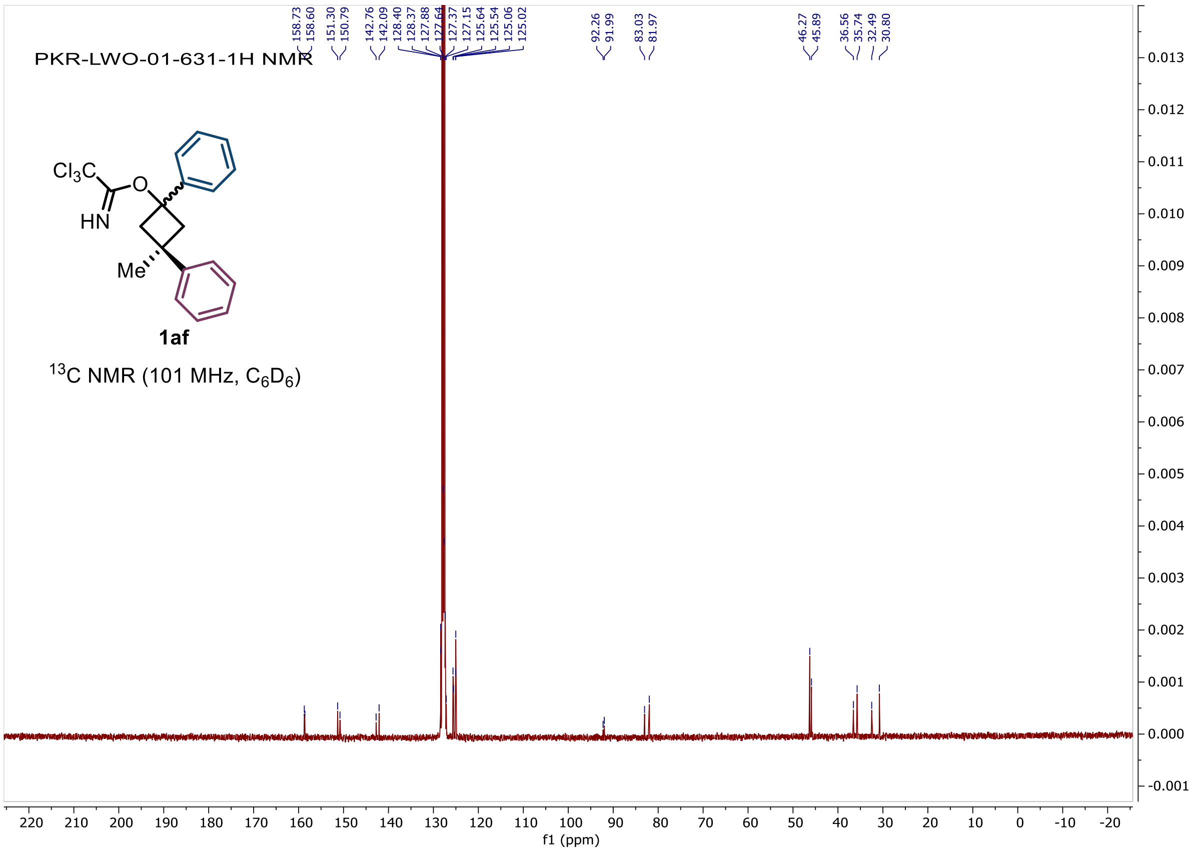
PKR-LWO-01-631-1H NMR
Cl3C
O
HN
Me
1af
13C NMR (101 MHz, C6D6)
158.73 158.60 151.30 150.79 142.76 142.09 128.40 128.37 127.88 127.64 127.37 127.15 125.64 125.54 125.06 125.02
92.26 91.99 83.03 81.97
46.27 45.89 36.56 35.74 32.49 30.80
f1 (ppm)

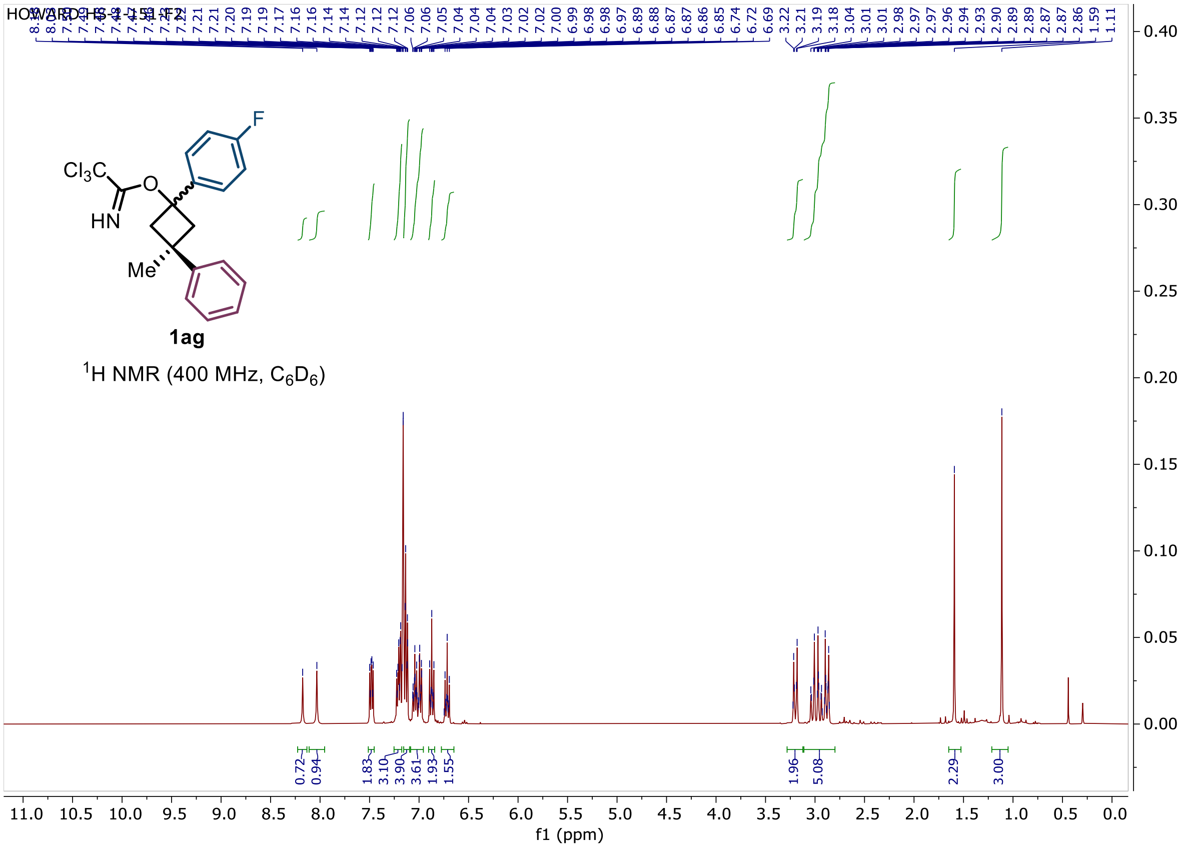
HOWARD HS-1-151-F2
1ag
^{1}H NMR (400 MHz, C_6D_6)
f1 (ppm)

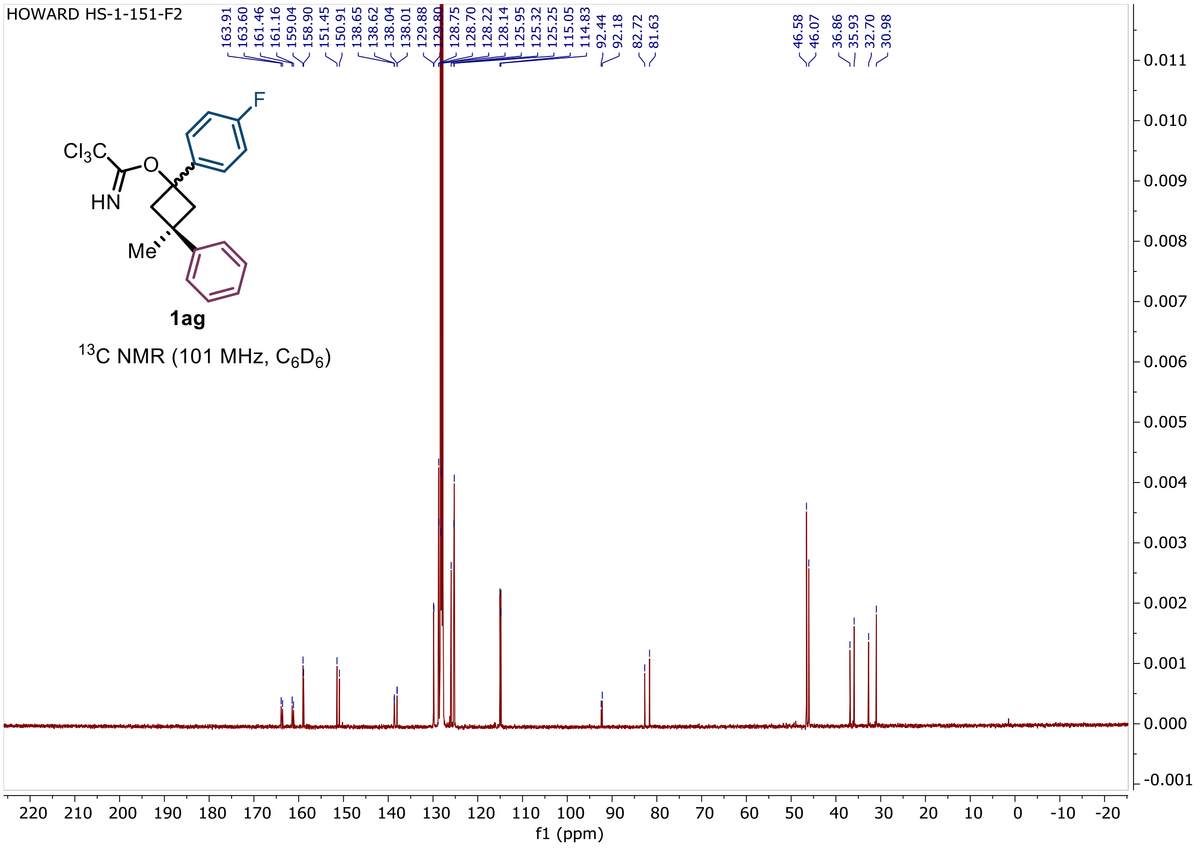
HOWARD HS-1-151-F2
1ag
^{13}C NMR (101 MHz, C_6D_6)
f1 (ppm)

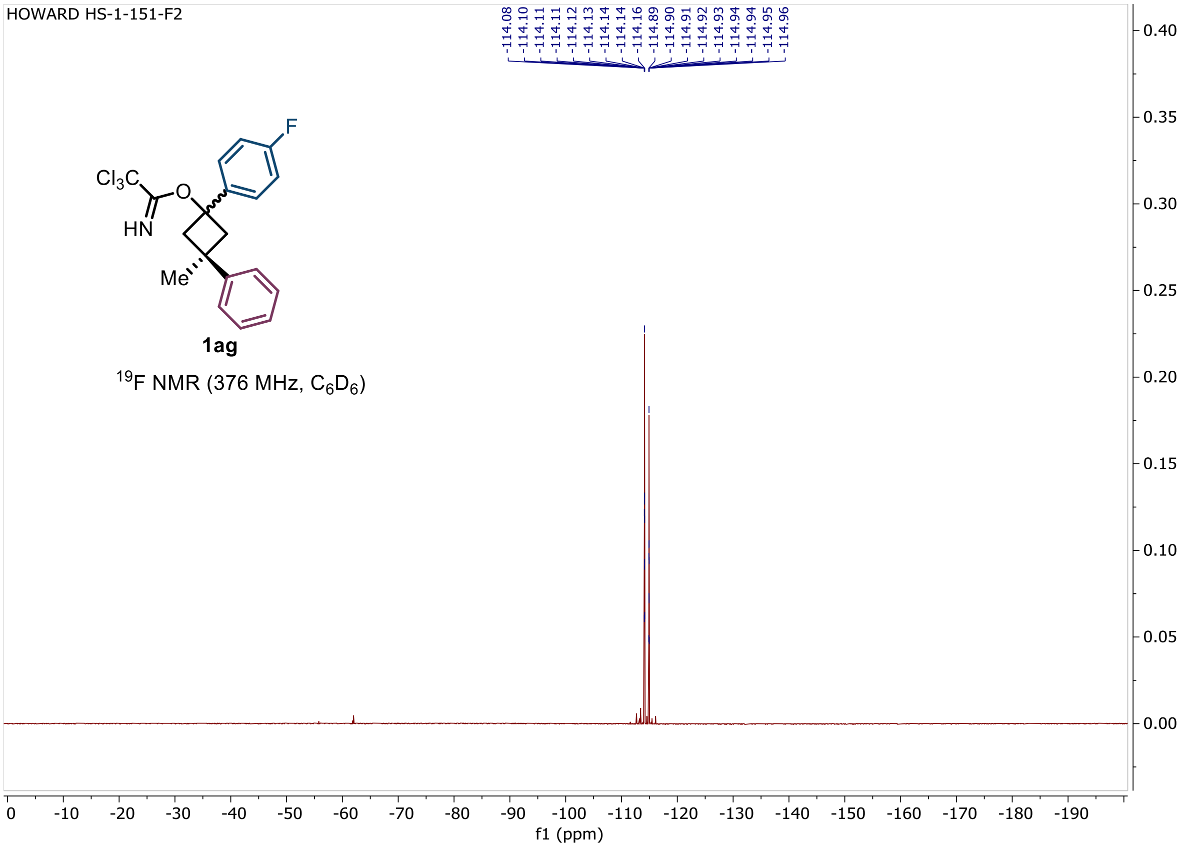
HOWARD HS-1-151-F2
-114.08
-114.10
-114.11
-114.11
-114.12
-114.13
-114.14
-114.14
-114.16
-114.89
-114.90
-114.91
-114.92
-114.93
-114.94
-114.94
-114.95
-114.96
Cl3C
O
F
HN
Me
1ag
19F NMR (376 MHz, C6D6)
f1 (ppm)

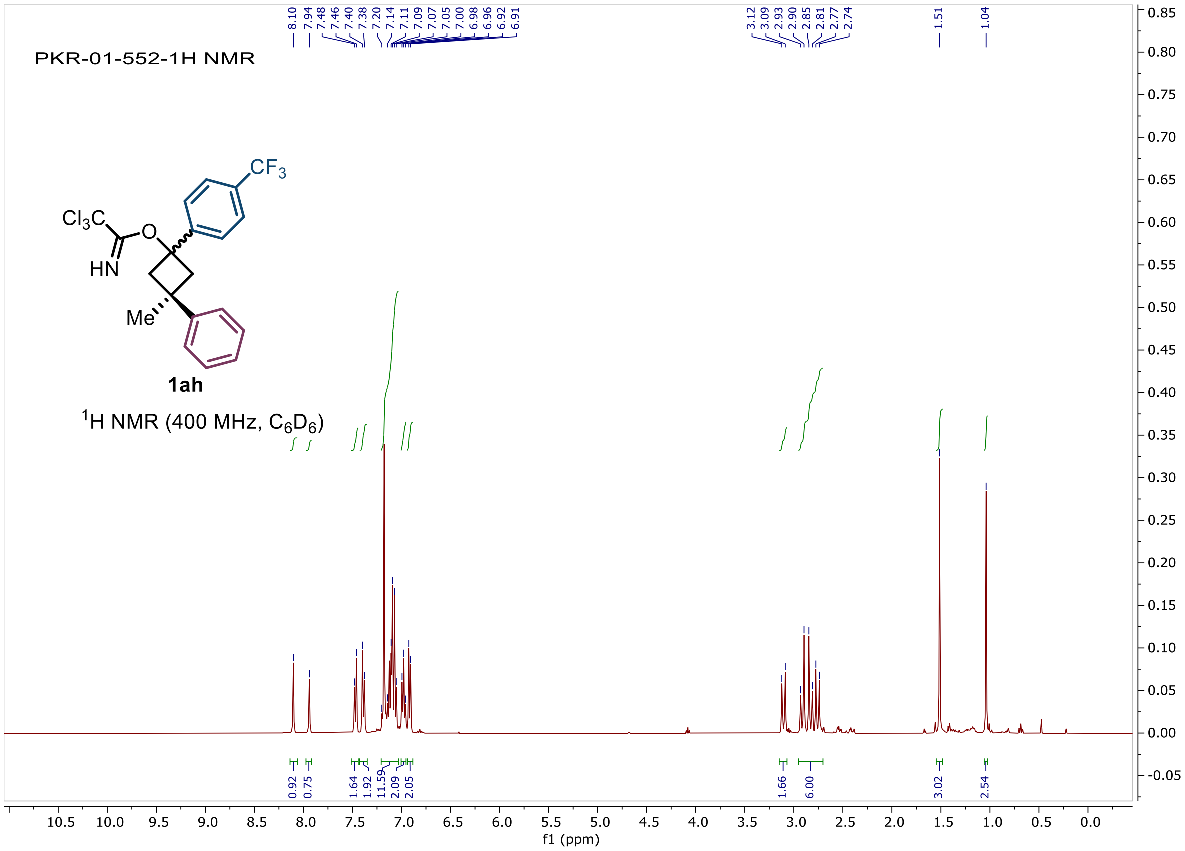
PKR-01-552-1H NMR
8.10
7.94
7.48
7.46
7.40
7.38
7.20
7.14
7.11
7.09
7.07
7.05
7.00
6.98
6.92
6.91
3.12
3.09
2.93
2.90
2.85
2.81
2.77
2.74
1.51
1.04
CF3
Cl3C
O
HN
Me
1ah
1H NMR (400 MHz, C6D6)
0.92
0.75
1.64
1.92
11.59
2.09
2.05
1.66
6.00
3.02
2.54
f1 (ppm)

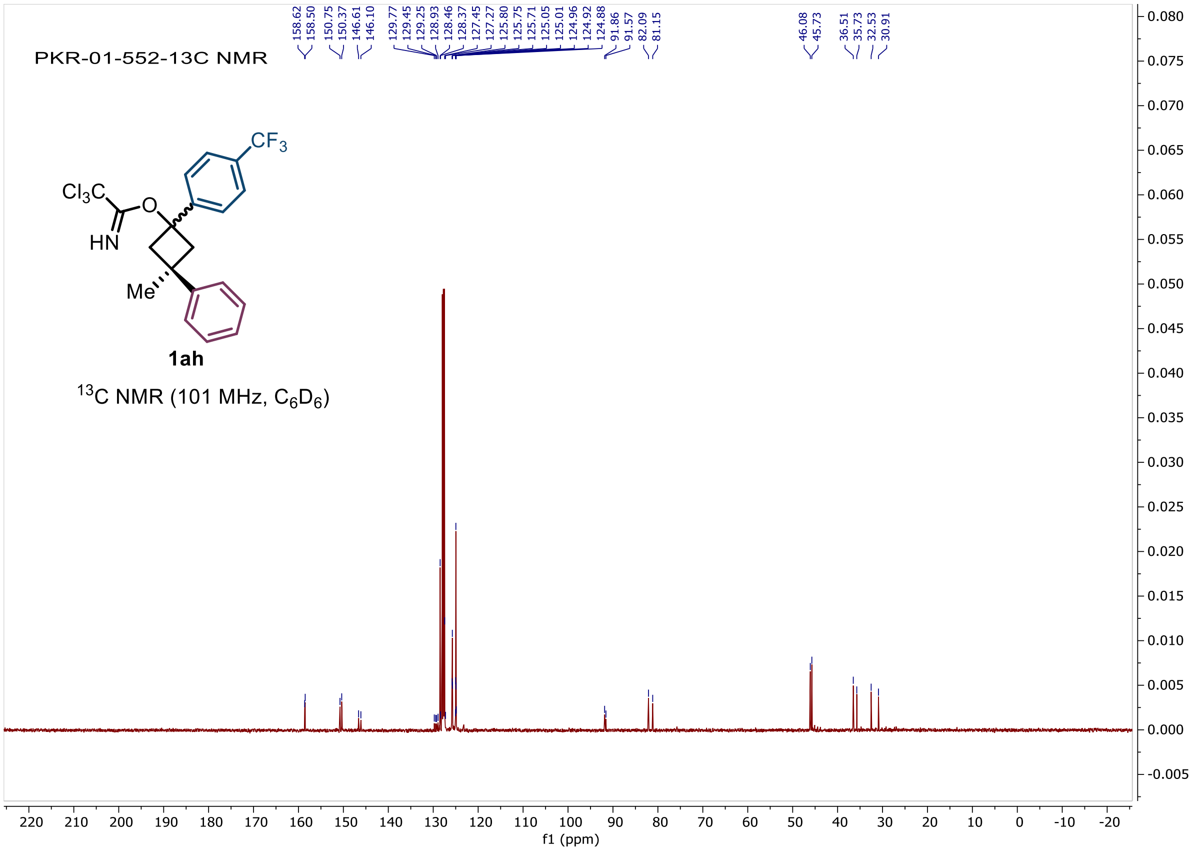

PKR-01-552-13C NMR
158.62
158.50
150.75
150.37
146.61
146.10
129.77
129.45
129.25
128.93
128.46
128.37
127.45
127.27
125.80
125.75
125.71
125.05
125.01
124.96
124.92
124.88
91.86
91.57
82.09
81.15
46.08
45.73
36.51
35.73
32.53
30.91
CF3
Cl3C
O
HN
Me
1ah
13C NMR (101 MHz, C6D6)
f1 (ppm)

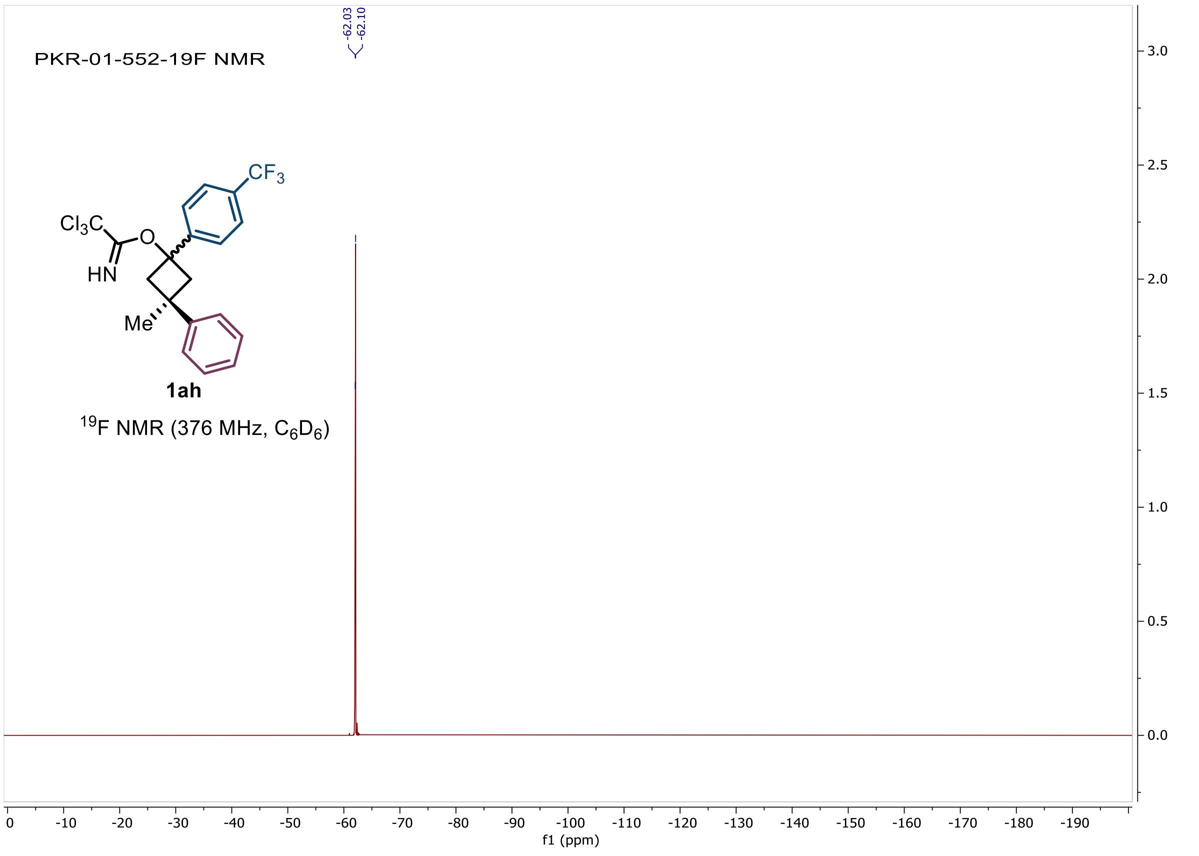

PKR-01-552-19F NMR
-62.03
-62.10
CF3
Cl3C
O
HN
Me
1ah
19F NMR (376 MHz, C6D6)
f1 (ppm)

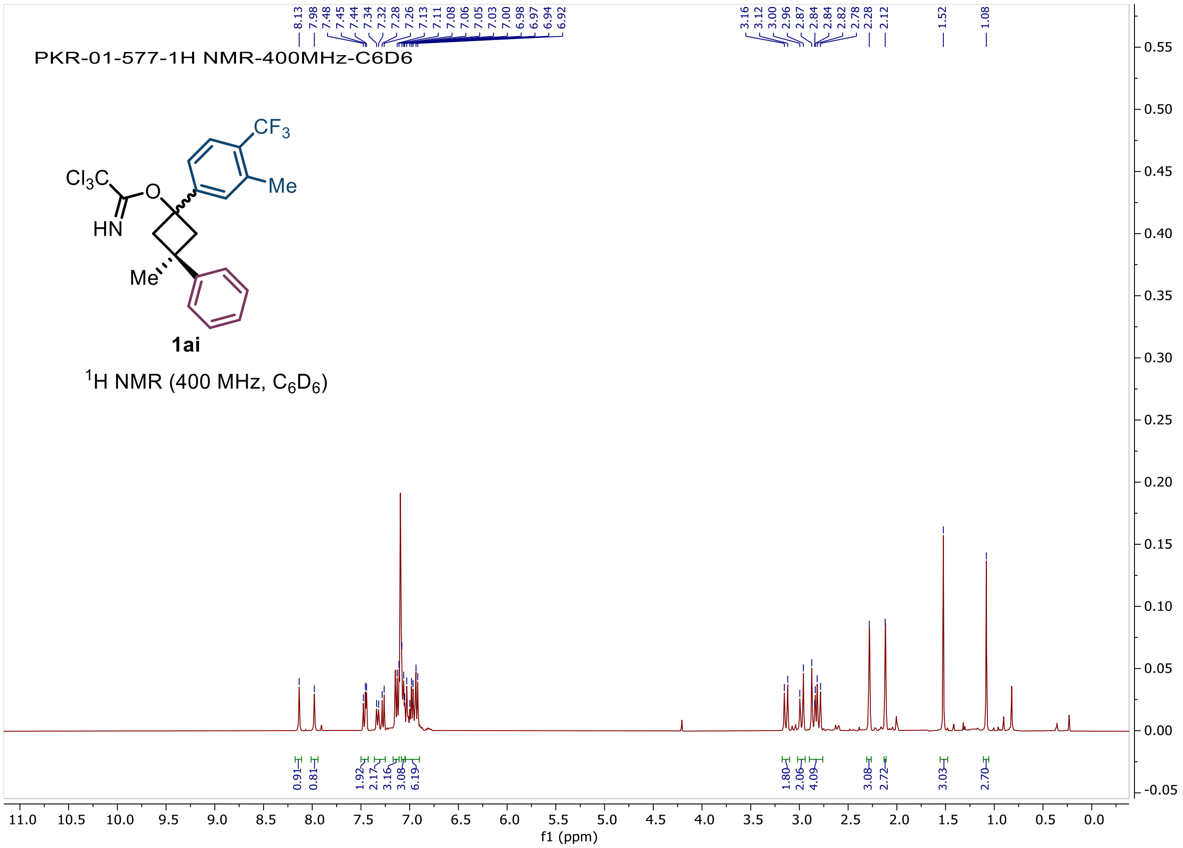
PKR-01-577-1H NMR-400MHz-C6D6
CF3
Me
Cl3C
O
HN
Me
1ai
1H NMR (400 MHz, C6D6)
f1 (ppm)

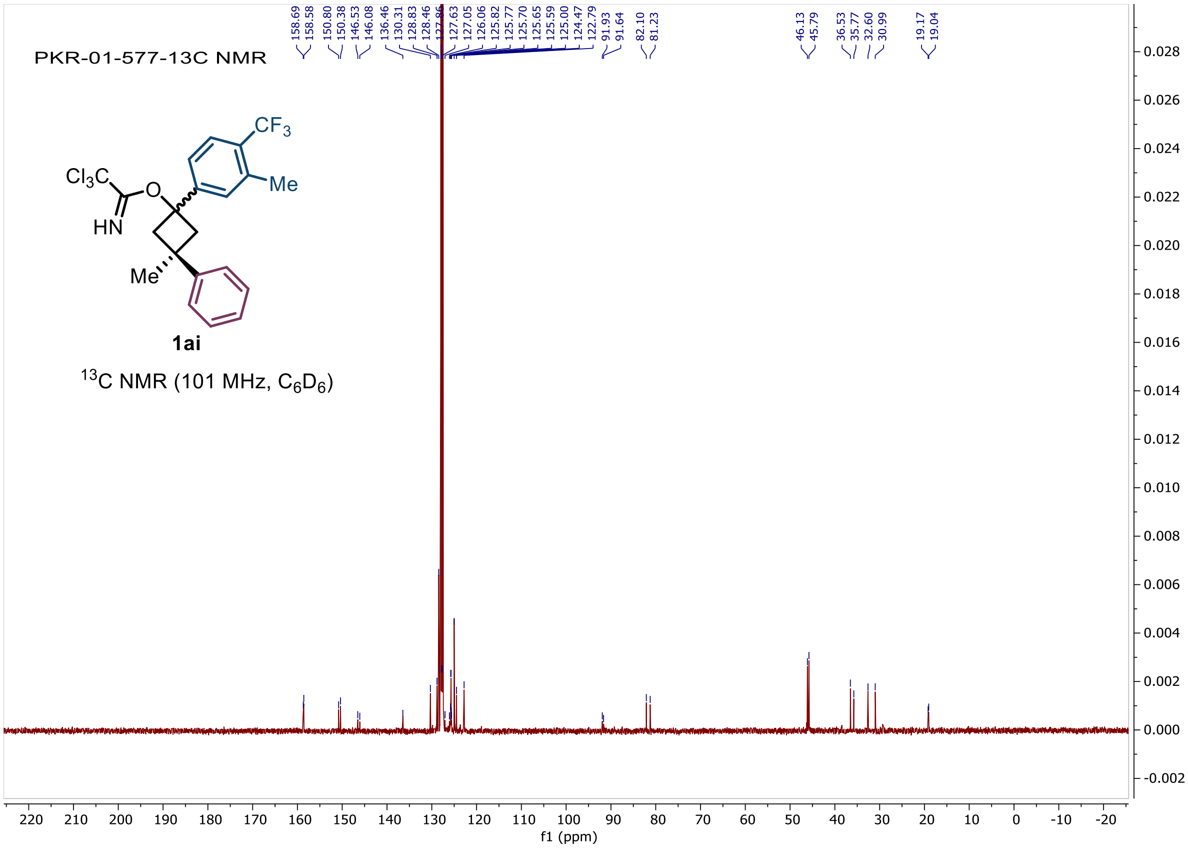
PKR-01-577-13C NMR
CF3
Me
Cl3C
O
HN
Me
1ai
13C NMR (101 MHz, C6D6)
f1 (ppm)

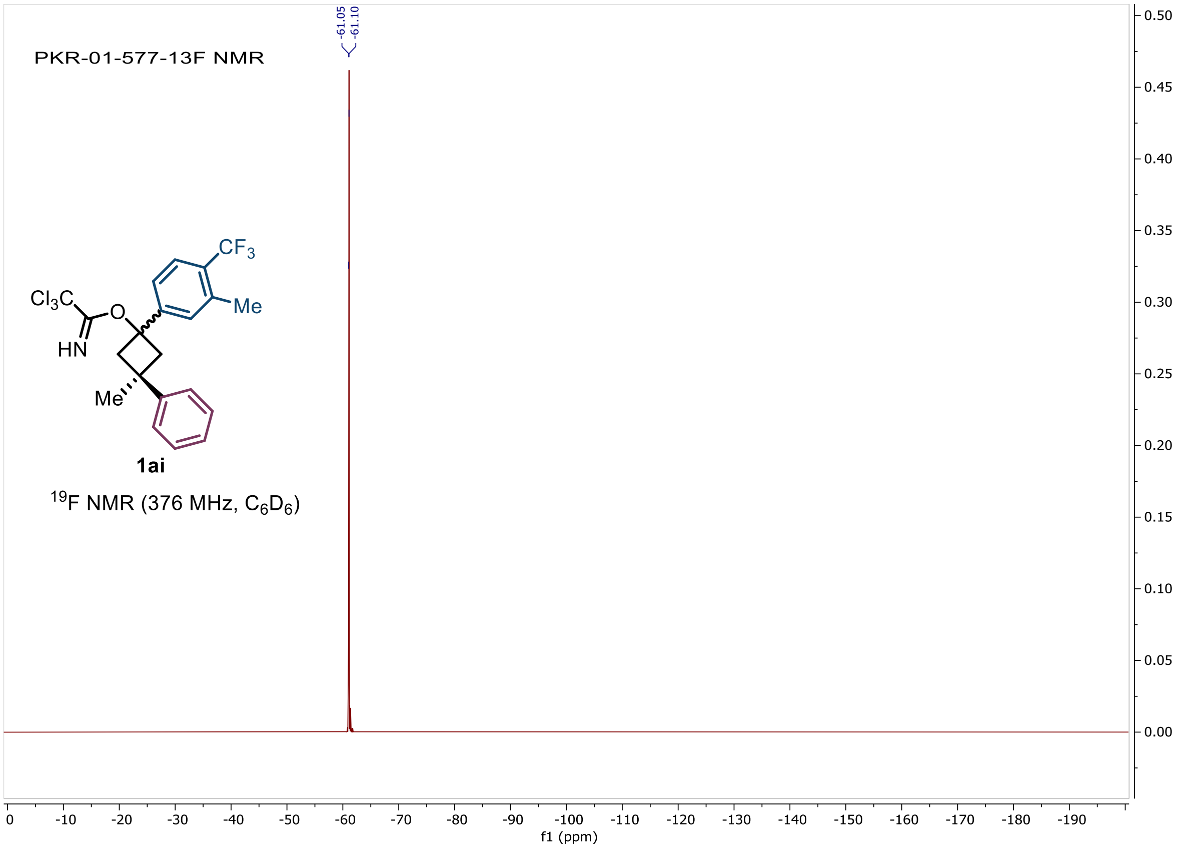
PKR-01-577-13F NMR
-61.05
-61.10
CF3
Me
Cl3C
O
HN
Me
1ai
19F NMR (376 MHz, C6D6)
f1 (ppm)

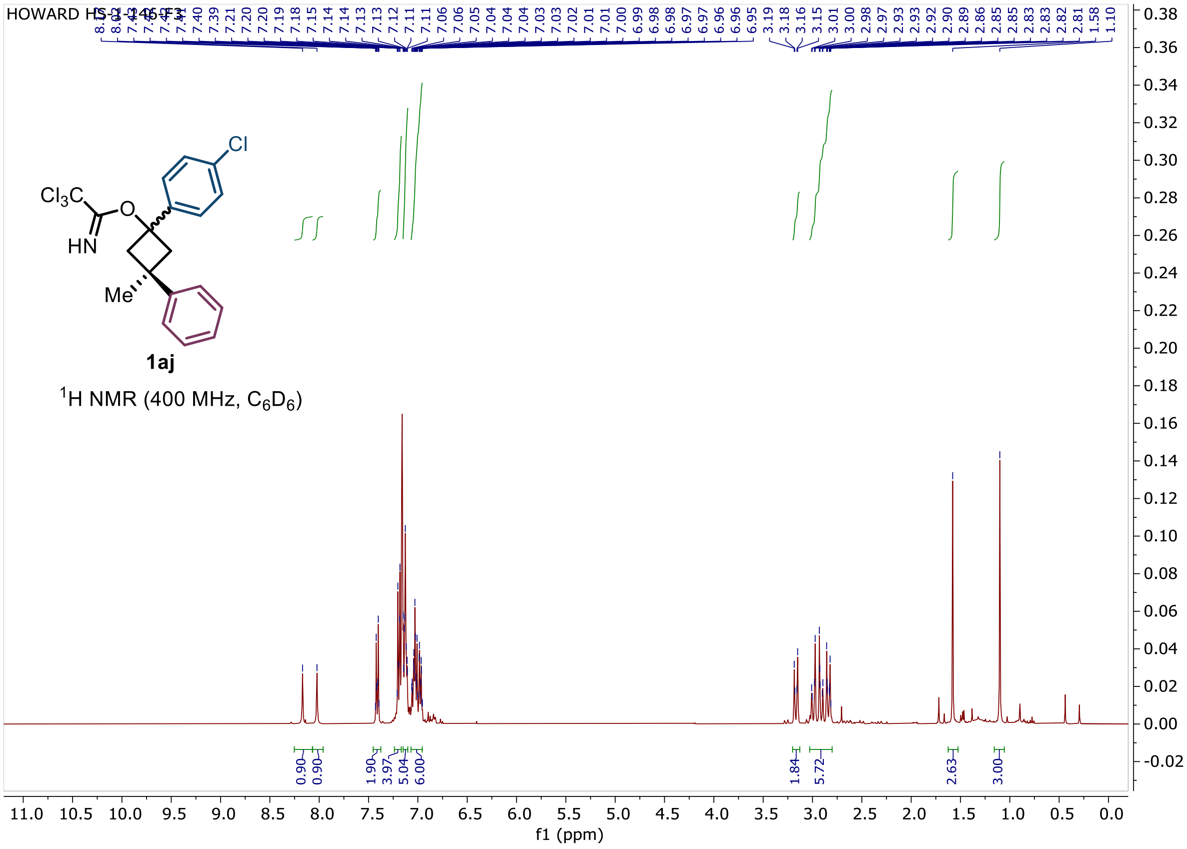
Cl
Cl3C
O
HN
Me
1aj
1H NMR (400 MHz, C6D6)
f1 (ppm)

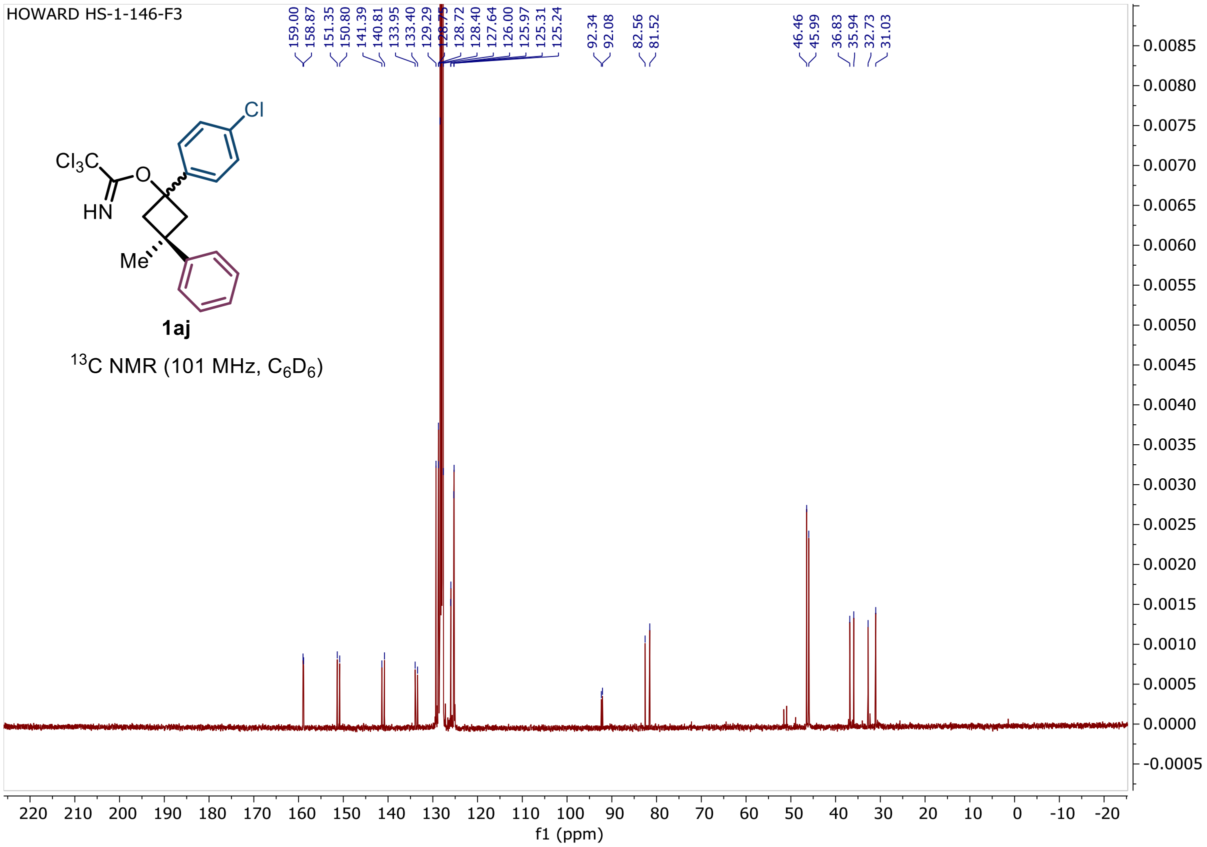
HOWARD HS-1-146-F3
159.00
158.87
151.35
150.80
141.39
140.81
133.95
133.40
129.29
128.72
128.40
127.64
126.00
125.97
125.31
125.24
92.34
92.08
82.56
81.52
46.46
45.99
36.83
35.94
32.73
31.03
Cl3C
O
HN
Cl
Me
1aj
13C NMR (101 MHz, C6D6)
f1 (ppm)

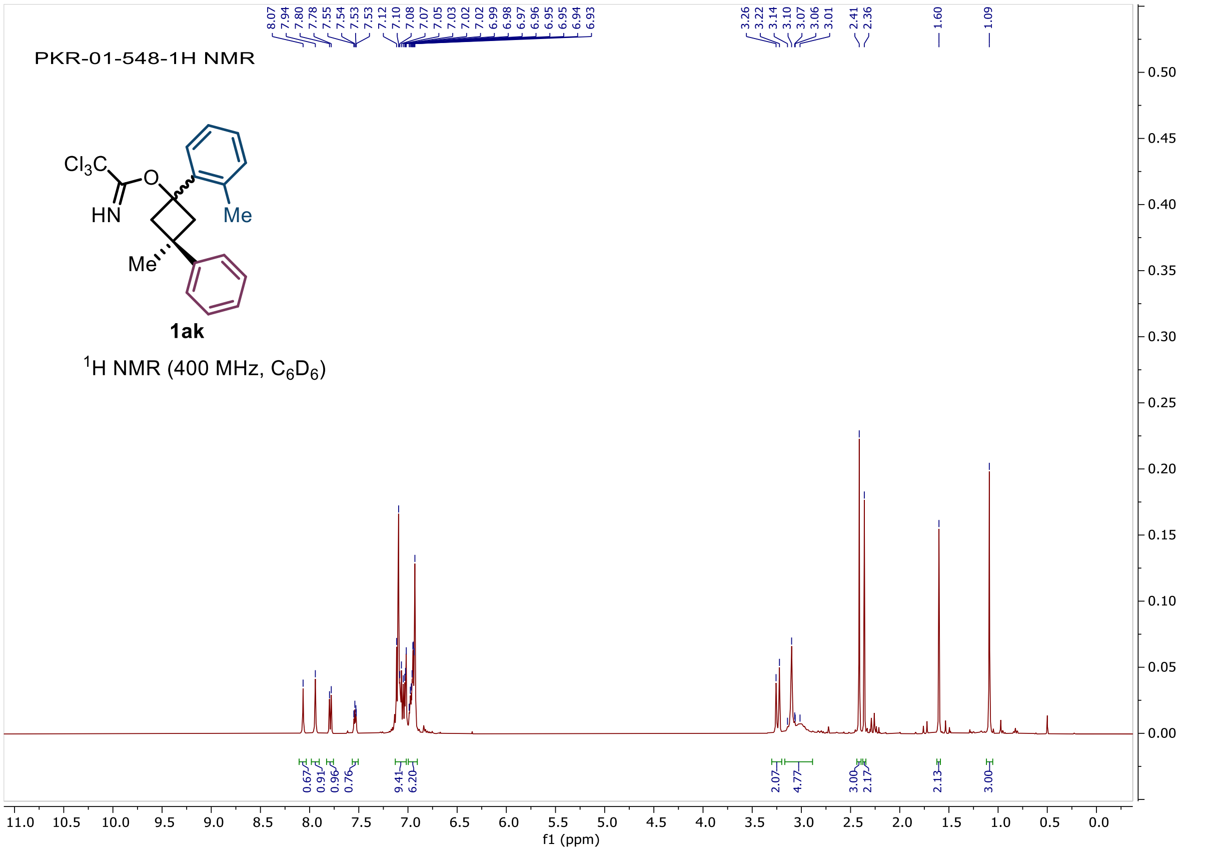
PKR-01-548-1H NMR
8.07
7.94
7.80
7.78
7.55
7.54
7.53
7.53
7.12
7.10
7.08
7.07
7.05
7.03
7.02
7.02
6.99
6.98
6.97
6.96
6.95
6.95
6.94
6.93
3.26
3.22
3.14
3.10
3.07
3.06
3.01
2.41
2.36
1.60
1.09
Cl3C
O
HN
Me
Me
1ak
1H NMR (400 MHz, C6D6)
0.67
0.91
0.96
0.76
9.41
6.20
2.07
4.77
3.00
2.17
2.13
3.00
f1 (ppm)

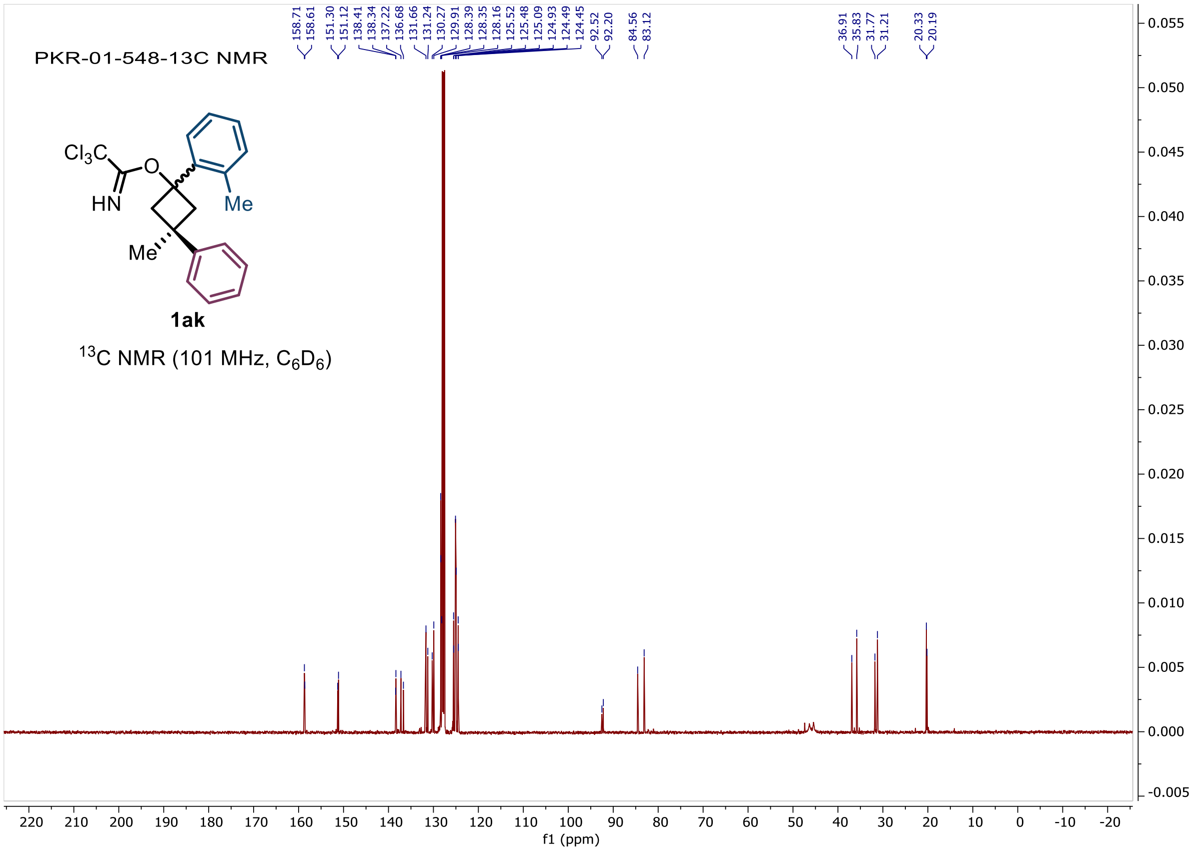
PKR-01-548-13C NMR
158.71
158.61
151.30
151.12
138.41
138.34
137.22
136.68
131.66
131.24
130.27
129.91
128.39
128.35
128.16
125.52
125.48
125.09
124.93
124.49
124.45
92.52
92.20
84.56
83.12
36.91
35.83
31.77
31.21
20.33
20.19
Cl3C
O
HN
Me
Me
1ak
13C NMR (101 MHz, C6D6)
f1 (ppm)

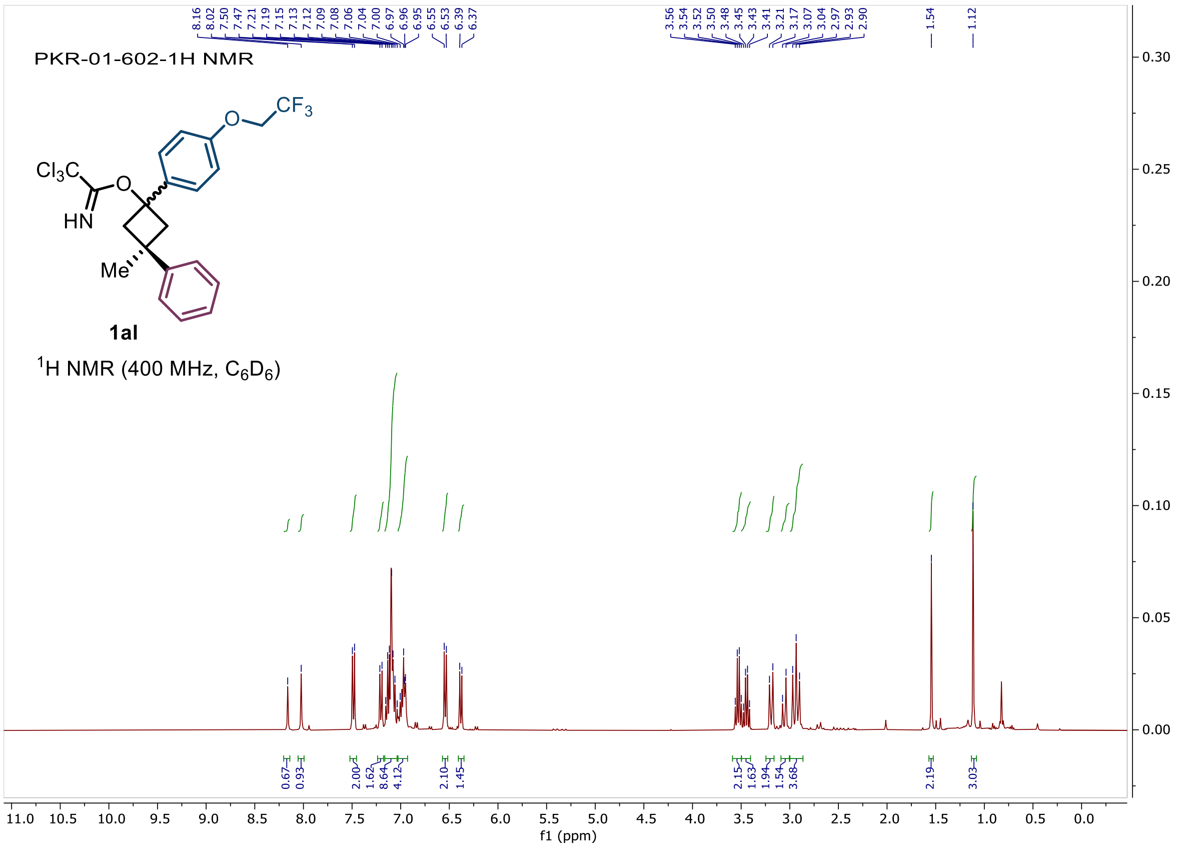
PKR-01-602-1H NMR
8.16
8.02
7.50
7.47
7.21
7.19
7.15
7.13
7.12
7.09
7.08
7.06
7.04
7.00
6.97
6.96
6.95
6.55
6.53
6.39
6.37
3.56
3.54
3.52
3.50
3.48
3.45
3.43
3.41
3.21
3.17
3.07
3.04
2.97
2.93
2.90
1.54
1.12
O
CF3
Cl3C
O
HN
Me
1al
1H NMR (400 MHz, C6D6)
0.67
0.93
2.00
1.62
8.64
4.12
2.10
1.45
2.15
1.63
1.94
1.54
3.68
2.19
3.03
f1 (ppm)

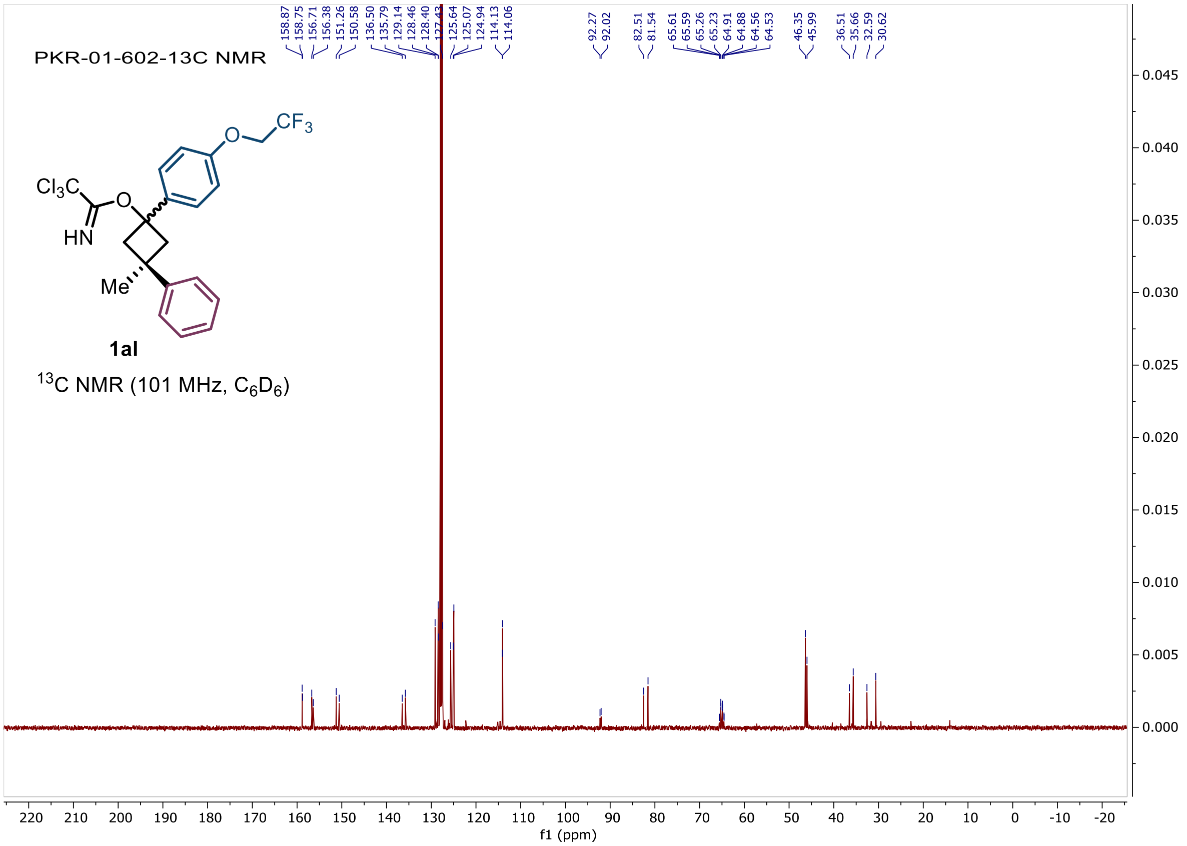
PKR-01-602-13C NMR
158.87
158.75
156.71
156.38
151.26
150.58
136.50
135.79
129.14
128.46
128.40
125.64
125.07
124.94
114.13
114.06
92.27
92.02
82.51
81.54
65.61
65.59
65.26
65.23
64.91
64.88
64.56
64.53
46.35
45.99
36.51
35.66
32.59
30.62
CF3
O
Cl3C
O
HN
Me
1al
13C NMR (101 MHz, C6D6)
f1 (ppm)

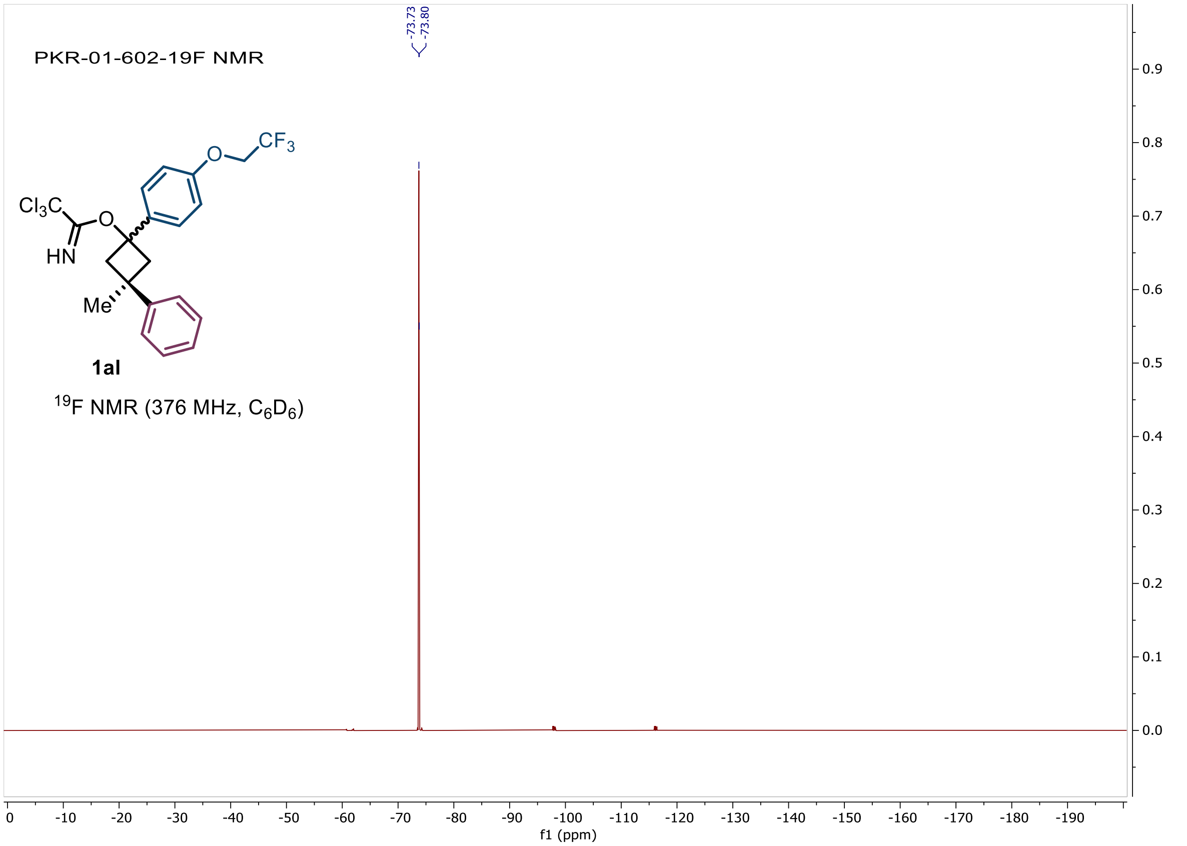
PKR-01-602-19F NMR
-73.73
-73.80
CF3
O
Cl3C
O
HN
Me
1al
19F NMR (376 MHz, C6D6)
f1 (ppm)

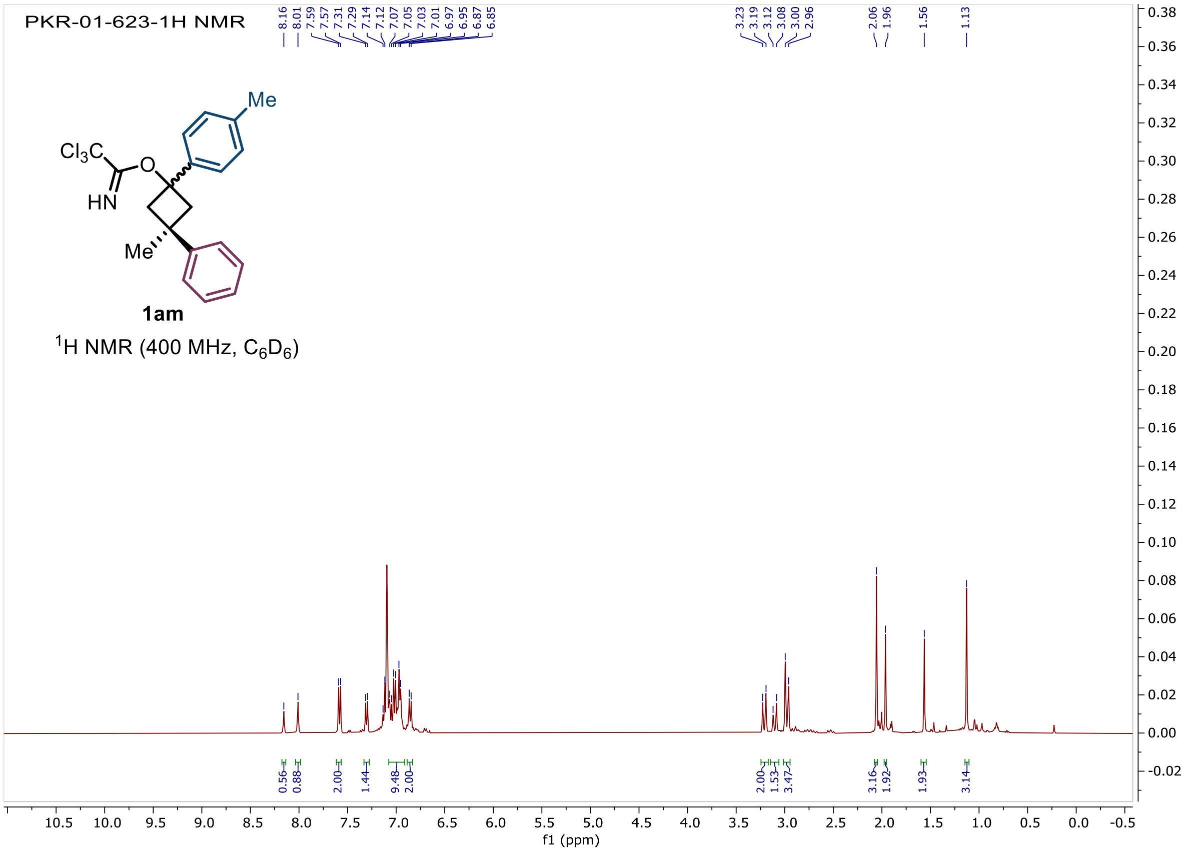
PKR-01-623-1H NMR
8.16
8.01
7.59
7.57
7.31
7.29
7.14
7.12
7.07
7.05
7.03
7.01
6.97
6.95
6.87
6.85
3.23
3.19
3.12
3.08
3.00
2.96
2.06
1.96
1.56
1.13
Me
Cl_3C
O
HN
Me
1am
^{1}H NMR (400 MHz, C_6D_6)
0.56
0.88
2.00
1.44
9.48
2.00
2.00
1.53
3.47
3.16
1.92
1.93
3.14
f1 (ppm)

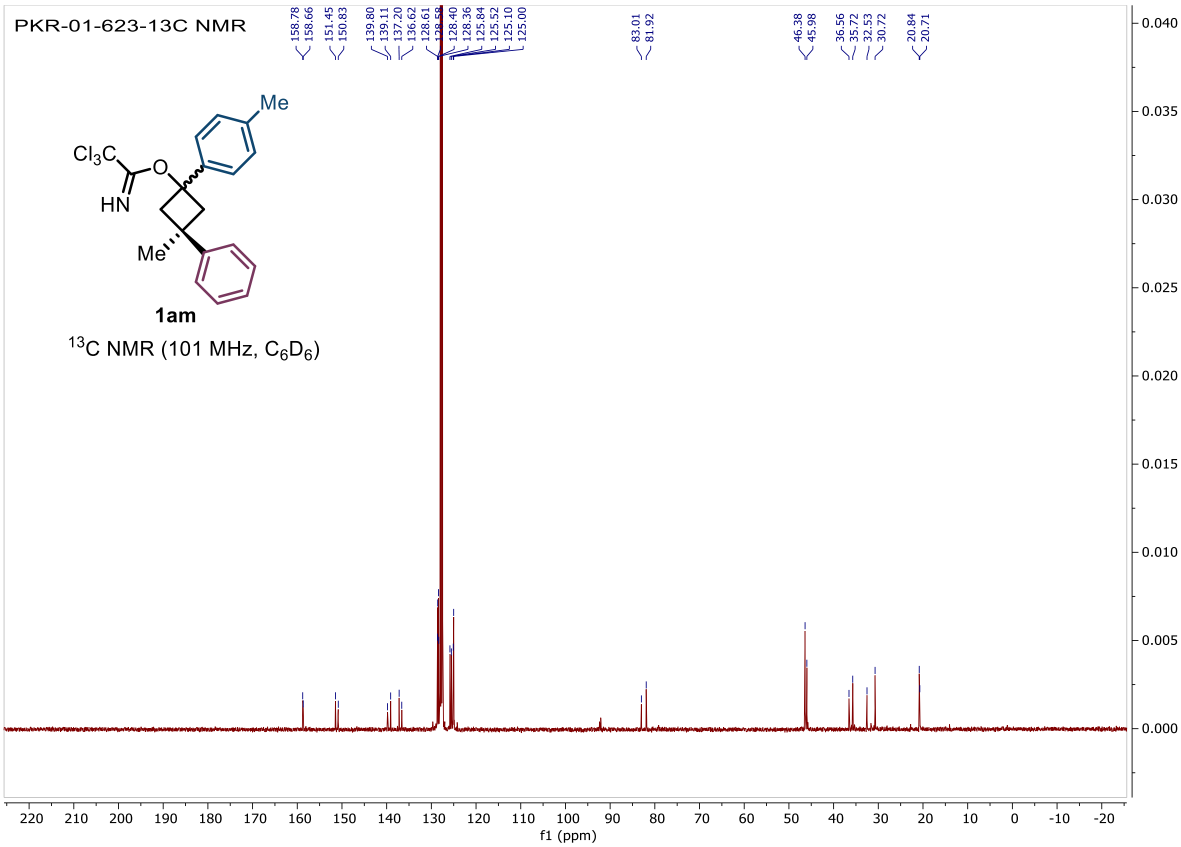
PKR-01-623-13C NMR
158.78
158.66
151.45
150.83
139.80
139.11
137.20
136.62
128.61
128.58
128.40
128.36
125.84
125.52
125.10
125.00
83.01
81.92
46.38
45.98
36.56
35.72
32.53
30.72
20.84
20.71
Me
Cl_3C
O
HN
Me
1am
^{13}C NMR (101 MHz, C_6D_6)
f1 (ppm)

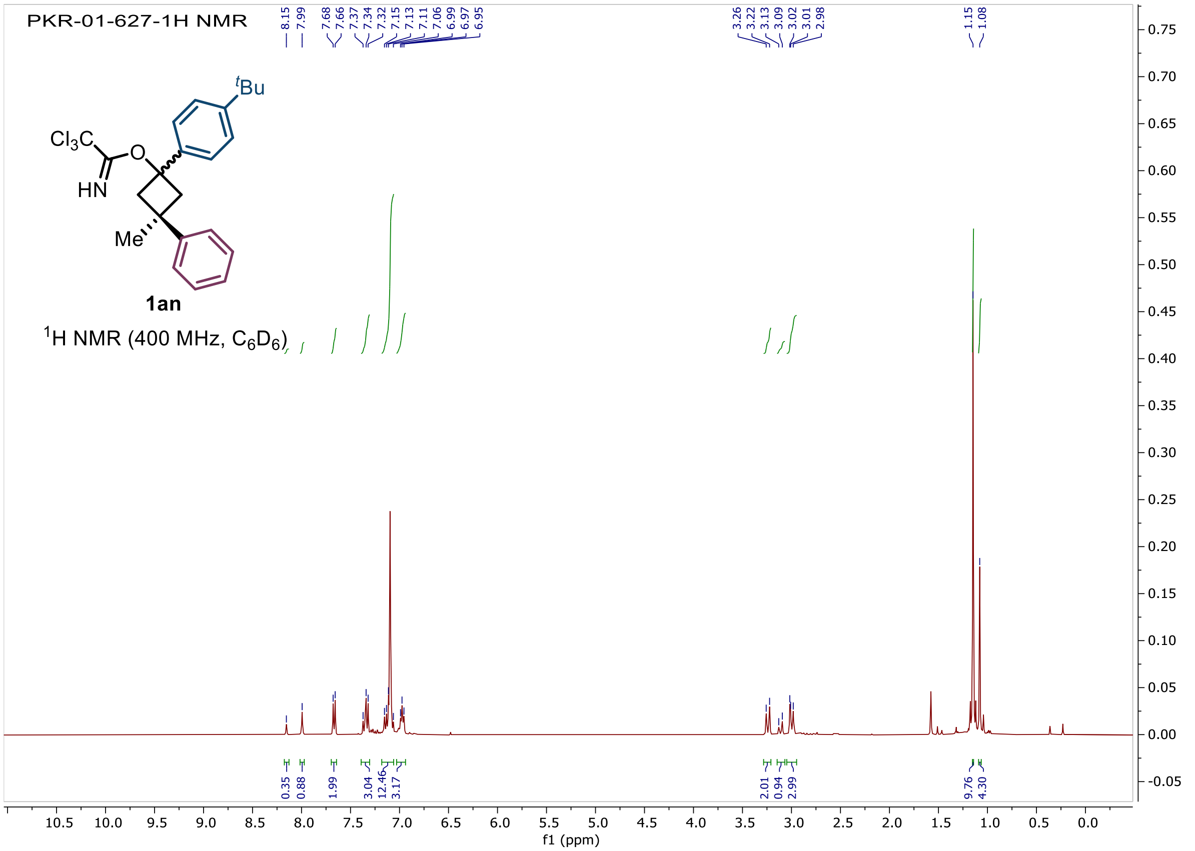
PKR-01-627-1H NMR
8.15
7.99
7.68
7.66
7.37
7.34
7.32
7.15
7.13
7.11
7.06
6.99
6.97
6.95
3.26
3.22
3.13
3.09
3.02
3.01
2.98
1.15
1.08
tBu
Cl3C
O
HN
Me
1an
1H NMR (400 MHz, C6D6)
0.35
0.88
1.99
3.04
12.46
3.17
2.01
0.94
2.99
9.76
4.30
f1 (ppm)

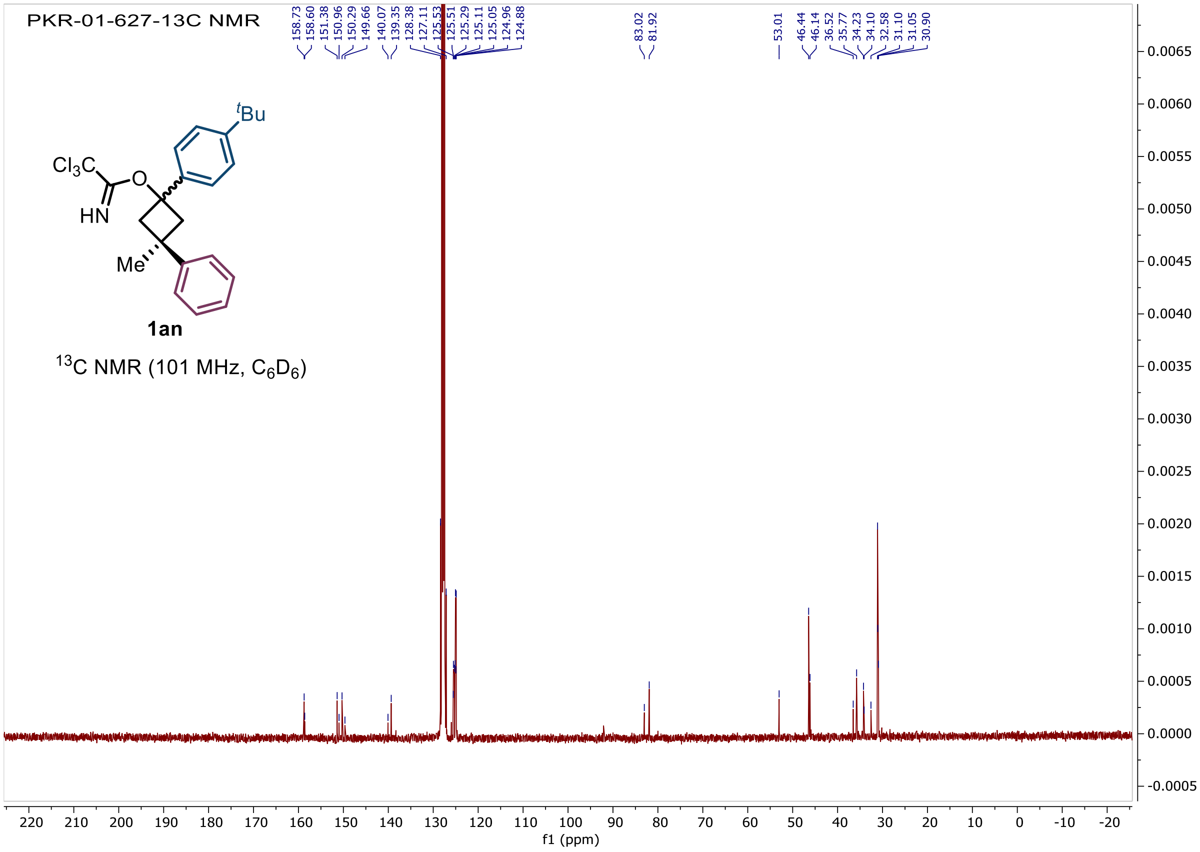
PKR-01-627-13C NMR
158.73
158.60
151.38
150.96
150.29
149.66
140.07
139.35
128.38
127.11
125.53
125.51
125.29
125.11
125.05
124.96
124.88
83.02
81.92
53.01
46.44
46.14
36.52
35.77
34.23
34.10
32.58
31.10
31.05
30.90
tBu
Cl3C
O
HN
Me
1an
13C NMR (101 MHz, C6D6)
f1 (ppm)

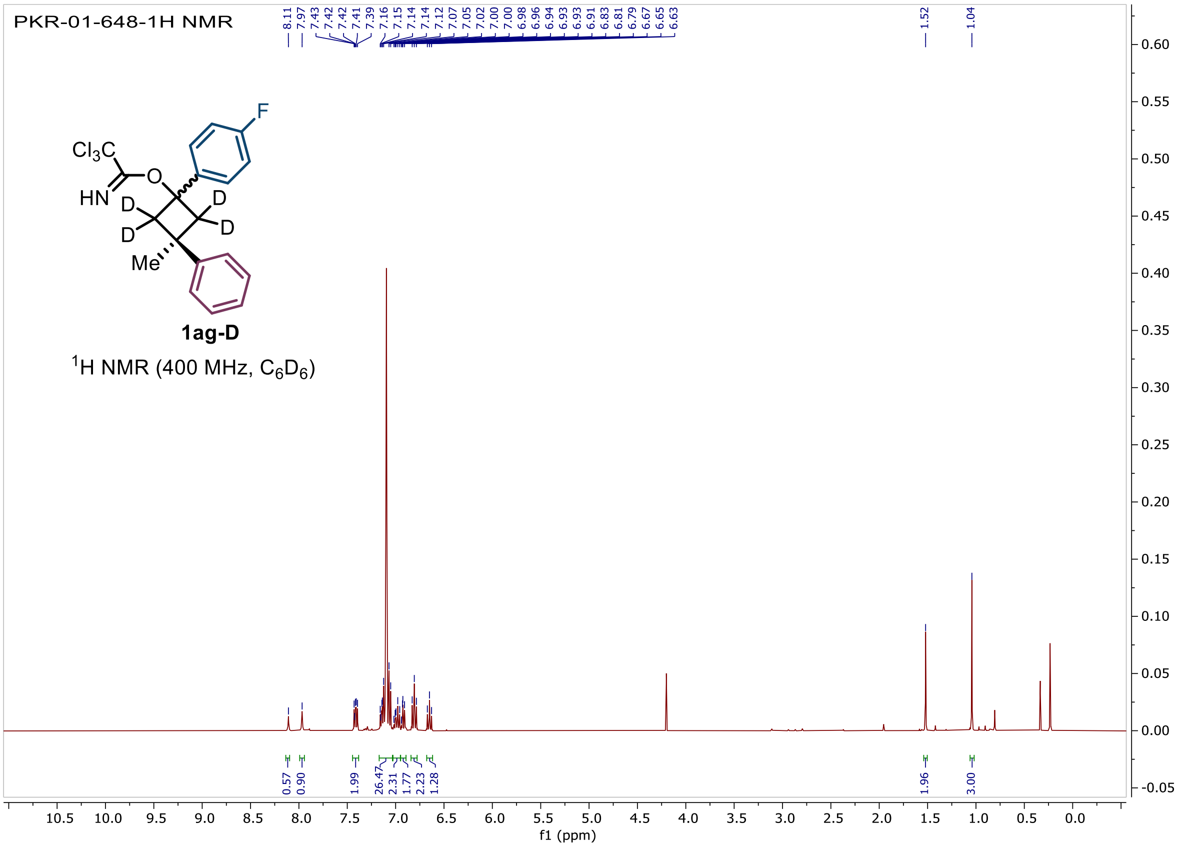
PKR-01-648-1H NMR
8.11 7.97 7.43 7.42 7.42 7.41 7.39 7.16 7.15 7.14 7.14 7.12 7.07 7.05 7.02 7.00 7.00 6.98 6.96 6.94 6.93 6.93 6.91 6.83 6.81 6.79 6.67 6.65 6.63
1.52 1.04
Cl_3C
O
F
HN
D
D
D
D
Me
1ag-D
^1H NMR (400 MHz, C_6D_6)
0.57 0.90 1.99 26.47 2.31 1.77 2.23 1.28 1.96 3.00
f1 (ppm)

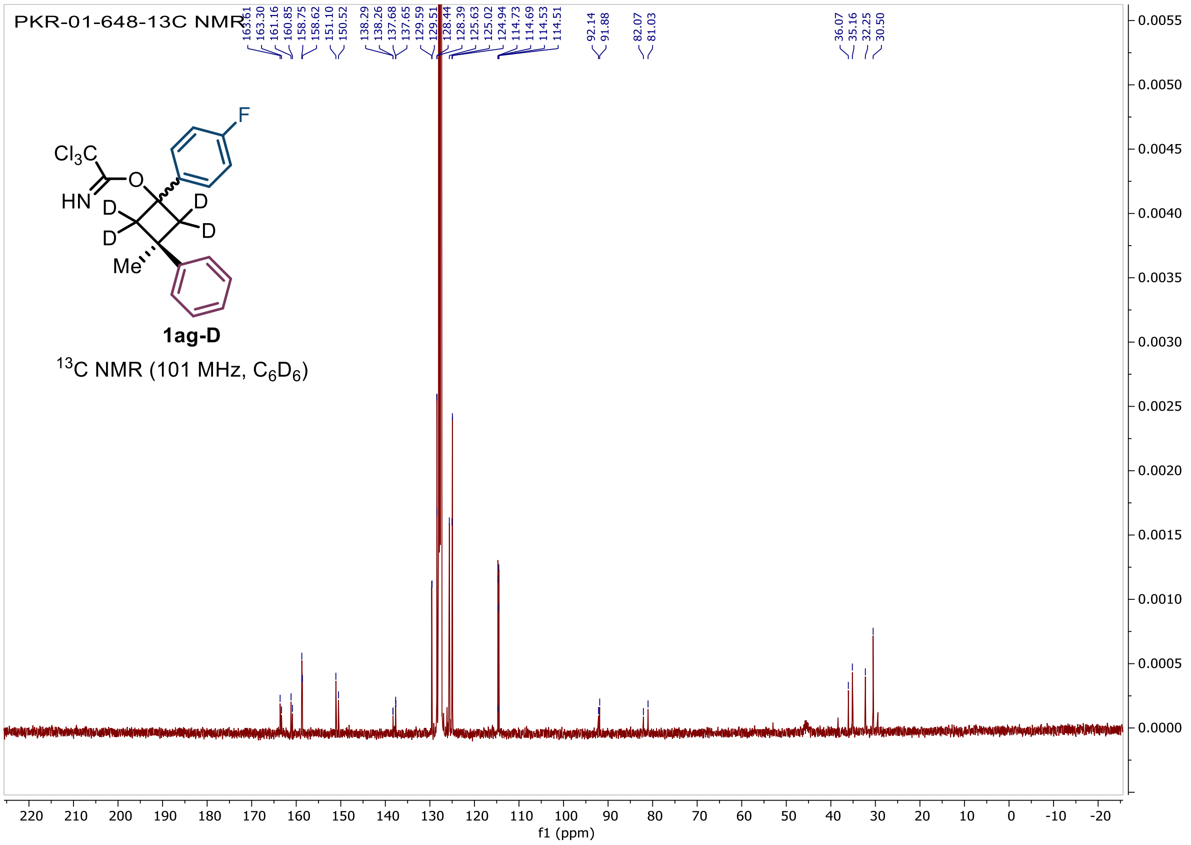
PKR-01-648-13C NMR
163.61 163.30 161.16 160.85 158.75 158.62 151.10 150.52 138.29 138.26 137.68 137.65 129.59 129.51 128.44 128.39 125.63 125.02 124.94 114.73 114.69 114.53 114.51
92.14 91.88 82.07 81.03
36.07 35.16 32.25 30.50
Cl_3C
O
F
HN
D
D
D
D
Me
1ag-D
^{13}C NMR (101 MHz, C_6D_6)
f1 (ppm)

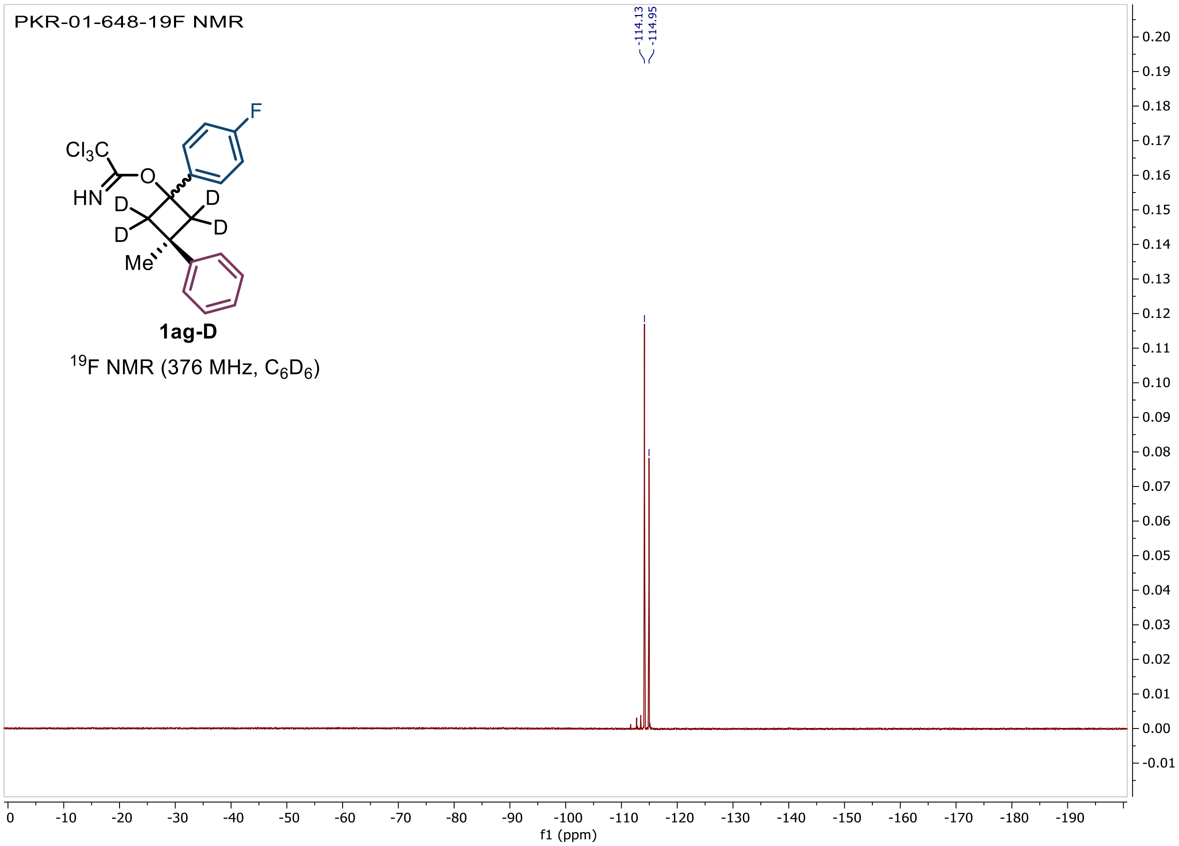
PKR-01-648-19F NMR
1ag-D
^{19}F NMR (376 MHz, C_6D_6)
-114.13
-114.95
f1 (ppm)

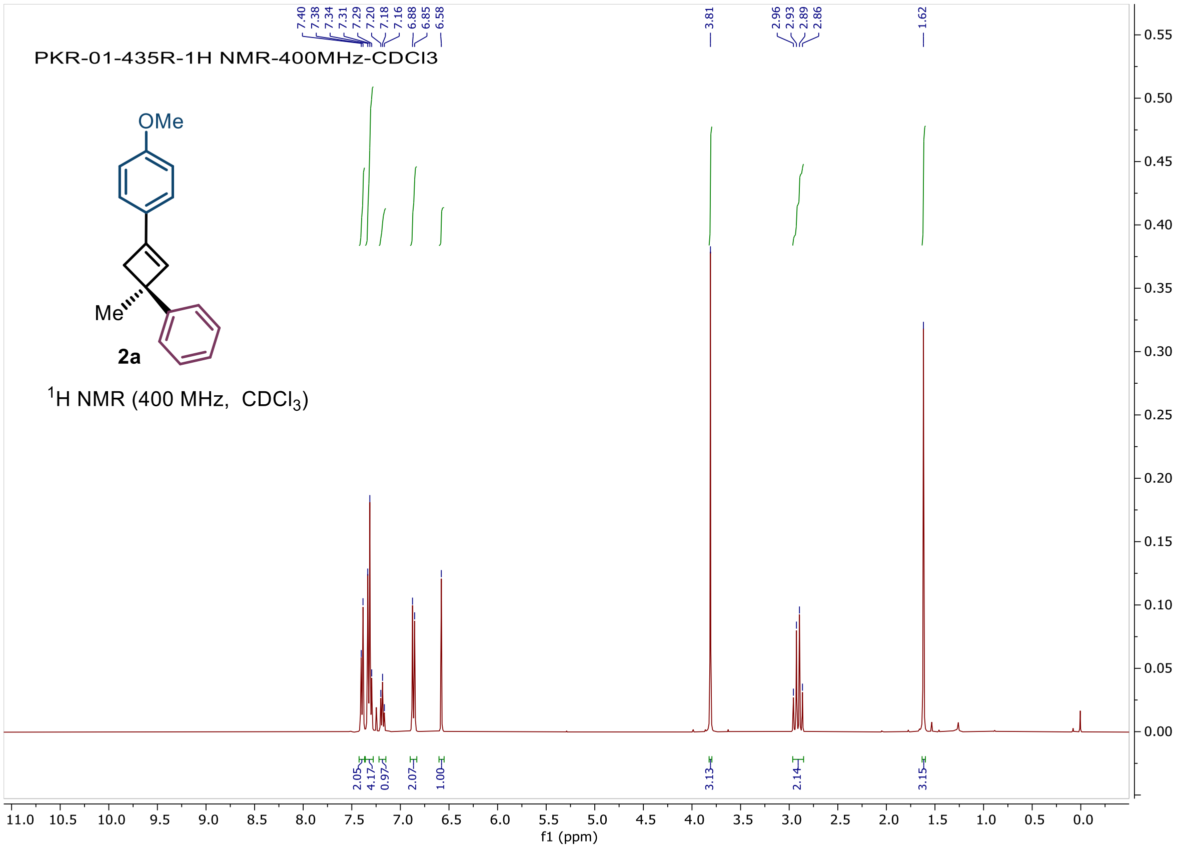
PKR-01-435R-1H NMR-400MHz-CDCl3
2a
^{1}H NMR (400 MHz, $CDCl_3$)
7.40
7.38
7.34
7.31
7.29
7.20
7.18
7.16
6.88
6.85
6.58
3.81
2.96
2.93
2.89
2.86
1.62
2.05
4.17
0.97
2.07
1.00
3.13
2.14
3.15
f1 (ppm)

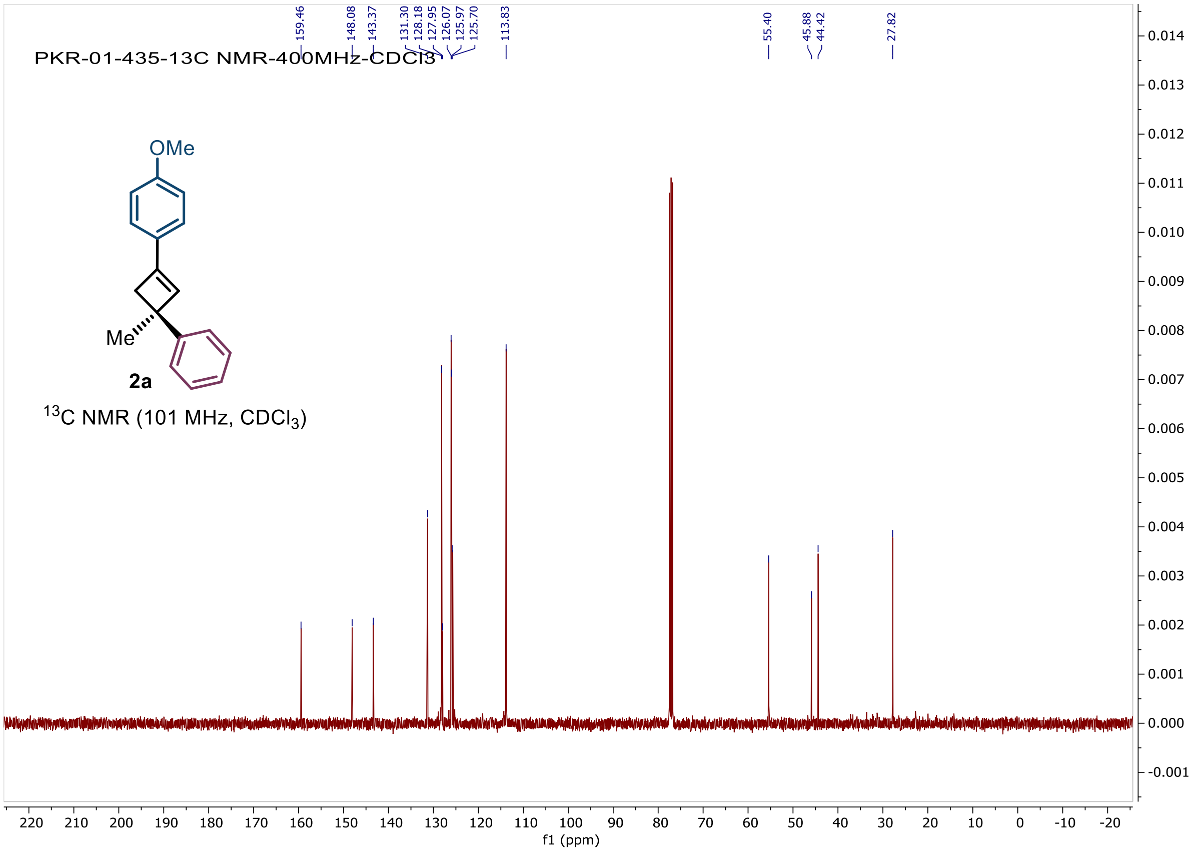
PKR-01-435-13C NMR-400MHz-CDCl3
OMe
Me
2a
^{13}C NMR (101 MHz, CDCl$_3$)
159.46
148.08
143.37
131.30
128.18
127.95
126.07
125.97
125.70
113.83
55.40
45.88
44.42
27.82
f1 (ppm)

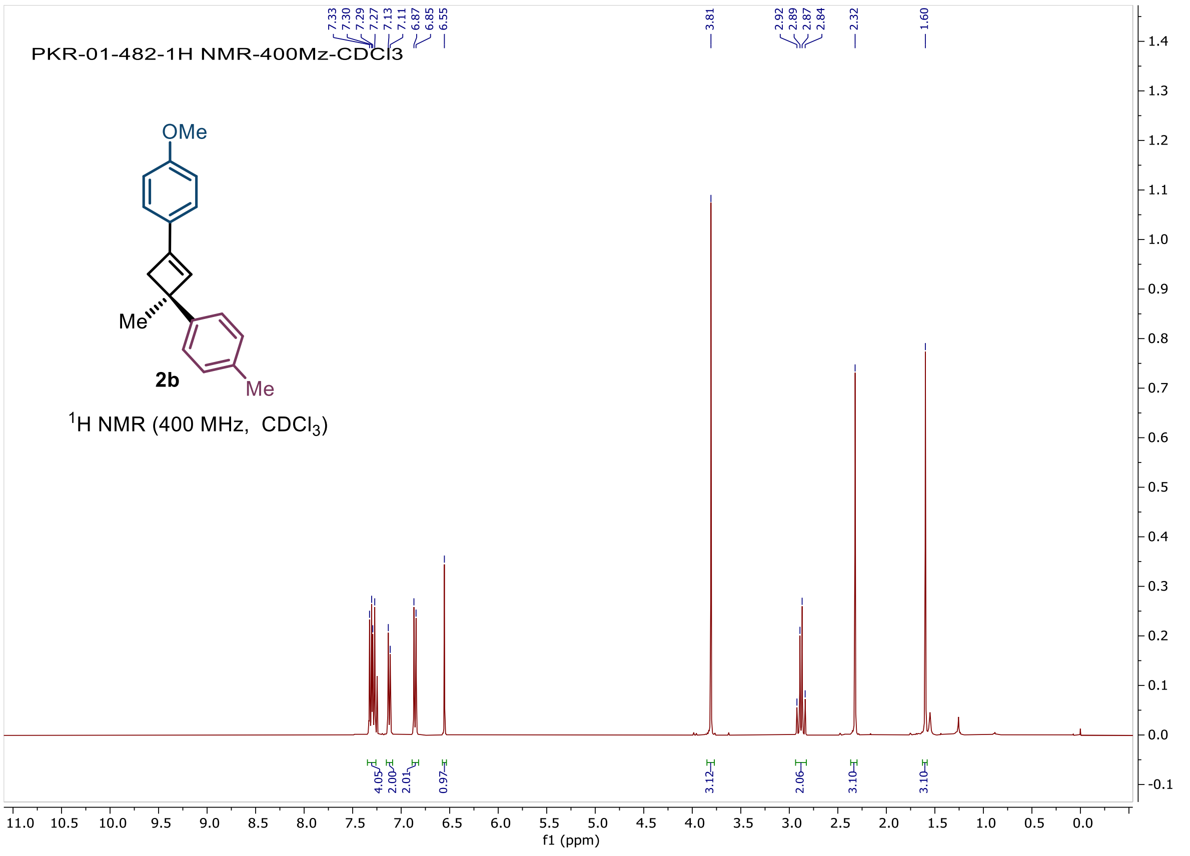
PKR-01-482-1H NMR-400Mz-CDCl3
OMe
Me
2b
Me
^{1}H NMR (400 MHz, CDCl$_3$)
7.33
7.30
7.29
7.27
7.13
7.11
6.87
6.85
6.55
3.81
2.92
2.89
2.87
2.84
2.32
1.60
4.05
2.00
2.01
0.97
3.12
2.06
3.10
3.10
f1 (ppm)

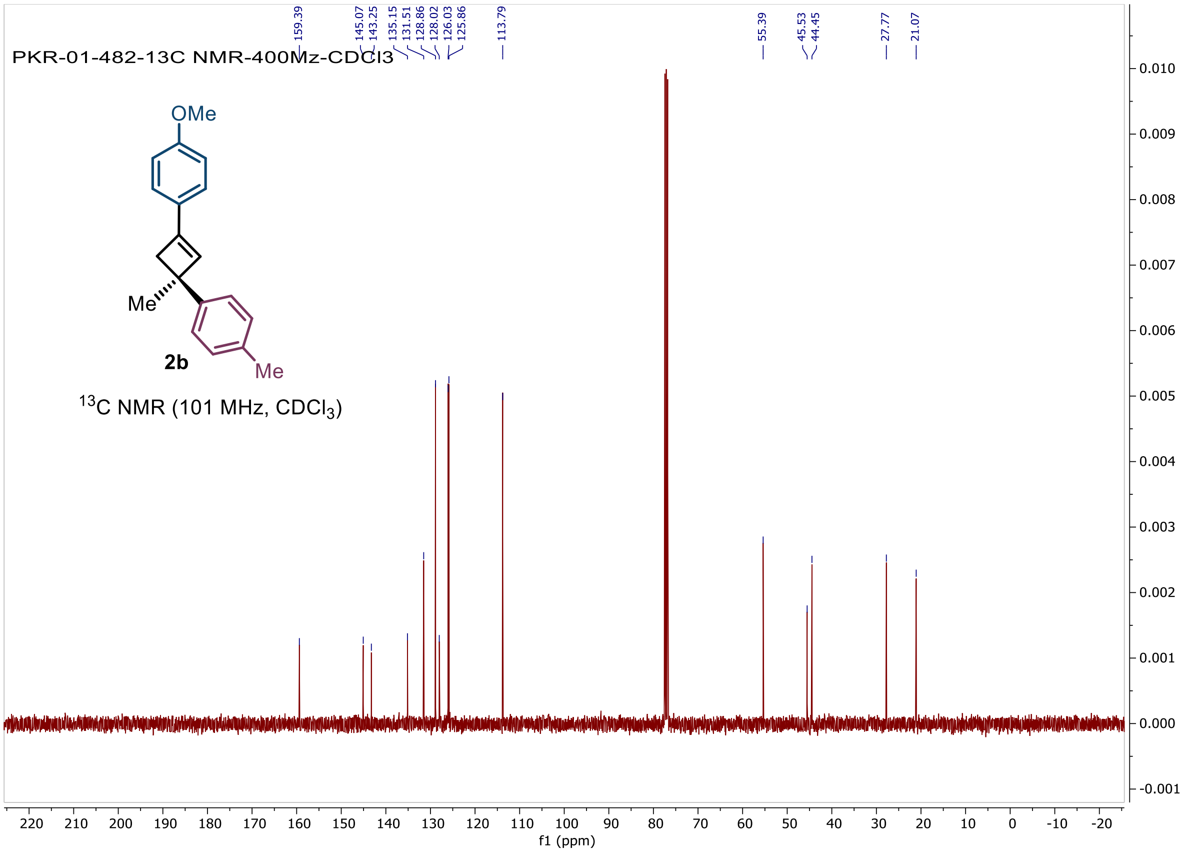
PKR-01-482-13C NMR-400Mz-CDCl3
OMe
Me
2b
Me
13C NMR (101 MHz, CDCl3)
159.39
145.07
143.25
135.15
131.51
128.86
128.02
126.03
125.86
113.79
55.39
45.53
44.45
27.77
21.07
f1 (ppm)

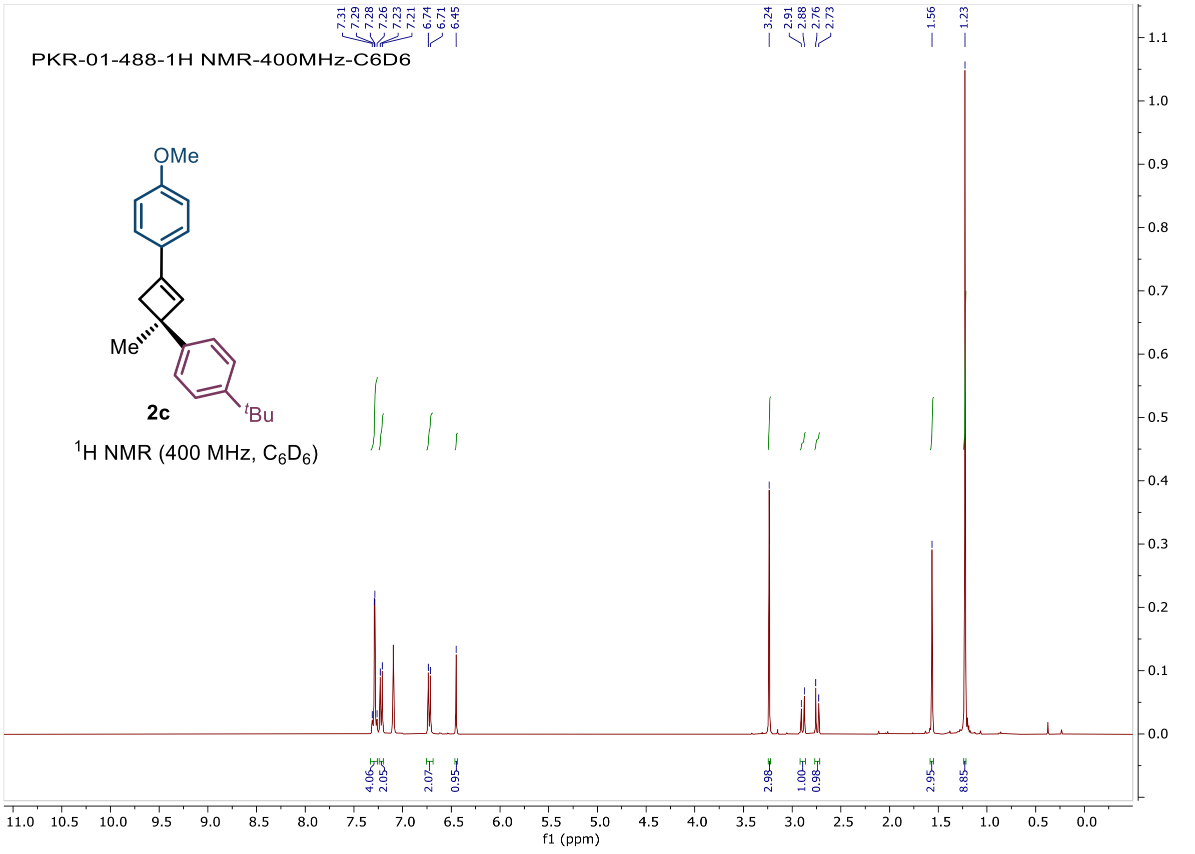
PKR-01-488-1H NMR-400MHz-C6D6
OMe
Me
2c
tBu
1H NMR (400 MHz, C6D6)
7.31
7.29
7.28
7.26
7.23
7.21
6.74
6.71
6.45
3.24
2.91
2.88
2.76
2.73
1.56
1.23
4.06
2.05
2.07
0.95
2.98
1.00
0.98
2.95
8.85
f1 (ppm)

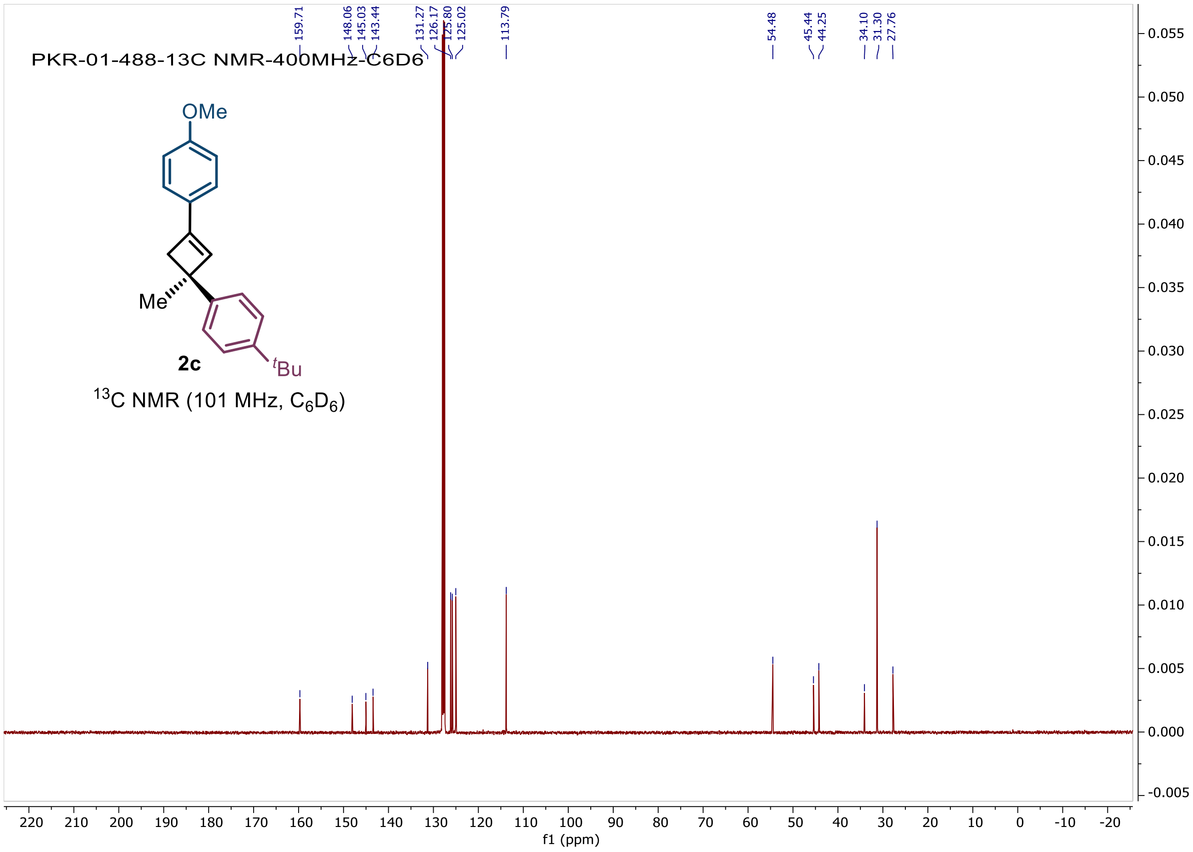
PKR-01-488-13C NMR-400MHz-C6D6
159.71
148.06
145.03
143.44
131.27
126.17
125.80
125.02
113.79
54.48
45.44
44.25
34.10
31.30
27.76
OMe
Me
2c
^{t}Bu
^{13}C NMR (101 MHz, C_6D_6)
f1 (ppm)

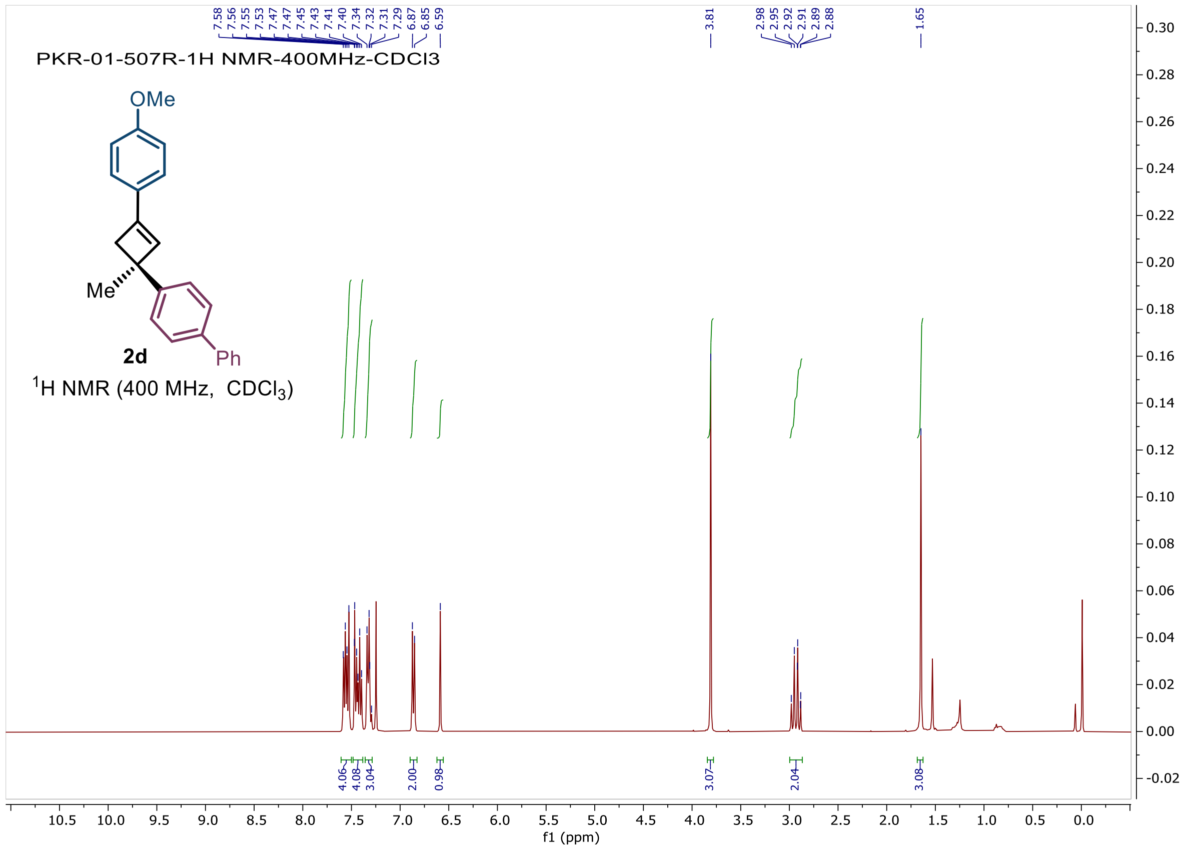
PKR-01-507R-1H NMR-400MHz-CDCl3
7.58
7.56
7.55
7.53
7.47
7.45
7.43
7.41
7.40
7.34
7.32
7.31
7.29
6.87
6.85
6.59
3.81
2.98
2.95
2.92
2.91
2.89
2.88
1.65
OMe
Me
2d
Ph
^{1}H NMR (400 MHz, $CDCl_3$)
4.06
4.08
3.04
2.00
0.98
3.07
2.04
3.08
f1 (ppm)

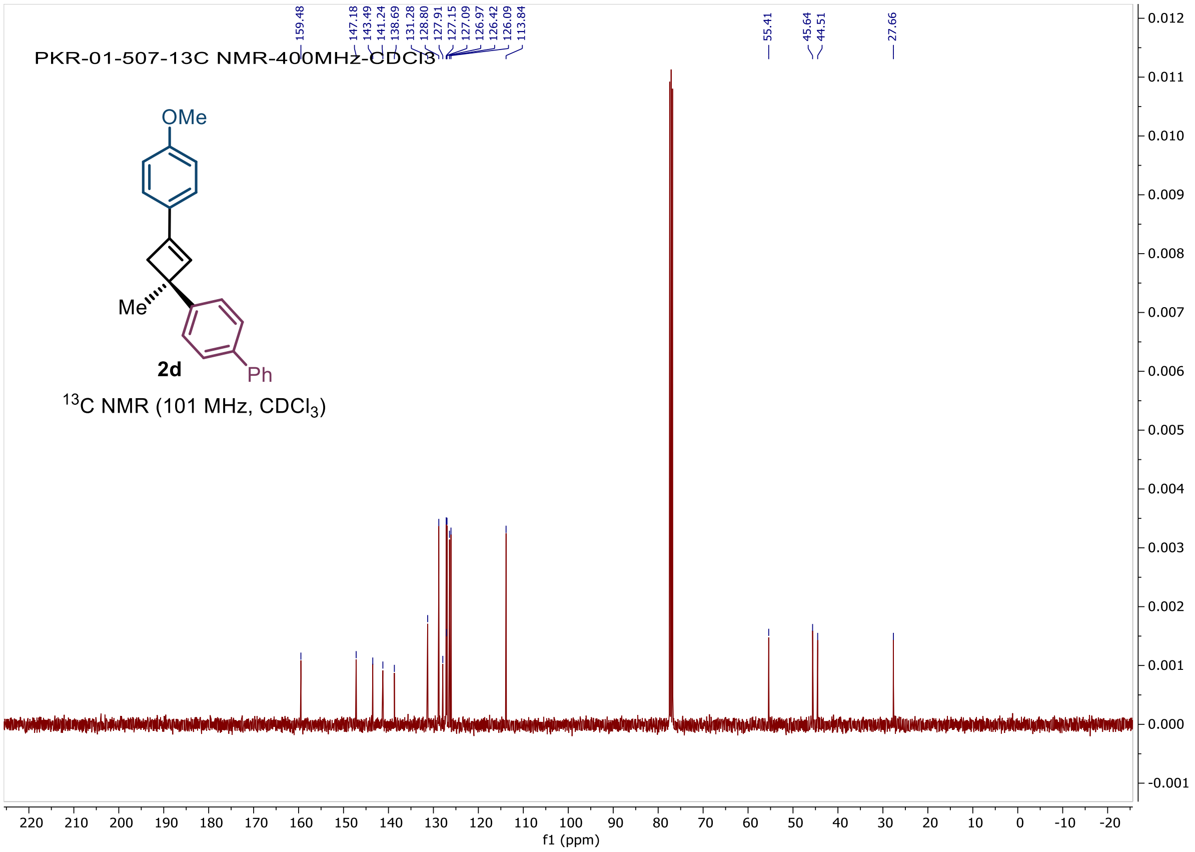
PKR-01-507-13C NMR-400MHz-CDCl3
2d
13C NMR (101 MHz, CDCl3)
159.48
147.18
143.49
141.24
138.69
131.28
128.80
127.91
127.15
127.09
126.97
126.42
126.09
113.84
55.41
45.64
44.51
27.66
f1 (ppm)

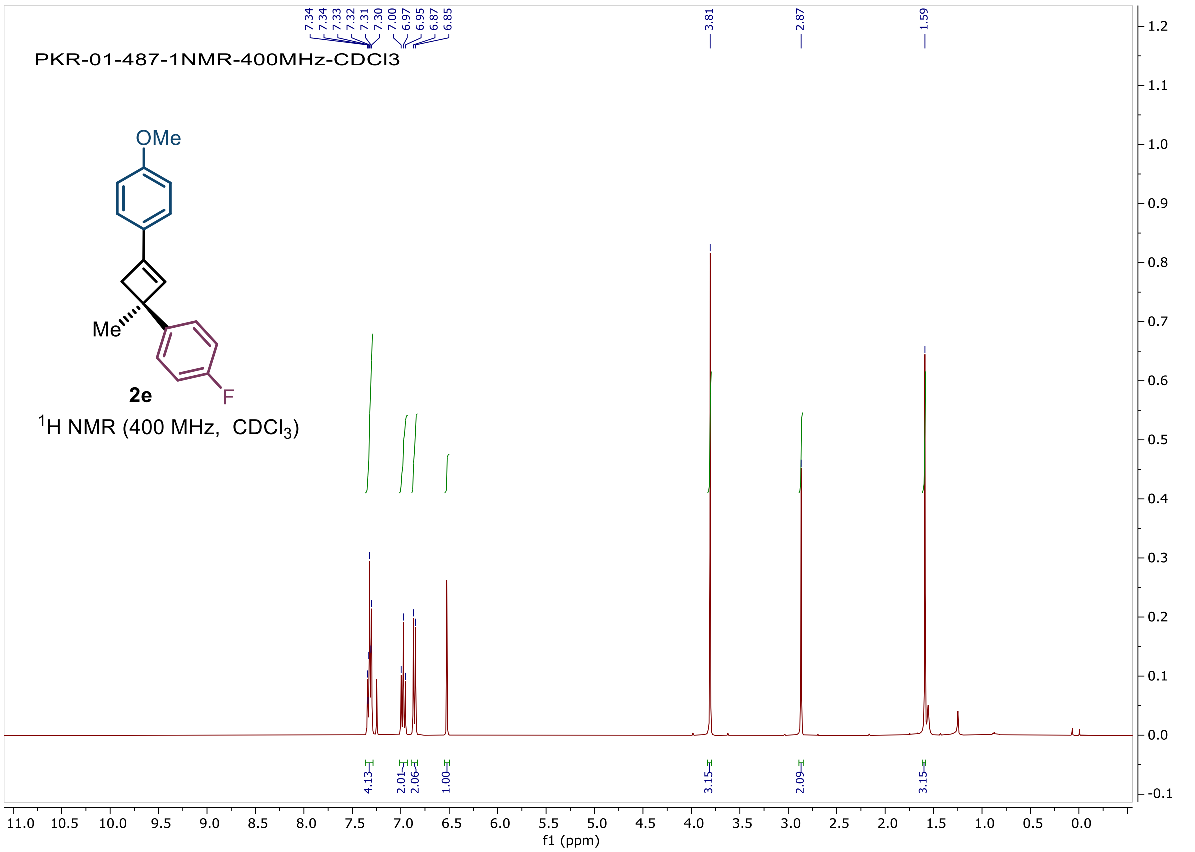
PKR-01-487-1NMR-400MHz-CDCl3
2e
1H NMR (400 MHz, CDCl3)
7.34
7.34
7.33
7.32
7.31
7.30
7.00
6.97
6.95
6.87
6.85
3.81
2.87
1.59
4.13
2.01
2.06
1.00
3.15
2.09
3.15
f1 (ppm)

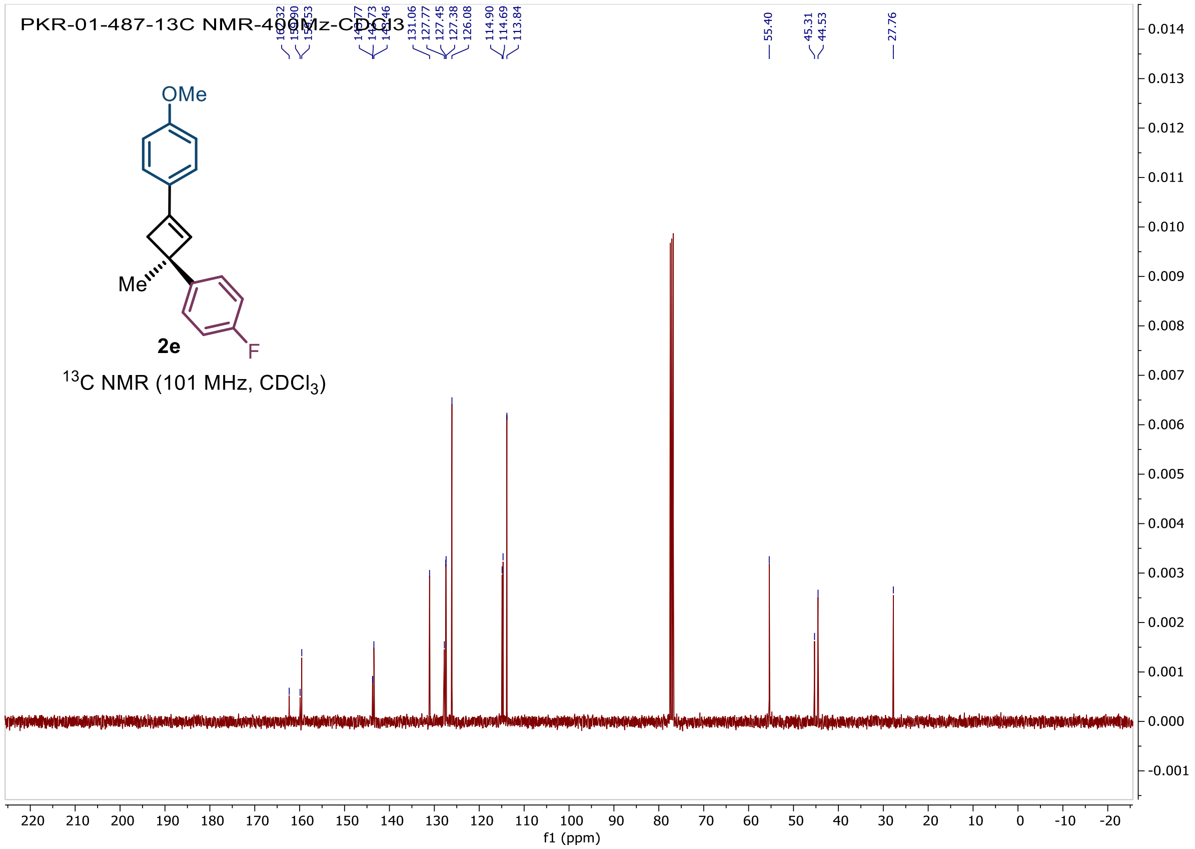
PKR-01-487-13C NMR-400MHz-CDCl3
OMe
Me
2e
F
^{13}C NMR (101 MHz, $CDCl_3$)
55.40
45.31
44.53
27.76
131.06
127.77
127.45
127.38
126.08
114.90
114.69
113.84
f1 (ppm)

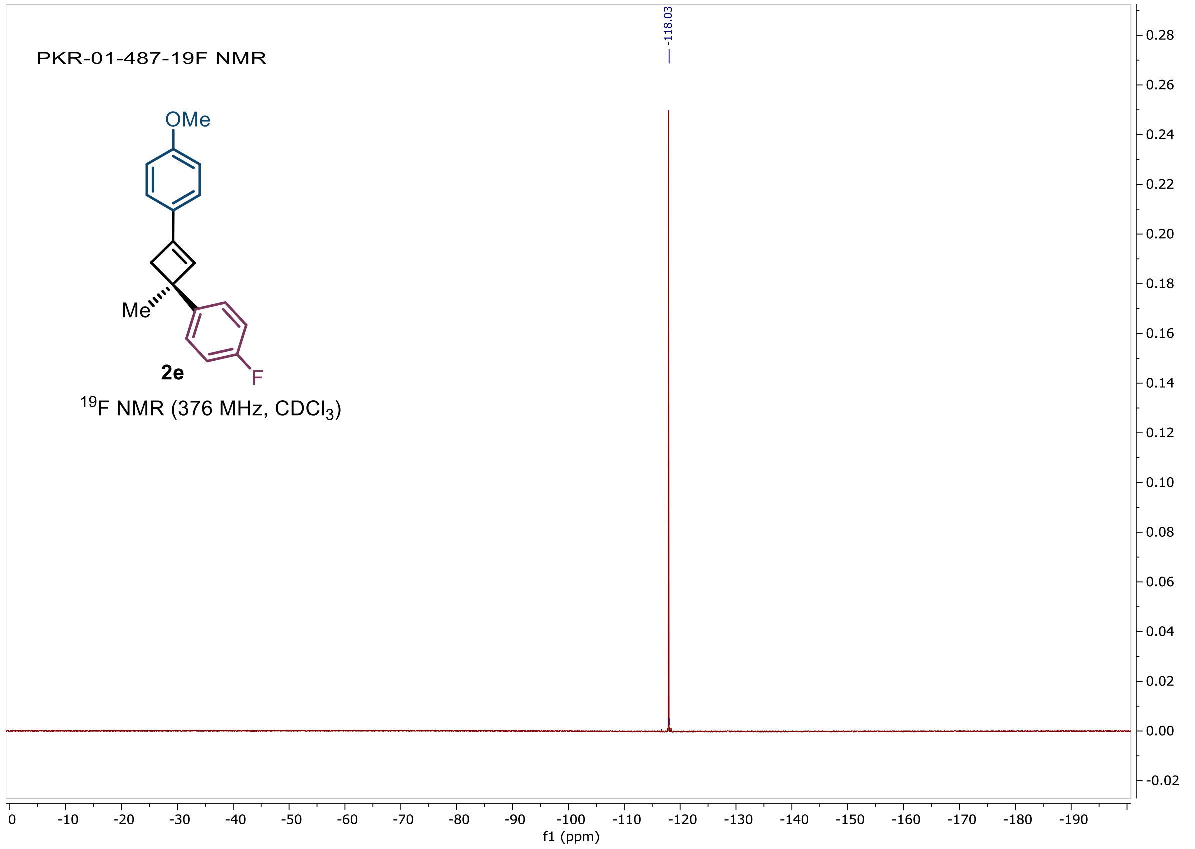
PKR-01-487-19F NMR
-118.03
OMe
Me
2e
F
^{19}F NMR (376 MHz, $CDCl_3$)
f1 (ppm)

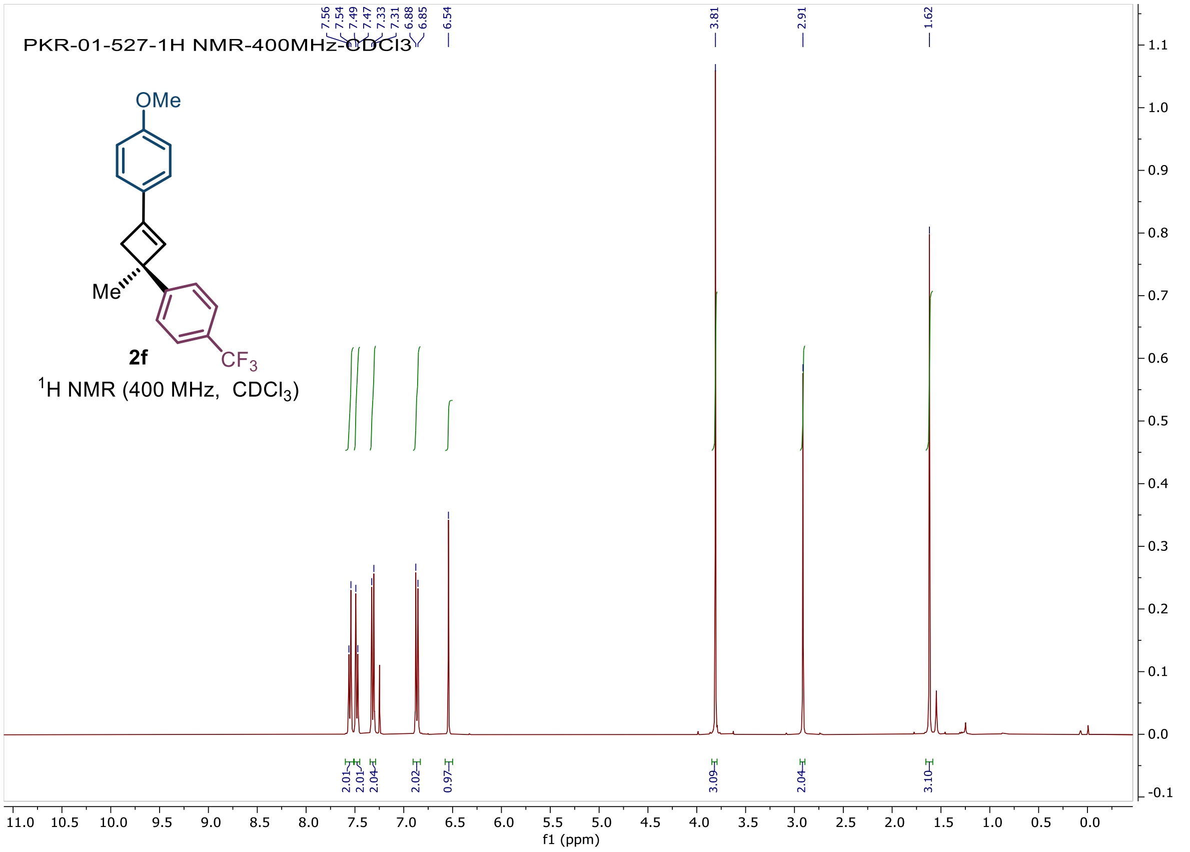
PKR-01-527-1H NMR-400MHz-CDCl3
OMe
Me
2f
CF3
1H NMR (400 MHz, CDCl3)
7.56
7.54
7.49
7.47
7.33
7.31
6.88
6.85
6.54
3.81
2.91
1.62
2.01
2.01
2.04
2.02
0.97
3.09
2.04
3.10
f1 (ppm)

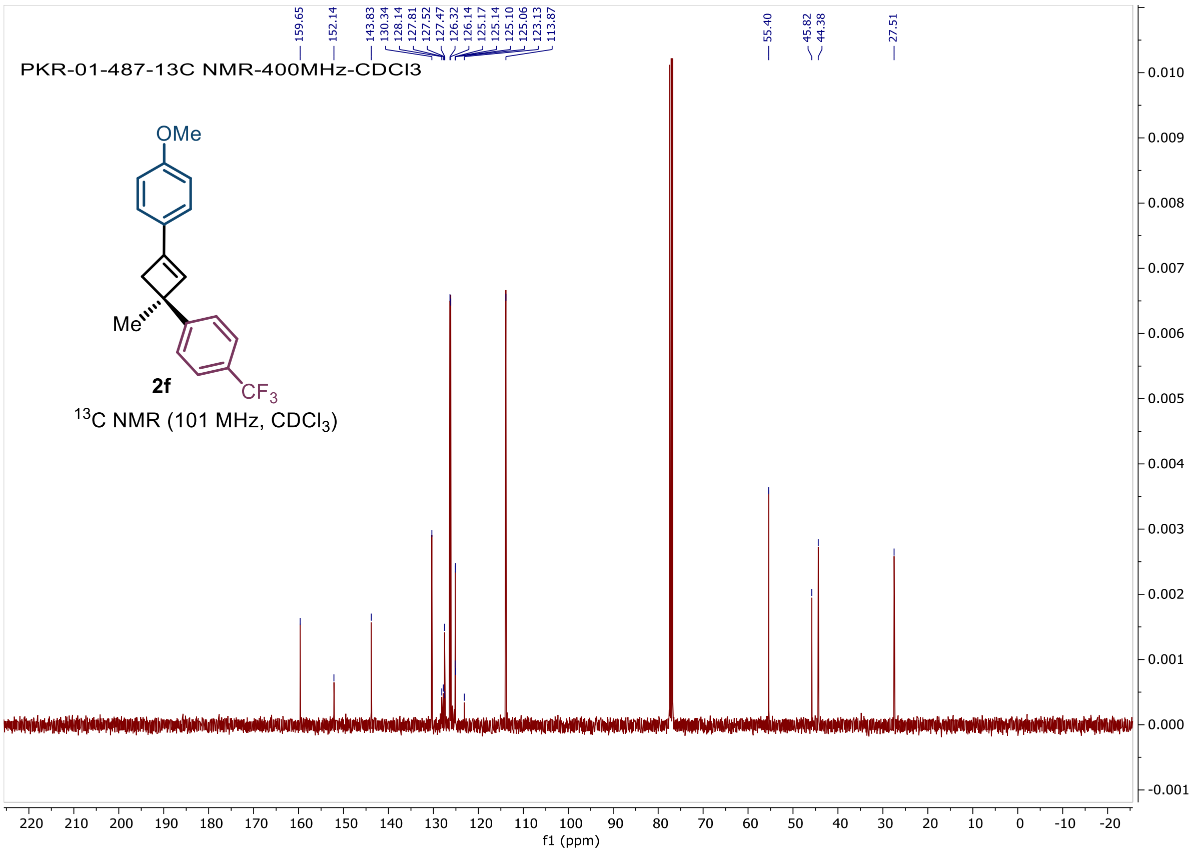
PKR-01-487-13C NMR-400MHz-CDCl3
OMe
Me
2f
CF3
13C NMR (101 MHz, CDCl3)
159.65
152.14
143.83
130.34
128.14
127.81
127.52
127.47
126.32
126.14
125.17
125.14
125.10
125.06
123.13
113.87
55.40
45.82
44.38
27.51
f1 (ppm)

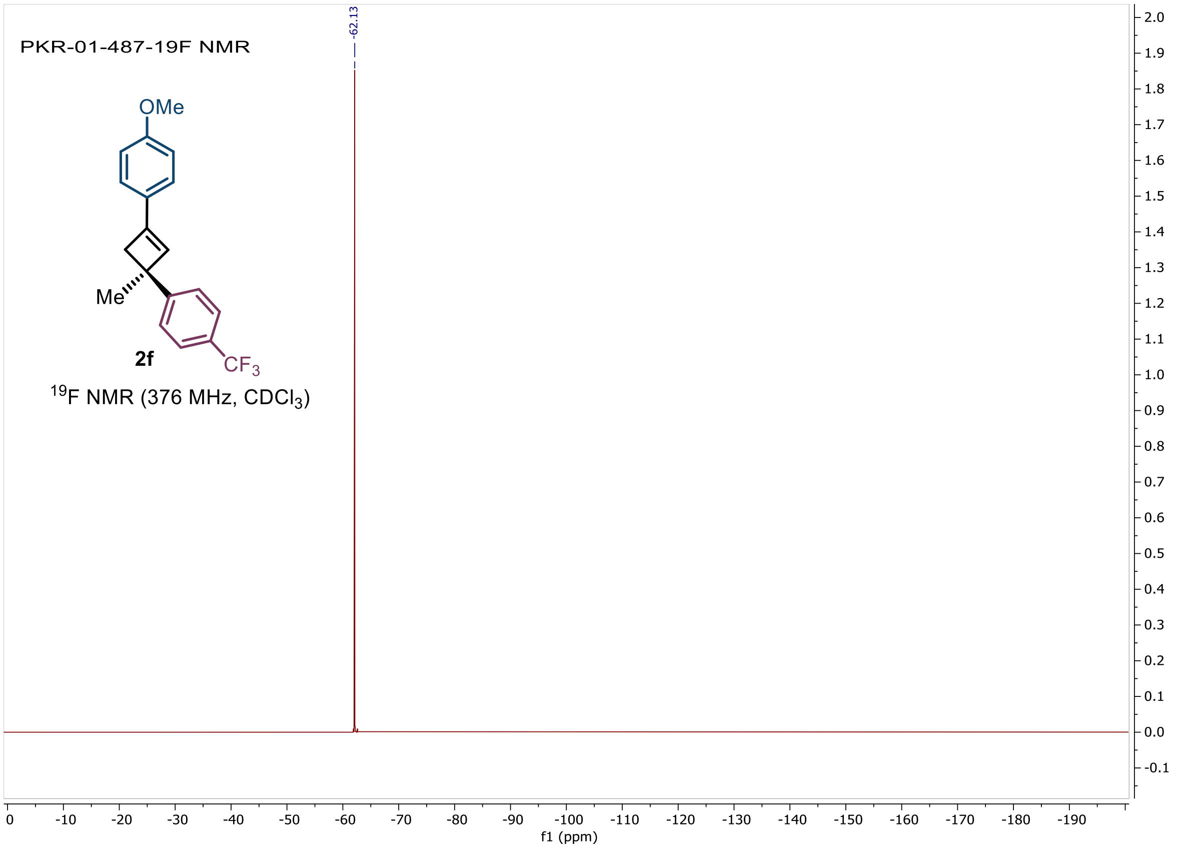
PKR-01-487-19F NMR
OMe
Me
2f
CF3
19F NMR (376 MHz, CDCl3)
-62.13
f1 (ppm)

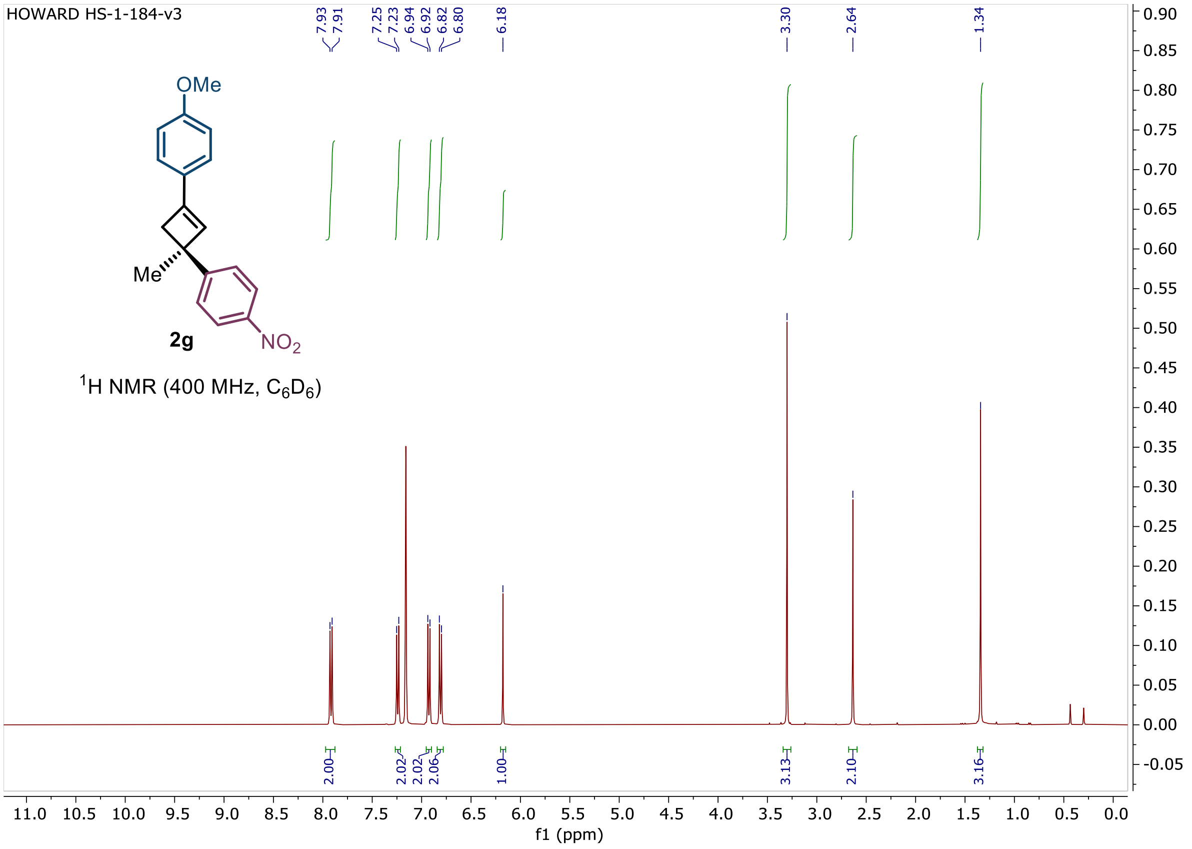
HOWARD HS-1-184-v3
OMe
Me
2g
NO2
1H NMR (400 MHz, C6D6)
7.93
7.91
7.25
7.23
6.94
6.92
6.82
6.80
6.18
3.30
2.64
1.34
2.00
2.02
2.02
2.06
1.00
3.13
2.10
3.16
f1 (ppm)

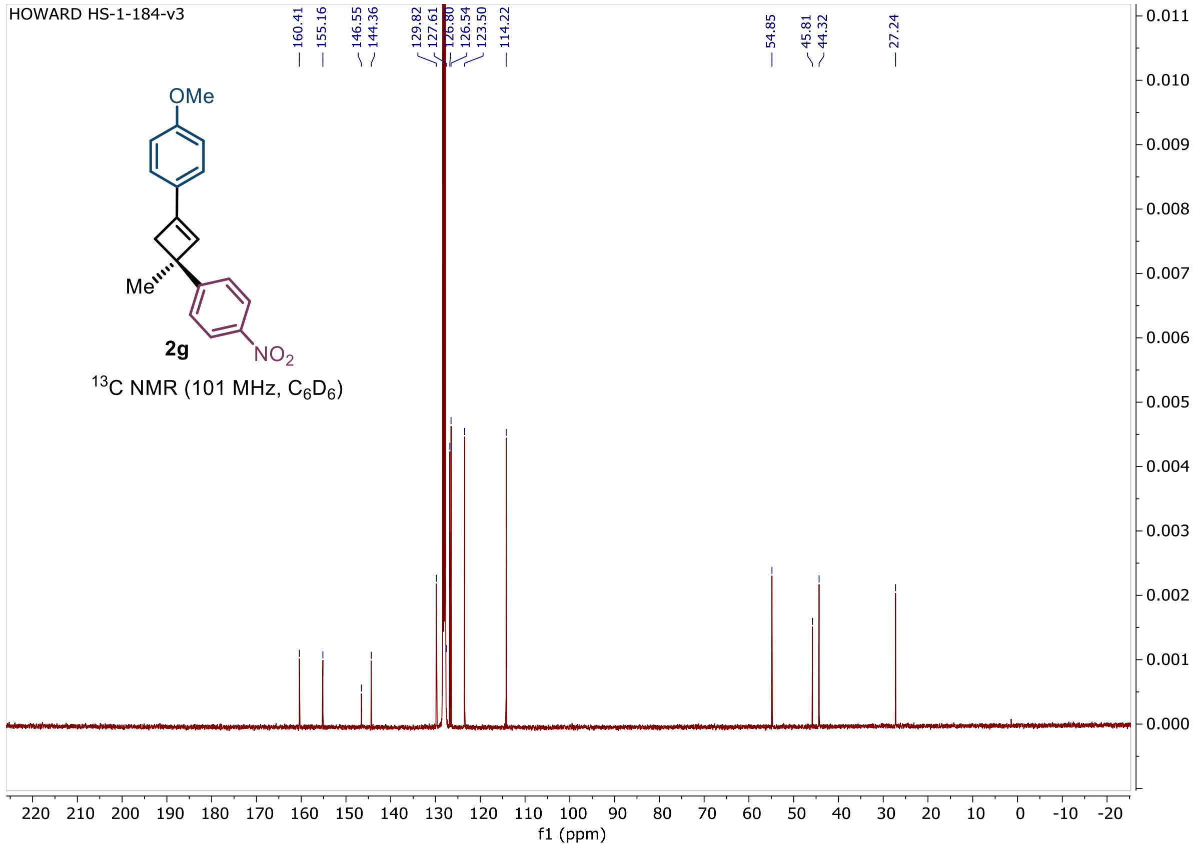
HOWARD HS-1-184-v3
160.41
155.16
146.55
144.36
129.82
127.61
126.80
126.54
123.50
114.22
54.85
45.81
44.32
27.24
OMe
Me
NO2
2g
13C NMR (101 MHz, C6D6)
f1 (ppm)

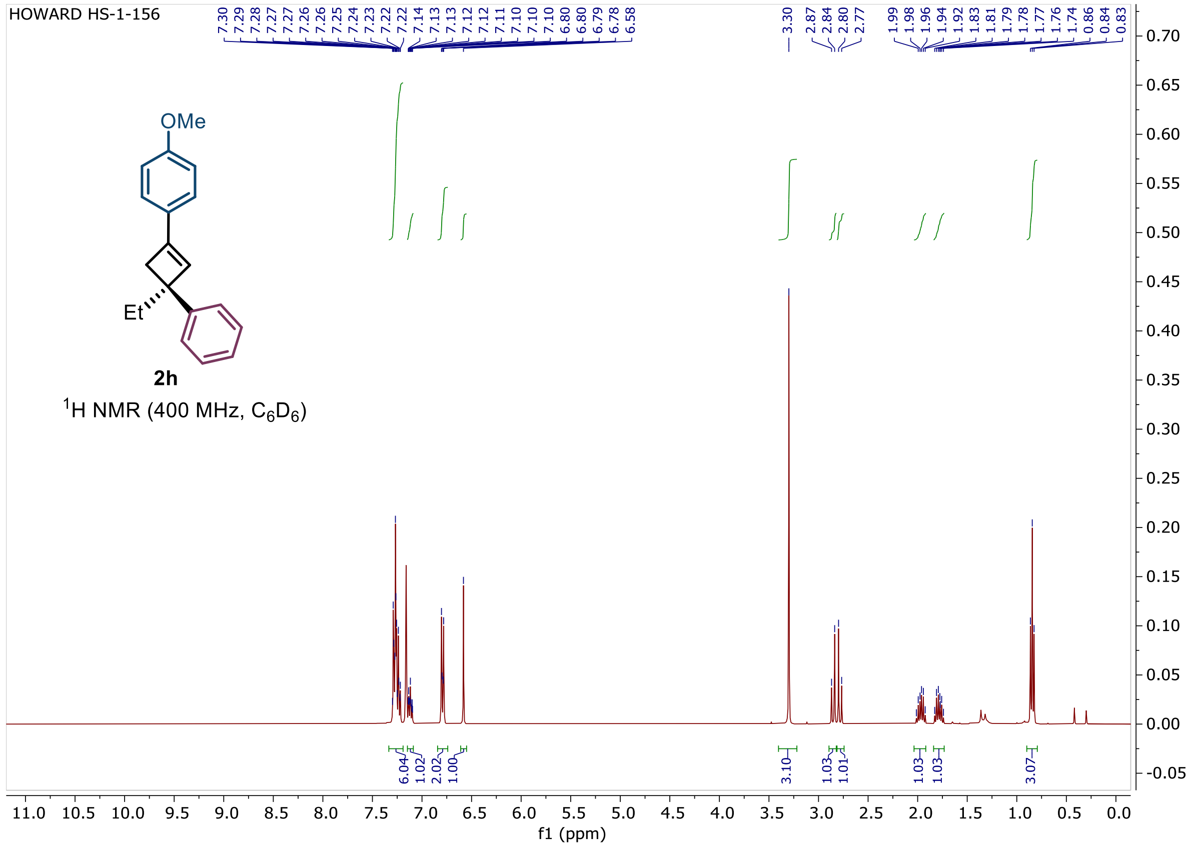
HOWARD HS-1-156
7.30
7.29
7.28
7.27
7.27
7.26
7.26
7.25
7.24
7.23
7.22
7.22
7.14
7.13
7.13
7.12
7.12
7.11
7.10
7.10
6.80
6.80
6.79
6.78
6.58
3.30
2.87
2.84
2.80
2.77
1.99
1.98
1.96
1.94
1.92
1.83
1.81
1.79
1.78
1.77
1.76
1.74
0.86
0.84
0.83
OMe
Et
2h
1H NMR (400 MHz, C6D6)
6.04
1.02
2.02
1.00
3.10
1.03
1.01
1.03
1.03
3.07
f1 (ppm)

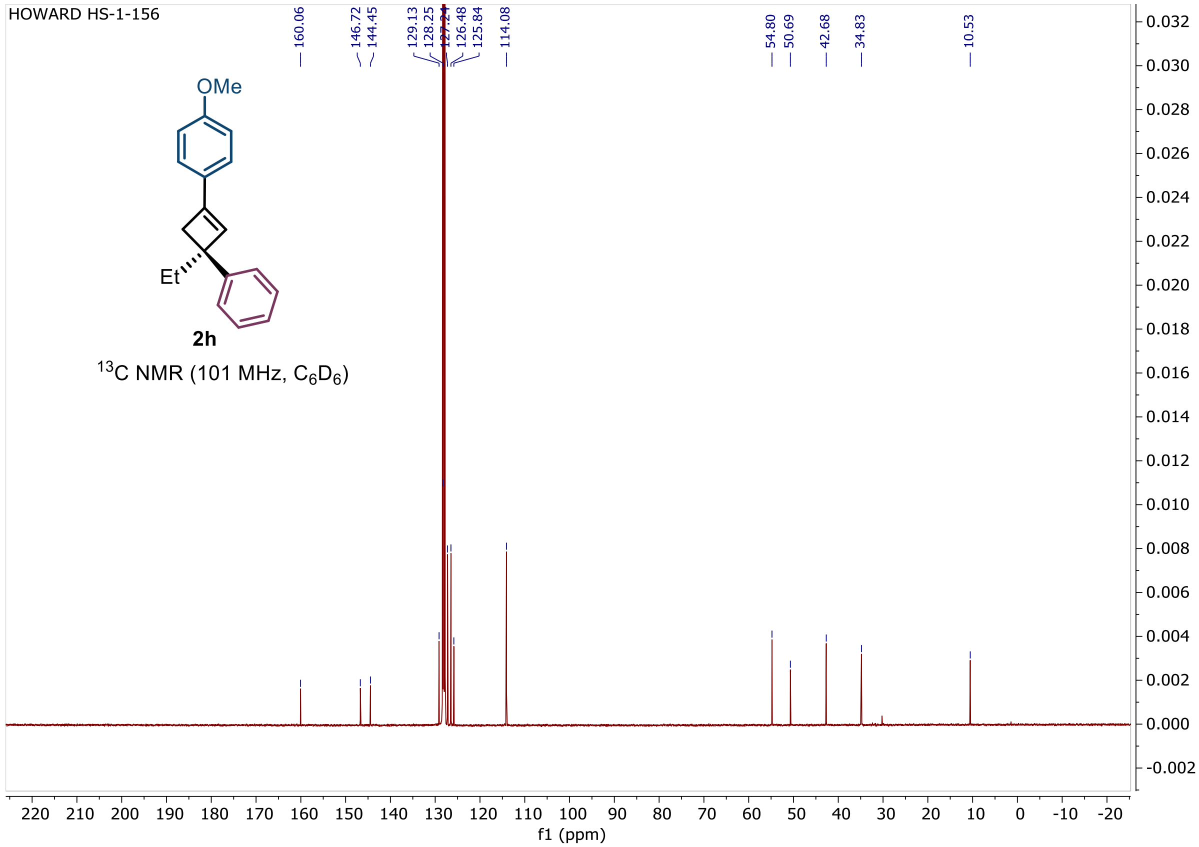
HOWARD HS-1-156
160.06
146.72
144.45
129.13
128.25
127.24
126.48
125.84
114.08
54.80
50.69
42.68
34.83
10.53
OMe
Et
2h
^{13}C NMR (101 MHz, C_6D_6)
f1 (ppm)

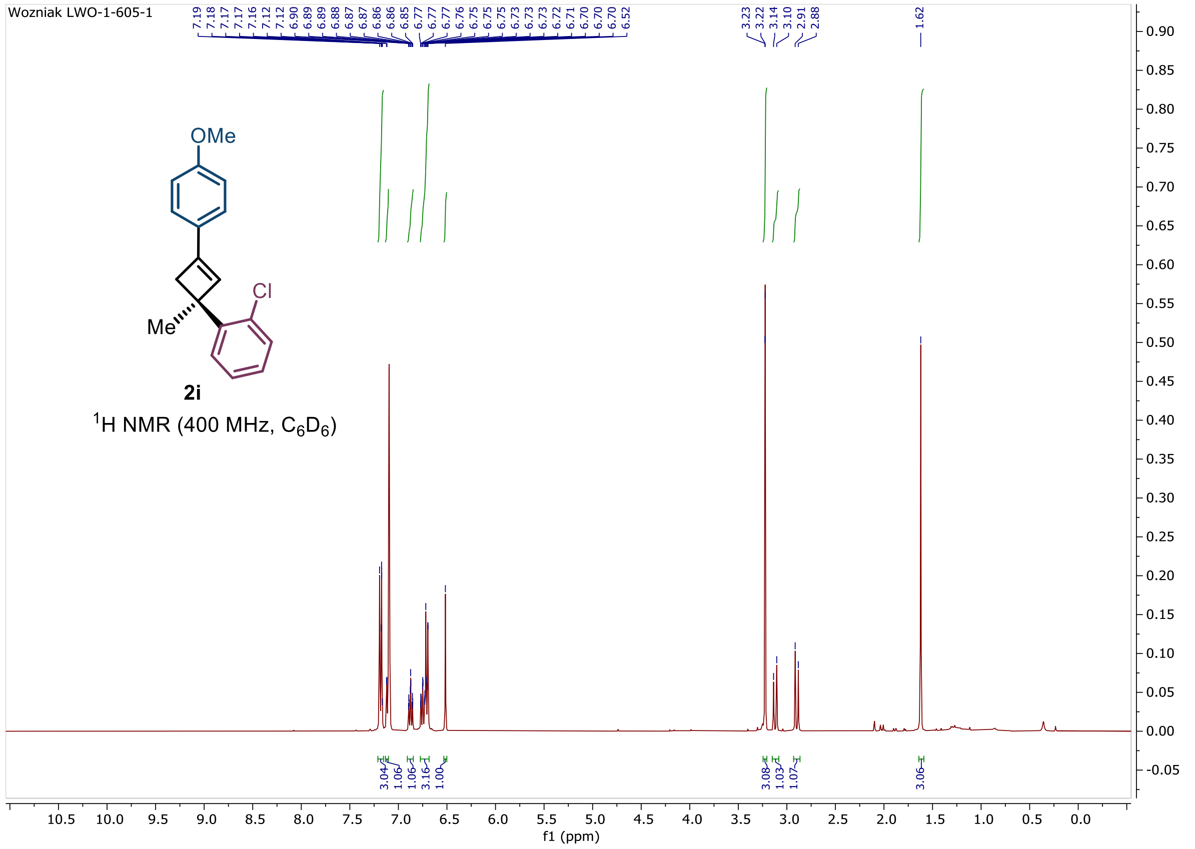
Wozniak LWO-1-605-1
7.19
7.18
7.17
7.16
7.12
7.12
6.90
6.89
6.89
6.88
6.87
6.87
6.86
6.85
6.77
6.77
6.77
6.76
6.75
6.75
6.73
6.73
6.73
6.72
6.71
6.70
6.70
6.52
3.23
3.22
3.14
3.10
2.91
2.88
1.62
OMe
Cl
Me
2i
^{1}H NMR (400 MHz, C_6D_6)
3.04
1.06
1.06
3.16
1.00
3.08
1.03
1.07
3.06
f1 (ppm)

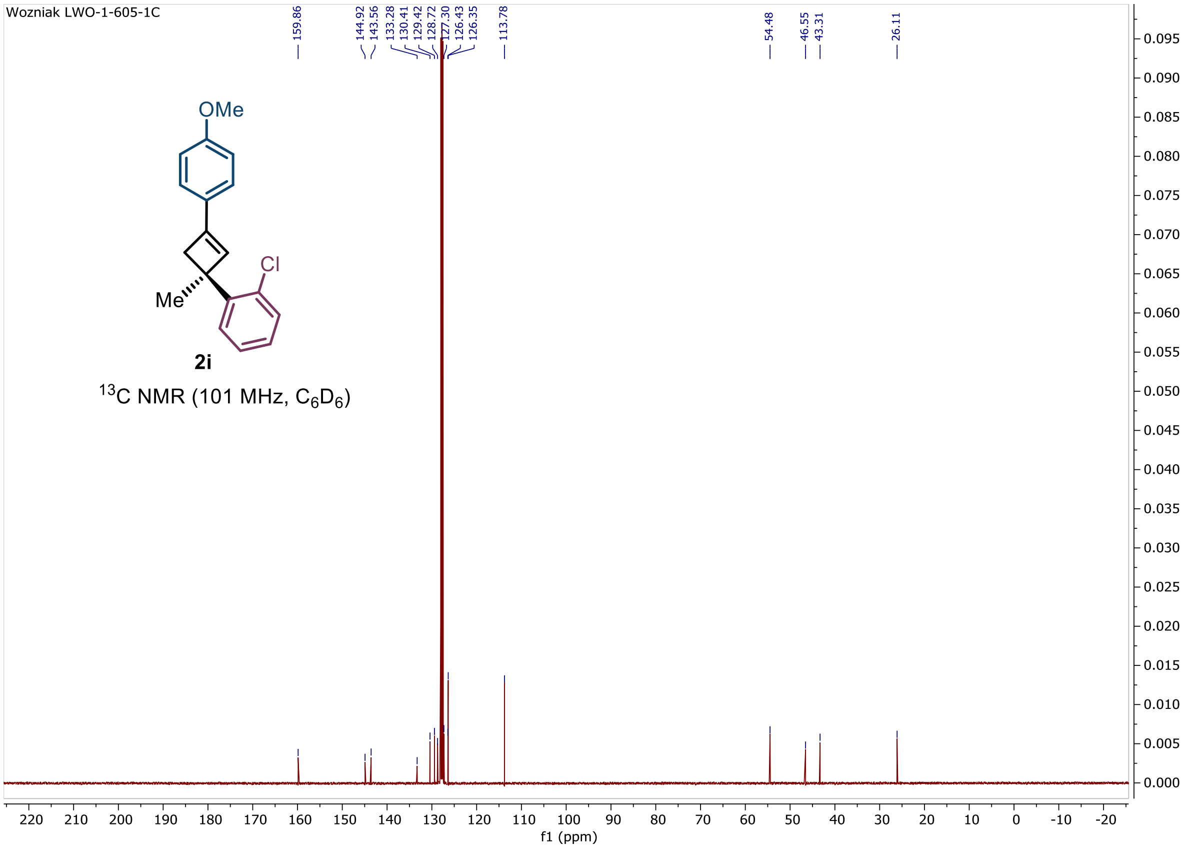
Wozniak LWO-1-605-1C
OMe
Cl
Me
2i
^{13}C NMR (101 MHz, C_6D_6)
f1 (ppm)

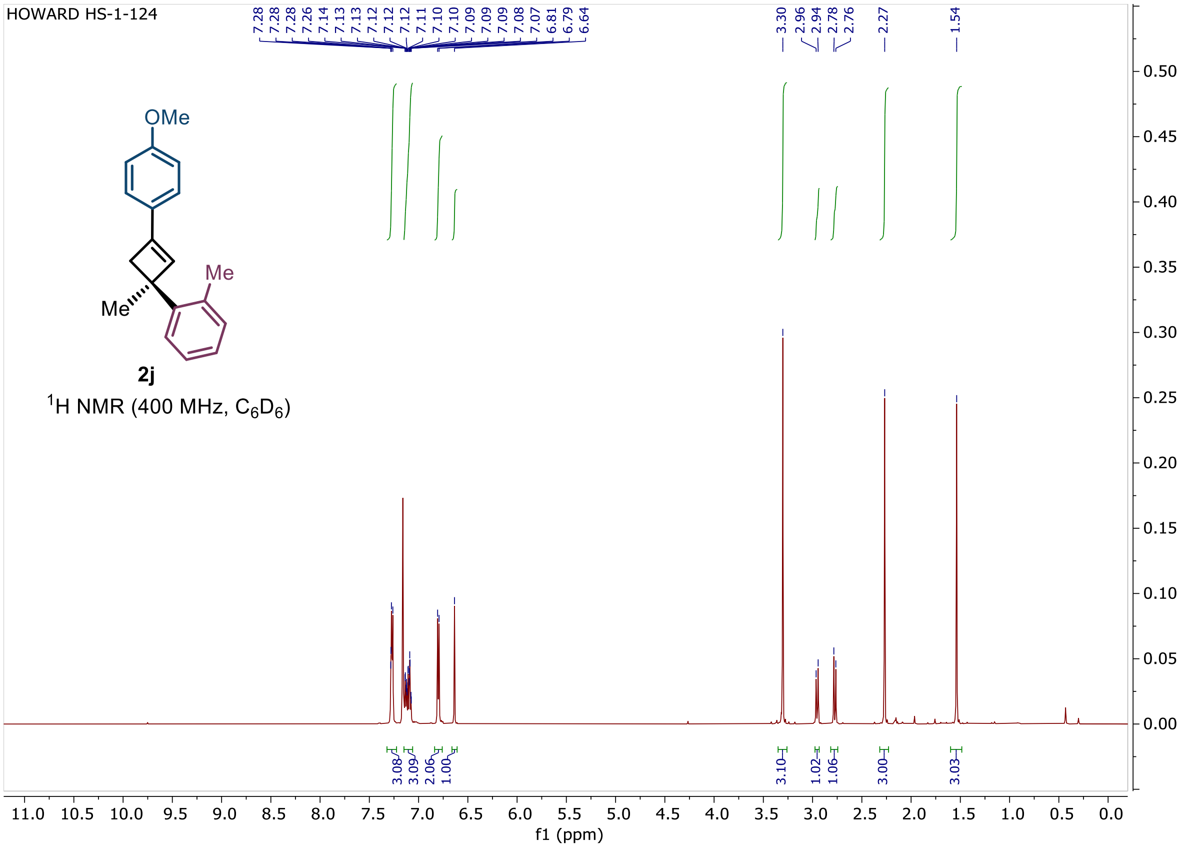
HOWARD HS-1-124
OMe
Me
Me
2j
^{1}H NMR (400 MHz, C_6D_6)
f1 (ppm)

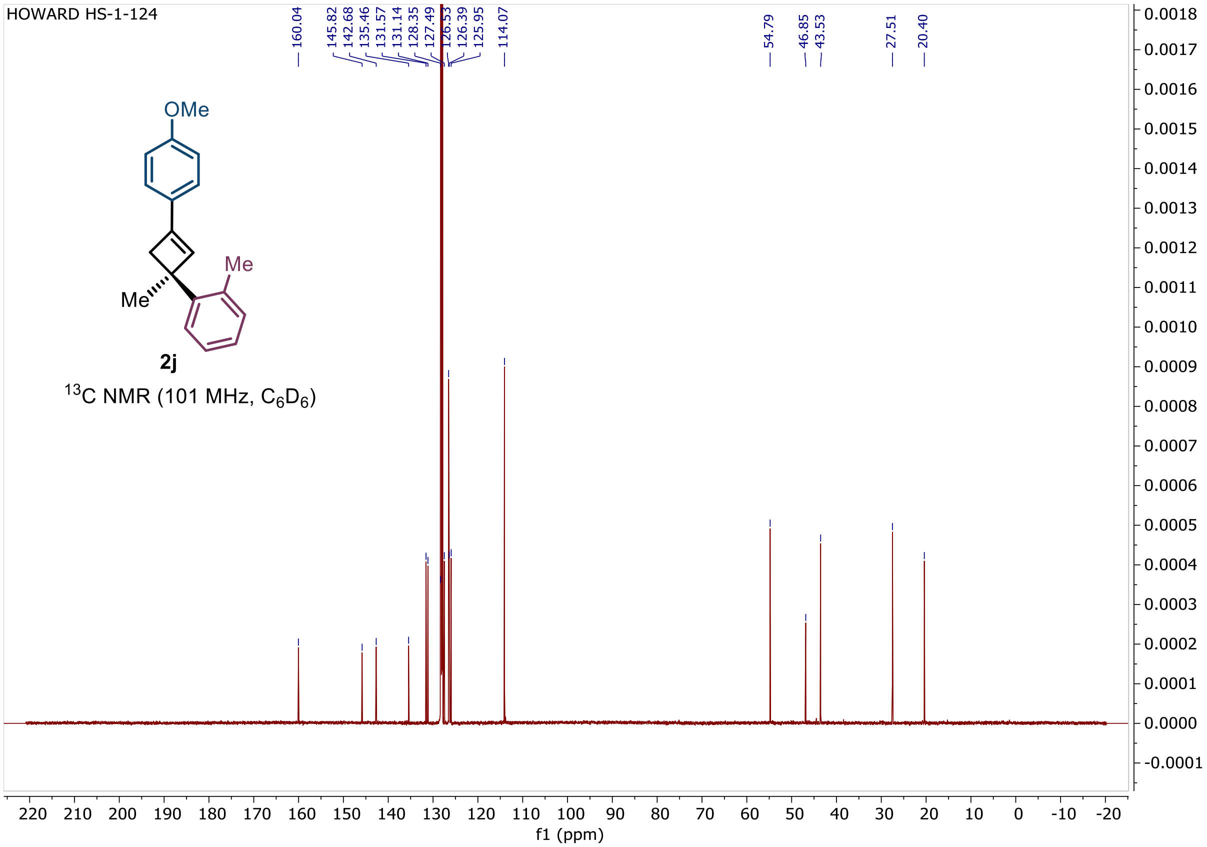
HOWARD HS-1-124
160.04
145.82
142.68
135.46
131.57
131.14
128.35
127.49
126.53
126.39
125.95
114.07
54.79
46.85
43.53
27.51
20.40
OMe
Me
Me
2j
^{13}C NMR (101 MHz, C_6D_6)
f1 (ppm)

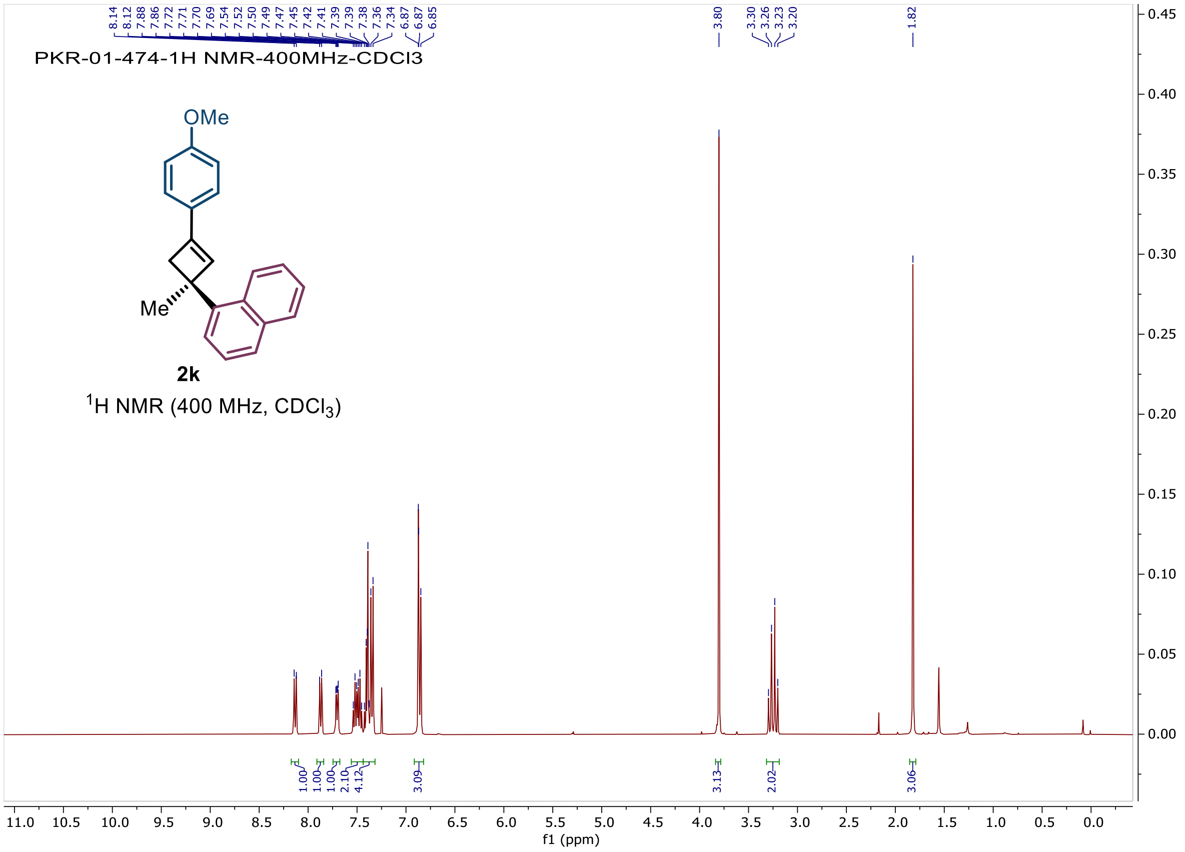
PKR-01-474-1H NMR-400MHz-CDCl3
8.14
8.12
7.88
7.86
7.72
7.71
7.70
7.69
7.54
7.52
7.50
7.49
7.47
7.45
7.42
7.41
7.39
7.39
7.38
7.36
7.34
6.87
6.87
6.85
3.80
3.30
3.26
3.23
3.20
1.82
OMe
Me
2k
^{1}H NMR (400 MHz, $CDCl_3$)
1.00
1.00
1.00
2.10
4.12
3.09
3.13
2.02
3.06
f1 (ppm)

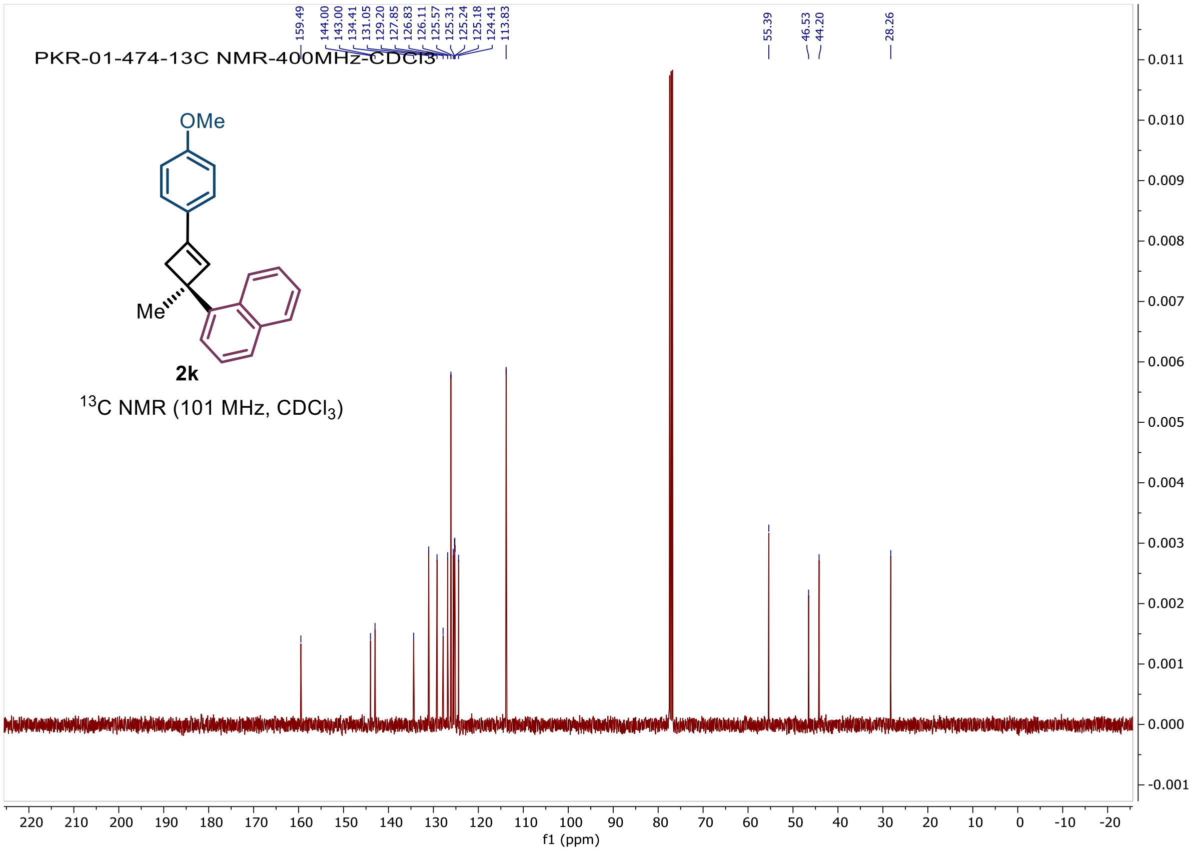
PKR-01-474-13C NMR-400MHz-CDCl3
159.49
144.00
143.00
134.41
131.05
129.20
127.85
126.83
126.11
125.57
125.31
125.24
125.18
124.41
113.83
55.39
46.53
44.20
28.26
OMe
Me
2k
13C NMR (101 MHz, CDCl3)
f1 (ppm)

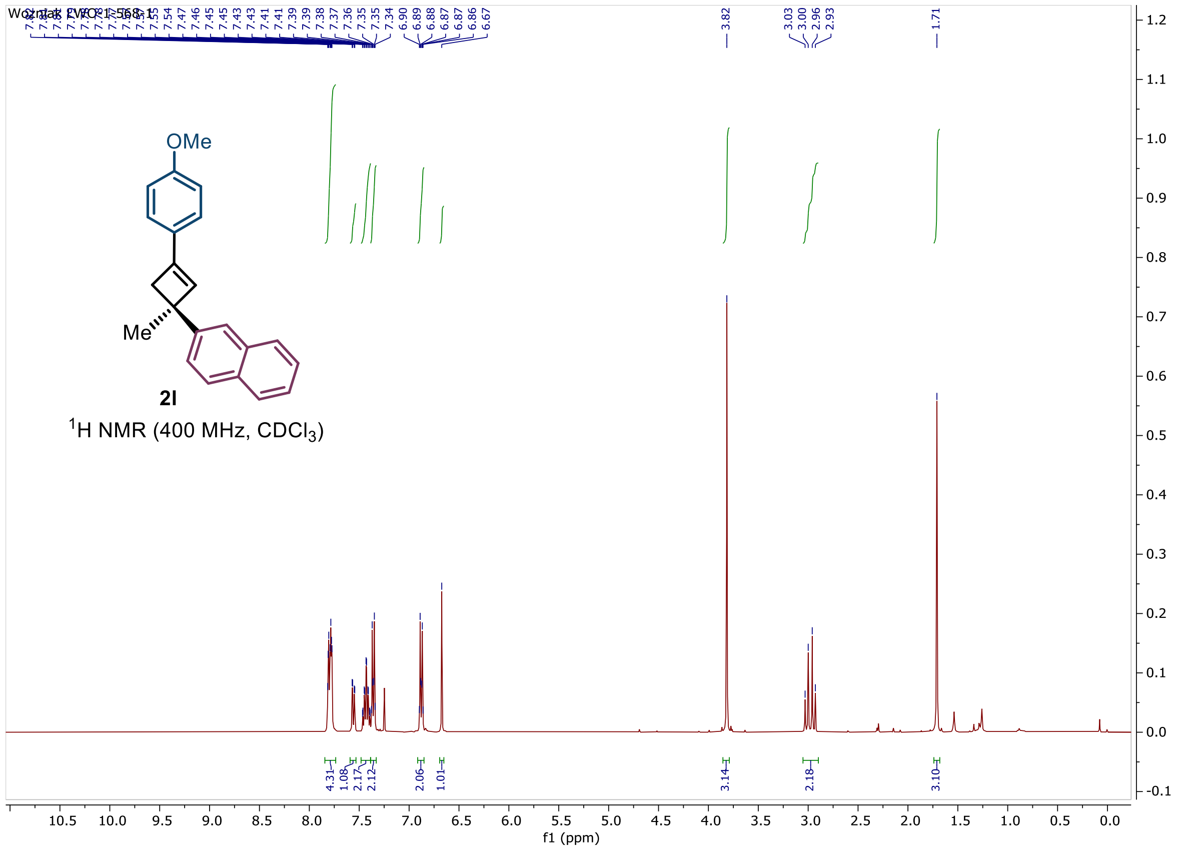
3.82
3.03
3.00
2.96
2.93
1.71
OMe
Me
2l
1H NMR (400 MHz, CDCl3)
4.31
1.08
2.17
2.12
2.06
1.01
3.14
2.18
3.10
f1 (ppm)

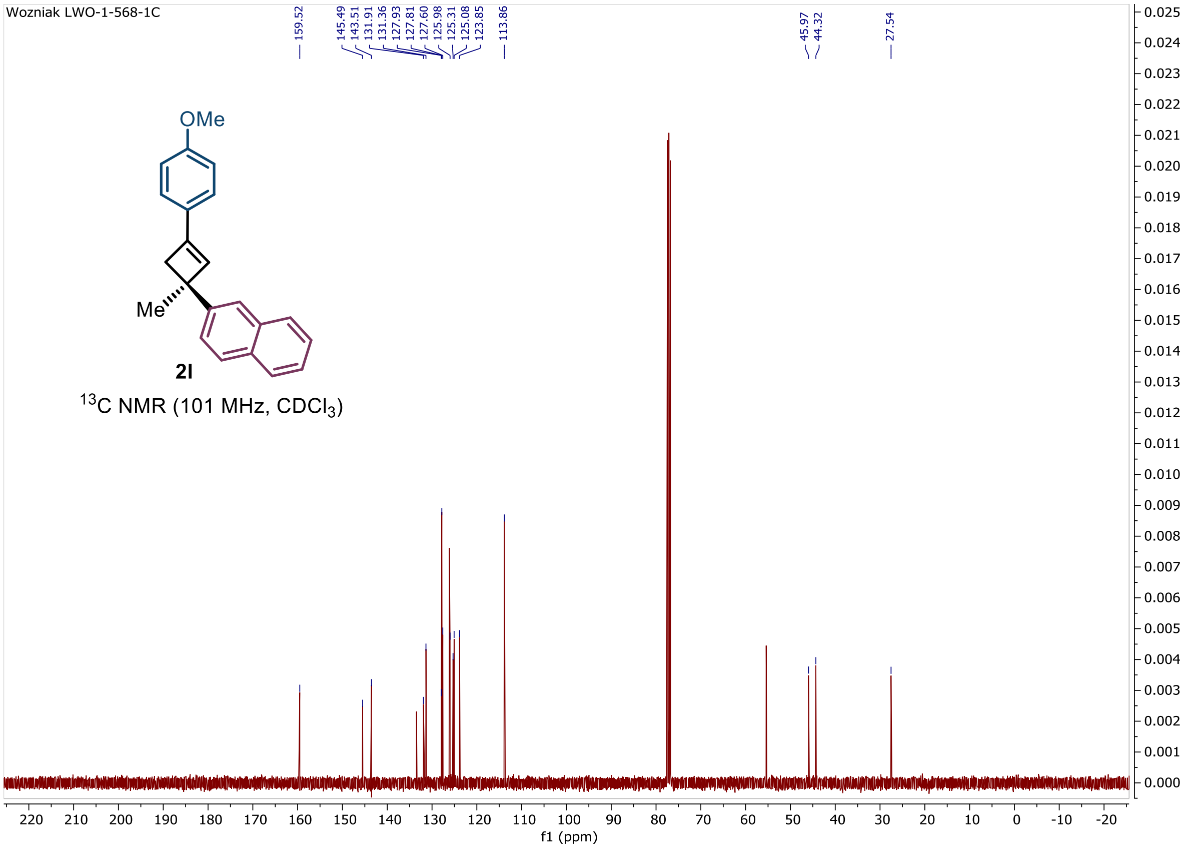
Wozniak LWO-1-568-1C
159.52
145.49
143.51
131.91
131.36
127.93
127.81
127.60
125.98
125.31
125.08
123.85
113.86
45.97
44.32
27.54
OMe
Me
2l
^{13}C NMR (101 MHz, CDCl$_3$)
f1 (ppm)

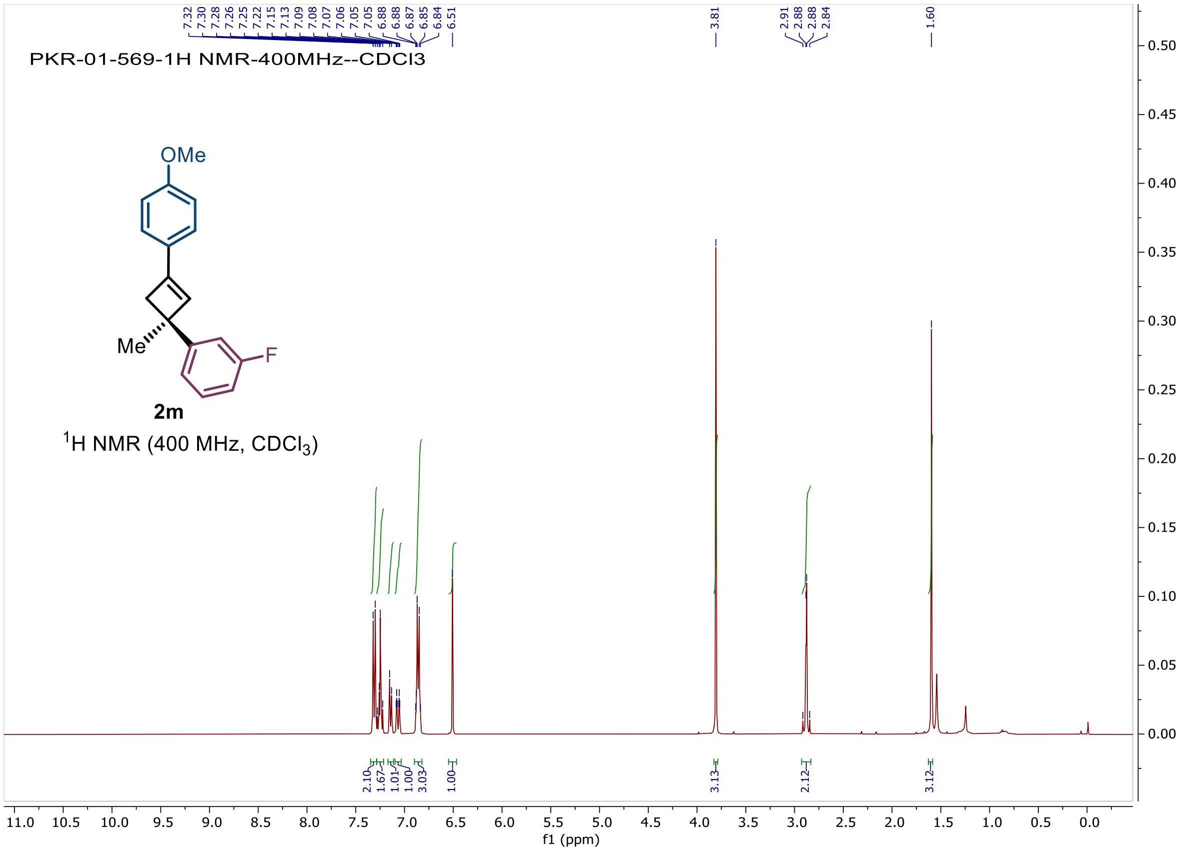
PKR-01-569-1H NMR-400MHz--CDCl3
7.32
7.30
7.28
7.26
7.25
7.22
7.15
7.13
7.09
7.08
7.07
7.06
7.05
7.05
6.88
6.88
6.87
6.85
6.84
6.51
3.81
2.91
2.88
2.88
2.84
1.60
OMe
Me
F
2m
^{1}H NMR (400 MHz, CDCl$_3$)
2.10
1.67
1.01
1.00
3.03
1.00
3.13
2.12
3.12
f1 (ppm)

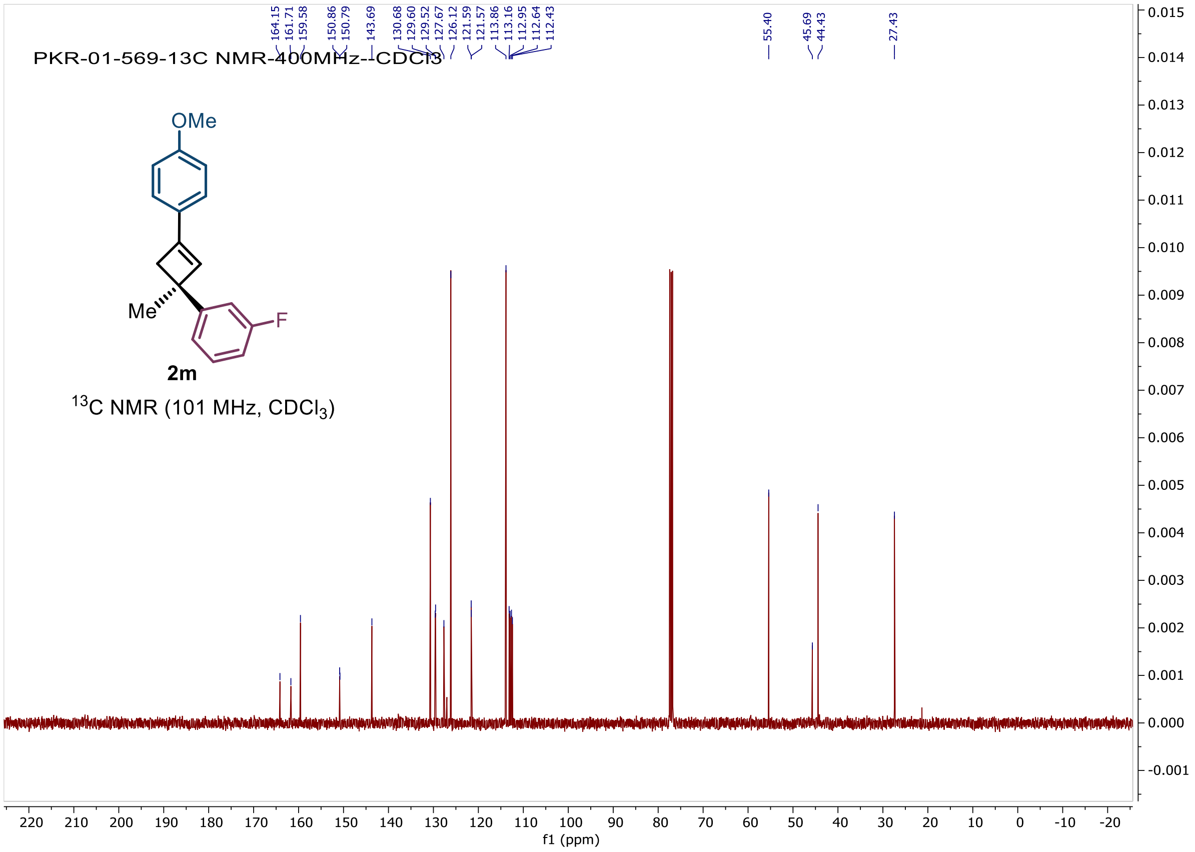
PKR-01-569-13C NMR-400MHz--CDCl3
OMe
Me
F
2m
^{13}C NMR (101 MHz, $CDCl_3$)
164.15
161.71
159.58
150.86
150.79
143.69
130.68
129.60
129.52
127.67
126.12
121.59
121.57
113.86
113.16
112.95
112.64
112.43
55.40
45.69
44.43
27.43
f1 (ppm)

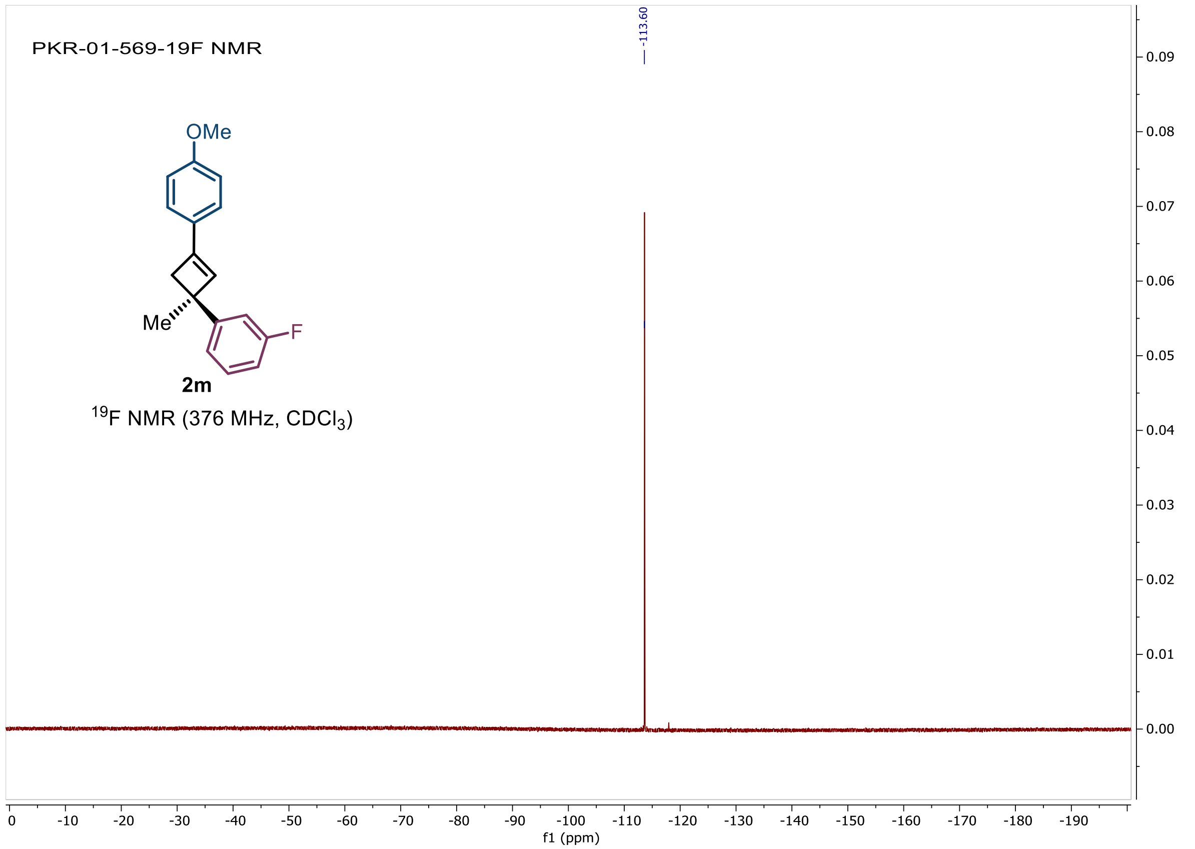
PKR-01-569-19F NMR
OMe
Me
F
2m
^{19}F NMR (376 MHz, $CDCl_3$)
-113.60
f1 (ppm)

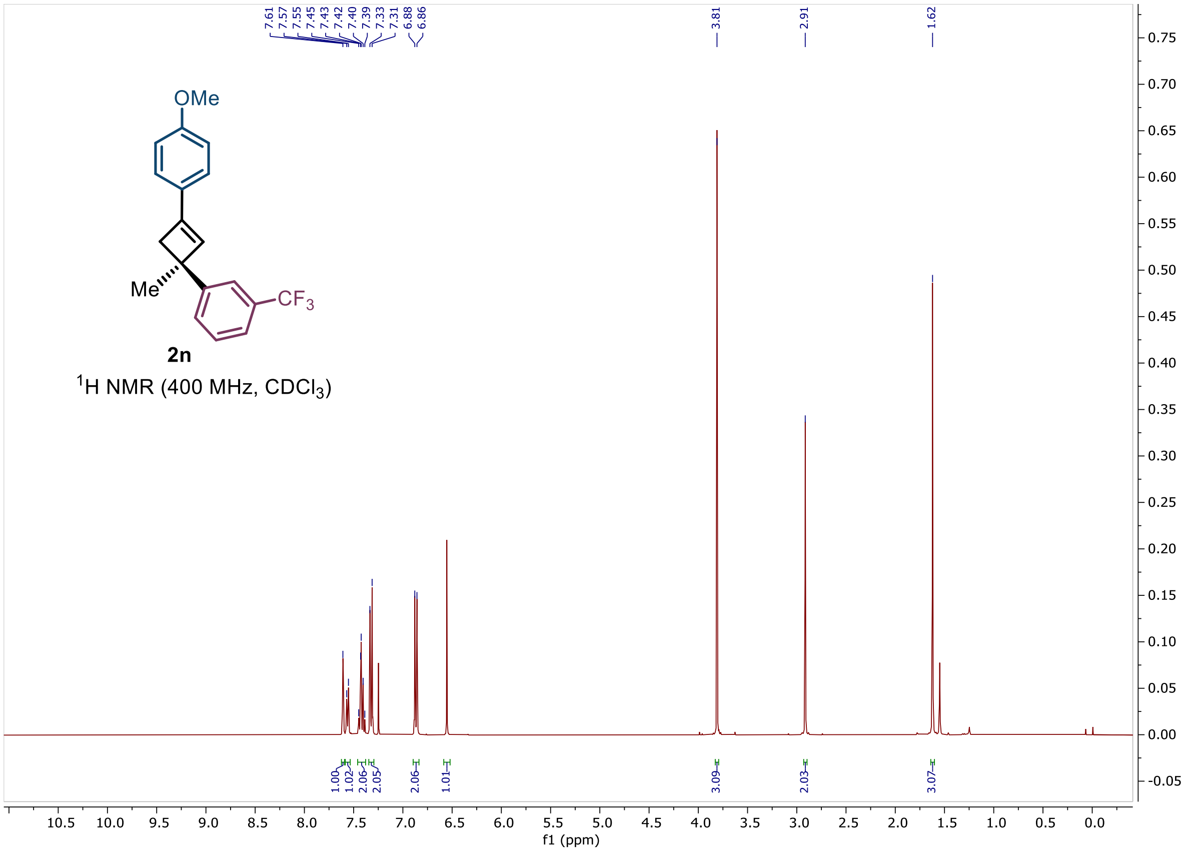
7.61
7.57
7.55
7.45
7.43
7.42
7.40
7.39
7.33
7.31
6.88
6.86
3.81
2.91
1.62
OMe
Me
CF3
2n
1H NMR (400 MHz, CDCl3)
1.00
1.02
2.06
2.05
2.06
1.01
3.09
2.03
3.07
f1 (ppm)

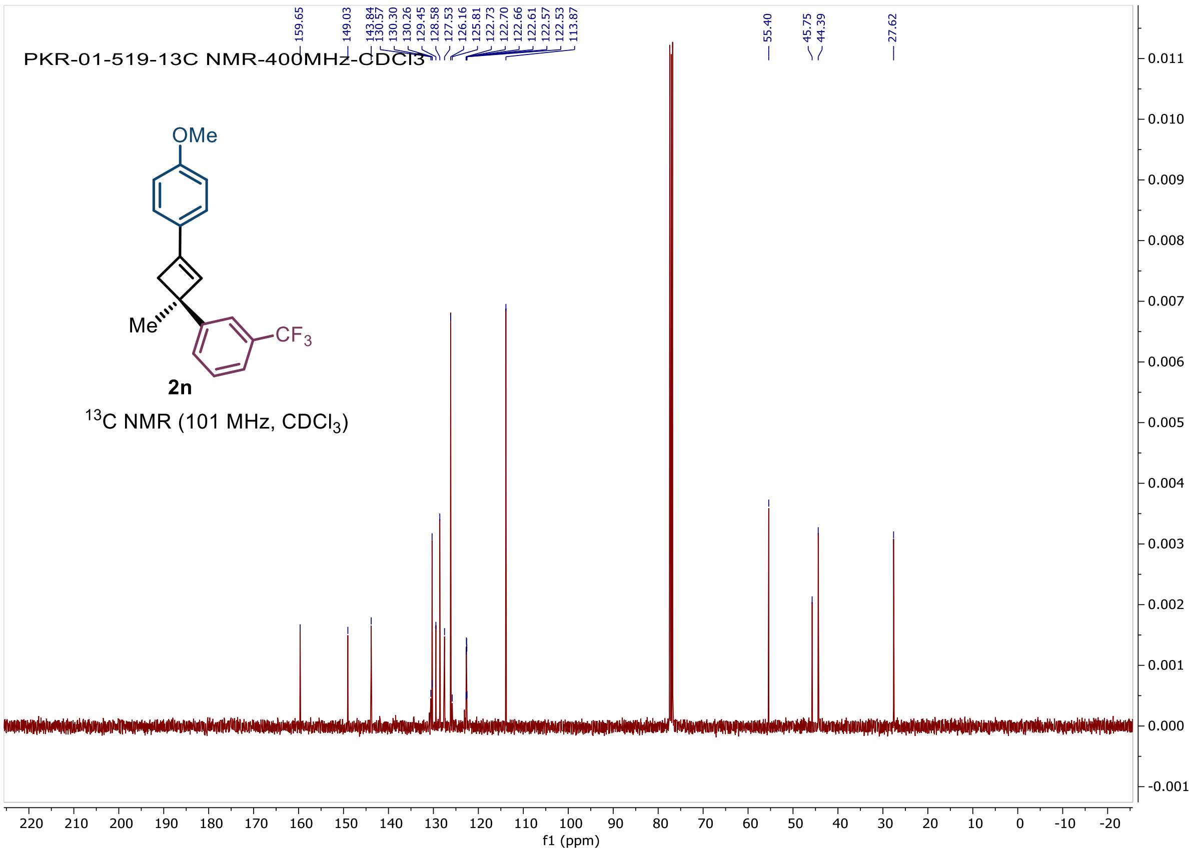
159.65
149.03
143.84
130.57
130.30
130.26
129.45
128.58
127.53
126.16
125.81
122.73
122.70
122.66
122.61
122.57
122.53
113.87
55.40
45.75
44.39
27.62
PKR-01-519-13C NMR-400MHz-CDCl3
OMe
Me
CF3
2n
13C NMR (101 MHz, CDCl3)
f1 (ppm)

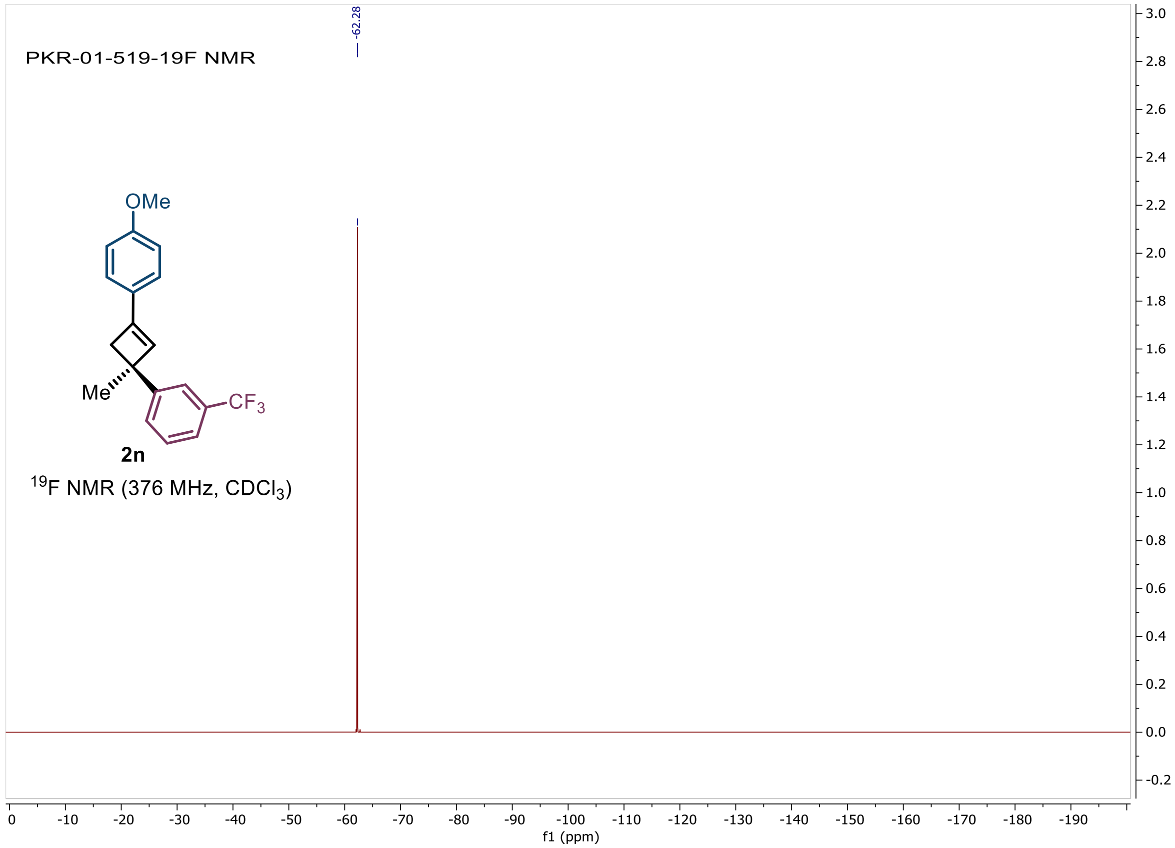
PKR-01-519-19F NMR
-62.28
OMe
Me
CF3
2n
19F NMR (376 MHz, CDCl3)
f1 (ppm)

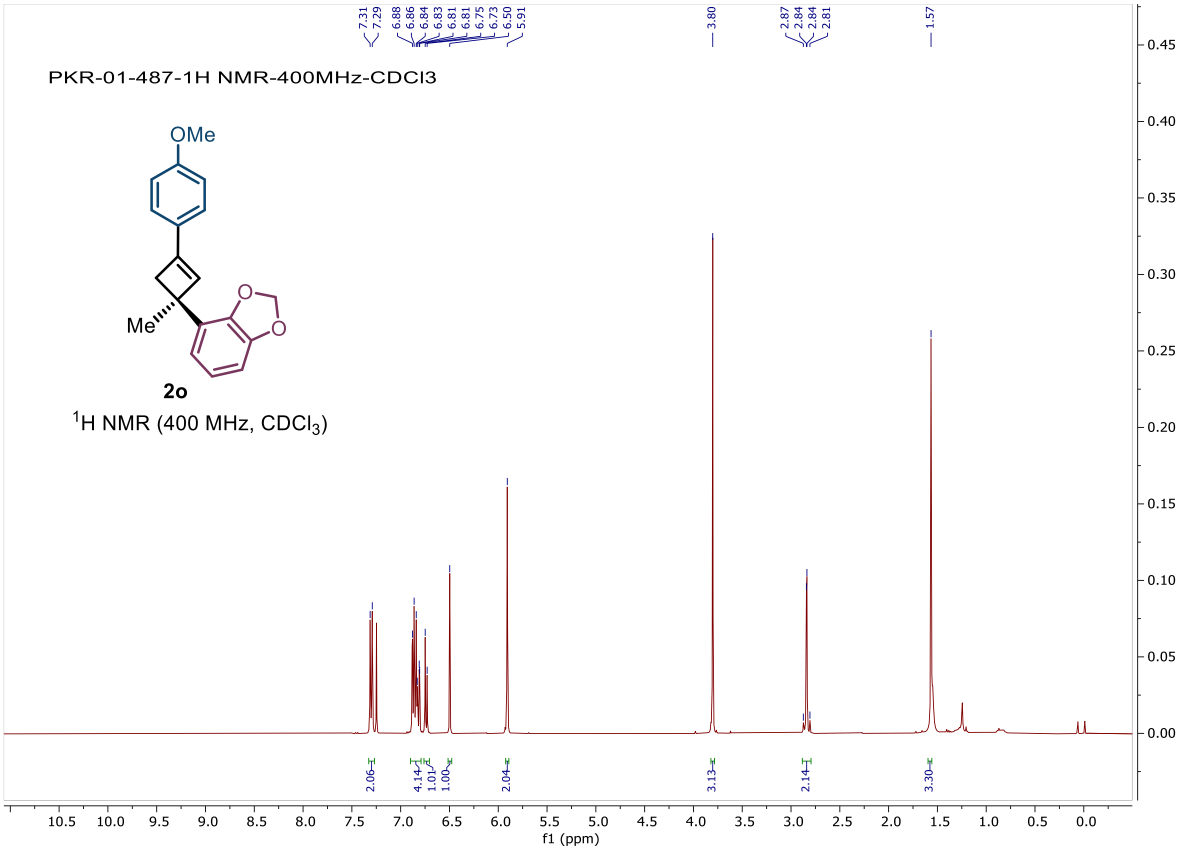
PKR-01-487-1H NMR-400MHz-CDCl3
7.31
7.29
6.88
6.86
6.84
6.83
6.81
6.81
6.75
6.73
6.50
5.91
3.80
2.87
2.84
2.84
2.81
1.57
OMe
Me
O
O
2o
1H NMR (400 MHz, CDCl3)
2.06
4.14
1.01
1.00
2.04
3.13
2.14
3.30
f1 (ppm)

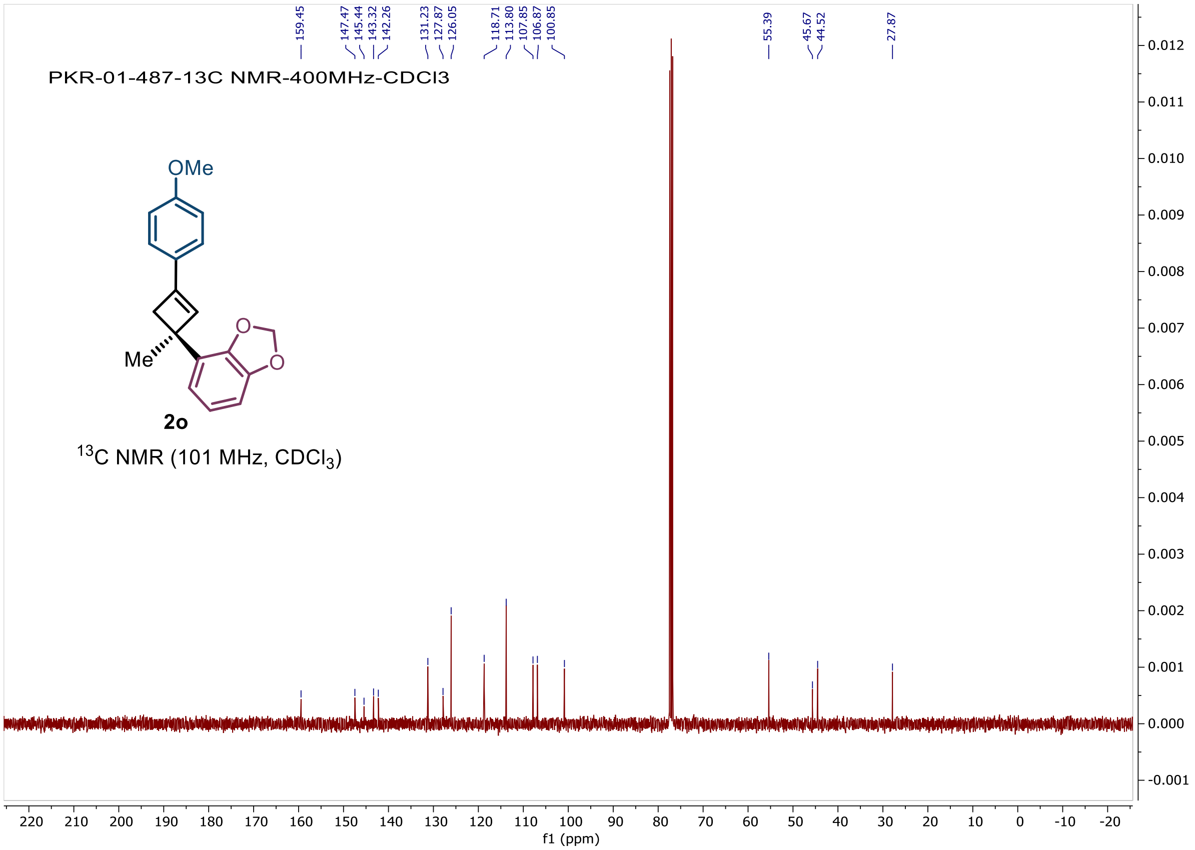

2o

^{13}C NMR (101 MHz, $CDCl_3$)

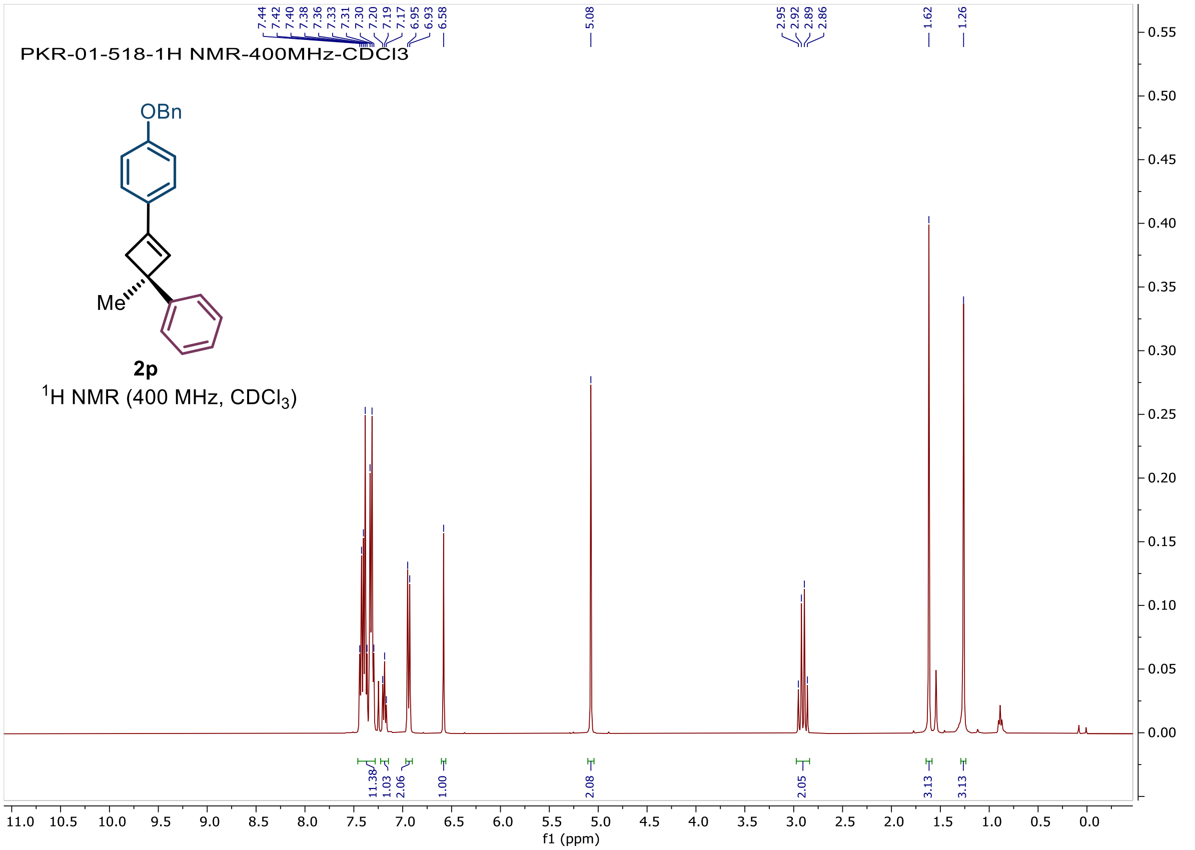

2p

^{1}H NMR (400 MHz, $CDCl_3$)

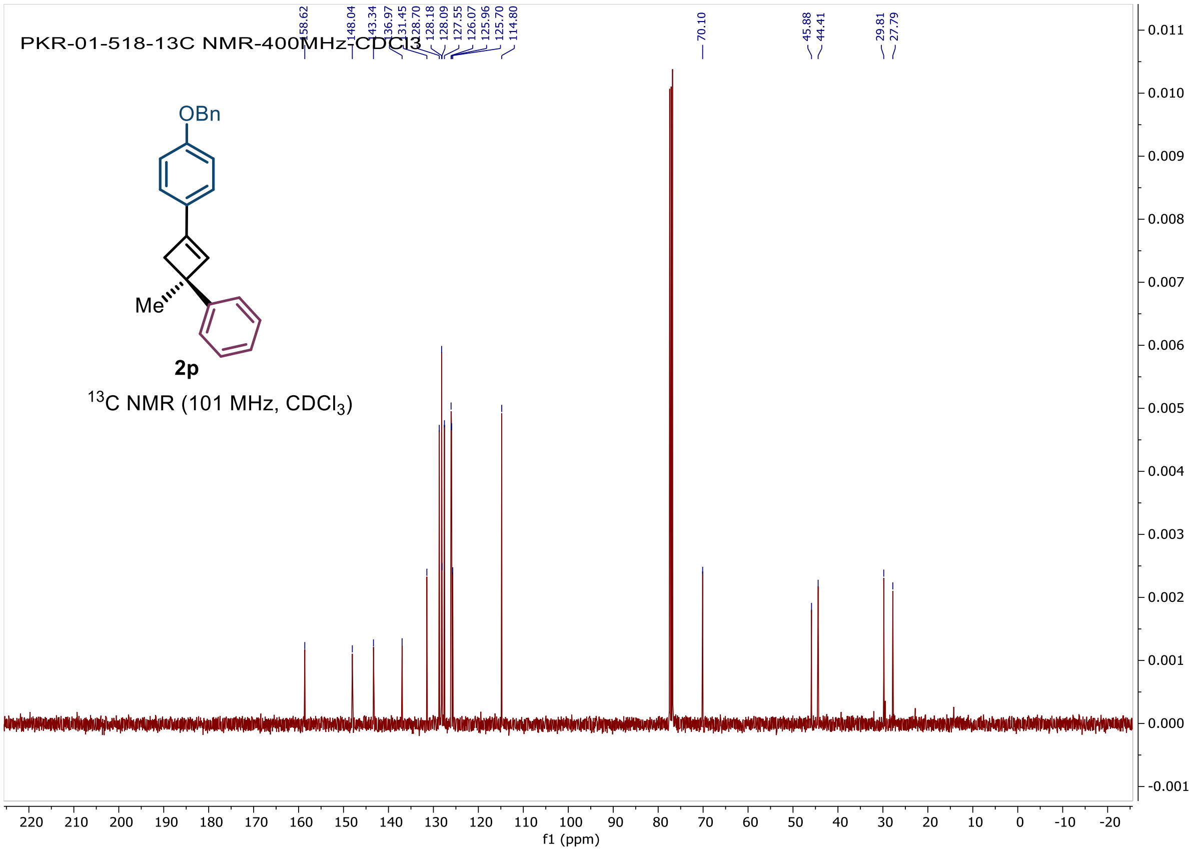

PKR-01-518-13C NMR-400MHz-CDCl3
158.62 148.04 143.34 136.97 131.45 128.70 128.18 128.09 127.55 126.07 125.96 125.70 114.80
70.10
45.88 44.41
29.81 27.79
OBn
Me
2p
13C NMR (101 MHz, CDCl3)
f1 (ppm)

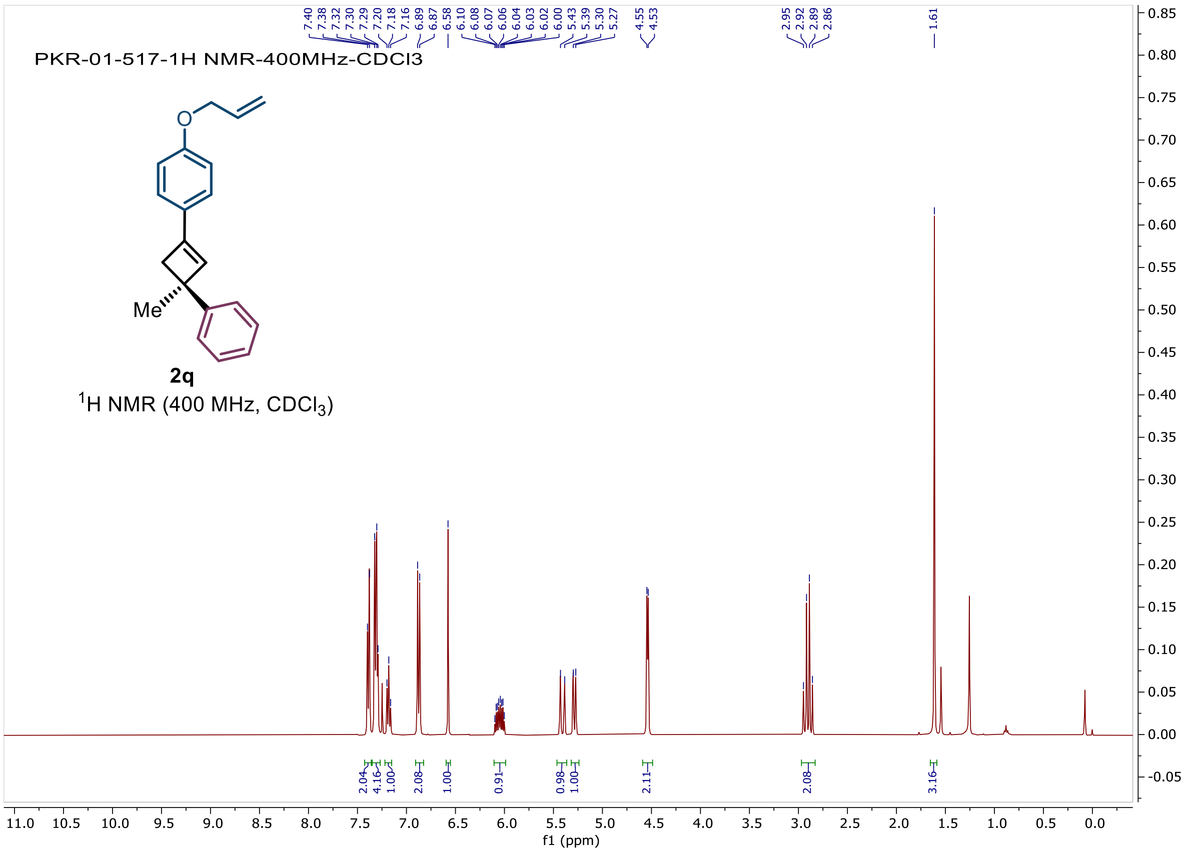

PKR-01-517-1H NMR-400MHz-CDCl3
7.40 7.38 7.32 7.30 7.29 7.20 7.18 7.16 6.89 6.87 6.58 6.10 6.08 6.07 6.06 6.04 6.03 6.02 6.00 5.43 5.39 5.30 5.27 4.55 4.53
2.95 2.92 2.89 2.86
1.61
O
Me
2q
1H NMR (400 MHz, CDCl3)
2.04 4.16 1.00 2.08 1.00 0.91 0.98 1.00 2.11 2.08 3.16
f1 (ppm)

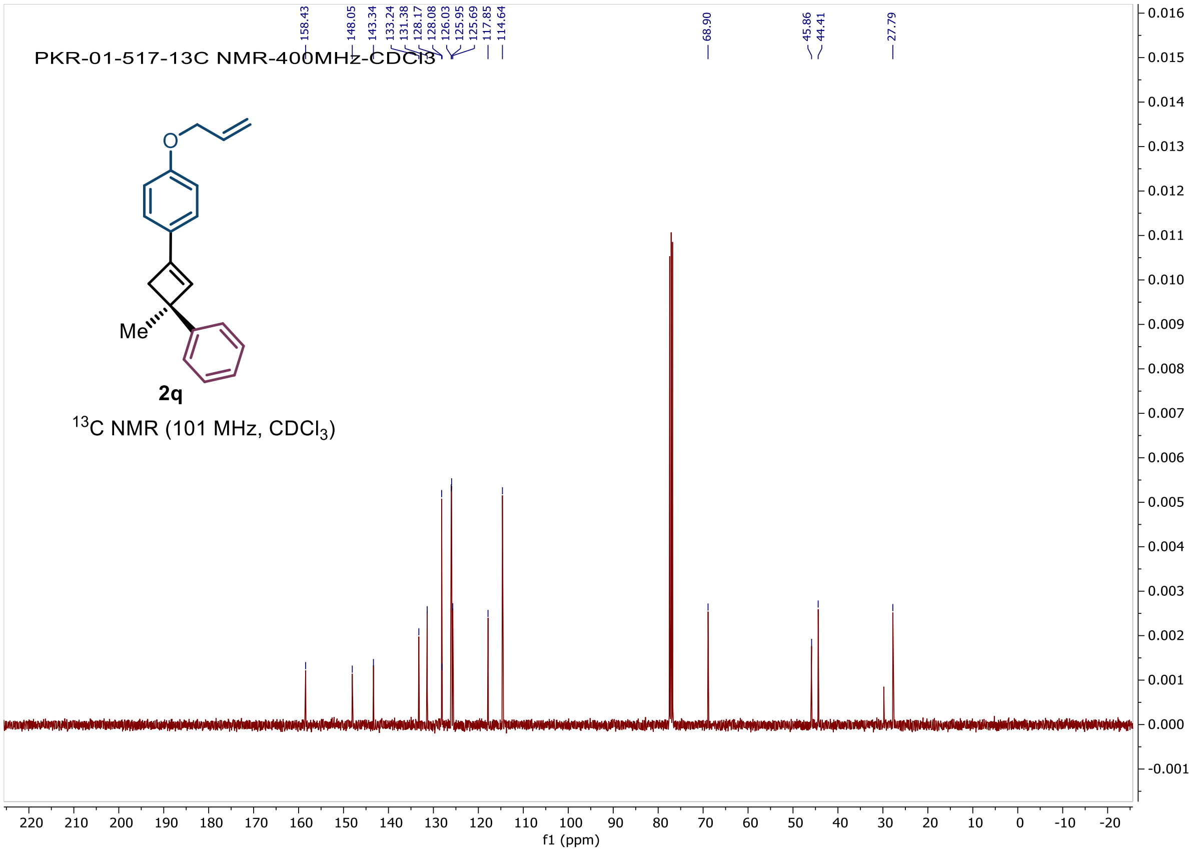
PKR-01-517-13C NMR-400MHz-CDCl3
O
Me
2q
^{13}C NMR (101 MHz, CDCl$_3$)
158.43
148.05
143.34
133.24
131.38
128.17
128.08
126.03
125.95
125.69
117.85
114.64
68.90
45.86
44.41
27.79
f1 (ppm)

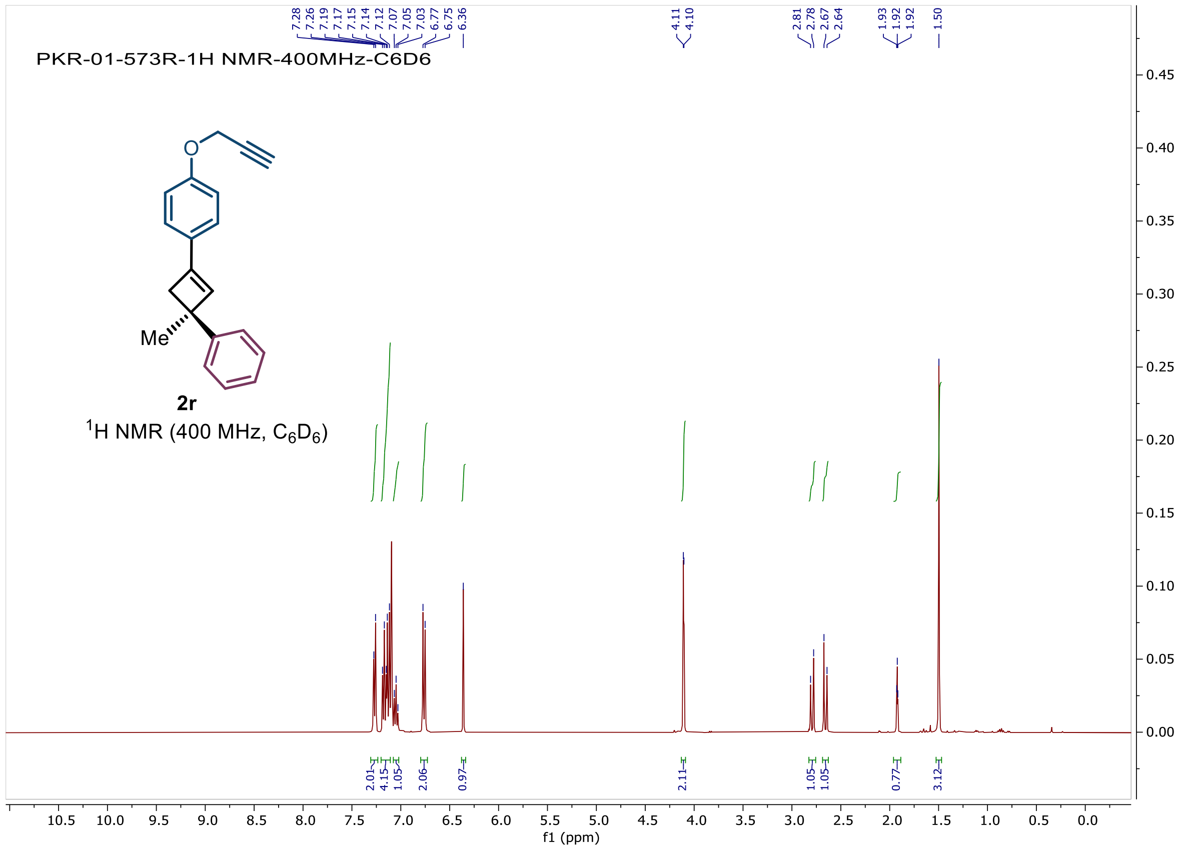
PKR-01-573R-1H NMR-400MHz-C6D6
O
Me
2r
^{1}H NMR (400 MHz, C$_6$D$_6$)
7.28
7.26
7.19
7.17
7.15
7.14
7.12
7.07
7.05
7.03
6.77
6.75
6.36
4.11
4.10
2.81
2.78
2.67
2.64
1.93
1.92
1.92
1.50
2.01
4.15
1.05
2.06
0.97
2.11
1.05
1.05
0.77
3.12
f1 (ppm)

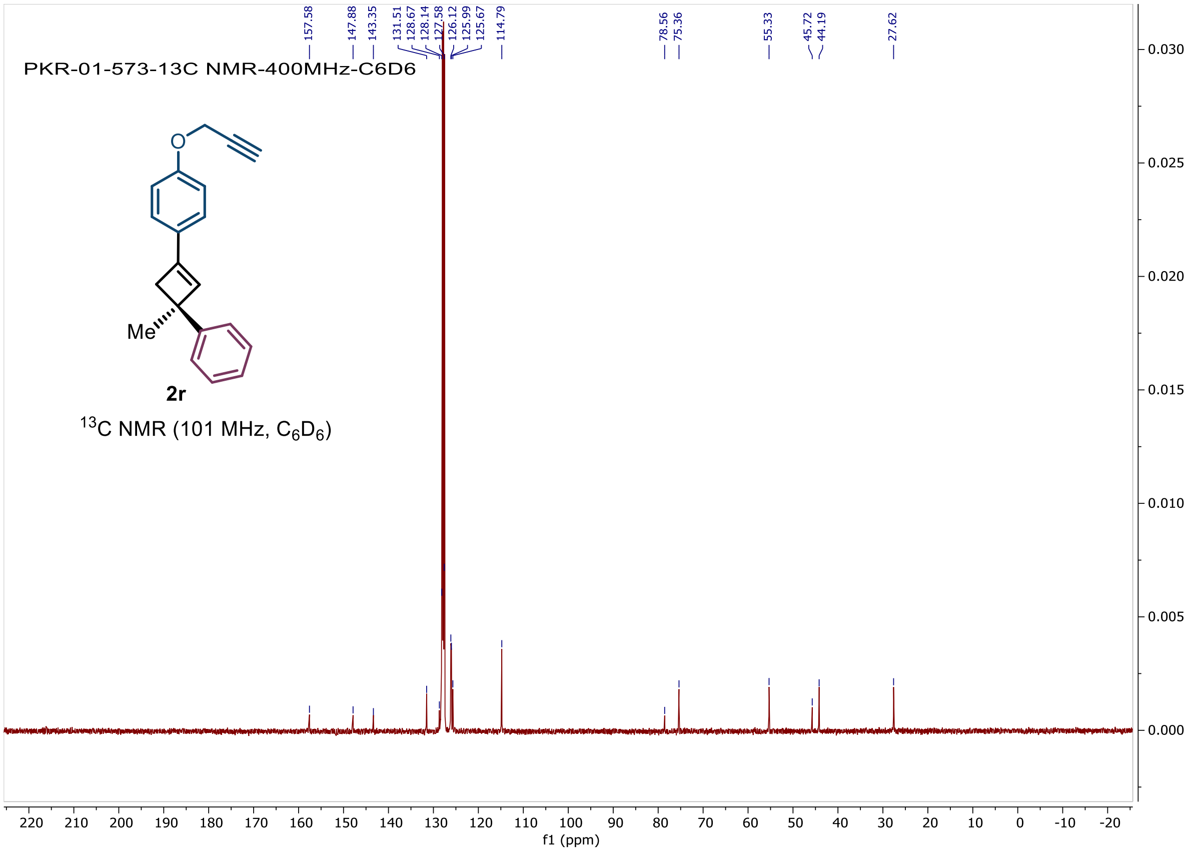
PKR-01-573-13C NMR-400MHz-C6D6
157.58
147.88
143.35
131.51
128.67
128.14
127.58
126.12
125.99
125.67
114.79
78.56
75.36
55.33
45.72
44.19
27.62
O
Me
2r
^{13}C NMR (101 MHz, C_6D_6)
f1 (ppm)

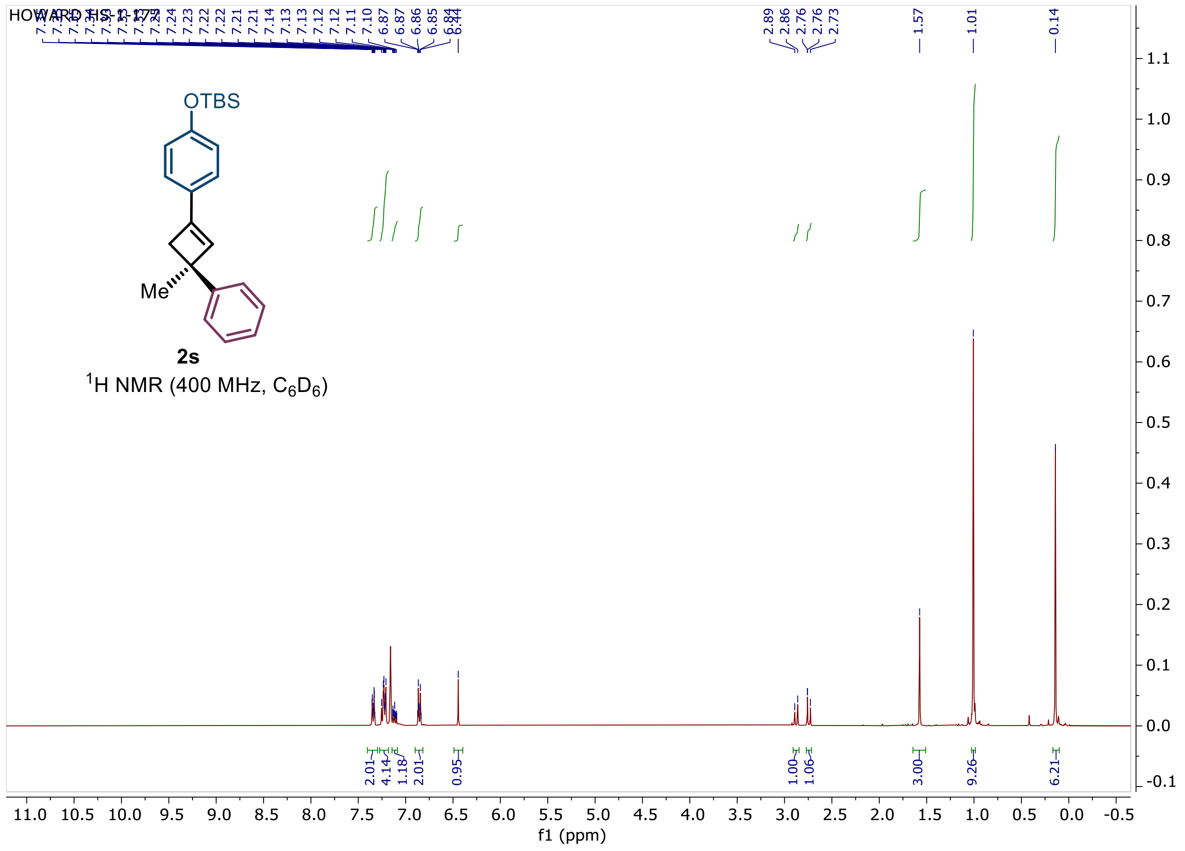
2.89
2.86
2.76
2.76
2.73
1.57
1.01
0.14
OTBS
Me
2s
^{1}H NMR (400 MHz, C_6D_6)
2.01
4.14
1.18
2.01
0.95
1.00
1.06
3.00
9.26
6.21
f1 (ppm)

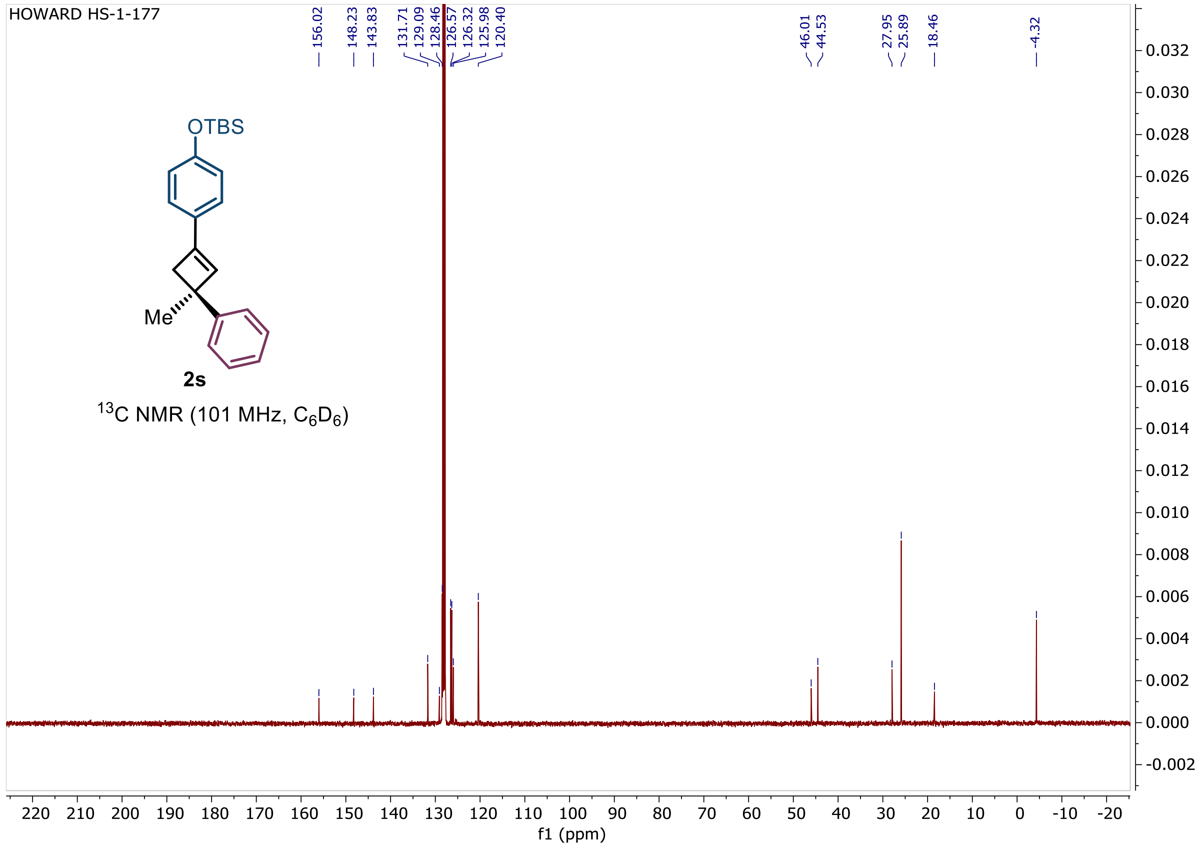
HOWARD HS-1-177
156.02
148.23
143.83
131.71
129.09
128.46
126.57
126.32
125.98
120.40
46.01
44.53
27.95
25.89
18.46
-4.32
OTBS
Me
2s
^{13}C NMR (101 MHz, C_6D_6)
f1 (ppm)

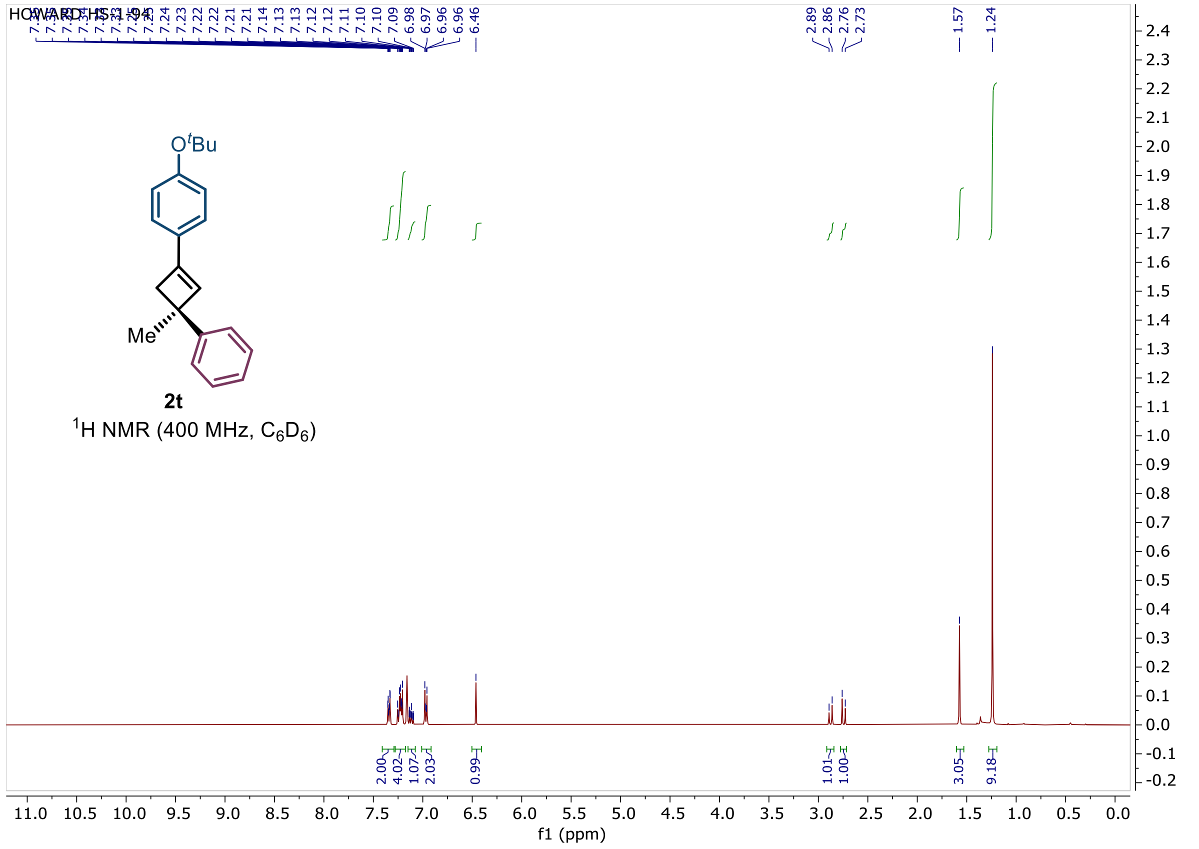
7.24
7.23
7.22
7.22
7.21
7.21
7.14
7.13
7.13
7.12
7.12
7.11
7.10
7.10
7.09
6.98
6.97
6.96
6.96
6.46
2.89
2.86
2.76
2.73
1.57
1.24
O^{t}Bu
Me
2t
^1H NMR (400 MHz, C_6D_6)
2.00
4.02
1.07
2.03
0.99
1.01
1.00
3.05
9.18
f1 (ppm)

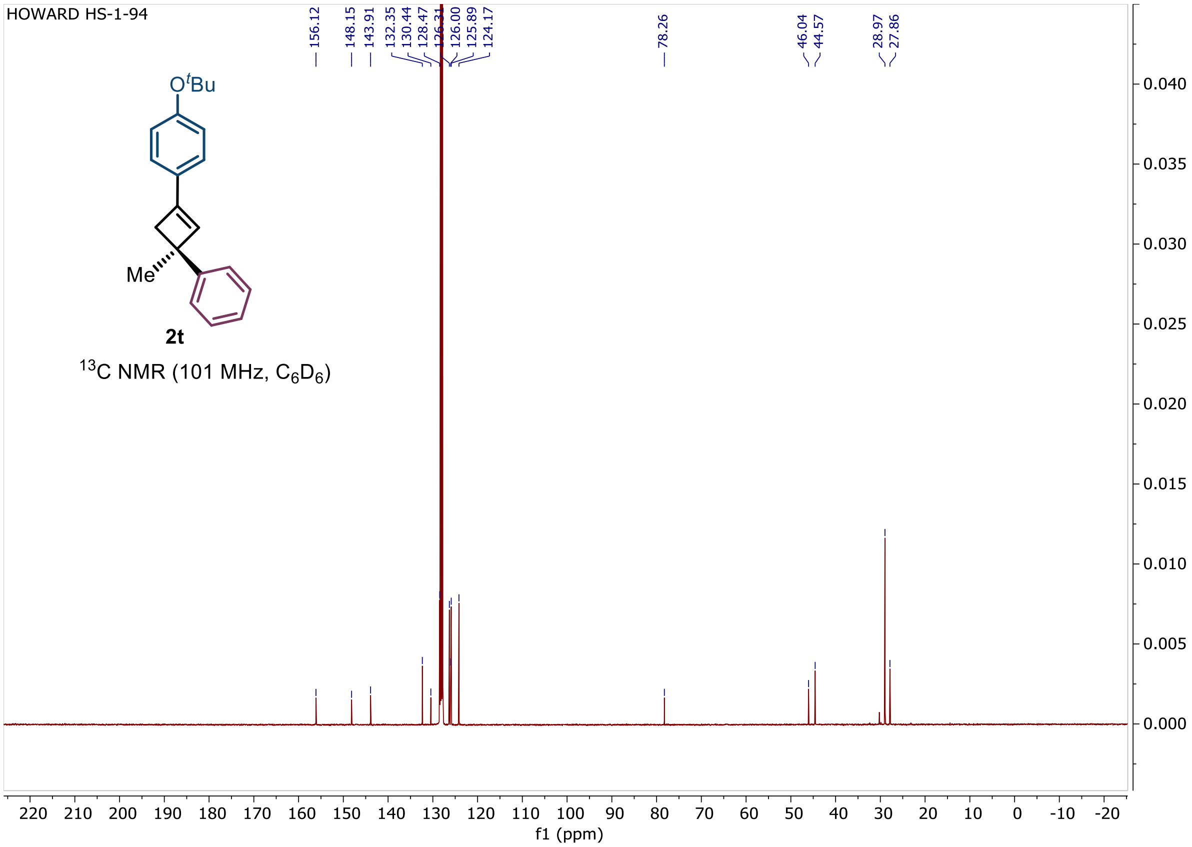
HOWARD HS-1-94
156.12
148.15
143.91
132.35
130.44
128.47
126.31
126.00
125.89
124.17
78.26
46.04
44.57
28.97
27.86
O^tBu
Me
2t
13C NMR (101 MHz, C6D6)
f1 (ppm)

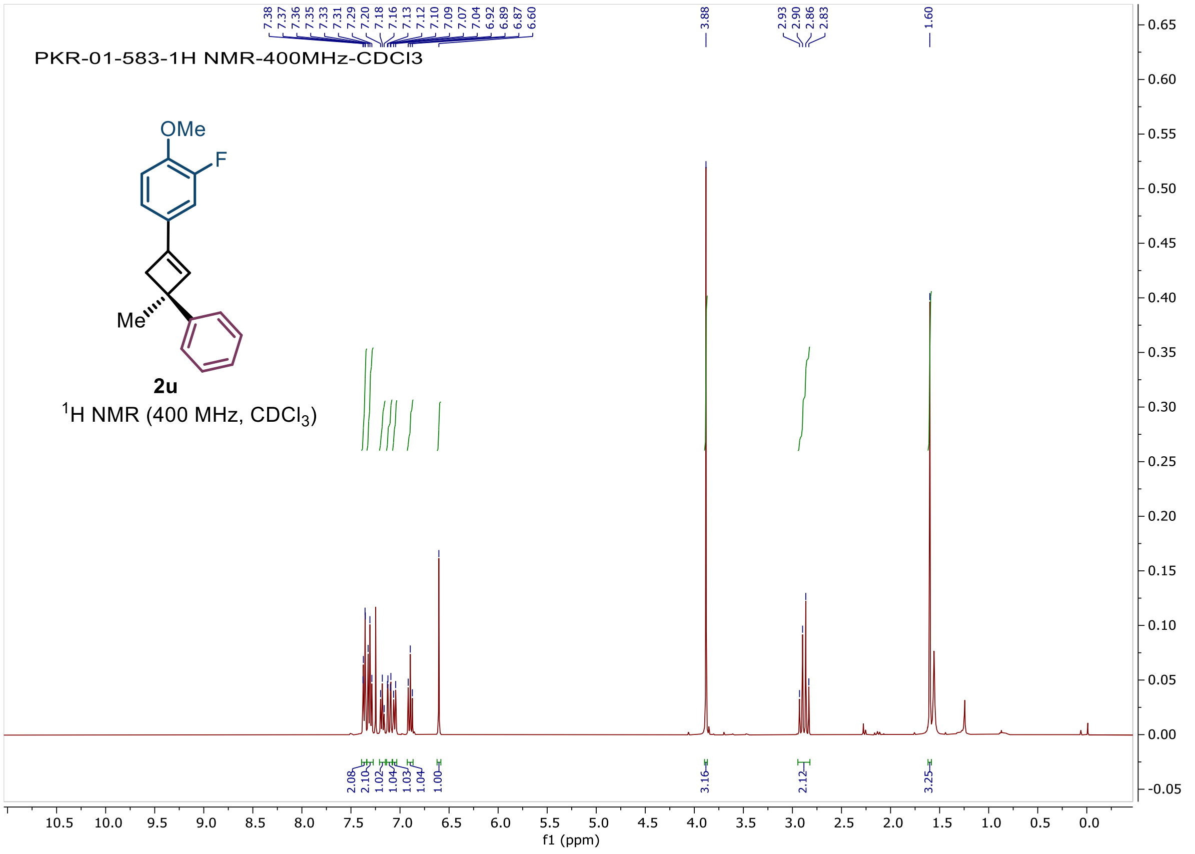
PKR-01-583-1H NMR-400MHz-CDCl3
OMe
F
Me
2u
1H NMR (400 MHz, CDCl3)
3.88
2.93
2.90
2.86
2.83
1.60
f1 (ppm)

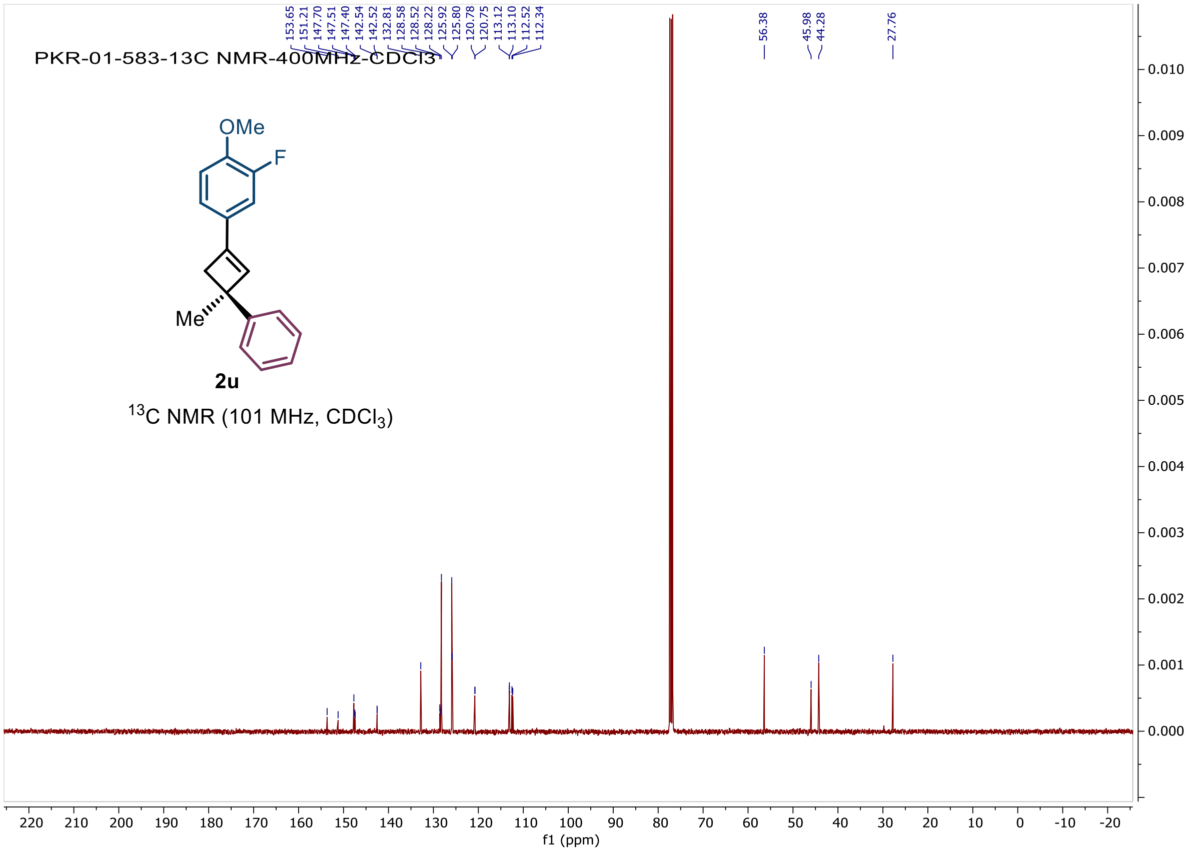
PKR-01-583-13C NMR-400MHz-CDCl3
OMe
F
Me
2u
^{13}C NMR (101 MHz, CDCl$_3$)
153.65
151.21
147.70
147.51
147.40
142.54
142.52
132.81
128.58
128.52
128.22
125.92
125.80
120.78
120.75
113.12
113.10
112.52
112.34
56.38
45.98
44.28
27.76
f1 (ppm)

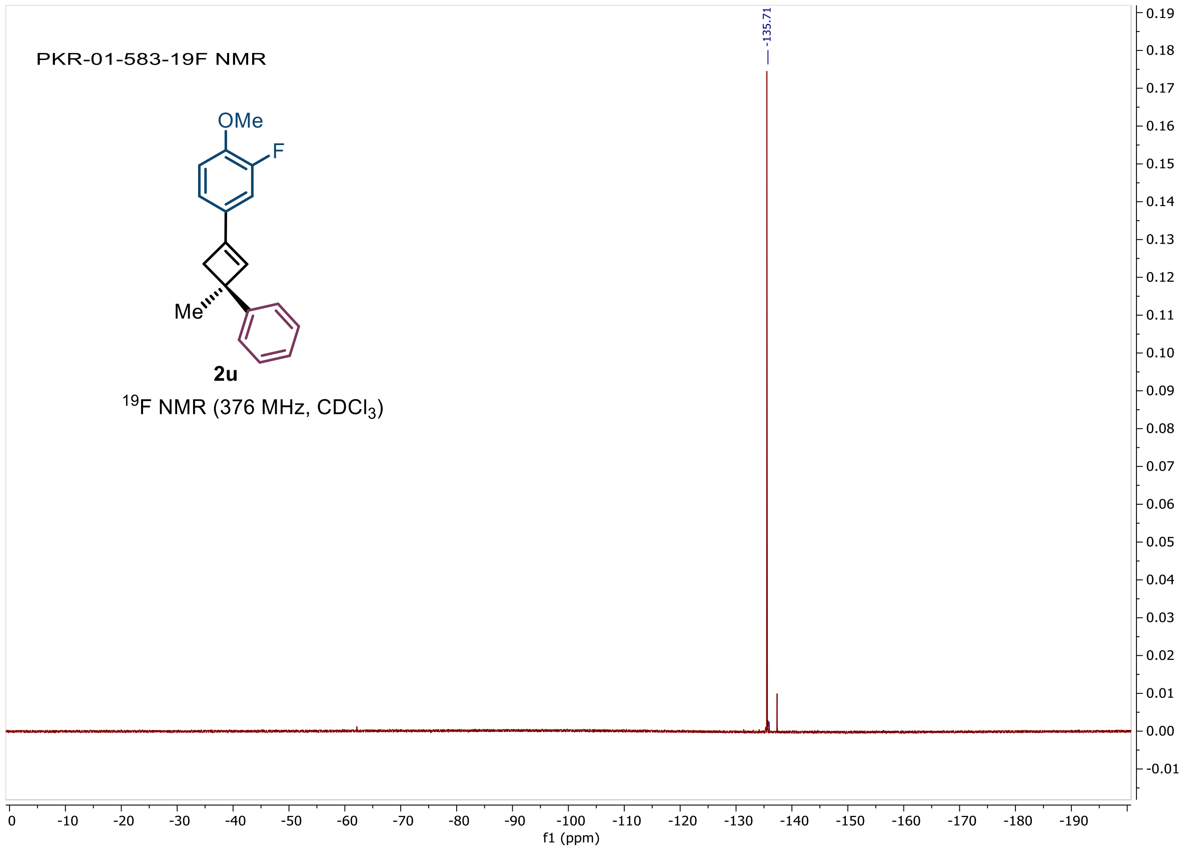
PKR-01-583-19F NMR
OMe
F
Me
2u
^{19}F NMR (376 MHz, CDCl$_3$)
-135.71
f1 (ppm)

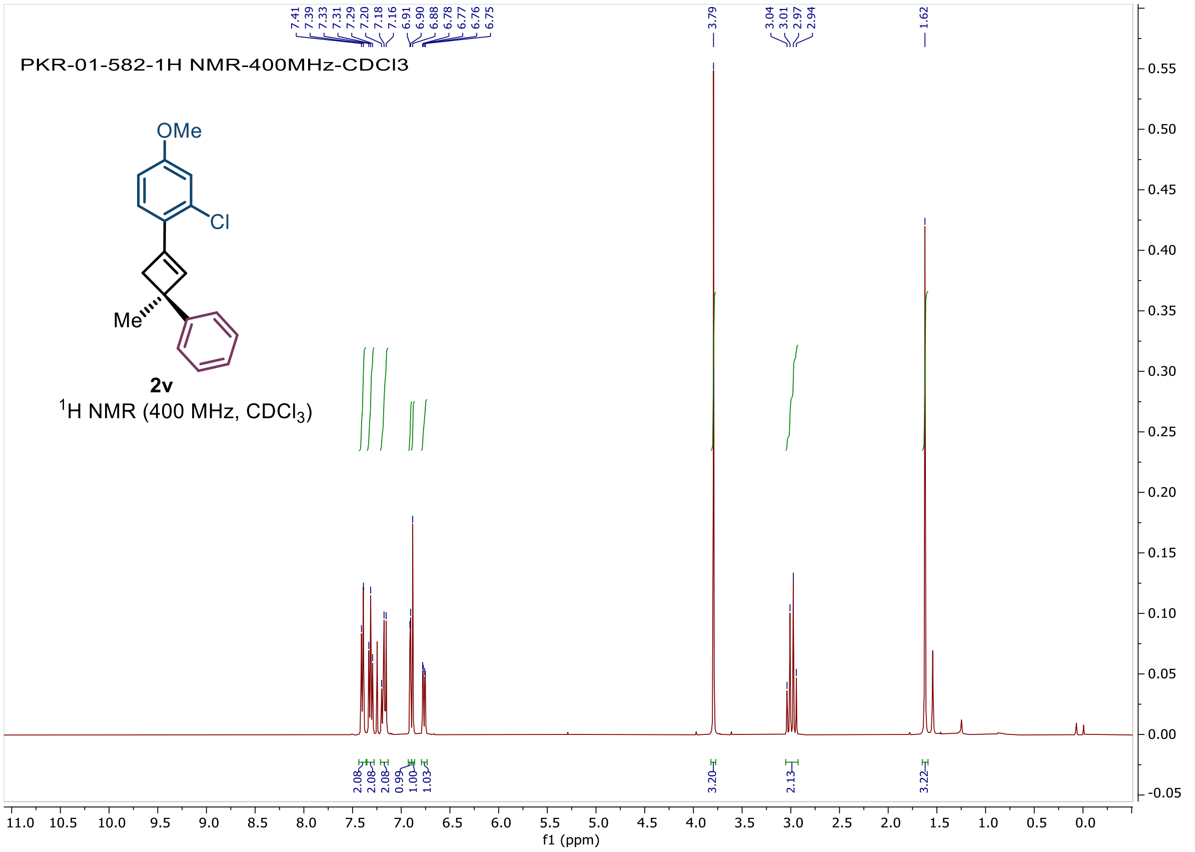
PKR-01-582-1H NMR-400MHz-CDCl3
OMe
Cl
Me
2v
¹H NMR (400 MHz, CDCl₃)
7.41
7.39
7.33
7.31
7.29
7.20
7.18
7.16
6.91
6.90
6.88
6.78
6.77
6.76
6.75
3.79
3.04
3.01
2.97
2.94
1.62
2.08
2.08
2.08
0.99
1.00
1.03
3.20
2.13
3.22
f1 (ppm)

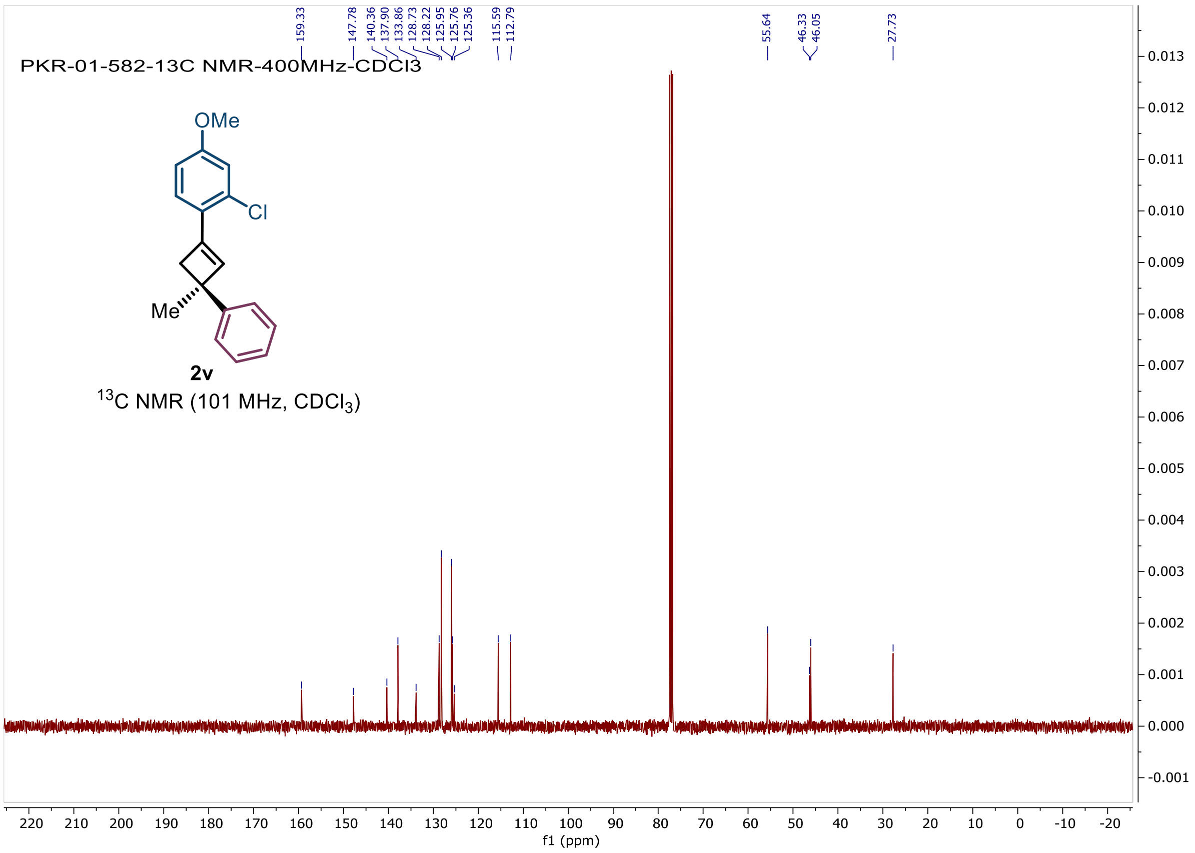
PKR-01-582-13C NMR-400MHz-CDCl3
OMe
Cl
Me
2v
¹³C NMR (101 MHz, CDCl₃)
159.33
147.78
140.36
137.90
133.86
128.73
128.22
125.95
125.76
125.36
115.59
112.79
55.64
46.33
46.05
27.73
f1 (ppm)

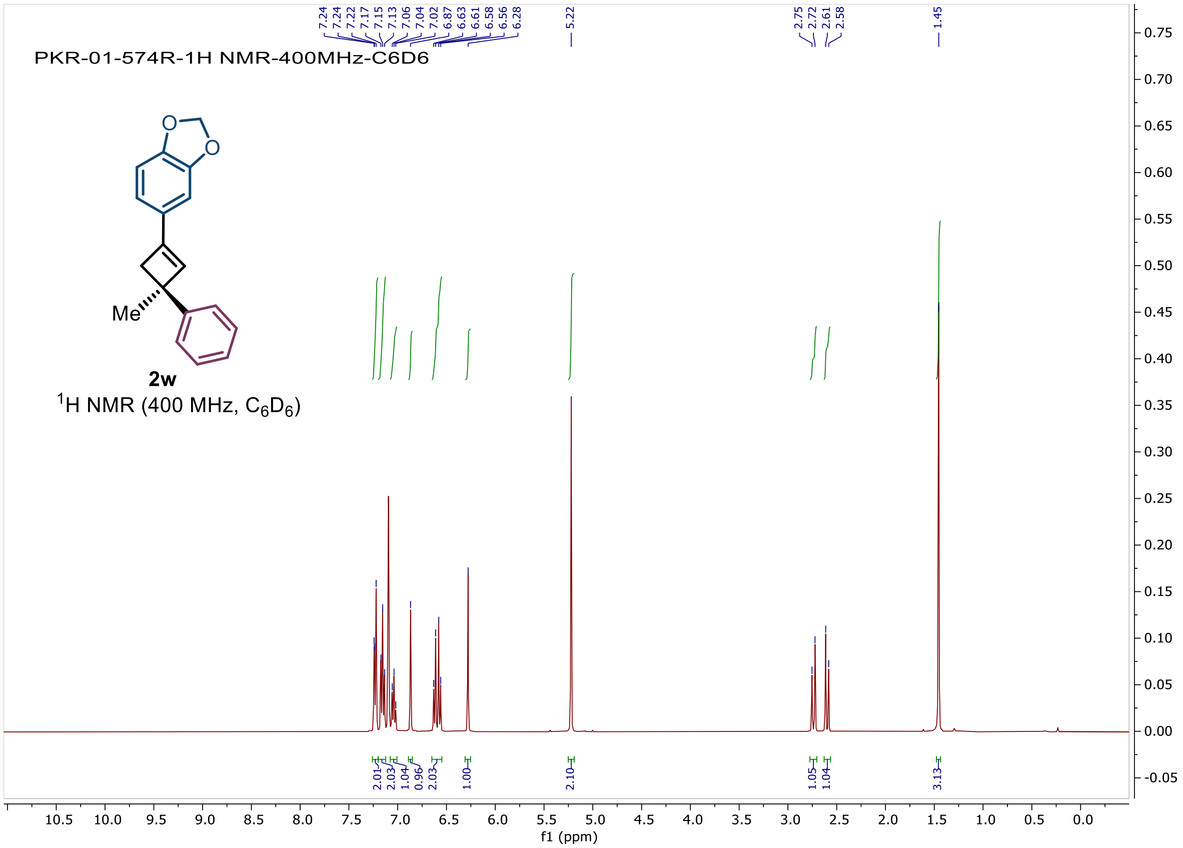
PKR-01-574R-1H NMR-400MHz-C6D6
7.24
7.24
7.22
7.17
7.15
7.13
7.06
7.04
7.02
6.87
6.63
6.61
6.58
6.56
6.28
5.22
2.75
2.72
2.61
2.58
1.45
2w
^{1}H NMR (400 MHz, C_6D_6)
2.01
2.03
1.04
0.96
2.03
1.00
2.10
1.05
1.04
3.13
f1 (ppm)

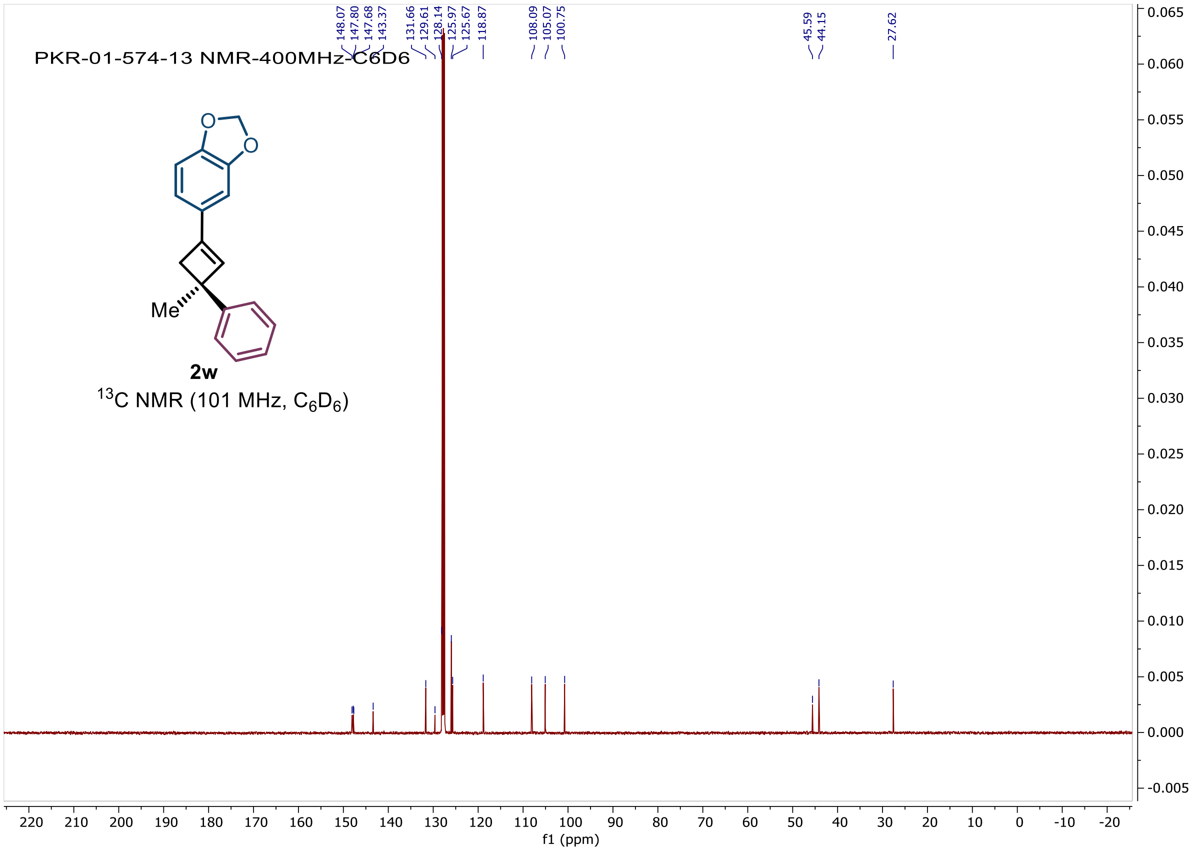
PKR-01-574-13 NMR-400MHz-C6D6
148.07
147.80
147.68
143.37
131.66
129.61
128.14
125.97
125.67
118.87
108.09
105.07
100.75
45.59
44.15
27.62
2w
^{13}C NMR (101 MHz, C_6D_6)
f1 (ppm)

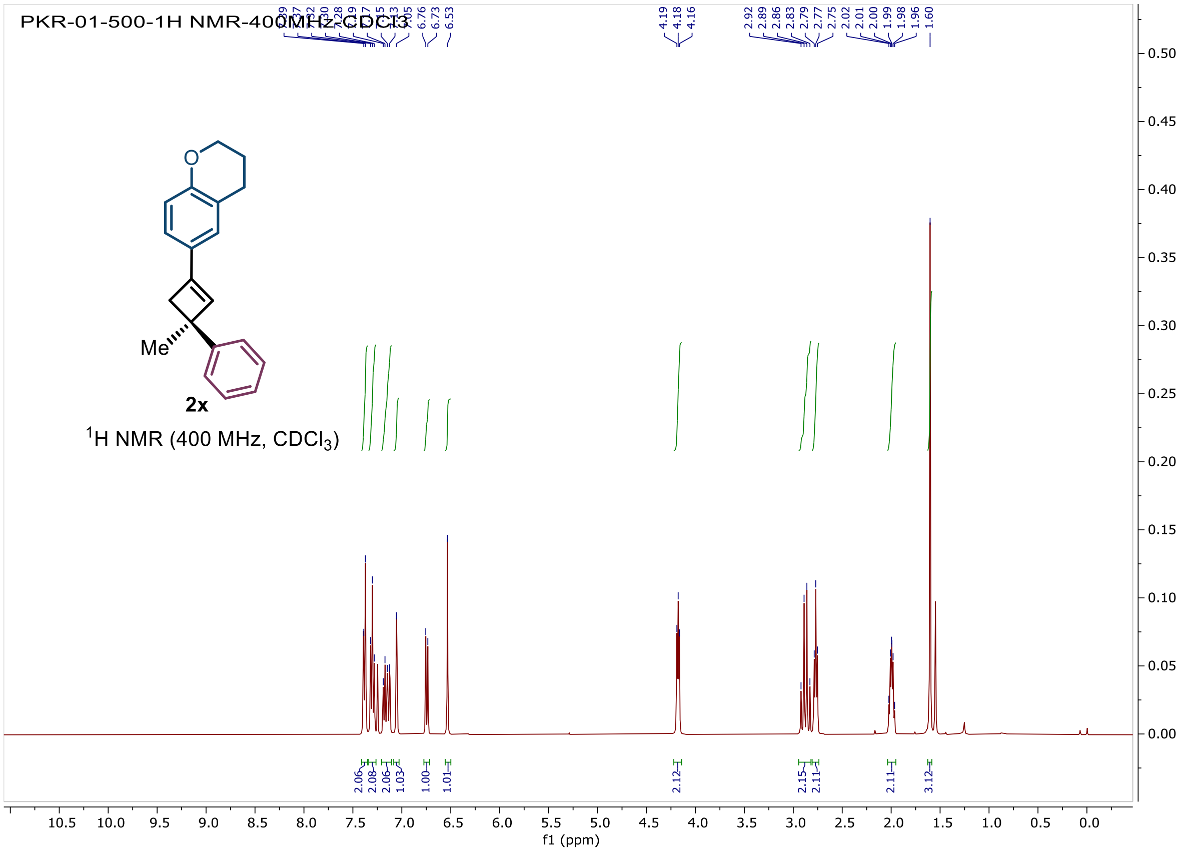
PKR-01-500-1H NMR-400MHz-CDCl3
Me
2x
^{1}H NMR (400 MHz, CDCl$_3$)
f1 (ppm)

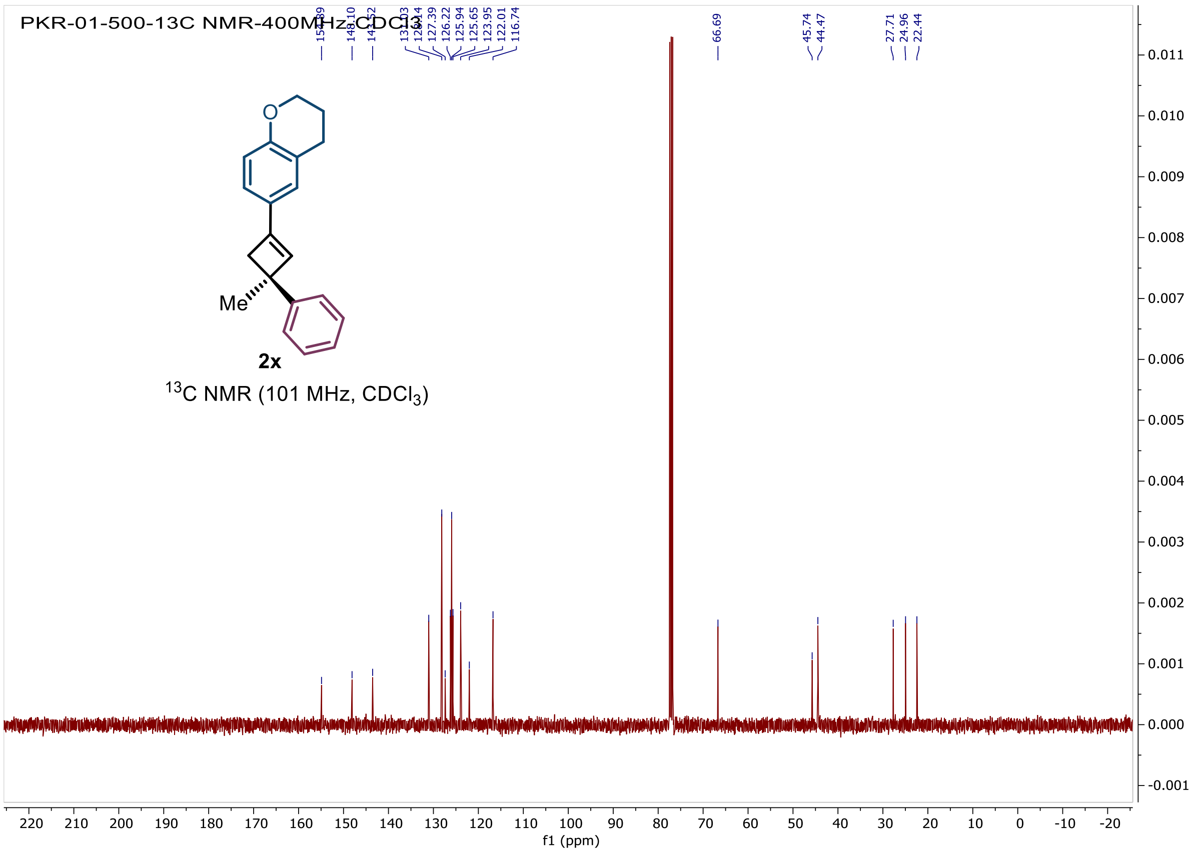
PKR-01-500-13C NMR-400MHz-CDCl3
Me
2x
^{13}C NMR (101 MHz, CDCl$_3$)
f1 (ppm)

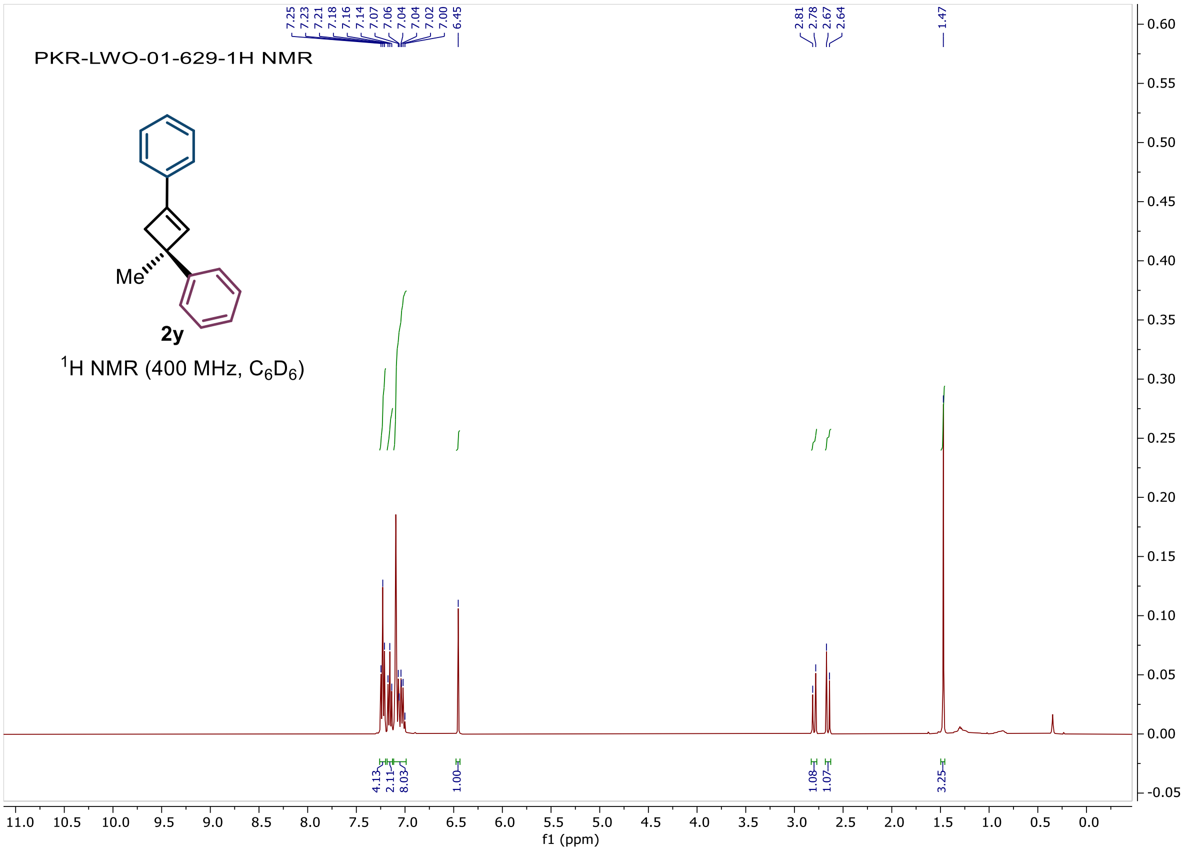
PKR-LWO-01-629-1H NMR
Me
2y
^{1}H NMR (400 MHz, C_6D_6)
f1 (ppm)

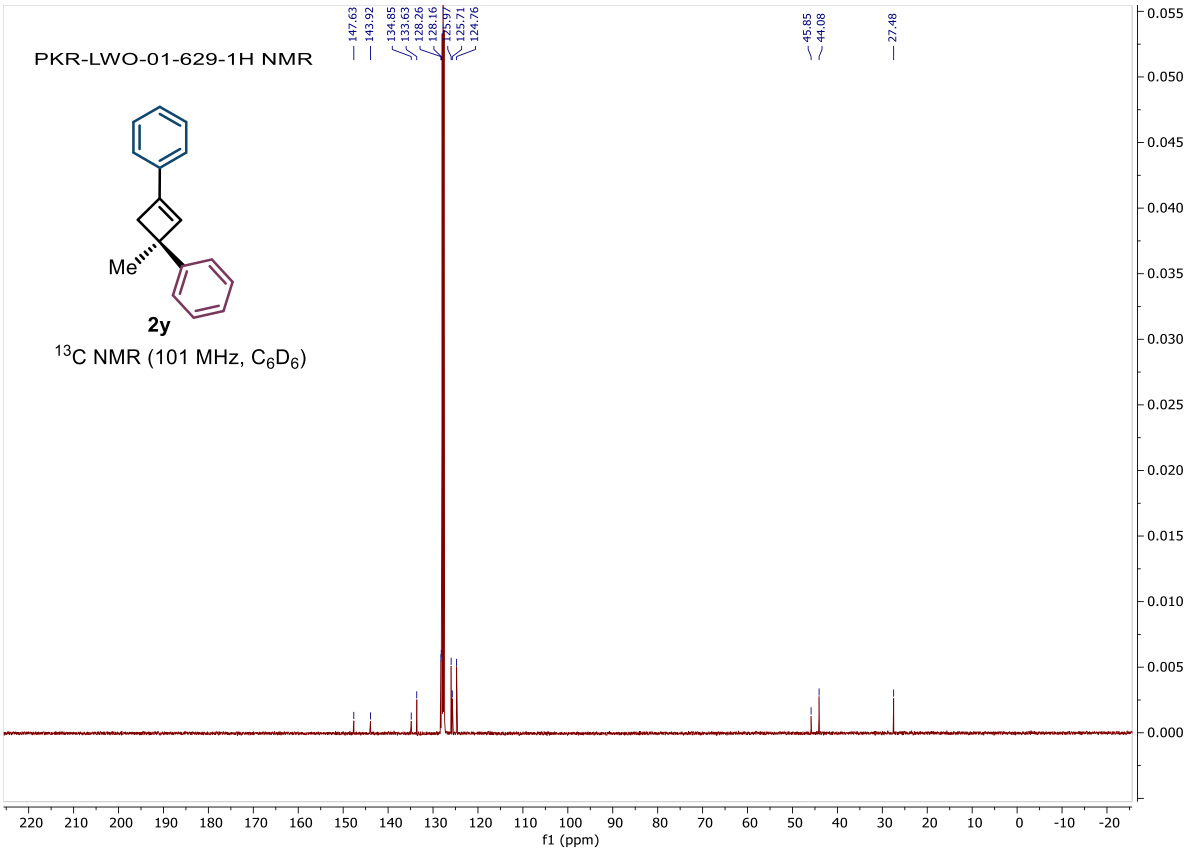
PKR-LWO-01-629-1H NMR
Me
2y
^{13}C NMR (101 MHz, C_6D_6)
f1 (ppm)

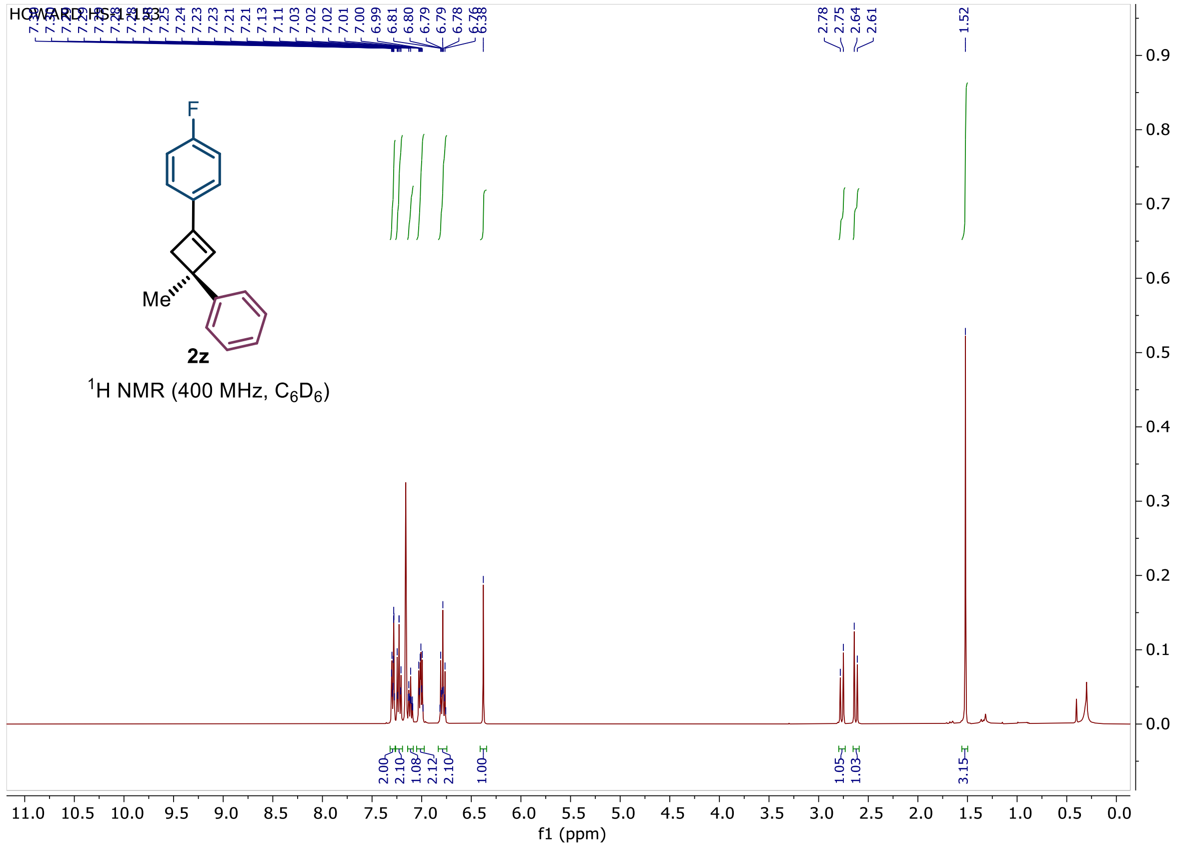
HOWARD HS-1-153
7.25 7.24 7.23 7.23 7.21 7.21 7.13 7.11 7.03 7.02 7.02 7.01 7.00 6.99 6.81 6.80 6.79 6.79 6.78 6.76 6.38
2.78 2.75 2.64 2.61
1.52
F
Me
2z
^{1}H NMR (400 MHz, C_6D_6)
2.00 2.10 1.08 2.12 2.10 1.00 1.05 1.03 3.15
f1 (ppm)

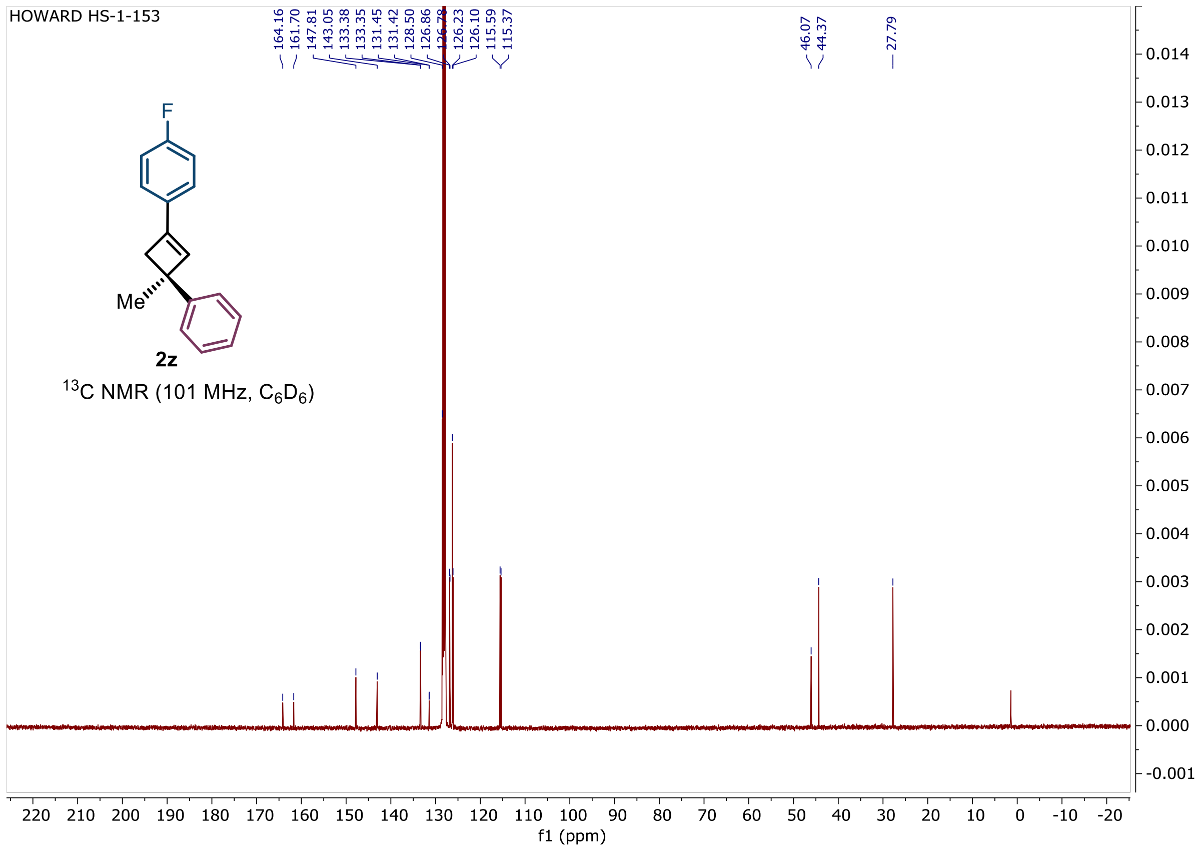
HOWARD HS-1-153
164.16 161.70 147.81 143.05 133.38 133.35 131.45 131.42 128.50 126.86 126.23 126.10 115.59 115.37
46.07 44.37
27.79
F
Me
2z
^{13}C NMR (101 MHz, C_6D_6)
f1 (ppm)

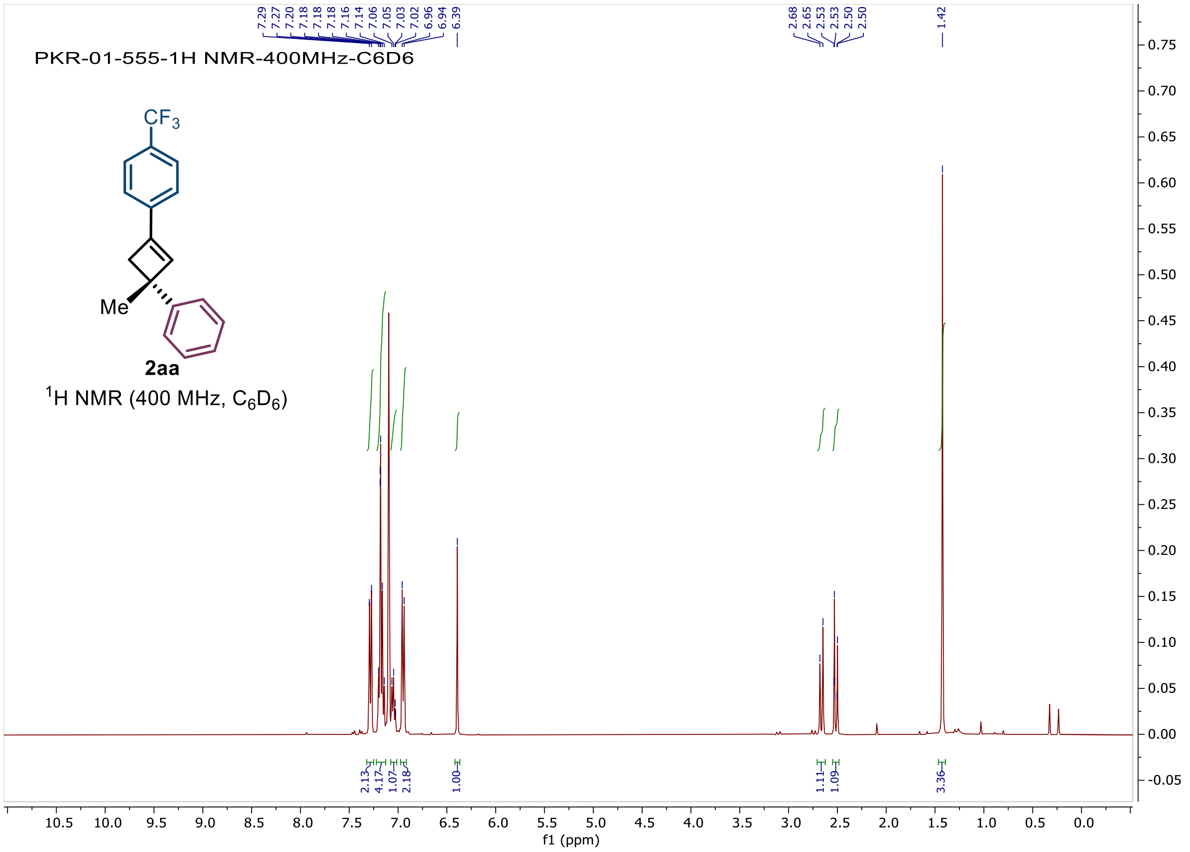
PKR-01-555-1H NMR-400MHz-C6D6
CF3
Me
2aa
1H NMR (400 MHz, C6D6)
7.29 7.27 7.20 7.18 7.18 7.18 7.16 7.14 7.06 7.05 7.03 7.02 6.96 6.94 6.39
2.68 2.65 2.53 2.53 2.50 2.50
1.42
2.13 4.17 1.07 2.18 1.00 1.11 1.09 3.36
f1 (ppm)

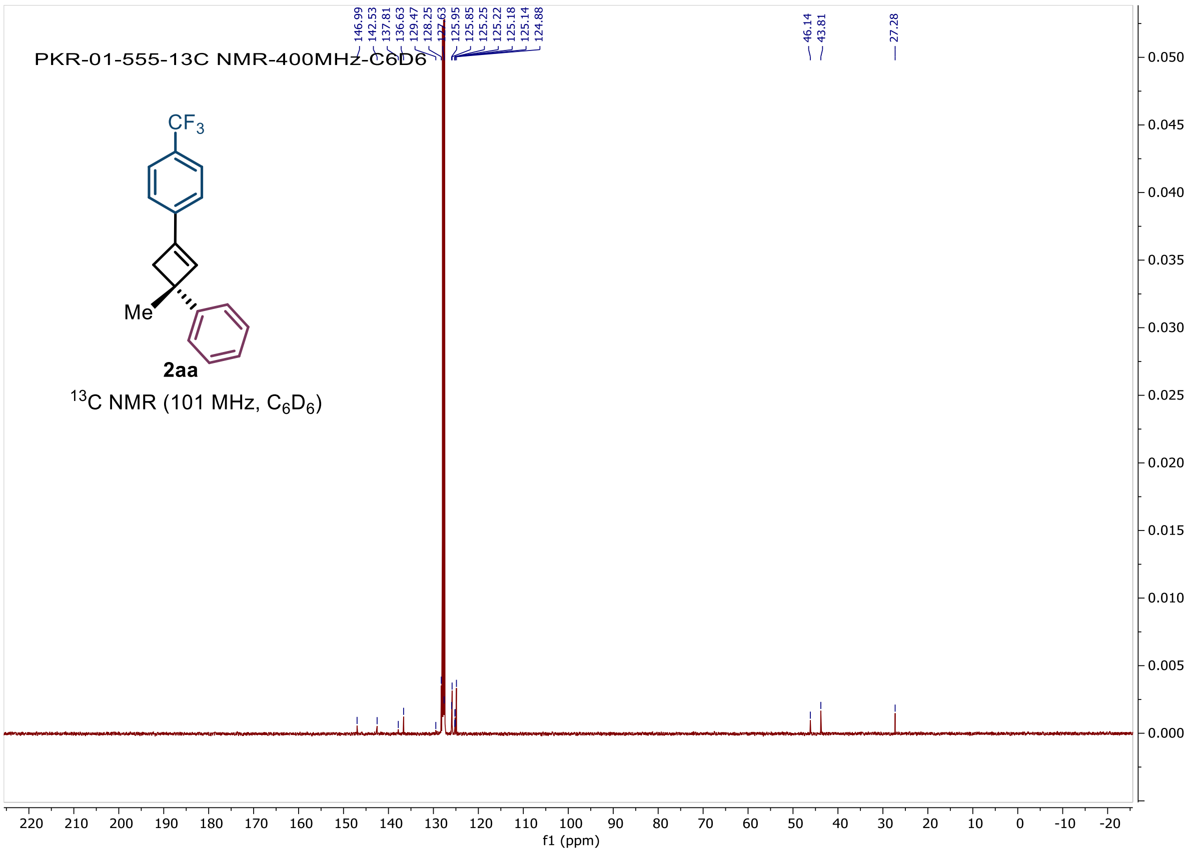
PKR-01-555-13C NMR-400MHz-C6D6
CF3
Me
2aa
13C NMR (101 MHz, C6D6)
146.99 142.53 137.81 136.63 129.47 128.25 127.63 125.95 125.85 125.25 125.22 125.18 125.14 124.88
46.14 43.81
27.28
f1 (ppm)

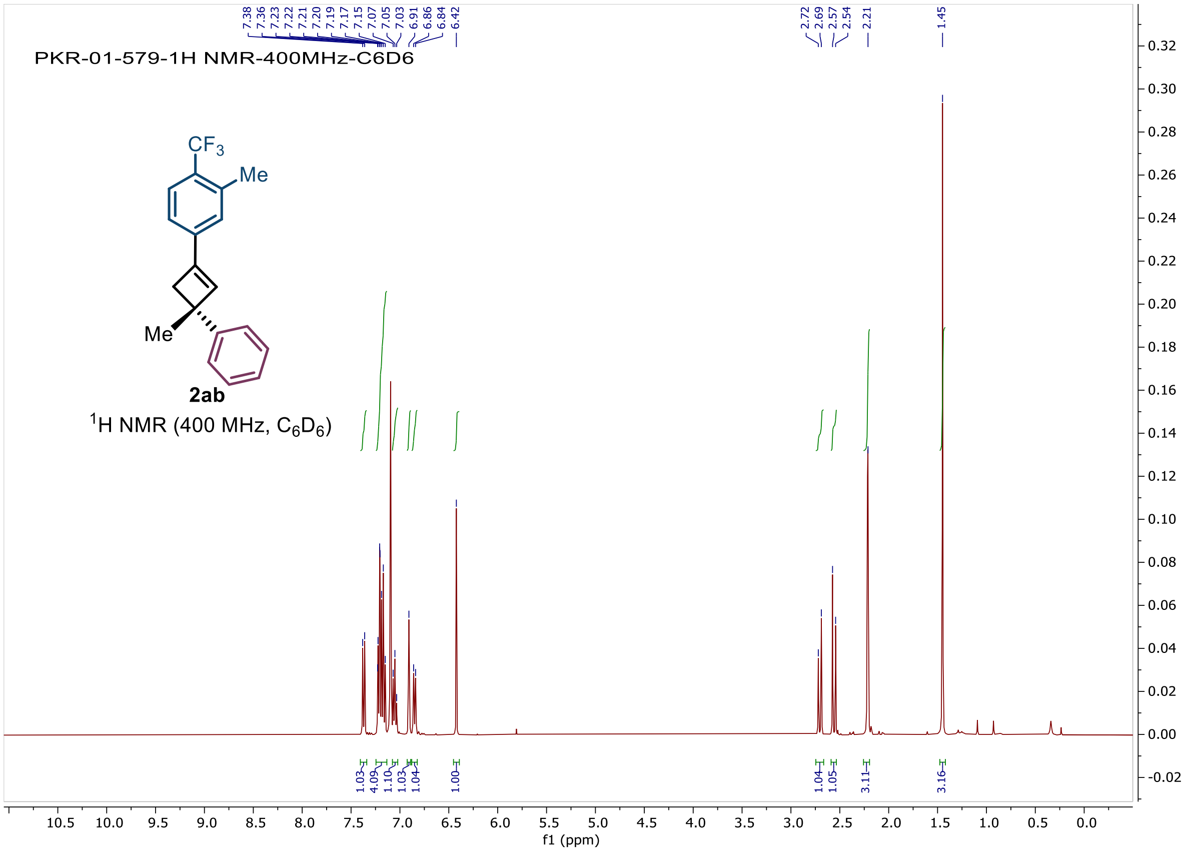
PKR-01-579-1H NMR-400MHz-C6D6
7.38 7.36 7.23 7.22 7.21 7.20 7.19 7.17 7.15 7.07 7.05 7.03 6.91 6.86 6.84 6.42
2.72 2.69 2.57 2.54 2.21
1.45
CF3
Me
Me
2ab
1H NMR (400 MHz, C6D6)
1.03 4.09 1.10 1.03 1.04 1.00 1.04 1.05 3.11 3.16
f1 (ppm)

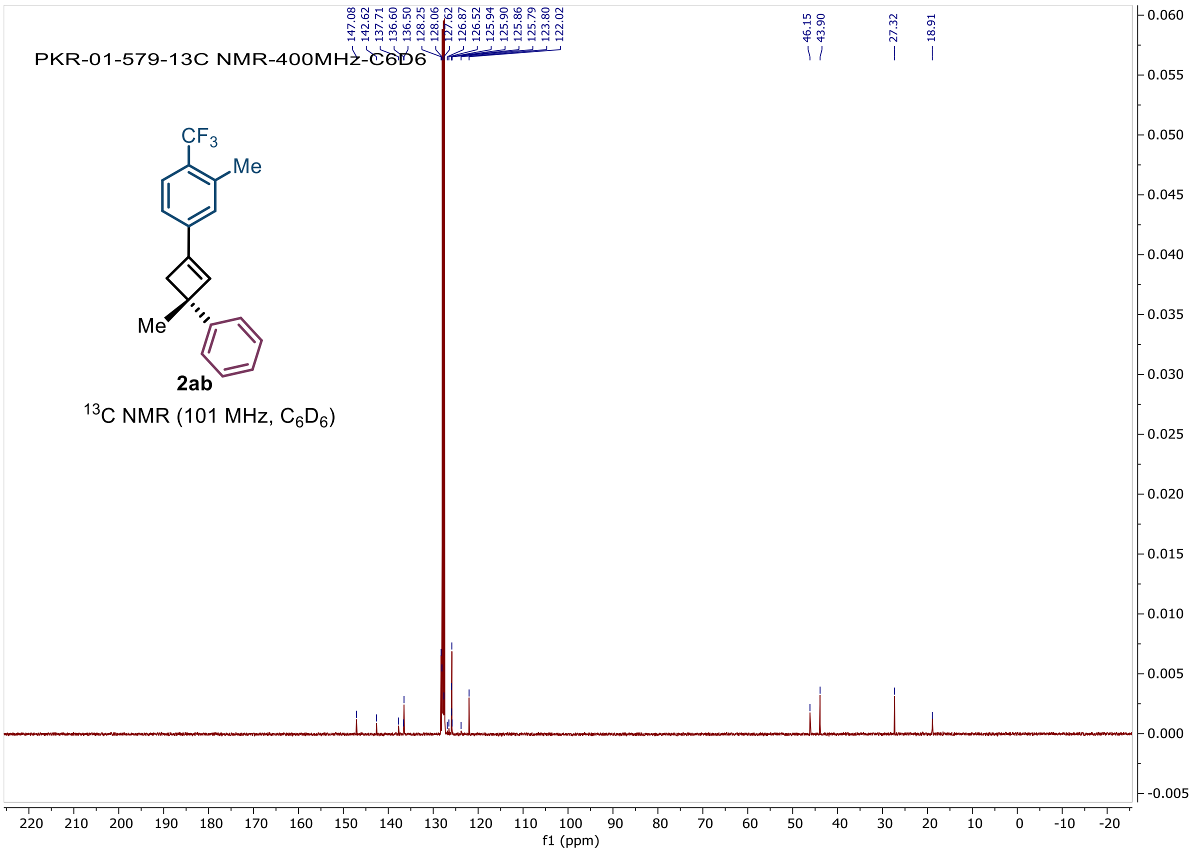
PKR-01-579-13C NMR-400MHz-C6D6
147.08 142.62 137.71 136.60 136.50 128.25 128.06 127.62 126.87 126.52 125.94 125.90 125.86 125.79 123.80 122.02
46.15 43.90
27.32
18.91
CF3
Me
Me
2ab
13C NMR (101 MHz, C6D6)
f1 (ppm)

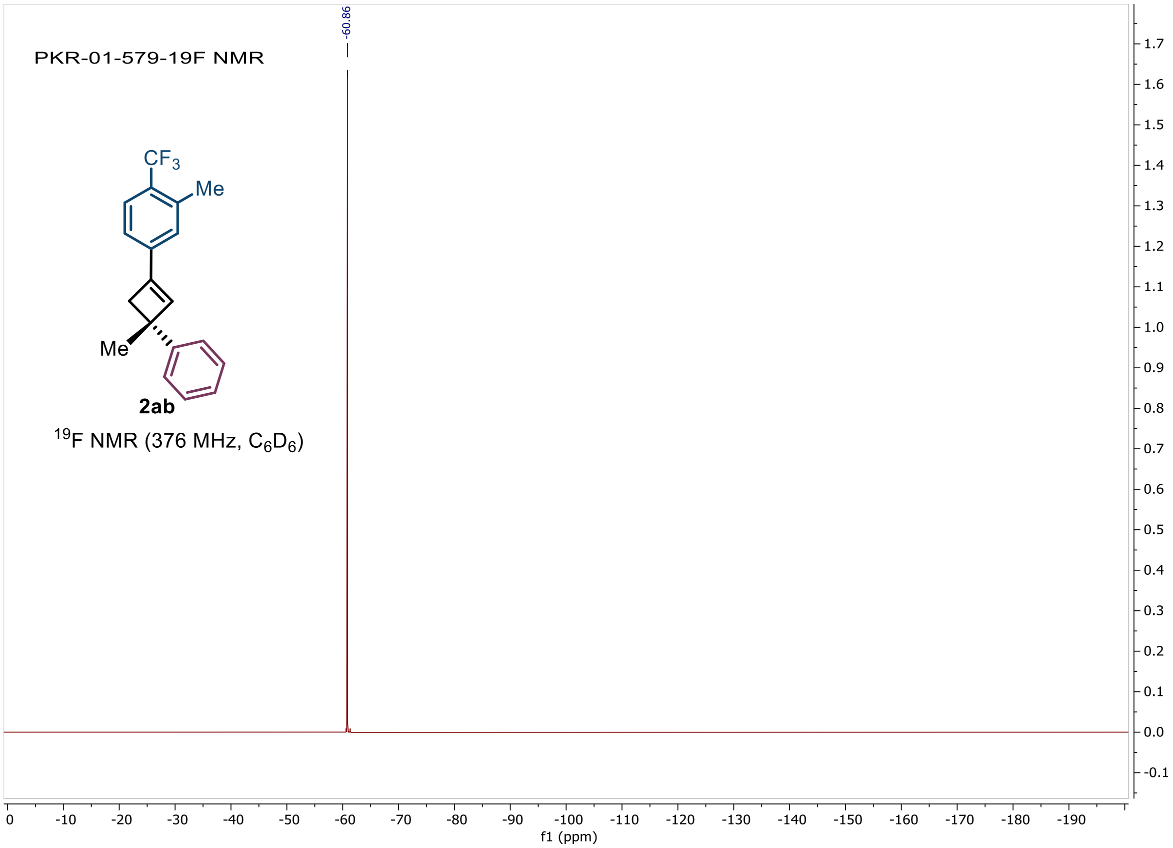
PKR-01-579-19F NMR
CF3
Me
Me
2ab
19F NMR (376 MHz, C6D6)
-60.86
f1 (ppm)

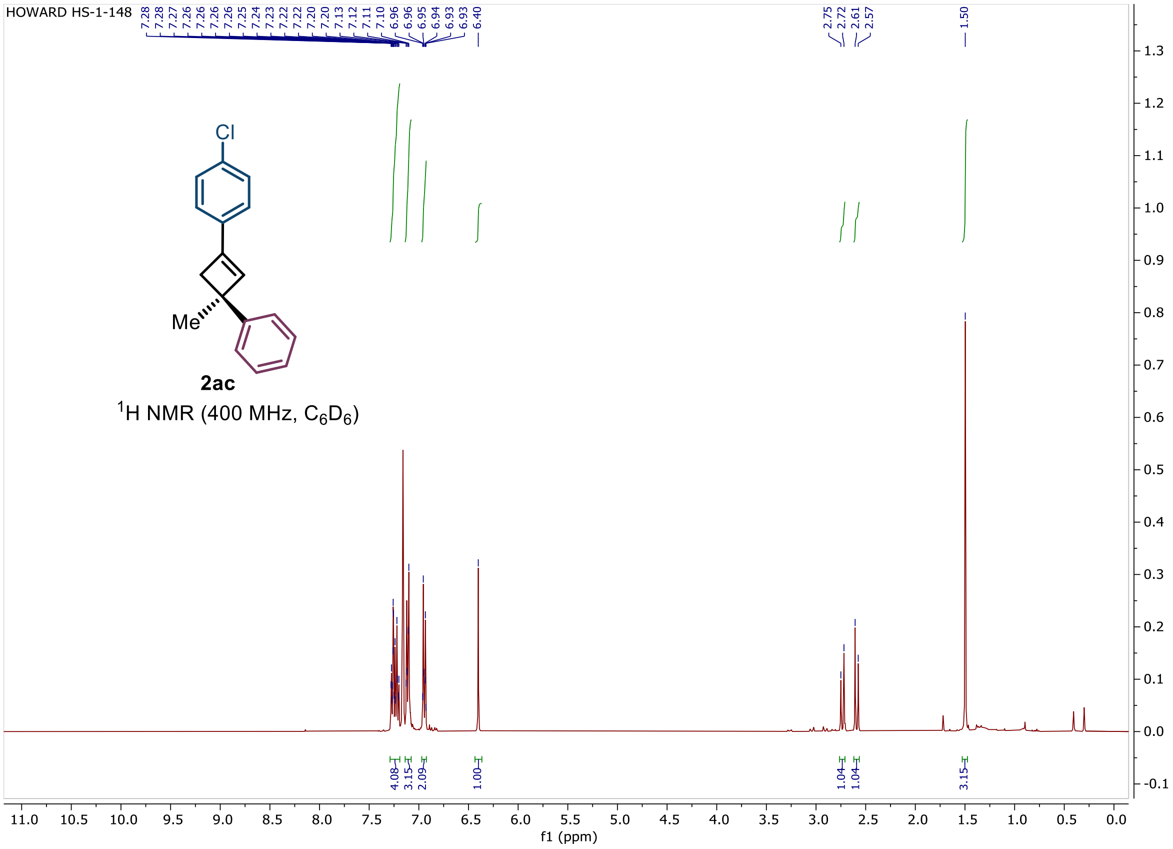
HOWARD HS-1-148
Cl
Me
2ac
1H NMR (400 MHz, C6D6)
7.28 7.28 7.27 7.26 7.26 7.26 7.25 7.24 7.23 7.22 7.22 7.20 7.20 7.13 7.12 7.11 7.10 6.96 6.96 6.95 6.94 6.93 6.93 6.40
2.75 2.72 2.61 2.57
1.50
4.08 3.15 2.09 1.00 1.04 1.04 3.15
f1 (ppm)

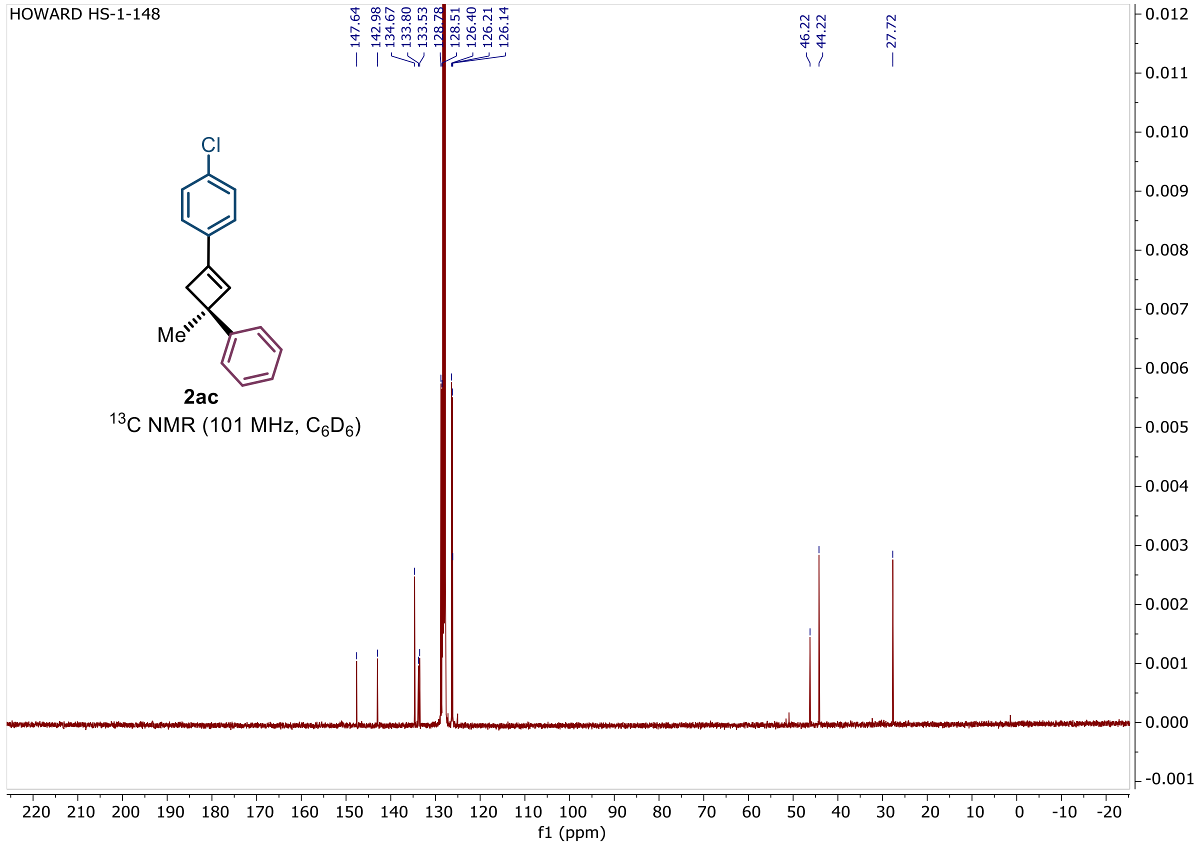
HOWARD HS-1-148
147.64
142.98
134.67
133.80
133.53
128.78
128.51
126.40
126.21
126.14
46.22
44.22
27.72
Cl
Me
2ac
13C NMR (101 MHz, C6D6)
f1 (ppm)

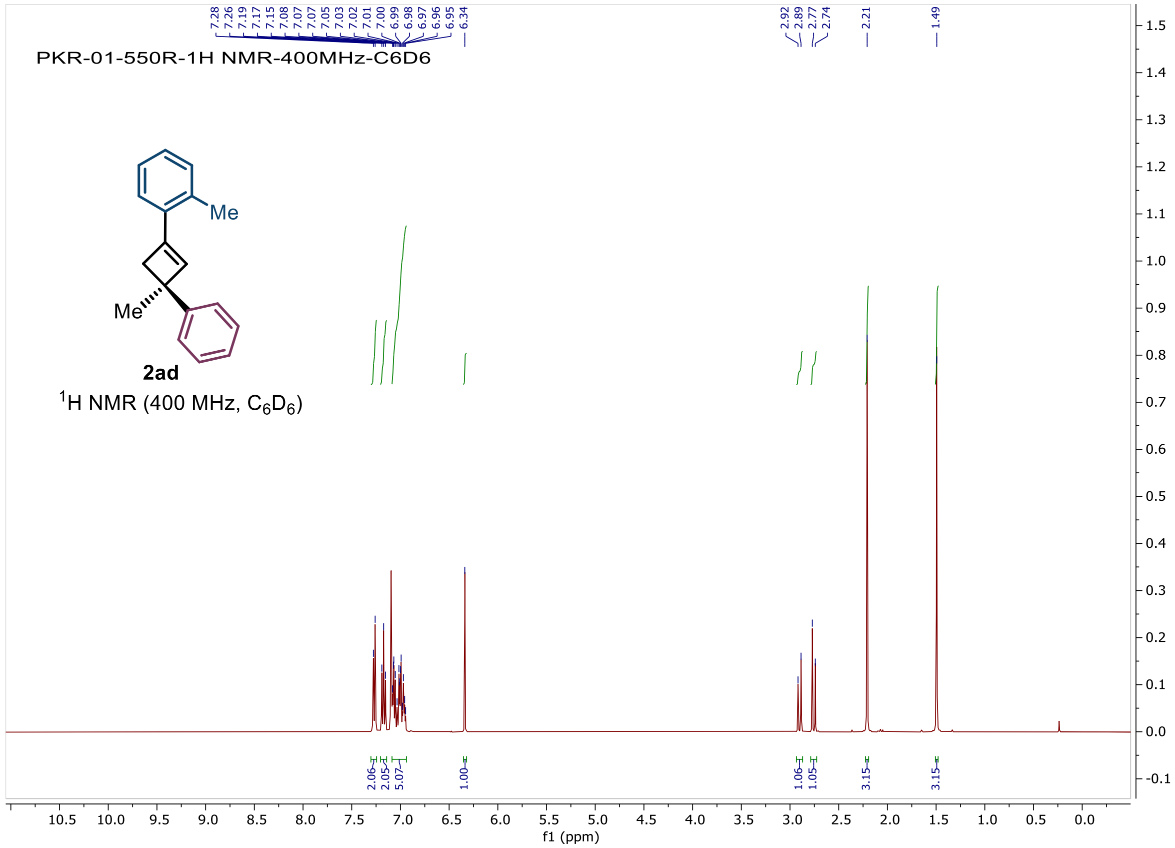
PKR-01-550R-1H NMR-400MHz-C6D6
7.28
7.26
7.19
7.17
7.15
7.08
7.07
7.07
7.05
7.03
7.02
7.01
7.00
6.99
6.98
6.97
6.96
6.95
6.34
2.92
2.89
2.77
2.74
2.21
1.49
Me
Me
2ad
1H NMR (400 MHz, C6D6)
2.06
2.05
5.07
1.00
1.06
1.05
3.15
3.15
f1 (ppm)

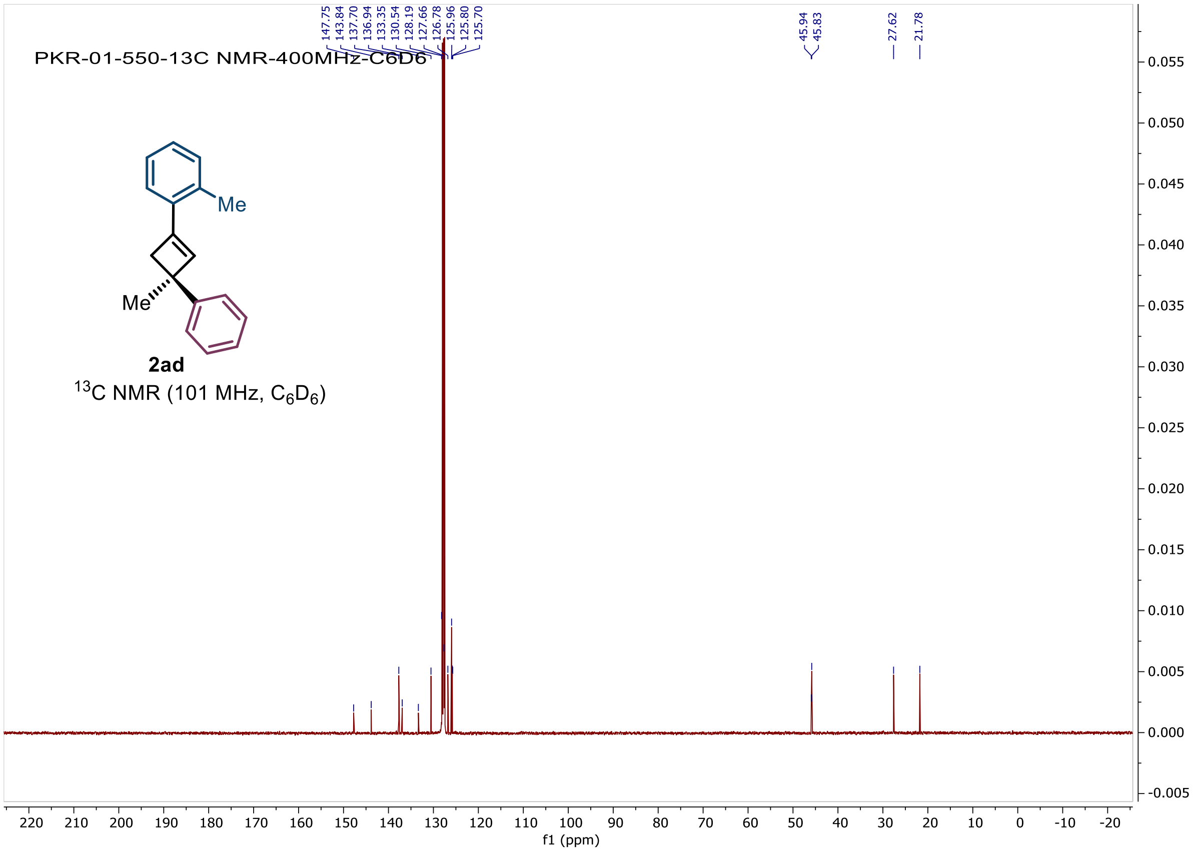
PKR-01-550-13C NMR-400MHz-C6D6
147.75
143.84
137.70
136.94
133.35
130.54
128.19
127.66
126.78
125.96
125.80
125.70
45.94
45.83
27.62
21.78
Me
Me
2ad
13C NMR (101 MHz, C6D6)
f1 (ppm)

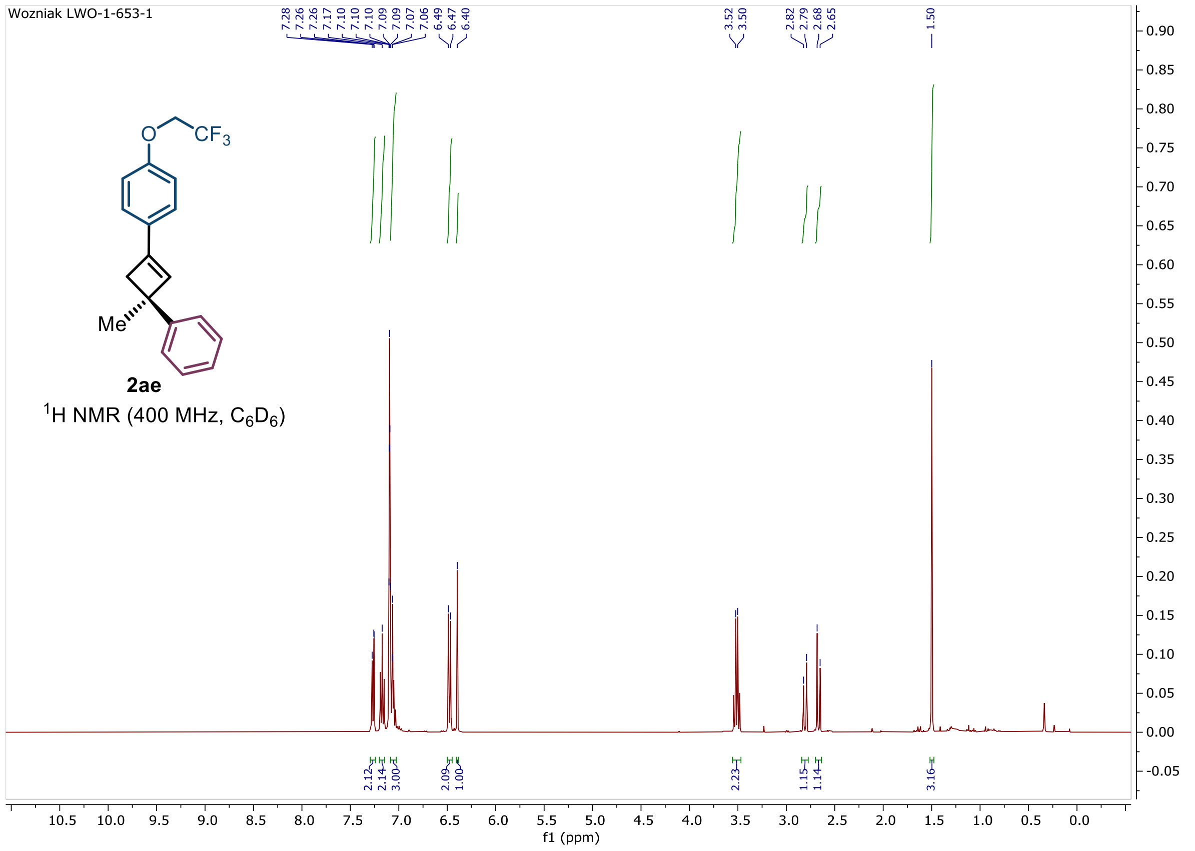
Wozniak LWO-1-653-1
7.28
7.26
7.26
7.17
7.10
7.10
7.10
7.09
7.09
7.07
7.06
6.49
6.47
6.40
3.52
3.50
2.82
2.79
2.68
2.65
1.50
O
CF3
Me
2ae
1H NMR (400 MHz, C6D6)
2.12
2.14
3.00
2.09
1.00
2.23
1.15
1.14
3.16
f1 (ppm)

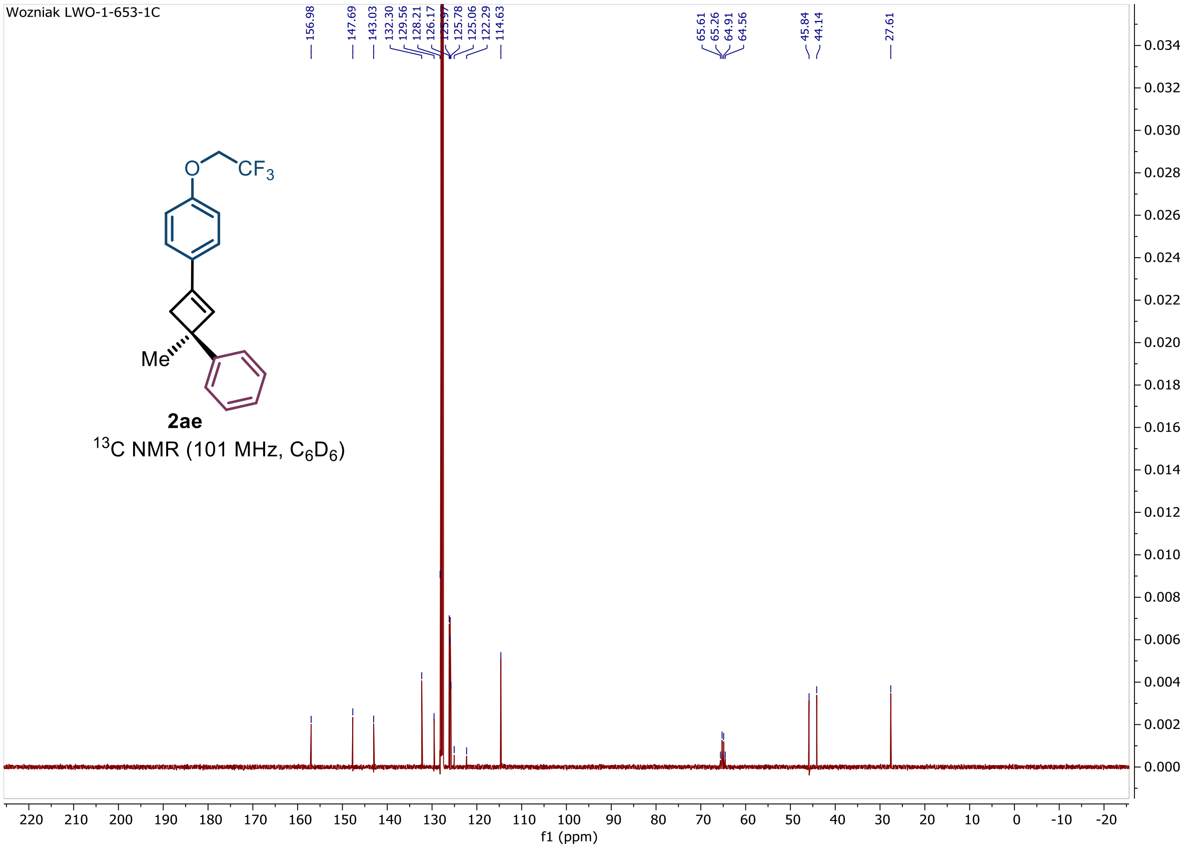
Wozniak LWO-1-653-1C
156.98
147.69
143.03
132.30
129.56
128.21
126.17
125.97
125.78
125.06
122.29
114.63
65.61
65.26
64.91
64.56
45.84
44.14
27.61
2ae
^{13}C NMR (101 MHz, C_6D_6)
f1 (ppm)

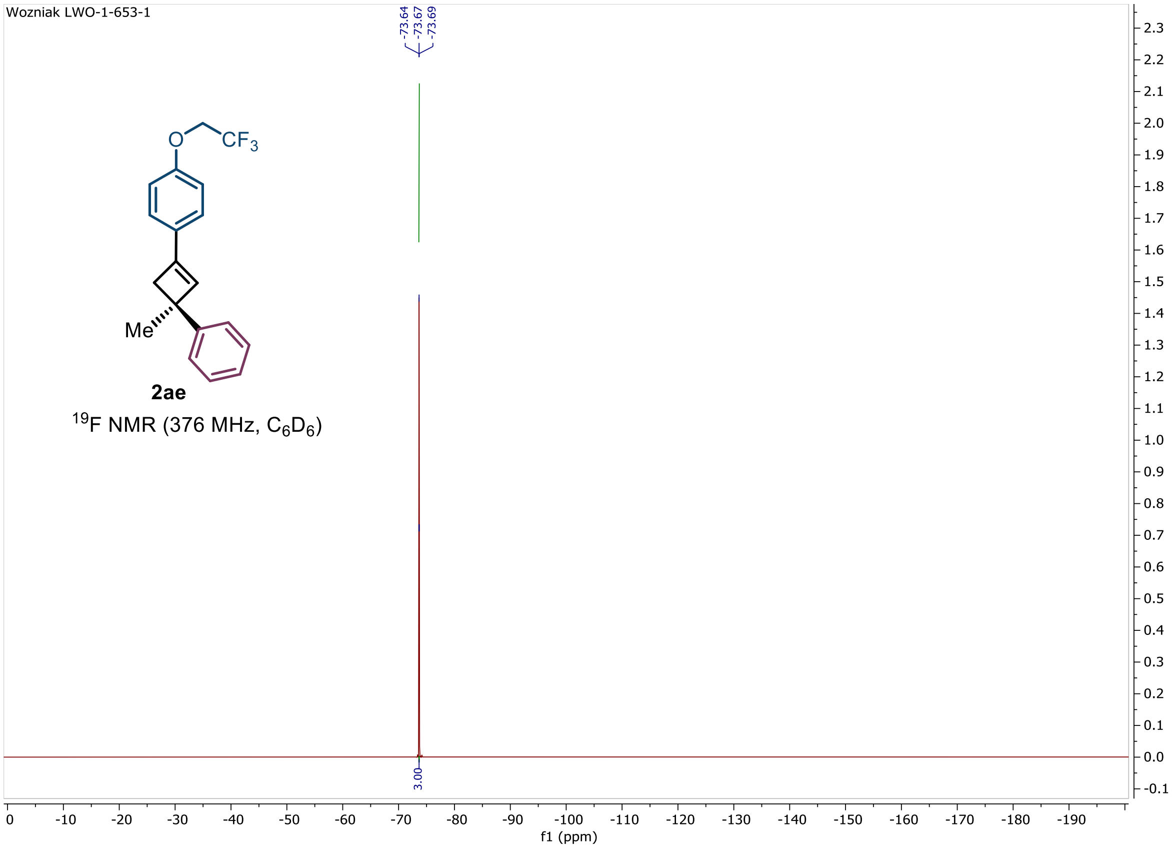
Wozniak LWO-1-653-1
-73.64
-73.67
-73.69
2ae
^{19}F NMR (376 MHz, C_6D_6)
3.00
f1 (ppm)

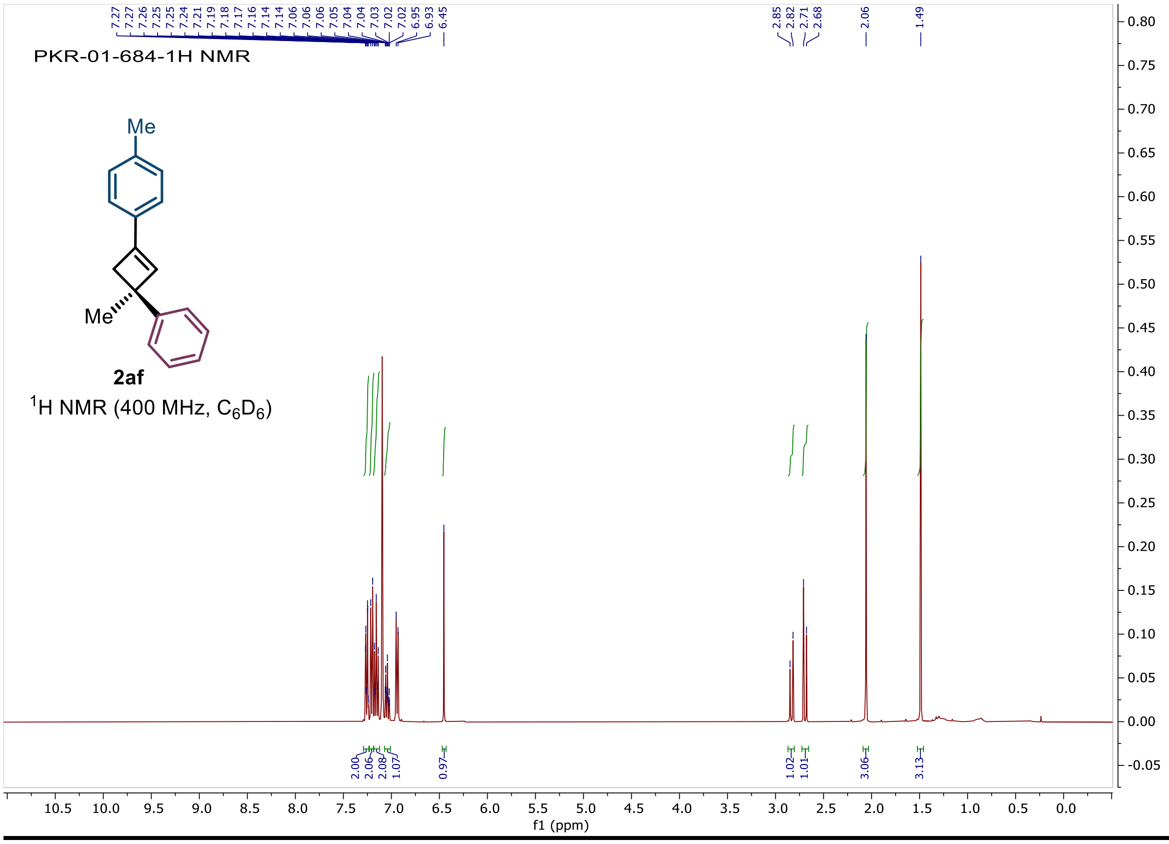
PKR-01-684-1H NMR
Me
Me
2af
¹H NMR (400 MHz, C6D6)
f1 (ppm)

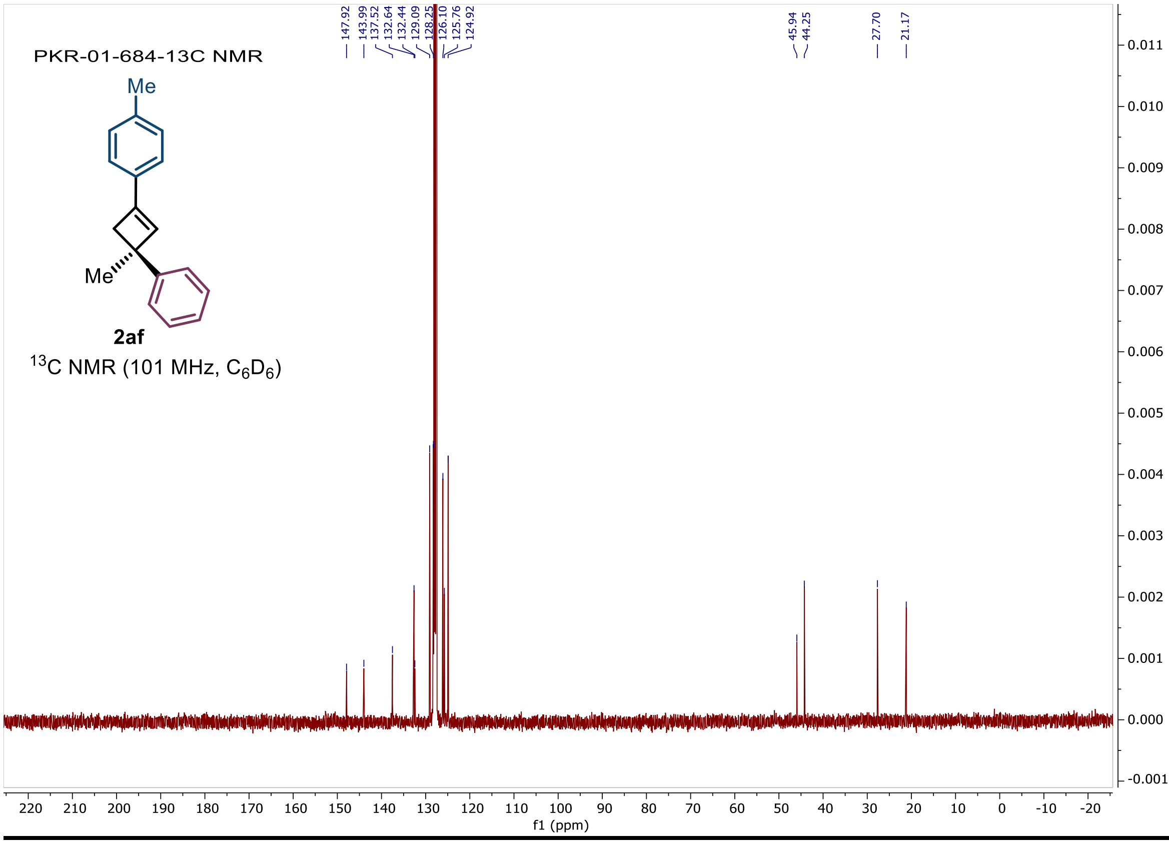
PKR-01-684-13C NMR
Me
Me
2af
¹³C NMR (101 MHz, C6D6)
f1 (ppm)

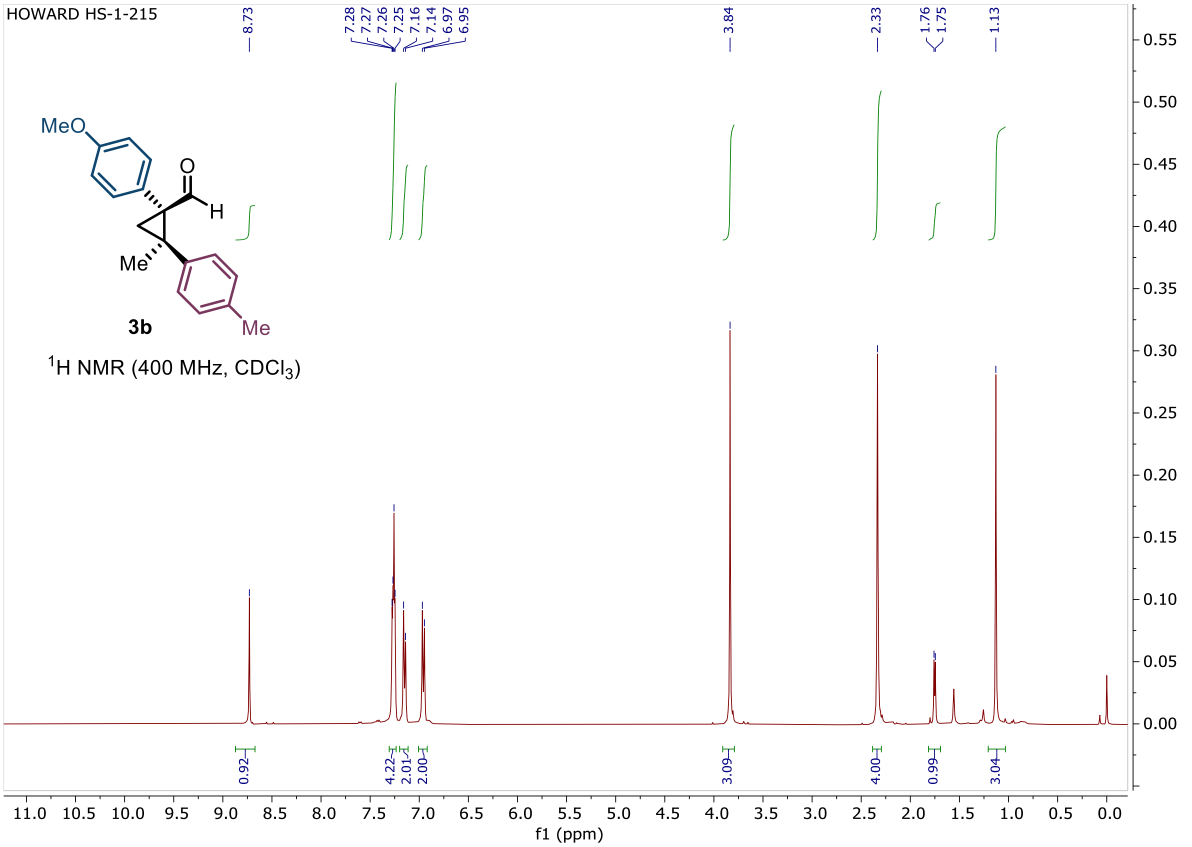
HOWARD HS-1-215
8.73
7.28
7.27
7.26
7.25
7.16
7.14
6.97
6.95
3.84
2.33
1.76
1.75
1.13
MeO
O
H
Me
Me
3b
^{1}H NMR (400 MHz, $CDCl_3$)
0.92
4.22
2.01
2.00
3.09
4.00
0.99
3.04
f1 (ppm)

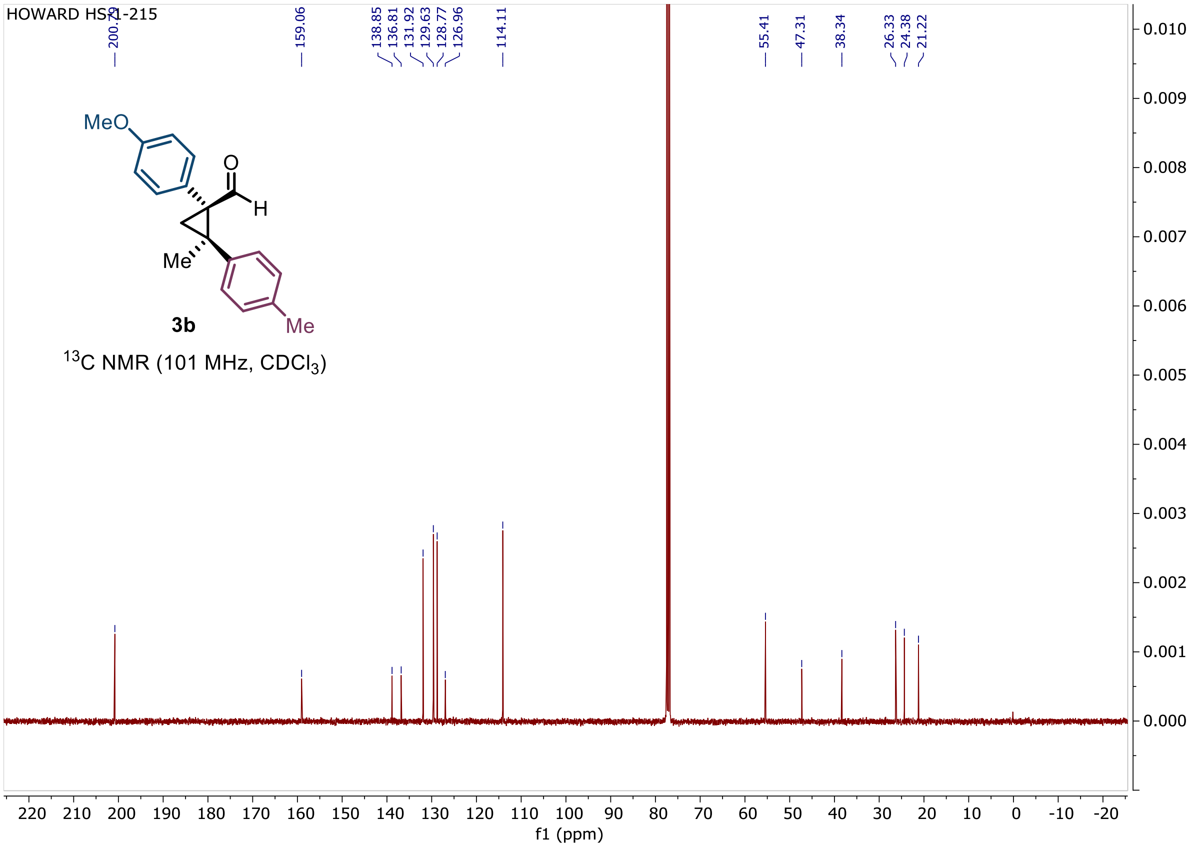
HOWARD HS-1-215
200.71
159.06
138.85
136.81
131.92
129.63
128.77
126.96
114.11
55.41
47.31
38.34
26.33
24.38
21.22
MeO
O
H
Me
Me
3b
^{13}C NMR (101 MHz, $CDCl_3$)
f1 (ppm)

PKR-01-637-1H NMR

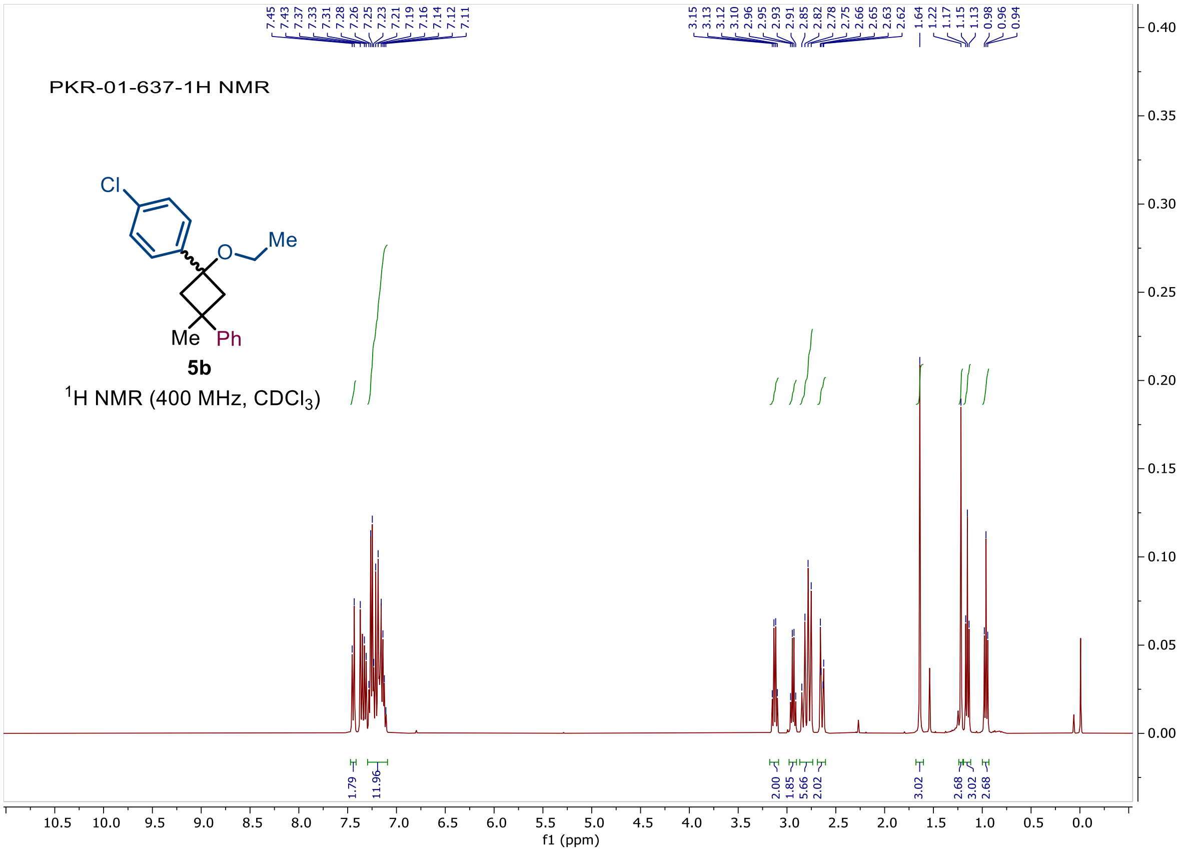

PKR-01-637-13C NMR

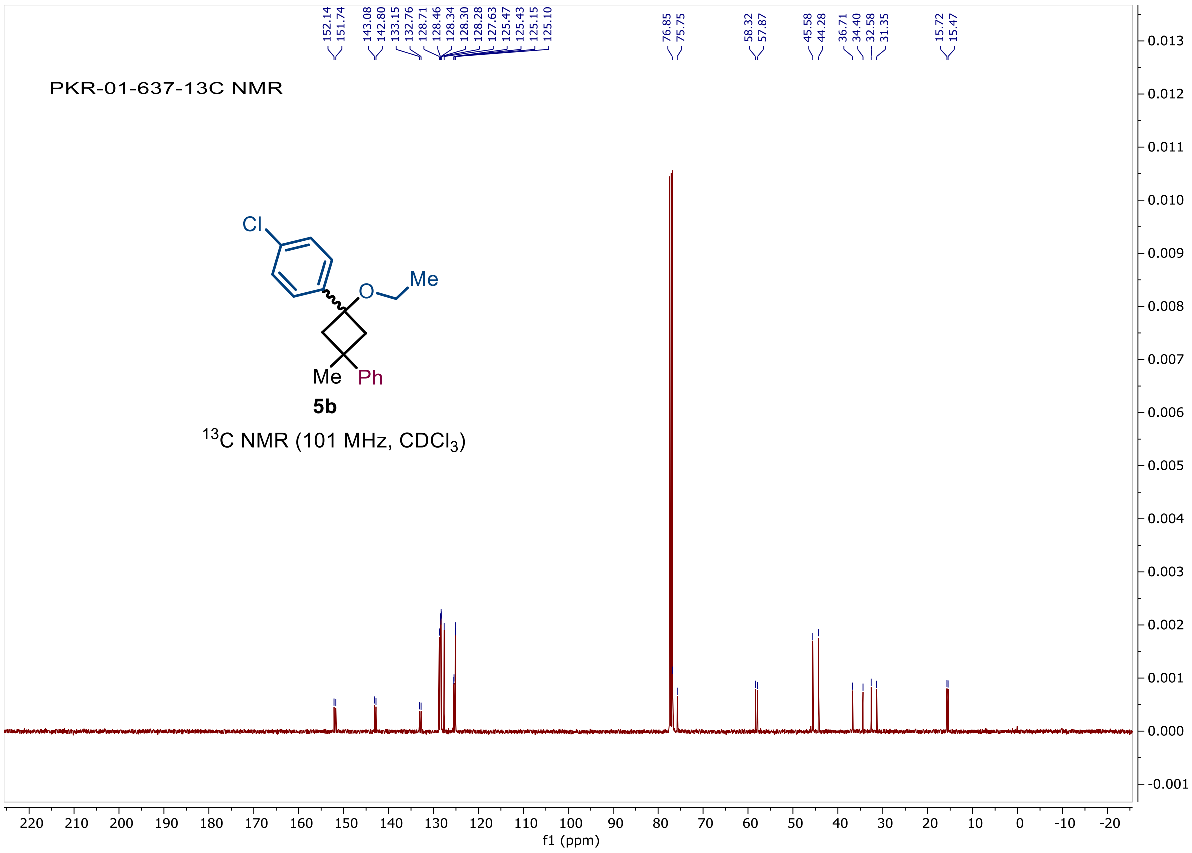

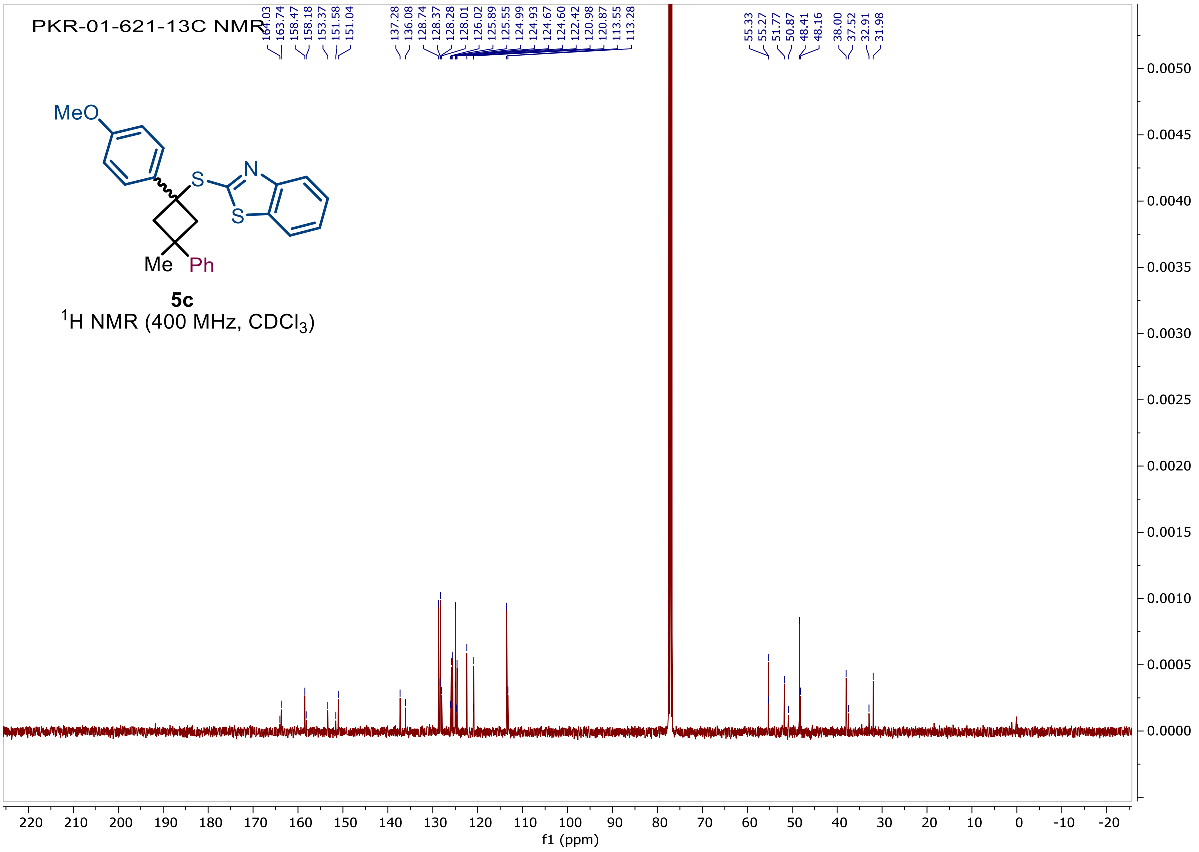
PKR-01-621-13C NMR
164.03 163.74 158.47 158.18 153.37 151.58 151.04 137.28 136.08 128.74 128.37 128.28 128.01 126.02 125.89 125.55 124.99 124.93 124.67 124.60 122.42 120.98 120.87 113.55 113.28
55.33 55.27 51.77 50.87 48.41 48.16 38.00 37.52 32.91 31.98
MeO
S
N
S
Me
Ph
5c
¹H NMR (400 MHz, CDCl₃)
f1 (ppm)

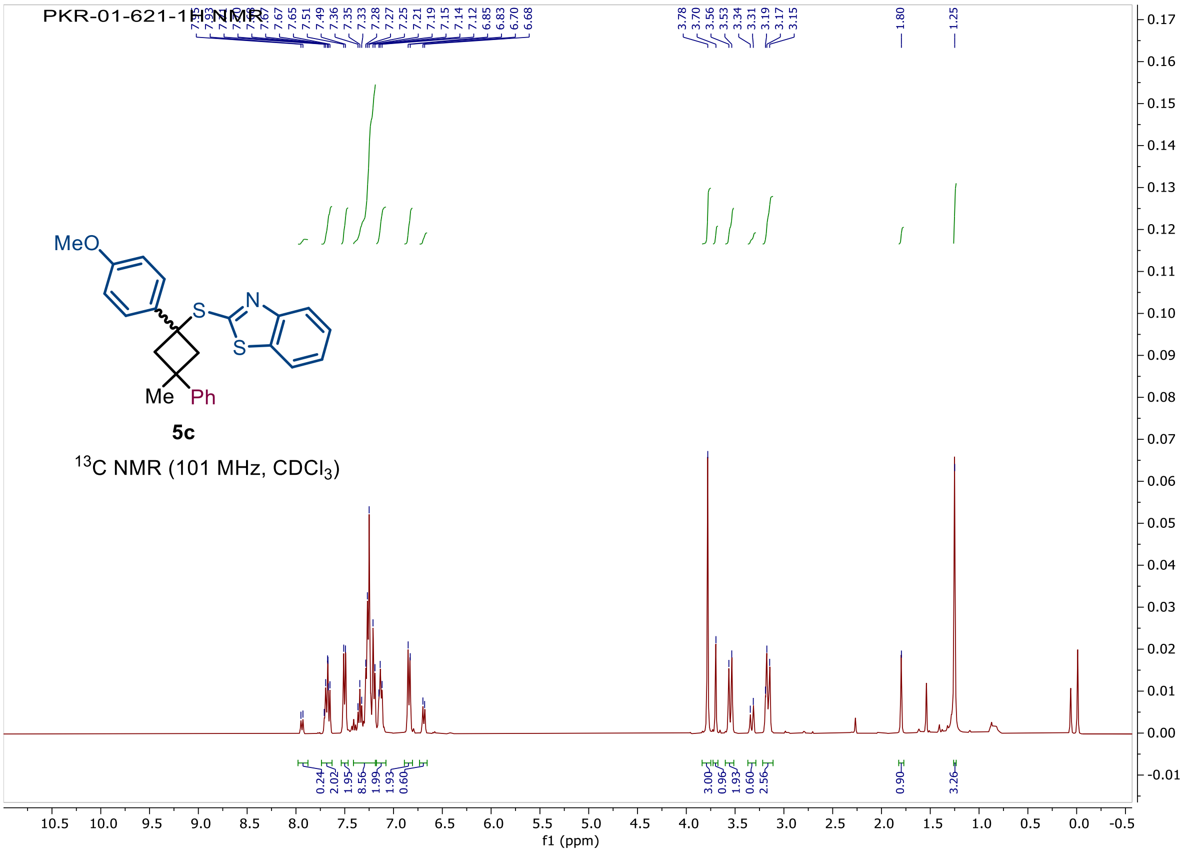
PKR-01-621-1H NMR
7.67 7.65 7.51 7.49 7.36 7.35 7.33 7.28 7.27 7.25 7.21 7.19 7.15 7.14 7.12 6.85 6.83 6.70 6.68
3.78 3.70 3.56 3.53 3.34 3.31 3.19 3.17 3.15
1.80
1.25
MeO
S
N
S
Me
Ph
5c
¹³C NMR (101 MHz, CDCl₃)
0.24 2.02 1.95 8.56 1.99 1.93 0.60
3.00 0.96 1.93 0.60 2.56
0.90
3.26
f1 (ppm)
